# Accessible, uniform protein property prediction with a scikit-learn based toolset AIDE

**DOI:** 10.1093/bioinformatics/btaf544

**Published:** 2025-09-24

**Authors:** Evan Komp, Kristoffer E Johansson, Nicholas P Gauthier, Japheth E Gado, Kresten Lindorff-Larsen, Gregg T Beckham

**Affiliations:** Renewable Resources and Enabling Sciences Center, National Renewable Energy Laboratory, Golden Colorado, CO 80401, United States; Agile BioFoundry, Emeryville, CA 94608, United States; Linderstrøm-Lang Centre for Protein Science, Section for Biomolecular Sciences, Department of Biology, University of Copenhagen, Copenhagen, Denmark; Department of Systems Biology, Harvard Medical School, Boston, MA 02115, United States; Department of Data Sciences, Dana-Farber Cancer Institute, Boston, MA 02115, United States; Renewable Resources and Enabling Sciences Center, National Renewable Energy Laboratory, Golden Colorado, CO 80401, United States; Linderstrøm-Lang Centre for Protein Science, Section for Biomolecular Sciences, Department of Biology, University of Copenhagen, Copenhagen, Denmark; Renewable Resources and Enabling Sciences Center, National Renewable Energy Laboratory, Golden Colorado, CO 80401, United States; Agile BioFoundry, Emeryville, CA 94608, United States

## Abstract

**Summary:**

Protein property prediction via machine learning with and without labeled data is becoming increasingly powerful, yet methods are disparate and capabilities vary widely over applications. The software presented here, “Artificial Intelligence Driven protein Estimation (AIDE)”, enables instantiating, optimizing, and testing many zero-shot and supervised property prediction methods for variants and variable length homologs in a single, reproducible notebook or script by defining a modular, standardized application programming interface (API), i.e. drop-in compatible with scikit-learn transformers and pipelines.

**Availability and implementation:**

AIDE is an installable, importable python package inheriting from scikit-learn classes and API and is installable on Windows, Mac, and Linux. Many of the wrapped models internal to AIDE will be effectively inaccessible without a GPU, and some assume CUDA. The newest stable, tested version can be found at https://github.com/beckham-lab/aide_predict and a full user guide and API reference can be found at https://beckham-lab.github.io/aide_predict/. Static versions of both at the time of writing can be found on Zenodo.

## 1 Introduction

Proteins achieve diverse functions across binding, transport, and catalysis, and they are being leveraged by humans for health and industrial applications (Ellis *et al.* 2021, Victorino de silva Amatto *et al.* 2022, Ebrahimi and Samanta 2023). The protein design space is nearly infinite and mostly not functional, making it difficult to navigate (Bank 2022, Ding *et al.* 2024, Johnston *et al.* 2024, Park *et al.* 2024). Researchers inform protein design by a variety of methods, from first principles to data-driven methods, and introduce variation targeting favorable properties with more likelihood than by random mutation (Madhavan *et al.* 2021). For the purposes of the software presented here, we reduce scope to methods whose parameters are determined by fitting to data, e.g. machine learning (ML).

ML has found use in protein property prediction (PPP) in a many applications (Ferruz *et al.* 2023, Notin *et al.* 2024), broadly categorized as supervised ML (SML), where parameters are determined to minimize error of model output on a set of examples with known experimental labels, and “zero-shot” (ZS) learning, where the model parameters are determined for a related task that does not require pre-determined labels and whose outputs may correlate to the property of interest (Hopf *et al.* 2019, Frazer *et al.* 2021, Notin *et al.* 2022a, 2022b, 2023b, Ding *et al.* 2024). Initially, proteins could be represented to ML algorithms via fixed-length vectors such as amino acid distributions, K-mer counts, structural surface properties, or binary and independent “one-hot” encodings of amino acids (Chen *et al.* 2022). These features can be given to traditional SML algorithms such as linear models, tree methods, or Gaussian processes, etc (Block *et al.* 2006, Fox *et al.* 2007, Romero *et al.* 2013, Wu *et al.* 2019). The introduction of self-supervised training in protein language and structure models (PLMs) has expanded the number and diversity of available methods. Such models can operate directly on potentially multiple intrinsic data modes of the protein, and the “learned” embeddings can be used to represent or predict properties of the system (Meier *et al.* 2021, Elnaggar *et al.* 2022, Hayes et al. 2025). Many examples of global predictors of, e.g. stability have been trained on this principle (Gado *et al.* 2025, Komp *et al.* 2023, Pudžiuvelytė *et al.* 2024). The reconstruction likelihoods they were initially trained for can also sometimes correlate with a property of interest, expanding available ZS methods beyond alignment scores (Eddy 2011, Meier *et al.* 2021, Marquet *et al.* 2022).

While the increase in quantity and capability of PPP methods is encouraging, there are nuances among available tools such as the required inputs, whether they be purely sequence-based, evolutionary homologs, or other data modes (Hopf *et al.* 2019, Frazer *et al.* 2021, Dauparas *et al.* 2022, Su *et al.* 2024). Given that there is not one method that performs best for every task (e.g. SaProt, which performs best on average in the ProteinGym deep mutational scan ZS benchmarks as of August 2024, is not in the top 5 scorers for 141/217 benchmarks (Notin *et al.* 2023a, Su *et al.* 2023)), researchers must attempt to install many different tools and run separate scripts to determine the right tool for their application.

**Figure 1.**
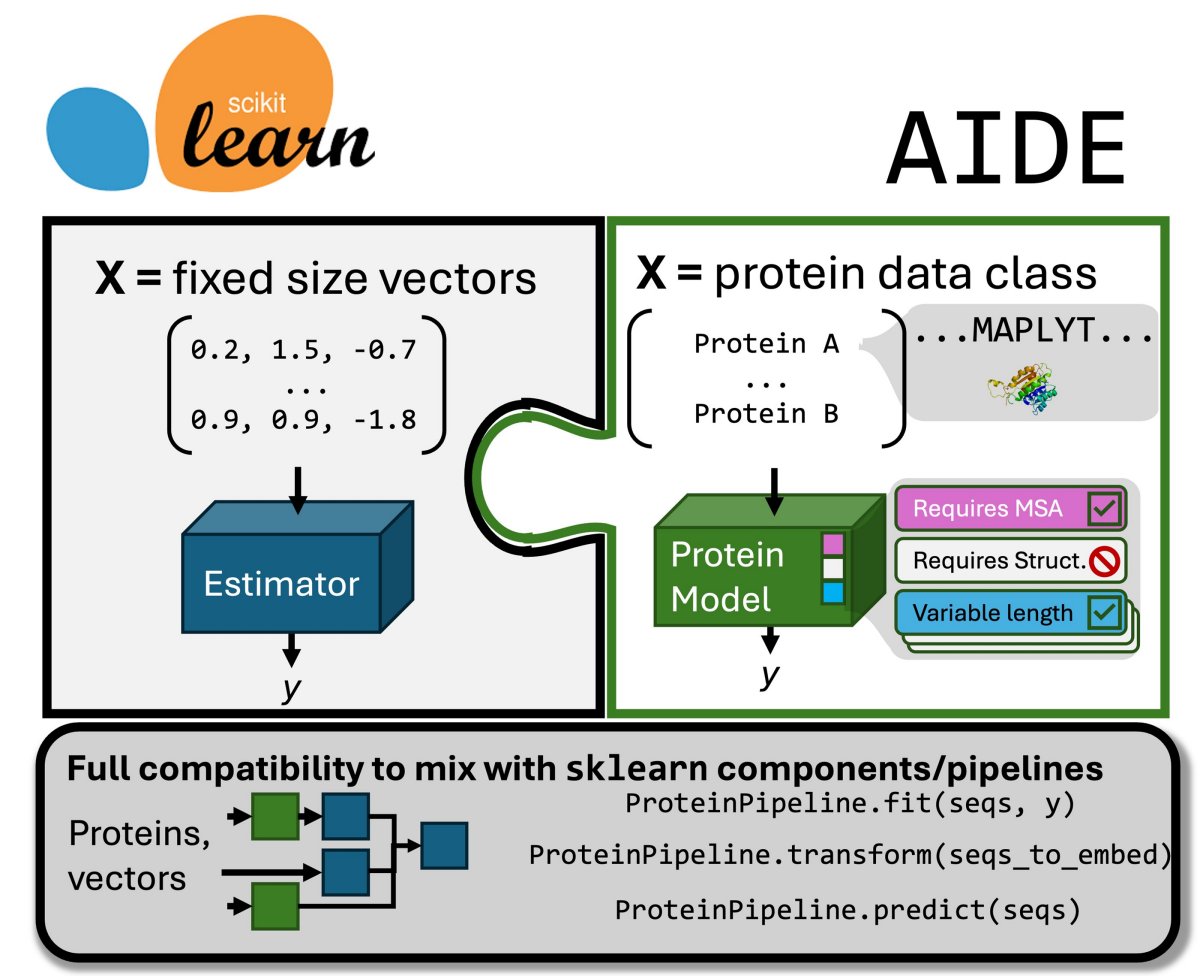
Overview of AIDE. The package supplies a scikit-learn model subclass that operates on a dataclass representing proteins as opposed to numpy matrices, and number of mixin protocols for protein models to adopt common behavior and compatibility checks for user data. These models are drop-in compatible with existing scikit-learn classes and pipelines, allowing for multistep processes to be defined within a single script in a reproducible way like one would for a traditional scikit-learn application.

To increase accessibility, reproducibility, and enable comparisons of PPP testing and utilization, here we present a software package Artificial Intelligence Driven protein Estimation (AIDE). The framework defines a scikit-learn derived application programming interface (API) for models in both the SML and ZS categories (Fig. 1) (Pedregosa *et al.* 2011). We provide a modular, generalized base class for protein models, and wrote protocols for the various model nuances that can be accommodated for new models using Mixins (Bourque *et al.* 2002). This means that wrapping a given PPP method is rigorously defined and once done, it can be can be imported within a single script or notebook along with other methods, and executed using the same scikit-learn API. Furthermore, disparate models with various dependencies are kept modular, calling subprocesses when necessary, instead of in a single “mega function”, meaning that the library can continue to expand without a growing difficulty of user installation and whole-codebase overhauls. We provide, in a single notebook each, exemplary applications of the library to (1) benchmark ZS models, embedders, and nonlinear models against an epistatic combinatorial dataset, and (2) optimize a supervised prediction pipeline of polyethylene terephthalate hydrolase (PETase) homolog activity at low pH that can be loaded and executed in two lines of code like any scikit-learn pipeline. The software is extensively unit tested with continuous integration at >80% coverage. This software provides researchers a “virtual toolbox” for PPP and we hope increases the visibility of any models added by the community. The full API documentation, roadmap, and user guide is in the Supplementary Data User Guide (UG), available as supplementary data at *Bioinformatics* online, and at https://beckham-lab.github.io/aide_predict/ with code at: https://github.com/beckham-lab/aide_predict.

## 2 Challenges with the current ecosystem

We suggest that the following issues are faced today in the ecosystem of PPP tools, whether for individual methods use or in tool aggregation:

1. There is no localized repository of methods that a researcher can use to sandbox their task.
2. Embedding and ZS methods in the literature use disparate, bespoke interfaces.
3. Existing tools do not typically adhere to common software engineering principles associated with long lasting, evolving toolsets, such as unit tests.

These challenges mean that when trying multiple methods on a new application, the use of each must be tracked in natural language, without the same reproducibility as one has with a repeatable script. A researcher thus must learn the specific format that each tool requires, and is less likely to find bugs, making community development harder wherein a contributor must first learn a previous researcher’s coding style while simultaneously trying to read the algorithm. Bespoke code also makes extending the toolset difficult from a dependency-management perspective; adding a new method likely involves whole-codebase updates and additional installation dependencies in the top-level module.

There have already been efforts to alleviate parts of the three noted cruxes, but to our knowledge, a single method does not address them all (Dallago *et al.* 2021, Siedhoff *et al.* 2021, Wittmann *et al.* 2021, Chen *et al.* 2022, Sequeira *et al.* 2022, Notin *et al.* 2023a, Funk *et al.* 2024, Su *et al.* 2024). While these toolsets are all useful, very few exhibit high test coverage, if present at all. Most importantly, each defines a unique API and environment that must be reworked to extend the toolset.

## 3 Software descriptor

Notably, the disparity in code architecture and model internals is the same challenge overcome by scikit-learn, a unifying ML package where a user can, e.g. test out a ridge regressor and a random forest regressor—very different algorithms—on their data in the same way (Pedregosa *et al.* 2011). Scikit-learn leverages an intuitive init-fit-transform-score API as a gateway to ML, and nearly all ML practitioners use it. The presented software operates on a protein data structures and protein models just as scikit-learn does with fixed-length vectors (Fig. 1). Given this, AIDE is built on and drop-in compatible with scikit-learn pipelines.

### 3.1 Core classes

The software centers around two types of classes: data structures like ProteinSequence, ProteinSequences, and ProteinStructure, as well as model classes deriving from ProteinModelWrapper. The data structures allow for quick input/output of protein structures, sequences, and multiple sequence alignments. They also expose helpful methods such as inducing mutations, checking for differences between two sequences, generating mutagenesis libraries to pass to models, and attributes such as whether it is gap-containing. These data structures are analogous to an X array in standard scikit-learn applications. The ProteinModelWrapper accepts these ProteinSequences as inputs. It can act as a transformer or a regressor depending on how it is inherited, and leverages the expected API of parameters to initialization, then fit-transform-score. Wrapping a new method into AIDE as a ProteinModelWrapper requires defining only the core behavior of the model, while existing Mixins can be added to handle commonalties exhibited in the literature, see the UG Section 2.5, available as supplementary data at *Bioinformatics* online. The Mixins check and handle the inputs and outputs for the expected format, such that the data are already converted to a format compatible for the tool. Definitions must be done only once, then the model can be used in scripts and notebooks. Example 6 in the UG Section 2.2, available as supplementary data at *Bioinformatics* online, depicts a wrapped model and UG Section 2.12, available as supplementary data at *Bioinformatics* online, provides a guide to doing so. Some common Mixins that a particular model may use are:

- RequiresMSAForFitMixin when mixed in for a bespoke model, AIDE will check that ProteinSequences coming into the fit method are aligned, and if not, it will attempt to align them.
- RequiresStructureMixin AIDE will ensure that incoming ProteinSequences has associated structures falling back to a wild-type structure if present and issue a warning.
- RequiresWTToFunctionMixin AIDE will require that a wild-type sequence be available within the model.

A full list of current Mixins is given in the UG Section 2.5.7, available as supplementary data at *Bioinformatics* online. Undoubtedly, more Mixins will be required as the PPP field progresses, and we focused on modularity to accommodate this.

### 3.2 Supporting functions

Various utilities are provided to both expand functionality and reduce user friction. For example, functions are provided to check which ProteinModels are installed, and which are compatible with the available data from the user. Others sample multiple sequence alignments (MSAs), align sequences in place, and map structures. We also provide tools for common tasks on either side of PPP, like predicting structures with Soloseq, creating MSAs with MMseqs2, or sampling designed sequences to maximize predictions with BADASS (Steinegger and Söding 2017, Ahdritz *et al.* 2024, Gomez-Uribe *et al.* 2024).

### 3.3 Currently supported tools and installation

In AIDE, we separate methods into their own modules and implement checks for whether they are available based on their dependencies. The PPP methods currently wrapped in the API are separated into three categories: those that are functional with only a lightweight dependency list of the base package, those that require additional pip installable dependencies, and those that require more setup such as cloning a repository and following installation instructions. This avoids requiring users to run environment integrations to use multiple toolsets simultaneously and instead install just the modules they would like, and a single package environment does not become too heavy to solve. For example, hidden Markov models (HMMs) and one-hot encodings are accessible with the base module, while ESM and a few others require the addition of PyTorch (Eddy 2011, Paszke *et al.* 2019, Meier *et al.* 2021). To access them, the user installs the additional “requirements-transformers.txt”. For EVE, the user is instructed to setup the original work’s EVE environment and set an environment variable (Frazer *et al.* 2021). To use EVE, AIDE then calls it as a subprocess, such that compatibility between other AIDE models and EVE is not an issue.

The currently available methods to the user based on their environment and their data can be checked from within AIDE via a function call. The full list of currently wrapped tools is given in Table 1 (Eddy 2011, Hopf *et al.* 2017, Frazer *et al.* 2021, Meier *et al.* 2021, Rao *et al.* 2021, Marquet *et al.* 2022, Su *et al.* 2023, Blaabjerg *et al.* 2024). Note that this is an initial list to serve as a proof of concept, and we plan to add, along with any community contributors, additional models, and data modes.

**Table 1.** Currently wrapped tools in AIDE.

Class	Type	Dependencies	Description
OneHotProteinEmbedding	Emb	–	20 AA binary indicators for a fixed sequence length
OneHotAlignedEmbedding	Emb	–	20 AA + gap indicators of an MSA, new sequences are aligned to MSA
KMerEmbedding	Emb	–	K-mer counts of specified order
HMMWrapper	ZS	–	likelihood scores compared to a set of related sequences assuming independent column wise frequencies
EVMutationWrapper	ZS	Evcouplings	Hamiltonian scores of pairwise evolutionary couplings compared to a wild type sequence
ESM2Embeddings	Emb	Transformers	Position specific embeddings from ESM2 PLM
ESM2LikelihoodWrapper	ZS	Transformers	Masked, wild type, mutant marginal likelihood from ESM2
SaProtEmbedding	Emb	Transformers, foldseek	Position specific embeddings from sequence and structure tokens from SaProt PLM
SaProtLikelihoodWrapper	ZS	Transformers, foldseek	Masked, wild type, or mutant marginal likelihood of sequence tokens given sequence and structure
MSATransformerEmbedding	Emb	Transformers, fair-esm	Position specific embeddings conditioned on a whole or sampled MSA
MSATransformerLikelihoodWrapper	ZS	Transformers, fair-esm	Masked, wild type, or mutant marginal likelihood of sequence tokens given MSA tokens
VESPA	ZS	Vespa-effect	Single substitution mutation scores given conservation predictions of a model trained on PLM embeddings and MSAs
EVE	ZS	^a^	Variant affect predication via VAE trained on MSA
SSEmbedder	Emb	^a^	Structure constrained MSA language model final layer
SSEmbPredictor	ZS	^a^	Structure constrained MSA language model additive log-likelihood

a The tool requires building the original independent environment.

### 3.4 Unit tests

All base classes and modules were extensively unit tested. We cannot ensure that wrapped methods are tested properly; however, we ensure that while the interpreter is within AIDE, it is executing tested code, and that the wrapped methods evaluated on the ProteinGym benchmark produce outputs matching the reported score for the ENV_ECOLI (Notin *et al.* 2023a). As of September 2025, AIDE has >80% code test coverage.

## 4 Showcases

We provide two illustrative cases for using AIDE to efficiently compare ML strategies, with all of the typical tasks such as cross validation and hyperparameter optimization. We do not intend to highlight a particular scientific finding for these systems or claim that they extend to other systems, but rather highlight the work that can be accomplished with relatively little coding in a reproducible manner. See Appendices A and B, available as supplementary data at *Bioinformatics* online, for a notebook of the complete details and execution of each task.

### 4.1 Showcase 1: benchmarking on an epistatic combinatorial dataset

**Figure 2.**
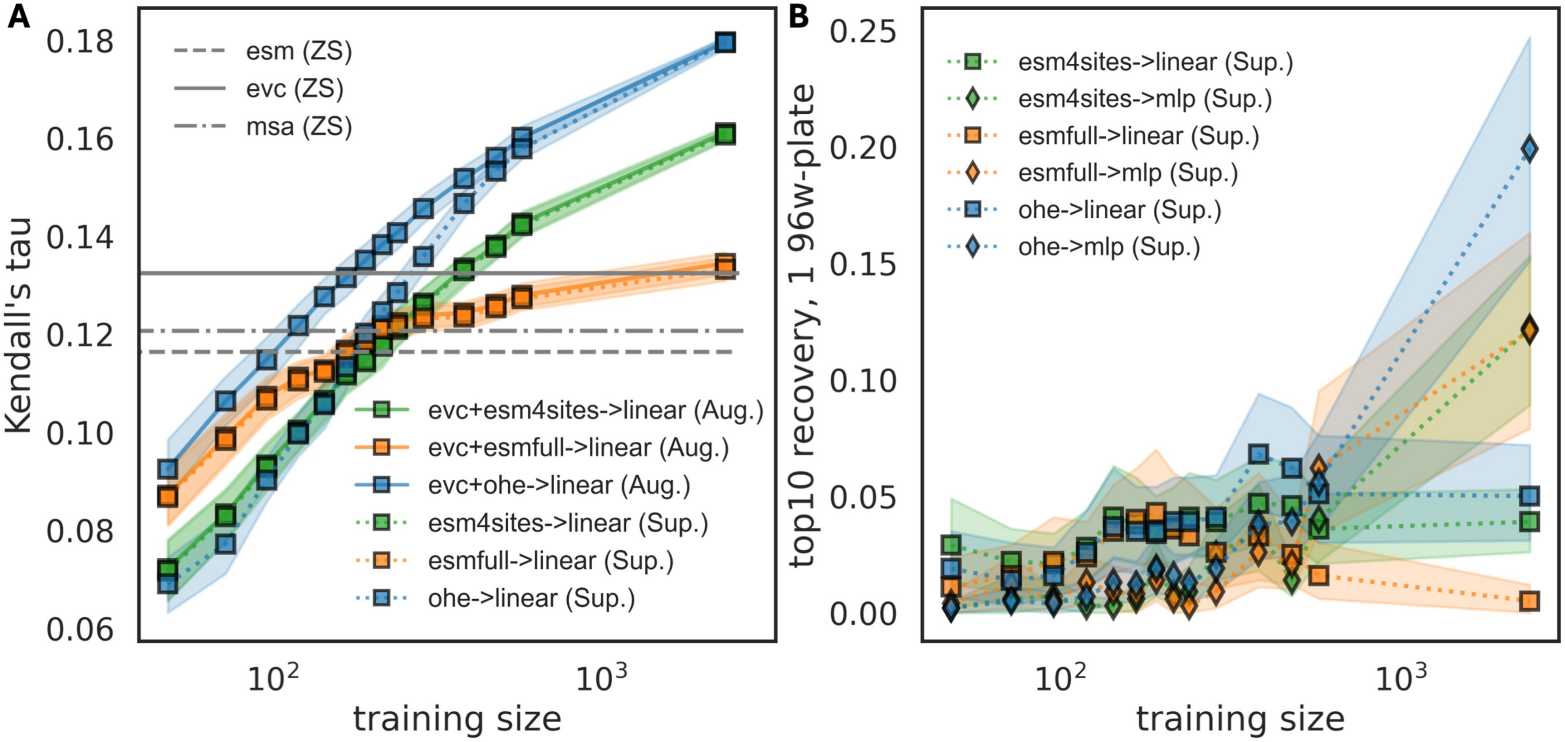
Performance of various models on four-site epistatic combinatorial dataset by Johnston *et al.* as a function of dataset size (Johnston *et al.* 2024). Embeddings for the supervised model include one-hot encoding (ohe), ESM2 mean pooling over the entire protein sequence (full), and only the four variable positions (four sites) (Meier *et al.* 2021). (A) Pure supervised only (“Sup.” dashed line with color) versus Zero Shot (ZS, grey) and zero shot augmented (“Aug.”, solid line with color) models with EVCouplings scores (Hopf *et al.* 2019). Augmentation improves performance of one-hot encoding at low training data but does not affect ESM based embedding and is negligible for >500 training points. (B) Top 10 recovery defined as the fraction of the 10 true best recovered in a final set of 96 chosen using linear versus nonlinear pure supervised models, where at about 1000 training examples, nonlinear models begin to significantly outperform linear ones.

We optimized and tested several prediction strategies on a recent, highly-epistatic, 4-site combinatorial enzyme activity dataset by Johnston *et al.* (2024). Similar to work by Hsu *et al.*, we tested “augmenting” ZS scorers with supervised predictors, and we include a few ZS scorers not explored in the original work, namely MSATransformer and ESM2 (Hsu *et al.* 2022, Meier *et al.* 2021, Hayes *et al.* 2025). We also test embedding strategies beyond one-hot encoding: ESM embeddings mean pooled over the whole sequence or just the four variable sites. Hyperparameter optimization was conducted for each strategy in five-fold cross validation. Embedders were defined and integrated into scikit-learn pipelines including feature and target scaling, principal component analysis, and predictor heads. We do this in a single jupyter notebook executed on a laptop with less than an 8-h runtime, which can be found in the repository or in PDF form in Appendix A, available as supplementary data at *Bioinformatics* online. The results of this comparison are shown in Fig. 2.

### 4.2 Showcase 2: PETase activity predictor for scraping natural sequences

**Figure 3.**
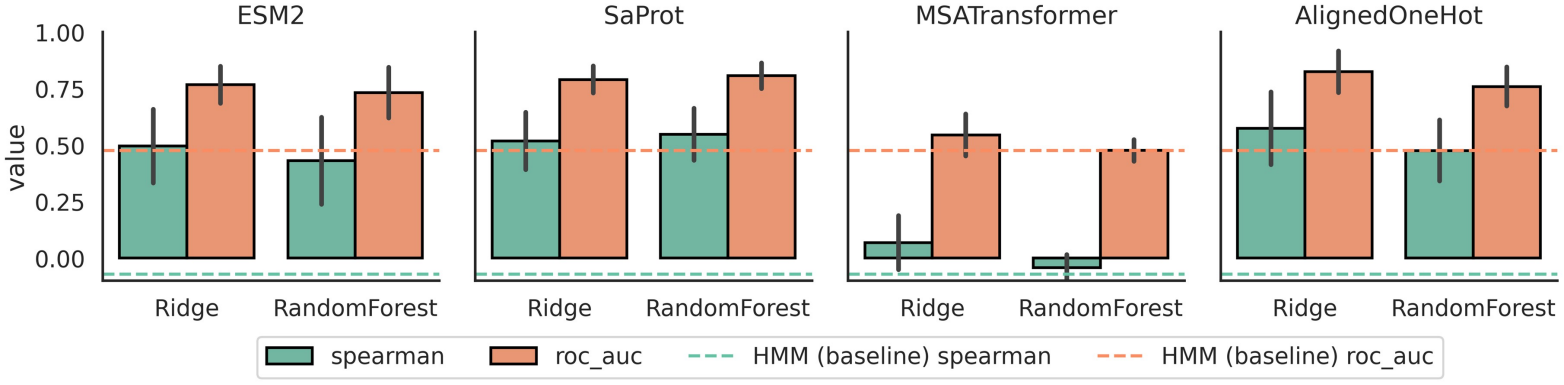
Performance of various combinations of embeddings strategy and predictor head for PETases homolog activity prediction. PET hydrolysis activity measured at pH = 5.5, 40°C (Norton-Baker *et al.* 2025). Horizontal lines are “null” model of using only an HMM score (cyan for spearman correlation to measured activity, salmon for area under the receiver operator curve “roc_auc” of nonzero measured activity). Null model is incapable of predicting active versus inactive PET homologs. Bars are five-fold CV scores after hyperparameter optimization of linear and nonlinear random forest models against four embedding strategies for PETase activity prediction at pH = 5.5, 40°C: ESM mean pooling, SaProt mean pooling, MSATransformer flattened embedding of query sequence, and one-hot encoding of a held out alignment (Meier *et al.* 2021, Rao *et al.* 2021, Su *et al.* 2024).

We recently conducted a large search for remote polyethylene terephthalate hydrolase (PETase) homologs to identify novel enzymes (Norton-Baker *et al.* 2025). Here, we use this dataset to produce an activity predictor for future searches. We tested several embedding strategies: one-hot encoding of PETase alignments, ESM2 pooled embeddings, SaProt (structure aware) embeddings using AF2 predicted structures, and MSATransformer embeddings using an MSA of 61 known PETases (Meier *et al.* 2021, Rao *et al.* 2021, Su *et al.* 2023). We tested these with a linear model head and a nonlinear random forest head. For each combination, we conducted five-fold cross validation and random hyperparameter optimization. The results are compared to an HMM score built from known PETases (Fig. 3). This is conducted in a single jupyter notebook on a laptop in less than 4-h runtime, which can be found in the code repository. A PDF of the notebook is also provided in Appendix B, available as supplementary data at *Bioinformatics* online.

### 4.3 Examples

In addition to the showcases, we provide several executable code examples to highlight the conciseness of readability of the API, given in the “demo” folder of the code repository and in the UG Section 2.2, available as supplementary data at *Bioinformatics* online, including checking which models are compatible with user data, *in silico* mutagenesis, training a global supervised WT predictor, combining ZS and supervised methods into a single scikit-learn pipeline, and wrapping a new method into the API.

## 5 Conclusion

We have written a set of scikit-learn-derived python classes that unify the use of protein models for both embedding and ZS prediction. Models are loaded and called in the same init-fit-transform-score API as users are familiar with in scikit-learn (Pedregosa *et al.* 2011). Common workflows associated with input and output requirements were codified, making adding new models straightforward and defined. This enables rapid comparison to user data to determine which methods are compatible, and execution of them in reproducible scripts. The open-source package is unit tested, and we anticipate will enable creating and testing reproducible protein property prediction pipelines more accessible.

## Supplementary Material

btaf544_Supplementary_Data

## Data Availability

See Github (https://github.com/beckham-lab/aide_predict/) to access the latest version software, or on Google Colab (https://colab.research.google.com/drive/1baz4DdYkxaw6pPRTDscwh2o-Xqum5Krp). The state of the software at the time of writing is given in version v1.1.03 on Zenodo (Komp and Beckham 2025). A full user guide and API reference can be found at https://beckham-lab.github.io/aide_predict/. Data files for Figs 2 and 3 are available in the Supplementary Data, available as supplementary data at *Bioinformatics* online.
