# Supplementary material for: Accessible, uniform protein property prediction with a scikit-learn based toolset AIDE
Source: Bioinformatics. 2025 Sep 24;41(10):btaf544. doi: 10.1093/bioinformatics/btaf544 (PMC12553329; doi:10.1093/bioinformatics/btaf544)
Supplement: btaf544_Supplementary_Data [file btaf544_supplementary_data.zip › UserGuide.pdf]

aide

Release 1.1.01

Evan Komp, Gregg T. Beckham

Aug 28, 2025

GETTING STARTED

1 AIDE **1**

1.1 API examples: 2
1.2 Available Tools 2
1.3 Helper tools 5
1.4 Installation 5
1.5 Installation of additional modules 6
1.6 Tests 7
1.7 Citations and Acknowledgements 7
1.8 License 8

2 User Guide **9**

2.1 Installing AIDE 9
2.2 API examples 11
2.3 Data Structures 17
2.4 Model Compatibility 22
2.5 ProteinModelWrapper 25
2.6 Zero-Shot Prediction 32
2.7 Supervised Learning 37
2.8 Saturation Mutagenesis 38
2.9 Building ML Pipelines 40
2.10 Caching Model Outputs 41
2.11 Position-Specific Models 42
2.12 Contributing Models to AIDE 44
2.13 Structure Prediction with SoloSeq 47
2.14 Generating MSAs with MMseqs2 49
2.15 Protein Optimization with BADASS 51
2.16 Roadmap 55
2.17 Resource testing 56
2.18 aide_predict 57

3 Indices and tables **197**

Python Module Index **199**

CHAPTER

ONE

AIDE

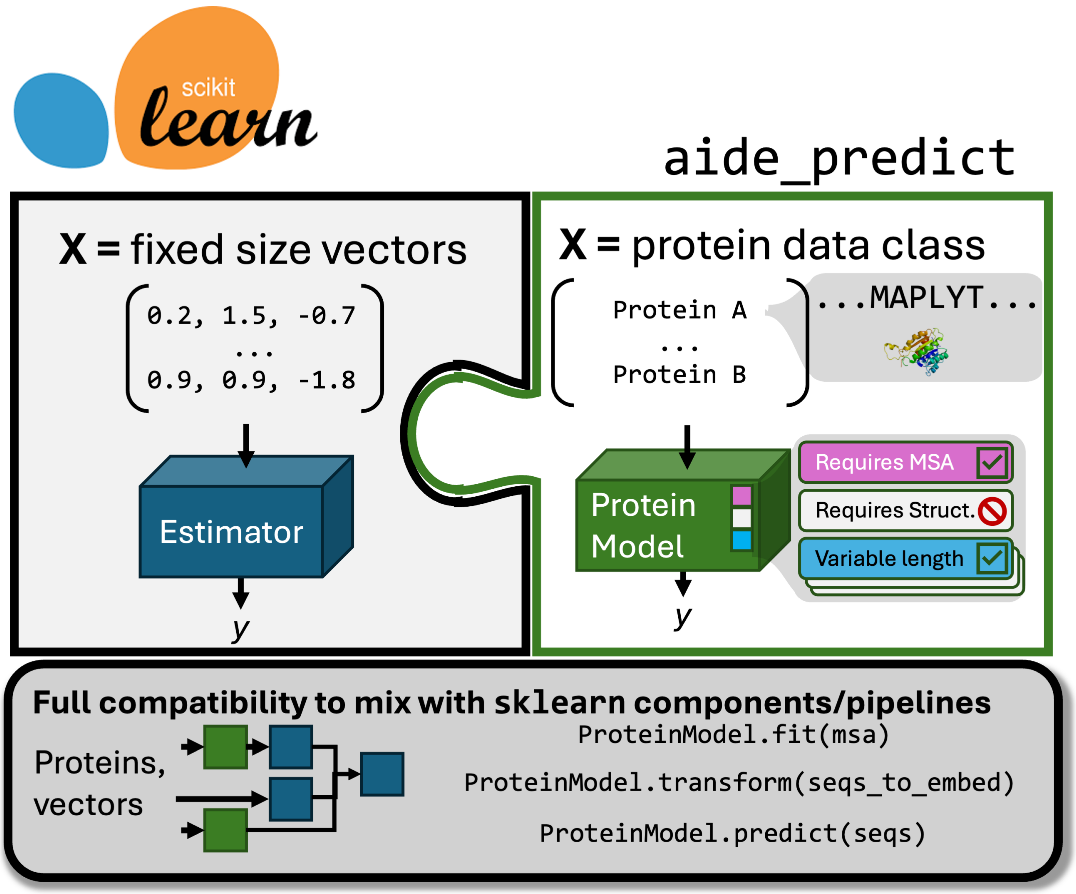

Authors: Evan Komp, Gregg T. Beckham **Associated manuscript**: TODO

This repository serves fundementally to increase the accessibility of protein engineering tasks that fall into the following catagory:

$$\hat{y} = f(X)$$

Here, X is a set of proteins, eg. their sequence and optionaly structure. y is a property of the protein that is difficult to measure, such as binding affinity, stability, or catalytic activity. $\hat{y}$ is the predicted value of y given X.

Existing models f in the literature are varied, and a huge amount of work has gone into designing clever algorithms that leverage labeled and unlabeled data. For example, models differ in the following ways (non exhaustive):

- Some require supervised labels y, while others do not
- Unsupervised models can be trained on vast sets of sequences, or MSAs of the related proteins
- Models exist to predict the effect of mutations on a wild type sequence, or to globally predict protein properties
- Some models incorporate structural information
- Some models are pretrained
- Some models are capable of position specific predictions, which can be useful for some tasks

The variety an nuance of each of these means that each application is a bespoke, independent codebase, and are generally inaccessible to those with little or no coding exprience. Some applications alleviate the second problem by hosting web servers. Add to this problem is a lack of standardization in API across applications, where individual code bases can be extremely poorly documented or hard to use due to hasty development to minimize time to publication.

The goals of this project are succinctly as follows:

- **Create a generalizable, unittested, API for protein prediction tasks that is compatible with scikit learn**. This API will allow those who are familiar with the gold standard of ML libraries to conduct protein prediction tasks in much the same way you'd see on an intro to ML Medium article. Further, it makes it much easier for bespoke strategies to be accessed and compared; any new method whose authors wrap their code in the API are easily accessed by the community without spending hours studying the codebase.
- **Use API components to create a DVC tracked pipeline for protein prediction tasks**. This pipeline will allow for those with zero software experience to conduct protein prediction tasks with a few simple commands. After (optionally) editting a config file, inputing their training data and their putative proteins, they can train and get predictions as simply as executing `dvc repro`.

1.1 API examples:

AIDE examples look and feel like canonical sklearn tasks/code. Aide is not limited to only combinatorial/mutant data or global wt sequence predictors: it is meant for all protein property prediction tasks. The complete API and user guide is available at: https://beckham-lab.github.io/aide_predict/

See also the `demo` folder for some executable examples. Also see the colab notebook to play with some if its capabilities in the cloud. Finally, checkout the notebooks in `showcase` where we conduct two full protein predictions optimization and scoring tasks on real data that are greater than small example sets.

1.2 Available Tools

You can always check which modules are installed/available to you by running `get_supported_tools()`. The following is a list of tools that are available. Models marked with a * require additional dependencies or envionments to be installed, see `Installation`

1.2.1 Data Structures and Utilities

- Protein Sequence and Structure data structures
- `StructureMapper` - A utility for mapping a folder of PDB structures to sequences

1.2.2 Prediction Models

1. HMM (Hidden Markov Model)
    - Computes statistics over matching columns in an MSA, treating each column independantly but allowing for alignment of query sequences before scoring
    - Requires MSA for fitting
    - Can handle aligned sequences during inference
2. EVMutation*
    - Computes pairwise couplings between AAs in an MSA for select positions well represented in the MSA, variants are scored by the change in coupling energy.
    - Requires MSA for fitting
    - Requires wild-type sequence for inference
    - Requires fixed-length sequences
    - Requires additional dependencies (see `requirements-evmutation.txt`)
3. ESM2 Likelihood Wrapper*
    - Pretrained PLM (BERT style) model for protein sequences, scores variants according to masked, mutant, or wild type marginal likelihoods. Mutant marginal computes likelihoods in the context of the mutant sequence, while masked and wild type marginal compute likelihoods in the context of the wild type sequence. These methods are apprximations of the joint likelihood.
    - Can handle aligned sequences
    - Requires additional dependencies (see `requirements-transformers.txt`)
4. SaProt Likelihood Wrapper*
    - ESM except using a size 400 vocabulary including local structure tokens from Foldseek's VAE. The authors only used Masked marginal, but we've made Wild type, Mutant, and masked marginals avialable.
    - Requires fixed-length sequences
    - Uses WT structure if structures of sequences are not passed
    - Requires additional dependencies:
        - `requirements-transformers.txt`
5. MSA Transformer Likelihood Wrapper*
    - Like ESM but with a transformer model that is trained on MSAs. The variants are placed at the top position in the MSA and scores are computed along that row. Wild type, Mutant, and masked marginals avialable.
    - Requires MSA for fitting
    - Requires wild-type sequence during inference
    - Requires additional dependencies (see `requirements-fair-esm.txt`)
6. VESPA*

- Conservation head model trained on PLM embeddings and logistic regression used to predict if mutation is detrimental.
- Requires wild type, only works for single point mutations
- Requires fixed-length sequences
- Requires additional dependencies (see `requirements-vespa.txt`)

7. EVE*
   - VAE trained on MSA, learns conditional distribution of AA. Latent space tends to be bimodal for deleterious vs neutral mutations.
   - Requires MSA for fitting
   - Requires fixed-length sequences
   - Requires independant EVE environment, see Installation.
8. SSEmb*
   - MSATransformer with attention constrained by 3D structure contacts, combined with Graph Neural network on the structure
   - Requires MSA for fitting
   - Requires wild-type sequence during inference
   - Requires fixed-length sequences
   - Requires Structure
   - Requires independant SSEmb environment, see Installation.

1.2.3 Embeddings for Downstream ML

1. One Hot Protein Embedding
   - Columnwise one hot encoding of amino acids for a fixed length set of sequences
   - Requires fixed-length sequences
   - Position specific
2. One Hot Aligned Embedding
   - Columnwise one hot encoding including gaps for sequences aligned to an MSA.
   - Requires MSA for fitting
   - Position specific
3. Kmer Embedding
   - Counts of observed amino acid kmers in the sequences
   - Allows for variable length sequences
4. ESM2 Embedding*
   - Pretrained PLM (BERT style) model for protein sequences, outputs embeddings for each amino acid in the sequece from the last transformer layer.
   - Position specific
   - Requires additional dependencies (see `requirements-transformers.txt`)

5. SaProt Embedding*
   - ESM except using a size 400 vocabulary including local structure tokens from Foldseek's VAE. AA embeddings from the last layer of the transformer are used.
   - Position specific
   - Requires additional dependencies:
     - `requirements-transformers.txt`
     - `foldseek` executable must be available in the PATH
6. MSA Transformer Embedding*
   - Like ESM but with a transformer model that is trained on MSAs. The embeddings are computed for each amino acid in the query sequence in the context of an existing MSA
   - Requires MSA for fitting
   - Requires fixed-length sequences
   - Requires additional dependencies (see `requirements-fair-esm.txt`)
7. SSEmb*
   - MSATransformer with attention constrained by 3D structure contacts, combined with Graph Neural network on the structure
   - Requires Per sequence msa and structure
   - Position specific
   - See installation for additional dependencies

Each model in this package is implemented as a subclass of `ProteinModelWrapper`, which provides a consistent interface for all models. The specific behaviors (e.g., requiring MSA, fixed-length sequences, etc.) are implemented using mixins, making it easy to understand and extend the functionality of each model.

1.3 Helper tools

The tools within the API often require somewhat expensive input information such as MSA and structures. We provide high level interfaces to predict structures and compute MSAs, see the user guide.

1.4 Installation

```
conda env create -f environment.yaml
pip install .
```

1.5 Installation of additional modules

Tools that require additional dependancies can be installed with the corresponding requirements file. See above for those files. For example, to access VESPA:

```
pip install -r requirements-vespa.txt
```

Some tools were deemed to heavy in terms of their environment to be included as a pip module. These require manual setup, see below.

1.5.1 Installation of EVE

To access the EVE module, first clone the repo (NOT inside of AIDE):

```
git clone https://github.com/OATML/EVE.git
```

IMPORTANT: set the environment variable `EVE_REPO` to the path of the cloned repo. This is used by AIDE to import EVE modules as it is not installable.

Build a new conda environment according to instructions/`.yaml` file there.

We recommend testing that the environment is set up correctly and that the package is using any GPUs but running their example script and observing the log.

IMPORTANT: set the environment variable `EVE_CONDA_ENV` to the name of the conda environment you created for EVE. This is used by AIDE to activate the EVE environment.

Confirm AIDE now has access to the EVE module:

```
from aide_predicts import get_supported_tools
get_supported_tools()
```

1.5.2 Installation of SSEmb

To access the SSEmb module, first clone the repo (NOT inside of AIDE):

```
git clone https://github.com/KULL-Centre/_2023_Blaabjerg_SSEmb
```

IMPORTANT: set the environment variable `SSEMB_REPO` to the path of the cloned repo. This is used by AIDE to import SSEmb modules as it is not installable.

Build a new conda environment:

```
conda create -n ssemb_env python=3.11 pytorch=2.3 scipy scikit-learn pandas fair-esm==2.
↪0.0 biopython==1.79 openmm==8.0 pdbfixer==1.8 pyyaml matplotlib mpl-scatter-density
conda activate ssemb_env
pip install torch_scatter torch_cluster
```

IMPORTANT: set the environment variable `SSEMB_CONDA_ENV` to the name of the conda environment you just created. This is used by AIDE to activate the SSEmb environment.

Download the model weights. This must be conducted in the root directory of the SSEmb repo:

```
wget https://zenodo.org/records/12798019/files/weights.tar.gz
tar -zxf weights.tar.gz
```

Confirm AIDE now has access to the SSEmb module:

```
from aide_predicts import get_supported_tools
get_supported_tools()
```

Its also probably worth running this test script to make sure it does not error:

```
python tests/test_not_base_models/test_ssemb_pred.py
```

1.6 Tests

Continuous integration only runs base module tests, eg. `pytest -v -m "not slow and not optional"`

Additional tests are availabe to check the scientific output of wrapped models, that they meet the expected values, such as:

- Score of ESM2 log liklihood, MSATransformer, SaProt, VESPA, EVE against ENVZ_ECOLI_Ghose benchmark of ProteinGym
- run with `pytest -v -m tests/not_base_models`

1.7 Citations and Acknowledgements

No software or code with viral licenses was used in the creation of this project.

The following deserve credit as they are either directly wrapped within AIDE, serve as code inspiration (noted in modules when necessary), or are used for testing:

1. Frazer, J. et al. Disease variant prediction with deep generative models of evolutionary data. Nature 599, 91–95 (2021).
2. Hopf, T. A. et al. The EVcouplings Python framework for coevolutionary sequence analysis. Bioinforma. Oxf. Engl. 35, 1582–1584 (2019).
3. Notin, P. et al. Tranception: protein fitness prediction with autoregressive transformers and inference-time retrieval. Preprint at https://doi.org/10.48550/arXiv.2205.13760 (2022).
4. Rao, R. et al. MSA Transformer. 2021.02.12.430858 Pre-print at https://doi.org/10.1101/2021.02.12.430858 (2021).
5. Hopf, T. A. et al. Mutation effects predicted from se-quence co-variation. Nat. Biotechnol. 35, 128–135 (2017).
6. Hsu, C., Nisonoff, H., Fannjiang, C. & Listgarten, J. Learning protein fitness models from evolutionary and assay-labeled data. Nat. Biotechnol. 40, 1114–1122 (2022).
7. Meier, J. et al. Language models enable zero-shot prediction of the effects of mutations on protein func-tion. Preprint at https://doi.org/10.1101/2021.07.09.450648 (2021).
8. Verkuil, R. et al. Language models generalize beyond natural proteins. 2022.12.21.521521 Preprint at https://doi.org/10.1101/2022.12.21.521521 (2022).
9. Su, J. et al. SaProt: Protein Language Modeling with Structure-aware Vocabulary. 2023.10.01.560349 Preprint at https://doi.org/10.1101/2023.10.01.560349 (2023).
10. Marquet, C. et al. Embeddings from protein language models predict conservation and variant effects. Hum Genet 141, 1629–1647 (2022).
11. Eddy, S. R. Accelerated Profile HMM Searches. PLOS Computational Biology 7, e1002195 (2011).

12. Pedregosa, F. et al. Scikit-learn: Machine Learning in Python. MACHINE LEARNING IN PYTHON.
13. Notin, P. et al. ProteinGym: Large-Scale Benchmarks for Protein Fitness Prediction and Design.
14. Blaabjerg, L.M., Jonsson, N., Boomsma, W. et al. SSEmb: A joint embedding of protein sequence and structure enables robust variant effect predictions. Nat Commun 15, 9646 (2024). https://doi.org/10.1038/s41467-024-53982-z

1.8 License

This project is licensed under the *MIT License*.

CHAPTER

TWO

USER GUIDE

2.1 Installing AIDE

AIDE is designed with a modular architecture to minimize dependency conflicts while providing access to a wide range of protein prediction tools. The base package has minimal dependencies and provides core functionality, while additional components can be installed based on your specific needs.

2.1.1 Quick Install

The package is not currently available on PyPI, please clone the repo:

```
git clone https://github.com/beckham-lab/aide_predict
```

For basic functionality, simply install AIDE using:

```
# Create and activate a new conda environment
conda env create -f environment.yaml

# Install AIDE
pip install .
```

2.1.2 Supported Tools by Installation Level

AIDE provides bespoke embedders and predictors as additional modules that can be installed. These fall into three categories, with environment weight in mind: those available in the base package, those that can be installed with minimal additional pip dependencies, and those that should be built as an independant environment.

Base Installation

The base installation provides:

- Core data structures for protein sequences and structures
- Sequence alignment utilities
- One-hot encoding embeddings
- K-mer based embeddings
- Basic Hidden Markov Model support
- mmseqs2 MSA generation pipeline

Minor Pip Dependencies

Pure `transformers` models

ESM2 and SaProt can be defined with the transformers library. To install these models:

```
pip install -r requirements-transformers.txt
```

This enables:

- ESM2 embeddings and likelihood scoring
- SaProt structure-aware embeddings and scoring

MSA Transformer

MSA transformer requires bespoke components from fair-esm:

```
pip install -r requirements-fair-esm.txt
```

This enables:

- MSA transformer embeddings and likelihood scoring

EVmutation

For evolutionary coupling analysis:

```
pip install -r requirements-evmutation.txt
```

This enables:

- EVMutation for protein mutation effect prediction

VESPA Integration

For conservation-based variant effect prediction:

```
pip install -r requirements-vespa.txt
```

Independent Environment

EVE Integration

EVE requires special handling due to its complex environment requirements:

1. Clone the EVE repository outside your AIDE directory:

```
git clone https://github.com/OATML/EVE.git
```

2. Set required environment variables:

```
export EVE_REPO=/path/to/eve/repo
```

3. Create a dedicated conda environment for EVE following their installation instructions.
4. Set the EVE environment name:

```
export EVE_CONDA_ENV=eve_env
```

2.1.3 Verifying Your Installation

You can check which components are available in your installation:

```
from aide_predict.utils.checks import get_supported_tools
print(get_supported_tools())
```

2.1.4 Common Installation Issues

CUDA Compatibility

If you're using GPU-accelerated components (ESMFold, transformers), ensure your CUDA drivers are compatible:

- Check CUDA version: `nvidia-smi`
- Match PyTorch installation with CUDA version
- For Apple Silicon users: Some components may require alternative installations

2.2 API examples

The following should look and feel like canonical sklearn tasks/code. See the `demo` folder for more details and executable examples. Also see the colab notebook to play with some if its capabilities in the cloud. Finally, checkout the notebooks in `showcase` where we conduct two full protein predictions optimization and scoring tasks on real data that are greater than small example sets.

2.2.1 1. Checking which protein models are available given the data you have

```
from aide_predict.utils.checks import check_model_compatability
seqs = ProteinSequences.from_csv('csv_file.csv', seq_col='sequence')
wt = seqs[0]

check_model_compatibility(
    training_msa=None,
    training_sequences=seqs,
    wt=wt,
)
>>>{'compatible': ['ESM2Embedding',
  'ESM2LikelihoodWrapper',
  'KmerEmbedding',
  'OneHotProteinEmbedding'],
```

(continues on next page)

(continued from previous page)

```
'incompatible': ['EVMutationWrapper',
  'HMMWrapper',
  'MSATransformerEmbedding',
  'MSATransformerLikelihoodWrapper',
  'OneHotAlignedEmbedding',
  'SaProtEmbedding',
  'SaProtLikelihoodWrapper',
  'VESPAWrapper']}
```

2.2.2 2. In silico mutagenesis using MSATransformer

```
# data preparation
wt = ProteinSequence.from_fasta("data/msa.fasta") # assigns the msa attribute of the␣
↪sequence
wt.has_msa
>>> True
wt.msa_same_width
>>> True
library = wt.saturation_mutagenesis()
mutations = library.ids
print(mutations[0])
>>> 'L1A'

# model fitting
model = MSATransformerLikelihoodWrapper(
   wt=wt,
   marginal_method="masked_marginal"
)
model.fit()

# make predictions for each mutated sequence
predictions = model.predict(library)

results = pd.DataFrame({'mutation': mutations, 'seqeunce': library,'prediction':␣
↪predictions})
```

2.2.3 3. Compare a couple of zero shot predictors against experimental data

```
# data preparation
X, y = ProteinSequences.from_csv("data/experimental_data.csv", seq_col='sequence', id_
↪col='id', label_cols='experimental_value')
wt = X['my_id_for_WT']
msa = ProteinSequences.from_fasta("data/msa.fasta")
wt.msa = msa

# model defenitions
evmut = EVMutation(wt=wt, metadata_folder='./tmp/evm')
evmut.fit()
esm2 = ESM2LikelihoodWrapper(wt=wt, model_checkpoint='esm2_t33_650M_UR50S')
```

(continues on next page)

(continued from previous page)

```
esm2.fit()
models = {'evmut': evmut, 'esm2': esm2}

# model fitting and scoring
for name, model in models.items():
    score = model.score(X, y)
    print(f"{name} score: {score}")
```

2.2.4 4. Train a supervised model to predict activity on an experimental combinatorial library, test on sequences with greater mutational depth than training

```
# data preparation
sequences, y = ProteinSequences.from_csv("data/experimental_data.csv", seq_col='sequence
↪', id_col='id', label_cols='experimental_value')
sequences.aligned
>>> True
sequences.fixed_length
>>> True

wt = sequences['my_id_for_WT']
mutational_depth = np.array([len(x.mutated_positions(wt)) for x in sequences])
test_mask = mutational_depth > 5
train_X = sequences[~test_mask]
train_y = y[~test_mask]
test_X = sequences[test_mask]
test_y = y[test_mask]

# embeddings protein sequences
# use mean pool embeddings of esm2
embedder = ESM2Embedding(pool='mean')
train_X = embedder.fit_transform(train_X)
test_X = embedder.transform(test_X)

# model fitting
model = RandomForestRegressor()
model.fit(train_X, train_y)

# model scoring
train_score = model.score(train_X, train_y)
test_score = model.score(test_X, test_y)
print(f"Train score: {train_score}, Test score: {test_score}")
```

2.2.5 5. Train a supervised predictor on a set of homologs, focusing only on positions of known importance, wrap the entire process into an sklearn pipeline including some standard sklearn transormers, and make predictions for a new set of homologs

```
# data preparation
sequences, y_train = ProteinSequences.from_csv("data/training_data.csv", seq_col=
↪'sequence', id_col='id', label_cols='experimental_value')

wt = sequences['my_id_for_WT']
wt_important_positions = np.array([20, 21, 22, 33, 45]) # zero indexed, known from␣
↪analysis elsewhere
sequences.aligned
>>> False
sequences.fixed_length
>>> False

# align the training sequences and get the important positions
msa = sequences.align_all()
msa.fixed_length
>>> False
msa.aligned
>>> True

wt_alignment_mapping = msa.get_alignment_mapping()['my_id_for_WT']
aligned_important_positions = wt_alignment_mapping[wt_important_positions]

# model defenitions
embedder = OneHotAlignedEmbedding(important_positions=aligned_important_positions).
↪fit(msa)
scaler = StandardScaler()
feature_selector = VarianceThreshold(threshold=0.2)
predictor = RandomForestRegressor()
pipeline = Pipeline([
    ('embedder', embedder),
    ('scaler', scaler),
    ('feature_selector', feature_selector),
    ('predictor', predictor)
])

# model fitting
pipeline.fit(sequences, y_train)

# score new unaligned homologs
new_homologs = ProteinSequences.from_fasta("data/new_homologs.fasta")
y_pred = pipeline.predict(new_homologs)
```

2.2.6 6. Create new embedder or predictor within the aide framework

Here we create a K-mer counting embedding, except use Foldseek structure tokens instead of amino acids. We set the `_available` attribute to allow aide to dynamically check if the model is available to call.

```
import numpy as np
from typing import List, Union, Optional
from collections import defaultdict

from aide_predict.bespoke_models.base import ProteinModelWrapper,␣
↪CanHandleAlignedSequencesMixin, RequiresStructureMixin
from aide_predict.utils.data_structures import ProteinSequences, ProteinSequence
from aide_predict.utils.common import MessageBool
from aide_predict.bespoke_models.predictors.saprot import get_structure_tokens
#check if 'foldseek' is available to path for a terminal
if shutil.which('foldseek') is None:
    AVAILABLE = MessageBool(False, 'Foldseek is not available, please install and set␣
↪environment variables.')
else:
    AVAILABLE = MessageBool(True, 'Foldseek is available')

class FoldseekKmerEmbedding(RequiresStructureMixin, CanHandleAlignedSequencesMixin,␣
↪ProteinModelWrapper):
    _available=AVAILABLE
    def __init__(self, metadata_folder: str = None,
                 k: int = 3,
                 wt: ProteinSequence = None):
        super().__init__(metadata_folder=metadata_folder, wt=None)
        self.k = k
        self._kmer_to_index = {}

    def _fit(self, X: ProteinSequences, y: Optional[np.ndarray] = None) -> 'KmerEmbedding
↪':
        unique_kmers = set()
        for seq in X:
            if seq.structure is None:
                raise ValueError("KmerEmbedding requires a structure to be present in␣
↪each sequence.")

            struct_str = get_structure_tokens(seq.structure)
            unique_kmers.update(struct_str[i:i+self.k] for i in range(len(struct_str) -␣
↪self.k + 1))

        self._kmer_to_index = {kmer: i for i, kmer in enumerate(sorted(unique_kmers))}
        self.n_features_ = len(self._kmer_to_index)
        self.fitted_ = True
        return self

    def _transform(self, X: ProteinSequences) -> np.ndarray:
        """
        Transform the protein sequences into K-mer embeddings.

        Args:
```

(continues on next page)

(continued from previous page)

```
            X (ProteinSequences): The input protein sequences.

        Returns:
            np.ndarray: The K-mer embeddings for the sequences.
        """
        embeddings = np.zeros((len(X), self.n_features_), dtype=np.float32)

        for i, seq in enumerate(X):
            if seq.structure is None:
                raise ValueError("KmerEmbedding requires a structure to be present in␣
↪each sequence.")

            struct_str = get_structure_tokens(seq.structure)
            for j in range(len(struct_str) - self.k + 1):
                kmer = struct_str[j:j+self.k]
                if kmer in self._kmer_to_index:
                    embeddings[i, self._kmer_to_index[kmer]] += 1

        return embeddings
```

Here we define an arbitrary predictor that calls a third party script and environment and communicates with aide via IO. We can check for model availability by checking for enviornment variables associated with the third party environment and location if necessary.

```
import numpy as np
from typing import List, Union, Optional
from collections import defaultdict
import os
import subprocess
import tempfile
from aide_predict.bespoke_models.base import ProteinModelWrapper, RequiresStructureMixin,
↪ RequiresFixedLengthSequencesMixin
from aide_predict.utils.data_structures import ProteinSequences, ProteinSequence
from aide_predict.utils.common import MessageBool
from aide_predict.bespoke_models.predictors.saprot import get_structure_tokens

try:
    ENV_NAME = os.environ['BESPOKE_ENV_NAME']
    MODEL_BASE_DIR = os.environ['BESPOKE_MODEL_BASE_DIR']
    AVAILABLE = MessageBool(True, 'Model is available')
except KeyError:
    AVAILABLE = MessageBool(False, 'Model is not available, please install and set␣
↪environment variables.')

class MyBespokeModel(ProteinModelWrapper, RequiresStructureMixin,␣
↪RequiresFixedLengthSequencesMixin):
    _available = AVAILABLE

    def __init__(self, *args, **kwargs):
        super().__init__(*args, **kwargs)
        # set params....
```

(continues on next page)

(continued from previous page)

```
    def _fit(self, X: ProteinSequences, y: Optional[np.ndarray] = None):
        # prepare data
        # call subprocess
        input_fasta = tempfile.NamedTemporaryFile(delete=False)
        X.to_fasta(input_fasta.name)
        # temdir for structures
        with tempfile.TemporaryDirectory() as tempdir:
            # pass structure files to the script
            structure_files = open(os.path.join(tempdir, 'structure_files.txt'), 'w')
            for seq in X:
                struct_path = seq.structure.pdb_file
                structure_files.write(f'{seq.id}\t{struct_path}\n')
            structure_files.close()

            # call subprocess in the environment and base dir
            subprocess.run(['python', 'path/to/train_script.py', '-s', structure_files,␣
↪input_fasta.name, '-o', os.path.join(self.metadata_folder, 'model.pkl'), env=ENV_NAME,␣
↪cwd=MODEL_BASE_DIR])
        return self

    def _transform(self, X: ProteinSequences) -> np.ndarray:
        # something similar to fit, call a predict script etc...

        return outputs.
```

2.3 Data Structures

AIDE provides several key data structures for working with protein sequences and structures. Models will receive these data structures as input in analog to how sklearn receives numpy arrays.

2.3.1 ProteinSequence

`ProteinSequence` is the basic unit for representing a protein sequence. It behaves like a string but provides additional functionality specific to protein sequences.

```
from aide_predict import ProteinSequence

# Create a basic sequence
seq = ProteinSequence("MKLLVLGLPGAGKGT")

# or get from a pdb (see ProteinStructure below)
seq = ProteinSequence.from_pdb("path/to/structure.pdb")

# Add identifier and optional structure
seq = ProteinSequence(
    "MKLLVLGLPGAGKGT",
    id="P1234",
    structure="path/to/structure.pdb"
)
```

(continues on next page)

(continued from previous page)

```
# Create a sequence with an associated MSA
seq = ProteinSequence(
    "MKLLVLGLPGAGKGT",
    id="P1234",
    msa=msa_sequences  # ProteinSequences object containing the MSA
)

# From a fasta file, assumed to be aligned. Uses first sequence in file, set's the MSA␣
↪attribute
seq = ProteinSequence.from_fasta("path/to/aligned.fasta")
seq.has_msa
>>> True
```

Key attributes and methods:

- `id`: Optional identifier for the sequence
- `structure`: Optional associated structure (as ProteinStructure object)
- `msa`: Optional associated multiple sequence alignment (as ProteinSequences object)
- `has_gaps`: Boolean indicating if sequence contains gaps ('-' or '.')
- `base_length`: Length excluding gaps
- `has_non_canonical`: Boolean indicating presence of non-standard amino acids
- `as_array`: Sequence as numpy array
- `has_msa`: Boolean indicating if the sequence has an associated MSA
- `msa_same_width`: Boolean indicating if the MSA has the same width as the sequence
- `is_in_msa`: Boolean indicating if the sequence is present in its MSA

Common operations:

```
# Sequence manipulation
ungapped = seq.with_no_gaps()  # Remove gaps
upper_seq = seq.upper()  # Convert to uppercase

# Mutation operations
mutated = seq.mutate("A123G")  # Single mutation
mutated = seq.mutate(["A123G", "L45R"])  # Multiple mutations

# Compare sequences
positions = seq.mutated_positions(other_seq)  # Get positions that differ
mutations = seq.get_mutations(other_seq)  # Get mutation strings (e.g., "A123G")

# Create all possible mutations
library = seq.saturation_mutagenesis()  # Generate all single mutants
library = seq.saturation_mutagenesis(positions=[1,2,3])  # Specific positions

# align to another sequence
seq2 = seq2.align(seq)

# align to an existing alignment
```

(continues on next page)

(continued from previous page)

```
seq2 = seq2.align(msa)  # Align to existing MSA
```

2.3.2 ProteinSequences

`ProteinSequences` manages collections of protein sequences, similar to a list but with additional functionality for protein-specific operations.

```
from aide_predict import ProteinSequences

# Create from various sources
sequences = ProteinSequences([seq1, seq2, seq3])  # From ProteinSequence objects
sequences = ProteinSequences.from_fasta("sequences.fasta")  # From FASTA file
sequences = ProteinSequences.from_a3m("alignment.a3m")  # From A3M file
sequences = ProteinSequences.from_list(["MKLL...", "MKLT..."])  # From strings
sequences = ProteinSequences.from_dict({"seq1": "MKLL...", "seq2": "MKLT..."})
sequences = ProteinSequences.from_dict(my_dataframe["sequence"].to_dict())
sequences = ProteinSequences.from_df(my_dataframe) # assumes first column is sequences
sequences = ProteinSequences.from_df(my_dataframe, sequence_col="seq_col", id_col="id_col
↪") # specify columns
sequences, labels = ProteinSequences.from_df(my_dataframe, label_cols='label') # get a␣
↪array of labels
```

Key attributes:

- `aligned`: Boolean indicating if all sequences have same length (including gaps)
- `fixed_length`: Boolean indicating if all sequences have same base length (excluding gaps)
- `width`: Length of sequences if aligned, None otherwise
- `has_gaps`: Boolean indicating if any sequence contains gaps
- `mutated_positions`: List of positions with variation (for aligned sequences)
- `weights`: Optional weights for each sequence (used in some models)
- `ids`: List of sequence IDs

Common operations:

```
# Sequence alignment
aligned = sequences.align_all()  # Align all sequences
aligned = sequences.align_to(reference_msa)  # Align to existing MSA

# Access and manipulation
sequences[0]  # Access by index
sequences["seq1"]  # Access by ID
sequences.with_no_gaps()  # Remove gaps from all sequences
sequences.upper()  # Convert all to uppercase

# MSA operations
msa = sequences.msa_process(
    focus_seq_id="wild_type",
    theta=0.8  # Sequence reweighting parameter
```

(continues on next page)

(continued from previous page)

```
)

# Sampling operations
sampled = sequences.sample(n=100, replace=False, keep_first=True)  # Sample sequences,␣
↪keeping the first one

# Save/export
sequences.to_fasta("output.fasta")
sequences_dict = sequences.to_dict()

# Batching for large datasets
for batch in sequences.iter_batches(batch_size=32):
    # Process batch
    pass

# Get mapping between aligned and original positions
if sequences.aligned:
    alignment_mapping = sequences.get_alignment_mapping()
    # Returns dict mapping sequence IDs to lists of positions

# Convert to on-file representation for large MSAs
on_file = sequences.to_on_file("output.fasta")  # Save to file and return a file-based␣
↪object
```

2.3.3 ProteinStructure

`ProteinStructure` represents the 3D structure of a protein, integrating with common structure file formats and analysis tools.

```
from aide_predict import ProteinStructure

# Create from PDB file
structure = ProteinStructure(
    pdb_file="protein.pdb",
    chain="A",  # Optional chain identifier
    plddt_file="confidence.json"  # Optional AlphaFold2 confidence scores
)

# Create from AlphaFold2 output folder
structure = ProteinStructure.from_af2_folder(
    folder_path="af2_results",
    chain="A"
)
```

Key methods:

```
# Access structure information
sequence = structure.get_sequence()  # Get amino acid sequence
plddt = structure.get_plddt()  # Get pLDDT confidence scores
dssp = structure.get_dssp()  # Get secondary structure assignments
```

(continues on next page)

(continued from previous page)

```
# Validation
structure.validate_sequence("MKLLVLGLPGAGKGT")  # Check if sequence matches structure

# Access underlying structure objects
structure_obj = structure.get_structure()  # Get BioPython Structure object
chain_obj = structure.get_chain()  # Get specific chain
positions = structure.get_residue_positions()  # Get residue numbers
```

2.3.4 StructureMapper

`StructureMapper` helps manage multiple structures and map them to sequences, particularly useful when working with structure-aware models.

```
from aide_predict import StructureMapper

# Initialize with folder containing structures
mapper = StructureMapper("path/to/structures")

# Structure can be PDB files or AlphaFold2 prediction folders
# Example folder structure:
# structures/
#   ├── protein1.pdb
#   ├── protein2.pdb
#   └── protein3/  # AlphaFold2 output folder
#       ├── ranked_0.pdb
#       └── ranking_confidence.json

# Assign structures to ProteinSequences already loaded
sequences = mapper.assign_structures(sequences)

# Get available structures
available_ids = mapper.get_available_structures()

# Get ProteinSequences with structures
sequences = mapper.get_protein_sequences()
```

The StructureMapper is particularly useful when working with structure-aware models like SaProt, which can use structure information to improve predictions:

```
# Example workflow with structure-aware model
mapper = StructureMapper("structures/")
sequences = ProteinSequences.from_fasta("sequences.fasta")
sequences = mapper.assign_structures(sequences)

model = SaProtLikelihoodWrapper(wt=sequences["wild_type"])
predictions = model.predict(sequences)  # Will use structures where available, falling␣
↪back to the WT structure
```

2.3.5 ProteinSequencesOnFile

`ProteinSequencesOnFile` provides a memory-efficient way to work with large alignments by only loading sequences when needed.

```
from aide_predict import ProteinSequencesOnFile

# Create from FASTA file
on_file_sequences = ProteinSequencesOnFile("large_alignment.fasta")

# Access properties efficiently without loading everything
print(on_file_sequences.aligned)  # Check if aligned
print(on_file_sequences.width)    # Get width if aligned
print(len(on_file_sequences))     # Get count of sequences

# Load a specific sequence
seq = on_file_sequences[0]  # Get by index
seq = on_file_sequences["seq1"]  # Get by ID

# Iterate through sequences without loading all at once
for seq in on_file_sequences:
    process_sequence(seq)

# Load into memory if needed
in_memory = on_file_sequences.to_memory()
```

2.3.6 ProteinTrajectory

NOT YET IMPLEMENTED

2.4 Model Compatibility

2.4.1 Understanding Model Requirements

AIDE models have different requirements and capabilities that determine whether they can be used with your data. Key considerations include:

- Whether the model requires training data (supervised vs zero-shot)
- Whether sequences must be aligned or of fixed length
- Whether the model requires a Multiple Sequence Alignment (MSA)
- Whether the model requires or can use structural information
- Whether the model needs a wild-type sequence for comparison
- Whether the model can handle variable-length sequences

2.4.2 Checking Model Compatibility

AIDE provides a utility function to check which models are compatible with your data:

```
from aide_predict.utils.checks import check_model_compatibility
from aide_predict import ProteinSequences, ProteinSequence

# Example setup
sequences = ProteinSequences.from_fasta("my_sequences.fasta")
msa = ProteinSequences.from_fasta("family_msa.fasta")
wt = ProteinSequence("MKLLVLGLPGAGKGT", id="wild_type")
wt.msa = msa

# Check compatibility
compatibility = check_model_compatibility(
    training_sequences=sequences,  # Optional: sequences for supervised learning
    testing_sequences=None,        # Optional: test sequences if different from training
    wt=wt                          # Optional: wild-type sequence, may have structure,␣
↪MSA
)

print("Compatible models:", compatibility["compatible"])
print("Incompatible models:", compatibility["incompatible"])
```

The compatibility checker performs several validation steps:

- Verifies if structure information is available (either in sequences or wild-type)
- Checks if MSAs are available and properly aligned
- Validates that sequence lengths match requirements
- Ensures wild-type sequences are available when needed
- Verifies that per-sequence MSAs match sequence lengths when required

You can also check which tools are available in your current installation:

```
from aide_predict.utils.checks import get_supported_tools
print(get_supported_tools())
```

2.4.3 Model Categories

AIDE models fall into several categories:

1. Zero-Shot Predictors

These models don't require training data but may have other requirements:

```
# ESM2 - Requires only sequences
from aide_predict import ESM2LikelihoodWrapper
model = ESM2LikelihoodWrapper(wt=wt)
model.fit()  # No training needed
scores = model.predict(sequences)
```

(continues on next page)

(continued from previous page)

```
# MSATransformer - Requires MSA for the WT
from aide_predict import MSATransformerLikelihoodWrapper
model = MSATransformerLikelihoodWrapper(wt=wt)
model.fit()
scores = model.predict(sequences)

# SaProt - Can use structural information
from aide_predict import SaProtLikelihoodWrapper
model = SaProtLikelihoodWrapper(wt=wt) # wt must have structure
model.fit()
scores = model.predict(sequences)  # Will use structure if available
```

Other zero-shot predictors include:

- **HMM**: Creates Hidden Markov Models from MSAs
- **EVMutation**: Uses evolutionary couplings from MSAs
- **VESPA**: Pre-trained model for variant effect prediction
- **EVE**: Evolutionary model using latent space representations
- **SSEmb**: Structure and sequence-based variant effect predictor

2. Embedding Models

These models convert sequences into numerical features for downstream ML:

```
# Simple one-hot encoding
from aide_predict import OneHotProteinEmbedding
embedder = OneHotProteinEmbedding()
X = embedder.fit_transform(sequences)

# Advanced language model embeddings
from aide_predict import ESM2Embedding
embedder = ESM2Embedding(pool=True)  # pool=True for sequence-level embeddings
X = embedder.fit_transform(sequences)

# K-mer based embeddings
from aide_predict import KmerEmbedding
embedder = KmerEmbedding(k=3)
X = embedder.fit_transform(sequences)
```

Other embedding models include:

- **MSATransformerEmbedding**: Produces embeddings using MSAs
- **SaProtEmbedding**: Structure-aware protein language model embeddings
- **OneHotAlignedEmbedding**: One-hot encodings for aligned sequences

2.4.4 Importance of Data Structure

The compatibility of models depends heavily on the structure of your data:

Data Characteristic	Compatible Models	Incompatible Models
Fixed-length sequences	All models	-
Variable-length sequences	Models without `RequiresFixedLengthMixin`	Models with `RequiresFixedLengthMixin`
Has MSA	All models	-
No MSA	Models without MSA requirements	MSATransformer, EVMutation, EVE
Has structure	All models	-
No structure	Models without structure requirements	SaProt, SSEmb
Has wild-type	All models	-
No wild-type	Models without WT requirements	Models with `RequiresWTToFunctionMixin`

Using the appropriate data structure for your specific modeling task ensures that AIDE can provide the most accurate predictions.

2.5 ProteinModelWrapper

2.5.1 Overview

`ProteinModelWrapper` is the base class for all protein prediction models in AIDE. It inherits from scikit-learn's `BaseEstimator` and `TransformerMixin`, providing a familiar API while adding protein-specific validation and functionality. The framework uses a powerful mixin architecture to define model requirements and capabilities.

2.5.2 Core Behavior

The wrapper handles all protein-specific validation through its public methods:

```python
# Public methods do all validation
def fit(self, X, y=None):
    # Validates sequences
    # Checks requirements based on mixins
    # Runs pre-fit hooks from mixins
    # Then calls _fit()
    # Runs post-fit hooks from mixins

def transform(self, X):
    # Validates sequences
    # Checks requirements based on mixins
    # Runs pre-transform hooks from mixins
    # Then calls _transform()
    # Runs post-transform hooks from mixins
```

You never need to call the private methods directly - they implement just the core model logic while the public methods handle validation, hooks, and other processing.

2.5.3 Key Attributes

Data Paths and References

- `metadata_folder`: Directory for model files (weights, checkpoints, etc.), randomly generated if not given.
  - This serves to give each model a dedicated place to store necessary files, for example if a fasta file needs to be passed to an external program. Some models do not use it. If the model is capable of Caching (see the caching section), it is stored here. Currently this location may break when saving and loading models across machines.
- `wt`: Optional wild-type sequence for comparative predictions

Automated Properties

These are set based on which mixins you use:

```
# Model requirements
model.expects_no_fit                   # Whether the model expects no fit
model.requires_msa_for_fit             # Whether the model requires an MSA as input for␣
↪fitting
model.requires_wt_msa                  # Whether the model requires a wild type MSA
model.requires_msa_per_sequence        # Whether the model requires an MSA for each sequence
model.requires_fixed_length            # Whether the model requires a fixed length input
model.requires_wt_to_function          # Whether the model requires the wild type sequence
model.requires_wt_during_inference # Whether wild is used during inference, see note␣
↪below
model.requires_structure               # Whether the model requires structure information

# Model capabilities
model.per_position_capable             # Whether the model can output per position scores
model.can_regress                      # Whether the model outputs are estimates of activity
model.can_handle_aligned_sequences # Whether the model can handle unaligned sequences
model.accepts_lower_case               # Whether the model can accept lowercase sequences
model.should_refit_on_sequences        # Whether model should refit on new sequences
```

2.5.4 Mixin Hooks System

AIDE uses a system of hooks to allow mixins to modify the behavior of fitting and transformation. Hooks are registered during subclass creation and executed in order:

- `_mixin_init_handlers`: Runs during initialization to extract mixin parameters
- `_pre_fit_hooks`: Runs before fitting to prepare data
- `_post_fit_hooks`: Runs after fitting to process results
- `_pre_transform_hooks`: Runs before transformation to prepare data
- `_post_transform_hooks`: Runs after transformation to process results

For example, the `CacheMixin` uses `_pre_transform_hook` to check if results are already cached and `_post_transform_hook` to store new results in the cache.

2.5.5 Wrapping a New Model

To wrap a new model:

1. Inherit from `ProteinModelWrapper` and any needed mixins
2. Implement only the core logic in private methods

Example:

```
class MyModel(CanRegressMixin, ProteinModelWrapper):
    def __init__(self, metadata_folder=None, my_param=1.0):
        super().__init__(metadata_folder=metadata_folder)
        self.my_param = my_param

    def _fit(self, X, y=None):
        # Just core training logic
        # X is guaranteed to be valid
        # set a fitted attribute so sklearn can check if it's been fit
        self.fitted_ = True
        return self

    def _transform(self, X):
        # Just core transformation
        # X is guaranteed to be valid
        return features
```

2.5.6 Availability Checking

Models can specify their availability based on installed dependencies:

```
try:
    import some_required_package
    AVAILABLE = MessageBool(True, "Model is available")
except ImportError:
    AVAILABLE = MessageBool(False, "Requires some_required_package")

class MyModel(ProteinModelWrapper):
    _available = AVAILABLE
```

2.5.7 Mixins

Mixins define model requirements and capabilities. When combined with `ProteinModelWrapper`, they automatically enable appropriate validation and behavior. Mixins are grouped by their general purpose:

Training Behavior

ExpectsNoFitMixin

For models that don't require training:

- Sets `expects_no_fit = True`
- Used by pretrained models like ESM2, SaProt
- Allows `fit()` to be called with no data

ShouldRefitOnSequencesMixin

For models that need retraining on new sequences:

- Sets `should_refit_on_sequences = True`
- By default sklearn will refit when fit is called, this is undesirable for some models (e.g., those that fit using an MSA)
- That default behavior was disabled for `ProteinModelWrapper` but can be re-enabled by using this mixin

Input Requirements

RequiresMSAForFitMixin

For models trained on multiple sequence alignments:

- Sets `requires_msa_for_fit = True`
- Base class ensures input is aligned during fit
- Model receives guaranteed aligned sequences in `_fit`
- Used by evolutionary models like EVMutation, HMM

RequiresWTMSAMixin

For models that need the wild-type MSA during training:

- Sets `requires_wt_msa = True`
- Ensures wild-type sequence has an associated MSA
- Used by models like EVE that need WT context

RequiresMSAPerSequenceMixin

For models requiring MSA information for each sequence:

- Sets `requires_msa_per_sequence = True`
- Ensures each sequence has its own MSA or falls back to WT MSA
- Used by MSA Transformer for embedding generation

RequiresFixedLengthMixin

For models requiring uniform sequence length:

- Sets `requires_fixed_length = True`
- Base class validates all sequences are same length
- Common in neural networks and position-specific models
- Validates wild-type length matches if present

RequiresStructureMixin

For models using structural information:

- Sets `requires_structure = True`
- Ensures structures available or falls back to wild-type structure
- Used by structure-aware models like SaProt

RequiresWTToFunctionMixin

For models that need a reference sequence:

- Sets `requires_wt_to_function = True`
- Ensures wild-type sequence provided at initialization
- Used by models computing mutation effects

RequiresWTDuringInferenceMixin

These models are expected to handle their own use of any widl type sequence:

- Sets `requires_wt_during_inference = True`
- If wt is present during inference, AIDE does no further processing, where by default AIDE will call the model on any WT present and normalize the output to that sequence

Output Capabilities

CanRegressMixin

For models producing numeric predictions:

- Sets `can_regress = True`
- Enables `predict()` method
- Adds Spearman correlation scoring
- Common in variant effect predictors

PositionSpecificMixin

For models with per-position outputs:

- Sets `per_position_capable = True`
- Adds position selection with `positions` parameter
- Controls output format with `pool` and `flatten` parameters
- Handles dimension validation and reshaping
- Common in language models and conservation analysis

Data Processing

CanHandleAlignedSequencesMixin

For models working with gapped sequences:

- Sets `can_handle_aligned_sequences = True`
- Prevents automatic gap removal by base class
- Essential for MSA-based models
- Ensures gap characters preserved during processing

AcceptsLowerCaseMixin

For case-sensitive models:

- Sets `accepts_lower_case = True`
- Disables automatic uppercase conversion
- Useful when case represents conservation or focus columns

Computational Behavior

CacheMixin

For caching model outputs:

- Adds disk-based caching system using SQLite and HDF5
- Thread-safe for parallel processing
- Automatic cache invalidation on parameter changes
- Particularly useful for computationally expensive models

Using Multiple Mixins

Mixins can be combined to define complex model requirements. Always put `ProteinModelWrapper` last in the inheritance chain:

```
class ComplexModel(
    RequiresMSAForFitMixin,       # Needs MSA for training
    RequiresWTToFunctionMixin,    # Needs wild-type reference
    CanRegressMixin,              # Makes predictions
    PositionSpecificMixin,        # Per-position outputs
    CacheMixin,                   # Caches results
    ProteinModelWrapper           # Always last
):
    def _fit(self, X: ProteinSequences, y=None):
        # X is guaranteed to be aligned
        # Implementation focuses on core logic
        pass

    def _transform(self, X: ProteinSequences):
        # All requirements validated by base class
        # Implementation focuses on core logic
        pass
```

2.5.8 Complete List of Mixins

Mixin	Purpose	Sets
ExpectsNoFitMixin	For models without fitting	expects_no_fit = True
RequiresMSAForFitMixin	For MSA-based training	requires_msa_for_fit = True
RequiresWTMSAMixin	For models needing WT MSA	requires_wt_msa = True
RequiresMSAPerSequenceMixin	For per-sequence MSA	requires_msa_per_sequence = True
RequiresFixedLengthMixin	For fixed-length inputs	requires_fixed_length = True
RequiresStructureMixin	For structure-aware models	requires_structure = True
RequiresWTToFunctionMixin	For models needing WT	requires_wt_to_function = True
RequiresWTDuringInferenceMixi	For WT during inference	requires_wt_during_inference = True
CanRegressMixin	For prediction models	can_regress = True
PositionSpecificMixin	For position outputs	per_position_capable = True
CanHandleAlignedSequencesMixi	For gapped sequences	can_handle_aligned_sequences = True
AcceptsLowerCaseMixin	For case sensitivity	accepts_lower_case = True
CacheMixin	For result caching	Adds caching hooks
ShouldRefitOnSequencesMixin	For retraining	should_refit_on_sequences = True

2.6 Zero-Shot Prediction

2.6.1 Overview

Zero-shot predictors in AIDE can assess protein variants without requiring training data. These models leverage different types of information:

- Pretrained language models that capture protein sequence patterns
- Multiple sequence alignments that capture evolutionary information
- Structural information for 3D context and conservation signals
- Combinations of these approaches for more robust predictions
- Note that the examples below are all single point mutations, but many of the models can also be used for multiple mutations.

2.6.2 Transformer-Based Models

ESM2

ESM2 uses masked language modeling to predict mutation effects based on the likelihood of amino acids in context:

```
from aide_predict import ESM2LikelihoodWrapper, ProteinSequence

# Setup wild type sequence
wt = ProteinSequence(
    "MKLLVLGLPGAGKGT",
    id="wild_type"
```

(continues on next page)

(continued from previous page)

```
)

# Choose marginal method for computing likelihoods
model = ESM2LikelihoodWrapper(
    wt=wt,
    marginal_method="masked_marginal",  # or "wildtype_marginal" or "mutant_marginal"
    pool=True  # True to get single score per sequence
)

# No training needed
model.fit()

# Score mutations
mutants = wt.saturation_mutagenesis()
scores = model.predict(mutants)
```

The marginal method determines how likelihoods are computed:

- `masked_marginal`: Masks each position to compute direct probability
- `wildtype_marginal`: Uses wild type context only
- `mutant_marginal`: Uses mutant sequence context

MSA Transformer

MSA Transformer extends ESM's approach by incorporating evolutionary information from multiple sequence alignments:

```
from aide_predict import MSATransformerLikelihoodWrapper, ProteinSequence

# Setup wild type sequence with MSA
wt = ProteinSequence.from_a3m("protein_family.a3m")

# Create model with MSA context
model = MSATransformerLikelihoodWrapper(
    wt=wt,
    marginal_method="masked_marginal",
    n_msa_seqs=360  # Number of MSA sequences to use
)

# Fit to MSA
model.fit()

# Score mutations
mutants = wt.saturation_mutagenesis()
scores = model.predict(mutants)
```

MSA Transformer combines the power of language models with evolutionary information, often improving predictions for proteins with rich evolutionary profiles.

VESPA

VESPA uses a pretrained model head on top of transformer embeddings specifically trained to predict variant effects:

```
from aide_predict import VESPAWrapper, ProteinSequence

# Setup wild type sequence
wt = ProteinSequence(
    "MKLLVLGLPGAGKGT",
    id="wild_type"
)

# Create VESPA model (light version by default)
model = VESPAWrapper(
    wt=wt,
    light=True  # Use lighter VESPAl model instead of full VESPA
)

# No training needed
model.fit()

# Score single mutations (VESPA is only for single mutations)
mutants = wt.saturation_mutagenesis()
scores = model.predict(mutants)
```

VESPA was trained on human disease variants and is particularly useful for predicting pathogenicity of human protein variants.

2.6.3 Structure-Aware Models

SaProt

SaProt incorporates protein structure information with sequence to improve predictions:

```
from aide_predict import SaProtLikelihoodWrapper, ProteinStructure

# Load sequences and map structures
wt = ProteinStructure.from_pdb("structures/structure.pdb")

# Create model
model = SaProtLikelihoodWrapper(
    wt=wt,
    marginal_method="masked_marginal"
)

# No training needed
model.fit()

# Score mutations with structure info
mutatnts = wt.saturation_mutagenesis()
scores = model.predict(mutants)
```

SaProt is particularly valuable for proteins where structural context plays a significant role in function or stability.

SSEmb

SSEmb combines structure and sequence information through a joint embedding approach:

```
from aide_predict import SSEmbWrapper, ProteinSequence, StructureMapper

# Setup environment variables first
# os.environ['SSEMB_CONDA_ENV'] = 'ssemb_env'
# os.environ['SSEMB_REPO'] = '/path/to/ssemb/repo'

# Setup wild type with structure and MSA
wt = ProteinSequence.from_a3m("protein_family.a3m")
wt.structure = "structures/structure.pdb"

# Create model
model = SSEmbWrapper(wt=wt)

# Fit using MSA
model.fit()

# Score mutations
mutants = wt.saturation_mutagenesis()
scores = model.predict(mutants)
```

SSEmb is especially effective for scoring mutations in proteins with known structures and rich evolutionary information.

2.6.4 Evolutionary Models

HMM

Hidden Markov Models capture position-specific amino acid preferences from MSAs:

```
from aide_predict import HMMWrapper, ProteinSequences

# Load MSA
msa = ProteinSequences.from_fasta("protein_family.a3m")

# Create and fit model
model = HMMWrapper(threshold=100)  # bit score threshold
model.fit(msa)

# Score new sequences
sequences = ProteinSequences.from_fasta("variants.fasta")
scores = model.predict(sequences)
```

HMMs are fast and interpretable but don't capture dependencies between positions.

EVMutation

EVMutation analyzes co-evolution patterns in MSAs to capture epistatic effects:

```
from aide_predict import EVMutationWrapper

# Load MSA and wild type
wt = ProteinSequence.from_a3m("protein_family.a3m")

# Create and fit model
model = EVMutationWrapper(
    wt=wt,
    theta=0.8,  # Sequence weighting parameter
    protocol="standard"  # or "complex" or "mean_field"
)

# Fit using MSA
model.fit()

# Score mutations
mutants = wt.saturation_mutagenesis()
scores = model.predict(mutants)
```

EVMutation captures pairwise dependencies between positions, making it effective for predicting epistatic effects where multiple mutations interact.

EVE

EVE constructs a posterior latent distribution over an MSA and scores how "in-distribution" a sequence is:

```
from aide_predict import EVEWrapper

# Load MSA and wild type
wt = ProteinSequence.from_a3m("protein_family.a3m")

# Create model with custom parameters
model = EVEWrapper(
    wt=wt,
    encoder_z_dim=50,  # Dimensionality of latent space
    training_steps=400000  # Number of training steps
)

# Fit using MSA
model.fit()

# Score mutations
mutants = wt.saturation_mutagenesis()
scores = model.predict(mutants)
```

2.6.5 Contributing

If you have a zero-shot method you would like to have added, please reach out:

evan.komp (at) nrel.gov

2.7 Supervised Learning

2.7.1 Overview

AIDE supports supervised machine learning by converting protein sequences into numerical features using embedding models. These features can then be used with any scikit-learn compatible model.

2.7.2 Basic Example

Here's a complete example using ESM2 embeddings and random forest regression with hyperparameter optimization:

```
from aide_predict import ESM2Embedding, ProteinSequences
from sklearn.ensemble import RandomForestRegressor
from sklearn.model_selection import train_test_split, RandomizedSearchCV
from sklearn.pipeline import Pipeline
from scipy.stats import randint, uniform
import numpy as np

# Load data
sequences = ProteinSequences.from_fasta("sequences.fasta")
y = np.load("activity_values.npy")

# Split data
X_train, X_test, y_train, y_test = train_test_split(
    sequences, y, test_size=0.2, random_state=42
)

# Create pipeline
pipeline = Pipeline([
    ('embedder', ESM2Embedding(pool='max', use_cache=True)),  # Create sequence-level␣
↪embeddings
    ('rf', RandomForestRegressor(random_state=42))
])

# Define parameter space
param_distributions = {
    'rf__n_estimators': randint(100, 500),
    'rf__max_depth': [None] + list(range(10, 50, 10)),
    'rf__min_samples_split': randint(2, 20),
    'rf__min_samples_leaf': randint(1, 10)
}

# Random search
search = RandomizedSearchCV(
    pipeline,
```

(continues on next page)

(continued from previous page)

```
    param_distributions=param_distributions,
    n_iter=20,  # Number of parameter settings sampled
    cv=5,       # 5-fold cross-validation
    n_jobs=-1,  # Use all available cores
    scoring='r2',
    verbose=1
)

# Fit model
search.fit(X_train, y_train)

# Print results
print("\nBest parameters:", search.best_params_)
print("Best CV score:", search.best_score_)
print("Test score:", search.score(X_test, y_test))

# Make predictions on new sequences
new_sequences = ProteinSequences.from_fasta("new_sequences.fasta")
predictions = search.predict(new_sequences)
```

2.7.3 Saving and loading models

Models can be dumped and loaded with joblib like any other scikit-learn model:

```
import joblib

# Save the best model
joblib.dump(search.best_estimator_, 'protein_model.joblib')

# Load the model later
loaded_model = joblib.load('protein_model.joblib')
```

Note that this may currently break the `metadata_folder` attribute of models, unless it is loaded on the same machine in the same location. In future, protocols to zip up this folder with the model during saving and loading will be provided.

2.8 Saturation Mutagenesis

2.8.1 Overview

We provide tools to quickly run in silico saturation mutagenesis.

Create a `ProteinSequences` object of all single point mutations.

```
from aide_predict import ProteinSequence, ESM2LikelihoodWrapper
import pandas as pd

# Define wild type sequence
wt = ProteinSequence(
    "MKLLVLGLPGAGKGT",
    id="wild_type"
```

(continues on next page)

(continued from previous page)

```
)

# Generate all single mutants
mutant_library = wt.saturation_mutagenesis()
print(f"Generated {len(mutant_library)} variants")
>>> Generated 285 variants  # (15 positions × 19 possible mutations)
```

Then pass these to a zero shot predictor of your choice:

```
# Score variants using a zero-shot predictor
model = ESM2LikelihoodWrapper(
    wt=wt,
    marginal_method="masked_marginal",
    pool=True  # Get one score per variant
)
model.fit()  # No training needed
scores = model.predict(mutant_library)

# Create results dataframe
results = pd.DataFrame({
    'mutation': mutant_library.ids,  # e.g., "M1A", "K2R", etc.
    'sequence': mutant_library,
    'prediction': scores
})

# Sort by predicted effect
results = results.sort_values('prediction', ascending=False)
print("Top 5 predicted beneficial mutations:")
print(results.head())
```

2.8.2 Visualizing Results

AIDE provides built-in visualization tools for mutation effects:

```
from aide_predict.utils.plotting import plot_mutation_heatmap

# Create heatmap of mutation effects
plot_mutation_heatmap(results['mutation'], results['prediction'])
```

The heatmap shows the predicted effect of each possible amino acid substitution at each position, making it easy to identify patterns and hotspots for engineering.

2.8.3 Notes

- The `mutation` IDs follow standard notation: "M1A" means the M at position 1 was mutated to A

2.9 Building ML Pipelines

AIDE models can be combined with standard scikit-learn components into pipelines. Here's an example that combines one-hot encoding and ESM2 ZS predictions with a random forest:

```
from aide_predict import OneHotProteinEmbedding, ESM2LikelihoodWrapper, ProteinSequence,␣
↪ProteinSequences
from sklearn.pipeline import Pipeline, FeatureUnion
from sklearn.preprocessing import StandardScaler, FunctionTransformer
from sklearn.ensemble import RandomForestRegressor

# Load data
sequences = ProteinSequences.from_fasta("sequences.fasta")
y = np.load("activity_values.npy")

# Create wild type reference
wt = sequences["wild_type"]

# Create feature union that combines raw OHE with scaled ESM2 scores
features = FeatureUnion([
    # One-hot encoding (keep as binary)
    ('ohe', OneHotProteinEmbedding(flatten=True)),

    # ESM2 features (apply scaling)
    ('esm2', Pipeline([
        ('predictor', ESM2LikelihoodWrapper(wt=wt, marginal_method="masked_marginal")),
        ('reshaper', FunctionTransformer(lambda x: x.reshape(-1, 1))),
        ('scaler', StandardScaler())
    ]))
])

# Create and train pipeline
pipeline = Pipeline([
    ('features', features),
    ('rf', RandomForestRegressor())
])

pipeline.fit(sequences, y)
predictions = pipeline.predict(sequences)
```

The pipeline can be saved and loaded like any scikit-learn model:

```
from joblib import dump, load
dump(pipeline, 'protein_model.joblib')
```

All standard scikit-learn tools like `GridSearchCV` or `cross_val_score` can be used with these pipelines.

2.10 Caching Model Outputs

2.10.1 Overview

Some AIDE models support caching their outputs to disk to avoid recomputing expensive transformations. This is made available with the `CacheMixin` class, which is inherited by models that support caching. You can check if a model supports caching by checking if it inherits from `CacheMixin`:

```
from aide_predict.bespoke_models.base import CacheMixin
assert isinstance(model, CacheMixin)  # True if model supports caching
```

2.10.2 Using Caches

Caching is enabled by default for models that support it. To explicitly control caching:

```
from aide_predict import ESM2Embedding

# Disable caching
model = ESM2Embedding(use_cache=False)

# Enable caching (default)
model = ESM2Embedding(use_cache=True)
```

2.10.3 How It Works

- Each protein sequence gets a unique hash based on its sequence, ID, and structure (if present)
- Outputs are stored in HDF5 format for efficient retrieval
- Cache also hashes the model parameters, so if model parameters change it will not use previous cache values
- Stores metadata in SQLite for quick cache checking
- Caches are stored in the model's metadata folder

2.10.4 Models Supporting Caching

You can check if a model supports caching by checking if it inherits from `CacheMixin`:

```
from aide_predict.bespoke_models.base import CacheMixin

isinstance(model, CacheMixin)  # True if model supports caching
```

NOTE: When wrapping a new model, it is recommended that `CacheMixin` be inherited first behind `ProteinModelWrapper`. This ensures that the final model outputs after any processing conducted by other mixins is what get cached, preventing any unnecessary recomputation.

2.10.5 Cache Location

Caches are stored in a `cache` subdirectory of the model's metadata folder:

```
# Specify cache location
model = ESM2Embedding(metadata_folder="my_model")
# Creates: my_model/cache/cache.db (metadata)
#         my_model/cache/embeddings.h5 (outputs)

# Random temporary directory if not specified
model = ESM2Embedding()
```

2.11 Position-Specific Models

2.11.1 Overview

Some protein models can generate outputs for each amino acid position in a sequence. These models use the `PositionSpecificMixin` to handle position selection and output formatting. EG. lanmguage models or one hot encodings. You might want to do this if only a few positions are changing among variants or you have a specific hypothesis about the importance of certain positions.

2.11.2 Using Position-Specific Models

Position-specific models have three key parameters that control their output. Flatten and pool are mutually exclusive.

```
from aide_predict import ESM2Embedding

# Basic usage - outputs pooled across all positions
model = ESM2Embedding(
    positions=None,  # Consider all positions
    pool='mean',      # Average across positions
    flatten=False    # because pooling by mean
)

# Position-specific - get embeddings for specific positions
model = ESM2Embedding(
    positions=[0, 1, 2],  # Only these positions
    pool=False,          # Keep positions separate
    flatten=True         # Flatten features for each position so we get a single vector
)
```

2.11.3 Output Shapes

The output shape depends on the parameter combination:

```
# Example with ESM2 (1280-dimensional embeddings)
X = ProteinSequences.from_fasta("sequences.fasta")

# Default: pooled across positions
model = ESM2Embedding(pool=True)
output = model.transform(X)  # Shape: (n_sequences, 1280)

# Selected positions, no pooling
model = ESM2Embedding(
    positions=[0, 1, 2],
    pool=False
)
output = model.transform(X)  # Shape: (n_sequences, 3, 1280)

# Selected positions, no pooling, flattened
model = ESM2Embedding(
    positions=[0, 1, 2],
    pool=False,
    flatten=True
)
output = model.transform(X)  # Shape: (n_sequences, 3*1280)
```

2.11.4 Position Specificity for Variable Length Sequences

In some cases models can be position specific even if not all sequences are the same length, such as when working with homologs. However, to map positions between sequences properly, we need to:

1. Know the positions of interest in a reference sequence (usually wild type)
2. Align all sequences
3. Map the reference positions to positions in the alignment

AIDE provides tools to handle this workflow:

```
# Start with unaligned sequences
X = ProteinSequences.from_fasta("sequences.fasta")
wt = X['wt']
wt_positions = [1, 2, 3]  # 0-indexed positions of interest in wild type

# Align sequences
X = X.align_all()
wt.msa = X

# Get alignment mapping and convert positions
alignment_mapping = X.get_alignment_mapping()
wt_alignment_mapping = alignment_mapping[wt.id]  # or use str(hash(wt)) if no ID
aligned_positions = wt_alignment_mapping[wt_positions]

# Now use these positions in any position-specific model
```

(continues on next page)

(continued from previous page)

```
model = MSATransformerEmbedding(
    positions=aligned_positions,
    pool=False,
    wt=wt,  # used to get the alignment to align incoming sequence to. Alternative, wt␣
↪can be None if all seqs in X have the msa attribute set to X

)
model.fit()
embeddings = model.transform(X)
```

2.11.5 Implementation Notes

- If `positions` is specified but `pool=True`, the model will first select the positions then pool across them
- `flatten=True` only applies when `pool=False` and there are multiple dimensions
- Models will raise an error if `positions` are specified but the sequences are not aligned or of fixed length

```

```

2.12 Contributing Models to AIDE

2.12.1 Overview

AIDE is designed to make it easy to wrap new protein prediction models into a scikit-learn compatible interface. This guide walks through the process of contributing a new model.

1. Setting Up Development Environment

```
git clone https://github.com/beckham-lab/aide_predict
cd aide_predict
conda env create -f environment.yaml
conda activate aide_predict
pip install -e ".[dev]"  # Installs in editable mode with development dependencies
```

2. Understanding Model Dependencies

AIDE uses a tiered dependency system to minimize conflicts and installation complexity:

1. **Base Dependencies**: If your model only needs numpy, scipy, scikit-learn, etc., it can be included in the base package.
2. **Optional Dependencies**: If your model needs additional pip-installable packages:
   - Create or update a `requirements-<feature>.txt` file
   - Example: `requirements-transformers.txt` for models using HuggingFace transformers
3. **Complex Dependencies**: If your model requires a specific environment or complex setup:
   - Package should be installed separately

- AIDE will call it via subprocess
- Model checks for environment variables pointing to installation
- Example: EVE model checking for `EVE_REPO` and `EVE_CONDA_ENV`

3. Creating the Model Class

Models should be placed in one of two directories:

- `aide_predict/bespoke_models/embedders/`: For models that create numerical features
- `aide_predict/bespoke_models/predictors/`: For models that predict protein properties

Basic structure:

```python
from aide_predict.bespoke_models.base import ProteinModelWrapper
from aide_predict.utils.common import MessageBool

# Check dependencies
try:
    import some_required_package
    AVAILABLE = MessageBool(True, "Model is available")
except ImportError:
    AVAILABLE = MessageBool(False, "Requires some_required_package")

class MyModel(ProteinModelWrapper):
    """Documentation in NumPy style.

    Parameters
    ----------
    param1 : type
        Description
    metadata_folder : str, optional
        Directory for model files
    wt : ProteinSequence, optional
        Wild-type sequence for comparative predictions

    Attributes
    ----------
    fitted_ : bool
        Whether model has been fitted
    """
    _available = AVAILABLE  # Class attribute for availability

    def __init__(self, param1, metadata_folder=None, wt=None, **kwargs):
        super().__init__(metadata_folder=metadata_folder, wt=wt, **kwargs)
        self.param1 = param1  # Save user parameters as attributes

    def _fit(self, X, y=None):
        """Fit the model. Called by public fit() method."""
        # Implementation
        self.fitted_ = True  # Mark as fitted
        return self
```

(continues on next page)

(continued from previous page)

```
    def _transform(self, X):
        """Transform sequences. Called by public transform() method."""
        # Implementation
        return features
```

4. Adding Model Requirements with Mixins

AIDE uses mixins to declare model requirements and capabilities. Common mixins:

```
# Input requirements
RequiresMSAForFitMixin          # Needs MSA for fit method. If not found, will attempt␣
↪to fall back to WT sequence msa
RequiresWTMSAMixin            # Needs a WT sequence with an msa
RequiresMSAPerSequenceMixin  # The model needs msas, but can handle having different␣
↪MSAs for each input. If inputs do not have MSAs, will attempt fall back to WT sequence␣
↪msa
RequiresFixedLengthMixin  # Sequences must be same length
RequiresStructureMixin    # Uses structural information
RequiresWTToFunctionMixin          # Needs wild-type sequence
RequiresWTDuringInferenceMixin # Model does its own normalization to any WT internally.␣
↪If not inheritted, aide will automatically normalize outputs to any WT sequence␣
↪provided


# Output capabilities
CanRegressMixin          # Can predict numeric values
PositionSpecificMixin    # Outputs per-position scores or embeddings

# Processing behavior
CacheMixin               # Enables result caching
AcceptsLowerCaseMixin    # Handles lowercase sequences
ExpectsNoFitMixin         # Does not require any inputs to the fit method
ShouldRefitOnSequencesMixin # restore sklearn default behaviour to refit when fit is␣
↪called or params are set. Be default, models do not refit.
```

Example with mixins:

```
class MyModel(
    RequiresMSAMixin,      # Needs MSA for training
    CanRegressMixin,       # Makes predictions
    PositionSpecificMixin, # Per-position outputs
    CacheMixin,           # Caches results
    ProteinModelWrapper    # Always last
):
    pass
```

Ensure that the `_avialable` attribute is set to a valid `MessageBool` object that is computed on import based on the availability of the model's dependencies.

5. Testing Your Model

If applicable, add scientific validation tests in `tests/test_not_base_models/`:

```
from aide_predict.bespoke_models.embedders.my_model import MyModel
def test_my_model_benchmark():
    """Test against published benchmark."""
    model = MyModel()
    score = model.score(benchmark_data)
    assert score >= expected_performance
```

Run the tests with `pytest tests/test_not_base_models/test_my_model.py`, and copy the results.

Ensure that this test is not tracked by coverage, as we do not run CI on non-base models that have additional dependencies:

Update `.coveragerc`:

```
omit =
    ... other omitted files are here ...
    aide_predict/bespoke_models/embedders/my_model.py
```

7. Expose your model so that AIDE can find it and test it against user data

Update `aide_predict/bespoke_models/__init__.py` to include your model in the `TOOLS` list:

```
from .embedders.my_model import MyModel

TOOLS = [
    ...other tools are here...
    MyModel
]
```

7. Submitting Your Contribution

1. Create a new branch
2. Implement your model in its own module
3. Add any tests
4. Submit a pull request, add any test results to the pull request so the expected performance can be verified

2.13 Structure Prediction with SoloSeq

We provide a wrapper interface to get protein structure predictions using SoloSeq, a deep learning model for protein structure prediction that requires no MSAs. It is recommended to use crystal structures or run AlphaFold2 for more accurate predictions if your task is deemed very structure sensitive.

2.13.1 Installation

SoloSeq requires additional setup beyond the base AIDE installation.

1. Follow the setup steps here

Once the environment is setup and unit tests pass:

2. Download the SoloSeq model weights:

```
bash scripts/download_openfold_soloseq_params.sh openfold/resources
```

3. Set environment variables (add to your `.bashrc` or equivalent):

```
export OPENFOLD_CONDA_ENV=openfold_env  # Name of conda environment
export OPENFOLD_REPO=/path/to/openfold  # Full path to OpenFold repo
```

2.13.2 Basic Usage

AIDE provides a simplified interface to SoloSeq for predicting protein structures:

```
from aide_predict import ProteinSequences
from aide_predict.utils.soloseq import run_soloseq

# Load sequences
sequences = ProteinSequences.from_fasta("proteins.fasta")

# Run prediction
pdb_paths = run_soloseq(
    sequences=sequences,
    output_dir="./predicted_structures"
)

# attach predicted structures to sequence using structure mapper
from aide_predict.utils.data_structures.structures import StructureMapper
mapper = StructureMapper("./predicted_structures")
mapper.assign_structures(sequences)
```

Command Line Interface

You can also run predictions directly from the command line:

```
python -m aide_predict.utils.soloseq proteins.fasta predicted_structures
```

2.13.3 Advanced Options

The function provides several options to control prediction:

```
pdb_paths = run_soloseq(
    sequences=sequences,
    output_dir="predicted_structures",
    use_gpu=True,          # Set to False for CPU-only
    skip_relaxation=False, # Skip refinement step
    save_embeddings=True,  # Keep ESM embeddings
    device="cuda:0",       # Specific GPU device
    force=False           # Force rerun of existing predictions
)
```

Command line equivalents:

```
python -m aide_predict.utils.soloseq proteins.fasta predicted_structures \
    --no_gpu \
    --skip_relaxation \
    --save_embeddings \
    --device cuda:1 \
    --force
```

2.14 Generating MSAs with MMseqs2

For problems where you have not already determined an MSA with another tool (eg. Jackhmmer, EVCouplings, MMseqs, etc.) AIDE provides a high lavel wrapper for generating Multiple Sequence Alignments (MSAs) using MMseqs2, implementing the sensitive search similar to colabfold. This can be useful when you need MSAs for models like EVMutation, MSATransformer, or EVE. This is literally just calling MMseqs with a few parameters set - all credit should go to the authors of MMseqs and Colabfold:

Steinegger M and Soeding J. MMseqs2 enables sensitive protein sequence searching for the analysis of massive data sets. Nature Biotechnology, doi: 10.1038/nbt.3988 (2017).

Mirdita, M., Schütze, K., Moriwaki, Y. et al. ColabFold: making protein folding accessible to all. Nat Methods 19, 679–682 (2022). https://doi.org/10.1038/s41592-022-01488-1

2.14.1 Installation

1. Ensure MMseqs2 is installed and available in your PATH:

```
conda install -c bioconda mmseqs2
```

2. Download the ColabFold database(s): https://colabfold.mmseqs.com/. You will need to point towards this database to run the search.

2.14.2 Basic Usage

Python Interface

```
from aide_predict import ProteinSequences
from aide_predict.utils.mmseqs_msa_search import run_mmseqs_search

# Load sequences
sequences = ProteinSequences.from_fasta("proteins.fasta")

# Generate MSAs
msa_paths = run_mmseqs_search(
    sequences=sequences,
    uniref_db="path/to/uniref30_2302",
    output_dir="./msas"
)

# Load MSAs for use with models
from aide_predict import ProteinSequences
msas = [ProteinSequences.from_a3m(path) for path in msa_paths]
```

Command Line Interface

You can also run MSA generation directly from the command line:

```
python -m aide_predict.utils.mmseqs_msa_search \
    proteins.fasta \
    path/to/uniref30_2302 \
    ./msas
```

2.14.3 Advanced Options

The search can be customized with several parameters:

```
msa_paths = run_mmseqs_search(
    sequences=sequences,
    uniref_db="path/to/uniref30_2302",
    output_dir="./msas",
    mode='sensitive',     # Search sensitivity: 'fast', 'standard', or 'sensitive'
    threads=8,           # Number of CPU threads
)
```

Command line equivalents:

```
python -m aide_predict.utils.mmseqs_msa_search \
    proteins.fasta \
    path/to/uniref30_2302 \
    ./msas \
    --mode sensitive \
    --threads 8 \
    --keep-tmp
```

2.14.4 Search Modes

Three sensitivity modes are available:

- `fast`: Quick search with sensitivity 4.0
- `standard`: Balanced approach with sensitivity 5.7 (default)
- `sensitive`: More thorough search with sensitivity 7.5

Higher sensitivity will find more distant homologs but takes longer to run.

2.14.5 Output Format

MSAs are generated in A3M format, one file per input sequence. The files are named based on the sequence IDs in your input FASTA file. These files can be directly used with AIDE's MSA-based models:

```
# Use MSA with a model
from aide_predict import MSATransformerLikelihoodWrapper

msa = ProteinSequences.from_a3m("msas/sequence1.a3m")
model = MSATransformerLikelihoodWrapper(wt=wt)
model.fit(msa)
```

2.15 Protein Optimization with BADASS

2.15.1 Overview

AIDE integrates BADASS, an adaptive simulated annealing algorithm that efficiently explores protein sequence space to find variants with optimal properties. The BADASS algorithm was introduced in this paper and has been adapted in AIDE to work with any of its protein prediction models.

2.15.2 Installation

To use BADASS with AIDE, install the required dependencies:

```
pip install -r requirements-badass.txt
```

2.15.3 Basic Usage

Here's a complete example of using BADASS with an ESM2 zero-shot predictor:

```
from aide_predict import ProteinSequence, ESM2LikelihoodWrapper
from aide_predict.utils.badass import BADASSOptimizer, BADASSOptimizerParams

# 1. Define your protein sequence and prediction model
wt = ProteinSequence(
↪"MKLLVLGLPGAGKGTQAEKIVAAYGIPHISTGDMFRAAMKEGTPLGLQAKQYMDEGDLVPDEVTIGIVRERLSKDDCQNGFLLDGFPRTVAQAEALETML
↪")
```

(continues on next page)

(continued from previous page)

```
# 2. Set up a prediction model
# Note that this can be a supervised model. In general, any ProteinModel or
# scikit-learn pipeline whose input models are ProteinModelWrapper can be used.
model = ESM2LikelihoodWrapper(wt=wt)
model.fit([])  # No training needed for zero-shot model

# 3. Configure optimization parameters
params = BADASSOptimizerParams(
    num_mutations=3,       # Maximum mutations per variant
    num_iter=100,          # Number of optimization iterations
    seqs_per_iter=200      # Sequences evaluated per iteration
)

# 4. Create and run the optimizer
optimizer = BADASSOptimizer(
    predictor=model.predict,
    reference_sequence=wt,
    params=params
)

# 5. Run optimization
# This returns protein variants as well as scores from the optimizer
# (which may be scaled and not equal to direct model outputs)
results_df, stats_df = optimizer.optimize()

# 6. Visualize the optimization process
optimizer.plot()

# 7. Print top variants
print(results_df.sort_values('scores', ascending=False).head(10))
```

2.15.4 Optimization Parameters

BADASS behavior can be extensively customized through the `BADASSOptimizerParams` class:

```
params = BADASSOptimizerParams(
    # Core parameters
    seqs_per_iter=500,              # Sequences per iteration
    num_iter=200,                   # Total optimization iterations
    num_mutations=5,                # Maximum mutations per variant
    init_score_batch_size=500,      # Batch size for initial scoring

    # Algorithm behavior
    temperature=1.5,                # Initial temperature
    cooling_rate=0.92,              # Cooling rate for SA
    seed=42,                        # Random seed
    gamma=0.5,                      # Variance boosting weight

    # Constraints
    sites_to_ignore=[1, 2, 3],      # Positions to exclude from mutation (1-indexed)
```

(continues on next page)

(continued from previous page)

```
    # Advanced options
    normalize_scores=True,           # Normalize scores
    simple_simulated_annealing=False, # Use simple SA without adaptation
    cool_then_heat=False,            # Use cooling-then-heating schedule
    adaptive_upper_threshold=None,   # Threshold for adaptivity (float for quantile, int␣
↪for top N)
    n_seqs_to_keep=None,             # Number of sequences to keep in results
    score_threshold=None,            # Score threshold for phase transitions (auto-
↪computed if None)
    reversal_threshold=None          # Score threshold for phase reversals (auto-computed␣
↪if None)
)
```

2.15.5 How BADASS Works

BADASS operates through the following key mechanisms:

1. **Initialization**: Computes a score matrix of all single-point mutations
2. **Sampling**: Uses Boltzmann sampling to generate candidate sequences
3. **Scoring**: Evaluates candidates with the provided predictor function
4. **Phase detection**: Identifies when the optimizer has found a promising region
5. **Adaptive temperature**: Adjusts temperature to balance exploration/exploitation
6. **Score normalization**: Standardizes scores for better comparison

During optimization, BADASS maintains several tracking matrices:

- Score matrix for each amino acid at each position
- Observation counts for statistical significance
- Variance estimates for uncertainty quantification

2.15.6 Optimization Results

The `optimize()` method returns two DataFrames:

1. `results_df`: Contains information about all evaluated sequences:
   - `sequences`: Compact mutation representation (e.g., “M1L-K5R”)
   - `scores`: Predicted fitness scores
   - `full_sequence`: Complete protein sequence
   - `counts`: Number of times each sequence was evaluated
   - `num_mutations`: Number of mutations in each sequence
   - `iteration`: When the sequence was first observed
2. `stats_df`: Contains statistics for each iteration:
   - `iteration`: Iteration number
   - `avg_score`: Average score per iteration

- var_score: Variance of scores
- n_eff_joint: Effective number of joint samples
- n_eff_sites: Effective number of sites explored
- n_eff_aa: Effective number of amino acids explored
- T: Temperature at each iteration
- n_seqs: Number of sequences evaluated
- n_new_seqs: Number of new sequences evaluated
- num_phase_transitions: Cumulative number of phase transitions

2.15.7 Analyzing Results

After optimization, BADASS offers several visualization and analysis options:

```
# Plot optimization progress
optimizer.plot()  # Creates multiple plots showing optimization trajectory

# Save results to CSV
optimizer.save_results("optimization_run")

# Get best sequences
best_sequences = results_df.sort_values('scores', ascending=False).head(10)

# Create a ProteinSequences object from best variants
from aide_predict import ProteinSequences
top_variants = ProteinSequences(best_sequences['full_sequence'].tolist())

# Further analyze with other AIDE tools
from aide_predict.utils.plotting import plot_mutation_heatmap
mutations = [seq.get_mutations(wt)[0] for seq in top_variants]
scores = best_sequences['scores'].values
plot_mutation_heatmap(mutations, scores)
```

The visualization includes:

1. Statistics by iteration (scores, effective samples, temperature)
2. Score distributions vs temperature
3. Score density distributions across early and late iterations

2.15.8 Performance Considerations

- BADASS evaluates thousands of sequences, so efficient predictors are important
- For computationally expensive models, consider:
  - Using model caching (via CacheMixin)
  - Reducing seqs_per_iter and num_iter
  - Using batch processing in custom predictors
  - Increasing init_score_batch_size for better initial sampling

2.15.9 References

- BADASS: biphasic annealing for diverse adaptive sequence sampling

2.16 Roadmap

2.16.1 Additional predictors from maintainers

Quarter 2, 2026

We have the following predictors planned to be wrapped:

1. ProteinMPNN (as a zero shot predictor)

- RequiresStructureMixin
- Does not require wildtype, eg. can do mutant marginal on variable length structures.
- Type 3 dependencies (sub environment)

2. NOMELT (as a zero shot predictor)

- Temperatute specific scores
- Type 3 dependencies

3. ESM3 (Zero shot prediction and embedder)

- Structure aware if available
- For now, the annotation data mode will not be considered. This will require an additional attribute of the ProteinSequence class (in addition to sequence, structure, msa data types already supported).

2.16.2 Contributions from the community

While we will maintain the codebase and address bugs identified by the community, the usefulness of the tool will ultimately require contributions from the community *a la* higgingface scikit-learn, etc. When / If we add (2) community contributed models we will start developping the next major version (v2)

2.16.3 Major update v2

Undetermined

The component specification and software engineering exercise conducted in AIDE for different types of predictors would also be helpful for the variable and dispirate **generator** methods available, eg. methods for producing new sequences. These broadly categorize into:

- Unconditional generators
- Conditional generators (eg. infilling, homolog aware, structure or other property conditioning)
  - ProteinMPNN
  - NOMELT
  - Tranception
- Score optimizers (black box or maybe gradient aware), eg. BADASS (already included in the package) which use a scoring function (AIDE predictor) to bias generation.

2.17 Resource testing

See below for the cost it takes to run each tool. This test was run with a Dual socket Intel Xeon Sapphire Rapids 52 core CPU. When the model supports GPU, one NVIDIA H100 was provided.

The test system was a GFP (238) amino acids, MSA depth (when applicable) was 201. Times measure the total time to fit the model (when applicable) and run prediction on 50 variants. Missing values are either because the core model does not support that type of prediction or because AIDE's wrapper does not support it.

NOTE: The cost of some of these (*) is significantly impacted by hyperparameters.

2.17.1 Zero shot predictors

Model Name	Marginal Method	GPU Total Time (s)	CPU Total Time (s)
HMMWrapper	-	-	0.136
ESM2LikelihoodWrapper	wildtype_marginal	0.980	2.560
ESM2LikelihoodWrapper	mutant_marginal	0.534	30.837
ESM2LikelihoodWrapper	masked_marginal	0.718	62.507
MSATransformerLikelihoodWrapper	wildtype_marginal	4.067	33.974
MSATransformerLikelihoodWrapper	mutant_marginal	57.297	Timeout (>1800s)
MSATransformerLikelihoodWrapper	masked_marginal	110.086	Timeout (>1800s)
EVMutationWrapper	-	-	96.697
SaProtLikelihoodWrapper	wildtype_marginal	5.356	24.291
SaProtLikelihoodWrapper	mutant_marginal	7.326	220.906
SaProtLikelihoodWrapper	masked_marginal	14.814	429.626
VESPAWrapper	-	244.852	-
EVEWrapper *	-	925.930	-
SSEmbWrapper	-	192.999	-

2.17.2 Embedders

Cost for embedding 21 GFP sequences.

Model Name	GPU Total Time (s)	CPU Total Time (s)
ESM2Embedding	0.887	1.477
OneHotAlignedEmbedding	-	0.092
OneHotProteinEmbedding	-	0.023
MSATransformerEmbedding	18.962	62.653
SaProtEmbedding	10.439	32.360
KmerEmbedding	-	0.005
SSEmbEmbedding	665.772	-

2.18 aide_predict

2.18.1 aide_predict package

Subpackages

aide_predict.bespoke_models package

Subpackages

aide_predict.bespoke_models.embedders package

Submodules

aide_predict.bespoke_models.embedders.esm2 module

- Author: Evan Komp
- Created: 7/5/2024
- Company: National Renewable Energy Lab, Bioeneergy Science and Technology
- License: MIT

ESM2 language model self supervised embeddings.

class aide_predict.bespoke_models.embedders.esm2.**ESM2Embedding**(*metadata_folder: str | None = None*, *model_checkpoint: str = 'esm2_t6_8M_UR50D'*, *layer: int = -1*, *positions: List[int] | None = None*, *flatten: bool = False*, *pool: bool | None = None*, *batch_size: int = 32*, *device: str = 'cpu'*, *wt: str | ProteinSequence | None = None*, ***kwargs*)

Bases: *ExpectsNoFitMixin*, *PositionSpecificMixin*, *CanHandleAlignedSequencesMixin*, *CacheMixin*, *ProteinModelWrapper*

A protein sequence embedder that uses the ESM2 model to generate embeddings.

This class wraps the ESM2 model to provide embeddings for protein sequences. It can handle both aligned and unaligned sequences and allows for retrieving embeddings from a specific layer of the model.

Variables

- **model_checkpoint** (*str*) – The name of the ESM2 model checkpoint to use.
- **layer** (*int*) – The layer from which to extract embeddings (-1 for last layer).
- **positions** (*Optional[List[int]]*) – Specific positions to encode. If None, all positions are encoded.
- **pool** (*bool*) – Whether to pool the encoded vectors across positions.
- **flatten** (*bool*) – Whether to flatten the output array.
- **batch_size** (*int*) – The batch size for processing sequences.

- **device** (*str*) – The device to use for computations ('cuda' or 'cpu').

__init__(*metadata_folder: str | None = None*, *model_checkpoint: str = 'esm2_t6_8M_UR50D'*, *layer: int = -1*, *positions: List[int] | None = None*, *flatten: bool = False*, *pool: bool | None = None*, *batch_size: int = 32*, *device: str = 'cpu'*, *wt: str |* ProteinSequence *| None = None*, ***kwargs*)

Initialize the ESM2Embedding.

Parameters

- **metadata_folder** (*str*) – The folder where metadata is stored.
- **model_checkpoint** (*str*) – The name of the ESM2 model checkpoint to use.
- **layer** (*int*) – The layer from which to extract embeddings (-1 for last layer).
- **positions** (*Optional[List[int]]*) – Specific positions to encode. If None, all positions are encoded.
- **flatten** (*bool*) – Whether to flatten the output array.
- **batch_size** (*int*) – The batch size for processing sequences.
- **device** (*str*) – The device to use for computations ('cuda' or 'cpu').
- **wt** (*Optional[Union[str, ProteinSequence]]*) – The wild type sequence, if any.

Notes: WT is set to None to avoid normalization. For an embedder this is effectively a feature scaler which you should do manually if you want

property accepts_lower_case: bool

Whether the model can accept lower case sequences.

property can_handle_aligned_sequences: bool

Whether the model can handle aligned sequences (with gaps) at predict time.

property can_regress: bool

Whether the model can perform regression.

check_metadata() → None

Ensures that everything this model class needs is in the metadata folder.

property expects_no_fit: bool

Whether the model expects no fit.

fit(*X:* ProteinSequences *| List[str] | None = None*, *y: ndarray | None = None*, *force: bool = False*) → *ProteinModelWrapper*

Fit the model with pre-processing and post-processing from mixins.

Parameters

- **X** (*Union[ProteinSequences, List[str]]*) – Input sequences.
- **y** (*Optional[np.ndarray]*) – Target values.
- **force** (*bool*) – Whether to force refitting if already fitted.

Returns

The fitted model.

Return type

ProteinModelWrapper

fit_transform(*X*, *y=None*, ***fit_params*)

Fit to data, then transform it.

Fits transformer to *X* and *y* with optional parameters *fit_params* and returns a transformed version of *X*.

Parameters

- **X** (*array-like of shape (n_samples, n_features)*) – Input samples.
- **y** (*array-like of shape (n_samples,) or (n_samples, n_outputs), default=None*) – Target values (None for unsupervised transformations).
- ****fit_params** (*dict*) – Additional fit parameters.

Returns

X_new – Transformed array.

Return type

ndarray array of shape (n_samples, n_features_new)

get_feature_names_out(*input_features: List[str] | None = None*) → List[str]

Get output feature names for transformation.

Parameters

input_features (*Optional[List[str]]*) – Ignored. Present for API consistency.

Returns

Output feature names.

Return type

List[str]

get_fitted_attributes() → List[str]

Get a list of attributes that are set during fitting.

get_metadata_routing()

Get metadata routing of this object.

Please check User Guide on how the routing mechanism works.

Returns

routing – A `MetadataRequest` encapsulating routing information.

Return type

MetadataRequest

get_params(*deep: bool = True*) → Dict[str, Any]

Get parameters for this estimator.

Parameters

deep (*bool*) – If True, will return the parameters for this estimator and contained subobjects.

Returns

Parameter names mapped to their values.

Return type

Dict[str, Any]

property metadata_folder

partial_fit(*X:* ProteinSequences *| List[str]*, *y: ndarray | None = None*) → *ProteinModelWrapper*

Partially fit the model to the given sequences.

This method can be called multiple times to incrementally fit the model.

Parameters

- **X** (*Union[ProteinSequences, List[str]]*) – The input sequences to partially fit the model on.
- **y** (*Optional[np.ndarray]*) – The target values, if applicable.

Returns
The partially fitted model.

Return type
ProteinModelWrapper

`property per_position_capable: bool`

Whether the model can output per position scores.

`predict`(*X:* ProteinSequences | *List[str]*) → ndarray

Predict the sequences.

Parameters
X (*Union[ProteinSequences, List[str]]*) – Input sequences.

Returns
Predicted values.

Return type
np.ndarray

Raises
ValueError – If the model is not capable of regression.

`property requires_fixed_length: bool`

Whether the model requires fixed length input.

`property requires_msa_for_fit: bool`

Whether the model requires an MSA for fitting.

`property requires_msa_per_sequence: bool`

Whether the model requires an MSA for each sequence during transform.

`property requires_structure: bool`

Whether the model requires structure information.

`property requires_wt_during_inference: bool`

Whether the model requires wild type during inference.

`property requires_wt_msa: bool`

Whether the model requires a wild type MSA for fitting.

`property requires_wt_to_function: bool`

Whether the model requires the wild type sequence to function.

`set_fit_request`(**, force: bool | None | str = '$UNCHANGED$'*) → *ESM2Embedding*

Request metadata passed to the `fit` method.

Note that this method is only relevant if `enable_metadata_routing=True` (see `sklearn.set_config()`). Please see User Guide on how the routing mechanism works.

The options for each parameter are:

- `True`: metadata is requested, and passed to `fit` if provided. The request is ignored if metadata is not provided.

- `False`: metadata is not requested and the meta-estimator will not pass it to `fit`.
- `None`: metadata is not requested, and the meta-estimator will raise an error if the user provides it.
- `str`: metadata should be passed to the meta-estimator with this given alias instead of the original name.

The default (`sklearn.utils.metadata_routing.UNCHANGED`) retains the existing request. This allows you to change the request for some parameters and not others.

Added in version 1.3.

Note: This method is only relevant if this estimator is used as a sub-estimator of a meta-estimator, e.g. used inside a `Pipeline`. Otherwise it has no effect.

Parameters
force (*str, True, False, or None, default=sklearn.utils.metadata_routing.UNCHANGED*) – Metadata routing for `force` parameter in `fit`.

Returns
self – The updated object.

Return type
object

`set_output`(*, *transform=None*)

Set output container.

See sphx_glr_auto_examples_miscellaneous_plot_set_output.py for an example on how to use the API.

Parameters
transform (*{"default", "pandas", "polars"}, default=None*) – Configure output of *transform* and *fit_transform*.

- *"default"*: Default output format of a transformer
- *"pandas"*: DataFrame output
- *"polars"*: Polars output
- *None*: Transform configuration is unchanged

Added in version 1.4: *"polars"* option was added.

Returns
self – Estimator instance.

Return type
estimator instance

`set_params`(***params: Any*) → *ProteinModelWrapper*

Set the parameters of this estimator.

Parameters
****params** – Estimator parameters.

Returns
Estimator instance.

Return type
ProteinModelWrapper

property should_refit_on_sequences: bool

Whether the model should refit on new sequences when given.

transform(*X: ProteinSequences | List[str]*) → ndarray

Transform sequences with pre-processing and post-processing from mixins.

Parameters

X (*Union[ProteinSequences, List[str]]*) – Input sequences.

Returns

Transformed sequences.

Return type

np.ndarray

property wt

aide_predict.bespoke_models.embedders.kmer module

- Author: Evan Komp
- Created: 8/9/2024
- Company: National Renewable Energy Lab, Bioeneergy Science and Technology
- License: MIT

class aide_predict.bespoke_models.embedders.kmer.**KmerEmbedding**(*metadata_folder: str | None = None, k: int = 3, normalize: bool = True, wt: ProteinSequence | None = None*)

Bases: *CanHandleAlignedSequencesMixin*, *ProteinModelWrapper*

A fast K-mer embedding class for protein sequences.

This class generates K-mer embeddings for protein sequences, handling both aligned and unaligned sequences efficiently.

Variables

- **k** (*int*) – The size of the K-mers.
- **normalize** (*bool*) – Whether to normalize the K-mer counts.

__init__(*metadata_folder: str | None = None, k: int = 3, normalize: bool = True, wt: ProteinSequence | None = None*)

Initialize the KmerEmbedding.

Parameters

- **metadata_folder** (*str*) – Folder to store metadata.
- **k** (*int*) – The size of the K-mers.
- **normalize** (*bool*) – Whether to normalize the K-mer counts.

property accepts_lower_case: bool

Whether the model can accept lower case sequences.

property can_handle_aligned_sequences: bool

Whether the model can handle aligned sequences (with gaps) at predict time.

property can_regress: bool

Whether the model can perform regression.

check_metadata() → None

Ensures that everything this model class needs is in the metadata folder.

property expects_no_fit: bool

Whether the model expects no fit.

fit(*X: ProteinSequences | List[str] | None = None*, *y: ndarray | None = None*, *force: bool = False*) → *ProteinModelWrapper*

Fit the model with pre-processing and post-processing from mixins.

Parameters

- **X** (*Union[ProteinSequences, List[str]]*) – Input sequences.
- **y** (*Optional[np.ndarray]*) – Target values.
- **force** (*bool*) – Whether to force refitting if already fitted.

Returns
The fitted model.

Return type
ProteinModelWrapper

fit_transform(*X*, *y=None*, ***fit_params*)

Fit to data, then transform it.

Fits transformer to *X* and *y* with optional parameters *fit_params* and returns a transformed version of *X*.

Parameters

- **X** (*array-like of shape (n_samples, n_features)*) – Input samples.
- **y** (*array-like of shape (n_samples,) or (n_samples, n_outputs), default=None*) – Target values (None for unsupervised transformations).
- ****fit_params** (*dict*) – Additional fit parameters.

Returns
X_new – Transformed array.

Return type
ndarray array of shape (n_samples, n_features_new)

get_feature_names_out(*input_features: List[str] | None = None*) → List[str]

Get output feature names for transformation.

Parameters
input_features (*Optional[List[str]]*) – Ignored. Present for API consistency.

Returns
Output feature names.

Return type
List[str]

get_metadata_routing()

Get metadata routing of this object.

Please check User Guide on how the routing mechanism works.

Returns
routing – A `MetadataRequest` encapsulating routing information.

Return type
MetadataRequest

get_params(*deep: bool = True*) → Dict[str, Any]

Get parameters for this estimator.

Parameters
deep (*bool*) – If True, will return the parameters for this estimator and contained subobjects.

Returns
Parameter names mapped to their values.

Return type
Dict[str, Any]

property metadata_folder

partial_fit(*X:* ProteinSequences | *List[str]*, *y: ndarray* | *None* = *None*) → *ProteinModelWrapper*

Partially fit the model to the given sequences.

This method can be called multiple times to incrementally fit the model.

Parameters

- **X** (*Union[*ProteinSequences*, List[str]]*) – The input sequences to partially fit the model on.
- **y** (*Optional[np.ndarray]*) – The target values, if applicable.

Returns
The partially fitted model.

Return type
ProteinModelWrapper

property per_position_capable: bool

Whether the model can output per position scores.

predict(*X:* ProteinSequences | *List[str]*) → ndarray

Predict the sequences.

Parameters
X (*Union[*ProteinSequences*, List[str]]*) – Input sequences.

Returns
Predicted values.

Return type
np.ndarray

Raises
ValueError – If the model is not capable of regression.

property requires_fixed_length: bool

Whether the model requires fixed length input.

property requires_msa_for_fit: bool

Whether the model requires an MSA for fitting.

property requires_msa_per_sequence: bool

Whether the model requires an MSA for each sequence during transform.

property requires_structure: bool

Whether the model requires structure information.

property requires_wt_during_inference: bool

Whether the model requires wild type during inference.

property requires_wt_msa: bool

Whether the model requires a wild type MSA for fitting.

property requires_wt_to_function: bool

Whether the model requires the wild type sequence to function.

set_fit_request(**, force: bool | None | str = '$UNCHANGED$'*) → *KmerEmbedding*

Request metadata passed to the `fit` method.

Note that this method is only relevant if `enable_metadata_routing=True` (see `sklearn.set_config()`). Please see User Guide on how the routing mechanism works.

The options for each parameter are:

- `True`: metadata is requested, and passed to `fit` if provided. The request is ignored if metadata is not provided.
- `False`: metadata is not requested and the meta-estimator will not pass it to `fit`.
- `None`: metadata is not requested, and the meta-estimator will raise an error if the user provides it.
- `str`: metadata should be passed to the meta-estimator with this given alias instead of the original name.

The default (`sklearn.utils.metadata_routing.UNCHANGED`) retains the existing request. This allows you to change the request for some parameters and not others.

Added in version 1.3.

Note: This method is only relevant if this estimator is used as a sub-estimator of a meta-estimator, e.g. used inside a `Pipeline`. Otherwise it has no effect.

Parameters

force (*str, True, False, or None, default=sklearn.utils.metadata_routing.UNCHANGED*) – Metadata routing for `force` parameter in `fit`.

Returns

self – The updated object.

Return type

object

set_output(**, transform=None*)

Set output container.

See sphx_glr_auto_examples_miscellaneous_plot_set_output.py for an example on how to use the API.

Parameters

transform (*{"default", "pandas", "polars"}, default=None*) – Configure output of *transform* and *fit_transform*.

- *"default"*: Default output format of a transformer
- *"pandas"*: DataFrame output
- *"polars"*: Polars output
- *None*: Transform configuration is unchanged

Added in version 1.4: *"polars"* option was added.

Returns
self – Estimator instance.

Return type
estimator instance

`set_params`(***params: Any*) → *ProteinModelWrapper*

Set the parameters of this estimator.

Parameters
`**params` – Estimator parameters.

Returns
Estimator instance.

Return type
ProteinModelWrapper

`property should_refit_on_sequences: bool`

Whether the model should refit on new sequences when given.

`transform`(*X:* ProteinSequences | *List[str]*) → ndarray

Transform sequences with pre-processing and post-processing from mixins.

Parameters
X (*Union[ProteinSequences, List[str]]*) – Input sequences.

Returns
Transformed sequences.

Return type
np.ndarray

`property wt`

aide_predict.bespoke_models.embedders.msa_transformer module

- Author: Evan Komp
- Created: 7/8/2024
- Company: National Renewable Energy Lab, Bioeneergy Science and Technology
- License: MIT

`class aide_predict.bespoke_models.embedders.msa_transformer.MSATransformerEmbedding`(*metadata_folder: str | None = None*, *layer: int = -1*, *positions: List[int] | None = None*, *flatten: bool = False*, *pool: bool = False*, *batch_size: int = 32*, *n_msa_seqs: int = 360*, *device: str = 'cpu'*, *use_cache: bool = True*, *wt: str | ProteinSequence | None = None*)

Bases: *PositionSpecificMixin*, *CanHandleAlignedSequencesMixin*, *RequiresMSAPerSequenceMixin*, *CacheMixin*, *ProteinModelWrapper*

A protein sequence embedder that uses the MSA Transformer model to generate embeddings.

This class wraps the MSA Transformer model to provide embeddings for protein sequences. It requires that each sequence has its own MSA. It can handle both aligned and unaligned sequences and allows for retrieving embeddings from a specific layer of the model.

Variables

- **layer** (*int*) – The layer from which to extract embeddings (-1 for last layer).
- **positions** (*Optional[List[int]]*) – Specific positions to encode. If None, all positions are encoded.
- **pool** (*bool*) – Whether to pool the encoded vectors across positions.

- **flatten** (*bool*) – Whether to flatten the output array.
- **batch_size** (*int*) – The batch size for processing sequences.
- **n_msa_seqs** (*int*) – The number of sequences to sample from each MSA.
- **device** (*str*) – The device to use for computations ('cuda' or 'cpu').

__init__(*metadata_folder: str | None = None*, *layer: int = -1*, *positions: List[int] | None = None*, *flatten: bool = False*, *pool: bool = False*, *batch_size: int = 32*, *n_msa_seqs: int = 360*, *device: str = 'cpu'*, *use_cache: bool = True*, *wt: str | ProteinSequence | None = None*)

Initialize the MSATransformerEmbedding.

Parameters

- **metadata_folder** (*str*) – The folder where metadata is stored.
- **layer** (*int*) – The layer from which to extract embeddings (-1 for last layer).
- **positions** (*Optional[List[int]]*) – Specific positions to encode. If None, all positions are encoded.
- **flatten** (*bool*) – Whether to flatten the output array.
- **pool** (*bool*) – Whether to pool the encoded vectors across positions.
- **batch_size** (*int*) – The batch size for processing MSA batches.
- **n_msa_seqs** (*int*) – The number of sequences to use from the MSA, sampled from the weight vector.
- **device** (*str*) – The device to use for computations ('cuda' or 'cpu').
- **use_cache** (*bool*) – Whether to cache results to avoid redundant computations.
- **wt** (*Optional[Union[str, ProteinSequence]]*) – The wild type sequence, if any.

property accepts_lower_case: bool

Whether the model can accept lower case sequences.

property can_handle_aligned_sequences: bool

Whether the model can handle aligned sequences (with gaps) at predict time.

property can_regress: bool

Whether the model can perform regression.

check_metadata() → None

Ensures that everything this model class needs is in the metadata folder.

property expects_no_fit: bool

Whether the model expects no fit.

fit(*X: ProteinSequences | List[str] | None = None*, *y: ndarray | None = None*, *force: bool = False*) → *ProteinModelWrapper*

Fit the model with pre-processing and post-processing from mixins.

Parameters

- **X** (*Union[ProteinSequences, List[str]]*) – Input sequences.
- **y** (*Optional[np.ndarray]*) – Target values.
- **force** (*bool*) – Whether to force refitting if already fitted.

Returns
The fitted model.

Return type
ProteinModelWrapper

`fit_transform`(*X*, *y=None*, ***fit_params*)

Fit to data, then transform it.

Fits transformer to *X* and *y* with optional parameters *fit_params* and returns a transformed version of *X*.

Parameters

- **X** (*array-like of shape (n_samples, n_features)*) – Input samples.
- **y** (*array-like of shape (n_samples,) or (n_samples, n_outputs), default=None*) – Target values (None for unsupervised transformations).
- ****fit_params** (*dict*) – Additional fit parameters.

Returns
X_new – Transformed array.

Return type
ndarray array of shape (n_samples, n_features_new)

`get_feature_names_out`(*input_features: List[str] | None = None*) → List[str]

Get output feature names for transformation.

Parameters
input_features (*Optional[List[str]]*) – Ignored. Present for API consistency.

Returns
Output feature names.

Return type
List[str]

`get_fitted_attributes`() → List[str]

Get a list of attributes that are set during fitting.

`get_metadata_routing`()

Get metadata routing of this object.

Please check User Guide on how the routing mechanism works.

Returns
routing – A `MetadataRequest` encapsulating routing information.

Return type
MetadataRequest

`get_params`(*deep: bool = True*) → Dict[str, Any]

Get parameters for this estimator.

Parameters
deep (*bool*) – If True, will return the parameters for this estimator and contained subobjects.

Returns
Parameter names mapped to their values.

Return type
Dict[str, Any]

property metadata_folder

partial_fit(*X: ProteinSequences | List[str], y: ndarray | None = None*) → *ProteinModelWrapper*

Partially fit the model to the given sequences.

This method can be called multiple times to incrementally fit the model.

Parameters

- **X** (*Union[ProteinSequences, List[str]]*) – The input sequences to partially fit the model on.
- **y** (*Optional[np.ndarray]*) – The target values, if applicable.

Returns

The partially fitted model.

Return type

ProteinModelWrapper

property per_position_capable: bool

Whether the model can output per position scores.

predict(*X: ProteinSequences | List[str]*) → ndarray

Predict the sequences.

Parameters

X (*Union[ProteinSequences, List[str]]*) – Input sequences.

Returns

Predicted values.

Return type

np.ndarray

Raises

ValueError – If the model is not capable of regression.

property requires_fixed_length: bool

Whether the model requires fixed length input.

property requires_msa_for_fit: bool

Whether the model requires an MSA for fitting.

property requires_msa_per_sequence: bool

Whether the model requires an MSA for each sequence during transform.

property requires_structure: bool

Whether the model requires structure information.

property requires_wt_during_inference: bool

Whether the model requires wild type during inference.

property requires_wt_msa: bool

Whether the model requires a wild type MSA for fitting.

property requires_wt_to_function: bool

Whether the model requires the wild type sequence to function.

set_fit_request(*, *force: bool | None | str = '$UNCHANGED$'*) → *MSATransformerEmbedding*

Request metadata passed to the `fit` method.

Note that this method is only relevant if `enable_metadata_routing=True` (see `sklearn.set_config()`). Please see User Guide on how the routing mechanism works.

The options for each parameter are:

- `True`: metadata is requested, and passed to `fit` if provided. The request is ignored if metadata is not provided.
- `False`: metadata is not requested and the meta-estimator will not pass it to `fit`.
- `None`: metadata is not requested, and the meta-estimator will raise an error if the user provides it.
- `str`: metadata should be passed to the meta-estimator with this given alias instead of the original name.

The default (`sklearn.utils.metadata_routing.UNCHANGED`) retains the existing request. This allows you to change the request for some parameters and not others.

Added in version 1.3.

Note: This method is only relevant if this estimator is used as a sub-estimator of a meta-estimator, e.g. used inside a `Pipeline`. Otherwise it has no effect.

Parameters
: **force** (*str, True, False, or None, default=sklearn.utils.metadata_routing.UNCHANGED*) – Metadata routing for `force` parameter in `fit`.

Returns
: **self** – The updated object.

Return type
: object

set_output(*, *transform=None*)

Set output container.

See sphx_glr_auto_examples_miscellaneous_plot_set_output.py for an example on how to use the API.

Parameters
: **transform** (*{"default", "pandas", "polars"}, default=None*) – Configure output of *transform* and *fit_transform*.

- *"default"*: Default output format of a transformer
- *"pandas"*: DataFrame output
- *"polars"*: Polars output
- *None*: Transform configuration is unchanged

Added in version 1.4: *"polars"* option was added.

Returns
: **self** – Estimator instance.

Return type
: estimator instance

set_params(***params: Any*) → *ProteinModelWrapper*

Set the parameters of this estimator.

Parameters
****params** – Estimator parameters.

Returns
Estimator instance.

Return type
ProteinModelWrapper

property should_refit_on_sequences: bool

Whether the model should refit on new sequences when given.

transform(*X:* ProteinSequences | *List[str]*) → ndarray

Transform sequences with pre-processing and post-processing from mixins.

Parameters
X (*Union[ProteinSequences, List[str]]*) – Input sequences.

Returns
Transformed sequences.

Return type
np.ndarray

property wt

aide_predict.bespoke_models.embedders.ohe module

- Author: Evan Komp
- Created: 7/5/2024
- Company: National Renewable Energy Lab, Bioeneergy Science and Technology
- License: MIT

Two classes: OneHotProteinEmbedding for fixed length sequences and OneHotAlignmentEmbedding which will dynamically align sequences to reference alignment before encoding.

class aide_predict.bespoke_models.embedders.ohe.**OneHotAlignedEmbedding**(*metadata_folder: str, wt: str |* ProteinSequence *| None = None, positions: List[int] | None = None, flatten: bool = True, pool: bool = False*)

Bases: *ShouldRefitOnSequencesMixin*, *PositionSpecificMixin*, *RequiresMSAForFitMixin*, *CanHandleAlignedSequencesMixin*, *ProteinModelWrapper*

A protein sequence embedder that performs one-hot encoding for aligned sequences.

This class allows for variable-length sequences and requires an MSA for fitting. It creates an encoding on the alignment including gaps. At prediction time, it can handle both aligned and unaligned sequences.

Variables

- **vocab** (*List[str]*) – The vocabulary of amino acids and gap characters used for encoding.

- **encoder** (*OneHotEncoder*) – The underlying sklearn OneHotEncoder.
- **positions** (*Optional[List[int]]*) – Specific positions to encode. If None, all positions are encoded.
- **pool** (*bool*) – Whether to pool the encoded vectors across positions.
- **flatten** (*bool*) – Whether to flatten the output array.
- **alignment_width** (*int*) – The width of the original alignment.
- **original_alignment** (*ProteinSequences*) – The original alignment used for fitting.

__init__(*metadata_folder: str*, *wt: str | ProteinSequence | None = None*, *positions: List[int] | None = None*, *flatten: bool = True*, *pool: bool = False*)

Initialize the OneHotAlignedEmbedding.

Parameters

- **metadata_folder** (*str*) – The folder where metadata is stored.
- **wt** (*Optional[Union[str, ProteinSequence]]*) – The wild type sequence, if any.
- **positions** (*Optional[List[int]]*) – Specific positions to encode. If None, all positions are encoded.
- **flatten** (*bool*) – Whether to flatten the output array.
- **pool** (*bool*) – Ignored

Notes: WT is set to None to avoid normalization. For an embedder this is effectively a feature scaler which you should do manually if you want

property accepts_lower_case: bool

Whether the model can accept lower case sequences.

property can_handle_aligned_sequences: bool

Whether the model can handle aligned sequences (with gaps) at predict time.

property can_regress: bool

Whether the model can perform regression.

check_metadata() → None

Ensures that everything this model class needs is in the metadata folder.

property expects_no_fit: bool

Whether the model expects no fit.

fit(*X: ProteinSequences | List[str] | None = None*, *y: ndarray | None = None*, *force: bool = False*) → *ProteinModelWrapper*

Fit the model with pre-processing and post-processing from mixins.

Parameters

- **X** (*Union[ProteinSequences, List[str]]*) – Input sequences.
- **y** (*Optional[np.ndarray]*) – Target values.
- **force** (*bool*) – Whether to force refitting if already fitted.

Returns

The fitted model.

Return type

ProteinModelWrapper

fit_transform(*X*, *y=None*, ***fit_params*)

Fit to data, then transform it.

Fits transformer to *X* and *y* with optional parameters *fit_params* and returns a transformed version of *X*.

Parameters

- **X** (*array-like of shape (n_samples, n_features)*) – Input samples.
- **y** (*array-like of shape (n_samples,) or (n_samples, n_outputs), default=None*) – Target values (None for unsupervised transformations).
- ****fit_params** (*dict*) – Additional fit parameters.

Returns

X_new – Transformed array.

Return type

ndarray array of shape (n_samples, n_features_new)

get_feature_names_out(*input_features: List[str] | None = None*) → List[str]

Get output feature names for transformation.

Parameters

input_features (*Optional[List[str]]*) – Ignored. Present for API consistency.

Returns

Output feature names.

Return type

List[str]

get_metadata_routing()

Get metadata routing of this object.

Please check User Guide on how the routing mechanism works.

Returns

routing – A `MetadataRequest` encapsulating routing information.

Return type

MetadataRequest

get_params(*deep: bool = True*) → Dict[str, Any]

Get parameters for this estimator.

Parameters

deep (*bool*) – If True, will return the parameters for this estimator and contained subobjects.

Returns

Parameter names mapped to their values.

Return type

Dict[str, Any]

property metadata_folder

partial_fit(*X: ProteinSequences | List[str]*, *y: ndarray | None = None*) → *ProteinModelWrapper*

Partially fit the model to the given sequences.

This method can be called multiple times to incrementally fit the model.

Parameters

- **X** (*Union*[`ProteinSequences`, *List[str]*]) – The input sequences to partially fit the model on.
- **y** (*Optional[np.ndarray]*) – The target values, if applicable.

Returns
The partially fitted model.

Return type
ProteinModelWrapper

property per_position_capable: bool

Whether the model can output per position scores.

predict(*X: ProteinSequences | List[str]*) → ndarray

Predict the sequences.

Parameters
X (*Union*[`ProteinSequences`, *List[str]*]) – Input sequences.

Returns
Predicted values.

Return type
np.ndarray

Raises
ValueError – If the model is not capable of regression.

property requires_fixed_length: bool

Whether the model requires fixed length input.

property requires_msa_for_fit: bool

Whether the model requires an MSA for fitting.

property requires_msa_per_sequence: bool

Whether the model requires an MSA for each sequence during transform.

property requires_structure: bool

Whether the model requires structure information.

property requires_wt_during_inference: bool

Whether the model requires wild type during inference.

property requires_wt_msa: bool

Whether the model requires a wild type MSA for fitting.

property requires_wt_to_function: bool

Whether the model requires the wild type sequence to function.

set_fit_request(**, force: bool | None | str = '$UNCHANGED$'*) → *OneHotAlignedEmbedding*

Request metadata passed to the `fit` method.

Note that this method is only relevant if `enable_metadata_routing=True` (see `sklearn.set_config()`). Please see User Guide on how the routing mechanism works.

The options for each parameter are:

- `True`: metadata is requested, and passed to `fit` if provided. The request is ignored if metadata is not provided.
- `False`: metadata is not requested and the meta-estimator will not pass it to `fit`.

- `None`: metadata is not requested, and the meta-estimator will raise an error if the user provides it.
- `str`: metadata should be passed to the meta-estimator with this given alias instead of the original name.

The default (`sklearn.utils.metadata_routing.UNCHANGED`) retains the existing request. This allows you to change the request for some parameters and not others.

Added in version 1.3.

Note: This method is only relevant if this estimator is used as a sub-estimator of a meta-estimator, e.g. used inside a `Pipeline`. Otherwise it has no effect.

Parameters
force (*str, True, False, or None, default=sklearn.utils.metadata_routing.UNCHANGED*) – Metadata routing for `force` parameter in `fit`.

Returns
self – The updated object.

Return type
object

`set_output`(*, *transform=None*)

Set output container.

See sphx_glr_auto_examples_miscellaneous_plot_set_output.py for an example on how to use the API.

Parameters
transform (*{"default", "pandas", "polars"}, default=None*) – Configure output of *transform* and *fit_transform*.

- *"default"*: Default output format of a transformer
- *"pandas"*: DataFrame output
- *"polars"*: Polars output
- *None*: Transform configuration is unchanged

Added in version 1.4: *"polars"* option was added.

Returns
self – Estimator instance.

Return type
estimator instance

`set_params`(***params: Any*) → *ProteinModelWrapper*

Set the parameters of this estimator.

Parameters
****params** – Estimator parameters.

Returns
Estimator instance.

Return type
ProteinModelWrapper

property should_refit_on_sequences: bool

Whether the model should refit on new sequences when given.

transform(*X: ProteinSequences | List[str]*) → ndarray

Transform sequences with pre-processing and post-processing from mixins.

Parameters
X (*Union[ProteinSequences, List[str]]*) – Input sequences.

Returns
Transformed sequences.

Return type
np.ndarray

property wt

class aide_predict.bespoke_models.embedders.ohe.**OneHotProteinEmbedding**(*metadata_folder: str | None = None, wt: str | ProteinSequence | None = None, positions: List[int] | None = None, flatten: bool = True, pool: bool = False*)

Bases: *PositionSpecificMixin*, *RequiresFixedLengthMixin*, *ProteinModelWrapper*

A protein sequence embedder that performs one-hot encoding with position-specific capabilities.

This class wraps sklearn's OneHotEncoder to provide one-hot encoding specifically for protein sequences. It expects fixed-length sequences without gaps and uses a 20 amino acid vocabulary. It also allows for position-specific encoding.

Variables

- **vocab** (*List[str]*) – The vocabulary of amino acids used for encoding.
- **encoder** (*OneHotEncoder*) – The underlying sklearn OneHotEncoder.
- **positions** (*Optional[List[int]]*) – Specific positions to encode. If None, all positions are encoded.
- **pool** (*bool*) – Ignored
- **flatten** (*bool*) – Whether to flatten the output array.
- **seq_length** (*Optional[int]*) – The length of the sequences, determined during fitting.

__init__(*metadata_folder: str | None = None, wt: str | ProteinSequence | None = None, positions: List[int] | None = None, flatten: bool = True, pool: bool = False*)

Initialize the OneHotProteinEmbedding.

Parameters

- **metadata_folder** (*str*) – The folder where metadata is stored.
- **wt** (*Optional[Union[str, ProteinSequence]]*) – The wild type sequence, if any.
- **positions** (*Optional[List[int]]*) – Specific positions to encode. If None, all positions are encoded.
- **flatten** (*bool*) – Whether to flatten the output array.

Notes: WT is set to None to avoid normalization. For an embedder this is effectively a feature scaler which you should do manually if you want

property accepts_lower_case: bool

Whether the model can accept lower case sequences.

property can_handle_aligned_sequences: bool

Whether the model can handle aligned sequences (with gaps) at predict time.

property can_regress: bool

Whether the model can perform regression.

check_metadata() → None

Ensures that everything this model class needs is in the metadata folder.

property expects_no_fit: bool

Whether the model expects no fit.

fit(*X: ProteinSequences | List[str] | None = None*, *y: ndarray | None = None*, *force: bool = False*) → *ProteinModelWrapper*

Fit the model with pre-processing and post-processing from mixins.

Parameters

- **X** (*Union[ProteinSequences, List[str]]*) – Input sequences.
- **y** (*Optional[np.ndarray]*) – Target values.
- **force** (*bool*) – Whether to force refitting if already fitted.

Returns
The fitted model.

Return type
ProteinModelWrapper

fit_transform(*X*, *y=None*, ***fit_params*)

Fit to data, then transform it.

Fits transformer to *X* and *y* with optional parameters *fit_params* and returns a transformed version of *X*.

Parameters

- **X** (*array-like of shape (n_samples, n_features)*) – Input samples.
- **y** (*array-like of shape (n_samples,) or (n_samples, n_outputs), default=None*) – Target values (None for unsupervised transformations).
- ****fit_params** (*dict*) – Additional fit parameters.

Returns
X_new – Transformed array.

Return type
ndarray array of shape (n_samples, n_features_new)

get_feature_names_out(*input_features: List[str] | None = None*) → List[str]

Get output feature names for transformation.

Parameters
input_features (*Optional[List[str]]*) – Ignored. Present for API consistency.

Returns
Output feature names.

Return type
List[str]

get_metadata_routing()

Get metadata routing of this object.

Please check User Guide on how the routing mechanism works.

Returns
routing – A `MetadataRequest` encapsulating routing information.

Return type
MetadataRequest

get_params(*deep: bool = True*) → Dict[str, Any]

Get parameters for this estimator.

Parameters
deep (*bool*) – If True, will return the parameters for this estimator and contained subobjects.

Returns
Parameter names mapped to their values.

Return type
Dict[str, Any]

inverse_transform(*X: ndarray*) → *ProteinSequences*

Convert one-hot encoded vectors back into protein sequences.

Parameters
X (*np.ndarray*) – The one-hot encoded sequences to inverse transform.

Returns
The reconstructed protein sequences.

Return type
ProteinSequences

Raises
ValueError – If the input shape is incompatible with the encoder's expectations.

property metadata_folder

partial_fit(*X:* ProteinSequences | *List[str]*, *y: ndarray* | *None = None*) → *ProteinModelWrapper*

Partially fit the model to the given sequences.

This method can be called multiple times to incrementally fit the model.

Parameters

- **X** (*Union[ProteinSequences, List[str]]*) – The input sequences to partially fit the model on.
- **y** (*Optional[np.ndarray]*) – The target values, if applicable.

Returns
The partially fitted model.

Return type
ProteinModelWrapper

property per_position_capable: bool

Whether the model can output per position scores.

`predict`(*X: ProteinSequences | List[str]*) → ndarray

Predict the sequences.

Parameters
: **X** (*Union[ProteinSequences, List[str]]*) – Input sequences.

Returns
: Predicted values.

Return type
: np.ndarray

Raises
: **ValueError** – If the model is not capable of regression.

property requires_fixed_length: bool

Whether the model requires fixed length input.

property requires_msa_for_fit: bool

Whether the model requires an MSA for fitting.

property requires_msa_per_sequence: bool

Whether the model requires an MSA for each sequence during transform.

property requires_structure: bool

Whether the model requires structure information.

property requires_wt_during_inference: bool

Whether the model requires wild type during inference.

property requires_wt_msa: bool

Whether the model requires a wild type MSA for fitting.

property requires_wt_to_function: bool

Whether the model requires the wild type sequence to function.

`set_fit_request`(**, force: bool | None | str = '$UNCHANGED$'*) → *OneHotProteinEmbedding*

Request metadata passed to the `fit` method.

Note that this method is only relevant if `enable_metadata_routing=True` (see `sklearn.set_config()`). Please see User Guide on how the routing mechanism works.

The options for each parameter are:

- `True`: metadata is requested, and passed to `fit` if provided. The request is ignored if metadata is not provided.
- `False`: metadata is not requested and the meta-estimator will not pass it to `fit`.
- `None`: metadata is not requested, and the meta-estimator will raise an error if the user provides it.
- `str`: metadata should be passed to the meta-estimator with this given alias instead of the original name.

The default (`sklearn.utils.metadata_routing.UNCHANGED`) retains the existing request. This allows you to change the request for some parameters and not others.

Added in version 1.3.

Note: This method is only relevant if this estimator is used as a sub-estimator of a meta-estimator, e.g. used inside a `Pipeline`. Otherwise it has no effect.

Parameters
`force` (*str, True, False, or None, default=sklearn.utils.metadata_routing.UNCHANGED*) – Metadata routing for `force` parameter in `fit`.

Returns
self – The updated object.

Return type
object

`set_output`(*, *transform=None*)

Set output container.

See sphx_glr_auto_examples_miscellaneous_plot_set_output.py for an example on how to use the API.

Parameters
`transform` (*{"default", "pandas", "polars"}, default=None*) – Configure output of *transform* and *fit_transform*.

- *"default"*: Default output format of a transformer
- *"pandas"*: DataFrame output
- *"polars"*: Polars output
- *None*: Transform configuration is unchanged

Added in version 1.4: *"polars"* option was added.

Returns
self – Estimator instance.

Return type
estimator instance

`set_params`(***params: Any*) → *ProteinModelWrapper*

Set the parameters of this estimator.

Parameters
`**params` – Estimator parameters.

Returns
Estimator instance.

Return type
ProteinModelWrapper

`property should_refit_on_sequences: bool`

Whether the model should refit on new sequences when given.

`transform`(*X:* ProteinSequences | *List[str]*) → ndarray

Transform sequences with pre-processing and post-processing from mixins.

Parameters
`X` (*Union[ProteinSequences, List[str]]*) – Input sequences.

Returns
Transformed sequences.

Return type
np.ndarray

`property wt`

aide_predict.bespoke_models.embedders.saprot module

- Author: Evan Komp
- Created: 7/16/2024
- Company: National Renewable Energy Lab, Bioeneergy Science and Technology
- License: MIT

class aide_predict.bespoke_models.embedders.saprot.**SaProtEmbedding**(*metadata_folder: str | None = None*, *model_checkpoint: str = 'westlake-repl/SaProt_650M_AF2'*, *layer: int = -1*, *positions: List[int] | None = None*, *flatten: bool = False*, *pool: bool = False*, *batch_size: int = 32*, *device: str = 'cpu'*, *foldseek_path: str = 'foldseek'*, *wt: str | ProteinSequence | None = None*, ***kwargs*)

Bases: *RequiresStructureMixin*, *ExpectsNoFitMixin*, *PositionSpecificMixin*, *CacheMixin*, *ProteinModelWrapper*

A protein sequence embedder that uses the SaProt model to generate embeddings.

This class wraps the SaProt model to provide embeddings for protein sequences. It can handle both aligned and unaligned sequences and allows for retrieving embeddings from a specific layer of the model.

Variables

- **model_checkpoint** (*str*) – The name of the SaProt model checkpoint to use.
- **layer** (*int*) – The layer from which to extract embeddings (-1 for last layer).
- **positions** (*Optional[List[int]]*) – Specific positions to encode. If None, all positions are encoded.
- **pool** (*bool*) – Whether to pool the encoded vectors across positions.
- **flatten** (*bool*) – Whether to flatten the output array.
- **batch_size** (*int*) – The batch size for processing sequences.
- **device** (*str*) – The device to use for computations (‘cuda’ or ‘cpu’).
- **foldseek_path** (*str*) – Path to the FoldSeek executable.

__init__(*metadata_folder: str | None = None*, *model_checkpoint: str = 'westlake-repl/SaProt_650M_AF2'*, *layer: int = -1*, *positions: List[int] | None = None*, *flatten: bool = False*, *pool: bool = False*, *batch_size: int = 32*, *device: str = 'cpu'*, *foldseek_path: str = 'foldseek'*, *wt: str | ProteinSequence | None = None*, ***kwargs*)

Initialize the SaProtEmbedding.

Parameters

- **metadata_folder** (*str*) – The folder where metadata is stored.
- **model_checkpoint** (*str*) – The name of the SaProt model checkpoint to use.
- **layer** (*int*) – The layer from which to extract embeddings (-1 for last layer).

- **positions** (*Optional[List[int]]*) – Specific positions to encode. If None, all positions are encoded.
- **flatten** (*bool*) – Whether to flatten the output array.
- **pool** (*bool*) – Whether to pool the encoded vectors across positions.
- **batch_size** (*int*) – The batch size for processing sequences.
- **device** (*str*) – The device to use for computations ('cuda' or 'cpu').
- **foldseek_path** (*str*) – Path to the FoldSeek executable.
- **wt** (*Optional[Union[str, ProteinSequence]]*) – The wild type sequence, if any.

property accepts_lower_case: bool

Whether the model can accept lower case sequences.

property can_handle_aligned_sequences: bool

Whether the model can handle aligned sequences (with gaps) at predict time.

property can_regress: bool

Whether the model can perform regression.

check_metadata() → None

Ensures that everything this model class needs is in the metadata folder.

property expects_no_fit: bool

Whether the model expects no fit.

fit(*X: ProteinSequences | List[str] | None = None*, *y: ndarray | None = None*, *force: bool = False*) → *ProteinModelWrapper*

Fit the model with pre-processing and post-processing from mixins.

Parameters

- **X** (*Union[ProteinSequences, List[str]]*) – Input sequences.
- **y** (*Optional[np.ndarray]*) – Target values.
- **force** (*bool*) – Whether to force refitting if already fitted.

Returns

The fitted model.

Return type

ProteinModelWrapper

fit_transform(*X*, *y=None*, ***fit_params*)

Fit to data, then transform it.

Fits transformer to *X* and *y* with optional parameters *fit_params* and returns a transformed version of *X*.

Parameters

- **X** (*array-like of shape (n_samples, n_features)*) – Input samples.
- **y** (*array-like of shape (n_samples,) or (n_samples, n_outputs), default=None*) – Target values (None for unsupervised transformations).
- ****fit_params** (*dict*) – Additional fit parameters.

Returns

X_new – Transformed array.

Return type
ndarray array of shape (n_samples, n_features_new)

`get_feature_names_out`(*input_features: List[str] | None = None*) → List[str]

Get output feature names for transformation.

Parameters
input_features (*Optional[List[str]]*) – Ignored. Present for API consistency.

Returns
Output feature names.

Return type
List[str]

`get_fitted_attributes`() → List[str]

Get a list of attributes that are set during fitting.

`get_metadata_routing`()

Get metadata routing of this object.

Please check User Guide on how the routing mechanism works.

Returns
routing – A `MetadataRequest` encapsulating routing information.

Return type
MetadataRequest

`get_params`(*deep: bool = True*) → Dict[str, Any]

Get parameters for this estimator.

Parameters
deep (*bool*) – If True, will return the parameters for this estimator and contained subobjects.

Returns
Parameter names mapped to their values.

Return type
Dict[str, Any]

`property metadata_folder`

`partial_fit`(*X: ProteinSequences | List[str], y: ndarray | None = None*) → *ProteinModelWrapper*

Partially fit the model to the given sequences.

This method can be called multiple times to incrementally fit the model.

Parameters

- **X** (*Union[ProteinSequences, List[str]]*) – The input sequences to partially fit the model on.
- **y** (*Optional[np.ndarray]*) – The target values, if applicable.

Returns
The partially fitted model.

Return type
ProteinModelWrapper

`property per_position_capable: bool`

Whether the model can output per position scores.

predict(*X: ProteinSequences | List[str]*) → ndarray

Predict the sequences.

Parameters
`X` (*Union[ProteinSequences, List[str]]*) – Input sequences.

Returns
Predicted values.

Return type
np.ndarray

Raises
`ValueError` – If the model is not capable of regression.

property requires_fixed_length: bool

Whether the model requires fixed length input.

property requires_msa_for_fit: bool

Whether the model requires an MSA for fitting.

property requires_msa_per_sequence: bool

Whether the model requires an MSA for each sequence during transform.

property requires_structure: bool

Whether the model requires structure information.

property requires_wt_during_inference: bool

Whether the model requires wild type during inference.

property requires_wt_msa: bool

Whether the model requires a wild type MSA for fitting.

property requires_wt_to_function: bool

Whether the model requires the wild type sequence to function.

set_fit_request(**, force: bool | None | str = '$UNCHANGED$'*) → *SaProtEmbedding*

Request metadata passed to the `fit` method.

Note that this method is only relevant if `enable_metadata_routing=True` (see `sklearn.set_config()`). Please see User Guide on how the routing mechanism works.

The options for each parameter are:

- `True`: metadata is requested, and passed to `fit` if provided. The request is ignored if metadata is not provided.
- `False`: metadata is not requested and the meta-estimator will not pass it to `fit`.
- `None`: metadata is not requested, and the meta-estimator will raise an error if the user provides it.
- `str`: metadata should be passed to the meta-estimator with this given alias instead of the original name.

The default (`sklearn.utils.metadata_routing.UNCHANGED`) retains the existing request. This allows you to change the request for some parameters and not others.

Added in version 1.3.

Note: This method is only relevant if this estimator is used as a sub-estimator of a meta-estimator, e.g. used inside a `Pipeline`. Otherwise it has no effect.

Parameters
`force` (*str, True, False, or None, default=sklearn.utils.metadata_routing.UNCHANGED*) – Metadata routing for `force` parameter in `fit`.

Returns
self – The updated object.

Return type
object

`set_output`(*, *transform=None*)

Set output container.

See sphx_glr_auto_examples_miscellaneous_plot_set_output.py for an example on how to use the API.

Parameters
`transform` (*{"default", "pandas", "polars"}, default=None*) – Configure output of *transform* and *fit_transform*.

- *"default"*: Default output format of a transformer
- *"pandas"*: DataFrame output
- *"polars"*: Polars output
- *None*: Transform configuration is unchanged

Added in version 1.4: *"polars"* option was added.

Returns
self – Estimator instance.

Return type
estimator instance

`set_params`(***params: Any*) → *ProteinModelWrapper*

Set the parameters of this estimator.

Parameters
`**params` – Estimator parameters.

Returns
Estimator instance.

Return type
ProteinModelWrapper

`property should_refit_on_sequences: bool`

Whether the model should refit on new sequences when given.

`transform`(*X: ProteinSequences | List[str]*) → ndarray

Transform sequences with pre-processing and post-processing from mixins.

Parameters
`X` (*Union[ProteinSequences, List[str]]*) – Input sequences.

Returns
Transformed sequences.

Return type
np.ndarray

`property wt`

Module contents

- Author: Evan Komp
- Created: 7/5/2024
- Company: National Renewable Energy Lab, Bioeneergy Science and Technology
- License: MIT

aide_predict.bespoke_models.predictors package

Submodules

aide_predict.bespoke_models.predictors.esm2 module

- Author: Evan Komp
- Created: 6/14/2024
- Company: Bottle Institute @ National Renewable Energy Lab, Bioeneergy Science and Technology
- License: MIT

Using ESM as a zero shot evaluator.

ESM has a few methods for which to evaluate likelihoods, see the paper: Meier, J. et al. Language models enable zero-shot prediction of the effects of mutations on protein function. Preprint at https://doi.org/10.1101/2021.07.09.450648 (2021).

The paper explored the following methods: 1. Masked Marginal Likelihood (masked_marginal) (Not yet implemented)

> Pass the wild type sequence L times, where L is the length of the sequence. Compute the likelihood of each AA at each position. Compare mutant vs wildtype AA at each position.

2. **Mutant Marginal Likelihood (mutant_marginal) (Not yet implemented)**
   Pass each variant sequence. N forward passes, where N is the count of variants. Compute the likelihood of mutated vs wildtype AA on each variant.
3. **Wildtype Marginal Likelihood (wildtype_marginal)**
   Pass the wild type sequence. 1 forward pass, regardless of count of variants Compute the likelihood of mutated vs wildtype AA.
4. **Psuedo-Likelihood (pseudo_likelihood) (Not implmented)**
   No plans to implement, proved poor performance in the paper.

Since ESM is a transformer, it can output position specific scores. Recall that such a model must adhere to the following rules: Inherits from PositionSpecificMixin, which enforces that *positions* is a parameter. We can use those positions to extract likelihoods at specific positions. If *positions* is None, we will return all positions.

There is a lot of here. Let's lay out a logic table to determine how to be most efficient here.

WT | Fixed Length | Positions passed | Pool | Method | N passes | Description

|----|——————|-----------------|——–|--------|—–| —- | Y | Y | N | Y | masked | M unique mutated positions in whole set, < L | Traditional masked marginal as described in the paper. Take WT, mask each mutated position, compare to WT, pool | Y | Y | N | N | masked | L | Can no longer only mask mutated positions since we are not pooling. Must mask all

positions. This is L forward passes. Return comparison of mut to wt for each position individually. Many will be zero if they are not mutated anywhere. | Y | Y | Y | Y | masked | Positions passed | Mask each position, compare to WT, pool | Y | Y | Y | N | masked | Positions passed | Mask each position, compare to WT, no pooling output positions | Y | Y | N | Y | wild_type | 1 | Traditional wild type marginal as described in the paper. Take WT and pass. Compare mutant likelihood to WT and pool only the mutated positions | Y | Y | N | N | wild_type | 1 | Take WT and pass. Compare mutant likelihood to WT on WT probability vector. Many positions will be zero since they are unmutated | Y | Y | Y | Y | wild_type | 1 | Take WT and pass. Compare mutant likelihood to WT on WT probability vector for only chosen positions. Pool. | Y | Y | Y | N | wild_type | 1 | Take WT and pass. Compare mutant likelihood to WT on WT probability vector for only chosen positions. No pooling. | Y | Y | N | Y | mutant | N | Traditional mutant marginal as described in the paper. Take each mutant and pass. Compare mutant likelihood to WT for only mutate positions on the mutant probability vector. Pool. | Y | Y | N | N | mutant | N | Take each mutant and pass. Compare mutant likelihood to WT for all positions on the mutant probability vector many will be zero. No pooling. | Y | Y | Y | Y | mutant | N | Take each mutant and pass. Compare mutant likelihood to WT on the mutant vector for positions specified. | Y | Y | Y | N | mutant | N | Take each mutant and pass. Compare mutant likelihood to WT on the mutant vector for positions specified. No pooling. | N | Y | N | Y | masked | L*N | Mask each position of each mutant, check probability of true AA at each position. Pool. | N | Y | N | N | masked | L*N | Mask each position of each mutant, check probability of true AA at each position. No pooling. | N | Y | Y | Y | masked | N * positions passed | Mask mutants on each position passed, check probability of true AA at each position. Pool. | N | Y | Y | N | masked | N * positions passed | Mask mutants on each position passed, check probability of true AA at each position. No pooling. | N | Any | Any | Any | wild_type | 0 | Not avialable. No wild type to compare to | N | Y | N | Y | mutant | N | Pass each mutant, check probability of true AA at each position. Pool. | N | Y | N | N | mutant | N | Pass each mutant, check probability of true AA at each position. No pooling. | N | Y | Y | Y | mutant | N | Pass each mutant, check probability of true AA at only passed positions. Pool. | N | Y | Y | N | mutant | N | Pass each mutant, check probability of true AA at only passed positions. No pooling. | N | N | N | Y | masked | ~L*N | Mask each position of each mutant, check probability of true AA at each position. Pool. | N | N | N | N | masked | 0 | Not available. Not pooling results in variabel length outputs. | N | N | Y | Y | masked | 0 | Not available. Cannot specify positions with variable length sequences. | N | N | Y | N | masked | 0 | Not available. Cannot specify positions with variable length sequences. | N | N | N | Y | mutant | N | Pass each mutant, check probability of true AA at each position. Pool. | N | N | N | N | mutant | 0 | Not available. Not pooling results in variabel length outputs. | N | N | Y | Y | mutant | 0 | Not available. Cannot specify positions with variable length sequences. | N | N | Y | N | mutant | 0 | Not available. Cannot specify positions with variable length sequences. | Y | N | N | Y | masked | ~L*(N+1) | Mask each position of each mutant, check probability of true AA at each position. Pool. Repeat for WT and noramlize. | Y | N | N | N | masked | 0 | Not available. Not pooling results in variabel length outputs. | Y | N | Y | Y | masked | 0 | Not available. Cannot specify positions with variable length sequences. | Y | N | Y | N | masked | 0 | Not available. Cannot specify positions with variable length sequences. | Y | N | N | Y | wild_type | 0 | Not available. Wild type not same length as mutants, so you cannit look at mutant likelihood from wt pass. | Y | N | N | N | wild_type | 0 | Not available. Wild type not same length as mutants, so you cannit look at mutant likelihood from wt pass. | Y | N | Y | Y | wild_type | 0 | Not available. Wild type not same length as mutants, so you cannit look at mutant likelihood from wt pass. | Y | N | Y | N | wild_type | 0 | Not available. Wild type not same length as mutants, so you cannit look at mutant likelihood from wt pass. | Y | N | N | Y | mutant | N+1 | Pass each mutant, check probability of true AA at each position on its own probability vector. Pool. Normalize by WT value | Y | N | N | N | mutant | 0 | Not available. Not pooling results in variabel length outputs. | Y | N | Y | Y | mutant | 0 | Not available. Cannot specify positions with variable length sequences. | Y | N | Y | N | mutant | 0 | Not available. Cannot specify positions with variable length sequences.

Conclusions:

1. If Variable length sequences, must pool. Cannot pass positions. wild_type marginal not available
2. If no wild type is given, only mutant or masked marginal is available.
3. Masked marginal removed for the case where wt is not given or sequences are variable length. For these cases, masks will have to be applied to all sequences not just the WT, vastly increasing cost.

Oh boy.

class aide_predict.bespoke_models.predictors.esm2.**ESM2LikelihoodWrapper**(*metadata_folder: str | None = None*, *model_checkpoint: str = 'esm2_t6_8M_UR50D'*, *marginal_method: MarginalMethod = 'mutant_marginal'*, *positions: list | None = None*, *pool: bool = True*, *flatten: bool = True*, *wt: str | None = None*, *batch_size: int = 2*, *device: str = 'cpu'*, *use_cache: bool = True*)

Bases: `LikelihoodTransformerBase`

property accepts_lower_case: bool

Whether the model can accept lower case sequences.

property can_handle_aligned_sequences: bool

Whether the model can handle aligned sequences (with gaps) at predict time.

property can_regress: bool

Whether the model can perform regression.

check_metadata() → None

Ensures that everything this model class needs is in the metadata folder.

property expects_no_fit: bool

Whether the model expects no fit.

fit(*X: ProteinSequences | List[str] | None = None*, *y: ndarray | None = None*, *force: bool = False*) → *ProteinModelWrapper*

Fit the model with pre-processing and post-processing from mixins.

Parameters

- **X** (*Union[ProteinSequences, List[str]]*) – Input sequences.
- **y** (*Optional[np.ndarray]*) – Target values.
- **force** (*bool*) – Whether to force refitting if already fitted.

Returns

The fitted model.

Return type

ProteinModelWrapper

fit_transform(*X*, *y=None*, ***fit_params*)

Fit to data, then transform it.

Fits transformer to *X* and *y* with optional parameters *fit_params* and returns a transformed version of *X*.

Parameters

- **X** (*array-like of shape (n_samples, n_features)*) – Input samples.

- **y** (*array-like of shape (n_samples,) or (n_samples, n_outputs), default=None*) – Target values (None for unsupervised transformations).
- ****fit_params** (*dict*) – Additional fit parameters.

Returns
X_new – Transformed array.

Return type
ndarray array of shape (n_samples, n_features_new)

`get_feature_names_out`(*input_features: List[str] | None = None*) → List[str]

Get output feature names for transformation.

Parameters
input_features (*Optional[List[str]]*) – Input feature names (not used in this method).

Returns
Output feature names.

Return type
List[str]

Raises
`ValueError` – If the model hasn't been fitted or if feature names can't be generated.

`get_fitted_attributes`() → List[str]

Get a list of attributes that are set during fitting.

`get_metadata_routing`()

Get metadata routing of this object.

Please check User Guide on how the routing mechanism works.

Returns
routing – A `MetadataRequest` encapsulating routing information.

Return type
MetadataRequest

`get_params`(*deep: bool = True*) → Dict[str, Any]

Get parameters for this estimator.

Parameters
deep (*bool*) – If True, will return the parameters for this estimator and contained subobjects.

Returns
Parameter names mapped to their values.

Return type
Dict[str, Any]

`property metadata_folder`

`partial_fit`(*X:* ProteinSequences *| List[str], y: ndarray | None = None*) → *ProteinModelWrapper*

Partially fit the model to the given sequences.

This method can be called multiple times to incrementally fit the model.

Parameters

- **X** (*Union[ProteinSequences, List[str]]*) – The input sequences to partially fit the model on.

- **y** (*Optional[np.ndarray]*) – The target values, if applicable.

Returns
The partially fitted model.

Return type
ProteinModelWrapper

property per_position_capable: bool

Whether the model can output per position scores.

predict(*X: ProteinSequences | List[str]*) → ndarray

Predict the sequences.

Parameters
X (*Union[ProteinSequences, List[str]]*) – Input sequences.

Returns
Predicted values.

Return type
np.ndarray

Raises
ValueError – If the model is not capable of regression.

property requires_fixed_length: bool

Whether the model requires fixed length input.

property requires_msa_for_fit: bool

Whether the model requires an MSA for fitting.

property requires_msa_per_sequence: bool

Whether the model requires an MSA for each sequence during transform.

property requires_structure: bool

Whether the model requires structure information.

property requires_wt_during_inference: bool

Whether the model requires wild type during inference.

property requires_wt_msa: bool

Whether the model requires a wild type MSA for fitting.

property requires_wt_to_function: bool

Whether the model requires the wild type sequence to function.

score(*X, y, sample_weight=None*)

Return the Spearman correlation

set_fit_request(**, force: bool | None | str = '$UNCHANGED$'*) → *ESM2LikelihoodWrapper*

Request metadata passed to the `fit` method.

Note that this method is only relevant if `enable_metadata_routing=True` (see `sklearn.set_config()`). Please see User Guide on how the routing mechanism works.

The options for each parameter are:

- `True`: metadata is requested, and passed to `fit` if provided. The request is ignored if metadata is not provided.
- `False`: metadata is not requested and the meta-estimator will not pass it to `fit`.

- `None`: metadata is not requested, and the meta-estimator will raise an error if the user provides it.
- `str`: metadata should be passed to the meta-estimator with this given alias instead of the original name.

The default (`sklearn.utils.metadata_routing.UNCHANGED`) retains the existing request. This allows you to change the request for some parameters and not others.

Added in version 1.3.

Note: This method is only relevant if this estimator is used as a sub-estimator of a meta-estimator, e.g. used inside a `Pipeline`. Otherwise it has no effect.

Parameters
force (*str, True, False, or None, default=sklearn.utils.metadata_routing.UNCHANGED*) – Metadata routing for `force` parameter in `fit`.

Returns
self – The updated object.

Return type
object

`set_output`(**, transform=None*)

Set output container.

See sphx_glr_auto_examples_miscellaneous_plot_set_output.py for an example on how to use the API.

Parameters
transform (*{"default", "pandas", "polars"}, default=None*) – Configure output of *transform* and *fit_transform*.

- *"default"*: Default output format of a transformer
- *"pandas"*: DataFrame output
- *"polars"*: Polars output
- *None*: Transform configuration is unchanged

Added in version 1.4: *"polars"* option was added.

Returns
self – Estimator instance.

Return type
estimator instance

`set_params`(***params: Any*) → *ProteinModelWrapper*

Set the parameters of this estimator.

Parameters
****params** – Estimator parameters.

Returns
Estimator instance.

Return type
ProteinModelWrapper

set_score_request(*, *sample_weight: bool | None | str = '$UNCHANGED$'*) → *ESM2LikelihoodWrapper*

Request metadata passed to the `score` method.

Note that this method is only relevant if `enable_metadata_routing=True` (see `sklearn.set_config()`). Please see User Guide on how the routing mechanism works.

The options for each parameter are:

- `True`: metadata is requested, and passed to `score` if provided. The request is ignored if metadata is not provided.
- `False`: metadata is not requested and the meta-estimator will not pass it to `score`.
- `None`: metadata is not requested, and the meta-estimator will raise an error if the user provides it.
- `str`: metadata should be passed to the meta-estimator with this given alias instead of the original name.

The default (`sklearn.utils.metadata_routing.UNCHANGED`) retains the existing request. This allows you to change the request for some parameters and not others.

Added in version 1.3.

Note: This method is only relevant if this estimator is used as a sub-estimator of a meta-estimator, e.g. used inside a `Pipeline`. Otherwise it has no effect.

Parameters
sample_weight (*str, True, False, or None, default=sklearn.utils.metadata_routing.UNCHANGED*) – Metadata routing for `sample_weight` parameter in `score`.

Returns
self – The updated object.

Return type
object

property should_refit_on_sequences: bool

Whether the model should refit on new sequences when given.

transform(*X: ProteinSequences | List[str]*) → ndarray

Transform sequences with pre-processing and post-processing from mixins.

Parameters
X (*Union[ProteinSequences, List[str]]*) – Input sequences.

Returns
Transformed sequences.

Return type
np.ndarray

property wt

aide_predict.bespoke_models.predictors.eve module

- Author: Evan Komp
- Created: 10/28/2024
- Company: National Renewable Energy Lab, Bioeneergy Science and Technology
- License: MIT

Wrapper for EVE (Evolutionary Variational Autoencoder) model. Please see original paper and implementation: https://github.com/OATML/EVE

class aide_predict.bespoke_models.predictors.eve.**EVEWrapper**(*metadata_folder: str | None = None, wt: str | ProteinSequence | None = None, theta: float = 0.2, encoder_hidden_layers: List[int] = [2000, 1000, 300], encoder_z_dim: int = 50, encoder_convolve_input: bool = False, encoder_convolution_input_depth: int = 40, encoder_nonlinear_activation: str = 'relu', encoder_dropout_proba: float = 0.0, decoder_hidden_layers: List[int] = [300, 1000, 2000], decoder_z_dim: int = 50, decoder_bayesian: bool = True, decoder_first_nonlinearity: str = 'relu', decoder_last_nonlinearity: str = 'relu', decoder_dropout_proba: float = 0.1, decoder_convolve_output: bool = True, decoder_convolution_output_depth: int = 40, decoder_temperature_scaler: bool = True, decoder_sparsity: bool = False, decoder_num_tiles_sparsity: int = 0, decoder_logit_sparsity_p: float = 0.0, training_steps: int = 400000, learning_rate: float = 0.0001, training_batch_size: int = 256, annealing_warm_up: int = 0, kl_latent_scale: float = 1.0, kl_global_params_scale: float = 1.0, l2_regularization: float = 0.0, use_lr_scheduler: bool = False, use_validation_set: bool = False, validation_set_pct: float = 0.2, validation_freq: int = 1000, log_training_info: bool = True, log_training_freq: int = 1000, save_model_freq: int = 500000, inference_batch_size: int = 256, num_samples: int = 10*)

Bases: `RequiresWTToFunctionMixin`, `RequiresFixedLengthMixin`, `RequiresWTDuringInferenceMixin`, `RequiresMSAForFitMixin`, `RequiresWTMSAMixin`, `AcceptsLowerCaseMixin`, `CanRegressMixin`, `ProteinModelWrapper`

Wrapper for EVE (Evolutionary Variational Autoencoder) model.

This wrapper provides an interface to train and use EVE models within the AIDE framework. EVE is run in a separate conda environment specified by EVE_CONDA_ENV environment variable. The EVE repository location must be specified in EVE_REPO environment variable.

NOTE: SHould this refit on sequences?

Variables

_available (MessageBool) – Indicates whether EVE is available based on environment setup.

__init__(*metadata_folder: str | None = None*, *wt: str | ProteinSequence | None = None*, *theta: float = 0.2*, *encoder_hidden_layers: List[int] = [2000, 1000, 300]*, *encoder_z_dim: int = 50*, *encoder_convolve_input: bool = False*, *encoder_convolution_input_depth: int = 40*, *encoder_nonlinear_activation: str = 'relu'*, *encoder_dropout_proba: float = 0.0*, *decoder_hidden_layers: List[int] = [300, 1000, 2000]*, *decoder_z_dim: int = 50*, *decoder_bayesian: bool = True*, *decoder_first_nonlinearity: str = 'relu'*, *decoder_last_nonlinearity: str = 'relu'*, *decoder_dropout_proba: float = 0.1*, *decoder_convolve_output: bool = True*, *decoder_convolution_output_depth: int = 40*, *decoder_temperature_scaler: bool = True*, *decoder_sparsity: bool = False*, *decoder_num_tiles_sparsity: int = 0*, *decoder_logit_sparsity_p: float = 0.0*, *training_steps: int = 400000*, *learning_rate: float = 0.0001*, *training_batch_size: int = 256*, *annealing_warm_up: int = 0*, *kl_latent_scale: float = 1.0*, *kl_global_params_scale: float = 1.0*, *l2_regularization: float = 0.0*, *use_lr_scheduler: bool = False*, *use_validation_set: bool = False*, *validation_set_pct: float = 0.2*, *validation_freq: int = 1000*, *log_training_info: bool = True*, *log_training_freq: int = 1000*, *save_model_freq: int = 500000*, *inference_batch_size: int = 256*, *num_samples: int = 10*)

Initialize the EVE wrapper with all configurable parameters exposed.

Parameters

- **metadata_folder** (*str*) – Folder to store intermediate files and model artifacts.
- **wt** (*Optional[Union[str, ProteinSequence]]*) – Wild-type sequence.
- **Processing** (*# MSA*)
- **theta** (*float*) – Parameter for MSA sequence reweighting.
- **Parameters** (*# Inference*)
- **encoder_hidden_layers** (*List[int]*) – Sizes of hidden layers in encoder.
- **encoder_z_dim** (*int*) – Dimensionality of latent space.
- **encoder_convolve_input** (*bool*) – Whether to apply convolution to input.
- **encoder_convolution_input_depth** (*int*) – Depth of input convolution.
- **encoder_nonlinear_activation** (*str*) – Activation function for encoder.
- **encoder_dropout_proba** (*float*) – Dropout probability in encoder.
- **Parameters**
- **decoder_hidden_layers** (*List[int]*) – Sizes of hidden layers in decoder.
- **decoder_z_dim** (*int*) – Dimensionality of latent space (should match encoder).
- **decoder_bayesian** (*bool*) – Whether to use Bayesian decoder.
- **decoder_first_nonlinearity** (*str*) – Activation for first layer.
- **decoder_last_nonlinearity** (*str*) – Activation for last layer.
- **decoder_dropout_proba** (*float*) – Dropout probability in decoder.

- **decoder_convolve_output** (*bool*) – Whether to apply convolution to output.
- **decoder_convolution_output_depth** (*int*) – Depth of output convolution.
- **decoder_temperature_scaler** (*bool*) – Whether to use temperature scaling.
- **decoder_sparsity** (*bool*) – Whether to enforce sparsity.
- **decoder_num_tiles_sparsity** (*int*) – Number of tiles for sparsity.
- **decoder_logit_sparsity_p** (*float*) – Sparsity parameter.
- **Parameters**
- **training_steps** (*int*) – Number of training steps.
- **learning_rate** (*float*) – Learning rate for optimization.
- **training_batch_size** (*int*) – Batch size during training.
- **annealing_warm_up** (*int*) – Steps for KL annealing warmup.
- **kl_latent_scale** (*float*) – Scale for latent KL term.
- **kl_global_params_scale** (*float*) – Scale for global parameters KL term.
- **l2_regularization** (*float*) – L2 regularization strength.
- **use_lr_scheduler** (*bool*) – Whether to use learning rate scheduler.
- **use_validation_set** (*bool*) – Whether to use validation set.
- **validation_set_pct** (*float*) – Percentage of data for validation.
- **validation_freq** (*int*) – Frequency of validation.
- **log_training_info** (*bool*) – Whether to log training information.
- **log_training_freq** (*int*) – Frequency of logging.
- **save_model_freq** (*int*) – Frequency of model saving.
- **Parameters**
- **inference_batch_size** (*int*) – Batch size for computing evolutionary indices.
- **num_samples** (*int*) – Number of samples for approximating delta ELBO.

property accepts_lower_case: bool

Whether the model can accept lower case sequences.

property can_handle_aligned_sequences: bool

Whether the model can handle aligned sequences (with gaps) at predict time.

property can_regress: bool

Whether the model can perform regression.

check_metadata() → None

Ensures that everything this model class needs is in the metadata folder.

property expects_no_fit: bool

Whether the model expects no fit.

fit(*X: ProteinSequences | List[str] | None = None, y: ndarray | None = None, force: bool = False*) → *ProteinModelWrapper*

Fit the model with pre-processing and post-processing from mixins.

Parameters

- **X** (*Union[ProteinSequences, List[str]]*) – Input sequences.
- **y** (*Optional[np.ndarray]*) – Target values.
- **force** (*bool*) – Whether to force refitting if already fitted.

Returns
The fitted model.

Return type
ProteinModelWrapper

fit_transform(*X*, *y=None*, ***fit_params*)

Fit to data, then transform it.

Fits transformer to *X* and *y* with optional parameters *fit_params* and returns a transformed version of *X*.

Parameters

- **X** (*array-like of shape (n_samples, n_features)*) – Input samples.
- **y** (*array-like of shape (n_samples,) or (n_samples, n_outputs), default=None*) – Target values (None for unsupervised transformations).
- ****fit_params** (*dict*) – Additional fit parameters.

Returns
X_new – Transformed array.

Return type
ndarray array of shape (n_samples, n_features_new)

get_feature_names_out(*input_features: List[str] | None = None*) → List[str]

Get output feature names.

get_metadata_routing()

Get metadata routing of this object.

Please check User Guide on how the routing mechanism works.

Returns
routing – A `MetadataRequest` encapsulating routing information.

Return type
MetadataRequest

get_params(*deep: bool = True*) → Dict[str, Any]

Get parameters for this estimator.

Parameters
deep (*bool*) – If True, will return the parameters for this estimator and contained subobjects that are estimators.

Returns
Parameter names mapped to their values.

Return type
Dict[str, Any]

property metadata_folder

partial_fit(*X: ProteinSequences | List[str]*, *y: ndarray | None = None*) → *ProteinModelWrapper*

Partially fit the model to the given sequences.

This method can be called multiple times to incrementally fit the model.

Parameters

- **X** (*Union[ProteinSequences, List[str]]*) – The input sequences to partially fit the model on.
- **y** (*Optional[np.ndarray]*) – The target values, if applicable.

Returns

The partially fitted model.

Return type

ProteinModelWrapper

property per_position_capable: bool

Whether the model can output per position scores.

predict(*X: ProteinSequences | List[str]*) → ndarray

Predict the sequences.

Parameters

X (*Union[ProteinSequences, List[str]]*) – Input sequences.

Returns

Predicted values.

Return type

np.ndarray

Raises

`ValueError` – If the model is not capable of regression.

property requires_fixed_length: bool

Whether the model requires fixed length input.

property requires_msa_for_fit: bool

Whether the model requires an MSA for fitting.

property requires_msa_per_sequence: bool

Whether the model requires an MSA for each sequence during transform.

property requires_structure: bool

Whether the model requires structure information.

property requires_wt_during_inference: bool

Whether the model requires wild type during inference.

property requires_wt_msa: bool

Whether the model requires a wild type MSA for fitting.

property requires_wt_to_function: bool

Whether the model requires the wild type sequence to function.

score(*X*, *y*, *sample_weight=None*)

Return the Spearman correlation

set_fit_request(*, *force: bool | None | str = '$UNCHANGED$'*) → *EVEWrapper*

Request metadata passed to the `fit` method.

Note that this method is only relevant if `enable_metadata_routing=True` (see `sklearn.set_config()`). Please see User Guide on how the routing mechanism works.

The options for each parameter are:

- `True`: metadata is requested, and passed to `fit` if provided. The request is ignored if metadata is not provided.
- `False`: metadata is not requested and the meta-estimator will not pass it to `fit`.
- `None`: metadata is not requested, and the meta-estimator will raise an error if the user provides it.
- `str`: metadata should be passed to the meta-estimator with this given alias instead of the original name.

The default (`sklearn.utils.metadata_routing.UNCHANGED`) retains the existing request. This allows you to change the request for some parameters and not others.

Added in version 1.3.

Note: This method is only relevant if this estimator is used as a sub-estimator of a meta-estimator, e.g. used inside a `Pipeline`. Otherwise it has no effect.

Parameters

force (*str, True, False, or None, default=sklearn.utils.metadata_routing.UNCHANGED*) – Metadata routing for `force` parameter in `fit`.

Returns

self – The updated object.

Return type

object

set_output(*, *transform=None*)

Set output container.

See sphx_glr_auto_examples_miscellaneous_plot_set_output.py for an example on how to use the API.

Parameters

transform (*{"default", "pandas", "polars"}, default=None*) – Configure output of *transform* and *fit_transform*.

- *"default"*: Default output format of a transformer
- *"pandas"*: DataFrame output
- *"polars"*: Polars output
- *None*: Transform configuration is unchanged

Added in version 1.4: *"polars"* option was added.

Returns

self – Estimator instance.

Return type

estimator instance

set_params(***params: Any*) → *EVEWrapper*

Set the parameters of this estimator.

Parameters
****params** – Estimator parameters.

Returns
Return self to enable chaining.

Return type
EVEWrapper

set_score_request(*, *sample_weight: bool | None | str = '$UNCHANGED$'*) → *EVEWrapper*

Request metadata passed to the `score` method.

Note that this method is only relevant if `enable_metadata_routing=True` (see `sklearn.set_config()`). Please see User Guide on how the routing mechanism works.

The options for each parameter are:

- `True`: metadata is requested, and passed to `score` if provided. The request is ignored if metadata is not provided.
- `False`: metadata is not requested and the meta-estimator will not pass it to `score`.
- `None`: metadata is not requested, and the meta-estimator will raise an error if the user provides it.
- `str`: metadata should be passed to the meta-estimator with this given alias instead of the original name.

The default (`sklearn.utils.metadata_routing.UNCHANGED`) retains the existing request. This allows you to change the request for some parameters and not others.

Added in version 1.3.

Note: This method is only relevant if this estimator is used as a sub-estimator of a meta-estimator, e.g. used inside a `Pipeline`. Otherwise it has no effect.

Parameters
sample_weight (*str, True, False, or None, default=sklearn.utils.metadata_routing.UNCHANGED*) – Metadata routing for `sample_weight` parameter in `score`.

Returns
self – The updated object.

Return type
object

property should_refit_on_sequences: bool

Whether the model should refit on new sequences when given.

transform(*X:* ProteinSequences | *List[str]*) → ndarray

Transform sequences with pre-processing and post-processing from mixins.

Parameters
X (*Union[ProteinSequences, List[str]]*) – Input sequences.

Returns
Transformed sequences.

Return type
np.ndarray

property wt

aide_predict.bespoke_models.predictors.evmutation module

- Author: Evan Komp
- Created: 7/12/2024
- Company: National Renewable Energy Lab, Bioeneergy Science and Technology
- License: MIT

Wrapper around EVmutation model from the EVCouplings repositorty: https://github.com/debbiemarkslab/EVcouplings/tree/develop

Hopf T. A., Green A. G., Schubert B., et al. The EVcouplings Python framework for coevolutionary sequence analysis. Bioinformatics 35, 1582–1584 (2019)

class aide_predict.bespoke_models.predictors.evmutation.**EVMutationWrapper**(*metadata_folder: str | None = None, wt: str | ProteinSequence | None = None, protocol: str = 'standard', theta: float = 0.8, iterations: int = 100, lambda_h: float = 0.01, lambda_J: float = 0.01, lambda_group: float | None = None, min_sequence_distance: int = 6, cpu: int = 1, use_cache: bool = False*)

Bases: *CacheMixin*, *RequiresWTToFunctionMixin*, *RequiresFixedLengthMixin*, *RequiresMSAForFitMixin*, *RequiresWTMSAMixin*, *CanRegressMixin*, *AcceptsLowerCaseMixin*, *ProteinModelWrapper*

A wrapper for EVCouplings that implements the ProteinModelWrapper interface.

__init__(*metadata_folder: str | None = None, wt: str | ProteinSequence | None = None, protocol: str = 'standard', theta: float = 0.8, iterations: int = 100, lambda_h: float = 0.01, lambda_J: float = 0.01, lambda_group: float | None = None, min_sequence_distance: int = 6, cpu: int = 1, use_cache: bool = False*)

Initialize the EVCouplingsWrapper.

Parameters

- **metadata_folder** (*str*) – Folder to store metadata and intermediate files.
- **wt** (*Optional[Union[str, ProteinSequence]]*) – Wild-type sequence.
- **protocol** (*str*) – EVCouplings protocol to use ("standard", "complex", or "mean_field").

- **theta** (*float*) – Sequence clustering threshold.
- **iterations** (*int*) – Number of iterations for inference.
- **lambda_h** (*float*) – Regularization strength on fields.
- **lambda_J** (*float*) – Regularization strength on couplings.
- **lambda_group** (*float*) – Group regularization strength.
- **cpu** (*int*) – Number of CPUs to use.

property accepts_lower_case: bool

Whether the model can accept lower case sequences.

property can_handle_aligned_sequences: bool

Whether the model can handle aligned sequences (with gaps) at predict time.

property can_regress: bool

Whether the model can perform regression.

check_metadata() → None

Ensures that everything this model class needs is in the metadata folder.

property expects_no_fit: bool

Whether the model expects no fit.

fit(*X: ProteinSequences | List[str] | None = None*, *y: ndarray | None = None*, *force: bool = False*) → *ProteinModelWrapper*

Fit the model with pre-processing and post-processing from mixins.

Parameters

- **X** (*Union[ProteinSequences, List[str]]*) – Input sequences.
- **y** (*Optional[np.ndarray]*) – Target values.
- **force** (*bool*) – Whether to force refitting if already fitted.

Returns

The fitted model.

Return type

ProteinModelWrapper

fit_transform(*X*, *y=None*, ***fit_params*)

Fit to data, then transform it.

Fits transformer to *X* and *y* with optional parameters *fit_params* and returns a transformed version of *X*.

Parameters

- **X** (*array-like of shape (n_samples, n_features)*) – Input samples.
- **y** (*array-like of shape (n_samples,) or (n_samples, n_outputs), default=None*) – Target values (None for unsupervised transformations).
- ****fit_params** (*dict*) – Additional fit parameters.

Returns

X_new – Transformed array.

Return type

ndarray array of shape (n_samples, n_features_new)

get_feature_names_out(*input_features: List[str] | None = None*) → List[str]

Get output feature names for transformation.

Parameters
input_features (*Optional[List[str]]*) – Ignored. Present for API consistency.

Returns
A list containing a single feature name.

Return type
List[str]

get_fitted_attributes() → List[str]

Get a list of attributes that are set during fitting.

get_metadata_routing()

Get metadata routing of this object.

Please check User Guide on how the routing mechanism works.

Returns
routing – A `MetadataRequest` encapsulating routing information.

Return type
MetadataRequest

get_params(*deep: bool = True*) → Dict[str, Any]

Get parameters for this estimator.

Parameters
deep (*bool*) – If True, will return the parameters for this estimator and contained subobjects.

Returns
Parameter names mapped to their values.

Return type
Dict[str, Any]

property metadata_folder

partial_fit(*X:* ProteinSequences *| List[str], y: ndarray | None = None*) → *ProteinModelWrapper*

Partially fit the model to the given sequences.

This method can be called multiple times to incrementally fit the model.

Parameters

- **X** (*Union[ProteinSequences, List[str]]*) – The input sequences to partially fit the model on.
- **y** (*Optional[np.ndarray]*) – The target values, if applicable.

Returns
The partially fitted model.

Return type
ProteinModelWrapper

property per_position_capable: bool

Whether the model can output per position scores.

predict(*X: ProteinSequences | List[str]*) → ndarray

Predict the sequences.

Parameters
X (*Union[*`ProteinSequences`*, List[str]]*) – Input sequences.

Returns
Predicted values.

Return type
np.ndarray

Raises
ValueError – If the model is not capable of regression.

property requires_fixed_length: bool

Whether the model requires fixed length input.

property requires_msa_for_fit: bool

Whether the model requires an MSA for fitting.

property requires_msa_per_sequence: bool

Whether the model requires an MSA for each sequence during transform.

property requires_structure: bool

Whether the model requires structure information.

property requires_wt_during_inference: bool

Whether the model requires wild type during inference.

property requires_wt_msa: bool

Whether the model requires a wild type MSA for fitting.

property requires_wt_to_function: bool

Whether the model requires the wild type sequence to function.

score(*X*, *y*, *sample_weight=None*)

Return the Spearman correlation

set_fit_request(*, *force: bool | None | str = '$UNCHANGED$'*) → *EVMutationWrapper*

Request metadata passed to the `fit` method.

Note that this method is only relevant if `enable_metadata_routing=True` (see `sklearn.set_config()`). Please see User Guide on how the routing mechanism works.

The options for each parameter are:

- `True`: metadata is requested, and passed to `fit` if provided. The request is ignored if metadata is not provided.
- `False`: metadata is not requested and the meta-estimator will not pass it to `fit`.
- `None`: metadata is not requested, and the meta-estimator will raise an error if the user provides it.
- `str`: metadata should be passed to the meta-estimator with this given alias instead of the original name.

The default (`sklearn.utils.metadata_routing.UNCHANGED`) retains the existing request. This allows you to change the request for some parameters and not others.

Added in version 1.3.

Note: This method is only relevant if this estimator is used as a sub-estimator of a meta-estimator, e.g. used inside a `Pipeline`. Otherwise it has no effect.

Parameters
`force` (*str, True, False, or None, default=sklearn.utils.metadata_routing.UNCHANGED*) – Metadata routing for `force` parameter in `fit`.

Returns
self – The updated object.

Return type
object

`set_output`(*, *transform=None*)

Set output container.

See sphx_glr_auto_examples_miscellaneous_plot_set_output.py for an example on how to use the API.

Parameters
`transform` (*{"default", "pandas", "polars"}, default=None*) – Configure output of *transform* and *fit_transform*.

- *"default"*: Default output format of a transformer
- *"pandas"*: DataFrame output
- *"polars"*: Polars output
- *None*: Transform configuration is unchanged

Added in version 1.4: *"polars"* option was added.

Returns
self – Estimator instance.

Return type
estimator instance

`set_params`(***params: Any*) → *ProteinModelWrapper*

Set the parameters of this estimator.

Parameters
`**params` – Estimator parameters.

Returns
Estimator instance.

Return type
ProteinModelWrapper

`set_score_request`(*, *sample_weight: bool | None | str = '$UNCHANGED$'*) → *EVMutationWrapper*

Request metadata passed to the `score` method.

Note that this method is only relevant if `enable_metadata_routing=True` (see `sklearn.set_config()`). Please see User Guide on how the routing mechanism works.

The options for each parameter are:

- `True`: metadata is requested, and passed to `score` if provided. The request is ignored if metadata is not provided.

- `False`: metadata is not requested and the meta-estimator will not pass it to `score`.
- `None`: metadata is not requested, and the meta-estimator will raise an error if the user provides it.
- `str`: metadata should be passed to the meta-estimator with this given alias instead of the original name.

The default (`sklearn.utils.metadata_routing.UNCHANGED`) retains the existing request. This allows you to change the request for some parameters and not others.

Added in version 1.3.

Note: This method is only relevant if this estimator is used as a sub-estimator of a meta-estimator, e.g. used inside a `Pipeline`. Otherwise it has no effect.

Parameters
`sample_weight` (*str, True, False, or None, default=sklearn.utils.metadata_routing.UNCHANGED*) – Metadata routing for `sample_weight` parameter in `score`.

Returns
self – The updated object.

Return type
object

property should_refit_on_sequences: bool

Whether the model should refit on new sequences when given.

transform(*X: ProteinSequences | List[str]*) → ndarray

Transform sequences with pre-processing and post-processing from mixins.

Parameters
X (*Union[ProteinSequences, List[str]]*) – Input sequences.

Returns
Transformed sequences.

Return type
np.ndarray

property wt

aide_predict.bespoke_models.predictors.hmm module

- Author: Evan Komp
- Created: 6/11/2024
- Company: Bottle Institute @ National Renewable Energy Lab, Bioeneergy Science and Technology
- License: MIT

Wrapper of HMMs into an sklearn transformer for use in the AIDE pipeline. Uses HMMsearch against the HMM

Here are the docs for HMMSearch:

Usage: hmmsearch [options] <hmmfile> <seqdb>

Basic options:
-h : show brief help on version and usage

Options directing output:

**-o <f>**	: direct output to file <f>, not stdout
**-A <f>**	: save multiple alignment of all hits to file <f>
**--tblout <f>**	: save parseable table of per-sequence hits to file <f>
**--domtblout <f>**	: save parseable table of per-domain hits to file <f>

–pfamtblout <f> : save table of hits and domains to file, in Pfam format <f> –acc : prefer accessions over names in output –noali : don't output alignments, so output is smaller –notextw : unlimit ASCII text output line width –textw <n> : set max width of ASCII text output lines [120] (n>=120)

Options controlling reporting thresholds:

**-E <x>**	: report sequences <= this E-value threshold in output [10.0] (x>0)
**-T <x>**	: report sequences >= this score threshold in output

–domE <x> : report domains <= this E-value threshold in output [10.0] (x>0) –domT <x> : report domains >= this score cutoff in output

Options controlling inclusion (significance) thresholds:

**--incE <x>**	: consider sequences <= this E-value threshold as significant
**--incT <x>**	: consider sequences >= this score threshold as significant

–incdomE <x> : consider domains <= this E-value threshold as significant –incdomT <x> : consider domains >= this score threshold as significant

Options controlling model-specific thresholding:
–cut_ga : use profile's GA gathering cutoffs to set all thresholding –cut_nc : use profile's NC noise cutoffs to set all thresholding –cut_tc : use profile's TC trusted cutoffs to set all thresholding

Options controlling acceleration heuristics:

**--max**	: Turn all heuristic filters off (less speed, more power)

–F1 <x> : Stage 1 (MSV) threshold: promote hits w/ P <= F1 [0.02] –F2 <x> : Stage 2 (Vit) threshold: promote hits w/ P <= F2 [1e-3] –F3 <x> : Stage 3 (Fwd) threshold: promote hits w/ P <= F3 [1e-5] –nobias : turn off composition bias filter

Other expert options:

**--nonull2**	: turn off biased composition score corrections
**-Z <x>**	: set # of comparisons done, for E-value calculation
**--domZ <x>**	: set # of significant seqs, for domain E-value calculation
**--seed <n>**	: set RNG seed to <n> (if 0: one-time arbitrary seed) [42]

–tformat <s> : assert target <seqfile> is in format <s>: no autodetection –cpu <n> : number of parallel CPU workers to use for multithreads [2]

Some of these need to be user parameterizable, and some need to be fixed.

`class aide_predict.bespoke_models.predictors.hmm.`**`HMMWrapper`**`(`*threshold: float = 100*, *metadata_folder: str | None = None*, *wt: str |* ProteinSequence *| None = None*`)`

Bases: *CanRegressMixin*, *RequiresMSAForFitMixin*, *ProteinModelWrapper*

Wrapper for Hidden Markov Models (HMMs) using HMMsearch to score sequences.

This wrapper builds an HMM from an input alignment and uses HMMsearch to get scores for new sequences. Bit scores are used to compare to the HMM as opposed to E values. Tune the threshold parameter accordingly.

Variables

- **threshold** (*float*) – Threshold for HMMsearch.
- **metadata_folder** (*str*) – Folder to store metadata.
- **wt** (*Optional[ProteinSequence]*) – Wild-type sequence.

__init__(*threshold: float = 100*, *metadata_folder: str | None = None*, *wt: str* | ProteinSequence | *None = None*)

Initialize the HMMWrapper.

Parameters

- **threshold** (*float*) – Threshold for HMMsearch. Defaults to 100.
- **metadata_folder** (*Optional[str]*) – Folder to store metadata. Defaults to None.
- **wt** (*Optional[Union[str, 'ProteinSequence']]*) – Wild-type sequence. Defaults to None.

property accepts_lower_case: bool

Whether the model can accept lower case sequences.

property can_handle_aligned_sequences: bool

Whether the model can handle aligned sequences (with gaps) at predict time.

property can_regress: bool

Whether the model can perform regression.

check_metadata() → None

Ensures that everything this model class needs is in the metadata folder.

property expects_no_fit: bool

Whether the model expects no fit.

fit(*X:* ProteinSequences | *List[str] | None = None*, *y: ndarray | None = None*, *force: bool = False*) → *ProteinModelWrapper*

Fit the model with pre-processing and post-processing from mixins.

Parameters

- **X** (*Union[ProteinSequences, List[str]]*) – Input sequences.
- **y** (*Optional[np.ndarray]*) – Target values.
- **force** (*bool*) – Whether to force refitting if already fitted.

Returns

The fitted model.

Return type

ProteinModelWrapper

fit_transform(*X*, *y=None*, ***fit_params*)

Fit to data, then transform it.

Fits transformer to *X* and *y* with optional parameters *fit_params* and returns a transformed version of *X*.

Parameters

- **X** (*array-like of shape (n_samples, n_features)*) – Input samples.
- **y** (*array-like of shape (n_samples,) or (n_samples, n_outputs), default=None*) – Target values (None for unsupervised transformations).
- ****fit_params** (*dict*) – Additional fit parameters.

Returns

X_new – Transformed array.

Return type

ndarray array of shape (n_samples, n_features_new)

get_feature_names_out(*input_features: List[str] | None = None*) → List[str]

Get output feature names for transformation.

Parameters

input_features (*Optional[List[str]]*) – Input feature names.

Returns

Output feature names.

Return type

List[str]

get_metadata_routing()

Get metadata routing of this object.

Please check User Guide on how the routing mechanism works.

Returns

routing – A `MetadataRequest` encapsulating routing information.

Return type

MetadataRequest

get_params(*deep: bool = True*) → Dict[str, Any]

Get parameters for this estimator.

Parameters

deep (*bool*) – If True, will return the parameters for this estimator and contained subobjects.

Returns

Parameter names mapped to their values.

Return type

Dict[str, Any]

property metadata_folder

partial_fit(*X: ProteinSequences | List[str], y: ndarray | None = None*) → *ProteinModelWrapper*

Partially fit the model to the given sequences.

This method can be called multiple times to incrementally fit the model.

Parameters

- **X** (*Union[ProteinSequences, List[str]]*) – The input sequences to partially fit the model on.
- **y** (*Optional[np.ndarray]*) – The target values, if applicable.

Returns
The partially fitted model.

Return type
ProteinModelWrapper

property per_position_capable: bool

Whether the model can output per position scores.

predict(*X: ProteinSequences | List[str]*) → ndarray

Predict the sequences.

Parameters
X (*Union[ProteinSequences, List[str]]*) – Input sequences.

Returns
Predicted values.

Return type
np.ndarray

Raises
ValueError – If the model is not capable of regression.

property requires_fixed_length: bool

Whether the model requires fixed length input.

property requires_msa_for_fit: bool

Whether the model requires an MSA for fitting.

property requires_msa_per_sequence: bool

Whether the model requires an MSA for each sequence during transform.

property requires_structure: bool

Whether the model requires structure information.

property requires_wt_during_inference: bool

Whether the model requires wild type during inference.

property requires_wt_msa: bool

Whether the model requires a wild type MSA for fitting.

property requires_wt_to_function: bool

Whether the model requires the wild type sequence to function.

score(*X*, *y*, *sample_weight=None*)

Return the Spearman correlation

set_fit_request(*, *force: bool | None | str = '$UNCHANGED$'*) → *HMMWrapper*

Request metadata passed to the `fit` method.

Note that this method is only relevant if `enable_metadata_routing=True` (see `sklearn.set_config()`). Please see User Guide on how the routing mechanism works.

The options for each parameter are:

- `True`: metadata is requested, and passed to `fit` if provided. The request is ignored if metadata is not provided.
- `False`: metadata is not requested and the meta-estimator will not pass it to `fit`.
- `None`: metadata is not requested, and the meta-estimator will raise an error if the user provides it.
- `str`: metadata should be passed to the meta-estimator with this given alias instead of the original name.

The default (`sklearn.utils.metadata_routing.UNCHANGED`) retains the existing request. This allows you to change the request for some parameters and not others.

Added in version 1.3.

Note: This method is only relevant if this estimator is used as a sub-estimator of a meta-estimator, e.g. used inside a `Pipeline`. Otherwise it has no effect.

Parameters
force (*str, True, False, or None, default=sklearn.utils.metadata_routing.UNCHANGED*) – Metadata routing for `force` parameter in `fit`.

Returns
self – The updated object.

Return type
object

`set_output`(*, *transform=None*)

Set output container.

See sphx_glr_auto_examples_miscellaneous_plot_set_output.py for an example on how to use the API.

Parameters
transform (*{"default", "pandas", "polars"}, default=None*) – Configure output of *transform* and *fit_transform*.

- *"default"*: Default output format of a transformer
- *"pandas"*: DataFrame output
- *"polars"*: Polars output
- *None*: Transform configuration is unchanged

Added in version 1.4: *"polars"* option was added.

Returns
self – Estimator instance.

Return type
estimator instance

`set_params`(***params: Any*) → *ProteinModelWrapper*

Set the parameters of this estimator.

Parameters
****params** – Estimator parameters.

Returns
Estimator instance.

Return type
ProteinModelWrapper

set_score_request(*, *sample_weight: bool | None | str = '$UNCHANGED$'*) → *HMMWrapper*

Request metadata passed to the `score` method.

Note that this method is only relevant if `enable_metadata_routing=True` (see `sklearn.set_config()`). Please see User Guide on how the routing mechanism works.

The options for each parameter are:

- `True`: metadata is requested, and passed to `score` if provided. The request is ignored if metadata is not provided.
- `False`: metadata is not requested and the meta-estimator will not pass it to `score`.
- `None`: metadata is not requested, and the meta-estimator will raise an error if the user provides it.
- `str`: metadata should be passed to the meta-estimator with this given alias instead of the original name.

The default (`sklearn.utils.metadata_routing.UNCHANGED`) retains the existing request. This allows you to change the request for some parameters and not others.

Added in version 1.3.

Note: This method is only relevant if this estimator is used as a sub-estimator of a meta-estimator, e.g. used inside a `Pipeline`. Otherwise it has no effect.

Parameters
sample_weight (*str, True, False, or None, default=sklearn.utils.metadata_routing.UNCHANGED*) – Metadata routing for `sample_weight` parameter in `score`.

Returns
self – The updated object.

Return type
object

property should_refit_on_sequences: bool

Whether the model should refit on new sequences when given.

transform(*X: ProteinSequences | List[str]*) → ndarray

Transform sequences with pre-processing and post-processing from mixins.

Parameters
X (*Union[ProteinSequences, List[str]]*) – Input sequences.

Returns
Transformed sequences.

Return type
np.ndarray

property wt

aide_predict.bespoke_models.predictors.msa_transformer module

- Author: Evan Komp
- Created: 7/8/2024
- Company: National Renewable Energy Lab, Bioeneergy Science and Technology
- License: MIT

class aide_predict.bespoke_models.predictors.msa_transformer.**MSATransformerLikelihoodWrapper**(*metadata_fol[illegible] str | None = None*, *marginal_me[illegible]* Marginal-Method *= Marginal-Method.WILL[illegible]*, *po-si-tions: List[int] | None = None*, *flat-ten: bool = False*, *pool: bool = True*, *batch_size: int = 32*, *de-vice: str = 'cpu'*, *n_msa_seqs: int = 360*, *use_cache: bool = False*, *wt: str |* Pro-tein-Se-quence *| None = None*)

Bases: *RequiresMSAPerSequenceMixin*, *LikelihoodTransformerBase*

A wrapper for the MSA Transformer model to compute log likelihoods for protein sequences.

This class uses the MSA Transformer model to calculate log likelihoods for protein sequences based on multiple sequence alignments (MSAs). It supports various marginal likelihood calculation methods and can handle masked positions.

Variables

_available (MessageBool) – Indicates whether the MSA Transformer model is available.

__init__(*metadata_folder: str | None = None*, *marginal_method:* MarginalMethod *= MarginalMethod.WILDTYPE*, *positions: List[int] | None = None*, *flatten: bool = False*, *pool: bool = True*, *batch_size: int = 32*, *device: str = 'cpu'*, *n_msa_seqs: int = 360*, *use_cache: bool = False*, *wt: str |* ProteinSequence *| None = None*)

Initialize the MSATransformerLikelihoodWrapper.

Parameters

- **metadata_folder** (*str*) – Folder to store metadata.
- **marginal_method** (MarginalMethod) – Method to compute marginal likelihoods.
- **positions** (*Optional[List[int]]*) – Specific positions to consider.
- **flatten** (*bool*) – Whether to flatten the output.
- **pool** (*bool*) – Whether to pool the likelihoods across positions.
- **batch_size** (*int*) – Number of sequences to process in each batch.
- **device** (*str*) – Device to use for computations ('cpu' or 'cuda').
- **wt** (*Optional[Union[str,* ProteinSequence*]]*) – Wild type sequence.

property accepts_lower_case: bool

Whether the model can accept lower case sequences.

property can_handle_aligned_sequences: bool

Whether the model can handle aligned sequences (with gaps) at predict time.

property can_regress: bool

Whether the model can perform regression.

check_metadata() → None

Ensures that everything this model class needs is in the metadata folder.

property expects_no_fit: bool

Whether the model expects no fit.

fit(*X:* ProteinSequences *| List[str] | None = None*, *y: ndarray | None = None*, *force: bool = False*) → *ProteinModelWrapper*

Fit the model with pre-processing and post-processing from mixins.

Parameters

- **X** (*Union[*ProteinSequences*, List[str]]*) – Input sequences.
- **y** (*Optional[np.ndarray]*) – Target values.
- **force** (*bool*) – Whether to force refitting if already fitted.

Returns
The fitted model.

Return type
ProteinModelWrapper

`fit_transform`(*X*, *y=None*, ***fit_params*)

Fit to data, then transform it.

Fits transformer to *X* and *y* with optional parameters *fit_params* and returns a transformed version of *X*.

Parameters

- **X** (*array-like of shape (n_samples, n_features)*) – Input samples.
- **y** (*array-like of shape (n_samples,) or (n_samples, n_outputs), default=None*) – Target values (None for unsupervised transformations).
- ****fit_params** (*dict*) – Additional fit parameters.

Returns
X_new – Transformed array.

Return type
ndarray array of shape (n_samples, n_features_new)

`get_feature_names_out`(*input_features: List[str] | None = None*) → List[str]

Get output feature names for transformation.

Parameters
input_features (*Optional[List[str]]*) – Input feature names (not used in this method).

Returns
Output feature names.

Return type
List[str]

Raises
ValueError – If the model hasn't been fitted or if feature names can't be generated.

`get_fitted_attributes`() → List[str]

Get a list of attributes that are set during fitting.

`get_metadata_routing`()

Get metadata routing of this object.

Please check User Guide on how the routing mechanism works.

Returns
routing – A `MetadataRequest` encapsulating routing information.

Return type
MetadataRequest

`get_params`(*deep: bool = True*) → Dict[str, Any]

Get parameters for this estimator.

Parameters
deep (*bool*) – If True, will return the parameters for this estimator and contained subobjects.

Returns
Parameter names mapped to their values.

Return type
Dict[str, Any]

property metadata_folder

partial_fit(*X:* ProteinSequences | *List[str]*, *y: ndarray* | *None* = *None*) → *ProteinModelWrapper*

Partially fit the model to the given sequences.

This method can be called multiple times to incrementally fit the model.

Parameters

- **X** (*Union[ProteinSequences, List[str]]*) – The input sequences to partially fit the model on.
- **y** (*Optional[np.ndarray]*) – The target values, if applicable.

Returns
The partially fitted model.

Return type
ProteinModelWrapper

property per_position_capable: bool

Whether the model can output per position scores.

predict(*X:* ProteinSequences | *List[str]*) → ndarray

Predict the sequences.

Parameters
X (*Union[ProteinSequences, List[str]]*) – Input sequences.

Returns
Predicted values.

Return type
np.ndarray

Raises
ValueError – If the model is not capable of regression.

property requires_fixed_length: bool

Whether the model requires fixed length input.

property requires_msa_for_fit: bool

Whether the model requires an MSA for fitting.

property requires_msa_per_sequence: bool

Whether the model requires an MSA for each sequence during transform.

property requires_structure: bool

Whether the model requires structure information.

property requires_wt_during_inference: bool

Whether the model requires wild type during inference.

property requires_wt_msa: bool

Whether the model requires a wild type MSA for fitting.

property requires_wt_to_function: bool

Whether the model requires the wild type sequence to function.

score(*X*, *y*, *sample_weight=None*)

Return the Spearman correlation

set_fit_request(*, *force: bool | None | str = '$UNCHANGED$'*) → *MSATransformerLikelihoodWrapper*

Request metadata passed to the `fit` method.

Note that this method is only relevant if `enable_metadata_routing=True` (see `sklearn.set_config()`). Please see User Guide on how the routing mechanism works.

The options for each parameter are:

- `True`: metadata is requested, and passed to `fit` if provided. The request is ignored if metadata is not provided.
- `False`: metadata is not requested and the meta-estimator will not pass it to `fit`.
- `None`: metadata is not requested, and the meta-estimator will raise an error if the user provides it.
- `str`: metadata should be passed to the meta-estimator with this given alias instead of the original name.

The default (`sklearn.utils.metadata_routing.UNCHANGED`) retains the existing request. This allows you to change the request for some parameters and not others.

Added in version 1.3.

Note: This method is only relevant if this estimator is used as a sub-estimator of a meta-estimator, e.g. used inside a `Pipeline`. Otherwise it has no effect.

Parameters

force (*str, True, False, or None, default=sklearn.utils.metadata_routing.UNCHANGED*) – Metadata routing for `force` parameter in `fit`.

Returns

self – The updated object.

Return type

object

set_output(*, *transform=None*)

Set output container.

See sphx_glr_auto_examples_miscellaneous_plot_set_output.py for an example on how to use the API.

Parameters

transform (*{"default", "pandas", "polars"}, default=None*) – Configure output of *transform* and *fit_transform*.

- *"default"*: Default output format of a transformer
- *"pandas"*: DataFrame output
- *"polars"*: Polars output
- *None*: Transform configuration is unchanged

Added in version 1.4: *"polars"* option was added.

Returns

self – Estimator instance.

Return type
estimator instance

`set_params`(***params: Any*) → *ProteinModelWrapper*

Set the parameters of this estimator.

Parameters
`**params` – Estimator parameters.

Returns
Estimator instance.

Return type
ProteinModelWrapper

`set_score_request`(*, *sample_weight: bool | None | str = '$UNCHANGED$'*) → *MSATransformerLikelihoodWrapper*

Request metadata passed to the `score` method.

Note that this method is only relevant if `enable_metadata_routing=True` (see `sklearn.set_config()`). Please see User Guide on how the routing mechanism works.

The options for each parameter are:

- `True`: metadata is requested, and passed to `score` if provided. The request is ignored if metadata is not provided.
- `False`: metadata is not requested and the meta-estimator will not pass it to `score`.
- `None`: metadata is not requested, and the meta-estimator will raise an error if the user provides it.
- `str`: metadata should be passed to the meta-estimator with this given alias instead of the original name.

The default (`sklearn.utils.metadata_routing.UNCHANGED`) retains the existing request. This allows you to change the request for some parameters and not others.

Added in version 1.3.

Note: This method is only relevant if this estimator is used as a sub-estimator of a meta-estimator, e.g. used inside a `Pipeline`. Otherwise it has no effect.

Parameters
sample_weight (*str, True, False, or None, default=sklearn.utils.metadata_routing.UNCHANGED*) – Metadata routing for `sample_weight` parameter in `score`.

Returns
self – The updated object.

Return type
object

property should_refit_on_sequences: bool

Whether the model should refit on new sequences when given.

`transform`(*X:* ProteinSequences | *List[str]*) → ndarray

Transform sequences with pre-processing and post-processing from mixins.

Parameters
X (*Union[ProteinSequences, List[str]]*) – Input sequences.

Returns
Transformed sequences.

Return type
np.ndarray

property wt

aide_predict.bespoke_models.predictors.pretrained_transformers module

- Author: Evan Komp
- Created: 7/11/2024
- Company: National Renewable Energy Lab, Bioeneergy Science and Technology
- License: MIT

Base class for log likelihood based transformer models. Supports wildtype, mutant, and masked marginal methods.

See: Meier, J. et al. Language models enable zero-shot prediction of the effects of mutations on protein function. Preprint at https://doi.org/10.1101/2021.07.09.450648 (2021).

class aide_predict.bespoke_models.predictors.pretrained_transformers.**LikelihoodTransformerBase**(*metadata_[illegible] str | None = None*, *marginal_[illegible] MarginalMethod = 'wild-type_marg[illegible]*, *positions: List[int] | None = None*, *flatten: bool = False*, *pool: bool = True*, *batch_size[illegible] int = 2*, *device: str = 'cpu'*, *wt: str | ProteinSequence | None = None*, ***kwargs*)

Bases: *PositionSpecificMixin*, *RequiresFixedLengthMixin*, *ExpectsNoFitMixin*, *CanRegressMixin*, *RequiresWTDuringInferenceMixin*, *CacheMixin*, *ProteinModelWrapper*, ABC

Base class for likelihood transformer models.

This abstract base class provides a framework for implementing likelihood transformer models that can compute various types of marginal likelihoods for protein sequences.

Variables

- **marginal_method** (MarginalMethod) – The method used to compute marginal likelihoods.
- **batch_size** (*int*) – The number of sequences to process in each batch.
- **device** (*str*) – The device to use for computations ('cpu' or 'cuda').

__init__(*metadata_folder: str | None = None*, *marginal_method:* MarginalMethod *= 'wildtype_marginal'*, *positions: List[int] | None = None*, *flatten: bool = False*, *pool: bool = True*, *batch_size: int = 2*, *device: str = 'cpu'*, *wt: str |* ProteinSequence *| None = None*, ***kwargs*)

Initialize the LikelihoodTransformerBase.

Parameters

- **metadata_folder** (*str*) – Folder to store metadata.
- **marginal_method** (MarginalMethod) – Method to compute marginal likelihoods.
- **positions** (*Optional[List[int]]*) – Specific positions to consider.
- **flatten** (*bool*) – Whether to flatten the output.
- **pool** (*bool*) – Whether to pool the likelihoods across positions.
- **batch_size** (*int*) – Number of sequences to process in each batch.
- **device** (*str*) – Device to use for computations ('cpu' or 'cuda').
- **wt** (*Optional[Union[str,* ProteinSequence*]]*) – Wild type sequence.

property accepts_lower_case: bool

Whether the model can accept lower case sequences.

property can_handle_aligned_sequences: bool

Whether the model can handle aligned sequences (with gaps) at predict time.

property can_regress: bool

Whether the model can perform regression.

check_metadata() → None

Ensures that everything this model class needs is in the metadata folder.

property expects_no_fit: bool

Whether the model expects no fit.

fit(*X:* ProteinSequences *| List[str] | None = None*, *y: ndarray | None = None*, *force: bool = False*) → *ProteinModelWrapper*

Fit the model with pre-processing and post-processing from mixins.

Parameters

- **X** (*Union[*ProteinSequences*, List[str]]*) – Input sequences.
- **y** (*Optional[np.ndarray]*) – Target values.
- **force** (*bool*) – Whether to force refitting if already fitted.

Returns

The fitted model.

Return type
ProteinModelWrapper

fit_transform(*X*, *y=None*, ***fit_params*)

Fit to data, then transform it.

Fits transformer to *X* and *y* with optional parameters *fit_params* and returns a transformed version of *X*.

Parameters

- **X** (*array-like of shape (n_samples, n_features)*) – Input samples.
- **y** (*array-like of shape (n_samples,) or (n_samples, n_outputs), default=None*) – Target values (None for unsupervised transformations).
- ****fit_params** (*dict*) – Additional fit parameters.

Returns
X_new – Transformed array.

Return type
ndarray array of shape (n_samples, n_features_new)

get_feature_names_out(*input_features: List[str] | None = None*) → List[str]

Get output feature names for transformation.

Parameters
input_features (*Optional[List[str]]*) – Input feature names (not used in this method).

Returns
Output feature names.

Return type
List[str]

Raises
ValueError – If the model hasn't been fitted or if feature names can't be generated.

get_fitted_attributes() → List[str]

Get a list of attributes that are set during fitting.

get_metadata_routing()

Get metadata routing of this object.

Please check User Guide on how the routing mechanism works.

Returns
routing – A `MetadataRequest` encapsulating routing information.

Return type
MetadataRequest

get_params(*deep: bool = True*) → Dict[str, Any]

Get parameters for this estimator.

Parameters
deep (*bool*) – If True, will return the parameters for this estimator and contained subobjects.

Returns
Parameter names mapped to their values.

Return type
Dict[str, Any]

property metadata_folder

partial_fit(*X: ProteinSequences | List[str], y: ndarray | None = None*) → *ProteinModelWrapper*

Partially fit the model to the given sequences.

This method can be called multiple times to incrementally fit the model.

Parameters

- **X** (*Union[ProteinSequences, List[str]]*) – The input sequences to partially fit the model on.
- **y** (*Optional[np.ndarray]*) – The target values, if applicable.

Returns

The partially fitted model.

Return type

ProteinModelWrapper

property per_position_capable: bool

Whether the model can output per position scores.

predict(*X: ProteinSequences | List[str]*) → ndarray

Predict the sequences.

Parameters

X (*Union[ProteinSequences, List[str]]*) – Input sequences.

Returns

Predicted values.

Return type

np.ndarray

Raises

ValueError – If the model is not capable of regression.

property requires_fixed_length: bool

Whether the model requires fixed length input.

property requires_msa_for_fit: bool

Whether the model requires an MSA for fitting.

property requires_msa_per_sequence: bool

Whether the model requires an MSA for each sequence during transform.

property requires_structure: bool

Whether the model requires structure information.

property requires_wt_during_inference: bool

Whether the model requires wild type during inference.

property requires_wt_msa: bool

Whether the model requires a wild type MSA for fitting.

property requires_wt_to_function: bool

Whether the model requires the wild type sequence to function.

score(*X, y, sample_weight=None*)

Return the Spearman correlation

`set_fit_request`(*, *force: bool | None | str = '$UNCHANGED$'*) → *LikelihoodTransformerBase*

Request metadata passed to the `fit` method.

Note that this method is only relevant if `enable_metadata_routing=True` (see `sklearn.set_config()`). Please see User Guide on how the routing mechanism works.

The options for each parameter are:

- `True`: metadata is requested, and passed to `fit` if provided. The request is ignored if metadata is not provided.
- `False`: metadata is not requested and the meta-estimator will not pass it to `fit`.
- `None`: metadata is not requested, and the meta-estimator will raise an error if the user provides it.
- `str`: metadata should be passed to the meta-estimator with this given alias instead of the original name.

The default (`sklearn.utils.metadata_routing.UNCHANGED`) retains the existing request. This allows you to change the request for some parameters and not others.

Added in version 1.3.

Note: This method is only relevant if this estimator is used as a sub-estimator of a meta-estimator, e.g. used inside a `Pipeline`. Otherwise it has no effect.

Parameters

force (*str, True, False, or None, default=sklearn.utils.metadata_routing.UNCHANGED*) – Metadata routing for `force` parameter in `fit`.

Returns

self – The updated object.

Return type

object

`set_output`(*, *transform=None*)

Set output container.

See sphx_glr_auto_examples_miscellaneous_plot_set_output.py for an example on how to use the API.

Parameters

transform (*{"default", "pandas", "polars"}, default=None*) – Configure output of *transform* and *fit_transform*.

- *"default"*: Default output format of a transformer
- *"pandas"*: DataFrame output
- *"polars"*: Polars output
- *None*: Transform configuration is unchanged

Added in version 1.4: *"polars"* option was added.

Returns

self – Estimator instance.

Return type

estimator instance

set_params(***params: Any*) → *ProteinModelWrapper*

Set the parameters of this estimator.

Parameters

****params** – Estimator parameters.

Returns

Estimator instance.

Return type

ProteinModelWrapper

set_score_request(**, sample_weight: bool | None | str = '$UNCHANGED$'*) → *LikelihoodTransformerBase*

Request metadata passed to the `score` method.

Note that this method is only relevant if `enable_metadata_routing=True` (see `sklearn.set_config()`). Please see User Guide on how the routing mechanism works.

The options for each parameter are:

- `True`: metadata is requested, and passed to `score` if provided. The request is ignored if metadata is not provided.
- `False`: metadata is not requested and the meta-estimator will not pass it to `score`.
- `None`: metadata is not requested, and the meta-estimator will raise an error if the user provides it.
- `str`: metadata should be passed to the meta-estimator with this given alias instead of the original name.

The default (`sklearn.utils.metadata_routing.UNCHANGED`) retains the existing request. This allows you to change the request for some parameters and not others.

Added in version 1.3.

Note: This method is only relevant if this estimator is used as a sub-estimator of a meta-estimator, e.g. used inside a `Pipeline`. Otherwise it has no effect.

Parameters

sample_weight (*str, True, False, or None, default=sklearn.utils.metadata_routing.UNCHANGED*) – Metadata routing for `sample_weight` parameter in `score`.

Returns

self – The updated object.

Return type

object

property should_refit_on_sequences: bool

Whether the model should refit on new sequences when given.

transform(*X: ProteinSequences | List[str]*) → ndarray

Transform sequences with pre-processing and post-processing from mixins.

Parameters

X (*Union[ProteinSequences, List[str]]*) – Input sequences.

Returns
Transformed sequences.

Return type
np.ndarray

property wt

class aide_predict.bespoke_models.predictors.pretrained_transformers.**MarginalMethod**(*value*)

Bases: Enum

An enumeration.

MASKED = 'masked_marginal'

MUTANT = 'mutant_marginal'

WILDTYPE = 'wildtype_marginal'

class aide_predict.bespoke_models.predictors.pretrained_transformers.**ModelDeviceManager**(*model_instance: Any*, *device: str = 'cpu'*)

Bases: object

classmethod get_instance(*model_instance: Any*, *device: str*)

model_on_device(*load_func: Callable[[], None]*, *cleanup_func: Callable[[], None]*)

aide_predict.bespoke_models.predictors.pretrained_transformers.**model_device_context**(*model_instance: Any*, *load_func: Callable[[], None]*, *cleanup_func: Callable[[], None]*, *device: str = 'cpu'*)

Context manager used to load and clean up a model on a specific device.

This ensures model weights are not sitting on the GPU when not being accessed, unless the KEEP_MODEL_ON_DEVICE environment variable is set to True. If set to True, the model is loaded only once and kept on the device across multiple calls.

aide_predict.bespoke_models.predictors.saprot module

- Author: Evan Komp
- Created: 7/16/2024
- Company: National Renewable Energy Lab, Bioeneergy Science and Technology
- License: MIT

Wrapper around SaProt model. Please see here and all credit to the oroginal authors for their method and model: https://www.biorxiv.org/content/10.1101/2023.10.01.560349v2

class aide_predict.bespoke_models.predictors.saprot.**SaProtLikelihoodWrapper**(*metadata_folder: str | None = None, model_checkpoint: str = 'westlake-repl/SaProt_650M_AF2', marginal_method:* MarginalMethod *= MarginalMethod.WILDTYPE, positions: list | None = None, pool: bool = True, flatten: bool = True, wt: str | None = None, batch_size: int = 2, use_cache: bool = False, device: str = 'cpu', foldseek_path: str = 'foldseek'*)

Bases: *RequiresStructureMixin*, *LikelihoodTransformerBase*

property accepts_lower_case: bool

Whether the model can accept lower case sequences.

property can_handle_aligned_sequences: bool

Whether the model can handle aligned sequences (with gaps) at predict time.

property can_regress: bool

Whether the model can perform regression.

check_metadata() → None

Ensures that everything this model class needs is in the metadata folder.

property expects_no_fit: bool

Whether the model expects no fit.

fit(*X:* ProteinSequences *| List[str] | None = None, y: ndarray | None = None, force: bool = False*) → *ProteinModelWrapper*

Fit the model with pre-processing and post-processing from mixins.

Parameters

- **X** (*Union[ProteinSequences, List[str]]*) – Input sequences.
- **y** (*Optional[np.ndarray]*) – Target values.
- **force** (*bool*) – Whether to force refitting if already fitted.

Returns
The fitted model.

Return type
ProteinModelWrapper

fit_transform(*X*, *y=None*, ***fit_params*)

Fit to data, then transform it.

Fits transformer to *X* and *y* with optional parameters *fit_params* and returns a transformed version of *X*.

Parameters

- **X** (*array-like of shape (n_samples, n_features)*) – Input samples.
- **y** (*array-like of shape (n_samples,) or (n_samples, n_outputs), default=None*) – Target values (None for unsupervised transformations).
- ****fit_params** (*dict*) – Additional fit parameters.

Returns
X_new – Transformed array.

Return type
ndarray array of shape (n_samples, n_features_new)

get_feature_names_out(*input_features: List[str] | None = None*) → List[str]

Get output feature names for transformation.

Parameters
input_features (*Optional[List[str]]*) – Input feature names (not used in this method).

Returns
Output feature names.

Return type
List[str]

Raises
ValueError – If the model hasn't been fitted or if feature names can't be generated.

get_fitted_attributes() → List[str]

Get a list of attributes that are set during fitting.

get_metadata_routing()

Get metadata routing of this object.

Please check User Guide on how the routing mechanism works.

Returns
routing – A `MetadataRequest` encapsulating routing information.

Return type
MetadataRequest

get_params(*deep: bool = True*) → Dict[str, Any]

Get parameters for this estimator.

Parameters

deep (*bool*) – If True, will return the parameters for this estimator and contained subobjects.

Returns

Parameter names mapped to their values.

Return type

Dict[str, Any]

property metadata_folder

partial_fit(*X: ProteinSequences | List[str], y: ndarray | None = None*) → *ProteinModelWrapper*

Partially fit the model to the given sequences.

This method can be called multiple times to incrementally fit the model.

Parameters

- **X** (*Union[ProteinSequences, List[str]]*) – The input sequences to partially fit the model on.
- **y** (*Optional[np.ndarray]*) – The target values, if applicable.

Returns

The partially fitted model.

Return type

ProteinModelWrapper

property per_position_capable: bool

Whether the model can output per position scores.

predict(*X: ProteinSequences | List[str]*) → ndarray

Predict the sequences.

Parameters

X (*Union[ProteinSequences, List[str]]*) – Input sequences.

Returns

Predicted values.

Return type

np.ndarray

Raises

ValueError – If the model is not capable of regression.

property requires_fixed_length: bool

Whether the model requires fixed length input.

property requires_msa_for_fit: bool

Whether the model requires an MSA for fitting.

property requires_msa_per_sequence: bool

Whether the model requires an MSA for each sequence during transform.

property requires_structure: bool

Whether the model requires structure information.

property requires_wt_during_inference: bool

Whether the model requires wild type during inference.

property requires_wt_msa: bool

Whether the model requires a wild type MSA for fitting.

property requires_wt_to_function: bool

Whether the model requires the wild type sequence to function.

score(*X*, *y*, *sample_weight=None*)

Return the Spearman correlation

set_fit_request(*, *force: bool | None | str = '$UNCHANGED$'*) → *SaProtLikelihoodWrapper*

Request metadata passed to the `fit` method.

Note that this method is only relevant if `enable_metadata_routing=True` (see `sklearn.set_config()`). Please see User Guide on how the routing mechanism works.

The options for each parameter are:

- `True`: metadata is requested, and passed to `fit` if provided. The request is ignored if metadata is not provided.
- `False`: metadata is not requested and the meta-estimator will not pass it to `fit`.
- `None`: metadata is not requested, and the meta-estimator will raise an error if the user provides it.
- `str`: metadata should be passed to the meta-estimator with this given alias instead of the original name.

The default (`sklearn.utils.metadata_routing.UNCHANGED`) retains the existing request. This allows you to change the request for some parameters and not others.

Added in version 1.3.

Note: This method is only relevant if this estimator is used as a sub-estimator of a meta-estimator, e.g. used inside a `Pipeline`. Otherwise it has no effect.

Parameters

force (*str, True, False, or None, default=sklearn.utils.metadata_routing.UNCHANGED*) – Metadata routing for `force` parameter in `fit`.

Returns

self – The updated object.

Return type

object

set_output(*, *transform=None*)

Set output container.

See sphx_glr_auto_examples_miscellaneous_plot_set_output.py for an example on how to use the API.

Parameters

transform (*{"default", "pandas", "polars"}, default=None*) – Configure output of *transform* and *fit_transform*.

- *"default"*: Default output format of a transformer
- *"pandas"*: DataFrame output

- *"polars"*: Polars output
- *None*: Transform configuration is unchanged

Added in version 1.4: *"polars"* option was added.

Returns
self – Estimator instance.

Return type
estimator instance

`set_params`(***params: Any*) → *ProteinModelWrapper*

Set the parameters of this estimator.

Parameters
`**params` – Estimator parameters.

Returns
Estimator instance.

Return type
ProteinModelWrapper

`set_score_request`(**, sample_weight: bool | None | str = '$UNCHANGED$'*) → *SaProtLikelihoodWrapper*

Request metadata passed to the `score` method.

Note that this method is only relevant if `enable_metadata_routing=True` (see `sklearn.set_config()`). Please see User Guide on how the routing mechanism works.

The options for each parameter are:

- `True`: metadata is requested, and passed to `score` if provided. The request is ignored if metadata is not provided.
- `False`: metadata is not requested and the meta-estimator will not pass it to `score`.
- `None`: metadata is not requested, and the meta-estimator will raise an error if the user provides it.
- `str`: metadata should be passed to the meta-estimator with this given alias instead of the original name.

The default (`sklearn.utils.metadata_routing.UNCHANGED`) retains the existing request. This allows you to change the request for some parameters and not others.

Added in version 1.3.

Note: This method is only relevant if this estimator is used as a sub-estimator of a meta-estimator, e.g. used inside a `Pipeline`. Otherwise it has no effect.

Parameters
`sample_weight` (*`str, True, False, or None, default=sklearn.utils.metadata_routing.UNCHANGED`*) – Metadata routing for `sample_weight` parameter in `score`.

Returns
self – The updated object.

Return type
object

property should_refit_on_sequences: bool

Whether the model should refit on new sequences when given.

transform(*X: ProteinSequences | List[str]*) → ndarray

Transform sequences with pre-processing and post-processing from mixins.

Parameters
X (*Union[ProteinSequences, List[str]]*) – Input sequences.

Returns
Transformed sequences.

Return type
np.ndarray

property wt

aide_predict.bespoke_models.predictors.saprot.**get_structure_tokens**(*structure: ProteinStructure, foldseek_path: str, process_id: int = 0, plddt_threshold: float = 70.0, return_seq_tokens: bool = False*) → str

Extract structure tokens from a ProteinStructure using FoldSeek.

Parameters

- **structure** (*ProteinStructure*) – The protein structure to process.
- **foldseek_path** (*str*) – Path to the FoldSeek executable.
- **process_id** (*int*) – Process ID for temporary files. Used for parallel processing.
- **plddt_threshold** (*float*) – Threshold for pLDDT scores. Regions below this are masked.

Returns
A string of structure tokens.

Return type
str

aide_predict.bespoke_models.predictors.ssemb module

- Author: Evan Komp
- Created: 4/2/2025
- Company: National Renewable Energy Lab, Bioeneergy Science and Technology
- License: MIT

Wrapper for SSEmb: Blaabjerg, L.M., Jonsson, N., Boomsma, W. et al. SSEmb: A joint embedding of protein sequence and structure enables robust variant effect predictions.

Nat Commun 15, 9646 (2024). https://doi.org/10.1038/s41467-024-53982-z

class aide_predict.bespoke_models.predictors.ssemb.**SSEmbWrapper**(*metadata_folder: str | None = None, wt: str | ProteinSequence | None = None, gpu_id: int = 0*)

Bases: *RequiresWTToFunctionMixin*, *RequiresWTDuringInferenceMixin*,

RequiresStructureMixin, *RequiresFixedLengthMixin*, *RequiresWTMSAMixin*, *CanRegressMixin*, *ProteinModelWrapper*

Wrapper for SSEmb model to predict variant effects on protein stability.

SSEmb combines protein structure and sequence information using a graph neural network and MSA Transformer to predict the effects of mutations. This wrapper provides an interface to run SSEmb within the AIDE framework.

The SSEmb model requires: 1. A multiple sequence alignment (MSA) for the protein family 2. A structure for the wild-type protein 3. A wild-type sequence reference

The model predicts a score for each variant, where higher scores indicate better predicted stability/function.

Variables

- **_available** (MessageBool) – Indicates whether SSEmb is available based on environment setup.
- **gpu_id** (*int*) – GPU device ID to use for model inference.
- **msa** (ProteinSequences) – The multiple sequence alignment used for training.
- **fitted** (*bool*) – Whether the model has been fitted.

__init__(*metadata_folder: str | None = None*, *wt: str |* ProteinSequence *| None = None*, *gpu_id: int = 0*)

Initialize the SSEmb wrapper.

Parameters

- **metadata_folder** (*str, optional*) – Folder to store metadata and intermediate files.
- **wt** (*Union[str,* ProteinSequence*], optional*) – Wild-type protein sequence.
- **gpu_id** (*int, optional*) – GPU device ID to use. Defaults to 0.

property accepts_lower_case: bool

Whether the model can accept lower case sequences.

property can_handle_aligned_sequences: bool

Whether the model can handle aligned sequences (with gaps) at predict time.

property can_regress: bool

Whether the model can perform regression.

check_metadata() → None

Ensures that everything this model class needs is in the metadata folder.

property expects_no_fit: bool

Whether the model expects no fit.

fit(*X:* ProteinSequences *| List[str] | None = None*, *y: ndarray | None = None*, *force: bool = False*) → *ProteinModelWrapper*

Fit the model with pre-processing and post-processing from mixins.

Parameters

- **X** (*Union[*ProteinSequences*, List[str]]*) – Input sequences.
- **y** (*Optional[np.ndarray]*) – Target values.
- **force** (*bool*) – Whether to force refitting if already fitted.

Returns

The fitted model.

Return type
ProteinModelWrapper

fit_transform(*X*, *y=None*, ***fit_params*)

Fit to data, then transform it.

Fits transformer to *X* and *y* with optional parameters *fit_params* and returns a transformed version of *X*.

Parameters

- **X** (*array-like of shape (n_samples, n_features)*) – Input samples.
- **y** (*array-like of shape (n_samples,) or (n_samples, n_outputs), default=None*) – Target values (None for unsupervised transformations).
- ****fit_params** (*dict*) – Additional fit parameters.

Returns
X_new – Transformed array.

Return type
ndarray array of shape (n_samples, n_features_new)

get_feature_names_out(*input_features: List[str] | None = None*) → List[str]

Get output feature names for transformation.

Parameters
input_features (*Optional[List[str]]*) – Input feature names (not used).

Returns
A list containing the name of the output feature.

Return type
List[str]

get_metadata_routing()

Get metadata routing of this object.

Please check User Guide on how the routing mechanism works.

Returns
routing – A `MetadataRequest` encapsulating routing information.

Return type
MetadataRequest

get_params(*deep: bool = True*) → Dict[str, Any]

Get parameters for this estimator.

Parameters
deep (*bool*) – If True, will return the parameters for this estimator and contained subobjects.

Returns
Parameter names mapped to their values.

Return type
Dict[str, Any]

property metadata_folder

partial_fit(*X: ProteinSequences | List[str], y: ndarray | None = None*) → *ProteinModelWrapper*

Partially fit the model to the given sequences.

This method can be called multiple times to incrementally fit the model.

Parameters

- **X** (*Union[ProteinSequences, List[str]]*) – The input sequences to partially fit the model on.
- **y** (*Optional[np.ndarray]*) – The target values, if applicable.

Returns
The partially fitted model.

Return type
ProteinModelWrapper

property per_position_capable: bool

Whether the model can output per position scores.

predict(*X: ProteinSequences | List[str]*) → ndarray

Predict the sequences.

Parameters
X (*Union[ProteinSequences, List[str]]*) – Input sequences.

Returns
Predicted values.

Return type
np.ndarray

Raises
ValueError – If the model is not capable of regression.

property requires_fixed_length: bool

Whether the model requires fixed length input.

property requires_msa_for_fit: bool

Whether the model requires an MSA for fitting.

property requires_msa_per_sequence: bool

Whether the model requires an MSA for each sequence during transform.

property requires_structure: bool

Whether the model requires structure information.

property requires_wt_during_inference: bool

Whether the model requires wild type during inference.

property requires_wt_msa: bool

Whether the model requires a wild type MSA for fitting.

property requires_wt_to_function: bool

Whether the model requires the wild type sequence to function.

score(*X, y, sample_weight=None*)

Return the Spearman correlation

set_fit_request(**, force: bool | None | str = '$UNCHANGED$'*) → *SSEmbWrapper*

Request metadata passed to the `fit` method.

Note that this method is only relevant if `enable_metadata_routing=True` (see `sklearn.set_config()`). Please see User Guide on how the routing mechanism works.

The options for each parameter are:

- `True`: metadata is requested, and passed to `fit` if provided. The request is ignored if metadata is not provided.
- `False`: metadata is not requested and the meta-estimator will not pass it to `fit`.
- `None`: metadata is not requested, and the meta-estimator will raise an error if the user provides it.
- `str`: metadata should be passed to the meta-estimator with this given alias instead of the original name.

The default (`sklearn.utils.metadata_routing.UNCHANGED`) retains the existing request. This allows you to change the request for some parameters and not others.

Added in version 1.3.

Note: This method is only relevant if this estimator is used as a sub-estimator of a meta-estimator, e.g. used inside a `Pipeline`. Otherwise it has no effect.

Parameters
force (*str, True, False, or None, default=sklearn.utils.metadata_routing.UNCHANGED*) – Metadata routing for `force` parameter in `fit`.

Returns
self – The updated object.

Return type
object

`set_output`(**, transform=None*)

Set output container.

See sphx_glr_auto_examples_miscellaneous_plot_set_output.py for an example on how to use the API.

Parameters
transform (*{"default", "pandas", "polars"}, default=None*) – Configure output of *transform* and *fit_transform*.

- *"default"*: Default output format of a transformer
- *"pandas"*: DataFrame output
- *"polars"*: Polars output
- *None*: Transform configuration is unchanged

Added in version 1.4: *"polars"* option was added.

Returns
self – Estimator instance.

Return type
estimator instance

`set_params`(***params: Any*) → *ProteinModelWrapper*

Set the parameters of this estimator.

Parameters
****params** – Estimator parameters.

Returns
Estimator instance.

Return type
ProteinModelWrapper

set_score_request(*, *sample_weight: bool | None | str = '$UNCHANGED$'*) → *SSEmbWrapper*

Request metadata passed to the `score` method.

Note that this method is only relevant if `enable_metadata_routing=True` (see `sklearn.set_config()`). Please see User Guide on how the routing mechanism works.

The options for each parameter are:

- `True`: metadata is requested, and passed to `score` if provided. The request is ignored if metadata is not provided.
- `False`: metadata is not requested and the meta-estimator will not pass it to `score`.
- `None`: metadata is not requested, and the meta-estimator will raise an error if the user provides it.
- `str`: metadata should be passed to the meta-estimator with this given alias instead of the original name.

The default (`sklearn.utils.metadata_routing.UNCHANGED`) retains the existing request. This allows you to change the request for some parameters and not others.

Added in version 1.3.

Note: This method is only relevant if this estimator is used as a sub-estimator of a meta-estimator, e.g. used inside a `Pipeline`. Otherwise it has no effect.

Parameters
sample_weight (*str, True, False, or None, default=sklearn.utils.metadata_routing.UNCHANGED*) – Metadata routing for `sample_weight` parameter in `score`.

Returns
self – The updated object.

Return type
object

property should_refit_on_sequences: bool

Whether the model should refit on new sequences when given.

transform(*X: ProteinSequences | List[str]*) → ndarray

Transform sequences with pre-processing and post-processing from mixins.

Parameters
X (*Union[ProteinSequences, List[str]]*) – Input sequences.

Returns
Transformed sequences.

Return type
np.ndarray

property wt

aide_predict.bespoke_models.predictors.vespa module

- Author: Evan Komp
- Created: 8/1/2024
- Company: National Renewable Energy Lab, Bioeneergy Science and Technology
- License: MIT

Wrapper of VESPA: Marquet, C. et al. Embeddings from protein language models predict conservation and variant effects. Hum Genet 141, 1629–1647 (2022).

This model embeds the sequences with a PLM, then uses the embeddings for a pretrained logistic regression model for conservation. These are input into a model to predict single mutation effects.

class aide_predict.bespoke_models.predictors.vespa.**VESPAWrapper**(*metadata_folder: str | None = None*, *wt: str | ProteinSequence | None = None*, *use_cache: bool = False*, *light: bool = True*)

Bases: *CanRegressMixin*, *ExpectsNoFitMixin*, *RequiresWTDuringInferenceMixin*, *RequiresWTToFunctionMixin*, *CacheMixin*, *ProteinModelWrapper*

A wrapper class for the VESPA (Variant Effect Score Prediction using Attention) model.

This class provides an interface to use VESPA within the AIDE framework, allowing for prediction of variant effects on protein sequences.

Variables

light (*bool*) – If True, uses the lighter VESPAl model. If False, uses the full VESPA model.

__init__(*metadata_folder: str | None = None*, *wt: str | ProteinSequence | None = None*, *use_cache: bool = False*, *light: bool = True*) → None

Initialize the VESPAWrapper.

Parameters

- **metadata_folder** (*Optional[str]*) – Folder to store metadata.
- **wt** (*Optional[Union[str, ProteinSequence]]*) – Wild-type protein sequence.
- **light** (*bool*) – If True, use the lighter VESPAl model. If False, use the full VESPA model.

property accepts_lower_case: bool

Whether the model can accept lower case sequences.

property can_handle_aligned_sequences: bool

Whether the model can handle aligned sequences (with gaps) at predict time.

property can_regress: bool

Whether the model can perform regression.

check_metadata() → None

Ensures that everything this model class needs is in the metadata folder.

property expects_no_fit: bool

Whether the model expects no fit.

fit(*X: ProteinSequences | List[str] | None = None*, *y: ndarray | None = None*, *force: bool = False*) → *ProteinModelWrapper*

Fit the model with pre-processing and post-processing from mixins.

Parameters

- **X** (*Union[ProteinSequences, List[str]]*) – Input sequences.
- **y** (*Optional[np.ndarray]*) – Target values.
- **force** (*bool*) – Whether to force refitting if already fitted.

Returns
The fitted model.

Return type
ProteinModelWrapper

fit_transform(*X*, *y=None*, ***fit_params*)

Fit to data, then transform it.

Fits transformer to *X* and *y* with optional parameters *fit_params* and returns a transformed version of *X*.

Parameters

- **X** (*array-like of shape (n_samples, n_features)*) – Input samples.
- **y** (*array-like of shape (n_samples,) or (n_samples, n_outputs), default=None*) – Target values (None for unsupervised transformations).
- ****fit_params** (*dict*) – Additional fit parameters.

Returns
X_new – Transformed array.

Return type
ndarray array of shape (n_samples, n_features_new)

get_feature_names_out(*input_features: List[str] | None = None*) → List[str]

Get the names of the output features.

Parameters
input_features (*Optional[List[str]]*) – Ignored. Present for API consistency.

Returns
A list containing the name of the output feature.

Return type
List[str]

get_fitted_attributes() → List[str]

Get a list of attributes that are set during fitting.

get_metadata_routing()

Get metadata routing of this object.

Please check User Guide on how the routing mechanism works.

Returns
routing – A `MetadataRequest` encapsulating routing information.

Return type
MetadataRequest

get_params(*deep: bool = True*) → Dict[str, Any]

Get parameters for this estimator.

Parameters
deep (*bool*) – If True, will return the parameters for this estimator and contained subobjects.

Returns
Parameter names mapped to their values.

Return type
Dict[str, Any]

property metadata_folder

partial_fit(*X:* ProteinSequences | *List[str]*, *y: ndarray* | *None* = *None*) → *ProteinModelWrapper*

Partially fit the model to the given sequences.

This method can be called multiple times to incrementally fit the model.

Parameters

- **X** (*Union[ProteinSequences, List[str]]*) – The input sequences to partially fit the model on.
- **y** (*Optional[np.ndarray]*) – The target values, if applicable.

Returns
The partially fitted model.

Return type
ProteinModelWrapper

property per_position_capable: bool

Whether the model can output per position scores.

predict(*X:* ProteinSequences | *List[str]*) → ndarray

Predict the sequences.

Parameters
X (*Union[ProteinSequences, List[str]]*) – Input sequences.

Returns
Predicted values.

Return type
np.ndarray

Raises
ValueError – If the model is not capable of regression.

property requires_fixed_length: bool

Whether the model requires fixed length input.

property requires_msa_for_fit: bool

Whether the model requires an MSA for fitting.

property requires_msa_per_sequence: bool

Whether the model requires an MSA for each sequence during transform.

property requires_structure: bool

Whether the model requires structure information.

property requires_wt_during_inference: bool

Whether the model requires wild type during inference.

property requires_wt_msa: bool

Whether the model requires a wild type MSA for fitting.

property requires_wt_to_function: bool

Whether the model requires the wild type sequence to function.

score(*X*, *y*, *sample_weight=None*)

Return the Spearman correlation

set_fit_request(*, *force: bool | None | str = '$UNCHANGED$'*) → *VESPAWrapper*

Request metadata passed to the `fit` method.

Note that this method is only relevant if `enable_metadata_routing=True` (see `sklearn.set_config()`). Please see User Guide on how the routing mechanism works.

The options for each parameter are:

- `True`: metadata is requested, and passed to `fit` if provided. The request is ignored if metadata is not provided.
- `False`: metadata is not requested and the meta-estimator will not pass it to `fit`.
- `None`: metadata is not requested, and the meta-estimator will raise an error if the user provides it.
- `str`: metadata should be passed to the meta-estimator with this given alias instead of the original name.

The default (`sklearn.utils.metadata_routing.UNCHANGED`) retains the existing request. This allows you to change the request for some parameters and not others.

Added in version 1.3.

Note: This method is only relevant if this estimator is used as a sub-estimator of a meta-estimator, e.g. used inside a `Pipeline`. Otherwise it has no effect.

Parameters

force (*str, True, False, or None, default=sklearn.utils.metadata_routing.UNCHANGED*) – Metadata routing for `force` parameter in `fit`.

Returns

self – The updated object.

Return type

object

set_output(*, *transform=None*)

Set output container.

See sphx_glr_auto_examples_miscellaneous_plot_set_output.py for an example on how to use the API.

Parameters

transform (*{"default", "pandas", "polars"}, default=None*) – Configure output of *transform* and *fit_transform*.

- *"default"*: Default output format of a transformer
- *"pandas"*: DataFrame output
- *"polars"*: Polars output
- *None*: Transform configuration is unchanged

Added in version 1.4: *"polars"* option was added.

Returns
self – Estimator instance.

Return type
estimator instance

`set_params`(***params: Any*) → *ProteinModelWrapper*

Set the parameters of this estimator.

Parameters
`**params` – Estimator parameters.

Returns
Estimator instance.

Return type
ProteinModelWrapper

`set_score_request`(*, *sample_weight: bool | None | str = '$UNCHANGED$'*) → *VESPAWrapper*

Request metadata passed to the `score` method.

Note that this method is only relevant if `enable_metadata_routing=True` (see `sklearn.set_config()`). Please see User Guide on how the routing mechanism works.

The options for each parameter are:

- `True`: metadata is requested, and passed to `score` if provided. The request is ignored if metadata is not provided.
- `False`: metadata is not requested and the meta-estimator will not pass it to `score`.
- `None`: metadata is not requested, and the meta-estimator will raise an error if the user provides it.
- `str`: metadata should be passed to the meta-estimator with this given alias instead of the original name.

The default (`sklearn.utils.metadata_routing.UNCHANGED`) retains the existing request. This allows you to change the request for some parameters and not others.

Added in version 1.3.

Note: This method is only relevant if this estimator is used as a sub-estimator of a meta-estimator, e.g. used inside a `Pipeline`. Otherwise it has no effect.

Parameters
sample_weight (*str, True, False, or None, default=sklearn.utils.metadata_routing.UNCHANGED*) – Metadata routing for `sample_weight` parameter in `score`.

Returns
self – The updated object.

Return type
object

`property should_refit_on_sequences: bool`

Whether the model should refit on new sequences when given.

transform(*X: ProteinSequences | List[str]*) → ndarray

Transform sequences with pre-processing and post-processing from mixins.

Parameters
X (*Union[ProteinSequences, List[str]]*) – Input sequences.

Returns
Transformed sequences.

Return type
np.ndarray

property wt

Module contents

- Author: Evan Komp
- Created: 6/26/2024
- Company: Bottle Institute @ National Renewable Energy Lab, Bioeneergy Science and Technology
- License: MIT

Submodules

aide_predict.bespoke_models.base module

- Author: Evan Komp
- Created: 5/7/2024
- (c) Copyright by Bottle Institute @ National Renewable Energy Lab, Bioeneergy Science and Technology

Base classes for models to be wrapped into the API as sklearn estimators

class aide_predict.bespoke_models.base.**AcceptsLowerCaseMixin**

Bases: `object`

Mixin to indicate that a model can accept lower case sequences.

This mixin overrides the accepts_lower_case attribute to be True.

class aide_predict.bespoke_models.base.**CacheMixin**

Bases: `object`

Mixin to provide per-protein caching functionality for ProteinModelWrapper subclasses.

This mixin adds efficient caching of model outputs to avoid redundant computations. It uses SQLite for metadata indexing and HDF5 for efficient embedding storage, optimized for batch operations and improved file handling.

Variables
use_cache (*bool*) – Whether to enable caching. Default is True.

get_fitted_attributes() → List[str]

Get a list of attributes that are set during fitting.

class aide_predict.bespoke_models.base.**CanHandleAlignedSequencesMixin**

Bases: object

Mixin to indicate that a model can handle aligned sequences (with gaps) during prediction.

This mixin overrides the can_handle_aligned_sequences attribute to be True.

class aide_predict.bespoke_models.base.**CanRegressMixin**

Bases: RegressorMixin

Mixin to ensure model can regress.

This mixin overrides the can_regress attribute to be True. It also overrides the score method to use spearman correlation isntead of R2, such that it can be used out of the mox with zero shot predicors.

score(*X*, *y*, *sample_weight=None*)

Return the Spearman correlation

class aide_predict.bespoke_models.base.**ExpectsNoFitMixin**

Bases: object

Either because the model is pretrained, or it will be trained based on WT sequence information only, the model expects the fit method to recieve None.

class aide_predict.bespoke_models.base.**PositionSpecificMixin**

Bases: object

Mixin for protein models that can output per position scores.

This mixin adds functionality for handling position-specific outputs from protein models. It allows selecting specific positions to analyze, pooling across positions, and flattening multi-dimensional outputs.

Variables

- **positions** (*Optional[List[int]]*) – The positions to output scores for. If None, all positions are used.
- **pool** (*Union[bool, str, Callable]*) – Whether/how to pool scores across positions: - If True: Uses mean pooling - If string: Uses named numpy function (e.g. 'mean', 'max') - If callable: Uses the provided function for pooling - If False: No pooling is performed
- **flatten** (*bool*) – Whether to flatten dimensions beyond the second dimension.

get_feature_names_out(*input_features=None*)

Get output feature names for transformation, considering position-specific output and flattening.

This method overrides the base implementation to provide more descriptive feature names that include position information when relevant.

Parameters

input_features (*Optional[List[str]]*) – Input feature names (unused).

Returns

Output feature names.

Return type

List[str]

class aide_predict.bespoke_models.base.**ProteinModelWrapper**(*metadata_folder: str | None = None*, *wt: str | ProteinSequence | None = None*, ***kwargs*)

Bases: `TransformerMixin`, `BaseEstimator`

Base class for bespoke models that take proteins as input.

This class serves as a foundation for creating protein-based models that can be used in machine learning pipelines, particularly those compatible with scikit-learn. It provides a standard interface for fitting, transforming, and predicting protein sequences, as well as handling metadata and wild-type sequences.

All models that take proteins as input should inherit from this class. They are considered transformers and can be used natively to produce features in the AIDE pipeline. Models can additionally be made regressors by inheriting from RegressorMixin.

X values for fit, transform, and predict are expected to be ProteinSequences objects.

Variables

- **metadata_folder** (*str*) – The folder where the metadata is stored.
- **wt** (*Optional[ProteinSequence]*) – The wild type sequence if present.

Class Attributes:

expects_no_fit (bool): Whether the model expects no fit. requires_msa_for_fit (bool): Whether the model requires an MSA as input for fitting. requires_wt_msa (bool): Whether the model requires a wild type MSA for fitting. requires_msa_per_sequence (bool): Whether the model requires an MSA for each sequence during transform. requires_wt_to_function (bool): Whether the model requires the wild type sequence to function. requires_wt_during_inference (bool): Whether the any wt is used direectly in model inference, otherwise normalize by any wt per_position_capable (bool): Whether the model can output per position scores. requires_fixed_length (bool): Whether the model requires a fixed length input. can_regress (bool): Whether the model outputs from transform can also be considered estimates of activity label. can_handle_aligned_sequences (bool): Whether the model can handle unaligned sequences at predict time. should_refit_on_sequences (bool): Whether the model should refit on new sequences when given. requires_structure (bool): Whether the model requires structure information. _available (bool): Flag to indicate whether the model is available for use.

To subclass ProteinModelWrapper: 1. Implement the abstract methods:

- _fit(self, X: ProteinSequences, y: Optional[np.ndarray] = None) -> None
- _transform(self, X: ProteinSequences) -> np.ndarray

2. If your model supports partial fitting, implement: - _partial_fit(self, X: ProteinSequences, y: Optional[np.ndarray] = None) -> None
3. If your model requires specific metadata, override: - check_metadata(self) -> None - _construct_necessary_metadata(cls, model_directory: str, necessary_metadata: dict) -> None
4. If your model has additional parameters, implement __init__ and call super().__init__ with the metadata_folder and wt arguments.
5. If your model requires specific behavior, consider inheriting from the provided mixins. See the mixins for the provided behaviors: - ExpectsNoFitMixin - if the model expects no fit - RequiresMSAForFitMixin - if the model requires aligned sequences at fit time - RequiresWTMSAMixin - if the model requires a wild type MSA for fit - RequiresMSAPerSequenceMixin - if the model requires an MSA for each sequence during transform - RequiresFixedLengthMixin - if the model requires fixed length sequences at predict time - CanRegressMixin - if the model can regress, otherwise it is assumed to be a transformer only eg. embedding - RequiresWTToFunctionMixin - if the model requires the wild type sequence to function - RequiresWTDuringInferenceMixin - if the model requires the wild type sequence duing inference in order to normalize by wt - PositionSpecificMixin - if the model can output per position scores - RequiresStructureMixin - if

the model requires structure information - AcceptsLowerCaseMixin - if the model can accept lower case sequences - ShouldRefitOnSequencesMixin - if the model should refit on new sequences when given. Often, we are calling fit on NOT raw sequences, eg. MSAs.

> We still want to be able to use the model in the context of sklearn pipelines which will attempt to clone and refit the model on X data. We want the models to return themselves already fitted when cloned, unless this is mixex in

6. If the model requires more than the base package, set the _available attribute to be dynamic based on a check in the module.

Example

ESM2 using WT marginal can be used as a "regressor".

try:
import transformers AVALABLE = MessageBool(True, "This model is available.")

except ImportError:
AVALABLE = MessageBool(False, "This model is not available, make sure transformers is installed.")

class ESM2Model(CanRegressMixin, PositionSpecificMixin, ProteinModelWrapper):
_available = AVAILABLE

def __init__(self, model_checkpoint: str, metadata_folder: str, wt: Optional[Union[str, ProteinSequence]] = None):
super().__init__(metadata_folder, wt) self.model_checkpoint = model_checkpoint

def _fit(self, X: ProteinSequences, y: Optional[np.ndarray] = None) -> None:
Fit the model … return self

def _transform(self, X: ProteinSequences) -> np.ndarray:
Transform the sequences … return outputs

`__init__`(*metadata_folder: str* | *None = None*, *wt: str* | ProteinSequence | *None = None*, ***kwargs*)

Initialize the ProteinModelWrapper with core attributes and process mixin initializations.

Parameters

- `metadata_folder` (*str*) – The folder where the metadata is stored.
- `wt` (*Optional[Union[str, ProteinSequence]]*) – The wild type sequence if present.
- `**kwargs` – Additional keyword arguments handled by mixins.

Raises

`ValueError` – If the wild type sequence contains gaps or if the model is not available.

`property accepts_lower_case: bool`

Whether the model can accept lower case sequences.

`property can_handle_aligned_sequences: bool`

Whether the model can handle aligned sequences (with gaps) at predict time.

`property can_regress: bool`

Whether the model can perform regression.

`check_metadata()` → None

Ensures that everything this model class needs is in the metadata folder.

property expects_no_fit: bool

Whether the model expects no fit.

fit(*X: ProteinSequences | List[str] | None = None*, *y: ndarray | None = None*, *force: bool = False*) → *ProteinModelWrapper*

Fit the model with pre-processing and post-processing from mixins.

Parameters

- **X** (*Union[ProteinSequences, List[str]]*) – Input sequences.
- **y** (*Optional[np.ndarray]*) – Target values.
- **force** (*bool*) – Whether to force refitting if already fitted.

Returns

The fitted model.

Return type

ProteinModelWrapper

fit_transform(*X*, *y=None*, ***fit_params*)

Fit to data, then transform it.

Fits transformer to *X* and *y* with optional parameters *fit_params* and returns a transformed version of *X*.

Parameters

- **X** (*array-like of shape (n_samples, n_features)*) – Input samples.
- **y** (*array-like of shape (n_samples,) or (n_samples, n_outputs), default=None*) – Target values (None for unsupervised transformations).
- ****fit_params** (*dict*) – Additional fit parameters.

Returns

X_new – Transformed array.

Return type

ndarray array of shape (n_samples, n_features_new)

get_feature_names_out(*input_features: List[str] | None = None*) → List[str]

Get output feature names for transformation.

Parameters

input_features (*Optional[List[str]]*) – Input feature names.

Returns

Output feature names.

Return type

List[str]

get_metadata_routing()

Get metadata routing of this object.

Please check User Guide on how the routing mechanism works.

Returns

routing – A `MetadataRequest` encapsulating routing information.

Return type

MetadataRequest

get_params(*deep: bool = True*) → Dict[str, Any]

Get parameters for this estimator.

Parameters
deep (*bool*) – If True, will return the parameters for this estimator and contained subobjects.

Returns
Parameter names mapped to their values.

Return type
Dict[str, Any]

property metadata_folder

partial_fit(*X: ProteinSequences | List[str], y: ndarray | None = None*) → *ProteinModelWrapper*

Partially fit the model to the given sequences.

This method can be called multiple times to incrementally fit the model.

Parameters

- **X** (*Union[ProteinSequences, List[str]]*) – The input sequences to partially fit the model on.
- **y** (*Optional[np.ndarray]*) – The target values, if applicable.

Returns
The partially fitted model.

Return type
ProteinModelWrapper

property per_position_capable: bool

Whether the model can output per position scores.

predict(*X: ProteinSequences | List[str]*) → ndarray

Predict the sequences.

Parameters
X (*Union[ProteinSequences, List[str]]*) – Input sequences.

Returns
Predicted values.

Return type
np.ndarray

Raises
ValueError – If the model is not capable of regression.

property requires_fixed_length: bool

Whether the model requires fixed length input.

property requires_msa_for_fit: bool

Whether the model requires an MSA for fitting.

property requires_msa_per_sequence: bool

Whether the model requires an MSA for each sequence during transform.

property requires_structure: bool

Whether the model requires structure information.

property requires_wt_during_inference: bool

Whether the model requires wild type during inference.

property requires_wt_msa: bool

Whether the model requires a wild type MSA for fitting.

property requires_wt_to_function: bool

Whether the model requires the wild type sequence to function.

set_fit_request(*, *force: bool | None | str = '$UNCHANGED$'*) → *ProteinModelWrapper*

Request metadata passed to the `fit` method.

Note that this method is only relevant if `enable_metadata_routing=True` (see `sklearn.set_config()`). Please see User Guide on how the routing mechanism works.

The options for each parameter are:

- `True`: metadata is requested, and passed to `fit` if provided. The request is ignored if metadata is not provided.
- `False`: metadata is not requested and the meta-estimator will not pass it to `fit`.
- `None`: metadata is not requested, and the meta-estimator will raise an error if the user provides it.
- `str`: metadata should be passed to the meta-estimator with this given alias instead of the original name.

The default (`sklearn.utils.metadata_routing.UNCHANGED`) retains the existing request. This allows you to change the request for some parameters and not others.

Added in version 1.3.

Note: This method is only relevant if this estimator is used as a sub-estimator of a meta-estimator, e.g. used inside a `Pipeline`. Otherwise it has no effect.

Parameters

force (*str, True, False, or None, default=sklearn.utils.metadata_routing.UNCHANGED*) – Metadata routing for `force` parameter in `fit`.

Returns

self – The updated object.

Return type

object

set_output(*, *transform=None*)

Set output container.

See sphx_glr_auto_examples_miscellaneous_plot_set_output.py for an example on how to use the API.

Parameters

transform (*{"default", "pandas", "polars"}, default=None*) – Configure output of *transform* and *fit_transform*.

- *"default"*: Default output format of a transformer
- *"pandas"*: DataFrame output
- *"polars"*: Polars output
- *None*: Transform configuration is unchanged

Added in version 1.4: *"polars"* option was added.

Returns
self – Estimator instance.

Return type
estimator instance

`set_params`(***params: Any*) → *ProteinModelWrapper*

Set the parameters of this estimator.

Parameters
`**params` – Estimator parameters.

Returns
Estimator instance.

Return type
ProteinModelWrapper

`property should_refit_on_sequences: bool`

Whether the model should refit on new sequences when given.

`transform`(*X:* ProteinSequences | *List[str]*) → ndarray

Transform sequences with pre-processing and post-processing from mixins.

Parameters
X (*Union[ProteinSequences, List[str]]*) – Input sequences.

Returns
Transformed sequences.

Return type
np.ndarray

`property wt`

`class aide_predict.bespoke_models.base.RequiresFixedLengthMixin`

Bases: `object`

Mixin to ensure model receives fixed length sequences at transform.

This mixin overrides the requires_fixed_length attribute to be True.

`class aide_predict.bespoke_models.base.RequiresMSAForFitMixin`

Bases: `object`

Mixin to ensure model receives aligned sequences at fit.

This mixin overrides the requires_msa_for_fit attribute to be True.

`class aide_predict.bespoke_models.base.RequiresMSAPerSequenceMixin`

Bases: `object`

Mixin to ensure model receives sequences at transform that each have an MSA. If that fails it will attempt to find one via the wild type sequence.

`class aide_predict.bespoke_models.base.RequiresStructureMixin`

Bases: `object`

Mixin to ensure model requires structure information.

This mixin overrides the requires_structure attribute to be True.

class aide_predict.bespoke_models.base.**RequiresWTDuringInferenceMixin**

Bases: object

Mixin to indicate whether the model uses a wild type directly during inference, otherwise outputs are noramlized to any wt sequence.

This mixin overrides the requires_wt_during_inference attribute to be True.

class aide_predict.bespoke_models.base.**RequiresWTMSAMixin**

Bases: object

Mixin to ensure model's WT has an alignment available.

class aide_predict.bespoke_models.base.**RequiresWTToFunctionMixin**

Bases: object

Mixin to ensure model requires wild type to function.

This mixin overrides the requires_wt_to_function attribute to be True.

class aide_predict.bespoke_models.base.**ShouldRefitOnSequencesMixin**

Bases: object

Mixin to indicate that a model should refit on new sequences when given.

This mixin overrides the should_refit_on_sequences attribute to be True.

aide_predict.bespoke_models.base.**is_jsonable**(*x*)

Checks if an object is JSON serializable.

Module contents

- Author: Evan Komp
- Created: 5/7/2024
- (c) Copyright by Bottle Institute @ National Renewable Energy Lab, Bioeneergy Science and Technology

aide_predict.io package

Submodules

aide_predict.io.bio_files module

- Author: Evan Komp
- Created: 5/22/2024
- Company: Bottle Institute @ National Renewable Energy Lab, Bioeneergy Science and Technology
- License: MIT

Some functions are copied from EVcoupling, as to avoid the additional required dependancy. All credit goes to the EVcouples team:

Hopf T. A., Green A. G., Schubert B., et al. The EVcouplings Python framework for coevolutionary sequence analysis. Bioinformatics 35, 1582–1584 (2019)

`aide_predict.io.bio_files.read_a3m(fileobj, inserts='first')`

Read an alignment in compressed a3m format and expand into a2m format.

Credit to EVcouplings

Args: - fileobj: file object opened for reading - inserts: how to handle insert gaps in alignment

(either "first" or "delete")

Returns: - OrderedDict of sequence_id -> sequence

`aide_predict.io.bio_files.read_fasta(fileobj)`

Generator function to read a FASTA-format file (includes aligned FASTA, A2M, A3M formats)

Credit to EVcouplings

Args: - fileobj: file object opened for reading

Returns: - Tuple of (sequence_id, sequence) for each entry

`aide_predict.io.bio_files.write_fasta(sequences, fileobj, width=80)`

Write a list of IDs/sequences to a FASTA-format file

Credit to EVcouplings

Args: - sequences: list of (sequence_id, sequence) tuples - fileobj: file object opened for writing - width: width of sequence lines in FASTA file

Returns: - None

Module contents

- Author: Evan Komp
- Created: 5/7/2024
- (c) Copyright by Bottle Institute @ National Renewable Energy Lab, Bioeneergy Science and Technology

aide_predict.utils package

Subpackages

aide_predict.utils.data_structures package

Submodules

aide_predict.utils.data_structures.sequences module

- Author: Evan Komp
- Created: 6/21/2024
- Company: Bottle Institute @ National Renewable Energy Lab, Bioeneergy Science and Technology
- License: MIT

Base data structures for the AIDE Predict package Where they do not exist in sklearn.

class aide_predict.utils.data_structures.sequences.**ProteinCharacter**(*seq: str*)

Bases: str

Represents a single character in a protein sequence.

This class inherits from UserString and provides additional properties to check the nature of the amino acid character.

capitalize()

Return a capitalized version of the string.

More specifically, make the first character have upper case and the rest lower case.

casefold()

Return a version of the string suitable for caseless comparisons.

center(*width*, *fillchar=' '*, */*)

Return a centered string of length width.

Padding is done using the specified fill character (default is a space).

count(*sub*[, *start*[, *end*]]) → int

Return the number of non-overlapping occurrences of substring sub in string S[start:end]. Optional arguments start and end are interpreted as in slice notation.

encode(*encoding='utf-8'*, *errors='strict'*)

Encode the string using the codec registered for encoding.

encoding
The encoding in which to encode the string.

errors
The error handling scheme to use for encoding errors. The default is 'strict' meaning that encoding errors raise a UnicodeEncodeError. Other possible values are 'ignore', 'replace' and 'xmlcharrefreplace' as well as any other name registered with codecs.register_error that can handle UnicodeEncodeErrors.

endswith(*suffix*[, *start*[, *end*]]) → bool

Return True if S ends with the specified suffix, False otherwise. With optional start, test S beginning at that position. With optional end, stop comparing S at that position. suffix can also be a tuple of strings to try.

expandtabs(*tabsize=8*)

Return a copy where all tab characters are expanded using spaces.

If tabsize is not given, a tab size of 8 characters is assumed.

find(*sub*[, *start*[, *end*]]) → int

Return the lowest index in S where substring sub is found, such that sub is contained within S[start:end]. Optional arguments start and end are interpreted as in slice notation.

Return -1 on failure.

format(**args*, ***kwargs*) → str

Return a formatted version of S, using substitutions from args and kwargs. The substitutions are identified by braces ('{' and '}').

format_map(*mapping*) → str

Return a formatted version of S, using substitutions from mapping. The substitutions are identified by braces ('{' and '}').

index(*sub*[, *start*[, *end*]]) → int

Return the lowest index in S where substring sub is found, such that sub is contained within S[start:end]. Optional arguments start and end are interpreted as in slice notation.

Raises ValueError when the substring is not found.

property is_gap: bool

Check if the character represents a gap in the sequence.

property is_non_canonical: bool

Check if the character represents a non-canonical amino acid.

property is_not_focus: bool

Check if the character is not in focus.

A character is considered not in focus if it's a gap or a lowercase letter.

isalnum()

Return True if the string is an alpha-numeric string, False otherwise.

A string is alpha-numeric if all characters in the string are alpha-numeric and there is at least one character in the string.

isalpha()

Return True if the string is an alphabetic string, False otherwise.

A string is alphabetic if all characters in the string are alphabetic and there is at least one character in the string.

isascii()

Return True if all characters in the string are ASCII, False otherwise.

ASCII characters have code points in the range U+0000-U+007F. Empty string is ASCII too.

isdecimal()

Return True if the string is a decimal string, False otherwise.

A string is a decimal string if all characters in the string are decimal and there is at least one character in the string.

isdigit()

Return True if the string is a digit string, False otherwise.

A string is a digit string if all characters in the string are digits and there is at least one character in the string.

isidentifier()

Return True if the string is a valid Python identifier, False otherwise.

Call keyword.iskeyword(s) to test whether string s is a reserved identifier, such as "def" or "class".

islower()

Return True if the string is a lowercase string, False otherwise.

A string is lowercase if all cased characters in the string are lowercase and there is at least one cased character in the string.

isnumeric()

Return True if the string is a numeric string, False otherwise.

A string is numeric if all characters in the string are numeric and there is at least one character in the string.

isprintable()

Return True if the string is printable, False otherwise.

A string is printable if all of its characters are considered printable in repr() or if it is empty.

isspace()

Return True if the string is a whitespace string, False otherwise.

A string is whitespace if all characters in the string are whitespace and there is at least one character in the string.

istitle()

Return True if the string is a title-cased string, False otherwise.

In a title-cased string, upper- and title-case characters may only follow uncased characters and lowercase characters only cased ones.

isupper()

Return True if the string is an uppercase string, False otherwise.

A string is uppercase if all cased characters in the string are uppercase and there is at least one cased character in the string.

join(*iterable*, */*)

Concatenate any number of strings.

The string whose method is called is inserted in between each given string. The result is returned as a new string.

Example: '.'.join(['ab', 'pq', 'rs']) -> 'ab.pq.rs'

ljust(*width*, *fillchar=' '*, */*)

Return a left-justified string of length width.

Padding is done using the specified fill character (default is a space).

lower()

Return a copy of the string converted to lowercase.

lstrip(*chars=None*, */*)

Return a copy of the string with leading whitespace removed.

If chars is given and not None, remove characters in chars instead.

static **maketrans**()

Return a translation table usable for str.translate().

If there is only one argument, it must be a dictionary mapping Unicode ordinals (integers) or characters to Unicode ordinals, strings or None. Character keys will be then converted to ordinals. If there are two arguments, they must be strings of equal length, and in the resulting dictionary, each character in x will be mapped to the character at the same position in y. If there is a third argument, it must be a string, whose characters will be mapped to None in the result.

partition(*sep*, */*)

Partition the string into three parts using the given separator.

This will search for the separator in the string. If the separator is found, returns a 3-tuple containing the part before the separator, the separator itself, and the part after it.

If the separator is not found, returns a 3-tuple containing the original string and two empty strings.

removeprefix(*prefix*, */*)

Return a str with the given prefix string removed if present.

If the string starts with the prefix string, return string[len(prefix):]. Otherwise, return a copy of the original string.

removesuffix(*suffix*, */*)

Return a str with the given suffix string removed if present.

If the string ends with the suffix string and that suffix is not empty, return string[:-len(suffix)]. Otherwise, return a copy of the original string.

replace(*old*, *new*, *count=-1*, */*)

Return a copy with all occurrences of substring old replaced by new.

count
Maximum number of occurrences to replace. -1 (the default value) means replace all occurrences.

If the optional argument count is given, only the first count occurrences are replaced.

rfind(*sub*[, *start*[, *end*]]) → int

Return the highest index in S where substring sub is found, such that sub is contained within S[start:end]. Optional arguments start and end are interpreted as in slice notation.

Return -1 on failure.

rindex(*sub*[, *start*[, *end*]]) → int

Return the highest index in S where substring sub is found, such that sub is contained within S[start:end]. Optional arguments start and end are interpreted as in slice notation.

Raises ValueError when the substring is not found.

rjust(*width*, *fillchar=' '*, */*)

Return a right-justified string of length width.

Padding is done using the specified fill character (default is a space).

rpartition(*sep*, */*)

Partition the string into three parts using the given separator.

This will search for the separator in the string, starting at the end. If the separator is found, returns a 3-tuple containing the part before the separator, the separator itself, and the part after it.

If the separator is not found, returns a 3-tuple containing two empty strings and the original string.

rsplit(*sep=None*, *maxsplit=-1*)

Return a list of the words in the string, using sep as the delimiter string.

sep
The delimiter according which to split the string. None (the default value) means split according to any whitespace, and discard empty strings from the result.

maxsplit
Maximum number of splits to do. -1 (the default value) means no limit.

Splits are done starting at the end of the string and working to the front.

rstrip(*chars=None*, */*)

Return a copy of the string with trailing whitespace removed.

If chars is given and not None, remove characters in chars instead.

`split`(*sep=None*, *maxsplit=-1*)

Return a list of the words in the string, using sep as the delimiter string.

sep
The delimiter according which to split the string. None (the default value) means split according to any whitespace, and discard empty strings from the result.

maxsplit
Maximum number of splits to do. -1 (the default value) means no limit.

`splitlines`(*keepends=False*)

Return a list of the lines in the string, breaking at line boundaries.

Line breaks are not included in the resulting list unless keepends is given and true.

`startswith`(*prefix*[, *start*[, *end*]]) → bool

Return True if S starts with the specified prefix, False otherwise. With optional start, test S beginning at that position. With optional end, stop comparing S at that position. prefix can also be a tuple of strings to try.

`strip`(*chars=None*, */*)

Return a copy of the string with leading and trailing whitespace removed.

If chars is given and not None, remove characters in chars instead.

`swapcase`()

Convert uppercase characters to lowercase and lowercase characters to uppercase.

`title`()

Return a version of the string where each word is titlecased.

More specifically, words start with uppercased characters and all remaining cased characters have lower case.

`translate`(*table*, */*)

Replace each character in the string using the given translation table.

table
Translation table, which must be a mapping of Unicode ordinals to Unicode ordinals, strings, or None.

The table must implement lookup/indexing via __getitem__, for instance a dictionary or list. If this operation raises LookupError, the character is left untouched. Characters mapped to None are deleted.

`upper`()

Return a copy of the string converted to uppercase.

`zfill`(*width*, */*)

Pad a numeric string with zeros on the left, to fill a field of the given width.

The string is never truncated.

class `aide_predict.utils.data_structures.sequences.`**`ProteinSequence`**(*seq: str*, *id: str | None = None*, *structure: str | ProteinStructure | None = None*, *msa: ProteinSequences | None = None*)

Bases: `str`

Represents a protein sequence.

This class inherits from UserString and provides additional methods and properties for analyzing and manipulating protein sequences.

`align`(*other:* ProteinSequence) → *ProteinSequence*

Align this sequence with another using global pairwise alignment.

Parameters
`other` (`ProteinSequence`) – The sequence to align with.

Returns
The aligned sequence.

Return type
ProteinSequence

`property as_array: ndarray`

Convert the sequence to a numpy array of characters.

`property base_length: int`

Get the length of the sequence excluding gaps.

`capitalize`()

Return a capitalized version of the string.

More specifically, make the first character have upper case and the rest lower case.

`casefold`()

Return a version of the string suitable for caseless comparisons.

`center`(*width*, *fillchar=' '*, */*)

Return a centered string of length width.

Padding is done using the specified fill character (default is a space).

`count`(*sub*[, *start*[, *end*]]) → int

Return the number of non-overlapping occurrences of substring sub in string S[start:end]. Optional arguments start and end are interpreted as in slice notation.

`encode`(*encoding='utf-8'*, *errors='strict'*)

Encode the string using the codec registered for encoding.

encoding
The encoding in which to encode the string.

errors
The error handling scheme to use for encoding errors. The default is 'strict' meaning that encoding errors raise a UnicodeEncodeError. Other possible values are 'ignore', 'replace' and 'xmlcharrefreplace' as well as any other name registered with codecs.register_error that can handle UnicodeEncodeErrors.

`endswith`(*suffix*[, *start*[, *end*]]) → bool

Return True if S ends with the specified suffix, False otherwise. With optional start, test S beginning at that position. With optional end, stop comparing S at that position. suffix can also be a tuple of strings to try.

`expandtabs`(*tabsize=8*)

Return a copy where all tab characters are expanded using spaces.

If tabsize is not given, a tab size of 8 characters is assumed.

find(*sub*[, *start*[, *end*]]) → int

Return the lowest index in S where substring sub is found, such that sub is contained within S[start:end]. Optional arguments start and end are interpreted as in slice notation.

Return -1 on failure.

format(**args*, ***kwargs*) → str

Return a formatted version of S, using substitutions from args and kwargs. The substitutions are identified by braces ('{' and '}').

format_map(*mapping*) → str

Return a formatted version of S, using substitutions from mapping. The substitutions are identified by braces ('{' and '}').

classmethod from_a3m(*a3m_file: str*, *inserts: str = 'first'*) → *ProteinSequence*

Create a ProteinSequence from an A3M file, assuming the first sequence is the query.

classmethod from_fasta(*fasta_file: str*) → *ProteinSequence*

Create a ProteinSequence from a FASTA file, assuming the first sequence is the query.

classmethod from_pdb(*pdb_file: str*, *chain: str = 'A'*, *id: str | None = None*) → *ProteinSequence*

Create a ProteinSequence from a PDB file.

This method extracts the amino acid sequence from the PDB file and creates a ProteinSequence object with the associated structure.

Parameters

- **pdb_file** (*str*) – Path to the PDB file.
- **chain** (*str*) – Chain identifier to extract sequence from. Defaults to 'A'.
- **id** (*Optional[str]*) – Identifier for the sequence. If None, uses the PDB filename.

Returns

A new ProteinSequence object with the extracted sequence and structure.

Return type

ProteinSequence

Raises

- **FileNotFoundError** – If the PDB file does not exist.
- **ValueError** – If the specified chain is not found in the PDB file.

Example

```
>>> seq = ProteinSequence.from_pdb("1abc.pdb", chain='A', id='my_protein')
>>> print(seq)
'MAEGEITTFTALTEKFNLPPGNYKKPKLLYCSNG...'
>>> print(seq.structure)
ProteinStructure(pdb_file='1abc.pdb', chain='A')
```

get_mutations(*other: str* | ProteinSequence) → List[str]

Find mutations between this sequence and another.

Parameters

other (*Union[str, ProteinSequence]*) – The sequence to compare against.

Returns
A list of mutations in the format 'A123B' where A is the original character, 123 is the position, and B is the new character.

Return type
List[str]

`get_protein_character`(*position: int*) → *ProteinCharacter*

Get the ProteinCharacter at the specified position.

Parameters
position (*int*) – The position to get the character from.

Returns
The character at the specified position.

Return type
ProteinCharacter

`property has_gaps: bool`

Check if the sequence contains any gaps.

`property has_msa: bool`

Check if the sequence has an associated MSA.

`property has_non_canonical: bool`

Check if the sequence contains any non-canonical amino acids.

`property has_structure: bool`

Check if the sequence has an associated structure.

`property id: str | None`

Get the identifier of the sequence.

`index`(*sub*[, *start*[, *end*]]) → int

Return the lowest index in S where substring sub is found, such that sub is contained within S[start:end]. Optional arguments start and end are interpreted as in slice notation.

Raises ValueError when the substring is not found.

`property is_in_msa: bool`

Check if the sequence is part of an MSA.

`isalnum`()

Return True if the string is an alpha-numeric string, False otherwise.

A string is alpha-numeric if all characters in the string are alpha-numeric and there is at least one character in the string.

`isalpha`()

Return True if the string is an alphabetic string, False otherwise.

A string is alphabetic if all characters in the string are alphabetic and there is at least one character in the string.

`isascii`()

Return True if all characters in the string are ASCII, False otherwise.

ASCII characters have code points in the range U+0000-U+007F. Empty string is ASCII too.

`isdecimal()`

Return True if the string is a decimal string, False otherwise.

A string is a decimal string if all characters in the string are decimal and there is at least one character in the string.

`isdigit()`

Return True if the string is a digit string, False otherwise.

A string is a digit string if all characters in the string are digits and there is at least one character in the string.

`isidentifier()`

Return True if the string is a valid Python identifier, False otherwise.

Call keyword.iskeyword(s) to test whether string s is a reserved identifier, such as "def" or "class".

`islower()`

Return True if the string is a lowercase string, False otherwise.

A string is lowercase if all cased characters in the string are lowercase and there is at least one cased character in the string.

`isnumeric()`

Return True if the string is a numeric string, False otherwise.

A string is numeric if all characters in the string are numeric and there is at least one character in the string.

`isprintable()`

Return True if the string is printable, False otherwise.

A string is printable if all of its characters are considered printable in repr() or if it is empty.

`isspace()`

Return True if the string is a whitespace string, False otherwise.

A string is whitespace if all characters in the string are whitespace and there is at least one character in the string.

`istitle()`

Return True if the string is a title-cased string, False otherwise.

In a title-cased string, upper- and title-case characters may only follow uncased characters and lowercase characters only cased ones.

`isupper()`

Return True if the string is an uppercase string, False otherwise.

A string is uppercase if all cased characters in the string are uppercase and there is at least one cased character in the string.

`iter_protein_characters()` → Iterator[*ProteinCharacter*]

Iterate over the ProteinCharacters in the sequence.

Returns

An iterator over the ProteinCharacters.

Return type

Iterator[*ProteinCharacter*]

join(*iterable*, /)

Concatenate any number of strings.

The string whose method is called is inserted in between each given string. The result is returned as a new string.

Example: '.'.join(['ab', 'pq', 'rs']) -> 'ab.pq.rs'

ljust(*width*, *fillchar=' '*, /)

Return a left-justified string of length width.

Padding is done using the specified fill character (default is a space).

lower()

Return a copy of the string converted to lowercase.

lstrip(*chars=None*, /)

Return a copy of the string with leading whitespace removed.

If chars is given and not None, remove characters in chars instead.

static maketrans()

Return a translation table usable for str.translate().

If there is only one argument, it must be a dictionary mapping Unicode ordinals (integers) or characters to Unicode ordinals, strings or None. Character keys will be then converted to ordinals. If there are two arguments, they must be strings of equal length, and in the resulting dictionary, each character in x will be mapped to the character at the same position in y. If there is a third argument, it must be a string, whose characters will be mapped to None in the result.

property msa: *ProteinSequences* | None

Get the MSA (multiple sequence alignment) of the sequence.

property msa_same_width: bool

Check if the MSA has the same width as the sequence.

mutate(*mutations: str | List[str]*, *one_indexed: bool = True*)

Create a new ProteinSequence with mutations applied.

Params

mutations: Union[str, List[str]]
A single mutation in the format 'A123B' or a list of mutations.

one_indexed: bool
If True, positions are one-indexed. If False, positions are zero-indexed.

mutated_positions(*other: str | ProteinSequence*) → List[int]

Find positions where this sequence differs from another.

Parameters
other (*Union[str, ProteinSequence]*) – The sequence to compare against.

Returns
A list of positions where the sequences differ.

Return type
List[int]

property num_gaps: int

Get the number of gaps in the sequence.

partition(*sep*, */*)

Partition the string into three parts using the given separator.

This will search for the separator in the string. If the separator is found, returns a 3-tuple containing the part before the separator, the separator itself, and the part after it.

If the separator is not found, returns a 3-tuple containing the original string and two empty strings.

removeprefix(*prefix*, */*)

Return a str with the given prefix string removed if present.

If the string starts with the prefix string, return string[len(prefix):]. Otherwise, return a copy of the original string.

removesuffix(*suffix*, */*)

Return a str with the given suffix string removed if present.

If the string ends with the suffix string and that suffix is not empty, return string[:-len(suffix)]. Otherwise, return a copy of the original string.

replace(*old*, *new*, *count=-1*, */*)

Return a copy with all occurrences of substring old replaced by new.

count
Maximum number of occurrences to replace. -1 (the default value) means replace all occurrences.

If the optional argument count is given, only the first count occurrences are replaced.

rfind(*sub*[, *start*[, *end*]]) → int

Return the highest index in S where substring sub is found, such that sub is contained within S[start:end]. Optional arguments start and end are interpreted as in slice notation.

Return -1 on failure.

rindex(*sub*[, *start*[, *end*]]) → int

Return the highest index in S where substring sub is found, such that sub is contained within S[start:end]. Optional arguments start and end are interpreted as in slice notation.

Raises ValueError when the substring is not found.

rjust(*width*, *fillchar=' '*, */*)

Return a right-justified string of length width.

Padding is done using the specified fill character (default is a space).

rpartition(*sep*, */*)

Partition the string into three parts using the given separator.

This will search for the separator in the string, starting at the end. If the separator is found, returns a 3-tuple containing the part before the separator, the separator itself, and the part after it.

If the separator is not found, returns a 3-tuple containing two empty strings and the original string.

rsplit(*sep=None*, *maxsplit=-1*)

Return a list of the words in the string, using sep as the delimiter string.

sep
The delimiter according which to split the string. None (the default value) means split according to any whitespace, and discard empty strings from the result.

maxsplit
Maximum number of splits to do. -1 (the default value) means no limit.

Splits are done starting at the end of the string and working to the front.

`rstrip`(*chars=None*, */*)

Return a copy of the string with trailing whitespace removed.

If chars is given and not None, remove characters in chars instead.

`saturation_mutagenesis`(*positions: List[int] | None = None*, *include_wt: bool = False*) → List[*ProteinSequence*]

Perform saturation mutagenesis at the specified positions.

Parameters
`positions` (*List[int]*) – The positions to mutate.

Returns
A list of mutated sequences.

Return type
ProteinSequences

`slice_as_protein_sequence`(*start: int*, *end: int*) → *ProteinSequence*

Create a new ProteinSequence from a slice of this sequence.

Parameters

- **start** (*int*) – The start position of the slice.
- **end** (*int*) – The end position of the slice.

Returns
A new ProteinSequence containing the specified slice.

Return type
ProteinSequence

`split`(*sep=None*, *maxsplit=-1*)

Return a list of the words in the string, using sep as the delimiter string.

sep
The delimiter according which to split the string. None (the default value) means split according to any whitespace, and discard empty strings from the result.

maxsplit
Maximum number of splits to do. -1 (the default value) means no limit.

`splitlines`(*keepends=False*)

Return a list of the lines in the string, breaking at line boundaries.

Line breaks are not included in the resulting list unless keepends is given and true.

`startswith`(*prefix*[, *start*[, *end*]]) → bool

Return True if S starts with the specified prefix, False otherwise. With optional start, test S beginning at that position. With optional end, stop comparing S at that position. prefix can also be a tuple of strings to try.

strip(*chars=None*, */*)

Return a copy of the string with leading and trailing whitespace removed.

If chars is given and not None, remove characters in chars instead.

property structure: str | None

Get the structure of the sequence.

swapcase()

Convert uppercase characters to lowercase and lowercase characters to uppercase.

title()

Return a version of the string where each word is titlecased.

More specifically, words start with uppercased characters and all remaining cased characters have lower case.

translate(*table*, */*)

Replace each character in the string using the given translation table.

table
Translation table, which must be a mapping of Unicode ordinals to Unicode ordinals, strings, or None.

The table must implement lookup/indexing via __getitem__, for instance a dictionary or list. If this operation raises LookupError, the character is left untouched. Characters mapped to None are deleted.

upper() → *ProteinSequence*

Return a new ProteinSequence with all characters converted to uppercase.

with_no_gaps() → *ProteinSequence*

Return a new ProteinSequence with all gaps removed.

zfill(*width*, */*)

Pad a numeric string with zeros on the left, to fill a field of the given width.

The string is never truncated.

class aide_predict.utils.data_structures.sequences.**ProteinSequences**(*sequences: List[ProteinSequence], weights: ndarray | None = None*)

Bases: `UserList`

A collection of ProteinSequence objects with additional functionality.

Variables

- **aligned** (*bool*) – True if all sequences have the same length, False otherwise.
- **fixed_length** (*bool*) – True if all sequences have the same base length, False otherwise.
- **width** (*Optional[int]*) – The length of the sequences if aligned, None otherwise.
- **has_gaps** (*bool*) – True if any sequence has gaps, False otherwise.
- **mutated_positions** (*Optional[List[int]]*) – List of mutated positions if aligned, None otherwise.

to_dict()

Convert ProteinSequences to a dictionary.

to_fasta()

Write sequences to a FASTA file.

from_fasta()

Create a ProteinSequences object from a FASTA file.

__init__(*sequences: List[*ProteinSequence*]*, *weights: ndarray | None = None*)

Initialize a ProteinSequences object.

Parameters

- **sequences** (*List[ProteinSequence]*) – A list of ProteinSequence objects.
- **weights** (*Optional[np.ndarray]*) – Weights for each sequence. If None, initialized as ones.

align_all(*output_fasta: str | None = None*) → *ProteinSequences* | *ProteinSequencesOnFile*

Align the sequences within this ProteinSequences object using MAFFT.

Parameters

output_fasta (*Optional[str]*) – Path to save the alignment. If None, a temporary file is used.

Returns

The aligned sequences, either in memory or on file depending on output_fasta.

Return type

Union[*ProteinSequences*, *ProteinSequencesOnFile*]

Raises

- **ValueError** – If the sequences already contain gaps.
- **RuntimeError** – If MAFFT alignment fails.
- **FileNotFoundError** – If MAFFT is not installed or not in PATH.

align_to(*existing_alignment:* ProteinSequences | ProteinSequencesOnFile, *realign: bool = False*, *return_only_new: bool = False*, *output_fasta: str | None = None*) → *ProteinSequences* | *ProteinSequencesOnFile*

Align this ProteinSequences object to an existing alignment using MAFFT.

Parameters

- **existing_alignment** (*Union[ProteinSequences, ProteinSequencesOnFile]*) – The existing alignment to align to.
- **realign** (*bool*) – If True, realign all sequences from scratch. If False, add new sequences to existing alignment. return_only_new (bool): If True, return only the newly aligned sequences. If False, return all sequences.
- **output_fasta** (*Optional[str]*) – Path to save the alignment. If None, a temporary file is used.

Returns

The aligned sequences, either in memory or on file depending on output_fasta.

Return type

Union[*ProteinSequences*, *ProteinSequencesOnFile*]

Raises

- **ValueError** – If the sequences already contain gaps or if the existing alignment is not aligned.

- **RuntimeError** – If MAFFT alignment fails.
- **FileNotFoundError** – If MAFFT is not installed or not in PATH.

property aligned: bool

Check if all sequences are of equal length (including gaps).

Returns
True if all sequences have the same length, False otherwise.

Return type
bool

append(*item*)

S.append(value) – append value to the end of the sequence

apply_alignment_mapping(*mapping: Dict[str, List[int | None]]*) → *ProteinSequences*

Apply an alignment mapping to the current sequences.

Parameters
mapping (*Dict[str, List[Optional[int]]]*) – The alignment mapping to apply.

Returns
A new ProteinSequences object with aligned sequences.

Return type
ProteinSequences

Raises
ValueError – If a sequence ID or hash is not found in the mapping or if the mapping is invalid.

as_array() → ndarray

Convert the sequence to a numpy array of characters.

clear() → None -- remove all items from S

copy()

count(*value*) → integer -- return number of occurrences of value

extend(*other*)

S.extend(iterable) – extend sequence by appending elements from the iterable

property fixed_length: bool

Check if all contained sequences have the same base length (excluding gaps).

Returns
True if all sequences have the same base length, False otherwise.

Return type
bool

classmethod from_a3m(*input_path: str*, *inserts: str = 'first'*) → *ProteinSequences*

Create a ProteinSequences object from an A3M file.

Parameters
input_path (*str*) – The path to the input A3M file.

Returns
A new ProteinSequences object containing the sequences from the A3M file.

Return type
ProteinSequences

classmethod from_csv(*filepath: str*, *id_col: str | None = None*, *seq_col: str | None = None*, *label_cols: List[str] | str | None = None*, ***kwargs*) → *ProteinSequences* | Tuple[*ProteinSequences*, ndarray]

Create a ProteinSequences object from a CSV file.

Parameters

- **filepath** (*str*) – Path to the CSV file.
- **id_col** (*Optional[str]*) – Name of column containing sequence IDs. If None, sequences will be assigned numeric IDs.
- **seq_col** (*Optional[str]*) – Name of column containing sequences. If None, uses first column.
- **label_cols** (*Optional[Union[str, List[str]]]*) – Name(s) of columns containing labels to return.
- ****kwargs** – Additional arguments passed to pandas.read_csv().

Returns

- If label_cols is None: ProteinSequences object
- If label_cols is provided: Tuple of (ProteinSequences, labels array)

Return type
Union[*ProteinSequences*, Tuple[*ProteinSequences*, np.ndarray]]

Raises

- **ValueError** – If specified columns are not found in the CSV file.
- **ValueError** – If any sequence contains invalid characters.

classmethod from_df(*df: pd.DataFrame*, *id_col: str | None = None*, *seq_col: str | None = None*, *label_cols: List[str] | str | None = None*) → *ProteinSequences* | Tuple[*ProteinSequences*, ndarray]

Create a ProteinSequences object from a pandas DataFrame.

Parameters

- **df** (*pd.DataFrame*) – Input DataFrame containing sequences.
- **id_col** (*Optional[str]*) – Name of column containing sequence IDs. If None, sequences will be assigned numeric IDs.
- **seq_col** (*Optional[str]*) – Name of column containing sequences. If None, uses first column.
- **label_cols** (*Optional[Union[str, List[str]]]*) – Name(s) of columns containing labels to return.

Returns

- If label_cols is None: ProteinSequences object
- If label_cols is provided: Tuple of (ProteinSequences, labels array)

Return type
Union[*ProteinSequences*, Tuple[*ProteinSequences*, np.ndarray]]

Raises

- **ValueError** – If specified columns are not found in the DataFrame.
- **ValueError** – If any sequence contains invalid characters.

classmethod from_dict(*sequences: Dict[str, str]*) → *ProteinSequences*

Create a ProteinSequences object from a dictionary.

Parameters
sequences (*Dict[str, str]*) – A dictionary with sequence IDs as keys and sequences as values.

Returns
A new ProteinSequences object containing the sequences from the dictionary.

Return type
ProteinSequences

classmethod from_fasta(*input_path: str*) → *ProteinSequences*

Create a ProteinSequences object from a FASTA file.

Parameters
input_path (*str*) – The path to the input FASTA file.

Returns
A new ProteinSequences object containing the sequences from the FASTA file.

Return type
ProteinSequences

classmethod from_list(*sequences: List[str]*) → *ProteinSequences*

Create a ProteinSequences object from a list of sequences.

Parameters
sequences (*List[str]*) – A list of protein sequences.

Returns
A new ProteinSequences object containing the sequences from the list.

Return type
ProteinSequences

get_alignment_mapping() → Dict[str, List[int | None]]

Create a mapping of original sequence positions to aligned positions for each sequence.

Returns
A dictionary where keys are sequence IDs or hashes and values are lists of integers. Each integer represents the position in the aligned sequence corresponding to the original sequence position. E.g., [0,1,2,5,6,7] indicates that there is a gap between amino acid 2 and 3, and 3 is in position 5 in the aligned sequence.

Return type
Dict[str, List[Optional[int]]]

Raises
ValueError – If the sequences are not aligned.

get_id_mapping() → Dict[str, int]

Create a mapping of sequence IDs to indices.

Returns
A dictionary where keys are sequence IDs and values are indices.

Return type
Dict[str, int]

property has_gaps: bool

Check if any sequences have gaps.

Returns
True if any sequence has gaps, False otherwise.

Return type
bool

has_lower() → bool

Check if any sequence contains lowercase characters.

property id_mapping: Dict[str, int]

property ids: List[str]

Get a list of sequence IDs.

index(*value*[, *start*[, *stop*]]) → integer -- return first index of value.

Raises ValueError if the value is not present.

Supporting start and stop arguments is optional, but recommended.

insert(*i*, *item*)

S.insert(index, value) – insert value before index

iter_batches(*batch_size: int*) → Iterable[*ProteinSequences*]

Iterate over batches of sequences.

Parameters
batch_size (*int*) – The size of each batch.

Yields
ProteinSequences – A batch of sequences.

msa_process(*focus_seq_id: str | None = None*, ***kwargs*) → *ProteinSequence*

Align this sequence with another using global pairwise alignment.

Kwargs:
**kwargs: Additional arguments to pass to MSAprocessing

Returns
The aligned sequence.

Return type
ProteinSequence

property mutated_positions: List[int] | None

List columns that have more than one character, assuming sequences are aligned.

Returns
List of mutated positions if aligned, None otherwise.

Return type
Optional[List[int]]

pop([*index*]) → item -- remove and return item at index (default last).

Raise IndexError if list is empty or index is out of range.

`remove`(*item*)

S.remove(value) – remove first occurrence of value. Raise ValueError if the value is not present.

`reverse`()

S.reverse() – reverse *IN PLACE*

`sample`(*n: int*, *replace: bool = False*, *keep_first: bool = False*, *seed: int | None = None*) → *ProteinSequences*

Sample n sequences from the ProteinSequences object.

Parameters

- **n** (*int*) – Number of sequences to sample.
- **replace** (*bool*) – Whether to sample with replacement. Default is False.

Returns

A new ProteinSequences object containing the sampled sequences.

Return type

ProteinSequences

Raises

ValueError – If n is greater than the number of sequences and replace is False.

`sort`(**args*, ***kwds*)

`to_dict`() → Dict[str, str]

Convert ProteinSequences to a dictionary.

Returns

A dictionary with sequence IDs as keys and sequences as values.

Return type

Dict[str, str]

`to_fasta`(*output_path: str*)

Write sequences to a FASTA file.

Parameters

output_path (*str*) – The path to the output FASTA file.

`to_on_file`(*output_path: str*) → None

Write sequences to a FASTA file.

Parameters

output_path (*str*) – The path to the output FASTA file.

`upper`() → *ProteinSequences*

Return a new ProteinSequences with all sequences converted to uppercase.

property weights: ndarray

Get the weights for each sequence.

property width: int | None

Get the length of the sequences if aligned.

Returns

The length of the sequences if aligned, None otherwise.

Return type

Optional[int]

with_no_gaps() → *ProteinSequences*

Return a new ProteinSequences with all gaps removed.

class aide_predict.utils.data_structures.sequences.**ProteinSequencesOnFile**(*file_path: str*, *weights: ndarray | None = None*)

Bases: `ProteinSequences`

A memory-efficient representation of protein sequences stored in a FASTA file.

This class maintains the same API as ProteinSequences but avoids loading all sequences into memory at once. It creates an index of the FASTA file for efficient access to individual sequences and precomputes some global properties for quick access.

Variables

- **aligned** (*bool*) – True if all sequences have the same length, False otherwise.
- **fixed_length** (*bool*) – True if all sequences have the same base length, False otherwise.
- **width** (*Optional[int]*) – The length of the sequences if aligned, None otherwise.
- **has_gaps** (*bool*) – True if any sequence has gaps, False otherwise.
- **mutated_positions** (*Optional[List[int]]*) – List of mutated positions if aligned, None otherwise.

to_dict()

Convert ProteinSequences to a dictionary.

to_fasta()

Write sequences to a FASTA file.

from_fasta()

Create a ProteinSequences object from a FASTA file.

__init__(*file_path: str*, *weights: ndarray | None = None*)

Initialize a ProteinSequencesOnFile object.

Parameters

- **file_path** (*str*) – Path to the FASTA file containing protein sequences.
- **weights** (*Optional[np.ndarray]*) – Weights for each sequence. If None, initialized as ones.

align_all(*output_fasta: str | None = None*) → *ProteinSequences* | *ProteinSequencesOnFile*

Align the sequences within this ProteinSequences object using MAFFT.

Parameters

output_fasta (*Optional[str]*) – Path to save the alignment. If None, a temporary file is used.

Returns

The aligned sequences, either in memory or on file depending on output_fasta.

Return type

Union[*ProteinSequences*, *ProteinSequencesOnFile*]

Raises

- **ValueError** – If the sequences already contain gaps.
- **RuntimeError** – If MAFFT alignment fails.

- **FileNotFoundError** – If MAFFT is not installed or not in PATH.

align_to(*existing_alignment: ProteinSequences | ProteinSequencesOnFile, realign: bool = False, return_only_new: bool = False, output_fasta: str | None = None*) → *ProteinSequences | ProteinSequencesOnFile*

Align this ProteinSequences object to an existing alignment using MAFFT.

Parameters

- **existing_alignment** (*Union[ProteinSequences, ProteinSequencesOnFile]*) – The existing alignment to align to.
- **realign** (*bool*) – If True, realign all sequences from scratch. If False, add new sequences to existing alignment. return_only_new (bool): If True, return only the newly aligned sequences. If False, return all sequences.
- **output_fasta** (*Optional[str]*) – Path to save the alignment. If None, a temporary file is used.

Returns
The aligned sequences, either in memory or on file depending on output_fasta.

Return type
Union[*ProteinSequences*, *ProteinSequencesOnFile*]

Raises

- **ValueError** – If the sequences already contain gaps or if the existing alignment is not aligned.
- **RuntimeError** – If MAFFT alignment fails.
- **FileNotFoundError** – If MAFFT is not installed or not in PATH.

property aligned: bool

Check if all sequences are of equal length (including gaps).

Returns
True if all sequences have the same length, False otherwise.

Return type
bool

append(*item*)

S.append(value) – append value to the end of the sequence

apply_alignment_mapping(*mapping: Dict[str, List[int | None]]*) → *ProteinSequences*

Apply an alignment mapping to the current sequences.

Parameters
mapping (*Dict[str, List[Optional[int]]]*) – The alignment mapping to apply.

Returns
A new ProteinSequences object with aligned sequences.

Return type
ProteinSequences

Raises
ValueError – If a sequence ID or hash is not found in the mapping or if the mapping is invalid.

as_array() → ndarray

Convert the sequence to a numpy array of characters.

clear() → None -- remove all items from S

copy()

count(*value*) → integer -- return number of occurrences of value

extend(*other*)

S.extend(iterable) – extend sequence by appending elements from the iterable

property fixed_length: bool

Check if all contained sequences have the same base length (excluding gaps).

Returns
True if all sequences have the same base length, False otherwise.

Return type
bool

classmethod from_a3m(*input_path: str*, *inserts: str = 'first'*) → *ProteinSequences*

Create a ProteinSequences object from an A3M file.

Parameters
input_path (*str*) – The path to the input A3M file.

Returns
A new ProteinSequences object containing the sequences from the A3M file.

Return type
ProteinSequences

classmethod from_csv(*filepath: str*, *id_col: str | None = None*, *seq_col: str | None = None*, *label_cols: List[str] | str | None = None*, ***kwargs*) → *ProteinSequences* | Tuple[*ProteinSequences*, ndarray]

Create a ProteinSequences object from a CSV file.

Parameters

- **filepath** (*str*) – Path to the CSV file.
- **id_col** (*Optional[str]*) – Name of column containing sequence IDs. If None, sequences will be assigned numeric IDs.
- **seq_col** (*Optional[str]*) – Name of column containing sequences. If None, uses first column.
- **label_cols** (*Optional[Union[str, List[str]]]*) – Name(s) of columns containing labels to return.
- ****kwargs** – Additional arguments passed to pandas.read_csv().

Returns

- If label_cols is None: ProteinSequences object
- If label_cols is provided: Tuple of (ProteinSequences, labels array)

Return type
Union[*ProteinSequences*, Tuple[*ProteinSequences*, np.ndarray]]

Raises

- **ValueError** – If specified columns are not found in the CSV file.
- **ValueError** – If any sequence contains invalid characters.

classmethod from_df(*df: pd.DataFrame*, *id_col: str | None = None*, *seq_col: str | None = None*, *label_cols: List[str] | str | None = None*) → *ProteinSequences* | Tuple[*ProteinSequences*, ndarray]

Create a ProteinSequences object from a pandas DataFrame.

Parameters

- **df** (*pd.DataFrame*) – Input DataFrame containing sequences.
- **id_col** (*Optional[str]*) – Name of column containing sequence IDs. If None, sequences will be assigned numeric IDs.
- **seq_col** (*Optional[str]*) – Name of column containing sequences. If None, uses first column.
- **label_cols** (*Optional[Union[str, List[str]]]*) – Name(s) of columns containing labels to return.

Returns

- If label_cols is None: ProteinSequences object
- If label_cols is provided: Tuple of (ProteinSequences, labels array)

Return type

Union[*ProteinSequences*, Tuple[*ProteinSequences*, np.ndarray]]

Raises

- **ValueError** – If specified columns are not found in the DataFrame.
- **ValueError** – If any sequence contains invalid characters.

classmethod from_dict(*sequences: Dict[str, str]*) → *ProteinSequences*

Create a ProteinSequences object from a dictionary.

Parameters

sequences (*Dict[str, str]*) – A dictionary with sequence IDs as keys and sequences as values.

Returns

A new ProteinSequences object containing the sequences from the dictionary.

Return type

ProteinSequences

classmethod from_fasta(*input_path: str*) → *ProteinSequencesOnFile*

Create a ProteinSequencesOnFile object from a FASTA file.

Parameters

input_path (*str*) – The path to the input FASTA file.

Returns

A new ProteinSequencesOnFile object.

Return type

ProteinSequencesOnFile

classmethod from_list(*sequences: List[str]*) → *ProteinSequences*

Create a ProteinSequences object from a list of sequences.

Parameters
sequences (*List[str]*) – A list of protein sequences.

Returns
A new ProteinSequences object containing the sequences from the list.

Return type
ProteinSequences

get_alignment_mapping() → Dict[str, List[int | None]]

Create a mapping of original sequence positions to aligned positions for each sequence.

Returns
A dictionary where keys are sequence IDs or hashes and values are lists of integers. Each integer represents the position in the aligned sequence corresponding to the original sequence position. E.g., [0,1,2,5,6,7] indicates that there is a gap between amino acid 2 and 3, and 3 is in position 5 in the aligned sequence.

Return type
Dict[str, List[Optional[int]]]

Raises
ValueError – If the sequences are not aligned.

get_id_mapping() → Dict[str, int]

Create a mapping of sequence IDs to indices.

Returns
A dictionary where keys are sequence IDs and values are indices.

Return type
Dict[str, int]

property has_gaps: bool

Check if any sequences have gaps.

Returns
True if any sequence has gaps, False otherwise.

Return type
bool

has_lower() → bool

Check if any sequence contains lowercase characters.

property id_mapping: Dict[str, int]

property ids: List[str]

Get a list of sequence IDs.

index(*value*[, *start*[, *stop*]]) → integer -- return first index of value.

Raises ValueError if the value is not present.

Supporting start and stop arguments is optional, but recommended.

insert(*i*, *item*)

S.insert(index, value) – insert value before index

iter_batches(*batch_size: int*) → Iterable[*ProteinSequences*]

Iterate over batches of sequences.

Parameters
batch_size (*int*) – The size of each batch.

Yields
ProteinSequences – A batch of sequences.

msa_process(*focus_seq_id: str | None = None*, ***kwargs*) → *ProteinSequence*

Align this sequence with another using global pairwise alignment.

Kwargs:
**kwargs: Additional arguments to pass to MSAprocessing

Returns
The aligned sequence.

Return type
ProteinSequence

property mutated_positions: List[int] | None

List columns that have more than one character, assuming sequences are aligned.

Returns
List of mutated positions if aligned, None otherwise.

Return type
Optional[List[int]]

pop([*index*]) → item -- remove and return item at index (default last).

Raise IndexError if list is empty or index is out of range.

remove(*item*)

S.remove(value) – remove first occurrence of value. Raise ValueError if the value is not present.

reverse()

S.reverse() – reverse *IN PLACE*

sample(*n: int*, *replace: bool = False*, *keep_first: bool = False*, *seed: int | None = None*) → *ProteinSequences*

Sample n sequences from the ProteinSequences object.

Parameters
- **n** (*int*) – Number of sequences to sample.
- **replace** (*bool*) – Whether to sample with replacement. Default is False.

Returns
A new ProteinSequences object containing the sampled sequences.

Return type
ProteinSequences

Raises
ValueError – If n is greater than the number of sequences and replace is False.

sort(**args*, ***kwds*)

`to_dict()` → Dict[str, str]

Convert sequences to a dictionary.

Returns
A dictionary with sequence IDs as keys and sequences as values.

Return type
Dict[str, str]

`to_fasta(`*output_path: str*`)` → None

Write sequences to a FASTA file.

Parameters
output_path (*str*) – The path to the output FASTA file.

`to_memory()` → *ProteinSequences*

Load all sequences into memory as a ProteinSequences object.

Returns
A new ProteinSequences object containing all sequences.

Return type
ProteinSequences

`to_on_file(`*output_path: str*`)` → None

Write sequences to a FASTA file.

Parameters
output_path (*str*) – The path to the output FASTA file.

`upper()` → *ProteinSequences*

Return a new ProteinSequences with all sequences converted to uppercase.

property weights: ndarray

Get the weights for each sequence.

property width: int | None

Get the length of the sequences if aligned.

Returns
The length of the sequences if aligned, None otherwise.

Return type
Optional[int]

`with_no_gaps()` → *ProteinSequences*

Return a new ProteinSequences with all gaps removed.

aide_predict.utils.data_structures.structures module

- Author: Evan Komp
- Created: 7/10/2024
- Company: National Renewable Energy Lab, Bioeneergy Science and Technology
- License: MIT

class aide_predict.utils.data_structures.structures.**ProteinStructure**(*pdb_file: str*, *chain: str = 'A'*, *plddt_file: str | None = None*)

Bases: object

chain: str = 'A'

classmethod from_af2_folder(*folder_path: str*, *chain: str = 'A'*) → *ProteinStructure*

Create a ProteinStructure object from an AlphaFold2 prediction folder.

This method prioritizes the top-ranked relaxed structure. If no relaxed structures are available, it selects the top-ranked unrelaxed structure.

Parameters

- **folder_path** (*str*) – Path to the folder containing AlphaFold2 predictions.
- **chain** (*str*) – Chain identifier (default is 'A').

Returns
A new ProteinStructure object.

Return type
ProteinStructure

Raises
FileNotFoundError – If no suitable PDB file is found in the folder.

get_chain() → <module 'Bio.PDB.Chain' from '/Users/ekomp/miniconda3/envs/aidep/lib/python3.9/site-packages/Bio/PDB/Chain.py'>

Load and return the specified chain.

Returns
The specified protein chain.

Return type
Chain

get_dssp() → Dict[str, str]

Get the DSSP secondary structure assignments.

Returns
Dictionary of DSSP assignments.

Return type
Dict[str, str]

get_plddt() → ndarray | None

Get the pLDDT scores if available.

Returns
Array of pLDDT scores or None if not available.

Return type
Optional[np.ndarray]

get_residue_positions() → List[int]

Get the residue positions present in the structure.

Returns
List of residue positions.

Return type
List[int]

get_sequence() → str

Get the amino acid sequence from the PDB file.

Returns
The amino acid sequence.

Return type
str

get_structure() → <module 'Bio.PDB.Structure' from '/Users/ekomp/miniconda3/envs/aidep/lib/python3.9/site-packages/Bio/PDB/Structure.py'>

Load and return the complete structure.

Returns
The complete protein structure.

Return type
Structure

pdb_file: str

plddt_file: str | None = None

validate_sequence(*protein_sequence: str*) → bool

Validate if the given sequence matches the structure's sequence.

Parameters
protein_sequence (*str*) – The sequence to validate.

Returns
True if the sequences match, False otherwise.

Return type
bool

class aide_predict.utils.data_structures.structures.**StructureMapper**(*structure_folder: str*)

Bases: `object`

A class for mapping protein structures to sequences based on files in a given folder.

This class scans a specified folder for PDB files and AlphaFold2 prediction folders, creates ProteinStructure objects, and can assign these structures to ProteinSequence or ProteinSequences objects based on their IDs.

Variables

- **structure_folder** (*str*) – The path to the folder containing structure files.
- **structure_map** (*Dict[str, ProteinStructure]*) – A dictionary mapping protein IDs to ProteinStructure objects.

__init__(*structure_folder: str*)

Initialize the StructureMapper with a folder containing structure files.

Parameters
structure_folder (*str*) – The path to the folder containing structure files.

assign_structures(*sequences: ProteinSequence | ProteinSequences*) → *ProteinSequence | ProteinSequences*

Assign structures to the given protein sequence(s).

This method attempts to assign a structure to each protein sequence based on its ID. If a matching structure is found in the structure_map, it is assigned to the sequence.

Parameters
`sequences` (*Union['ProteinSequence', 'ProteinSequences']*) – The protein sequence(s) to assign structures to.

Returns
The input sequence(s) with structures assigned where possible.

Return type
Union['ProteinSequence', 'ProteinSequences']

Raises
`ValueError` – If the input is neither a ProteinSequence nor a ProteinSequences object.

`get_available_structures()` → List[str]

Get a list of all available structure IDs.

Returns
A list of structure IDs available in the structure_map.

Return type
List[str]

`get_protein_sequences()` → *ProteinSequences*

Get a ProteinSequences object containing all available protein sequences.

Returns
A ProteinSequences object containing all available protein sequences.

Return type
ProteinSequences

Module contents

- Author: Evan Komp
- Created: 7/10/2024
- Company: National Renewable Energy Lab, Bioeneergy Science and Technology
- License: MIT

Submodules

aide_predict.utils.alignment_calls module

- Author: Evan Komp
- Created: 6/12/2024
- Company: Bottle Institute @ National Renewable Energy Lab, Bioeneergy Science and Technology
- License: MIT

`aide_predict.utils.alignment_calls.mafft_align`(*sequences: ProteinSequences*, *existing_alignment: ProteinSequences | None = None*, *realign: bool = False*, *output_fasta: str | None = None*) → *ProteinSequences*

Perform multiple sequence alignment using MAFFT.

Parameters

- **sequences** (ProteinSequences) – The sequences to align.
- **existing_alignment** (*Optional[ProteinSequences]*) – An existing alignment to add sequences to.
- **realign** (*bool*) – If True, realign all sequences from scratch. If False, add new sequences to existing alignment.
- **output_fasta** (*Optional[str]*) – Path to save the alignment. If None, a temporary file is used.

Returns

The aligned sequences, either in memory or on file depending on output_fasta.

Return type

ProteinSequences

Raises

- **subprocess.CalledProcessError** – If MAFFT execution fails.
- **FileNotFoundError** – If MAFFT is not installed or not in PATH.

`aide_predict.utils.alignment_calls.sw_global_pairwise`(*seq1: ProteinSequence*, *seq2: ProteinSequence*, *matrix: str = 'BLOSUM62'*, *gap_open: float = -10*, *gap_extend: float = -0.5*) → tuple[*ProteinSequence*, *ProteinSequence*]

Align two ProteinSequence objects using global alignment with a specified substitution matrix.

Parameters

- **seq1** (ProteinSequence) – The first protein sequence to align.
- **seq2** (ProteinSequence) – The second protein sequence to align.
- **matrix** (*str, optional*) – The substitution matrix to use. Defaults to 'BLOSUM62'.
- **gap_open** (*float, optional*) – The gap opening penalty. Defaults to -10.
- **gap_extend** (*float, optional*) – The gap extension penalty. Defaults to -0.5.

Returns

A tuple containing the aligned sequences as ProteinSequence objects.

Return type

tuple[*ProteinSequence*, *ProteinSequence*]

aide_predict.utils.badass module

- Author: Evan Komp
- Created: 1/16/2025
- Company: National Renewable Energy Lab, Bioeneergy Science and Technology
- License: MIT

Adaptation of the following work to use AIDE models as predictors: https://www.biorxiv.org/content/10.1101/2024.10.25.620340v1

class aide_predict.utils.badass.**BADASSOptimizer**(*predictor: Callable[[List[str]], ndarray]*, *reference_sequence:* ProteinSequence, *params:* BADASSOptimizerParams)

Bases: `object`

Wrapper for the BADASS protein sequence optimizer.

This class wraps the BADASS optimizer to work with AIDE's data structures and provides a simplified interface for optimization.

The optimizer uses simulated annealing with adaptive temperature cycling to explore the protein sequence space, detecting phase transitions to balance exploration and exploitation.

Parameters

- **predictor** – Function that takes a list of sequences and returns scores
- **reference_sequence** – Reference/wild-type protein sequence
- **params** – Optimization parameters

Example

```python # Define predictor that takes list of sequences, returns scores def predict(sequences: List[str]) -> np.ndarray:

return model.predict(sequences)

# Create optimizer optimizer = BADASSOptimizer(

predictor=predict, reference_sequence=wt, params=BADASSOptimizerParams(

num_mutations=3, num_iter=100

)

)

# Run optimization results, stats = optimizer.optimize()

# Plot results optimizer.plot() ```

---

**__init__**(*predictor: Callable[[List[str]], ndarray]*, *reference_sequence:* ProteinSequence, *params:* BADASSOptimizerParams)

Initialize the optimizer.

**optimize**() → Tuple[DataFrame, DataFrame]

Run the optimization process.

**Returns**
```

(results_df, stats_df)

- results_df: DataFrame with sequences and scores
- stats_df: DataFrame with optimization statistics

Return type
tuple

`plot`(*save_figs: bool = True*) → None

Generate visualization plots of the optimization process.

Parameters
`save_figs` – Whether to save plots to files

`property results: Tuple[DataFrame, DataFrame]`

Get the latest optimization results.

Returns

(results_df, stats_df) if optimize has been run,
(None, None) otherwise

Return type
tuple

`save_results`(*filename: str | None = None*) → None

Save optimization results to CSV files.

Parameters
`filename` – Base filename for saving results

`class aide_predict.utils.badass.BADASSOptimizerParams`(*seqs_per_iter: int = 500, num_iter: int = 200, init_score_batch_size: int = 500, temperature: float = 1.5, seed: int = 42, gamma: float = 0.5, cooling_rate: float = 0.92, num_mutations: int = 5, sites_to_ignore: List[int] | None = None, normalize_scores: bool = True, simple_simulated_annealing: bool = False, cool_then_heat: bool = False, adaptive_upper_threshold: float | int | None = None, n_seqs_to_keep: int | None = None, score_threshold: float | None = None, reversal_threshold: float | None = None*)

Bases: `object`

Parameters for the BADASS optimizer algorithm.

Parameters

- `seqs_per_iter` – Number of sequences to evaluate per iteration
- `num_iter` – Number of iterations to run
- `init_score_batch_size` – Batch size for initial scoring of single mutants
- `temperature` – Initial temperature for simulated annealing
- `seed` – Random seed
- `gamma` – Weight for variance boosting

- **cooling_rate** – Rate at which temperature decreases
- **num_mutations** – Number of mutations per sequence
- **sites_to_ignore** – Sites to exclude from mutation (1-indexed)
- **normalize_scores** – Whether to normalize scores
- **simple_simulated_annealing** – Use simple SA without adaptation
- **cool_then_heat** – Use cooling-then-heating schedule
- **adaptive_upper_threshold** – If float, use quantile. If int, use top N sequences
- **n_seqs_to_keep** – Number of sequences to keep in results
- **score_threshold** – Score threshold for phase transitions. If None, computed from data
- **reversal_threshold** – Score threshold for phase transition reversal. If None, computed

`adaptive_upper_threshold: float | int | None = None`

`cool_then_heat: bool = False`

`cooling_rate: float = 0.92`

`gamma: float = 0.5`

`init_score_batch_size: int = 500`

`n_seqs_to_keep: int | None = None`

`normalize_scores: bool = True`

`num_iter: int = 200`

`num_mutations: int = 5`

`reversal_threshold: float | None = None`

`score_threshold: float | None = None`

`seed: int = 42`

`seqs_per_iter: int = 500`

`simple_simulated_annealing: bool = False`

`sites_to_ignore: List[int] = None`

`temperature: float = 1.5`

`to_dict()` → dict

Convert parameters to dictionary format expected by BADASS.

aide_predict.utils.checks module

- Author: Evan Komp
- Created: 6/13/2024
- Company: Bottle Institute @ National Renewable Energy Lab, Bioeneergy Science and Technology
- License: MIT

Common checks to ensure that different pipeline components are compatable.

`aide_predict.utils.checks.`**`check_model_compatibility`**(*training_sequences: ProteinSequences | None = None*, *testing_sequences: ProteinSequences | None = None*, *wt: ProteinSequence | None = None*) → Dict[str, List[str]]

Check which models are compatible with the given data.

Parameters

- **training_sequences** (*Optional[ProteinSequences]*) – Training protein sequences.
- **testing_sequences** (*Optional[ProteinSequences]*) – Testing protein sequences.
- **wt** (*Optional[ProteinSequence]*) – Wild-type protein sequence.

Returns

A dictionary with two keys: 'compatible' and 'incompatible', each containing a list of compatible and incompatible model names respectively.

Return type

Dict[str, List[str]]

`aide_predict.utils.checks.`**`get_supported_tools`**()

aide_predict.utils.common module

- Author: Evan Komp
- Created: 6/11/2024
- Company: Bottle Institute @ National Renewable Energy Lab, Bioeneergy Science and Technology
- License: MIT

Common utility functions

class `aide_predict.utils.common.`**`MessageBool`**(*value*, *message*)

Bases: `object`

`aide_predict.utils.common.`**`convert_dvc_params`**(*dvc_params_dict: dict*)

DVC Creates a nested dict with the parameters.

We want an object that has nested attributes so that we can access parameters with dot notation.

`aide_predict.utils.common.`**`wrap`**(*text*, *width=80*)

Wraps a string at a fixed width.

Parameters

- **text** (*str*) – Text to be wrapped
- **width** (*int*) – Line width

Returns
Wrapped string

Return type
str

aide_predict.utils.conservation module

- Author: Evan Komp
- Created: 9/9/2024
- Company: National Renewable Energy Lab, Bioeneergy Science and Technology
- License: MIT

class aide_predict.utils.conservation.**ConservationAnalysis**(*protein_sequences: ProteinSequences*, *ignore_gaps: bool = True*)

Bases: `object`

A class for analyzing amino acid property conservation in protein sequence alignments.

This class provides methods to compute conservation scores and their statistical significance for various amino acid properties across aligned protein sequences. It can also compare conservation between two alignments.

Variables

- **PROPERTIES** (*Dict[str, set]*) – A dictionary mapping property names to sets of amino acids that possess that property.
- **EXPECTED_FREQUENCIES** (*Dict[str, float]*) – A dictionary mapping property names to their expected frequencies based on the 20 standard amino acids.

Parameters

- **protein_sequences** (ProteinSequences) – An aligned set of protein sequences.
- **ignore_gaps** (*bool*) – Whether to ignore gaps in conservation calculations. Default is True.

Raises
ValueError – If the input ProteinSequences object is not aligned.

EXPECTED_FREQUENCIES = {'Aliphatic': 0.13636363636363635, 'Aromatic': 0.18181818181818182, 'Charged': 0.22727272727272727, 'Hydrophobic': 0.5909090909090909, 'Negative': 0.09090909090909091, 'Polar': 0.5909090909090909, 'Positive': 0.13636363636363635, 'Proline': 0.045454545454545456, 'Small': 0.4090909090909091, 'Tiny': 0.13636363636363635, 'not_Aliphatic': 0.7272727272727273, 'not_Aromatic': 0.8181818181818182, 'not_Charged': 0.7727272727272727, 'not_Hydrophobic': 0.4090909090909091, 'not_Negative': 0.9090909090909091, 'not_Polar': 0.4090909090909091, 'not_Positive': 0.8636363636363636, 'not_Proline': 0.9545454545454546, 'not_Small': 0.5909090909090909, 'not_Tiny': 0.8636363636363636}

```
PROPERTIES = {'Aliphatic': {'I', 'L', 'V'}, 'Aromatic': {'F', 'H', 'W', 'Y'},
'Charged': {'D', 'E', 'H', 'K', 'R'}, 'Hydrophobic': {'A', 'C', 'F', 'G', 'H',
'I', 'K', 'L', 'M', 'T', 'V', 'W', 'Y'}, 'Negative': {'D', 'E'}, 'Polar': {'B',
'D', 'E', 'H', 'K', 'N', 'Q', 'R', 'S', 'T', 'W', 'Y', 'Z'}, 'Positive': {'H', 'K',
'R'}, 'Proline': {'P'}, 'Small': {'A', 'C', 'D', 'G', 'N', 'P', 'S', 'T', 'V'},
'Tiny': {'A', 'G', 'S'}, 'not_Aliphatic': {'A', 'C', 'D', 'E', 'F', 'G', 'H', 'K',
'M', 'N', 'Q', 'R', 'S', 'T', 'W', 'Y'}, 'not_Aromatic': {'A', 'B', 'C', 'D', 'E',
'G', 'I', 'K', 'L', 'M', 'N', 'P', 'Q', 'R', 'S', 'T', 'V', 'Z'}, 'not_Charged':
{'A', 'B', 'C', 'F', 'G', 'I', 'L', 'M', 'N', 'P', 'Q', 'S', 'T', 'V', 'W', 'Y',
'Z'}, 'not_Hydrophobic': {'B', 'D', 'E', 'N', 'P', 'Q', 'R', 'S', 'Z'},
'not_Negative': {'A', 'B', 'C', 'F', 'G', 'H', 'I', 'K', 'L', 'M', 'N', 'P', 'Q',
'R', 'S', 'T', 'V', 'W', 'Y', 'Z'}, 'not_Polar': {'A', 'C', 'F', 'G', 'I', 'L',
'M', 'P', 'V'}, 'not_Positive': {'A', 'B', 'C', 'D', 'E', 'F', 'G', 'I', 'L', 'M',
'N', 'P', 'Q', 'S', 'T', 'V', 'W', 'Y', 'Z'}, 'not_Proline': {'A', 'B', 'C', 'D',
'E', 'F', 'G', 'H', 'I', 'K', 'L', 'M', 'N', 'Q', 'R', 'S', 'T', 'V', 'W', 'Y',
'Z'}, 'not_Small': {'B', 'E', 'F', 'H', 'I', 'K', 'L', 'M', 'Q', 'R', 'W', 'Y',
'Z'}, 'not_Tiny': {'B', 'C', 'D', 'E', 'F', 'H', 'I', 'K', 'L', 'M', 'N', 'P', 'Q',
'R', 'T', 'V', 'W', 'Y', 'Z'}}
```

static compare_alignments(*alignment1: ProteinSequences*, *alignment2: ProteinSequences*, *ignore_gaps: bool = True*, *alpha: float = 0.01*) → Tuple[Dict[str, ndarray], Dict[str, ndarray]]

Compare conservation scores between two alignments and compute statistical significance.

Parameters

- **alignment1** (`ProteinSequences`) – The first aligned set of protein sequences.
- **alignment2** (`ProteinSequences`) – The second aligned set of protein sequences.
- **ignore_gaps** (*bool*) – Whether to ignore gaps in conservation calculations. Default is True.
- **alpha** (*float*) – The significance level for the binomial test. Default is 0.01.

Returns

A tuple containing:

1. A dictionary mapping property names to arrays of conservation score differences.
2. A dictionary mapping property names to arrays of p-values for the differences.

Return type

Tuple[Dict[str, np.ndarray], Dict[str, np.ndarray]]

Raises

`ValueError` – If the two alignments have different lengths.

compute_conservation() → Dict[str, ndarray]

Compute conservation scores for each amino acid property across all alignment positions.

Returns

A dictionary mapping property names to arrays of conservation scores. Each array has a length equal to the alignment width, with values between 0 and 1 representing the fraction of sequences that have the property at each position.

Return type

Dict[str, np.ndarray]

compute_significance(*alpha: float = 0.01*) → Dict[str, ndarray]

Compute the statistical significance of conservation for each property and position.

This method uses a binomial test to compare the observed frequency of each property to its expected frequency based on amino acid composition.

Parameters

alpha (*float, optional*) – The significance level for the binomial test. Defaults to 0.01.

Returns

A tuple containing:

1. A boolean array indicating significant positions (True if any property is significant).
2. A dictionary mapping property names to arrays of p-values for each position.

Return type

Tuple[np.ndarray, Dict[str, np.ndarray]]

aide_predict.utils.constants module

- Author: Evan Komp
- Created: 6/11/2024
- Company: Bottle Institute @ National Renewable Energy Lab, Bioeneergy Science and Technology
- License: MIT

aide_predict.utils.mmseqs_msa_search module

- Author: Evan Komp
- Created: 2/10/2025
- Company: National Renewable Energy Lab, Bioeneergy Science and Technology
- License: MIT

aide_predict.utils.mmseqs_msa_search.**main**()

Command line interface.

aide_predict.utils.mmseqs_msa_search.**run_mmseqs_command**(*params: List[str | Path]*, *capture_stderr: bool = False*, *dry_run: bool = False*) → None

Run an MMseqs2 command with logging.

aide_predict.utils.mmseqs_msa_search.**run_mmseqs_search**(*sequences:* ProteinSequences, *uniref_db: str | Path*, *output_dir: str | Path*, *metagenomic_db: str | Path | None = None*, *mode: str = 'standard'*, *threads: int = 4*, *remove_tmp: bool = False*, *dry_run: bool = False*) → List[str]

Generate MSAs for protein sequences using MMseqs2.

Parameters

- **sequences** – Input sequences to generate MSAs for

- **uniref_db** – Path to UniRef30 MMseqs2 database
- **output_dir** – Directory to save MSAs
- **metagenomic_db** – Optional path to environmental sequence database
- **mode** – Search sensitivity: - 'fast': Quick search (sensitivity 4.0) - 'standard': Balanced (sensitivity 5.7) - 'sensitive': More thorough (sensitivity 7.5)
- **threads** – Number of CPU threads to use
- **remove_tmp** – Whether to remove temporary files

Returns
List of paths to generated MSA files (one per sequence)

aide_predict.utils.msa module

- MSAProcessing class Refactored from Frazer et al.

@article{Frazer2021DiseaseVP,
title={Disease variant prediction with deep generative models of evolutionary data.}, author={Jonathan Frazer and Pascal Notin and Mafalda Dias and Aidan Gomez and Joseph K Min and Kelly P. Brock and Yarin Gal and Debora S. Marks}, journal={Nature}, year={2021}

}

- Author: Evan Komp
- Created: 5/8/2024
- (c) Copyright by Bottle Institute @ National Renewable Energy Lab, Bioeneergy Science and Technology

Peocessing of MSAs for preparation of input data for the zero-shot model. Note that The MSAProcessing class IS A REFACTORING of the MSA processing class from The marks Lab https://github.com/OATML-Markslab/EVE/blob/master/utils/data_utils.py Credit is given to them for the original implementation and the methodology of sequence weighting. Here, we make it more pythonic and readbale, as well as an order of magnitude speed up.

In addition to refactoring, we add some additional functionality: - A focus seq need not be present, in which case all columns are considered focus columns and contribute to weight computation - One Hot encoding is reworked to use sklearn's OneHotEncoder instead of a loop of loops, with about an order of magnitude speedup - Weight computation leverages numpy array indexing instead of a loop, and if torch is available

and advanced hardware is present, GPU is used.

Tested on 10000 protein sequences sequences of length 55:

- original: 8.9 seconds
- cpu array operations: 1.2 seconds (7.4x speedup)
- gpu array operations: 0.2 seconds (44.5x speedup)

- other minor speedups with array operations

`class aide_predict.utils.msa.MSAProcessing`(*theta: float = 0.2*, *use_weights: bool = True*, *preprocess_msa: bool = True*, *threshold_sequence_frac_gaps: float = 0.5*, *threshold_focus_cols_frac_gaps: float = 0.3*, *remove_sequences_with_indeterminate_aa_in_focus_cols: bool = True*, *weight_computation_batch_size: int = 10000*, *ignore_gaps_in_weighting: bool = False*)

Bases: `object`

__init__(*theta: float = 0.2*, *use_weights: bool = True*, *preprocess_msa: bool = True*, *threshold_sequence_frac_gaps: float = 0.5*, *threshold_focus_cols_frac_gaps: float = 0.3*, *remove_sequences_with_indeterminate_aa_in_focus_cols: bool = True*, *weight_computation_batch_size: int = 10000*, *ignore_gaps_in_weighting: bool = False*)

Initialize the MSAProcessing class.

Parameters

- **theta** (*float*) – Sequence weighting hyperparameter.
- **use_weights** (*bool*) – Whether to compute and use sequence weights.
- **preprocess_msa** (*bool*) – Whether to preprocess the MSA.
- **threshold_sequence_frac_gaps** (*float*) – Threshold for removing sequences with too many gaps.
- **threshold_focus_cols_frac_gaps** (*float*) – Threshold for determining focus columns.
- **remove_sequences_with_indeterminate_aa_in_focus_cols** (*bool*) – Whether to remove sequences with indeterminate AAs in focus columns.
- **weight_computation_batch_size** (*int*) – Batch size for weight computation.

compute_conservation(*msa*, *normalize=True*, *gap_treatment='exclude'*, *gap_characters={'-', '.'}*)

Compute the conservation score for each column in the MSA.

This method calculates the entropy-based conservation for each position in the alignment, with an option to normalize values between 0 (variable) and 1 (conserved).

Parameters

- **msa** (*ProteinSequences*) – The multiple sequence alignment to analyze.
- **normalize** (*bool, optional (default=True)*) – Whether to normalize entropy scores to range from 0 (variable) to 1 (conserved).
- **gap_treatment** (*str, optional (default='exclude')*) – How to handle gaps in conservation calculation: - 'exclude': Gaps are excluded from frequency calculation - 'include': Gaps are treated as normal characters - 'penalize': Columns with high gap content are penalized
- **gap_characters** (*set or list, optional (default=GAP_CHARACTERS)*) – Characters to be considered as gaps.

Returns

Vector of length L with conservation scores for each column.

Return type

numpy.ndarray

Notes

- If sequence weights are available in the MSA, they will be used to calculate

weighted frequencies for more accurate conservation measurement. - Conservation is calculated using the Shannon entropy of the amino acid distribution at each position, with an option to normalize to the [0,1] range. - Gaps can significantly affect conservation scores. The 'exclude' option removes gaps from consideration, 'include' treats them as valid characters, and 'penalize' reduces the conservation score based on gap frequency.

---

get_most_populated_chunk(*msa: ProteinSequences*, *chunk_size: int*) → *ProteinSequences*

Get the most populated chunk of contiguous columns from the MSA.

Parameters

- **msa** (ProteinSequences) – The input MSA.
- **chunk_size** (*int*) – The size of the chunk.

Returns
The chunk of contiguous columns.

Return type
ProteinSequences

process(*msa: ProteinSequences*, *focus_seq_id: str | None = None*) → *ProteinSequences*

Process the input MSA.

Parameters

- **msa** (ProteinSequences) – The input multiple sequence alignment.
- **focus_seq_id** (*Optional[str]*) – The ID of the focus sequence. If None, no focus sequence is used.

Returns
The processed MSA with computed weights.

Return type
ProteinSequences

remove_gappy_columns(*msa: ProteinSequences*, *gap_threshold: float | None = None*, *focus_seq_id: str | None = None*) → *ProteinSequences*

Remove columns from the MSA that exceed the gap threshold.

All sequences are retained, but columns with gap fraction above the threshold are completely removed from the alignment.

Parameters

- **msa** (ProteinSequences) – The input multiple sequence alignment.
- **gap_threshold** (*Optional[float]*) – Maximum allowed fraction of gaps per column. If None, uses self.threshold_focus_cols_frac_gaps.
- **focus_seq_id** (*Optional[str]*) – If provided, only consider gaps relative to non-gap positions in the focus sequence. If None, consider all positions.

Returns
New MSA with high-gap columns removed.

Return type
ProteinSequences

Raises

- **ValueError** – If the input MSA is not aligned.
- **ValueError** – If gap_threshold is not between 0 and 1.
- **ValueError** – If focus_seq_id is provided but not found in MSA.

aide_predict.utils.plotting module

- Author: Evan Komp
- Created: 7/26/2024
- Company: National Renewable Energy Lab, Bioeneergy Science and Technology
- License: MIT

Common plotting calls.

`aide_predict.utils.plotting.`**`plot_conservation`**(*conservation_scores: Dict[str, ndarray]*, *p_values: Dict[str, ndarray] | None = None*, *alpha: float = 1e-10*, *stacked: bool = False*, *figsize: tuple = (20, 6)*, *title: str = 'Conservation Scores Across Alignment Positions'*) → Figure

Create a bar plot of conservation scores across alignment positions.

Parameters

- **conservation_scores** (*Dict[str, np.ndarray]*) – Dictionary of conservation scores for each property.
- **p_values** (*Optional[Dict[str, np.ndarray]]*) – Dictionary of p-values for each property. If provided, insignificant bars will be colored grey.
- **alpha** (*float*) – Significance level for p-values. Default is 0.05.
- **stacked** (*bool*) – If True, create a stacked bar plot with colors for different properties. If False, create a single bar plot with height determined by sum of conservation scores.
- **figsize** (*tuple*) – Figure size (width, height) in inches. Default is (12, 6).
- **title** (*str*) – Title of the plot. Default is "Conservation Scores Across Alignment Positions".

Returns

The matplotlib Figure object containing the plot.

Return type

plt.Figure

`aide_predict.utils.plotting.`**`plot_mutation_heatmap`**(*mutations*, *scores*)

Plot a heatmap of single point mutation scores.

Parameters: mutations (list): List of mutation strings (e.g., ["L1V", "A2G", …]) scores (list): List of corresponding scores

Returns: None (displays the plot)

`aide_predict.utils.plotting.`**`plot_protein_sequence_heatmap`**(*sequences: ProteinSequences*, *figsize: tuple = (20, 5)*, *cmap: str = 'viridis'*, *title: str = 'Protein Sequence Heatmap'*) → Figure

Create a heatmap visualization of protein sequences with additional sequence properties.

Parameters

- **sequences** (*ProteinSequences*) – A ProteinSequences object containing the protein sequences.
- **figsize** (*tuple*) – Figure size (width, height) in inches.

- **cmap** (*str*) – Colormap to use for the heatmap.
- **title** (*str*) – Title of the plot.

Returns
The matplotlib Figure object containing the heatmap.

Return type
plt.Figure

aide_predict.utils.soloseq module

- Author: Evan Komp
- Created: 2/7/2025
- Company: National Renewable Energy Lab, Bioeneergy Science and Technology
- License: MIT

Wrapper for SoloSeq structure prediction. See: https://github.com/aqlaboratory/openfold/blob/main/docs/inference.md#soloseq-inference

Requires: 1. OpenFold repo cloned and environment installed 2. Model weights downloaded 3. Environment variables set:

- OPENFOLD_ENV_NAME: Name of conda environment
- OPENFOLD_DIR: Path to OpenFold repo

`aide_predict.utils.soloseq.main()`

Command line interface.

`aide_predict.utils.soloseq.run_soloseq(`*sequences: ProteinSequences, output_dir: str, use_gpu: bool = True, skip_relaxation: bool = False, save_embeddings: bool = False, device: str = 'cuda:0', force: bool = False*`)` → List[str]

Run SoloSeq structure prediction on a set of sequences.

Parameters

- **sequences** – Input sequences to predict
- **output_dir** – Directory to save results
- **use_gpu** – Whether to use GPU
- **skip_relaxation** – Skip relaxation step
- **save_embeddings** – Save ESM embeddings
- **device** – GPU device to use
- **force** – If True, rerun predictions even if they exist

Returns
List of paths to predicted structure files

Note: Sequences longer than 1022 residues will be truncated.

Module contents

- Author: Evan Komp
- Created: 5/7/2024
- (c) Copyright by Bottle Institute @ National Renewable Energy Lab, Bioeneergy Science and Technology

Submodules

aide_predict.patches_ module

aide_predict.patches_.**patch_pandas_append**()

aide_predict.patches_.**patched_parse_plmc_log**(*log*)

A patched version of parse_plmc_log that handles the new output format.

Module contents

- Author: Evan Komp
- Created: 5/7/2024
- (c) Copyright by Bottle Institute @ National Renewable Energy Lab, Bioeneergy Science and Technology

CHAPTER

THREE

INDICES AND TABLES

- genindex
- modindex
- search

PYTHON MODULE INDEX

a

aide_predict, 196
aide_predict.bespoke_models, 152
aide_predict.bespoke_models.base, 144
aide_predict.bespoke_models.embedders, 87
aide_predict.bespoke_models.embedders.esm2, 57
aide_predict.bespoke_models.embedders.kmer, 62
aide_predict.bespoke_models.embedders.msa_transformer, 66
aide_predict.bespoke_models.embedders.ohe, 72
aide_predict.bespoke_models.embedders.saprot, 82
aide_predict.bespoke_models.predictors, 144
aide_predict.bespoke_models.predictors.esm2, 87
aide_predict.bespoke_models.predictors.eve, 94
aide_predict.bespoke_models.predictors.evmutation, 101
aide_predict.bespoke_models.predictors.hmm, 106
aide_predict.bespoke_models.predictors.msa_transformer, 113
aide_predict.bespoke_models.predictors.pretrained_transformers, 120
aide_predict.bespoke_models.predictors.saprot, 128
aide_predict.bespoke_models.predictors.ssemb, 133
aide_predict.bespoke_models.predictors.vespa, 139
aide_predict.io, 153
aide_predict.io.bio_files, 152
aide_predict.patches_, 196
aide_predict.utils, 196
aide_predict.utils.alignment_calls, 182
aide_predict.utils.badass, 184
aide_predict.utils.checks, 187
aide_predict.utils.common, 187
aide_predict.utils.conservation, 188
aide_predict.utils.constants, 190
aide_predict.utils.data_structures, 182
aide_predict.utils.data_structures.sequences, 153
aide_predict.utils.data_structures.structures, 179
aide_predict.utils.mmseqs_msa_search, 190
aide_predict.utils.msa, 191
aide_predict.utils.plotting, 194
aide_predict.utils.soloseq, 195

INDEX

Symbols

__init__() (*aide_predict.bespoke_models.base.ProteinModelWrapper method*), 147
__init__() (*aide_predict.bespoke_models.embedders.esm2.ESM2Embedding method*), 58
__init__() (*aide_predict.bespoke_models.embedders.kmer.KmerEmbedding method*), 62
__init__() (*aide_predict.bespoke_models.embedders.msa_transformer.MSATransformerEmbedding method*), 68
__init__() (*aide_predict.bespoke_models.embedders.ohe.OneHotAlignedEmbedding method*), 73
__init__() (*aide_predict.bespoke_models.embedders.ohe.OneHotProteinEmbedding method*), 77
__init__() (*aide_predict.bespoke_models.embedders.saprot.SaProtEmbedding method*), 82
__init__() (*aide_predict.bespoke_models.predictors.eve.EVEWrapper method*), 95
__init__() (*aide_predict.bespoke_models.predictors.evmutation.EVMutationWrapper method*), 101
__init__() (*aide_predict.bespoke_models.predictors.hmm.HMMWrapper method*), 108
__init__() (*aide_predict.bespoke_models.predictors.msa_transformer.MSATransformerLikelihoodWrapper method*), 115
__init__() (*aide_predict.bespoke_models.predictors.pretrained_transformers.LikelihoodTransformerBase method*), 122
__init__() (*aide_predict.bespoke_models.predictors.ssemb.SSEmbWrapper method*), 134
__init__() (*aide_predict.bespoke_models.predictors.vespa.VESPAWrapper method*), 139
__init__() (*aide_predict.utils.badass.BADASSOptimizer method*), 184
__init__() (*aide_predict.utils.data_structures.sequences.ProteinSequences method*), 167
__init__() (*aide_predict.utils.data_structures.sequences.ProteinSequencesOnFile method*), 173
__init__() (*aide_predict.utils.data_structures.structures.StructureMapper method*), 181
__init__() (*aide_predict.utils.msa.MSAProcessing method*), 191

A

accepts_lower_case (*aide_predict.bespoke_models.base.ProteinModelWrapper property*), 147
accepts_lower_case (*aide_predict.bespoke_models.embedders.esm2.ES*[illegible] *property*), 58
accepts_lower_case (*aide_predict.bespoke_models.embedders.kmer.Km*[illegible] *property*), 62
accepts_lower_case (*aide_predict.bespoke_models.embedders.msa_tran*[illegible] *property*), 68
accepts_lower_case (*aide_predict.bespoke_models.embedders.ohe.One*[illegible] *property*), 73
accepts_lower_case (*aide_predict.bespoke_models.embedders.ohe.One*[illegible] *property*), 78
accepts_lower_case (*aide_predict.bespoke_models.embedders.saprot.Sa*[illegible] *property*), 83
accepts_lower_case (*aide_predict.bespoke_models.predictors.esm2.ESM*[illegible] *property*), 89
accepts_lower_case (*aide_predict.bespoke_models.predictors.eve.EVEW*[illegible] *property*), 96
accepts_lower_case (*aide_predict.bespoke_models.predictors.evmutatio*[illegible] *property*), 102
accepts_lower_case (*aide_predict.bespoke_models.predictors.hmm.HM*[illegible] *property*), 108
accepts_lower_case (*aide_predict.bespoke_models.predictors.msa_trans*[illegible] *property*), 115
accepts_lower_case (*aide_predict.bespoke_models.predictors.pretrained*[illegible] *property*), 122
accepts_lower_case (*aide_predict.bespoke_models.predictors.saprot.Sa*[illegible] *property*), 128
accepts_lower_case (*aide_predict.bespoke_models.predictors.ssemb.SS*[illegible] *property*), 134
accepts_lower_case (*aide_predict.bespoke_models.predictors.vespa.VE*[illegible] *property*), 139
AcceptsLowerCaseMixin (*class in aide_predict.bespoke_models.base*), 144
adaptive_upper_threshold (*aide_predict.utils.badass.BADASSOptimizerParams attribute*), 186
aide_predict
    module, 196
aide_predict.bespoke_models
    module, 152
aide_predict.bespoke_models.base
    module, 144

aide_predict.bespoke_models.embedders
module, 87
aide_predict.bespoke_models.embedders.esm2
module, 57
aide_predict.bespoke_models.embedders.kmer
module, 62
aide_predict.bespoke_models.embedders.msa_transformer
module, 66
aide_predict.bespoke_models.embedders.ohe
module, 72
aide_predict.bespoke_models.embedders.saprot
module, 82
aide_predict.bespoke_models.predictors
module, 144
aide_predict.bespoke_models.predictors.esm2
module, 87
aide_predict.bespoke_models.predictors.eve
module, 94
aide_predict.bespoke_models.predictors.evmutation
module, 101
aide_predict.bespoke_models.predictors.hmm
module, 106
aide_predict.bespoke_models.predictors.msa_transformer
module, 113
aide_predict.bespoke_models.predictors.pretrained_transformers
module, 120
aide_predict.bespoke_models.predictors.saprot
module, 128
aide_predict.bespoke_models.predictors.ssemb
module, 133
aide_predict.bespoke_models.predictors.vespa
module, 139
aide_predict.io
module, 153
aide_predict.io.bio_files
module, 152
aide_predict.patches_
module, 196
aide_predict.utils
module, 196
aide_predict.utils.alignment_calls
module, 182
aide_predict.utils.badass
module, 184
aide_predict.utils.checks
module, 187
aide_predict.utils.common
module, 187
aide_predict.utils.conservation
module, 188
aide_predict.utils.constants
module, 190
aide_predict.utils.data_structures
module, 182
aide_predict.utils.data_structures.sequences
module, 153
aide_predict.utils.data_structures.structures
module, 179
aide_predict.utils.mmseqs_msa_search
module, 190
aide_predict.utils.msa
module, 191
aide_predict.utils.plotting
module, 194
aide_predict.utils.soloseq
module, 195
align() (*aide_predict.utils.data_structures.sequences.ProteinSequence method*), 159
align_all() (*aide_predict.utils.data_structures.sequences.ProteinSequen method*), 167
align_all() (*aide_predict.utils.data_structures.sequences.ProteinSequen method*), 173
align_to() (*aide_predict.utils.data_structures.sequences.ProteinSequence method*), 167
align_to() (*aide_predict.utils.data_structures.sequences.ProteinSequence method*), 174
aligned (*aide_predict.utils.data_structures.sequences.ProteinSequences property*), 168
aligned (*aide_predict.utils.data_structures.sequences.ProteinSequencesOn property*), 174
append() (*aide_predict.utils.data_structures.sequences.ProteinSequences method*), 168
append() (*aide_predict.utils.data_structures.sequences.ProteinSequencesO method*), 174
apply_alignment_mapping() (*aide_predict.utils.data_structures.sequences.ProteinSequences method*), 168
apply_alignment_mapping() (*aide_predict.utils.data_structures.sequences.ProteinSequencesO method*), 174
as_array (*aide_predict.utils.data_structures.sequences.ProteinSequence property*), 159
as_array() (*aide_predict.utils.data_structures.sequences.ProteinSequence method*), 168
as_array() (*aide_predict.utils.data_structures.sequences.ProteinSequence method*), 174
assign_structures() (*aide_predict.utils.data_structures.structures.StructureMapper method*), 181

B

BADASSOptimizer (*class in aide_predict.utils.badass*), 184
BADASSOptimizerParams (*class in aide_predict.utils.badass*), 185
base_length (*aide_predict.utils.data_structures.sequences.ProteinSequen property*), 159

C

CacheMixin (*class in aide_predict.bespoke_models.base*), 144

can_handle_aligned_sequences (*aide_predict.bespoke_models.base.ProteinModelWrapper property*), 147

can_handle_aligned_sequences (*aide_predict.bespoke_models.embedders.esm2.ESM2Embedding property*), 58

can_handle_aligned_sequences (*aide_predict.bespoke_models.embedders.kmer.KmerEmbedding property*), 62

can_handle_aligned_sequences (*aide_predict.bespoke_models.embedders.msa_transformer.MSATransformerEmbedding property*), 68

can_handle_aligned_sequences (*aide_predict.bespoke_models.embedders.ohe.OneHotAlignedEmbedding property*), 73

can_handle_aligned_sequences (*aide_predict.bespoke_models.embedders.ohe.OneHotProteinEmbedding property*), 78

can_handle_aligned_sequences (*aide_predict.bespoke_models.embedders.saprot.SaProtEmbedding property*), 83

can_handle_aligned_sequences (*aide_predict.bespoke_models.predictors.esm2.ESM2LikelihoodWrapper property*), 89

can_handle_aligned_sequences (*aide_predict.bespoke_models.predictors.eve.EVEWrapper property*), 96

can_handle_aligned_sequences (*aide_predict.bespoke_models.predictors.evmutation.EVMutationWrapper property*), 102

can_handle_aligned_sequences (*aide_predict.bespoke_models.predictors.hmm.HMMWrapper property*), 108

can_handle_aligned_sequences (*aide_predict.bespoke_models.predictors.msa_transformer.MSATransformerLikelihoodWrapper property*), 115

can_handle_aligned_sequences (*aide_predict.bespoke_models.predictors.pretrained_transformers.LikelihoodTransformerBase property*), 122

can_handle_aligned_sequences (*aide_predict.bespoke_models.predictors.saprot.SaProtLikelihoodWrapper property*), 128

can_handle_aligned_sequences (*aide_predict.bespoke_models.predictors.ssemb.SSEmbWrapper property*), 134

can_handle_aligned_sequences (*aide_predict.bespoke_models.predictors.vespa.VESPAWrapper property*), 139

can_regress (*aide_predict.bespoke_models.base.ProteinModelWrapper property*), 147

can_regress (*aide_predict.bespoke_models.embedders.esm2.ESM2Embedding property*), 58

can_regress (*aide_predict.bespoke_models.embedders.kmer.KmerEmbedd property*), 62

can_regress (*aide_predict.bespoke_models.embedders.msa_transformer.M property*), 68

can_regress (*aide_predict.bespoke_models.embedders.ohe.OneHotAligne property*), 73

can_regress (*aide_predict.bespoke_models.embedders.ohe.OneHotProtei property*), 78

can_regress (*aide_predict.bespoke_models.embedders.saprot.SaProtEmb property*), 83

can_regress (*aide_predict.bespoke_models.predictors.esm2.ESM2Likelih property*), 89

can_regress (*aide_predict.bespoke_models.predictors.eve.EVEWrapper property*), 96

can_regress (*aide_predict.bespoke_models.predictors.evmutation.EVMut property*), 102

can_regress (*aide_predict.bespoke_models.predictors.hmm.HMMWrappe property*), 108

can_regress (*aide_predict.bespoke_models.predictors.msa_transformer.M property*), 115

can_regress (*aide_predict.bespoke_models.predictors.pretrained_transfo property*), 122

can_regress (*aide_predict.bespoke_models.predictors.saprot.SaProtLikel property*), 128

can_regress (*aide_predict.bespoke_models.predictors.ssemb.SSEmbWrap property*), 134

can_regress (*aide_predict.bespoke_models.predictors.vespa.VESPAWrap property*), 139

CanHandleAlignedSequencesMixin (*class in aide_predict.bespoke_models.base*), 144

CanRegressMixin (*class in aide_predict.bespoke_models.base*), 145

capitalize() (*aide_predict.utils.data_structures.sequences.ProteinChara method*), 154

capitalize() (*aide_predict.utils.data_structures.sequences.ProteinSeque method*), 159

casefold() (*aide_predict.utils.data_structures.sequences.ProteinCharacte method*), 154

casefold() (*aide_predict.utils.data_structures.sequences.ProteinSequence method*), 159

center() (*aide_predict.utils.data_structures.sequences.ProteinCharacter method*), 154

center() (*aide_predict.utils.data_structures.sequences.ProteinSequence method*), 159

chain (*aide_predict.utils.data_structures.structures.ProteinStructure attribute*), 180

check_metadata() (*aide_predict.bespoke_models.base.ProteinModelWra method*), 147

check_metadata() (*aide_predict.bespoke_models.embedders.esm2.ESM2 method*), 58

check_metadata() (*aide_predict.bespoke_models.embedders.kmer.Kmerl method*), 63

check_metadata() (*aide_predict.bespoke_models.embedders.msa_transfo*

method), 68
check_metadata() (*aide_predict.bespoke_models.embedders.ohe.OneHotAlignedEmbedding method*), 73
check_metadata() (*aide_predict.bespoke_models.embedders.ohe.OneHotProteinEmbedding method*), 78
check_metadata() (*aide_predict.bespoke_models.embedders.saprot.SaProtEmbedding method*), 83
check_metadata() (*aide_predict.bespoke_models.predictors.esm2.ESM2LikelihoodWrapper method*), 89
check_metadata() (*aide_predict.bespoke_models.predictors.eve.EVEWrapper method*), 96
check_metadata() (*aide_predict.bespoke_models.predictors.evmutation.EVMutationWrapper method*), 102
check_metadata() (*aide_predict.bespoke_models.predictors.hmm.HMMWrapper method*), 108
check_metadata() (*aide_predict.bespoke_models.predictors.msa_transformer.MSATransformerLikelihoodWrapper method*), 115
check_metadata() (*aide_predict.bespoke_models.predictors.pretrained_transformers.LikelihoodTransformerBase method*), 122
check_metadata() (*aide_predict.bespoke_models.predictors.saprot.SaProtLikelihoodWrapper method*), 128
check_metadata() (*aide_predict.bespoke_models.predictors.ssemb.SSEmbWrapper method*), 134
check_metadata() (*aide_predict.bespoke_models.predictors.vespa.VESPAWrapper method*), 139
check_model_compatibility() (*in module aide_predict.utils.checks*), 187
clear() (*aide_predict.utils.data_structures.sequences.ProteinSequences method*), 168
clear() (*aide_predict.utils.data_structures.sequences.ProteinSequencesOnFile method*), 175
compare_alignments() (*aide_predict.utils.conservation.ConservationAnalysis static method*), 189
compute_conservation() (*aide_predict.utils.conservation.ConservationAnalysis method*), 189
compute_conservation() (*aide_predict.utils.msa.MSAProcessing method*), 192
compute_significance() (*aide_predict.utils.conservation.ConservationAnalysis method*), 189
ConservationAnalysis (*class in aide_predict.utils.conservation*), 188
convert_dvc_params() (*in module aide_predict.utils.common*), 187
cool_then_heat (*aide_predict.utils.badass.BADASSOptimizerParams attribute*), 186
cooling_rate (*aide_predict.utils.badass.BADASSOptimizerParams attribute*), 186
copy() (*aide_predict.utils.data_structures.sequences.ProteinSequences method*), 168
copy() (*aide_predict.utils.data_structures.sequences.ProteinSequencesOnFile*
method), 175
count() (*aide_predict.utils.data_structures.sequences.ProteinCharacter method*), 154
count() (*aide_predict.utils.data_structures.sequences.ProteinSequence method*), 159
count() (*aide_predict.utils.data_structures.sequences.ProteinSequences method*), 168
count() (*aide_predict.utils.data_structures.sequences.ProteinSequencesOn method*), 175

E

encode() (*aide_predict.utils.data_structures.sequences.ProteinCharacter method*), 154
encode() (*aide_predict.utils.data_structures.sequences.ProteinSequence method*), 159
endswith() (*aide_predict.utils.data_structures.sequences.ProteinCharacte method*), 154
endswith() (*aide_predict.utils.data_structures.sequences.ProteinSequenc method*), 159
ESM2Embedding (*class in aide_predict.bespoke_models.embedders.esm2*), 57
ESM2LikelihoodWrapper (*class in aide_predict.bespoke_models.predictors.esm2*), 88
EVEWrapper (*class in aide_predict.bespoke_models.predictors.eve*), 94
EVMutationWrapper (*class in aide_predict.bespoke_models.predictors.evmutation*), 101
expandtabs() (*aide_predict.utils.data_structures.sequences.ProteinChara method*), 154
expandtabs() (*aide_predict.utils.data_structures.sequences.ProteinSeque method*), 159
EXPECTED_FREQUENCIES (*aide_predict.utils.conservation.ConservationAnalysis attribute*), 188
expects_no_fit (*aide_predict.bespoke_models.base.ProteinModelWrapp property*), 147
expects_no_fit (*aide_predict.bespoke_models.embedders.esm2.ESM2Em property*), 58
expects_no_fit (*aide_predict.bespoke_models.embedders.kmer.KmerEm property*), 63
expects_no_fit (*aide_predict.bespoke_models.embedders.msa_transform property*), 68
expects_no_fit (*aide_predict.bespoke_models.embedders.ohe.OneHotAl property*), 73
expects_no_fit (*aide_predict.bespoke_models.embedders.ohe.OneHotPr property*), 78
expects_no_fit (*aide_predict.bespoke_models.embedders.saprot.SaProtE property*), 83
expects_no_fit (*aide_predict.bespoke_models.predictors.esm2.ESM2Lik property*), 89

`expects_no_fit` (*aide_predict.bespoke_models.predictors.eve.EVEWrapper property*), 96
`expects_no_fit` (*aide_predict.bespoke_models.predictors.evmutation.EVMutationWrapper property*), 102
`expects_no_fit` (*aide_predict.bespoke_models.predictors.hmm.HMMWrapper property*), 108
`expects_no_fit` (*aide_predict.bespoke_models.predictors.msa_transformer.MSATransformerLikelihoodWrapper property*), 115
`expects_no_fit` (*aide_predict.bespoke_models.predictors.pretrained_transformers.LikelihoodTransformerBase property*), 122
`expects_no_fit` (*aide_predict.bespoke_models.predictors.saprot.SaProtLikelihoodWrapper property*), 128
`expects_no_fit` (*aide_predict.bespoke_models.predictors.ssemb.SSEmbWrapper property*), 134
`expects_no_fit` (*aide_predict.bespoke_models.predictors.vespa.VESPAWrapper property*), 139
`ExpectsNoFitMixin` (*class in aide_predict.bespoke_models.base*), 145
`extend()` (*aide_predict.utils.data_structures.sequences.Prot[illegible] method*), 168
`extend()` (*aide_predict.utils.data_structures.sequences.Prot[illegible] method*), 175

F

`find()` (*aide_predict.utils.data_structures.sequences.ProteinCharacter method*), 154
`find()` (*aide_predict.utils.data_structures.sequences.ProteinSequence method*), 159
`fit()` (*aide_predict.bespoke_models.base.ProteinModelWrapper method*), 148
`fit()` (*aide_predict.bespoke_models.embedders.esm2.ESM2Embedding method*), 58
`fit()` (*aide_predict.bespoke_models.embedders.kmer.KmerEmbedding method*), 63
`fit()` (*aide_predict.bespoke_models.embedders.msa_transformer.MSATransformerEmbedding method*), 68
`fit()` (*aide_predict.bespoke_models.embedders.ohe.OneHotAlignedEmbedding method*), 73
`fit()` (*aide_predict.bespoke_models.embedders.ohe.OneHotProteinEmbedding method*), 78
`fit()` (*aide_predict.bespoke_models.embedders.saprot.SaProtEmbedding method*), 83
`fit()` (*aide_predict.bespoke_models.predictors.esm2.ESM2LikelihoodWrapper method*), 89
`fit()` (*aide_predict.bespoke_models.predictors.eve.EVEWrapper method*), 96
`fit()` (*aide_predict.bespoke_models.predictors.evmutation.EVMutationWrapper method*), 102
`fit()` (*aide_predict.bespoke_models.predictors.hmm.HMMWrapper method*), 108
`fit()` (*aide_predict.bespoke_models.predictors.msa_transformer.MSATransformerLikelihoodWrapper method*), 115
`fit()` (*aide_predict.bespoke_models.predictors.pretrained_transformers.LikelihoodTransformerBase method*), 122
`fit()` (*aide_predict.bespoke_models.predictors.saprot.SaProtLikelihoodW method*), 128
`fit()` (*aide_predict.bespoke_models.predictors.ssemb.SSEmbWrapper method*), 134
`fit()` (*aide_predict.bespoke_models.predictors.vespa.VESPAWrapper method*), 139
`fit_transform()` (*aide_predict.bespoke_models.base.ProteinModelWrap method*), 148
`fit_transform()` (*aide_predict.bespoke_models.embedders.esm2.ESM2E method*), 58
`fit_transform()` (*aide_predict.bespoke_models.embedders.kmer.KmerE method*), 63
`fit_transform()` (*aide_predict.bespoke_models.embedders.msa_transfor method*), 69
`fit_transform()` (*aide_predict.bespoke_models.embedders.ohe.OneHotA method*), 74
`fit_transform()` (*aide_predict.bespoke_models.embedders.ohe.OneHotP method*), 78
`fit_transform()` (*aide_predict.bespoke_models.embedders.saprot.SaPro method*), 83
`fit_transform()` (*aide_predict.bespoke_models.predictors.esm2.ESM2L method*), 89
`fit_transform()` (*aide_predict.bespoke_models.predictors.eve.EVEWrap method*), 97
`fit_transform()` (*aide_predict.bespoke_models.predictors.evmutation.E method*), 102
`fit_transform()` (*aide_predict.bespoke_models.predictors.hmm.HMMW method*), 108
`fit_transform()` (*aide_predict.bespoke_models.predictors.msa_transfor method*), 116
`fit_transform()` (*aide_predict.bespoke_models.predictors.pretrained_tr method*), 123
`fit_transform()` (*aide_predict.bespoke_models.predictors.saprot.SaProt method*), 129
`fit_transform()` (*aide_predict.bespoke_models.predictors.ssemb.SSEmb method*), 135
`fit_transform()` (*aide_predict.bespoke_models.predictors.vespa.VESPA method*), 140
`fixed_length` (*aide_predict.utils.data_structures.sequences.ProteinSeque property*), 168
`fixed_length` (*aide_predict.utils.data_structures.sequences.ProteinSeque property*), 175
`format()` (*aide_predict.utils.data_structures.sequences.ProteinCharacter method*), 154
`format()` (*aide_predict.utils.data_structures.sequences.ProteinSequence method*), 160
`format_map()` (*aide_predict.utils.data_structures.sequences.ProteinChara method*), 154
`format_map()` (*aide_predict.utils.data_structures.sequences.ProteinSeque method*), 160
`from_a3m()` (*aide_predict.utils.data_structures.sequences.ProteinSequence class method*), 160
`from_ca3m()` (*aide_predict.utils.data_structures.sequences.ProteinSequence class method*), 168

from_a3m() (*aide_predict.utils.data_structures.sequences.ProteinSequencesOnFile class method*), 175
from_af2_folder() (*aide_predict.utils.data_structures.structures.ProteinStructure class method*), 180
from_csv() (*aide_predict.utils.data_structures.sequences.ProteinSequences class method*), 169
from_csv() (*aide_predict.utils.data_structures.sequences.ProteinSequencesOnFile class method*), 175
from_df() (*aide_predict.utils.data_structures.sequences.ProteinSequences class method*), 169
from_df() (*aide_predict.utils.data_structures.sequences.ProteinSequencesOnFile class method*), 176
from_dict() (*aide_predict.utils.data_structures.sequences.ProteinSequences class method*), 170
from_dict() (*aide_predict.utils.data_structures.sequences.ProteinSequencesOnFile class method*), 176
from_fasta() (*aide_predict.utils.data_structures.sequences.ProteinSequence class method*), 160
from_fasta() (*aide_predict.utils.data_structures.sequences.ProteinSequences class method*), 170
from_fasta() (*aide_predict.utils.data_structures.sequences.ProteinSequences method*), 167
from_fasta() (*aide_predict.utils.data_structures.sequences.ProteinSequencesOnFile class method*), 176
from_fasta() (*aide_predict.utils.data_structures.sequences.ProteinSequencesOnFile method*), 173
from_list() (*aide_predict.utils.data_structures.sequences.ProteinSequences class method*), 170
from_list() (*aide_predict.utils.data_structures.sequences.ProteinSequencesOnFile class method*), 176
from_pdb() (*aide_predict.utils.data_structures.sequences.ProteinSequence class method*), 160

G

gamma (*aide_predict.utils.badass.BADASSOptimizerParams attribute*), 186
get_alignment_mapping() (*aide_predict.utils.data_structures.sequences.ProteinSequences method*), 170
get_alignment_mapping() (*aide_predict.utils.data_structures.sequences.ProteinSequencesOnFile method*), 177
get_available_structures() (*aide_predict.utils.data_structures.structures.StructureMapper method*), 182
get_chain() (*aide_predict.utils.data_structures.structures.ProteinStructure method*), 180
get_dssp() (*aide_predict.utils.data_structures.structures.ProteinStructure method*), 180
get_feature_names_out() (*aide_predict.bespoke_models.base.PositionSpecificMixin method*), 145
get_feature_names_out() (*aide_predict.bespoke_models.base.ProteinModelWrapper method*), 148
get_feature_names_out() (*aide_predict.bespoke_models.embedders.esm2.ESM2Embedding method*), 59
get_feature_names_out() (*aide_predict.bespoke_models.embedders.kmer.KmerEmbedding method*), 65
get_feature_names_out() (*aide_predict.bespoke_models.embedders.msa_transformer.MSAT method*), 69
get_feature_names_out() (*aide_predict.bespoke_models.embedders.ohe.OneHotAlignedEm method*), 74
get_feature_names_out() (*aide_predict.bespoke_models.embedders.ohe.OneHotProteinEm method*), 78
get_feature_names_out() (*aide_predict.bespoke_models.embedders.saprot.SaProtEmbeddi method*), 84
get_feature_names_out() (*aide_predict.bespoke_models.predictors.esm2.ESM2LikelihoodW method*), 90
get_feature_names_out() (*aide_predict.bespoke_models.predictors.eve.EVEWrapper method*), 97
get_feature_names_out() (*aide_predict.bespoke_models.predictors.evmutation.EVMutation method*), 102
get_feature_names_out() (*aide_predict.bespoke_models.predictors.hmm.HMMWrapper method*), 109
get_feature_names_out() (*aide_predict.bespoke_models.predictors.msa_transformer.MSAT method*), 116
get_feature_names_out() (*aide_predict.bespoke_models.predictors.pretrained_transformer. method*), 123
get_feature_names_out() (*aide_predict.bespoke_models.predictors.saprot.SaProtLikelihood method*), 129
get_feature_names_out() (*aide_predict.bespoke_models.predictors.ssemb.SSEmbWrapper method*), 135
get_feature_names_out() (*aide_predict.bespoke_models.predictors.vespa.VESPAWrapper method*), 140
get_fitted_attributes() (*aide_predict.bespoke_models.base.CacheMixin method*), 144
get_fitted_attributes() (*aide_predict.bespoke_models.embedders.esm2.ESM2Embedding method*), 59
get_fitted_attributes() (*aide_predict.bespoke_models.embedders.msa_transformer.MSAT

method), 69
get_fitted_attributes() (*aide_predict.bespoke_models.embedders.saprot.SaProtEmbedding method*), 84
get_fitted_attributes() (*aide_predict.bespoke_models.predictors.esm2.ESM2LikelihoodWrapper method*), 90
get_fitted_attributes() (*aide_predict.bespoke_models.predictors.evmutation.EVMutationWrapper method*), 103
get_fitted_attributes() (*aide_predict.bespoke_models.predictors.msa_transformer.MSATransformerLikelihoodWrapper method*), 116
get_fitted_attributes() (*aide_predict.bespoke_models.predictors.pretrained_transformers.LikelihoodTransformerBase method*), 123
get_fitted_attributes() (*aide_predict.bespoke_models.predictors.saprot.SaProtLikelihoodWrapper method*), 129
get_fitted_attributes() (*aide_predict.bespoke_models.predictors.vespa.VESPAWrapper method*), 140
get_id_mapping() (*aide_predict.utils.data_structures.sequences.ProteinSequences method*), 170
get_id_mapping() (*aide_predict.utils.data_structures.sequences.ProteinSequencesOnFile method*), 177
get_instance() (*aide_predict.bespoke_models.predictors.pretrained_transformers.ModelDeviceManager class method*), 127
get_metadata_routing() (*aide_predict.bespoke_models.base.ProteinModelWrapper method*), 148
get_metadata_routing() (*aide_predict.bespoke_models.embedders.esm2.ESM2Embedding method*), 59
get_metadata_routing() (*aide_predict.bespoke_models.embedders.kmer.KmerEmbedding method*), 63
get_metadata_routing() (*aide_predict.bespoke_models.embedders.msa_transformer.MSATransformerEmbedding method*), 69
get_metadata_routing() (*aide_predict.bespoke_models.embedders.ohe.OneHotAlignedEmbedding method*), 74
get_metadata_routing() (*aide_predict.bespoke_models.embedders.ohe.OneHotProteinEmbedding method*), 79
get_metadata_routing() (*aide_predict.bespoke_models.embedders.saprot.SaProtEmbedding method*), 84
get_metadata_routing() (*aide_predict.bespoke_models.predictors.esm2.ESM2LikelihoodWrapper method*), 90
get_metadata_routing() (*aide_predict.bespoke_models.predictors.eve.EVEWrapper method*), 97
get_metadata_routing() (*aide_predict.bespoke_models.predictors.evmutation.EVMutation method*), 103
get_metadata_routing() (*aide_predict.bespoke_models.predictors.hmm.HMMWrapper method*), 109
get_metadata_routing() (*aide_predict.bespoke_models.predictors.msa_transformer.MSAT method*), 116
get_metadata_routing() (*aide_predict.bespoke_models.predictors.pretrained_transformer method*), 123
get_metadata_routing() (*aide_predict.bespoke_models.predictors.saprot.SaProtLikelihood method*), 129
get_metadata_routing() (*aide_predict.bespoke_models.predictors.ssemb.SSEmbWrapper method*), 135
get_metadata_routing() (*aide_predict.bespoke_models.predictors.vespa.VESPAWrapper method*), 140
get_most_populated_chunk() (*aide_predict.utils.msa.MSAProcessing method*), 192
get_mutations() (*aide_predict.utils.data_structures.sequences.ProteinSe method*), 160
get_params() (*aide_predict.bespoke_models.base.ProteinModelWrapper method*), 148
get_params() (*aide_predict.bespoke_models.embedders.esm2.ESM2Embe method*), 59
get_params() (*aide_predict.bespoke_models.embedders.kmer.KmerEmbe method*), 64
get_params() (*aide_predict.bespoke_models.embedders.msa_transformer method*), 69
get_params() (*aide_predict.bespoke_models.embedders.ohe.OneHotAlign method*), 74
get_params() (*aide_predict.bespoke_models.embedders.ohe.OneHotProte method*), 79
get_params() (*aide_predict.bespoke_models.embedders.saprot.SaProtEm method*), 84
get_params() (*aide_predict.bespoke_models.predictors.esm2.ESM2Likeli method*), 90
get_params() (*aide_predict.bespoke_models.predictors.eve.EVEWrapper method*), 97
get_params() (*aide_predict.bespoke_models.predictors.evmutation.EVMu method*), 103
get_params() (*aide_predict.bespoke_models.predictors.hmm.HMMWrapp method*), 109
get_params() (*aide_predict.bespoke_models.predictors.msa_transformer. method*), 116
get_params() (*aide_predict.bespoke_models.predictors.pretrained_transf method*), 123
get_params() (*aide_predict.bespoke_models.predictors.saprot.SaProtLike*

method), 129

get_params() (*aide_predict.bespoke_models.predictors.ssemb.SSEmbWrapper method*), 135

get_params() (*aide_predict.bespoke_models.predictors.vespa.VESPAWrapper method*), 140

get_plddt() (*aide_predict.utils.data_structures.structures.ProteinStructure method*), 180

get_protein_character() (*aide_predict.utils.data_structures.sequences.ProteinSequence method*), 161

get_protein_sequences() (*aide_predict.utils.data_structures.structures.StructureMapper method*), 182

get_residue_positions() (*aide_predict.utils.data_structures.structures.ProteinStructure method*), 180

get_sequence() (*aide_predict.utils.data_structures.structures.ProteinStructure method*), 181

get_structure() (*aide_predict.utils.data_structures.structures.ProteinStructure method*), 181

get_structure_tokens() (*in module aide_predict.bespoke_models.predictors.saprot*), 133

get_supported_tools() (*in module aide_predict.utils.checks*), 187

H

has_gaps (*aide_predict.utils.data_structures.sequences.ProteinSequence property*), 161

has_gaps (*aide_predict.utils.data_structures.sequences.ProteinSequences property*), 171

has_gaps (*aide_predict.utils.data_structures.sequences.ProteinSequencesOnFile property*), 177

has_lower() (*aide_predict.utils.data_structures.sequences.ProteinSequences method*), 171

has_lower() (*aide_predict.utils.data_structures.sequences.ProteinSequencesOnFile method*), 177

has_msa (*aide_predict.utils.data_structures.sequences.ProteinSequence property*), 161

has_non_canonical (*aide_predict.utils.data_structures.sequences.ProteinSequence property*), 161

has_structure (*aide_predict.utils.data_structures.sequences.ProteinSequence property*), 161

HMMWrapper (*class in aide_predict.bespoke_models.predictors.hmm*), 107

I

id (*aide_predict.utils.data_structures.sequences.ProteinSequence property*), 161

id_mapping (*aide_predict.utils.data_structures.sequences.ProteinSequences property*), 171

id_mapping (*aide_predict.utils.data_structures.sequences.ProteinSequencesOnFile property*), 177

ids (*aide_predict.utils.data_structures.sequences.ProteinSequences property*), 171

ids (*aide_predict.utils.data_structures.sequences.ProteinSequencesOnFile property*), 177

index() (*aide_predict.utils.data_structures.sequences.ProteinCharacter method*), 154

index() (*aide_predict.utils.data_structures.sequences.ProteinSequence method*), 161

index() (*aide_predict.utils.data_structures.sequences.ProteinSequences method*), 171

index() (*aide_predict.utils.data_structures.sequences.ProteinSequencesOn*[illegible] *method*), 177

init_score_batch_size (*aide_predict.utils.badass.BADASSOptimizerParams attribute*), 186

insert() (*aide_predict.utils.data_structures.sequences.ProteinSequences method*), 171

insert() (*aide_predict.utils.data_structures.sequences.ProteinSequencesO*[illegible] *method*), 177

inverse_transform() (*aide_predict.bespoke_models.embedders.ohe.OneHotProteinEm*[illegible] *method*), 79

is_gap (*aide_predict.utils.data_structures.sequences.ProteinCharacter property*), 155

is_in_msa (*aide_predict.utils.data_structures.sequences.ProteinSequence property*), 161

is_jsonable() (*in module aide_predict.bespoke_models.base*), 152

is_non_canonical (*aide_predict.utils.data_structures.sequences.ProteinC*[illegible] *property*), 155

is_not_focus (*aide_predict.utils.data_structures.sequences.ProteinChara*[illegible] *property*), 155

isalnum() (*aide_predict.utils.data_structures.sequences.ProteinCharacter method*), 155

isalnum() (*aide_predict.utils.data_structures.sequences.ProteinSequence method*), 161

isalpha() (*aide_predict.utils.data_structures.sequences.ProteinCharacter method*), 155

isalpha() (*aide_predict.utils.data_structures.sequences.ProteinSequence method*), 161

isascii() (*aide_predict.utils.data_structures.sequences.ProteinCharacter method*), 155

isascii() (*aide_predict.utils.data_structures.sequences.ProteinSequence method*), 161

isdecimal() (*aide_predict.utils.data_structures.sequences.ProteinCharac*[illegible] *method*), 155

isdecimal() (*aide_predict.utils.data_structures.sequences.ProteinSequen*[illegible] *method*), 161

isdigit() (*aide_predict.utils.data_structures.sequences.ProteinCharacter method*), 155

isdigit() (*aide_predict.utils.data_structures.sequences.ProteinSequence method*), 162

isidentifier() (*aide_predict.utils.data_structures.sequences.ProteinCha*[illegible] *method*), 155

isidentifier() (*aide_predict.utils.data_structures.sequences.ProteinSequence method*), 162
islower() (*aide_predict.utils.data_structures.sequences.ProteinCharacter method*), 155
islower() (*aide_predict.utils.data_structures.sequences.ProteinSequence method*), 162
isnumeric() (*aide_predict.utils.data_structures.sequences.ProteinCharacter method*), 155
isnumeric() (*aide_predict.utils.data_structures.sequences.ProteinSequence method*), 162
isprintable() (*aide_predict.utils.data_structures.sequences.ProteinCharacter method*), 155
isprintable() (*aide_predict.utils.data_structures.sequences.ProteinSequence method*), 162
isspace() (*aide_predict.utils.data_structures.sequences.ProteinCharacter method*), 156
isspace() (*aide_predict.utils.data_structures.sequences.ProteinSequence method*), 162
istitle() (*aide_predict.utils.data_structures.sequences.ProteinCharacter method*), 156
istitle() (*aide_predict.utils.data_structures.sequences.ProteinSequence method*), 162
isupper() (*aide_predict.utils.data_structures.sequences.ProteinCharacter method*), 156
isupper() (*aide_predict.utils.data_structures.sequences.ProteinSequence method*), 162
iter_batches() (*aide_predict.utils.data_structures.sequences.ProteinSequences method*), 171
iter_batches() (*aide_predict.utils.data_structures.sequences.ProteinSequencesOnFile method*), 177
iter_protein_characters() (*aide_predict.utils.data_structures.sequences.ProteinSequence method*), 162

J

join() (*aide_predict.utils.data_structures.sequences.ProteinCharacter method*), 156
join() (*aide_predict.utils.data_structures.sequences.ProteinSequence method*), 162

K

KmerEmbedding (*class in aide_predict.bespoke_models.embedders.kmer*), 62

L

LikelihoodTransformerBase (*class in aide_predict.bespoke_models.predictors.pretrained_transformers*), 120
ljust() (*aide_predict.utils.data_structures.sequences.ProteinCharacter method*), 156
ljust() (*aide_predict.utils.data_structures.sequences.ProteinSequence method*), 163
lower() (*aide_predict.utils.data_structures.sequences.ProteinCharacter method*), 156
lower() (*aide_predict.utils.data_structures.sequences.ProteinSequence method*), 163
lstrip() (*aide_predict.utils.data_structures.sequences.ProteinCharacter method*), 156
lstrip() (*aide_predict.utils.data_structures.sequences.ProteinSequence method*), 163

M

mafft_align() (*in module aide_predict.utils.alignment_calls*), 182
main() (*in module aide_predict.utils.mmseqs_msa_search*), 190
main() (*in module aide_predict.utils.soloseq*), 195
maketrans() (*aide_predict.utils.data_structures.sequences.ProteinCharac static method*), 156
maketrans() (*aide_predict.utils.data_structures.sequences.ProteinSequen static method*), 163
MarginalMethod (*class in aide_predict.bespoke_models.predictors.pretrained_transformers*), 127
MASKED (*aide_predict.bespoke_models.predictors.pretrained_transformers.I attribute*), 127
MessageBool (*class in aide_predict.utils.common*), 187
metadata_folder (*aide_predict.bespoke_models.base.ProteinModelWrap property*), 149
metadata_folder (*aide_predict.bespoke_models.embedders.esm2.ESM2E property*), 59
metadata_folder (*aide_predict.bespoke_models.embedders.kmer.KmerE property*), 64
metadata_folder (*aide_predict.bespoke_models.embedders.msa_transfor property*), 69
metadata_folder (*aide_predict.bespoke_models.embedders.ohe.OneHotA property*), 74
metadata_folder (*aide_predict.bespoke_models.embedders.ohe.OneHotF property*), 79
metadata_folder (*aide_predict.bespoke_models.embedders.saprot.SaPro property*), 84
metadata_folder (*aide_predict.bespoke_models.predictors.esm2.ESM2L property*), 90
metadata_folder (*aide_predict.bespoke_models.predictors.eve.EVEWrap property*), 97
metadata_folder (*aide_predict.bespoke_models.predictors.evmutation.E property*), 103
metadata_folder (*aide_predict.bespoke_models.predictors.hmm.HMMW property*), 109
metadata_folder (*aide_predict.bespoke_models.predictors.msa_transfor property*), 117
metadata_folder (*aide_predict.bespoke_models.predictors.pretrained_tra property*), 123
metadata_folder (*aide_predict.bespoke_models.predictors.saprot.SaProt property*), 130

metadata_folder (*aide_predict.bespoke_models.predictors.ssemb.SSEmbWrapper property*), 135
metadata_folder (*aide_predict.bespoke_models.predictors.vespa.VESPAWrapper property*), 141
model_device_context() (*in module aide_predict.bespoke_models.predictors.pretrained_transformers*), 127
model_on_device() (*aide_predict.bespoke_models.predictors.pretrained_transformers.ModelDeviceManager method*), 127
ModelDeviceManager (*class in aide_predict.bespoke_models.predictors.pretrained_transformers*), 127
module
- aide_predict, 196
- aide_predict.bespoke_models, 152
- aide_predict.bespoke_models.base, 144
- aide_predict.bespoke_models.embedders, 87
- aide_predict.bespoke_models.embedders.esm2, 57
- aide_predict.bespoke_models.embedders.kmer, 62
- aide_predict.bespoke_models.embedders.msa_transformer, 66
- aide_predict.bespoke_models.embedders.ohe, 72
- aide_predict.bespoke_models.embedders.saprot, 82
- aide_predict.bespoke_models.predictors, 144
- aide_predict.bespoke_models.predictors.esm2, 87
- aide_predict.bespoke_models.predictors.eve, 94
- aide_predict.bespoke_models.predictors.evmutation, 101
- aide_predict.bespoke_models.predictors.hmm, 106
- aide_predict.bespoke_models.predictors.msa_transformer, 113
- aide_predict.bespoke_models.predictors.pretrained_transformers, 120
- aide_predict.bespoke_models.predictors.saprot, 128
- aide_predict.bespoke_models.predictors.ssemb, 133
- aide_predict.bespoke_models.predictors.vespa, 139
- aide_predict.io, 153
- aide_predict.io.bio_files, 152
- aide_predict.patches_, 196
- aide_predict.utils, 196
- aide_predict.utils.alignment_calls, 182
- aide_predict.utils.badass, 184
- aide_predict.utils.checks, 187
- aide_predict.utils.common, 187
- aide_predict.utils.conservation, 188
- aide_predict.utils.constants, 190
- aide_predict.utils.data_structures, 182
- aide_predict.utils.data_structures.sequences, 153
- aide_predict.utils.data_structures.structures, 179
- aide_predict.utils.mmseqs_msa_search, 190
- aide_predict.utils.msa, 191
- aide_predict.utils.plotting, 194
- aide_predict.utils.soloseq, 195

msa (*aide_predict.utils.data_structures.sequences.ProteinSequence property*), 163
msa_process() (*aide_predict.utils.data_structures.sequences.ProteinSequ method*), 171
msa_process() (*aide_predict.utils.data_structures.sequences.ProteinSequ method*), 178
msa_same_width (*aide_predict.utils.data_structures.sequences.ProteinSeq property*), 163
MSAProcessing (*class in aide_predict.utils.msa*), 191
MSATransformerEmbedding (*class in aide_predict.bespoke_models.embedders.msa_transformer*), 66
MSATransformerLikelihoodWrapper (*class in aide_predict.bespoke_models.predictors.msa_transformer*), 113
MUTANT (*aide_predict.bespoke_models.predictors.pretrained_transformers.I attribute*), 127
mutate() (*aide_predict.utils.data_structures.sequences.ProteinSequence method*), 163
mutated_positions (*aide_predict.utils.data_structures.sequences.Protein property*), 171
mutated_positions (*aide_predict.utils.data_structures.sequences.Protein property*), 178
mutated_positions() (*aide_predict.utils.data_structures.sequences.ProteinSequence method*), 163

N

n_seqs_to_keep (*aide_predict.utils.badass.BADASSOptimizerParams attribute*), 186
normalize_scores (*aide_predict.utils.badass.BADASSOptimizerParams attribute*), 186
num_gaps (*aide_predict.utils.data_structures.sequences.ProteinSequence property*), 163
num_iter (*aide_predict.utils.badass.BADASSOptimizerParams attribute*), 186
num_mutations (*aide_predict.utils.badass.BADASSOptimizerParams attribute*), 186

O

OneHotAlignedEmbedding (*class in aide_predict.bespoke_models.embedders.ohe*),

72
OneHotProteinEmbedding (*class in aide_predict.bespoke_models.embedders.ohe*), 77
optimize() (*aide_predict.utils.badass.BADASSOptimizer method*), 184

P

partial_fit() (*aide_predict.bespoke_models.base.ProteinModelWrapper method*), 149
partial_fit() (*aide_predict.bespoke_models.embedders.esm2.ESM2Embedding method*), 59
partial_fit() (*aide_predict.bespoke_models.embedders.kmer.KmerEmbedding method*), 64
partial_fit() (*aide_predict.bespoke_models.embedders.msa_transformer.MSATransformerEmbedding method*), 70
partial_fit() (*aide_predict.bespoke_models.embedders.ohe.OneHotAlignedEmbedding method*), 74
partial_fit() (*aide_predict.bespoke_models.embedders.ohe.OneHotProteinEmbedding method*), 79
partial_fit() (*aide_predict.bespoke_models.embedders.saprot.SaProtEmbedding method*), 84
partial_fit() (*aide_predict.bespoke_models.predictors.esm2.ESM2LikelihoodWrapper method*), 90
partial_fit() (*aide_predict.bespoke_models.predictors.eve.EVEWrapper method*), 97
partial_fit() (*aide_predict.bespoke_models.predictors.evmutation.EVMutationWrapper method*), 103
partial_fit() (*aide_predict.bespoke_models.predictors.hmm.HMMWrapper method*), 109
partial_fit() (*aide_predict.bespoke_models.predictors.msa_transformer.MSATransformerLikelihoodWrapper method*), 117
partial_fit() (*aide_predict.bespoke_models.predictors.pretrained_transformers.LikelihoodTransformerBase method*), 124
partial_fit() (*aide_predict.bespoke_models.predictors.saprot.SaProtLikelihoodWrapper method*), 130
partial_fit() (*aide_predict.bespoke_models.predictors.ssemb.SSEmbWrapper method*), 135
partial_fit() (*aide_predict.bespoke_models.predictors.vespa.VESPAWrapper method*), 141
partition() (*aide_predict.utils.data_structures.sequences.ProteinCharacter method*), 156
partition() (*aide_predict.utils.data_structures.sequences.ProteinSequence method*), 164
patch_pandas_append() (*in module aide_predict.patches_*), 196
patched_parse_plmc_log() (*in module aide_predict.patches_*), 196
pdb_file (*aide_predict.utils.data_structures.structures.ProteinStructure attribute*), 181
per_position_capable (*aide_predict.bespoke_models.base.ProteinModelWrapper property*), 149
per_position_capable (*aide_predict.bespoke_models.embedders.esm2.ESM2Embedding property*), 60
per_position_capable (*aide_predict.bespoke_models.embedders.kmer.KmerEmbedding property*), 64
per_position_capable (*aide_predict.bespoke_models.embedders.msa_transformer.MSAT property*), 70
per_position_capable (*aide_predict.bespoke_models.embedders.ohe.OneHotAlignedEm property*), 75
per_position_capable (*aide_predict.bespoke_models.embedders.ohe.OneHotProteinEm property*), 79
per_position_capable (*aide_predict.bespoke_models.embedders.saprot.SaProtEmbeddin property*), 84
per_position_capable (*aide_predict.bespoke_models.predictors.esm2.ESM2LikelihoodW property*), 91
per_position_capable (*aide_predict.bespoke_models.predictors.eve.EVEWrapper property*), 98
per_position_capable (*aide_predict.bespoke_models.predictors.evmutation.EVMutation property*), 103
per_position_capable (*aide_predict.bespoke_models.predictors.hmm.HMMWrapper property*), 110
per_position_capable (*aide_predict.bespoke_models.predictors.msa_transformer.MSAT property*), 117
per_position_capable (*aide_predict.bespoke_models.predictors.pretrained_transformer. property*), 124
per_position_capable (*aide_predict.bespoke_models.predictors.saprot.SaProtLikelihooc property*), 130
per_position_capable (*aide_predict.bespoke_models.predictors.ssemb.SSEmbWrapper property*), 136
per_position_capable (*aide_predict.bespoke_models.predictors.vespa.VESPAWrapper property*), 141
plddt_file (*aide_predict.utils.data_structures.structures.ProteinStructure attribute*), 181
plot() (*aide_predict.utils.badass.BADASSOptimizer method*), 185
plot_conservation() (*in module aide_predict.utils.plotting*), 194
plot_mutation_heatmap() (*in module aide_predict.utils.plotting*), 194
plot_protein_sequence_heatmap() (*in module*

aide_predict.utils.plotting), 194
pop() (*aide_predict.utils.data_structures.sequences.ProteinSequences method*), 171
pop() (*aide_predict.utils.data_structures.sequences.ProteinSequencesOnFile method*), 178
PositionSpecificMixin (*class in aide_predict.bespoke_models.base*), 145
predict() (*aide_predict.bespoke_models.base.ProteinModelWrapper method*), 149
predict() (*aide_predict.bespoke_models.embedders.esm2.ESM2Embedding method*), 60
predict() (*aide_predict.bespoke_models.embedders.kmer.KmerEmbedding method*), 64
predict() (*aide_predict.bespoke_models.embedders.msa_transformer.MSATransformerEmbedding method*), 70
predict() (*aide_predict.bespoke_models.embedders.ohe.OneHotAlignedEmbedding method*), 75
predict() (*aide_predict.bespoke_models.embedders.ohe.OneHotProteinEmbedding method*), 79
predict() (*aide_predict.bespoke_models.embedders.saprot.SaProtEmbedding method*), 84
predict() (*aide_predict.bespoke_models.predictors.esm2.ESM2LikelihoodWrapper method*), 91
predict() (*aide_predict.bespoke_models.predictors.eve.EVEWrapper method*), 98
predict() (*aide_predict.bespoke_models.predictors.evmutation.EVMutationWrapper method*), 103
predict() (*aide_predict.bespoke_models.predictors.hmm.HMMWrapper method*), 110
predict() (*aide_predict.bespoke_models.predictors.msa_transformer.MSATransformerLikelihoodWrapper method*), 117
predict() (*aide_predict.bespoke_models.predictors.pretrained_transformers.LikelihoodTransformerBase method*), 124
predict() (*aide_predict.bespoke_models.predictors.saprot.SaProtLikelihoodWrapper method*), 130
predict() (*aide_predict.bespoke_models.predictors.ssemb.SSEmbWrapper method*), 136
predict() (*aide_predict.bespoke_models.predictors.vespa.VESPAWrapper method*), 141
process() (*aide_predict.utils.msa.MSAProcessing method*), 193
PROPERTIES (*aide_predict.utils.conservation.ConservationAnalysis attribute*), 188
ProteinCharacter (*class in aide_predict.utils.data_structures.sequences*), 153
ProteinModelWrapper (*class in aide_predict.bespoke_models.base*), 145
ProteinSequence (*class in aide_predict.utils.data_structures.sequences*), 158
ProteinSequences (*class in aide_predict.utils.data_structures.sequences*), 166
ProteinSequencesOnFile (*class in aide_predict.utils.data_structures.sequences*), 173
ProteinStructure (*class in aide_predict.utils.data_structures.structures*), 179

R

read_a3m() (*in module aide_predict.io.bio_files*), 152
read_fasta() (*in module aide_predict.io.bio_files*), 153
remove() (*aide_predict.utils.data_structures.sequences.ProteinSequences method*), 171
remove() (*aide_predict.utils.data_structures.sequences.ProteinSequencesO[illegible] method*), 178
remove_gappy_columns() (*aide_predict.utils.msa.MSAProcessing method*), 193
removeprefix() (*aide_predict.utils.data_structures.sequences.ProteinCha[illegible] method*), 156
removeprefix() (*aide_predict.utils.data_structures.sequences.ProteinSeq[illegible] method*), 164
removesuffix() (*aide_predict.utils.data_structures.sequences.ProteinCha[illegible] method*), 157
removesuffix() (*aide_predict.utils.data_structures.sequences.ProteinSeq[illegible] method*), 164
replace() (*aide_predict.utils.data_structures.sequences.ProteinCharacter[illegible] method*), 157
replace() (*aide_predict.utils.data_structures.sequences.ProteinSequence method*), 164
requires_fixed_length (*aide_predict.bespoke_models.base.ProteinModelWrapper property*), 149
requires_fixed_length (*aide_predict.bespoke_models.embedders.esm2.ESM2Embedding[illegible] property*), 60
requires_fixed_length (*aide_predict.bespoke_models.embedders.kmer.KmerEmbedding property*), 64
requires_fixed_length (*aide_predict.bespoke_models.embedders.msa_transformer.MSAT[illegible] property*), 70
requires_fixed_length (*aide_predict.bespoke_models.embedders.ohe.OneHotAlignedEm[illegible] property*), 75
requires_fixed_length (*aide_predict.bespoke_models.embedders.ohe.OneHotProteinEm[illegible] property*), 80
requires_fixed_length (*aide_predict.bespoke_models.embedders.saprot.SaProtEmbeddi[illegible] property*), 85
requires_fixed_length (*aide_predict.bespoke_models.predictors.esm2.ESM2LikelihoodW[illegible] property*), 91
requires_fixed_length

(*aide_predict.bespoke_models.predictors.eve.EVEWrapper property*), 98

requires_fixed_length (*aide_predict.bespoke_models.predictors.evmutation.EVMutationWrapper property*), 104

requires_fixed_length (*aide_predict.bespoke_models.predictors.hmm.HMMWrapper property*), 110

requires_fixed_length (*aide_predict.bespoke_models.predictors.msa_transformer.MSATransformerLikelihoodWrapper property*), 117

requires_fixed_length (*aide_predict.bespoke_models.predictors.pretrained_transformers.LikelihoodTransformerBase property*), 124

requires_fixed_length (*aide_predict.bespoke_models.predictors.saprot.SaProtLikelihoodWrapper property*), 130

requires_fixed_length (*aide_predict.bespoke_models.predictors.ssemb.SSEmbWrapper property*), 136

requires_fixed_length (*aide_predict.bespoke_models.predictors.vespa.VESPAWrapper property*), 141

requires_msa_for_fit (*aide_predict.bespoke_models.base.ProteinModelWrapper property*), 149

requires_msa_for_fit (*aide_predict.bespoke_models.embedders.esm2.ESM2Embedding property*), 60

requires_msa_for_fit (*aide_predict.bespoke_models.embedders.kmer.KmerEmbedding property*), 64

requires_msa_for_fit (*aide_predict.bespoke_models.embedders.msa_transformer.MSATransformerEmbedding property*), 70

requires_msa_for_fit (*aide_predict.bespoke_models.embedders.ohe.OneHotAlignedEmbedding property*), 75

requires_msa_for_fit (*aide_predict.bespoke_models.embedders.ohe.OneHotProteinEmbedding property*), 80

requires_msa_for_fit (*aide_predict.bespoke_models.embedders.saprot.SaProtEmbedding property*), 85

requires_msa_for_fit (*aide_predict.bespoke_models.predictors.esm2.ESM2LikelihoodWrapper property*), 91

requires_msa_for_fit (*aide_predict.bespoke_models.predictors.eve.EVEWrapper property*), 98

requires_msa_for_fit (*aide_predict.bespoke_models.predictors.evmutation.EVMutationWrapper property*), 104

requires_msa_for_fit (*aide_predict.bespoke_models.predictors.hmm.HMMWrapper property*), 110

requires_msa_for_fit (*aide_predict.bespoke_models.predictors.msa_transformer.MSAT property*), 117

requires_msa_for_fit (*aide_predict.bespoke_models.predictors.pretrained_transformers property*), 124

requires_msa_for_fit (*aide_predict.bespoke_models.predictors.saprot.SaProtLikelihood property*), 130

requires_msa_for_fit (*aide_predict.bespoke_models.predictors.ssemb.SSEmbWrapper property*), 136

requires_msa_for_fit (*aide_predict.bespoke_models.predictors.vespa.VESPAWrapper property*), 141

requires_msa_per_sequence (*aide_predict.bespoke_models.base.ProteinModelWrapper property*), 149

requires_msa_per_sequence (*aide_predict.bespoke_models.embedders.esm2.ESM2Embedding property*), 60

requires_msa_per_sequence (*aide_predict.bespoke_models.embedders.kmer.KmerEmbedding property*), 64

requires_msa_per_sequence (*aide_predict.bespoke_models.embedders.msa_transformer.MSAT property*), 70

requires_msa_per_sequence (*aide_predict.bespoke_models.embedders.ohe.OneHotAlignedEm property*), 75

requires_msa_per_sequence (*aide_predict.bespoke_models.embedders.ohe.OneHotProteinEm property*), 80

requires_msa_per_sequence (*aide_predict.bespoke_models.embedders.saprot.SaProtEmbeddin property*), 85

requires_msa_per_sequence (*aide_predict.bespoke_models.predictors.esm2.ESM2LikelihoodW property*), 91

requires_msa_per_sequence (*aide_predict.bespoke_models.predictors.eve.EVEWrapper property*), 98

requires_msa_per_sequence (*aide_predict.bespoke_models.predictors.evmutation.EVMutation property*), 104

requires_msa_per_sequence (*aide_predict.bespoke_models.predictors.hmm.HMMWrapper property*), 110

requires_msa_per_sequence (*aide_predict.bespoke_models.predictors.msa_transformer.MSAT property*), 117

requires_msa_per_sequence

(*aide_predict.bespoke_models.predictors.pretrained_transformers.LikelihoodTransformerBase property*), 124
requires_msa_per_sequence (*aide_predict.bespoke_models.predictors.saprot.SaProtLikelihoodWrapper property*), 130
requires_msa_per_sequence (*aide_predict.bespoke_models.predictors.ssemb.SSEmbWrapper property*), 136
requires_msa_per_sequence (*aide_predict.bespoke_models.predictors.vespa.VESPAWrapper property*), 141
requires_structure (*aide_predict.bespoke_models.base.ProteinModelWrapper property*), 149
requires_structure (*aide_predict.bespoke_models.embedders.esm2.ESM2Embedding property*), 60
requires_structure (*aide_predict.bespoke_models.embedders.kmer.KmerEmbedding property*), 65
requires_structure (*aide_predict.bespoke_models.embedders.msa_transformer.MSATransformerEmbedding property*), 70
requires_structure (*aide_predict.bespoke_models.embedders.ohe.OneHotAlignedEmbedding property*), 75
requires_structure (*aide_predict.bespoke_models.embedders.ohe.OneHotProteinEmbedding property*), 80
requires_structure (*aide_predict.bespoke_models.embedders.saprot.SaProtEmbedding property*), 85
requires_structure (*aide_predict.bespoke_models.predictors.esm2.ESM2LikelihoodWrapper property*), 91
requires_structure (*aide_predict.bespoke_models.predictors.eve.EVEWrapper property*), 98
requires_structure (*aide_predict.bespoke_models.predictors.evmutation.EVMutationWrapper property*), 104
requires_structure (*aide_predict.bespoke_models.predictors.hmm.HMMWrapper property*), 110
requires_structure (*aide_predict.bespoke_models.predictors.msa_transformer.MSATransformerLikelihoodWrapper property*), 117
requires_structure (*aide_predict.bespoke_models.predictors.pretrained_transformers.LikelihoodTransformerBase property*), 124
requires_structure (*aide_predict.bespoke_models.predictors.saprot.SaProtLikelihoodWrapper property*), 130
requires_structure (*aide_predict.bespoke_models.predictors.ssemb.SSEmbWrapper property*), 136
requires_structure (*aide_predict.bespoke_models.predictors.vespa.VESPAWrapper property*), 141
requires_wt_during_inference (*aide_predict.bespoke_models.base.ProteinModelWrapper property*), 149
requires_wt_during_inference (*aide_predict.bespoke_models.embedders.esm2.ESM2Embedding property*), 60
requires_wt_during_inference (*aide_predict.bespoke_models.embedders.kmer.KmerEmbedding property*), 65
requires_wt_during_inference (*aide_predict.bespoke_models.embedders.msa_transformer.MSATransformerEmbedding property*), 70
requires_wt_during_inference (*aide_predict.bespoke_models.embedders.ohe.OneHotAlignedEm property*), 75
requires_wt_during_inference (*aide_predict.bespoke_models.embedders.ohe.OneHotProteinEm property*), 80
requires_wt_during_inference (*aide_predict.bespoke_models.embedders.saprot.SaProtEmbeddi property*), 85
requires_wt_during_inference (*aide_predict.bespoke_models.predictors.esm2.ESM2LikelihoodW property*), 91
requires_wt_during_inference (*aide_predict.bespoke_models.predictors.eve.EVEWrapper property*), 98
requires_wt_during_inference (*aide_predict.bespoke_models.predictors.evmutation.EVMutation property*), 104
requires_wt_during_inference (*aide_predict.bespoke_models.predictors.hmm.HMMWrapper property*), 110
requires_wt_during_inference (*aide_predict.bespoke_models.predictors.msa_transformer.MSAT property*), 117
requires_wt_during_inference (*aide_predict.bespoke_models.predictors.pretrained_transformer property*), 124
requires_wt_during_inference (*aide_predict.bespoke_models.predictors.saprot.SaProtLikelihoo property*), 130
requires_wt_during_inference (*aide_predict.bespoke_models.predictors.ssemb.SSEmbWrapper property*), 136
requires_wt_during_inference (*aide_predict.bespoke_models.predictors.vespa.VESPAWrapper property*), 141
requires_wt_msa (*aide_predict.bespoke_models.base.ProteinModelWrap property*), 150
requires_wt_msa (*aide_predict.bespoke_models.embedders.esm2.ESM2E property*), 60
requires_wt_msa (*aide_predict.bespoke_models.embedders.kmer.KmerEr property*), 65
requires_wt_msa (*aide_predict.bespoke_models.embedders.msa_transfor property*), 70
requires_wt_msa (*aide_predict.bespoke_models.embedders.ohe.OneHotA property*), 75
requires_wt_msa (*aide_predict.bespoke_models.embedders.ohe.OneHotI property*), 80
requires_wt_msa (*aide_predict.bespoke_models.embedders.saprot.SaPro property*), 85
requires_wt_msa (*aide_predict.bespoke_models.predictors.esm2.ESM2Li property*), 91
requires_wt_msa (*aide_predict.bespoke_models.predictors.eve.EVEWrap*

property), 98
`requires_wt_msa` (*aide_predict.bespoke_models.predictors.evmutation.EVMutationWrapper property*), 104
`requires_wt_msa` (*aide_predict.bespoke_models.predictors.hmm.HMMWrapper property*), 110
`requires_wt_msa` (*aide_predict.bespoke_models.predictors.msa_transformer.MSATransformerLikelihoodWrapper property*), 117
`requires_wt_msa` (*aide_predict.bespoke_models.predictors.pretrained_transformers.LikelihoodTransformerBase property*), 124
`requires_wt_msa` (*aide_predict.bespoke_models.predictors.saprot.SaProtLikelihoodWrapper property*), 131
`requires_wt_msa` (*aide_predict.bespoke_models.predictors.ssemb.SSEmbWrapper property*), 136
`requires_wt_msa` (*aide_predict.bespoke_models.predictors.vespa.VESPAWrapper property*), 141
`requires_wt_to_function` (*aide_predict.bespoke_models.base.ProteinModelWrapper property*), 150
`requires_wt_to_function` (*aide_predict.bespoke_models.embedders.esm2.ESM2Embedding property*), 60
`requires_wt_to_function` (*aide_predict.bespoke_models.embedders.kmer.KmerEmbedding property*), 65
`requires_wt_to_function` (*aide_predict.bespoke_models.embedders.msa_transformer.MSATransformerEmbedding property*), 70
`requires_wt_to_function` (*aide_predict.bespoke_models.embedders.ohe.OneHotAlignedEmbedding property*), 75
`requires_wt_to_function` (*aide_predict.bespoke_models.embedders.ohe.OneHotProteinEmbedding property*), 80
`requires_wt_to_function` (*aide_predict.bespoke_models.embedders.saprot.SaProtEmbedding property*), 85
`requires_wt_to_function` (*aide_predict.bespoke_models.predictors.esm2.ESM2LikelihoodWrapper property*), 91
`requires_wt_to_function` (*aide_predict.bespoke_models.predictors.eve.EVEWrapper property*), 98
`requires_wt_to_function` (*aide_predict.bespoke_models.predictors.evmutation.EVMutationWrapper property*), 104
`requires_wt_to_function` (*aide_predict.bespoke_models.predictors.hmm.HMMWrapper property*), 110
`requires_wt_to_function` (*aide_predict.bespoke_models.predictors.msa_transformer.MSATransformerLikelihoodWrapper property*), 117
`requires_wt_to_function` (*aide_predict.bespoke_models.predictors.pretrained_transformers.LikelihoodTransformerBase property*), 124
`requires_wt_to_function` (*aide_predict.bespoke_models.predictors.saprot.SaProtLikelihood property*), 131
`requires_wt_to_function` (*aide_predict.bespoke_models.predictors.ssemb.SSEmbWrapper property*), 136
`requires_wt_to_function` (*aide_predict.bespoke_models.predictors.vespa.VESPAWrapper property*), 141
`RequiresFixedLengthMixin` (*class in aide_predict.bespoke_models.base*), 151
`RequiresMSAForFitMixin` (*class in aide_predict.bespoke_models.base*), 151
`RequiresMSAPerSequenceMixin` (*class in aide_predict.bespoke_models.base*), 151
`RequiresStructureMixin` (*class in aide_predict.bespoke_models.base*), 151
`RequiresWTDuringInferenceMixin` (*class in aide_predict.bespoke_models.base*), 151
`RequiresWTMSAMixin` (*class in aide_predict.bespoke_models.base*), 152
`RequiresWTToFunctionMixin` (*class in aide_predict.bespoke_models.base*), 152
`results` (*aide_predict.utils.badass.BADASSOptimizer property*), 185
`reversal_threshold` (*aide_predict.utils.badass.BADASSOptimizerParam attribute*), 186
`reverse()` (*aide_predict.utils.data_structures.sequences.ProteinSequences method*), 172
`reverse()` (*aide_predict.utils.data_structures.sequences.ProteinSequences method*), 178
`rfind()` (*aide_predict.utils.data_structures.sequences.ProteinCharacter method*), 157
`rfind()` (*aide_predict.utils.data_structures.sequences.ProteinSequence method*), 164
`rindex()` (*aide_predict.utils.data_structures.sequences.ProteinCharacter method*), 157
`rindex()` (*aide_predict.utils.data_structures.sequences.ProteinSequence method*), 164
`rjust()` (*aide_predict.utils.data_structures.sequences.ProteinCharacter method*), 157
`rjust()` (*aide_predict.utils.data_structures.sequences.ProteinSequence method*), 164
`rpartition()` (*aide_predict.utils.data_structures.sequences.ProteinChara method*), 157
`rpartition()` (*aide_predict.utils.data_structures.sequences.ProteinSeque method*), 164
`rsplit()` (*aide_predict.utils.data_structures.sequences.ProteinCharacter method*), 157
`rsplit()` (*aide_predict.utils.data_structures.sequences.ProteinSequence method*), 164
`rstrip()` (*aide_predict.utils.data_structures.sequences.ProteinCharacter method*), 157
`rstrip()` (*aide_predict.utils.data_structures.sequences.ProteinSequence*

method), 165
run_mmseqs_command() (in module aide_predict.utils.mmseqs_msa_search), 190
run_mmseqs_search() (in module aide_predict.utils.mmseqs_msa_search), 190
run_soloseq() (in module aide_predict.utils.soloseq), 195

S

sample() (aide_predict.utils.data_structures.sequences.ProteinSequences method), 172
sample() (aide_predict.utils.data_structures.sequences.ProteinSequencesOnFile method), 178
SaProtEmbedding (class in aide_predict.bespoke_models.embedders.saprot), 82
SaProtLikelihoodWrapper (class in aide_predict.bespoke_models.predictors.saprot), 128
saturation_mutagenesis() (aide_predict.utils.data_structures.sequences.ProteinSequence method), 165
save_results() (aide_predict.utils.badass.BADASSOptimizer method), 185
score() (aide_predict.bespoke_models.base.CanRegressMixin method), 145
score() (aide_predict.bespoke_models.predictors.esm2.ESM2LikelihoodWrapper method), 91
score() (aide_predict.bespoke_models.predictors.eve.EVEWrapper method), 98
score() (aide_predict.bespoke_models.predictors.evmutation.EVMutationWrapper method), 104
score() (aide_predict.bespoke_models.predictors.hmm.HMMWrapper method), 110
score() (aide_predict.bespoke_models.predictors.msa_transformer.MSATransformerLikelihoodWrapper method), 117
score() (aide_predict.bespoke_models.predictors.pretrained_transformers.LikelihoodTransformerBase method), 124
score() (aide_predict.bespoke_models.predictors.saprot.SaProtLikelihoodWrapper method), 131
score() (aide_predict.bespoke_models.predictors.ssemb.SSEmbWrapper method), 136
score() (aide_predict.bespoke_models.predictors.vespa.VESPAWrapper method), 142
score_threshold (aide_predict.utils.badass.BADASSOptimizerParams attribute), 186
seed (aide_predict.utils.badass.BADASSOptimizerParams attribute), 186
seqs_per_iter (aide_predict.utils.badass.BADASSOptimizerParams attribute), 186
set_fit_request() (aide_predict.bespoke_models.base.ProteinModelWrapper method), 150
set_fit_request() (aide_predict.bespoke_models.embedders.esm2.ESM method), 60
set_fit_request() (aide_predict.bespoke_models.embedders.kmer.Kme method), 65
set_fit_request() (aide_predict.bespoke_models.embedders.msa_trans method), 70
set_fit_request() (aide_predict.bespoke_models.embedders.ohe.OneH method), 75
set_fit_request() (aide_predict.bespoke_models.embedders.ohe.OneH method), 80
set_fit_request() (aide_predict.bespoke_models.embedders.saprot.SaP method), 85
set_fit_request() (aide_predict.bespoke_models.predictors.esm2.ESM2 method), 91
set_fit_request() (aide_predict.bespoke_models.predictors.eve.EVEWr method), 98
set_fit_request() (aide_predict.bespoke_models.predictors.evmutation method), 104
set_fit_request() (aide_predict.bespoke_models.predictors.hmm.HMM method), 110
set_fit_request() (aide_predict.bespoke_models.predictors.msa_transf method), 118
set_fit_request() (aide_predict.bespoke_models.predictors.pretrained_ method), 124
set_fit_request() (aide_predict.bespoke_models.predictors.saprot.SaP method), 131
set_fit_request() (aide_predict.bespoke_models.predictors.ssemb.SSE method), 136
set_fit_request() (aide_predict.bespoke_models.predictors.vespa.VES method), 142
set_output() (aide_predict.bespoke_models.base.ProteinModelWrapper method), 150
set_output() (aide_predict.bespoke_models.embedders.esm2.ESM2Embe method), 61
set_output() (aide_predict.bespoke_models.embedders.kmer.KmerEmbe method), 65
set_output() (aide_predict.bespoke_models.embedders.msa_transformer method), 71
set_output() (aide_predict.bespoke_models.embedders.ohe.OneHotAlign method), 76
set_output() (aide_predict.bespoke_models.embedders.ohe.OneHotProte method), 81
set_output() (aide_predict.bespoke_models.embedders.saprot.SaProtEm method), 86
set_output() (aide_predict.bespoke_models.predictors.esm2.ESM2Likeli method), 92
set_output() (aide_predict.bespoke_models.predictors.eve.EVEWrapper method), 99
set_output() (aide_predict.bespoke_models.predictors.evmutation.EVMu method), 105
set_output() (aide_predict.bespoke_models.predictors.hmm.HMMWrapp method), 111
set_output() (aide_predict.bespoke_models.predictors.msa_transformer. method), 118

set_output() (*aide_predict.bespoke_models.predictors.pretrained_transformers.LikelihoodTransformerBase method*), 125
set_output() (*aide_predict.bespoke_models.predictors.saprot.SaProtLikelihoodWrapper method*), 131
set_output() (*aide_predict.bespoke_models.predictors.ssemb.SSEmbWrapper method*), 137
set_output() (*aide_predict.bespoke_models.predictors.vespa.VESPAWrapper method*), 142
set_params() (*aide_predict.bespoke_models.base.ProteinModelWrapper method*), 151
set_params() (*aide_predict.bespoke_models.embedders.esm2.ESM2Embedding method*), 61
set_params() (*aide_predict.bespoke_models.embedders.kmer.KmerEmbedding method*), 66
set_params() (*aide_predict.bespoke_models.embedders.msa_transformer.MSATransformerEmbedding method*), 71
set_params() (*aide_predict.bespoke_models.embedders.ohe.OneHotAlignedEmbedding method*), 76
set_params() (*aide_predict.bespoke_models.embedders.ohe.OneHotProteinEmbedding method*), 81
set_params() (*aide_predict.bespoke_models.embedders.saprot.SaProtEmbedding method*), 86
set_params() (*aide_predict.bespoke_models.predictors.esm2.ESM2LikelihoodWrapper method*), 92
set_params() (*aide_predict.bespoke_models.predictors.eve.EVEWrapper method*), 99
set_params() (*aide_predict.bespoke_models.predictors.evmutation.EVMutationWrapper method*), 105
set_params() (*aide_predict.bespoke_models.predictors.hmm.HMMWrapper method*), 111
set_params() (*aide_predict.bespoke_models.predictors.msa_transformer.MSATransformerLikelihoodWrapper method*), 119
set_params() (*aide_predict.bespoke_models.predictors.pretrained_transformers.LikelihoodTransformerBase method*), 125
set_params() (*aide_predict.bespoke_models.predictors.saprot.SaProtLikelihoodWrapper method*), 132
set_params() (*aide_predict.bespoke_models.predictors.ssemb.SSEmbWrapper method*), 137
set_params() (*aide_predict.bespoke_models.predictors.vespa.VESPAWrapper method*), 143
set_score_request() (*aide_predict.bespoke_models.predictors.esm2.ESM2LikelihoodWrapper method*), 92
set_score_request() (*aide_predict.bespoke_models.predictors.eve.EVEWrapper method*), 100
set_score_request() (*aide_predict.bespoke_models.predictors.evmutation.EVMutationWrapper method*), 105
set_score_request() (*aide_predict.bespoke_models.predictors.hmm.HMMWrapper method*), 112
set_score_request() (*aide_predict.bespoke_models.predictors.msa_transformer.MSATransformerLikelihoodWrapper*
method), 119
set_score_request() (*aide_predict.bespoke_models.predictors.pretrained_transformers. method*), 126
set_score_request() (*aide_predict.bespoke_models.predictors.saprot.SaProtLikelihood method*), 132
set_score_request() (*aide_predict.bespoke_models.predictors.ssemb.SSEmbWrapper method*), 138
set_score_request() (*aide_predict.bespoke_models.predictors.vespa.VESPAWrapper method*), 143
should_refit_on_sequences (*aide_predict.bespoke_models.base.ProteinModelWrapper property*), 151
should_refit_on_sequences (*aide_predict.bespoke_models.embedders.esm2.ESM2Embedding property*), 61
should_refit_on_sequences (*aide_predict.bespoke_models.embedders.kmer.KmerEmbedding property*), 66
should_refit_on_sequences (*aide_predict.bespoke_models.embedders.msa_transformer.MSAT property*), 72
should_refit_on_sequences (*aide_predict.bespoke_models.embedders.ohe.OneHotAlignedEm property*), 76
should_refit_on_sequences (*aide_predict.bespoke_models.embedders.ohe.OneHotProteinEm property*), 81
should_refit_on_sequences (*aide_predict.bespoke_models.embedders.saprot.SaProtEmbeddin property*), 86
should_refit_on_sequences (*aide_predict.bespoke_models.predictors.esm2.ESM2LikelihoodW property*), 93
should_refit_on_sequences (*aide_predict.bespoke_models.predictors.eve.EVEWrapper property*), 100
should_refit_on_sequences (*aide_predict.bespoke_models.predictors.evmutation.EVMutation property*), 106
should_refit_on_sequences (*aide_predict.bespoke_models.predictors.hmm.HMMWrapper property*), 112
should_refit_on_sequences (*aide_predict.bespoke_models.predictors.msa_transformer.MSAT property*), 119
should_refit_on_sequences (*aide_predict.bespoke_models.predictors.pretrained_transformer. property*), 126
should_refit_on_sequences (*aide_predict.bespoke_models.predictors.saprot.SaProtLikelihood*

property), 132
should_refit_on_sequences (*aide_predict.bespoke_models.predictors.ssemb.SSEmbWrapper property*), 138
should_refit_on_sequences (*aide_predict.bespoke_models.predictors.vespa.VESPAWrapper property*), 143
ShouldRefitOnSequencesMixin (*class in aide_predict.bespoke_models.base*), 152
simple_simulated_annealing (*aide_predict.utils.badass.BADASSOptimizerParams attribute*), 186
sites_to_ignore (*aide_predict.utils.badass.BADASSOptimizerParams attribute*), 186
slice_as_protein_sequence() (*aide_predict.utils.data_structures.sequences.ProteinSequence method*), 165
sort() (*aide_predict.utils.data_structures.sequences.ProteinSequences method*), 172
sort() (*aide_predict.utils.data_structures.sequences.ProteinSequencesOnFile method*), 178
split() (*aide_predict.utils.data_structures.sequences.ProteinCharacter method*), 157
split() (*aide_predict.utils.data_structures.sequences.ProteinSequence method*), 165
splitlines() (*aide_predict.utils.data_structures.sequences.ProteinCharacter method*), 158
splitlines() (*aide_predict.utils.data_structures.sequences.ProteinSequence method*), 165
SSEmbWrapper (*class in aide_predict.bespoke_models.predictors.ssemb*), 133
startswith() (*aide_predict.utils.data_structures.sequences.ProteinCharacter method*), 158
startswith() (*aide_predict.utils.data_structures.sequences.ProteinSequence method*), 165
strip() (*aide_predict.utils.data_structures.sequences.ProteinCharacter method*), 158
strip() (*aide_predict.utils.data_structures.sequences.ProteinSequence method*), 165
structure (*aide_predict.utils.data_structures.sequences.ProteinSequence property*), 166
StructureMapper (*class in aide_predict.utils.data_structures.structures*), 181
sw_global_pairwise() (*in module aide_predict.utils.alignment_calls*), 183
swapcase() (*aide_predict.utils.data_structures.sequences.ProteinCharacter method*), 158
swapcase() (*aide_predict.utils.data_structures.sequences.ProteinSequence method*), 166

T

temperature (*aide_predict.utils.badass.BADASSOptimizerParams attribute*), 186
title() (*aide_predict.utils.data_structures.sequences.ProteinCharacter method*), 158
title() (*aide_predict.utils.data_structures.sequences.ProteinSequence method*), 166
to_dict() (*aide_predict.utils.badass.BADASSOptimizerParams method*), 186
to_dict() (*aide_predict.utils.data_structures.sequences.ProteinSequences method*), 166, 172
to_dict() (*aide_predict.utils.data_structures.sequences.ProteinSequences method*), 173, 178
to_fasta() (*aide_predict.utils.data_structures.sequences.ProteinSequence method*), 166, 172
to_fasta() (*aide_predict.utils.data_structures.sequences.ProteinSequence method*), 173, 179
to_memory() (*aide_predict.utils.data_structures.sequences.ProteinSequen method*), 179
to_on_file() (*aide_predict.utils.data_structures.sequences.ProteinSeque method*), 172
to_on_file() (*aide_predict.utils.data_structures.sequences.ProteinSeque method*), 179
transform() (*aide_predict.bespoke_models.base.ProteinModelWrapper method*), 151
transform() (*aide_predict.bespoke_models.embedders.esm2.ESM2Embed method*), 62
transform() (*aide_predict.bespoke_models.embedders.kmer.KmerEmbed method*), 66
transform() (*aide_predict.bespoke_models.embedders.msa_transformer. method*), 72
transform() (*aide_predict.bespoke_models.embedders.ohe.OneHotAligne method*), 77
transform() (*aide_predict.bespoke_models.embedders.ohe.OneHotProtei method*), 81
transform() (*aide_predict.bespoke_models.embedders.saprot.SaProtEmb method*), 86
transform() (*aide_predict.bespoke_models.predictors.esm2.ESM2Likelih method*), 93
transform() (*aide_predict.bespoke_models.predictors.eve.EVEWrapper method*), 100
transform() (*aide_predict.bespoke_models.predictors.evmutation.EVMut method*), 106
transform() (*aide_predict.bespoke_models.predictors.hmm.HMMWrappe method*), 112
transform() (*aide_predict.bespoke_models.predictors.msa_transformer.M method*), 119
transform() (*aide_predict.bespoke_models.predictors.pretrained_transfo method*), 126
transform() (*aide_predict.bespoke_models.predictors.saprot.SaProtLikel method*), 133
transform() (*aide_predict.bespoke_models.predictors.ssemb.SSEmbWrap method*), 138
transform() (*aide_predict.bespoke_models.predictors.vespa.VESPAWrapp method*), 143
translate() (*aide_predict.utils.data_structures.sequences.ProteinCharac*

method), 158
translate() (*aide_predict.utils.data_structures.sequences.ProteinSequence method*), 166

U

upper() (*aide_predict.utils.data_structures.sequences.ProteinCharacter method*), 158
upper() (*aide_predict.utils.data_structures.sequences.ProteinSequence method*), 166
upper() (*aide_predict.utils.data_structures.sequences.ProteinSequences method*), 172
upper() (*aide_predict.utils.data_structures.sequences.ProteinSequencesOnFile method*), 179

V

validate_sequence() (*aide_predict.utils.data_structures.structures.ProteinStructure method*), 181
VESPAWrapper (*class in aide_predict.bespoke_models.predictors.vespa*), 139

W

weights (*aide_predict.utils.data_structures.sequences.ProteinSequences property*), 172
weights (*aide_predict.utils.data_structures.sequences.ProteinSequencesOnFile property*), 179
width (*aide_predict.utils.data_structures.sequences.ProteinSequences property*), 172
width (*aide_predict.utils.data_structures.sequences.ProteinSequencesOnFile property*), 179
WILDTYPE (*aide_predict.bespoke_models.predictors.pretrained_transformers.MarginalMethod attribute*), 127
with_no_gaps() (*aide_predict.utils.data_structures.sequences.ProteinSequence method*), 166
with_no_gaps() (*aide_predict.utils.data_structures.sequences.ProteinSequences method*), 172
with_no_gaps() (*aide_predict.utils.data_structures.sequences.ProteinSequencesOnFile method*), 179
wrap() (*in module aide_predict.utils.common*), 187
write_fasta() (*in module aide_predict.io.bio_files*), 153
wt (*aide_predict.bespoke_models.base.ProteinModelWrapper property*), 151
wt (*aide_predict.bespoke_models.embedders.esm2.ESM2Embedding property*), 62
wt (*aide_predict.bespoke_models.embedders.kmer.KmerEmbedding property*), 66
wt (*aide_predict.bespoke_models.embedders.msa_transformer.MSATransformerEmbedding property*), 72
wt (*aide_predict.bespoke_models.embedders.ohe.OneHotAlignedEmbedding property*), 77
wt (*aide_predict.bespoke_models.embedders.ohe.OneHotProteinEmbedding property*), 81
wt (*aide_predict.bespoke_models.embedders.saprot.SaProtEmbedding property*), 86
wt (*aide_predict.bespoke_models.predictors.esm2.ESM2LikelihoodWrapper property*), 93
wt (*aide_predict.bespoke_models.predictors.eve.EVEWrapper property*), 101
wt (*aide_predict.bespoke_models.predictors.evmutation.EVMutationWrapp property*), 106
wt (*aide_predict.bespoke_models.predictors.hmm.HMMWrapper property*), 112
wt (*aide_predict.bespoke_models.predictors.msa_transformer.MSATransfor property*), 120
wt (*aide_predict.bespoke_models.predictors.pretrained_transformers.Likeli property*), 127
wt (*aide_predict.bespoke_models.predictors.saprot.SaProtLikelihoodWrapp property*), 133
wt (*aide_predict.bespoke_models.predictors.ssemb.SSEmbWrapper property*), 138
wt (*aide_predict.bespoke_models.predictors.vespa.VESPAWrapper property*), 144

Z

zfill() (*aide_predict.utils.data_structures.sequences.ProteinCharacter method*), 158
zfill() (*aide_predict.utils.data_structures.sequences.ProteinSequence method*), 166
