# Supplementary material for: Accessible, uniform protein property prediction with a scikit-learn based toolset AIDE
Source: Bioinformatics. 2025 Sep 24;41(10):btaf544. doi: 10.1093/bioinformatics/btaf544 (PMC12553329; doi:10.1093/bioinformatics/btaf544)
Supplement: btaf544_Supplementary_Data [file btaf544_supplementary_data.zip › AppendixB.pdf]

wt_petase_model_creation

May 14, 2025

1 Creating a competative supervised model for wild type PETase activity at low pH

We show in a seperate paper that supervised models should be used for predicting PETase activity at unique conditions (eg. low pH) once some assay labeled data is available, and this outperforms HMMs. Here we create models that: 1. Take in embeddings as input, explore over: Aligned OHE, ES|M2, SaProt, MSATransformer 2. Use linear vs non-linear models: Linear regression, Random Forest

Download the data: >Norton-Baker, B., Komp, E., Gado, J., Denton, M. C. R., Mathews, I. I., Murphy, N. P., Erickson, E., Storment, O. O., Sarangi, R., Gauthier, N. P., McGeehan, J., & Beckham, G. (2025). Activity across temperature and pH of PET hydrolase candidates. [Data set]. Zenodo. https://doi.org/10.5281/zenodo.15417757

Hyperperameter optimization is conducted over the models for each input type.

Save the final model, which can be loaded like any other sklearn model if AIDE is installed.

eg. `model=joblib.load('model.pkl')`

[2]:
```
import os

import pandas as pd
import numpy as np
from sklearn.model_selection import RandomizedSearchCV, KFold, cross_validate
from sklearn.feature_selection import VarianceThreshold
from sklearn.linear_model import ElasticNet, LinearRegression, Ridge
from sklearn.neural_network import MLPRegressor
from sklearn.preprocessing import StandardScaler
from sklearn.decomposition import PCA
from sklearn.pipeline import Pipeline
from sklearn.metrics import roc_auc_score
from sklearn.ensemble import RandomForestRegressor
from scipy.stats import loguniform, spearmanr
import seaborn as sns
import matplotlib.pyplot as plt
sns.set_style("white")
sns.set_context("talk")
```

```
import aide_predict as ap
from aide_predict.utils.data_structures.structures import StructureMapper
```

```
/kfs2/projects/proteinml/repos/aide_predict/aide_predict/patches_.py:7:
FutureWarning: In the future `np.str` will be defined as the corresponding NumPy
scalar.
  if not hasattr(np, 'str'):
/projects/proteinml/.links/miniconda3/envs/aidep/lib/python3.10/site-
packages/Bio/pairwise2.py:278: BiopythonDeprecationWarning: Bio.pairwise2 has
been deprecated, and we intend to remove it in a future release of Biopython. As
an alternative, please consider using Bio.Align.PairwiseAligner as a
replacement, and contact the Biopython developers if you still need the
Bio.pairwise2 module.
  warnings.warn(
```

1.1 1. Load and prepare data

We need to get: 1. The sequences and labels 2. Assign their structures (for SaProt Embedding) 3. Get and MSA of known PETases (for baseline HMMscore and MSA transformer)

[3]:
```
RAW_DATA_DIR = os.path.join('.', 'data', 'p740')
```

1.1.1 1.1 Label data

[4]:
```
df = pd.read_csv(os.path.join(RAW_DATA_DIR, 'label_data.csv')).sample(frac=1,␣
↪random_state=42)
```

[5]:
```
df
```

[5]:
```
     Unnamed: 0                                           sequence  round  \
9         DP021  ADNPYQRGPAPTAASISADTGPFATATTVAEGTGFGGATIYYPTDT…      1
197      ESM041  AAAAGRADQRGPDPSVAGVAATYGPFATAQLTVPAGNGFNGGYIYY…      3
66       TEP081  MHPTPDRAKVLPVNVSRGPAEPPAARSARPGGRSAPDGLRPGRRRP…      2
191      ESM053  VQIGPAPTKASLEASRGPFTVATTRLSANGHGGGTIYYPTNAGAKV…      3
117      TEP182  MAENPYERGPAPTTSSIEASRGSFATSTVTVSRLAVSGFGGGTIYY…      2
..          …                                                …    …
106      TEP014  ANPYERGPNPTQALLEARSGPFSVSSERAWRLGSDGFGGGTIYYPR…      2
14        DP009  SAQVTRQAAGSYARGPAPTLAGIRAALGPFAYSTVTVTAAQAGGAF…      1
92       TEP024  RPASAQDNPYERGPAPTVSSVAAQRGTFATAELTVPPGNGFNGGKI…      2
179      ESM011  DSPYQRGPDPTLASVAATRGPFATTQATVPAGNGFNGGFVYYPTDT…      3
102      TEP188  ADNPYERGPAPTNASIEAVRGPYAVSQATVSSLAVTGFGGGTIYYP…      2

     temporal_split  cross_val_split  has_nonzero_activity_anywhere  \
9                 0                3                          False
197               4                2                          False
66                1                1                          False
191               4                2                           True
```

```
117              2               0                            True
..             …              …                            …
106              2               4                            True
14               0               2                            True
92               2               1                            True
179              4               1                           False
102              2               0                           False

     max_observed_activity  activity_at_5.5_40_cryPow  \
9                    0.000                       0.00
197                  0.000                       0.00
66                   0.000                       0.00
191                  0.030                       0.01
117                  0.020                       0.01
..                    …                        …
106                  0.005                       0.00
14                   0.010                       0.00
92                   0.010                       0.00
179                  0.000                       0.00
102                  0.000                       0.00

     activity_at_5.5_60_cryPow  activity_at_6.5_40_aFilm  \
9                          0.0                       NaN
197                        NaN                       NaN
66                         NaN                       NaN
191                        0.0                       NaN
117                        0.0                       0.0
..                        …                        …
106                        0.0                       0.0
14                         0.0                       NaN
92                         0.0                       0.0
179                        0.0                       0.0
102                        0.0                       NaN

     activity_at_6.5_40_cryPow  activity_at_6.5_60_cryPow  \
9                         0.00                       0.00
197                       0.00                        NaN
66                        0.00                        NaN
191                       0.01                       0.01
117                       0.01                       0.01
..                        …                         …
106                       0.00                       0.00
14                        0.00                       0.00
92                        0.01                       0.00
179                       0.00                       0.00
102                       0.00                       0.00
```

```
     activity_at_7.5_40_aFilm  activity_at_7.5_40_cryPow  \
9                         NaN                      0.000
197                       NaN                      0.000
66                        NaN                      0.000
191                       NaN                      0.030
117                       0.0                      0.020
..                        …                        …
106                       0.0                      0.005
14                        NaN                      0.010
92                        0.0                      0.010
179                       0.0                      0.000
102                       NaN                      0.000

     activity_at_7.5_60_aFilm  activity_at_7.5_60_cryPow  \
9                         NaN                       0.00
197                       NaN                        NaN
66                        NaN                        NaN
191                       NaN                       0.01
117                       0.0                       0.02
..                        …                         …
106                       0.0                       0.00
14                        NaN                       0.00
92                        0.0                       0.00
179                       0.0                       0.00
102                       NaN                       0.00

     activity_at_8.5_40_cryPow  activity_at_4.5_40_cryPow
9                         0.00                        NaN
197                        NaN                        0.0
66                         NaN                        0.0
191                        NaN                        0.0
117                        NaN                        0.0
..                         …                          …
106                        NaN                        0.0
14                        0.01                        NaN
92                         NaN                        0.0
179                        NaN                        0.0
102                        NaN                        0.0

[213 rows x 18 columns]
```

[6]:

```
df = df.dropna(subset=['activity_at_5.5_40_cryPow'])
# drop rows with non canonical AAs - these were not predicted properly by AF
X = ap.ProteinSequences.from_df(df,seq_col='sequence', id_col='Unnamed: 0')
has_non_canonical = [x.has_non_canonical for x in X]
df = df[~np.array(has_non_canonical)]
```

[7]:
```
X, y = ap.ProteinSequences.from_df(df, seq_col='sequence', id_col='Unnamed: 0',␣
↪label_cols=['activity_at_5.5_40_cryPow'])
```

[8]:
```
sns.kdeplot(y, bw_adjust=0.5)
plt.xlabel('Activity at 5.5 pH, 40°C')
```

[8]:
```
Text(0.5, 0, 'Activity at 5.5 pH, 40°C')
```

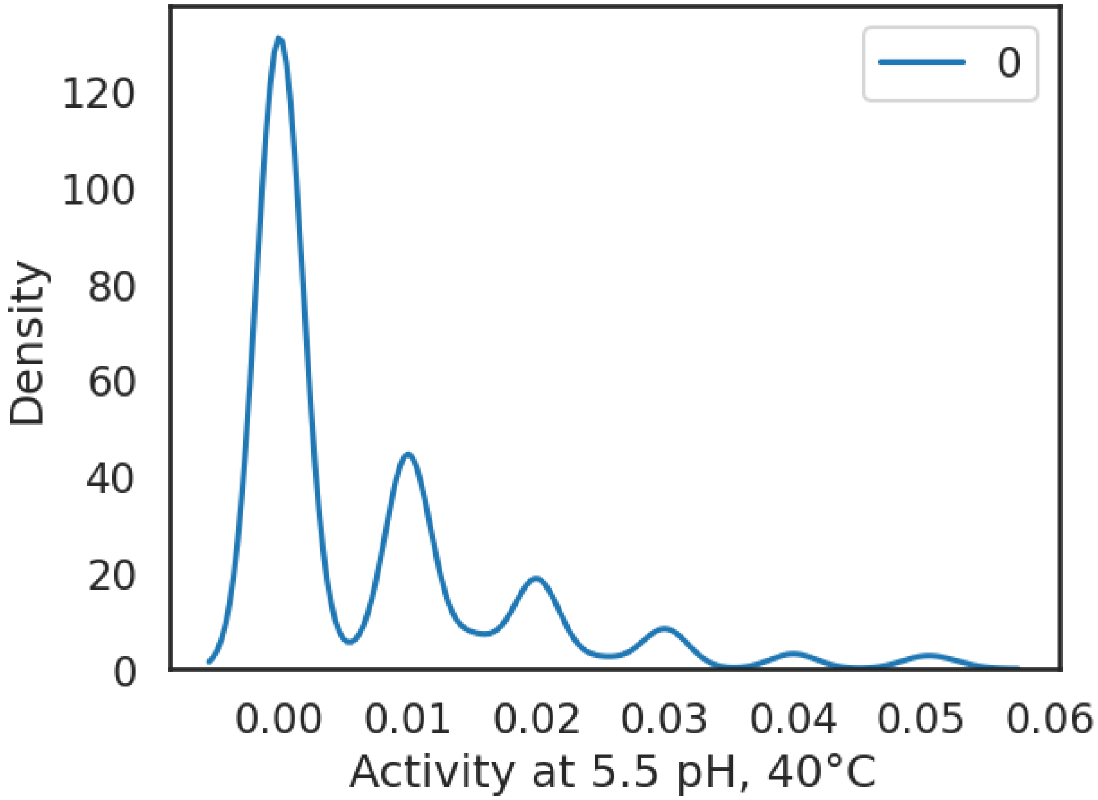

1.1.2 1.2 Structures

[9]:
```
# get the structures - Needed for SaProt embedding
mapper = StructureMapper(os.path.join(RAW_DATA_DIR, 'structures'))
mapper.assign_structures(X)
```

[9]:
```
ProteinSequences(count=212)
```

1.1.3 1.3 Homolog MSA (for MSA transformer)

Compute weights so that MSA transformer can sample it properly.

[10]:
```
msa = ap.ProteinSequences.from_fasta(os.path.join(RAW_DATA_DIR, 'hmm-61.mfa'))
msa.aligned
```

[10]: True

[11]:
```
msa.width
```

[11]: 898

[12]:
```
# also assign the msa to the sequences so that msa transformer can access it
for seq in X:
    seq.msa = msa
```

1.2 2. Define scoring functions

[13]:
```
# 5 fold cv
cv_obj = KFold(n_splits=5, shuffle=True, random_state=42)
```

[14]:
```
# metrics to measure
# marks magnitude of error eg R2, also score AUROC to see if the model can␣
↪classify active or not
scoring = {
    'spearman': lambda est, X, y: spearmanr(y, est.predict(X))[0],
    'roc_auc': lambda est, X, y: roc_auc_score(y > 0.001, est.predict(X))
}
```

[15]:
```
def construct_pipeline(embedder, model, pca: bool=True):
    if not pca:
        return Pipeline([
            ('embedder', embedder),
            ('var', VarianceThreshold()),
            ('scaler', StandardScaler()),
            ('model', model)
        ])
    else:
        return Pipeline([
            ('embedder', embedder),
            ('var', VarianceThreshold()),
            ('scaler', StandardScaler()),
            ('pca', PCA(n_components=0.98)),
            ('model', model)
        ])

def evaluate_pipeline_with_hyperopt(embedder_name, embedder, model_info,␣
↪n_iter=10):
```

```
    """Run hyperparameter optimization on a pipeline with a given embedder and␣
↪model

    Params:
    embedder: Embedder object eg ap.BaseProteinModel
    model_info: dict with keys:
        'model': sklearn model object
        'param_distributions': dict of hyperparameter distributions for␣
↪RandomizedSearchCV
    """
    do_pca = 'OneHot' not in embedder_name
    pipeline = construct_pipeline(embedder, model_info['model'], pca=do_pca)
    random_search = RandomizedSearchCV(
        pipeline,
        param_distributions=model_info['param_dist'],
        n_iter=n_iter,
        cv=cv_obj,
        scoring=scoring,
        refit='spearman',
        verbose=2,
        n_jobs=1)
    random_search.fit(X, y)

    best_params = random_search.best_params_
    cv_scores = cross_validate(pipeline.set_params(**best_params), X, y,␣
↪cv=cv_obj, scoring=scoring)
    return best_params, cv_scores
```

1.3 3. Baseline model: HMM

[16]:
```
hmm = ap.HMMWrapper()
hmm.fit(msa)

baseline_scores = {
    k: v(hmm, X, y) for k, v in scoring.items()
}
print('Baseline scores:', baseline_scores)
```

```
# hmmbuild :: profile HMM construction from multiple sequence alignments
# HMMER 3.4 (Aug 2023); http://hmmer.org/
# Copyright (C) 2023 Howard Hughes Medical Institute.
# Freely distributed under the BSD open source license.
# - - - - - - - - - - - - - - - - - - - - - - - - - - - - - - - - - - - -
# input alignment file:
/tmp/HMMWrapper_20250506_092305/alignment.a2m
# output HMM file:
/tmp/HMMWrapper_20250506_092305/alignment.hmm
```

```
# - - - - - - - - - - - - - - - - - - - - - - - - - - - - - - - - - - - -

# idx name                  nseq  alen  mlen eff_nseq re/pos description
#---- -------------------- ----- ----- ----- --------- ------ -----------
1     alignment               61   898   332      4.32  0.590

# CPU time: 0.14u 0.00s 00:00:00.14 Elapsed: 00:00:00.14
```

```
/kfs2/projects/proteinml/repos/aide_predict/aide_predict/bespoke_models/predicto
rs/hmm.py:176: FutureWarning: The 'delim_whitespace' keyword in pd.read_csv is
deprecated and will be removed in a future version. Use ``sep='\s+'`` instead
  data = pd.read_csv(out_tbl, delim_whitespace=True, comment='#', header=None)
```

```
Baseline scores: {'spearman': -0.06975930257389627, 'roc_auc':
0.47665585919407133}
```

```
/kfs2/projects/proteinml/repos/aide_predict/aide_predict/bespoke_models/predicto
rs/hmm.py:176: FutureWarning: The 'delim_whitespace' keyword in pd.read_csv is
deprecated and will be removed in a future version. Use ``sep='\s+'`` instead
  data = pd.read_csv(out_tbl, delim_whitespace=True, comment='#', header=None)
```

1.4 4. Supervised learning: Define embedders, models, and hyperparameter space

[17]:

```python
embedders = {
    'ESM2': ap.ESM2Embedding(
        metadata_folder='esm2_embeddings',␣
↪model_checkpoint='esm2_t33_650M_UR50D', device='cuda:1', pool='mean'),
    'SaProt': ap.SaProtEmbedding(metadata_folder='saprot_embeddings',␣
↪device='cuda:1', pool='mean'),
    'MSATransformer': ap.MSATransformerEmbedding(
        metadata_folder='msa_embeddings', device='cuda:1', pool=False,␣
↪flatten=True, # chosen because there will be a lot of gaps, so mean pool␣
↪will get saturated by gaps
        n_msa_seqs=31, batch_size=32
    ),
    'AlignedOneHot': ap.OneHotAlignedEmbedding(
        metadata_folder='onehot_embeddings')
}
# fit the models that have fixed fitting over folds
embedders['ESM2'].fit()
embedders['SaProt'].fit()
embedders['MSATransformer'].fit()
```

```
Some weights of EsmModel were not initialized from the model checkpoint at
facebook/esm2_t33_650M_UR50D and are newly initialized: ['pooler.dense.bias',
'pooler.dense.weight']
```

```
You should probably TRAIN this model on a down-stream task to be able to use it
for predictions and inference.
Some weights of EsmModel were not initialized from the model checkpoint at
westlake-repl/SaProt_650M_AF2 and are newly initialized:
['contact_head.regression.bias', 'contact_head.regression.weight',
'embeddings.position_embeddings.weight', 'pooler.dense.bias',
'pooler.dense.weight']
You should probably TRAIN this model on a down-stream task to be able to use it
for predictions and inference.
```

[17]:
```
MSATransformerEmbedding(device='cuda:1', flatten=True,
                        metadata_folder='msa_embeddings', n_msa_seqs=31)
```

[20]:
```python
models = {
    'Ridge': {
        'model': Ridge(),
        'param_dist': {
            'model__alpha': loguniform(1e-5, 1e2),
        }
    },
    'RandomForest': {
        'model': RandomForestRegressor(n_estimators=10),
        'param_dist': {
            'model__max_depth': [None, 10, 100],
            'model__min_samples_split': [2, 5, 10],
            'model__min_samples_leaf': [1, 5, 10]
        }
    }
}
```

1.5 5. Train and evaluate models with hyperparameter optimization

[21]:
```python
import joblib
if not os.path.exists('search_results.pkl'):
    results = {}
else:
    results = joblib.load('search_results.pkl')
```

[22]:
```python
results
```

[22]:
```
{'ESM2_Ridge': {'embedder': 'ESM2',
  'model': 'Ridge',
  'best_params': {'model__alpha': 67.83794155101033},
  'spearman': array([0.73325634, 0.51952198, 0.5185911 , 0.41303091,
0.29284354]),
  'roc_auc': array([0.8959276 , 0.76973684, 0.76417234, 0.73333333,
```

```
0.67108753])},
 'ESM2_RandomForest': {'embedder': 'ESM2',
  'model': 'RandomForest',
  'best_params': {'model__min_samples_split': 10,
   'model__min_samples_leaf': 5,
   'model__max_depth': 10},
  'spearman': array([0.71394883, 0.52054878, 0.23287475, 0.39442078,
0.29086657]),
  'roc_auc': array([0.89819005, 0.75438596, 0.58956916, 0.72345679,
0.69230769])},
 'SaProt_Ridge': {'embedder': 'SaProt',
  'model': 'Ridge',
  'best_params': {'model__alpha': 86.68878466127286},
  'spearman': array([0.71892587, 0.49434321, 0.54815407, 0.44436428,
0.38740863]),
  'roc_auc': array([0.89140271, 0.76535088, 0.79365079, 0.74814815,
0.75066313])},
 'SaProt_RandomForest': {'embedder': 'SaProt',
  'model': 'RandomForest',
  'best_params': {'model__min_samples_split': 10,
   'model__min_samples_leaf': 10,
   'model__max_depth': 10},
  'spearman': array([0.68908961, 0.64365332, 0.48380593, 0.50731589,
0.41422383]),
  'roc_auc': array([0.8800905 , 0.85526316, 0.75510204, 0.77037037,
0.77320955])},
 'MSATransformer_Ridge': {'embedder': 'MSATransformer',
  'model': 'Ridge',
  'best_params': {'model__alpha': 1.0774036315824223e-05},
  'spearman': array([ 0.13592487,  0.21124989,  0.10028396, -0.08393548,
-0.01968863]),
  'roc_auc': array([0.5678733 , 0.65131579, 0.60544218, 0.43209877, 0.4668435
])},
 'MSATransformer_RandomForest': {'embedder': 'MSATransformer',
  'model': 'RandomForest',
  'best_params': {'model__min_samples_split': 2,
   'model__min_samples_leaf': 10,
   'model__max_depth': 100},
  'spearman': array([-0.03629287,  0.01247378, -0.1253086 ,  0.00811826,
-0.07255363]),
  'roc_auc': array([0.51809955, 0.50877193, 0.4399093 , 0.50493827,
0.40981432])},
 'AlignedOneHot_Ridge': {'embedder': 'AlignedOneHot',
  'model': 'Ridge',
  'best_params': {'model__alpha': 0.001558737330417496},
  'spearman': array([0.76998351, 0.71717534, 0.39874602, 0.51453206,
0.47153279]),
```

```
  'roc_auc': array([0.92986425, 0.90789474, 0.70975057, 0.78765432,
0.78779841])},
 'AlignedOneHot_RandomForest': {'embedder': 'AlignedOneHot',
  'model': 'RandomForest',
  'best_params': {'model__min_samples_split': 2,
   'model__min_samples_leaf': 5,
   'model__max_depth': 10},
  'spearman': array([0.68039666, 0.52833455, 0.3305744 , 0.40241285,
0.43515628]),
  'roc_auc': array([0.86877828, 0.80701754, 0.64172336, 0.71604938,
0.76127321])}}
```

[23]:

```
for embedder_name, embedder in embedders.items():
    for model_name, model_info in models.items():
        if f'{embedder_name}_{model_name}' in results:
            print(f"Skipping {embedder_name} with {model_name}...")
            continue
        else:
            print(f"Evaluating {embedder_name} with {model_name}...")
        best_params, scores = evaluate_pipeline_with_hyperopt(embedder_name,␣
↪embedder, model_info, n_iter=50)
        results[f'{embedder_name}_{model_name}'] = {
            'embedder': embedder_name,
            'model': model_name,
            'best_params': best_params,
            'spearman': scores['test_spearman'],
            'roc_auc': scores['test_roc_auc']
        }
        joblib.dump(results, 'search_results.pkl')
```

```
Skipping ESM2 with Ridge…
Skipping ESM2 with RandomForest…
Skipping SaProt with Ridge…
Skipping SaProt with RandomForest…
Skipping MSATransformer with Ridge…
Skipping MSATransformer with RandomForest…
Skipping AlignedOneHot with Ridge…
Skipping AlignedOneHot with RandomForest…
```

[24]:

```
# convert to long
df_list = []
for item in results.values():
    for i in range(5):  # Assuming 5 values for each metric
        df_list.append({
            'embedder': item['embedder'],
            'model': item['model'],
            'spearman': item['spearman'][i],
```

```
            'roc_auc': item['roc_auc'][i]
        })

df = pd.DataFrame(df_list)

# Melt the DataFrame to create a column for the metric type
df_melted = pd.melt(df, id_vars=['embedder', 'model'], var_name='metric',␣
 ↪value_name='value')
```

[25]:
```
df_melted.to_csv('fig3_data.csv', index=False)
```

[30]:
```
plt.figure(figsize=(8, 10))

# Create the faceted plot
g = sns.catplot(
    data=df_melted,
    kind="bar",
    x="model",
    y="value",
    hue="metric",
    col="embedder",
    height=4,
    aspect=1.0,
    palette="Set2",
    col_wrap=4,
    ci="sd",
    legend=True,  # We'll add the legend manually
    # change color
    edgecolor='black',
    linewidth=2

)
# remove legend from seaborn
g._legend.remove()
# add baselines of the same color as the bars
for ax in g.axes.flat:
    for i, metric in enumerate(['spearman', 'roc_auc']):
        ax.axhline(baseline_scores[metric], color=sns.color_palette("Set2")[i],␣
 ↪linestyle='--', label=f'HMM (baseline) {metric}')
    ax.set_ylim(-.1, 1)

plt.legend(loc='upper center', bbox_to_anchor=(-1.1, -0.18), ncol=4)
# Customize the plot
g.set_axis_labels("")
g.set_titles("{col_name}")

# lower the
```

```python
# Display the plot
plt.savefig('p740_model_comparison.png', bbox_inches='tight', dpi=300)
```

```
/tmp/ipykernel_2947768/1272998501.py:4: FutureWarning:

The `ci` parameter is deprecated. Use `errorbar='sd'` for the same effect.

  g = sns.catplot(
```

```
<Figure size 800x1000 with 0 Axes>
```

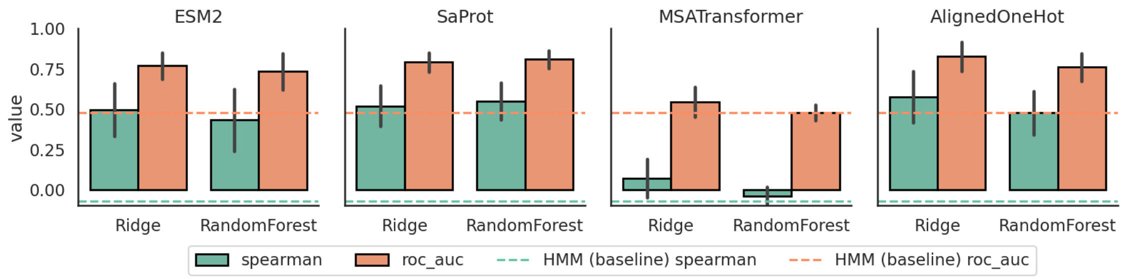

1.6 6. Train final model and save

[31]:
```python
best_row = df.groupby(['embedder', 'model']).mean().sort_values('roc_auc',␣
↪ascending=False).iloc[0]
best_row
```

[31]:
```
spearman    0.574394
roc_auc     0.824592
Name: (AlignedOneHot, Ridge), dtype: float64
```

[36]:
```python
best_pipeline = construct_pipeline(embedders[best_row.name[0]], models[best_row.
↪name[1]]['model'], pca=False)
```

[37]:
```python
best_params = results[f'{best_row.name[0]}_{best_row.name[1]}']['best_params']
```

[38]:
```python
models[best_row.name[1]]['model']
```

[38]:
```
Ridge(alpha=0.001558737330417496)
```

[39]:
```python
best_pipeline.set_params(**best_params)
```

[39]:
```
Pipeline(steps=[('embedder',
                 OneHotAlignedEmbedding(metadata_folder='onehot_embeddings')),
                ('var', VarianceThreshold()), ('scaler', StandardScaler()),
                ('model', Ridge(alpha=0.001558737330417496))])
```

1.7 First do a CV prediction so we can plot parity

[40]:
```
y_trues = []
y_preds = []
for train_idx, test_idx in cv_obj.split(X):
    best_pipeline.fit(X[train_idx], y[train_idx])
    y_pred = best_pipeline.predict(X[test_idx])
    y_trues.append(y[test_idx])
    y_preds.append(y_pred)

y_trues = np.concatenate(y_trues)
y_preds = np.concatenate(y_preds)
y_trues = y_trues > 0.001
```

```
/kfs2/projects/proteinml/repos/aide_predict/aide_predict/bespoke_models/embedder
s/ohe.py:278: UserWarning: Input sequences are not aligned. Aligning them to the
original alignment.
  warnings.warn("Input sequences are not aligned. Aligning them to the original
alignment.")
/kfs2/projects/proteinml/repos/aide_predict/aide_predict/bespoke_models/embedder
s/ohe.py:278: UserWarning: Input sequences are not aligned. Aligning them to the
original alignment.
  warnings.warn("Input sequences are not aligned. Aligning them to the original
alignment.")
/kfs2/projects/proteinml/repos/aide_predict/aide_predict/bespoke_models/embedder
s/ohe.py:278: UserWarning: Input sequences are not aligned. Aligning them to the
original alignment.
  warnings.warn("Input sequences are not aligned. Aligning them to the original
alignment.")
/kfs2/projects/proteinml/repos/aide_predict/aide_predict/bespoke_models/embedder
s/ohe.py:278: UserWarning: Input sequences are not aligned. Aligning them to the
original alignment.
  warnings.warn("Input sequences are not aligned. Aligning them to the original
alignment.")
/kfs2/projects/proteinml/repos/aide_predict/aide_predict/bespoke_models/embedder
s/ohe.py:278: UserWarning: Input sequences are not aligned. Aligning them to the
original alignment.
  warnings.warn("Input sequences are not aligned. Aligning them to the original
alignment.")
/kfs2/projects/proteinml/repos/aide_predict/aide_predict/bespoke_models/embedder
s/ohe.py:278: UserWarning: Input sequences are not aligned. Aligning them to the
original alignment.
  warnings.warn("Input sequences are not aligned. Aligning them to the original
alignment.")
/kfs2/projects/proteinml/repos/aide_predict/aide_predict/bespoke_models/embedder
s/ohe.py:278: UserWarning: Input sequences are not aligned. Aligning them to the
original alignment.
  warnings.warn("Input sequences are not aligned. Aligning them to the original
```

```
alignment.")
/kfs2/projects/proteinml/repos/aide_predict/aide_predict/bespoke_models/embedder
s/ohe.py:278: UserWarning: Input sequences are not aligned. Aligning them to the
original alignment.
  warnings.warn("Input sequences are not aligned. Aligning them to the original
alignment.")
/kfs2/projects/proteinml/repos/aide_predict/aide_predict/bespoke_models/embedder
s/ohe.py:278: UserWarning: Input sequences are not aligned. Aligning them to the
original alignment.
  warnings.warn("Input sequences are not aligned. Aligning them to the original
alignment.")
/kfs2/projects/proteinml/repos/aide_predict/aide_predict/bespoke_models/embedder
s/ohe.py:278: UserWarning: Input sequences are not aligned. Aligning them to the
original alignment.
  warnings.warn("Input sequences are not aligned. Aligning them to the original
alignment.")
```

[41]:

```
def Find_Optimal_Cutoff(target, predicted):
    """ Find the optimal probability cutoff point for a classification model␣
 ↪related to event rate
    Parameters
    ----------
    target : Matrix with dependent or target data, where rows are observations

    predicted : Matrix with predicted data, where rows are observations

    Returns
    -------
    list type, with optimal cutoff value

    """
    from sklearn.metrics import roc_curve
    fpr, tpr, threshold = roc_curve(target, predicted)
    i = np.arange(len(tpr))
    roc = pd.DataFrame({'tf' : pd.Series(tpr-(1-fpr), index=i), 'threshold' :␣
 ↪pd.Series(threshold, index=i)})
    roc_t = roc.iloc[(roc.tf-0).abs().argsort()[:1]]

    return list(roc_t['threshold'])

cutoff = Find_Optimal_Cutoff(y_trues, y_preds)
cutoff
```

[41]: [0.005645442926271432]

[42]:

```
precision = np.sum((y_preds > cutoff) & y_trues) / np.sum(y_preds > cutoff)
precision
```

[42]: 0.6391752577319587

[45]:
```
fig, ax = plt.subplots(figsize=(8, 2))

df_ = pd.DataFrame({
    'y_true': y_trues.flatten(),
    'y_pred': y_preds.flatten(),
})

sns.boxplot(data=df_, x='y_pred', y='y_true', ax=ax, orient='h')
ax.set_xlabel('Model score')
ax.set_ylabel('Experimentally active \nat pH=5.5, 40°C')
ax.set_title('Precision (expected hit rate): {:.2f}'.format(precision))

ax.fill_between([ax.get_xlim()[0], cutoff[0]], [ax.get_ylim()[0], ax.
 ↪get_ylim()[0]], [ax.get_ylim()[1], ax.get_ylim()[1]], color='grey', alpha=0.
 ↪5)
ax.vlines(cutoff[0], ax.get_ylim()[0], ax.get_ylim()[1], color='red',␣
 ↪linestyle='--', label='Decision boundary')
plt.legend(bbox_to_anchor=(.25, -.4,), loc='upper left')
plt.savefig('p740_precision.png', bbox_inches='tight', dpi=300)
```

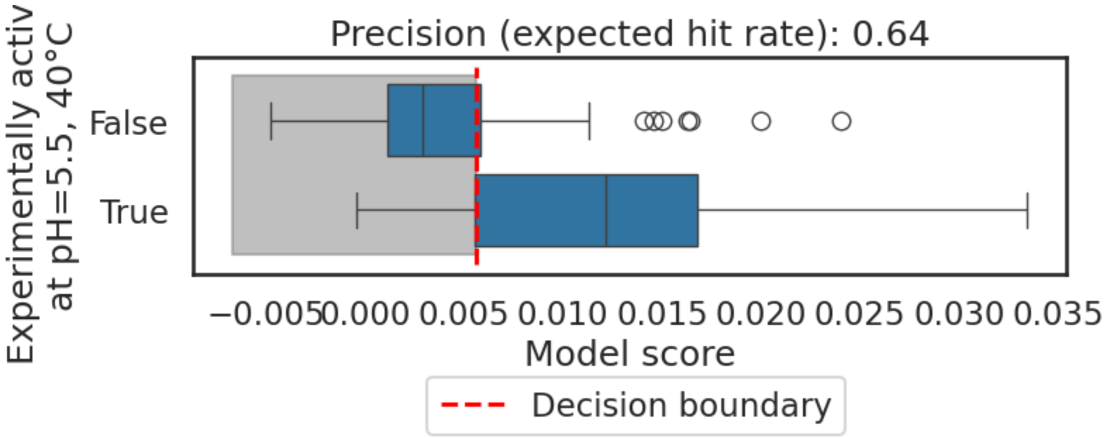

[46]:
```
best_pipeline = best_pipeline.fit(X, y)
```

```
/kfs2/projects/proteinml/repos/aide_predict/aide_predict/bespoke_models/embedder
s/ohe.py:278: UserWarning: Input sequences are not aligned. Aligning them to the
original alignment.
  warnings.warn("Input sequences are not aligned. Aligning them to the original
alignment.")
```

[47]:
```
preds = best_pipeline.predict(X)
```

```
/kfs2/projects/proteinml/repos/aide_predict/aide_predict/bespoke_models/embedder
s/ohe.py:278: UserWarning: Input sequences are not aligned. Aligning them to the
original alignment.
  warnings.warn("Input sequences are not aligned. Aligning them to the original
alignment.")
```

[48]:
```
joblib.dump(best_pipeline, 'p740_best_pipeline.pkl')
```

[48]:
```
['p740_best_pipeline.pkl']
```

1.8 7. Load model and predict

[49]:
```
import joblib
best_pipeline = joblib.load('p740_best_pipeline.pkl')
```

[50]:
```
preds = best_pipeline.predict(X)
```

```
/kfs2/projects/proteinml/repos/aide_predict/aide_predict/bespoke_models/embedder
s/ohe.py:278: UserWarning: Input sequences are not aligned. Aligning them to the
original alignment.
  warnings.warn("Input sequences are not aligned. Aligning them to the original
alignment.")
```

[51]:
```
preds
```

[51]:
```
array([[ 4.09470608e-10],
       [ 2.62595394e-09],
       [-1.49346806e-10],
       [ 9.99999950e-03],
       [ 1.00000015e-02],
       [ 3.05852435e-10],
       [ 4.82003894e-09],
       [ 3.98192960e-10],
       [ 8.21041975e-09],
       [ 6.91821198e-10],
       [ 9.99999856e-03],
       [ 3.99999322e-02],
       [ 2.14445496e-09],
       [ 2.64940886e-10],
       [-5.17693135e-10],
       [ 2.99999893e-02],
       [ 2.99999703e-02],
       [ 1.63759887e-09],
       [ 3.36233957e-09],
       [ 1.99999930e-02],
       [ 1.02378027e-08],
       [-4.85636236e-10],
```

```
[ 2.28727708e-09],
[ 1.00000000e-02],
[ 1.99999995e-02],
[ 1.99999967e-02],
[ 1.22329702e-09],
[ 9.99998230e-03],
[ 1.81703685e-09],
[ 4.79542311e-10],
[ 2.99999674e-02],
[ 4.99999815e-02],
[ 2.93682240e-09],
[ 9.43059512e-10],
[ 1.81236652e-10],
[ 3.40490398e-09],
[-1.66555745e-09],
[ 9.99999614e-03],
[ 9.99999811e-03],
[ 9.99999452e-03],
[ 1.99999937e-02],
[ 3.99999899e-02],
[-8.41008469e-11],
[ 1.99999952e-02],
[ 7.51803440e-09],
[ 1.99999993e-02],
[ 4.99999910e-03],
[ 1.99999954e-02],
[ 4.23487816e-10],
[ 6.53770181e-10],
[ 2.90781677e-09],
[ 5.96691266e-10],
[ 1.08329407e-09],
[ 1.99999946e-02],
[ 1.90299555e-09],
[ 1.99999986e-02],
[ 4.84301615e-10],
[ 2.00000030e-02],
[ 4.82629546e-09],
[ 4.48992308e-09],
[ 9.99999994e-03],
[ 9.99999343e-03],
[ 9.99999742e-03],
[ 9.99999987e-03],
[ 1.74454894e-08],
[ 1.00000049e-02],
[ 8.23468618e-10],
[ 3.74034980e-10],
[ 1.81816497e-09],
```

```
[ 2.04303712e-09],
[ 9.99999785e-03],
[ 9.99998983e-03],
[ 1.56742151e-09],
[ 9.99999570e-03],
[ 3.10711320e-10],
[ 7.10378426e-09],
[ 2.78351574e-09],
[ 5.14522925e-09],
[ 9.83719645e-10],
[ 3.11738056e-10],
[ 6.64873797e-10],
[ 1.37286022e-09],
[ 3.78983846e-09],
[ 9.99999585e-03],
[ 5.24034515e-09],
[ 1.71144497e-09],
[ 9.99999841e-03],
[ 1.99999834e-02],
[ 5.88160239e-09],
[ 1.84952039e-10],
[ 9.99999969e-03],
[ 2.99999945e-02],
[ 9.99999831e-03],
[ 9.26675082e-11],
[ 5.87372872e-09],
[ 1.12435145e-08],
[ 5.36214040e-10],
[ 1.87812789e-09],
[ 1.67306797e-09],
[ 1.04455376e-09],
[ 1.38335092e-09],
[ 9.99998835e-03],
[-2.70749365e-10],
[ 2.90283882e-09],
[ 3.24197599e-09],
[ 9.99999437e-03],
[ 1.88300813e-09],
[ 9.99999646e-03],
[ 1.99999961e-02],
[ 1.49155519e-09],
[ 2.36352568e-09],
[ 1.16145253e-09],
[ 3.55474928e-09],
[ 9.98354955e-10],
[ 1.66175792e-09],
[ 1.99999995e-02],
```

```
[ 2.61023906e-09],
[ 1.00000058e-02],
[ 1.56580710e-09],
[ 7.68034560e-10],
[ 1.00000008e-02],
[ 3.88377377e-09],
[ 1.50000142e-02],
[ 1.33091808e-10],
[ 5.76884456e-09],
[ 1.00000026e-02],
[ 9.69297915e-11],
[ 2.99999717e-02],
[ 3.89117379e-09],
[ 2.89131351e-09],
[ 1.00000263e-02],
[ 3.37972027e-09],
[ 3.99999714e-02],
[ 2.50280752e-09],
[ 1.04850780e-08],
[ 7.51749635e-11],
[ 3.21758458e-09],
[ 5.19999843e-02],
[ 2.00000164e-02],
[ 3.19438413e-09],
[ 1.49999974e-02],
[ 9.99999675e-03],
[ 9.99999703e-03],
[ 1.99999971e-02],
[ 9.99999835e-03],
[ 5.00000568e-03],
[ 2.48338943e-09],
[ 1.99999915e-02],
[ 4.99999863e-02],
[ 2.49999910e-02],
[ 1.58545946e-09],
[ 1.49999967e-02],
[ 2.99999932e-02],
[ 5.68301739e-09],
[ 8.80656181e-09],
[ 8.50935275e-10],
[ 5.39803827e-10],
[ 1.08686036e-09],
[ 1.00000045e-02],
[ 9.99999787e-03],
[ 9.35810171e-09],
[ 1.00000159e-02],
[ 7.39228063e-10],
```

```
[-2.38702096e-10],
[ 2.00000117e-02],
[ 2.60109920e-10],
[ 1.52954845e-09],
[ 1.99999961e-02],
[ 1.68812737e-09],
[ 1.37579154e-09],
[ 1.00000162e-02],
[ 1.50000188e-02],
[ 1.30482774e-09],
[ 4.08411322e-09],
[ 8.65600377e-09],
[ 9.99999592e-03],
[ 1.00000001e-02],
[ 1.49999947e-02],
[ 1.96691585e-09],
[ 1.50000117e-02],
[ 2.44886844e-10],
[ 1.30798086e-09],
[ 2.49999974e-02],
[ 1.07223158e-09],
[ 1.00000053e-02],
[ 1.00000095e-02],
[ 2.16768613e-09],
[ 1.81267683e-09],
[ 1.00000188e-02],
[ 2.01293365e-09],
[ 1.04728018e-09],
[ 9.99999461e-03],
[ 4.88358373e-10],
[ 5.54614634e-09],
[ 2.99999822e-02],
[ 1.76539674e-10],
[ 1.26165288e-09],
[ 1.00000070e-02],
[ 2.96559986e-09],
[ 1.00000070e-02],
[ 3.78598194e-09],
[ 1.04319380e-09],
[ 9.99999594e-03],
[ 5.68815614e-10],
[ 9.66619623e-11],
[ 2.99999743e-02],
[ 5.58217745e-10],
[ 2.08647065e-09],
[ 3.40143808e-09],
[ 3.34551228e-09],
```

```
         [ 1.95501513e-09],
         [ 6.85415975e-10]])
```

```
[ ]:
```
