# Supplementary material for: Accessible, uniform protein property prediction with a scikit-learn based toolset AIDE
Source: Bioinformatics. 2025 Sep 24;41(10):btaf544. doi: 10.1093/bioinformatics/btaf544 (PMC12553329; doi:10.1093/bioinformatics/btaf544)
Supplement: btaf544_Supplementary_Data [file btaf544_supplementary_data.zip › AppendixA.pdf]

epistatic_benchmarking

May 14, 2025

1 Benchmarking unsupervised and supervised models on the 4 site combinatorial library of TrpB

1. Johnston, K. E. et al. A combinatorially complete epistatic fitness landscape in an enzyme active site. Proceedings of the National Academy of Sciences 121, e2400439121 (2024).

The work in this notebook will look fundamentally similar to a subset of the work done by *Hsu et al.*, but in a single, readable, notebook. Additionally, we consider more than just OHE for AA embeddings.

We will probe slightly different factors than the original work as an "update" for those conclusions, on a epistatic benchmark. The goal is to do so in a way that highlights the integrated nature of AIDE and showcase how many different model types/methods can be accessed ina single, readable, notebook with the API in place.

Differences in this work: - Compare zero shot methods not in original paper to EV mutation: ESM2 and MSA transformer wild type marginal - Compare different embedding methods as oposed to just one hot encoding: ESM2 mean pooling over whole sequence, ESM2 mean pooling over only the 4 variable residues - Compare linear to a nonlinear top model - Conduct 5 fold CV not just for hyperparameter optimization, but also test set performance. Repeat with 20 random instantiations like the original paper.

Naming conventions:

<zs_model>_<embedding>_<top_model>

[1]:
```
import os
import json

import pandas as pd
import numpy as np
import joblib

import matplotlib.pyplot as plt
import seaborn as sns
sns.set(style="white")
sns.set_context("talk")

from sklearn.pipeline import Pipeline
from sklearn.compose import TransformedTargetRegressor
```

```python
from sklearn.neural_network import MLPRegressor
from sklearn.ensemble import RandomForestRegressor
from sklearn.model_selection import KFold, RandomizedSearchCV, cross_validate
from sklearn.metrics import make_scorer
from sklearn.preprocessing import StandardScaler
from sklearn.decomposition import PCA
from sklearn.linear_model import Ridge

from scipy.stats import kendalltau, spearmanr
from scipy.stats import loguniform, uniform

import aide_predict as ap
from aide_predict.utils.msa import MSAProcessing

import logging
logging.basicConfig(level=logging.INFO)
```

```
/kfs2/projects/proteinml/repos/aide_predict/aide_predict/patches_.py:7:
FutureWarning: In the future `np.str` will be defined as the corresponding NumPy
scalar.
  if not hasattr(np, 'str'):
/projects/proteinml/.links/miniconda3/envs/aidep/lib/python3.10/site-
packages/Bio/pairwise2.py:278: BiopythonDeprecationWarning: Bio.pairwise2 has
been deprecated, and we intend to remove it in a future release of Biopython. As
an alternative, please consider using Bio.Align.PairwiseAligner as a
replacement, and contact the Biopython developers if you still need the
Bio.pairwise2 module.
  warnings.warn(
```

1.1 TOC

0. Acquiring the data
1. Data preprocessing
2. Self supervised predictors
3. Supervised learning
4. Combined models
5. Compare results

1.2 0. Acquiring the data

1. Download and extract the data from here. You want `data.zip`
2. Extract the data to a directory of your choice
3. Assign the global variable `RAW_DATA_DIR` to the extracted directory

[2]:
```python
RAW_DATA_DIR = os.path.join('.', 'data', 'epistatic', 'data')
```

1.2.1 0.1. Retrieve the assay labeled data

[3]:
```
df = pd.read_csv(os.path.join(RAW_DATA_DIR, 'figure_data',␣
↪'4-site_merged_replicates', '20230827', 'four-site_summary_AA_data.csv'))
```

[4]:
```
df = df[['AAs', 'avg_mu-bg/max']].rename(columns={'AAs': 'sequence', 'avg_mu-bg/
↪max': 'fitness'})
```

[5]:
```
# remove star aas
df = df[~df['sequence'].apply(lambda x: '*' in x)]
```

1.2.2 0.2. Define wildtype sequence, get full sequences of variants

The wildtype in the study is not acutally the wildtype protein, they started with a variant. Define the true wt, apply mutations.

[ ]:
```
wt = ap.ProteinSequence(
    ␣
↪'MKGYFGPYGGQYVPEILMGALEELEAAYEGIMKDESFWKEFNDLLRDYAGRPTPLYFARRLSEKYGARVYLKREDLLHTGAHKINNAIGQ
    id='wt',
)
```

[ ]:
```
mutations = ['P18G', 'E29G', 'I68V', 'K95L', 'P139L', 'N166D', 'I183F',␣
↪'L212P', 'G227S', 'T291S']
wt = wt.mutate(mutations, one_indexed=False)
wt.id='Tm8D9'
```

[ ]:
```
msa = ap.ProteinSequences.from_fasta(os.path.join(RAW_DATA_DIR, 'EVmutation',␣
↪'TARGET_b0.1', 'align', 'TARGET_b0.1.a2m'))
```

[ ]:
```
wt.id = msa[0].id
```

[ ]:
```
msa_prc = MSAProcessing()
msa_sub = msa_prc.process(msa, focus_seq_id=wt.id)
```

```
INFO:aide_predict.utils.msa:Starting MSA processing with 138861 sequences
INFO:aide_predict.utils.msa:Preprocessing MSA
INFO:aide_predict.utils.msa:Preprocessed MSA: 137434 sequences, 397 columns
INFO:aide_predict.utils.msa:Computing sequence weights
INFO:aide_predict.bespoke_models.base:Created metadata folder:
/tmp/OneHotAlignedEmbedding_20250428_142459
INFO:aide_predict.bespoke_models.base:Fitting OneHotAlignedEmbedding
INFO:aide_predict.bespoke_models.base:Transforming input using
OneHotAlignedEmbedding
/kfs2/projects/proteinml/repos/aide_predict/aide_predict/bespoke_models/embedder
s/ohe.py:275: UserWarning: Input sequences are already aligned. Using them as-is
for encoding.
  warnings.warn("Input sequences are already aligned. Using them as-is for
```

```
encoding.")
INFO:aide_predict.utils.msa:Computed weights: Neff (effective sequence count) =
57762.59
INFO:aide_predict.utils.msa:MSA processing completed
```

[20]:
```
# sample 1k highly weighted sequences for computation cost reasons
msa_sub = msa_sub.sample(n=1000, keep_first=True)
```

[21]:
```
msa_sub.to_fasta('selected_msa.fasta')
```

[6]:
```
wt = ap.ProteinSequence.from_fasta('selected_msa.fasta')
```

The authors only provide the identity of the 4 variable positions - convert them to full protein sequences to be compatible with aide.

[7]:
```
library_positions = [182, 183, 226, 227]
mutation_strings_base = [
    f"{wt[pos]}{pos}" for pos in library_positions
]
def get_full_sequence(wt, aas):
    mutation_strings = [mutation_strings_base[i]+aa for i, aa in enumerate(aas)]
    return wt.mutate(mutation_strings, one_indexed=False)
df['full_sequence'] = df['sequence'].apply(lambda x: get_full_sequence(wt, x))
```

Shuffle the data and create X, y

[8]:
```
df = df.sample(frac=1, random_state=0)
X = ap.ProteinSequences(df['full_sequence'].tolist())
y = df['fitness'].values
```

Plot the activity data

[9]:
```
wt_activity = df[df['sequence'] == 'VFVS'].iloc[0]['fitness']
wt_activity
```

[9]:
```
0.4080739252340478
```

[10]:
```
fig, ax = plt.subplots(figsize=(6, 6))
sns.kdeplot(y, ax=ax, bw_adjust=0.5)
ax.vlines(wt_activity, 0, ax.get_ylim()[1], color='red', label='WT activity')
ax.legend()
ax.set_xlabel('Fitness')
```

[10]:
```
Text(0.5, 0, 'Fitness')
```

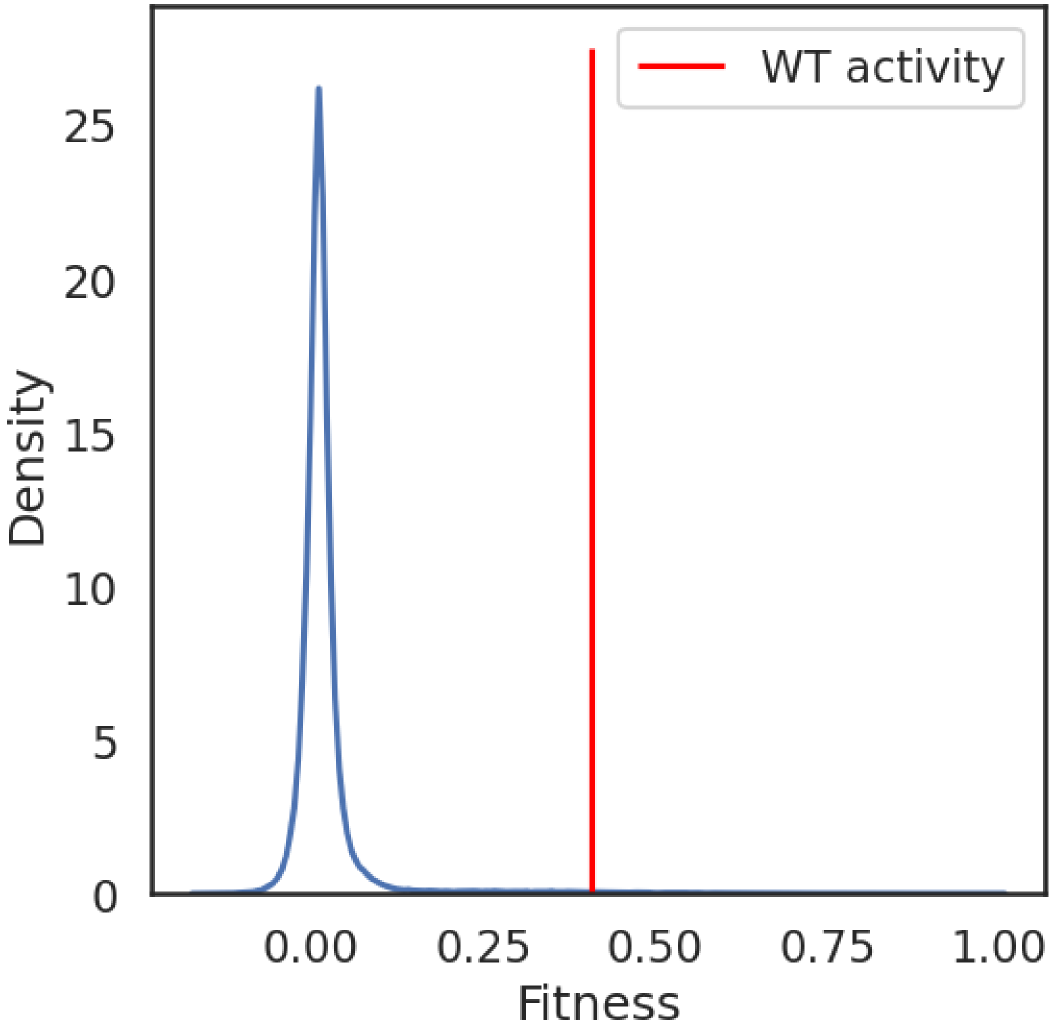

1.3 1. Data preprocessing

We need to: 1. Define splits for Kfold the better resemble actual training set sizes, eg. train on 130k variants, test on remaining 20k is not reasonable. Note that plenty of work on active learning suggest we can get accuracy with fewer samples by non random selection, but acquisition functions/active learning are currently native to AIDE. It is not hard to use AIDE models in an active learning loop, but we do not have boilerplate importable code for that yet so here we are doing more standard supervised model evaluation. 2. Define metrics that are relevant to the study, kendall's tau and top 10 recovery is a better representation of our experimental goal than spearman. 3. Define a wrapper function to run hyperparameter optimization and test set as training size increases, over random instatiation

1.3.1 1.1. Define splits

[11]:
```
def modified_kfold_iterator(X, n_splits=5, shuffle=True, random_state=42,␣
↪training_size=96*100, test_size='all'):
    """KFold iterator that uses smaller training set and larger test set

    Params:
    -------
    X: array-like
        The data to split
    n_splits: int
        Number of splits
    shuffle: bool
        Whether to shuffle the data
    random_state: int
        Random state
    training_size: int
        Size of the training set, will be sampled from fold
    test_size: int or 'all'
        Size of the test set, if 'all' then all data not in training set will␣
↪be used
    """
    kf = KFold(n_splits=n_splits, shuffle=shuffle, random_state=random_state)
    np.random.seed(random_state)
    for test_index, train_index in kf.split(X):
        # sample to training size
        train_index = np.random.choice(train_index, training_size,␣
↪replace=False)
        if test_size != 'all':
            test_index = np.random.choice(test_index, test_size, replace=False)
        yield train_index, test_index
```

1.3.2 1.2. Define metrics

[12]:
```
def top10_in_plate(y_true, y_pred):
    """Calculate top 10 recovery in a single plate, eg. how many of the top 10␣
↪true values are in the top 96 predicted values"""
    # now get top 10 recovery in a single plate
    y_true = np.array(y_true)
    y_pred = np.array(y_pred)

    top_10_idx = list(np.argsort(y_true)[-10:])
    top_100_predicted_idx = list(np.argsort(y_pred)[-96:])
    top_10_recovery = sum([1 for idx in top_10_idx if idx in␣
↪top_100_predicted_idx]) / 10
    return top_10_recovery
```

[13]:
```python
scoring = {
    'kendall_tau': make_scorer(lambda y_true, y_pred: kendalltau(y_true,␣
↪y_pred)[0]),
    'top10_in_plate': make_scorer(top10_in_plate),
    'spearman': make_scorer(lambda y_true, y_pred: spearmanr(y_true,␣
↪y_pred)[0]),
}
```

[14]:
```python
def cv_1(model, X, y, training_size=96*10, random_state=42):
    """Cross validate a model with a single training size.
    """
    return cross_validate(
        model,
        X,
        y,
        cv=modified_kfold_iterator(X, training_size=training_size,␣
↪random_state=random_state),
        scoring=scoring,
    )

def cv_increasing_data_size(model, X, y, training_sizes=[24*2, 24*3, 24*4,␣
↪24*5, 24*6, 24*7, 24*8, 24*9, 24*10, 24*12, 24*16, 24*20, 24*24, 24*100],␣
↪n_repeats=20):
    """Cross validate a model with increasing training sizes, over a number of␣
↪repeats."""
    results = []
    for repeat in range(n_repeats):
        for training_size in training_sizes:
            results.append(cv_1(model, X, y, training_size=training_size,␣
↪random_state=repeat))
            results[-1]['training_size'] = training_size
    return results

def hyperopt_and_scoring(model, X, y, param_distributions,␣
↪ho_training_size=96*10, eval_training_sizes=[24*2, 24*3, 24*4, 24*5, 24*6,␣
↪24*7, 24*8, 24*9, 24*10, 24*12, 24*16, 24*20, 24*24, 24*100], n_repeats=20):
    """Hyperparameter optimization and scoring over increasing training sizes␣
↪in one call.

    Params
    ------
    model: sklearn estimator
        The model to optimize and score
    X: array-like
    y: array-like
    param_distributions: dict
```

```
        The hyperparameter distributions to search over, to be fed to␣
↪RandomizedSearchCV
    ho_training_size: int
        The size of the training set for hyperparameter optimization
    eval_training_sizes: list of ints
        The training sizes to evaluate the model on
    n_repeats: int
        Number of repeats to average over
    """
    searcher = RandomizedSearchCV(
        model,
        param_distributions=param_distributions,
        n_iter=100,
        n_jobs=1,
        cv=modified_kfold_iterator(X, training_size=ho_training_size),
        scoring=scoring,
        refit='kendall_tau',
    )
    searcher.fit(X, y)
    best_params = searcher.best_params_
    scores = cv_increasing_data_size(model.set_params(**best_params), X, y,␣
↪training_sizes=eval_training_sizes, n_repeats=n_repeats)
    return scores
```

1.4 2. Self supervised predictors

1.4.1 2.1 ESM2 with wildtype marginal

[15]:
```
esm2liklihood = ap.ESM2LikelihoodWrapper(
    wt=wt,
    marginal_method='wildtype_marginal',
    device='cuda:1',
    metadata_folder=os.path.join('.', 'data', 'epistatic', 'esm2likelihood'),
    model_checkpoint='esm2_t33_650M_UR50D',
    use_cache=True,
)
esm2liklihood.fit()
```

[15]:
```
ESM2LikelihoodWrapper(device='cuda:1', marginal_method='wildtype_marginal',
                      metadata_folder='./data/epistatic/esm2likelihood',
                      model_checkpoint='esm2_t33_650M_UR50D',
                      wt=ProteinSequence(id='TARGET/1-397',
seq='mkgyfgpyggqyvpeilmga…'))
```

[34]:
```
esm_none_none_scores = cv_1(esm2liklihood, X, y)
```

```
/kfs2/projects/proteinml/repos/aide_predict/aide_predict/bespoke_models/base.py:
388: UserWarning: This model expects no fit, but received input. Ignoring input:
[ProteinSequence(id=None, seq='mkgyfgpyggqyvpeilmga…'),
ProteinSequence(id=None, seq='mkgyfgpyggqyvpeilmga…'),
ProteinSequence(id=None, seq='mkgyfgpyggqyvpeilmga…'),
ProteinSequence(id=None, seq='mkgyfgpyggqyvpeilmga…'),
ProteinSequence(id=None, seq='mkgyfgpyggqyvpeilmga…'),
ProteinSequence(id=None, seq='mkgyfgpyggqyvpeilmga…'),
ProteinSequence(id=None, seq='mkgyfgpyggqyvpeilmga…'),
ProteinSequence(id=None, seq='mkgyfgpyggqyvpeilmga…'),
ProteinSequence(id=None, seq='mkgyfgpyggqyvpeilmga…'),
ProteinSequence(id=None, seq='mkgyfgpyggqyvpeilmga…'),
ProteinSequence(id=None, seq='mkgyfgpyggqyvpeilmga…'),
ProteinSequence(id=None, seq='mkgyfgpyggqyvpeilmga…'),
ProteinSequence(id=None, seq='mkgyfgpyggqyvpeilmga…'),
ProteinSequence(id=None, seq='mkgyfgpyggqyvpeilmga…'),
ProteinSequence(id=None, seq='mkgyfgpyggqyvpeilmga…'),
ProteinSequence(id=None, seq='mkgyfgpyggqyvpeilmga…'),
ProteinSequence(id=None, seq='mkgyfgpyggqyvpeilmga…'),
ProteinSequence(id=None, seq='mkgyfgpyggqyvpeilmga…'),
ProteinSequence(id=None, seq='mkgyfgpyggqyvpeilmga…'),
ProteinSequence(id=None, seq='mkgyfgpyggqyvpeilmga…'),
ProteinSequence(id=None, seq='mkgyfgpyggqyvpeilmga…'),
ProteinSequence(id=None, seq='mkgyfgpyggqyvpeilmga…'),
ProteinSequence(id=None, seq='mkgyfgpyggqyvpeilmga…'),
ProteinSequence(id=None, seq='mkgyfgpyggqyvpeilmga…'),
ProteinSequence(id=None, seq='mkgyfgpyggqyvpeilmga…'),
ProteinSequence(id=None, seq='mkgyfgpyggqyvpeilmga…'),
ProteinSequence(id=None, seq='mkgyfgpyggqyvpeilmga…'),
ProteinSequence(id=None, seq='mkgyfgpyggqyvpeilmga…'),
ProteinSequence(id=None, seq='mkgyfgpyggqyvpeilmga…'),
ProteinSequence(id=None, seq='mkgyfgpyggqyvpeilmga…'),
ProteinSequence(id=None, seq='mkgyfgpyggqyvpeilmga…'),
ProteinSequence(id=None, seq='mkgyfgpyggqyvpeilmga…'),
ProteinSequence(id=None, seq='mkgyfgpyggqyvpeilmga…'),
ProteinSequence(id=None, seq='mkgyfgpyggqyvpeilmga…'),
ProteinSequence(id=None, seq='mkgyfgpyggqyvpeilmga…'),
ProteinSequence(id=None, seq='mkgyfgpyggqyvpeilmga…'),
ProteinSequence(id=None, seq='mkgyfgpyggqyvpeilmga…'),
ProteinSequence(id=None, seq='mkgyfgpyggqyvpeilmga…'),
ProteinSequence(id=None, seq='mkgyfgpyggqyvpeilmga…'),
ProteinSequence(id=None, seq='mkgyfgpyggqyvpeilmga…'),
ProteinSequence(id=None, seq='mkgyfgpyggqyvpeilmga…'),
ProteinSequence(id=None, seq='mkgyfgpyggqyvpeilmga…'),
ProteinSequence(id=None, seq='mkgyfgpyggqyvpeilmga…'),
ProteinSequence(id=None, seq='mkgyfgpyggqyvpeilmga…'),
ProteinSequence(id=None, seq='mkgyfgpyggqyvpeilmga…'),
ProteinSequence(id=None, seq='mkgyfgpyggqyvpeilmga…'),
```

```
ProteinSequence(id=None, seq='mkgyfgpyggqyvpeilmga…'),
ProteinSequence(id=None, seq='mkgyfgpyggqyvpeilmga…'),
ProteinSequence(id=None, seq='mkgyfgpyggqyvpeilmga…'),
ProteinSequence(id=None, seq='mkgyfgpyggqyvpeilmga…'),
ProteinSequence(id=None, seq='mkgyfgpyggqyvpeilmga…'),
ProteinSequence(id=None, seq='mkgyfgpyggqyvpeilmga…'),
ProteinSequence(id=None, seq='mkgyfgpyggqyvpeilmga…'),
ProteinSequence(id=None, seq='mkgyfgpyggqyvpeilmga…'),
ProteinSequence(id=None, seq='mkgyfgpyggqyvpeilmga…'),
ProteinSequence(id=None, seq='mkgyfgpyggqyvpeilmga…'),
ProteinSequence(id=None, seq='mkgyfgpyggqyvpeilmga…'),
ProteinSequence(id=None, seq='mkgyfgpyggqyvpeilmga…'),
ProteinSequence(id=None, seq='mkgyfgpyggqyvpeilmga…'),
ProteinSequence(id=None, seq='mkgyfgpyggqyvpeilmga…'),
ProteinSequence(id=None, seq='mkgyfgpyggqyvpeilmga…'),
ProteinSequence(id=None, seq='mkgyfgpyggqyvpeilmga…'),
ProteinSequence(id=None, seq='mkgyfgpyggqyvpeilmga…'),
ProteinSequence(id=None, seq='mkgyfgpyggqyvpeilmga…'),
ProteinSequence(id=None, seq='mkgyfgpyggqyvpeilmga…'),
ProteinSequence(id=None, seq='mkgyfgpyggqyvpeilmga…'),
ProteinSequence(id=None, seq='mkgyfgpyggqyvpeilmga…'),
ProteinSequence(id=None, seq='mkgyfgpyggqyvpeilmga…'),
ProteinSequence(id=None, seq='mkgyfgpyggqyvpeilmga…'),
ProteinSequence(id=None, seq='mkgyfgpyggqyvpeilmga…'),
ProteinSequence(id=None, seq='mkgyfgpyggqyvpeilmga…'),
ProteinSequence(id=None, seq='mkgyfgpyggqyvpeilmga…'),
ProteinSequence(id=None, seq='mkgyfgpyggqyvpeilmga…'),
ProteinSequence(id=None, seq='mkgyfgpyggqyvpeilmga…'),
ProteinSequence(id=None, seq='mkgyfgpyggqyvpeilmga…'),
ProteinSequence(id=None, seq='mkgyfgpyggqyvpeilmga…'),
ProteinSequence(id=None, seq='mkgyfgpyggqyvpeilmga…'),
ProteinSequence(id=None, seq='mkgyfgpyggqyvpeilmga…'),
ProteinSequence(id=None, seq='mkgyfgpyggqyvpeilmga…'),
ProteinSequence(id=None, seq='mkgyfgpyggqyvpeilmga…'),
ProteinSequence(id=None, seq='mkgyfgpyggqyvpeilmga…'),
ProteinSequence(id=None, seq='mkgyfgpyggqyvpeilmga…'),
ProteinSequence(id=None, seq='mkgyfgpyggqyvpeilmga…'),
ProteinSequence(id=None, seq='mkgyfgpyggqyvpeilmga…'),
ProteinSequence(id=None, seq='mkgyfgpyggqyvpeilmga…'),
ProteinSequence(id=None, seq='mkgyfgpyggqyvpeilmga…'),
ProteinSequence(id=None, seq='mkgyfgpyggqyvpeilmga…'),
ProteinSequence(id=None, seq='mkgyfgpyggqyvpeilmga…'),
ProteinSequence(id=None, seq='mkgyfgpyggqyvpeilmga…'),
ProteinSequence(id=None, seq='mkgyfgpyggqyvpeilmga…'),
ProteinSequence(id=None, seq='mkgyfgpyggqyvpeilmga…'),
ProteinSequence(id=None, seq='mkgyfgpyggqyvpeilmga…'),
ProteinSequence(id=None, seq='mkgyfgpyggqyvpeilmga…'),
ProteinSequence(id=None, seq='mkgyfgpyggqyvpeilmga…'),
```

```
ProteinSequence(id=None, seq='mkgyfgpyggqyvpeilmga…'),
ProteinSequence(id=None, seq='mkgyfgpyggqyvpeilmga…'),
ProteinSequence(id=None, seq='mkgyfgpyggqyvpeilmga…'),
ProteinSequence(id=None, seq='mkgyfgpyggqyvpeilmga…'),
ProteinSequence(id=None, seq='mkgyfgpyggqyvpeilmga…'),
ProteinSequence(id=None, seq='mkgyfgpyggqyvpeilmga…'),
ProteinSequence(id=None, seq='mkgyfgpyggqyvpeilmga…'),
ProteinSequence(id=None, seq='mkgyfgpyggqyvpeilmga…'),
ProteinSequence(id=None, seq='mkgyfgpyggqyvpeilmga…'),
ProteinSequence(id=None, seq='mkgyfgpyggqyvpeilmga…'),
ProteinSequence(id=None, seq='mkgyfgpyggqyvpeilmga…'),
ProteinSequence(id=None, seq='mkgyfgpyggqyvpeilmga…'),
ProteinSequence(id=None, seq='mkgyfgpyggqyvpeilmga…'),
ProteinSequence(id=None, seq='mkgyfgpyggqyvpeilmga…'),
ProteinSequence(id=None, seq='mkgyfgpyggqyvpeilmga…'),
ProteinSequence(id=None, seq='mkgyfgpyggqyvpeilmga…'),
ProteinSequence(id=None, seq='mkgyfgpyggqyvpeilmga…'),
ProteinSequence(id=None, seq='mkgyfgpyggqyvpeilmga…'),
ProteinSequence(id=None, seq='mkgyfgpyggqyvpeilmga…'),
ProteinSequence(id=None, seq='mkgyfgpyggqyvpeilmga…'),
ProteinSequence(id=None, seq='mkgyfgpyggqyvpeilmga…'),
ProteinSequence(id=None, seq='mkgyfgpyggqyvpeilmga…'),
ProteinSequence(id=None, seq='mkgyfgpyggqyvpeilmga…'),
ProteinSequence(id=None, seq='mkgyfgpyggqyvpeilmga…'),
ProteinSequence(id=None, seq='mkgyfgpyggqyvpeilmga…'),
ProteinSequence(id=None, seq='mkgyfgpyggqyvpeilmga…'),
ProteinSequence(id=None, seq='mkgyfgpyggqyvpeilmga…'),
ProteinSequence(id=None, seq='mkgyfgpyggqyvpeilmga…'),
ProteinSequence(id=None, seq='mkgyfgpyggqyvpeilmga…'),
ProteinSequence(id=None, seq='mkgyfgpyggqyvpeilmga…'),
ProteinSequence(id=None, seq='mkgyfgpyggqyvpeilmga…'),
ProteinSequence(id=None, seq='mkgyfgpyggqyvpeilmga…'),
ProteinSequence(id=None, seq='mkgyfgpyggqyvpeilmga…'),
ProteinSequence(id=None, seq='mkgyfgpyggqyvpeilmga…'),
ProteinSequence(id=None, seq='mkgyfgpyggqyvpeilmga…'),
ProteinSequence(id=None, seq='mkgyfgpyggqyvpeilmga…'),
ProteinSequence(id=None, seq='mkgyfgpyggqyvpeilmga…'),
ProteinSequence(id=None, seq='mkgyfgpyggqyvpeilmga…'),
ProteinSequence(id=None, seq='mkgyfgpyggqyvpeilmga…'),
ProteinSequence(id=None, seq='mkgyfgpyggqyvpeilmga…'),
ProteinSequence(id=None, seq='mkgyfgpyggqyvpeilmga…'),
ProteinSequence(id=None, seq='mkgyfgpyggqyvpeilmga…'),
ProteinSequence(id=None, seq='mkgyfgpyggqyvpeilmga…'),
ProteinSequence(id=None, seq='mkgyfgpyggqyvpeilmga…'),
ProteinSequence(id=None, seq='mkgyfgpyggqyvpeilmga…'),
ProteinSequence(id=None, seq='mkgyfgpyggqyvpeilmga…'),
ProteinSequence(id=None, seq='mkgyfgpyggqyvpeilmga…'),
ProteinSequence(id=None, seq='mkgyfgpyggqyvpeilmga…'),
```

```
ProteinSequence(id=None, seq='mkgyfgpyggqyvpeilmga…'),
ProteinSequence(id=None, seq='mkgyfgpyggqyvpeilmga…'),
ProteinSequence(id=None, seq='mkgyfgpyggqyvpeilmga…'),
ProteinSequence(id=None, seq='mkgyfgpyggqyvpeilmga…'),
ProteinSequence(id=None, seq='mkgyfgpyggqyvpeilmga…'),
ProteinSequence(id=None, seq='mkgyfgpyggqyvpeilmga…'),
ProteinSequence(id=None, seq='mkgyfgpyggqyvpeilmga…'),
ProteinSequence(id=None, seq='mkgyfgpyggqyvpeilmga…'),
ProteinSequence(id=None, seq='mkgyfgpyggqyvpeilmga…'),
ProteinSequence(id=None, seq='mkgyfgpyggqyvpeilmga…'),
ProteinSequence(id=None, seq='mkgyfgpyggqyvpeilmga…'),
ProteinSequence(id=None, seq='mkgyfgpyggqyvpeilmga…'),
ProteinSequence(id=None, seq='mkgyfgpyggqyvpeilmga…'),
ProteinSequence(id=None, seq='mkgyfgpyggqyvpeilmga…'),
ProteinSequence(id=None, seq='mkgyfgpyggqyvpeilmga…'),
ProteinSequence(id=None, seq='mkgyfgpyggqyvpeilmga…'),
ProteinSequence(id=None, seq='mkgyfgpyggqyvpeilmga…'),
ProteinSequence(id=None, seq='mkgyfgpyggqyvpeilmga…'),
ProteinSequence(id=None, seq='mkgyfgpyggqyvpeilmga…'),
ProteinSequence(id=None, seq='mkgyfgpyggqyvpeilmga…'),
ProteinSequence(id=None, seq='mkgyfgpyggqyvpeilmga…'),
ProteinSequence(id=None, seq='mkgyfgpyggqyvpeilmga…'),
ProteinSequence(id=None, seq='mkgyfgpyggqyvpeilmga…'),
ProteinSequence(id=None, seq='mkgyfgpyggqyvpeilmga…'),
ProteinSequence(id=None, seq='mkgyfgpyggqyvpeilmga…'),
ProteinSequence(id=None, seq='mkgyfgpyggqyvpeilmga…'),
ProteinSequence(id=None, seq='mkgyfgpyggqyvpeilmga…'),
ProteinSequence(id=None, seq='mkgyfgpyggqyvpeilmga…'),
ProteinSequence(id=None, seq='mkgyfgpyggqyvpeilmga…'),
ProteinSequence(id=None, seq='mkgyfgpyggqyvpeilmga…'),
ProteinSequence(id=None, seq='mkgyfgpyggqyvpeilmga…'),
ProteinSequence(id=None, seq='mkgyfgpyggqyvpeilmga…'),
ProteinSequence(id=None, seq='mkgyfgpyggqyvpeilmga…'),
ProteinSequence(id=None, seq='mkgyfgpyggqyvpeilmga…'),
ProteinSequence(id=None, seq='mkgyfgpyggqyvpeilmga…'),
ProteinSequence(id=None, seq='mkgyfgpyggqyvpeilmga…'),
ProteinSequence(id=None, seq='mkgyfgpyggqyvpeilmga…'),
ProteinSequence(id=None, seq='mkgyfgpyggqyvpeilmga…'),
ProteinSequence(id=None, seq='mkgyfgpyggqyvpeilmga…'),
ProteinSequence(id=None, seq='mkgyfgpyggqyvpeilmga…'),
ProteinSequence(id=None, seq='mkgyfgpyggqyvpeilmga…'),
ProteinSequence(id=None, seq='mkgyfgpyggqyvpeilmga…'),
ProteinSequence(id=None, seq='mkgyfgpyggqyvpeilmga…'),
ProteinSequence(id=None, seq='mkgyfgpyggqyvpeilmga…'),
ProteinSequence(id=None, seq='mkgyfgpyggqyvpeilmga…'),
ProteinSequence(id=None, seq='mkgyfgpyggqyvpeilmga…'),
ProteinSequence(id=None, seq='mkgyfgpyggqyvpeilmga…'),
ProteinSequence(id=None, seq='mkgyfgpyggqyvpeilmga…'),
```

```
ProteinSequence(id=None, seq='mkgyfgpyggqyvpeilmga…'),
ProteinSequence(id=None, seq='mkgyfgpyggqyvpeilmga…'),
ProteinSequence(id=None, seq='mkgyfgpyggqyvpeilmga…'),
ProteinSequence(id=None, seq='mkgyfgpyggqyvpeilmga…'),
ProteinSequence(id=None, seq='mkgyfgpyggqyvpeilmga…'),
ProteinSequence(id=None, seq='mkgyfgpyggqyvpeilmga…'),
ProteinSequence(id=None, seq='mkgyfgpyggqyvpeilmga…'),
ProteinSequence(id=None, seq='mkgyfgpyggqyvpeilmga…'),
ProteinSequence(id=None, seq='mkgyfgpyggqyvpeilmga…'),
ProteinSequence(id=None, seq='mkgyfgpyggqyvpeilmga…'),
ProteinSequence(id=None, seq='mkgyfgpyggqyvpeilmga…'),
ProteinSequence(id=None, seq='mkgyfgpyggqyvpeilmga…'),
ProteinSequence(id=None, seq='mkgyfgpyggqyvpeilmga…'),
ProteinSequence(id=None, seq='mkgyfgpyggqyvpeilmga…'),
ProteinSequence(id=None, seq='mkgyfgpyggqyvpeilmga…'),
ProteinSequence(id=None, seq='mkgyfgpyggqyvpeilmga…'),
ProteinSequence(id=None, seq='mkgyfgpyggqyvpeilmga…'),
ProteinSequence(id=None, seq='mkgyfgpyggqyvpeilmga…'),
ProteinSequence(id=None, seq='mkgyfgpyggqyvpeilmga…'),
ProteinSequence(id=None, seq='mkgyfgpyggqyvpeilmga…'),
ProteinSequence(id=None, seq='mkgyfgpyggqyvpeilmga…'),
ProteinSequence(id=None, seq='mkgyfgpyggqyvpeilmga…'),
ProteinSequence(id=None, seq='mkgyfgpyggqyvpeilmga…'),
ProteinSequence(id=None, seq='mkgyfgpyggqyvpeilmga…'),
ProteinSequence(id=None, seq='mkgyfgpyggqyvpeilmga…'),
ProteinSequence(id=None, seq='mkgyfgpyggqyvpeilmga…'),
ProteinSequence(id=None, seq='mkgyfgpyggqyvpeilmga…'),
ProteinSequence(id=None, seq='mkgyfgpyggqyvpeilmga…'),
ProteinSequence(id=None, seq='mkgyfgpyggqyvpeilmga…'),
ProteinSequence(id=None, seq='mkgyfgpyggqyvpeilmga…'),
ProteinSequence(id=None, seq='mkgyfgpyggqyvpeilmga…'),
ProteinSequence(id=None, seq='mkgyfgpyggqyvpeilmga…'),
ProteinSequence(id=None, seq='mkgyfgpyggqyvpeilmga…'),
ProteinSequence(id=None, seq='mkgyfgpyggqyvpeilmga…'),
ProteinSequence(id=None, seq='mkgyfgpyggqyvpeilmga…'),
ProteinSequence(id=None, seq='mkgyfgpyggqyvpeilmga…'),
ProteinSequence(id=None, seq='mkgyfgpyggqyvpeilmga…'),
ProteinSequence(id=None, seq='mkgyfgpyggqyvpeilmga…'),
ProteinSequence(id=None, seq='mkgyfgpyggqyvpeilmga…'),
ProteinSequence(id=None, seq='mkgyfgpyggqyvpeilmga…'),
ProteinSequence(id=None, seq='mkgyfgpyggqyvpeilmga…'),
ProteinSequence(id=None, seq='mkgyfgpyggqyvpeilmga…'),
ProteinSequence(id=None, seq='mkgyfgpyggqyvpeilmga…'),
ProteinSequence(id=None, seq='mkgyfgpyggqyvpeilmga…'),
ProteinSequence(id=None, seq='mkgyfgpyggqyvpeilmga…'),
ProteinSequence(id=None, seq='mkgyfgpyggqyvpeilmga…'),
ProteinSequence(id=None, seq='mkgyfgpyggqyvpeilmga…'),
ProteinSequence(id=None, seq='mkgyfgpyggqyvpeilmga…'),
```

```
ProteinSequence(id=None, seq='mkgyfgpyggqyvpeilmga…'),
ProteinSequence(id=None, seq='mkgyfgpyggqyvpeilmga…'),
ProteinSequence(id=None, seq='mkgyfgpyggqyvpeilmga…'),
ProteinSequence(id=None, seq='mkgyfgpyggqyvpeilmga…'),
ProteinSequence(id=None, seq='mkgyfgpyggqyvpeilmga…'),
ProteinSequence(id=None, seq='mkgyfgpyggqyvpeilmga…'),
ProteinSequence(id=None, seq='mkgyfgpyggqyvpeilmga…'),
ProteinSequence(id=None, seq='mkgyfgpyggqyvpeilmga…'),
ProteinSequence(id=None, seq='mkgyfgpyggqyvpeilmga…'),
ProteinSequence(id=None, seq='mkgyfgpyggqyvpeilmga…'),
ProteinSequence(id=None, seq='mkgyfgpyggqyvpeilmga…'),
ProteinSequence(id=None, seq='mkgyfgpyggqyvpeilmga…'),
ProteinSequence(id=None, seq='mkgyfgpyggqyvpeilmga…'),
ProteinSequence(id=None, seq='mkgyfgpyggqyvpeilmga…'),
ProteinSequence(id=None, seq='mkgyfgpyggqyvpeilmga…'),
ProteinSequence(id=None, seq='mkgyfgpyggqyvpeilmga…'),
ProteinSequence(id=None, seq='mkgyfgpyggqyvpeilmga…'),
ProteinSequence(id=None, seq='mkgyfgpyggqyvpeilmga…'),
ProteinSequence(id=None, seq='mkgyfgpyggqyvpeilmga…'),
ProteinSequence(id=None, seq='mkgyfgpyggqyvpeilmga…'),
ProteinSequence(id=None, seq='mkgyfgpyggqyvpeilmga…'),
ProteinSequence(id=None, seq='mkgyfgpyggqyvpeilmga…'),
ProteinSequence(id=None, seq='mkgyfgpyggqyvpeilmga…'),
ProteinSequence(id=None, seq='mkgyfgpyggqyvpeilmga…'),
ProteinSequence(id=None, seq='mkgyfgpyggqyvpeilmga…'),
ProteinSequence(id=None, seq='mkgyfgpyggqyvpeilmga…'),
ProteinSequence(id=None, seq='mkgyfgpyggqyvpeilmga…'),
ProteinSequence(id=None, seq='mkgyfgpyggqyvpeilmga…'),
ProteinSequence(id=None, seq='mkgyfgpyggqyvpeilmga…'),
ProteinSequence(id=None, seq='mkgyfgpyggqyvpeilmga…'),
ProteinSequence(id=None, seq='mkgyfgpyggqyvpeilmga…'),
ProteinSequence(id=None, seq='mkgyfgpyggqyvpeilmga…'),
ProteinSequence(id=None, seq='mkgyfgpyggqyvpeilmga…'),
ProteinSequence(id=None, seq='mkgyfgpyggqyvpeilmga…'),
ProteinSequence(id=None, seq='mkgyfgpyggqyvpeilmga…'),
ProteinSequence(id=None, seq='mkgyfgpyggqyvpeilmga…'),
ProteinSequence(id=None, seq='mkgyfgpyggqyvpeilmga…'),
ProteinSequence(id=None, seq='mkgyfgpyggqyvpeilmga…'),
ProteinSequence(id=None, seq='mkgyfgpyggqyvpeilmga…'),
ProteinSequence(id=None, seq='mkgyfgpyggqyvpeilmga…'),
ProteinSequence(id=None, seq='mkgyfgpyggqyvpeilmga…'),
ProteinSequence(id=None, seq='mkgyfgpyggqyvpeilmga…'),
ProteinSequence(id=None, seq='mkgyfgpyggqyvpeilmga…'),
ProteinSequence(id=None, seq='mkgyfgpyggqyvpeilmga…'),
ProteinSequence(id=None, seq='mkgyfgpyggqyvpeilmga…'),
ProteinSequence(id=None, seq='mkgyfgpyggqyvpeilmga…'),
ProteinSequence(id=None, seq='mkgyfgpyggqyvpeilmga…'),
ProteinSequence(id=None, seq='mkgyfgpyggqyvpeilmga…'),
```

```
ProteinSequence(id=None, seq='mkgyfgpyggqyvpeilmga…'),
ProteinSequence(id=None, seq='mkgyfgpyggqyvpeilmga…'),
ProteinSequence(id=None, seq='mkgyfgpyggqyvpeilmga…'),
ProteinSequence(id=None, seq='mkgyfgpyggqyvpeilmga…'),
ProteinSequence(id=None, seq='mkgyfgpyggqyvpeilmga…'),
ProteinSequence(id=None, seq='mkgyfgpyggqyvpeilmga…'),
ProteinSequence(id=None, seq='mkgyfgpyggqyvpeilmga…'),
ProteinSequence(id=None, seq='mkgyfgpyggqyvpeilmga…'),
ProteinSequence(id=None, seq='mkgyfgpyggqyvpeilmga…'),
ProteinSequence(id=None, seq='mkgyfgpyggqyvpeilmga…'),
ProteinSequence(id=None, seq='mkgyfgpyggqyvpeilmga…'),
ProteinSequence(id=None, seq='mkgyfgpyggqyvpeilmga…'),
ProteinSequence(id=None, seq='mkgyfgpyggqyvpeilmga…'),
ProteinSequence(id=None, seq='mkgyfgpyggqyvpeilmga…'),
ProteinSequence(id=None, seq='mkgyfgpyggqyvpeilmga…'),
ProteinSequence(id=None, seq='mkgyfgpyggqyvpeilmga…'),
ProteinSequence(id=None, seq='mkgyfgpyggqyvpeilmga…'),
ProteinSequence(id=None, seq='mkgyfgpyggqyvpeilmga…'),
ProteinSequence(id=None, seq='mkgyfgpyggqyvpeilmga…'),
ProteinSequence(id=None, seq='mkgyfgpyggqyvpeilmga…'),
ProteinSequence(id=None, seq='mkgyfgpyggqyvpeilmga…'),
ProteinSequence(id=None, seq='mkgyfgpyggqyvpeilmga…'),
ProteinSequence(id=None, seq='mkgyfgpyggqyvpeilmga…'),
ProteinSequence(id=None, seq='mkgyfgpyggqyvpeilmga…'),
ProteinSequence(id=None, seq='mkgyfgpyggqyvpeilmga…'),
ProteinSequence(id=None, seq='mkgyfgpyggqyvpeilmga…'),
ProteinSequence(id=None, seq='mkgyfgpyggqyvpeilmga…'),
ProteinSequence(id=None, seq='mkgyfgpyggqyvpeilmga…'),
ProteinSequence(id=None, seq='mkgyfgpyggqyvpeilmga…'),
ProteinSequence(id=None, seq='mkgyfgpyggqyvpeilmga…'),
ProteinSequence(id=None, seq='mkgyfgpyggqyvpeilmga…'),
ProteinSequence(id=None, seq='mkgyfgpyggqyvpeilmga…'),
ProteinSequence(id=None, seq='mkgyfgpyggqyvpeilmga…'),
ProteinSequence(id=None, seq='mkgyfgpyggqyvpeilmga…'),
ProteinSequence(id=None, seq='mkgyfgpyggqyvpeilmga…'),
ProteinSequence(id=None, seq='mkgyfgpyggqyvpeilmga…'),
ProteinSequence(id=None, seq='mkgyfgpyggqyvpeilmga…'),
ProteinSequence(id=None, seq='mkgyfgpyggqyvpeilmga…'),
ProteinSequence(id=None, seq='mkgyfgpyggqyvpeilmga…'),
ProteinSequence(id=None, seq='mkgyfgpyggqyvpeilmga…'),
ProteinSequence(id=None, seq='mkgyfgpyggqyvpeilmga…'),
ProteinSequence(id=None, seq='mkgyfgpyggqyvpeilmga…'),
ProteinSequence(id=None, seq='mkgyfgpyggqyvpeilmga…'),
ProteinSequence(id=None, seq='mkgyfgpyggqyvpeilmga…'),
ProteinSequence(id=None, seq='mkgyfgpyggqyvpeilmga…'),
ProteinSequence(id=None, seq='mkgyfgpyggqyvpeilmga…'),
ProteinSequence(id=None, seq='mkgyfgpyggqyvpeilmga…'),
ProteinSequence(id=None, seq='mkgyfgpyggqyvpeilmga…'),
```

```
ProteinSequence(id=None, seq='mkgyfgpyggqyvpeilmga…'),
ProteinSequence(id=None, seq='mkgyfgpyggqyvpeilmga…'),
ProteinSequence(id=None, seq='mkgyfgpyggqyvpeilmga…'),
ProteinSequence(id=None, seq='mkgyfgpyggqyvpeilmga…'),
ProteinSequence(id=None, seq='mkgyfgpyggqyvpeilmga…'),
ProteinSequence(id=None, seq='mkgyfgpyggqyvpeilmga…'),
ProteinSequence(id=None, seq='mkgyfgpyggqyvpeilmga…'),
ProteinSequence(id=None, seq='mkgyfgpyggqyvpeilmga…'),
ProteinSequence(id=None, seq='mkgyfgpyggqyvpeilmga…'),
ProteinSequence(id=None, seq='mkgyfgpyggqyvpeilmga…'),
ProteinSequence(id=None, seq='mkgyfgpyggqyvpeilmga…'),
ProteinSequence(id=None, seq='mkgyfgpyggqyvpeilmga…'),
ProteinSequence(id=None, seq='mkgyfgpyggqyvpeilmga…'),
ProteinSequence(id=None, seq='mkgyfgpyggqyvpeilmga…'),
ProteinSequence(id=None, seq='mkgyfgpyggqyvpeilmga…'),
ProteinSequence(id=None, seq='mkgyfgpyggqyvpeilmga…'),
ProteinSequence(id=None, seq='mkgyfgpyggqyvpeilmga…'),
ProteinSequence(id=None, seq='mkgyfgpyggqyvpeilmga…'),
ProteinSequence(id=None, seq='mkgyfgpyggqyvpeilmga…'),
ProteinSequence(id=None, seq='mkgyfgpyggqyvpeilmga…'),
ProteinSequence(id=None, seq='mkgyfgpyggqyvpeilmga…'),
ProteinSequence(id=None, seq='mkgyfgpyggqyvpeilmga…'),
ProteinSequence(id=None, seq='mkgyfgpyggqyvpeilmga…'),
ProteinSequence(id=None, seq='mkgyfgpyggqyvpeilmga…'),
ProteinSequence(id=None, seq='mkgyfgpyggqyvpeilmga…'),
ProteinSequence(id=None, seq='mkgyfgpyggqyvpeilmga…'),
ProteinSequence(id=None, seq='mkgyfgpyggqyvpeilmga…'),
ProteinSequence(id=None, seq='mkgyfgpyggqyvpeilmga…'),
ProteinSequence(id=None, seq='mkgyfgpyggqyvpeilmga…'),
ProteinSequence(id=None, seq='mkgyfgpyggqyvpeilmga…'),
ProteinSequence(id=None, seq='mkgyfgpyggqyvpeilmga…'),
ProteinSequence(id=None, seq='mkgyfgpyggqyvpeilmga…'),
ProteinSequence(id=None, seq='mkgyfgpyggqyvpeilmga…'),
ProteinSequence(id=None, seq='mkgyfgpyggqyvpeilmga…'),
ProteinSequence(id=None, seq='mkgyfgpyggqyvpeilmga…'),
ProteinSequence(id=None, seq='mkgyfgpyggqyvpeilmga…'),
ProteinSequence(id=None, seq='mkgyfgpyggqyvpeilmga…'),
ProteinSequence(id=None, seq='mkgyfgpyggqyvpeilmga…'),
ProteinSequence(id=None, seq='mkgyfgpyggqyvpeilmga…'),
ProteinSequence(id=None, seq='mkgyfgpyggqyvpeilmga…'),
ProteinSequence(id=None, seq='mkgyfgpyggqyvpeilmga…'),
ProteinSequence(id=None, seq='mkgyfgpyggqyvpeilmga…'),
ProteinSequence(id=None, seq='mkgyfgpyggqyvpeilmga…'),
ProteinSequence(id=None, seq='mkgyfgpyggqyvpeilmga…'),
ProteinSequence(id=None, seq='mkgyfgpyggqyvpeilmga…'),
ProteinSequence(id=None, seq='mkgyfgpyggqyvpeilmga…'),
ProteinSequence(id=None, seq='mkgyfgpyggqyvpeilmga…'),
ProteinSequence(id=None, seq='mkgyfgpyggqyvpeilmga…'),
```

```
ProteinSequence(id=None, seq='mkgyfgpyggqyvpeilmga…'),
ProteinSequence(id=None, seq='mkgyfgpyggqyvpeilmga…'),
ProteinSequence(id=None, seq='mkgyfgpyggqyvpeilmga…'),
ProteinSequence(id=None, seq='mkgyfgpyggqyvpeilmga…'),
ProteinSequence(id=None, seq='mkgyfgpyggqyvpeilmga…'),
ProteinSequence(id=None, seq='mkgyfgpyggqyvpeilmga…'),
ProteinSequence(id=None, seq='mkgyfgpyggqyvpeilmga…'),
ProteinSequence(id=None, seq='mkgyfgpyggqyvpeilmga…'),
ProteinSequence(id=None, seq='mkgyfgpyggqyvpeilmga…'),
ProteinSequence(id=None, seq='mkgyfgpyggqyvpeilmga…'),
ProteinSequence(id=None, seq='mkgyfgpyggqyvpeilmga…'),
ProteinSequence(id=None, seq='mkgyfgpyggqyvpeilmga…'),
ProteinSequence(id=None, seq='mkgyfgpyggqyvpeilmga…'),
ProteinSequence(id=None, seq='mkgyfgpyggqyvpeilmga…'),
ProteinSequence(id=None, seq='mkgyfgpyggqyvpeilmga…'),
ProteinSequence(id=None, seq='mkgyfgpyggqyvpeilmga…'),
ProteinSequence(id=None, seq='mkgyfgpyggqyvpeilmga…'),
ProteinSequence(id=None, seq='mkgyfgpyggqyvpeilmga…'),
ProteinSequence(id=None, seq='mkgyfgpyggqyvpeilmga…'),
ProteinSequence(id=None, seq='mkgyfgpyggqyvpeilmga…'),
ProteinSequence(id=None, seq='mkgyfgpyggqyvpeilmga…'),
ProteinSequence(id=None, seq='mkgyfgpyggqyvpeilmga…'),
ProteinSequence(id=None, seq='mkgyfgpyggqyvpeilmga…'),
ProteinSequence(id=None, seq='mkgyfgpyggqyvpeilmga…'),
ProteinSequence(id=None, seq='mkgyfgpyggqyvpeilmga…'),
ProteinSequence(id=None, seq='mkgyfgpyggqyvpeilmga…'),
ProteinSequence(id=None, seq='mkgyfgpyggqyvpeilmga…'),
ProteinSequence(id=None, seq='mkgyfgpyggqyvpeilmga…'),
ProteinSequence(id=None, seq='mkgyfgpyggqyvpeilmga…'),
ProteinSequence(id=None, seq='mkgyfgpyggqyvpeilmga…'),
ProteinSequence(id=None, seq='mkgyfgpyggqyvpeilmga…'),
ProteinSequence(id=None, seq='mkgyfgpyggqyvpeilmga…'),
ProteinSequence(id=None, seq='mkgyfgpyggqyvpeilmga…'),
ProteinSequence(id=None, seq='mkgyfgpyggqyvpeilmga…'),
ProteinSequence(id=None, seq='mkgyfgpyggqyvpeilmga…'),
ProteinSequence(id=None, seq='mkgyfgpyggqyvpeilmga…'),
ProteinSequence(id=None, seq='mkgyfgpyggqyvpeilmga…'),
ProteinSequence(id=None, seq='mkgyfgpyggqyvpeilmga…'),
ProteinSequence(id=None, seq='mkgyfgpyggqyvpeilmga…'),
ProteinSequence(id=None, seq='mkgyfgpyggqyvpeilmga…'),
ProteinSequence(id=None, seq='mkgyfgpyggqyvpeilmga…'),
ProteinSequence(id=None, seq='mkgyfgpyggqyvpeilmga…'),
ProteinSequence(id=None, seq='mkgyfgpyggqyvpeilmga…'),
ProteinSequence(id=None, seq='mkgyfgpyggqyvpeilmga…'),
ProteinSequence(id=None, seq='mkgyfgpyggqyvpeilmga…'),
ProteinSequence(id=None, seq='mkgyfgpyggqyvpeilmga…'),
ProteinSequence(id=None, seq='mkgyfgpyggqyvpeilmga…'),
ProteinSequence(id=None, seq='mkgyfgpyggqyvpeilmga…'),
```

```
ProteinSequence(id=None, seq='mkgyfgpyggqyvpeilmga…'),
ProteinSequence(id=None, seq='mkgyfgpyggqyvpeilmga…'),
ProteinSequence(id=None, seq='mkgyfgpyggqyvpeilmga…'),
ProteinSequence(id=None, seq='mkgyfgpyggqyvpeilmga…'),
ProteinSequence(id=None, seq='mkgyfgpyggqyvpeilmga…'),
ProteinSequence(id=None, seq='mkgyfgpyggqyvpeilmga…'),
ProteinSequence(id=None, seq='mkgyfgpyggqyvpeilmga…'),
ProteinSequence(id=None, seq='mkgyfgpyggqyvpeilmga…'),
ProteinSequence(id=None, seq='mkgyfgpyggqyvpeilmga…'),
ProteinSequence(id=None, seq='mkgyfgpyggqyvpeilmga…'),
ProteinSequence(id=None, seq='mkgyfgpyggqyvpeilmga…'),
ProteinSequence(id=None, seq='mkgyfgpyggqyvpeilmga…'),
ProteinSequence(id=None, seq='mkgyfgpyggqyvpeilmga…'),
ProteinSequence(id=None, seq='mkgyfgpyggqyvpeilmga…'),
ProteinSequence(id=None, seq='mkgyfgpyggqyvpeilmga…'),
ProteinSequence(id=None, seq='mkgyfgpyggqyvpeilmga…'),
ProteinSequence(id=None, seq='mkgyfgpyggqyvpeilmga…'),
ProteinSequence(id=None, seq='mkgyfgpyggqyvpeilmga…'),
ProteinSequence(id=None, seq='mkgyfgpyggqyvpeilmga…'),
ProteinSequence(id=None, seq='mkgyfgpyggqyvpeilmga…'),
ProteinSequence(id=None, seq='mkgyfgpyggqyvpeilmga…'),
ProteinSequence(id=None, seq='mkgyfgpyggqyvpeilmga…'),
ProteinSequence(id=None, seq='mkgyfgpyggqyvpeilmga…'),
ProteinSequence(id=None, seq='mkgyfgpyggqyvpeilmga…'),
ProteinSequence(id=None, seq='mkgyfgpyggqyvpeilmga…'),
ProteinSequence(id=None, seq='mkgyfgpyggqyvpeilmga…'),
ProteinSequence(id=None, seq='mkgyfgpyggqyvpeilmga…'),
ProteinSequence(id=None, seq='mkgyfgpyggqyvpeilmga…'),
ProteinSequence(id=None, seq='mkgyfgpyggqyvpeilmga…'),
ProteinSequence(id=None, seq='mkgyfgpyggqyvpeilmga…'),
ProteinSequence(id=None, seq='mkgyfgpyggqyvpeilmga…'),
ProteinSequence(id=None, seq='mkgyfgpyggqyvpeilmga…'),
ProteinSequence(id=None, seq='mkgyfgpyggqyvpeilmga…'),
ProteinSequence(id=None, seq='mkgyfgpyggqyvpeilmga…'),
ProteinSequence(id=None, seq='mkgyfgpyggqyvpeilmga…'),
ProteinSequence(id=None, seq='mkgyfgpyggqyvpeilmga…'),
ProteinSequence(id=None, seq='mkgyfgpyggqyvpeilmga…'),
ProteinSequence(id=None, seq='mkgyfgpyggqyvpeilmga…'),
ProteinSequence(id=None, seq='mkgyfgpyggqyvpeilmga…'),
ProteinSequence(id=None, seq='mkgyfgpyggqyvpeilmga…'),
ProteinSequence(id=None, seq='mkgyfgpyggqyvpeilmga…'),
ProteinSequence(id=None, seq='mkgyfgpyggqyvpeilmga…'),
ProteinSequence(id=None, seq='mkgyfgpyggqyvpeilmga…'),
ProteinSequence(id=None, seq='mkgyfgpyggqyvpeilmga…'),
ProteinSequence(id=None, seq='mkgyfgpyggqyvpeilmga…'),
ProteinSequence(id=None, seq='mkgyfgpyggqyvpeilmga…'),
ProteinSequence(id=None, seq='mkgyfgpyggqyvpeilmga…'),
ProteinSequence(id=None, seq='mkgyfgpyggqyvpeilmga…'),
```

```
ProteinSequence(id=None, seq='mkgyfgpyggqyvpeilmga…'),
ProteinSequence(id=None, seq='mkgyfgpyggqyvpeilmga…'),
ProteinSequence(id=None, seq='mkgyfgpyggqyvpeilmga…'),
ProteinSequence(id=None, seq='mkgyfgpyggqyvpeilmga…'),
ProteinSequence(id=None, seq='mkgyfgpyggqyvpeilmga…'),
ProteinSequence(id=None, seq='mkgyfgpyggqyvpeilmga…'),
ProteinSequence(id=None, seq='mkgyfgpyggqyvpeilmga…'),
ProteinSequence(id=None, seq='mkgyfgpyggqyvpeilmga…'),
ProteinSequence(id=None, seq='mkgyfgpyggqyvpeilmga…'),
ProteinSequence(id=None, seq='mkgyfgpyggqyvpeilmga…'),
ProteinSequence(id=None, seq='mkgyfgpyggqyvpeilmga…'),
ProteinSequence(id=None, seq='mkgyfgpyggqyvpeilmga…'),
ProteinSequence(id=None, seq='mkgyfgpyggqyvpeilmga…'),
ProteinSequence(id=None, seq='mkgyfgpyggqyvpeilmga…'),
ProteinSequence(id=None, seq='mkgyfgpyggqyvpeilmga…'),
ProteinSequence(id=None, seq='mkgyfgpyggqyvpeilmga…'),
ProteinSequence(id=None, seq='mkgyfgpyggqyvpeilmga…'),
ProteinSequence(id=None, seq='mkgyfgpyggqyvpeilmga…'),
ProteinSequence(id=None, seq='mkgyfgpyggqyvpeilmga…'),
ProteinSequence(id=None, seq='mkgyfgpyggqyvpeilmga…'),
ProteinSequence(id=None, seq='mkgyfgpyggqyvpeilmga…'),
ProteinSequence(id=None, seq='mkgyfgpyggqyvpeilmga…'),
ProteinSequence(id=None, seq='mkgyfgpyggqyvpeilmga…'),
ProteinSequence(id=None, seq='mkgyfgpyggqyvpeilmga…'),
ProteinSequence(id=None, seq='mkgyfgpyggqyvpeilmga…'),
ProteinSequence(id=None, seq='mkgyfgpyggqyvpeilmga…'),
ProteinSequence(id=None, seq='mkgyfgpyggqyvpeilmga…'),
ProteinSequence(id=None, seq='mkgyfgpyggqyvpeilmga…'),
ProteinSequence(id=None, seq='mkgyfgpyggqyvpeilmga…'),
ProteinSequence(id=None, seq='mkgyfgpyggqyvpeilmga…'),
ProteinSequence(id=None, seq='mkgyfgpyggqyvpeilmga…'),
ProteinSequence(id=None, seq='mkgyfgpyggqyvpeilmga…'),
ProteinSequence(id=None, seq='mkgyfgpyggqyvpeilmga…'),
ProteinSequence(id=None, seq='mkgyfgpyggqyvpeilmga…'),
ProteinSequence(id=None, seq='mkgyfgpyggqyvpeilmga…'),
ProteinSequence(id=None, seq='mkgyfgpyggqyvpeilmga…'),
ProteinSequence(id=None, seq='mkgyfgpyggqyvpeilmga…'),
ProteinSequence(id=None, seq='mkgyfgpyggqyvpeilmga…'),
ProteinSequence(id=None, seq='mkgyfgpyggqyvpeilmga…'),
ProteinSequence(id=None, seq='mkgyfgpyggqyvpeilmga…'),
ProteinSequence(id=None, seq='mkgyfgpyggqyvpeilmga…'),
ProteinSequence(id=None, seq='mkgyfgpyggqyvpeilmga…'),
ProteinSequence(id=None, seq='mkgyfgpyggqyvpeilmga…'),
ProteinSequence(id=None, seq='mkgyfgpyggqyvpeilmga…'),
ProteinSequence(id=None, seq='mkgyfgpyggqyvpeilmga…'),
ProteinSequence(id=None, seq='mkgyfgpyggqyvpeilmga…'),
ProteinSequence(id=None, seq='mkgyfgpyggqyvpeilmga…'),
ProteinSequence(id=None, seq='mkgyfgpyggqyvpeilmga…'),
```

```
ProteinSequence(id=None, seq='mkgyfgpyggqyvpeilmga…'),
ProteinSequence(id=None, seq='mkgyfgpyggqyvpeilmga…'),
ProteinSequence(id=None, seq='mkgyfgpyggqyvpeilmga…'),
ProteinSequence(id=None, seq='mkgyfgpyggqyvpeilmga…'),
ProteinSequence(id=None, seq='mkgyfgpyggqyvpeilmga…'),
ProteinSequence(id=None, seq='mkgyfgpyggqyvpeilmga…'),
ProteinSequence(id=None, seq='mkgyfgpyggqyvpeilmga…'),
ProteinSequence(id=None, seq='mkgyfgpyggqyvpeilmga…'),
ProteinSequence(id=None, seq='mkgyfgpyggqyvpeilmga…'),
ProteinSequence(id=None, seq='mkgyfgpyggqyvpeilmga…'),
ProteinSequence(id=None, seq='mkgyfgpyggqyvpeilmga…'),
ProteinSequence(id=None, seq='mkgyfgpyggqyvpeilmga…'),
ProteinSequence(id=None, seq='mkgyfgpyggqyvpeilmga…'),
ProteinSequence(id=None, seq='mkgyfgpyggqyvpeilmga…'),
ProteinSequence(id=None, seq='mkgyfgpyggqyvpeilmga…'),
ProteinSequence(id=None, seq='mkgyfgpyggqyvpeilmga…'),
ProteinSequence(id=None, seq='mkgyfgpyggqyvpeilmga…'),
ProteinSequence(id=None, seq='mkgyfgpyggqyvpeilmga…'),
ProteinSequence(id=None, seq='mkgyfgpyggqyvpeilmga…'),
ProteinSequence(id=None, seq='mkgyfgpyggqyvpeilmga…'),
ProteinSequence(id=None, seq='mkgyfgpyggqyvpeilmga…'),
ProteinSequence(id=None, seq='mkgyfgpyggqyvpeilmga…'),
ProteinSequence(id=None, seq='mkgyfgpyggqyvpeilmga…'),
ProteinSequence(id=None, seq='mkgyfgpyggqyvpeilmga…'),
ProteinSequence(id=None, seq='mkgyfgpyggqyvpeilmga…'),
ProteinSequence(id=None, seq='mkgyfgpyggqyvpeilmga…'),
ProteinSequence(id=None, seq='mkgyfgpyggqyvpeilmga…'),
ProteinSequence(id=None, seq='mkgyfgpyggqyvpeilmga…'),
ProteinSequence(id=None, seq='mkgyfgpyggqyvpeilmga…'),
ProteinSequence(id=None, seq='mkgyfgpyggqyvpeilmga…'),
ProteinSequence(id=None, seq='mkgyfgpyggqyvpeilmga…'),
ProteinSequence(id=None, seq='mkgyfgpyggqyvpeilmga…'),
ProteinSequence(id=None, seq='mkgyfgpyggqyvpeilmga…'),
ProteinSequence(id=None, seq='mkgyfgpyggqyvpeilmga…'),
ProteinSequence(id=None, seq='mkgyfgpyggqyvpeilmga…'),
ProteinSequence(id=None, seq='mkgyfgpyggqyvpeilmga…'),
ProteinSequence(id=None, seq='mkgyfgpyggqyvpeilmga…'),
ProteinSequence(id=None, seq='mkgyfgpyggqyvpeilmga…'),
ProteinSequence(id=None, seq='mkgyfgpyggqyvpeilmga…'),
ProteinSequence(id=None, seq='mkgyfgpyggqyvpeilmga…'),
ProteinSequence(id=None, seq='mkgyfgpyggqyvpeilmga…'),
ProteinSequence(id=None, seq='mkgyfgpyggqyvpeilmga…'),
ProteinSequence(id=None, seq='mkgyfgpyggqyvpeilmga…'),
ProteinSequence(id=None, seq='mkgyfgpyggqyvpeilmga…'),
ProteinSequence(id=None, seq='mkgyfgpyggqyvpeilmga…'),
ProteinSequence(id=None, seq='mkgyfgpyggqyvpeilmga…'),
ProteinSequence(id=None, seq='mkgyfgpyggqyvpeilmga…'),
ProteinSequence(id=None, seq='mkgyfgpyggqyvpeilmga…'),
```

```
ProteinSequence(id=None, seq='mkgyfgpyggqyvpeilmga…'),
ProteinSequence(id=None, seq='mkgyfgpyggqyvpeilmga…'),
ProteinSequence(id=None, seq='mkgyfgpyggqyvpeilmga…'),
ProteinSequence(id=None, seq='mkgyfgpyggqyvpeilmga…'),
ProteinSequence(id=None, seq='mkgyfgpyggqyvpeilmga…'),
ProteinSequence(id=None, seq='mkgyfgpyggqyvpeilmga…'),
ProteinSequence(id=None, seq='mkgyfgpyggqyvpeilmga…'),
ProteinSequence(id=None, seq='mkgyfgpyggqyvpeilmga…'),
ProteinSequence(id=None, seq='mkgyfgpyggqyvpeilmga…'),
ProteinSequence(id=None, seq='mkgyfgpyggqyvpeilmga…'),
ProteinSequence(id=None, seq='mkgyfgpyggqyvpeilmga…'),
ProteinSequence(id=None, seq='mkgyfgpyggqyvpeilmga…'),
ProteinSequence(id=None, seq='mkgyfgpyggqyvpeilmga…'),
ProteinSequence(id=None, seq='mkgyfgpyggqyvpeilmga…'),
ProteinSequence(id=None, seq='mkgyfgpyggqyvpeilmga…'),
ProteinSequence(id=None, seq='mkgyfgpyggqyvpeilmga…'),
ProteinSequence(id=None, seq='mkgyfgpyggqyvpeilmga…'),
ProteinSequence(id=None, seq='mkgyfgpyggqyvpeilmga…'),
ProteinSequence(id=None, seq='mkgyfgpyggqyvpeilmga…'),
ProteinSequence(id=None, seq='mkgyfgpyggqyvpeilmga…'),
ProteinSequence(id=None, seq='mkgyfgpyggqyvpeilmga…'),
ProteinSequence(id=None, seq='mkgyfgpyggqyvpeilmga…'),
ProteinSequence(id=None, seq='mkgyfgpyggqyvpeilmga…'),
ProteinSequence(id=None, seq='mkgyfgpyggqyvpeilmga…'),
ProteinSequence(id=None, seq='mkgyfgpyggqyvpeilmga…'),
ProteinSequence(id=None, seq='mkgyfgpyggqyvpeilmga…'),
ProteinSequence(id=None, seq='mkgyfgpyggqyvpeilmga…'),
ProteinSequence(id=None, seq='mkgyfgpyggqyvpeilmga…'),
ProteinSequence(id=None, seq='mkgyfgpyggqyvpeilmga…'),
ProteinSequence(id=None, seq='mkgyfgpyggqyvpeilmga…'),
ProteinSequence(id=None, seq='mkgyfgpyggqyvpeilmga…'),
ProteinSequence(id=None, seq='mkgyfgpyggqyvpeilmga…'),
ProteinSequence(id=None, seq='mkgyfgpyggqyvpeilmga…'),
ProteinSequence(id=None, seq='mkgyfgpyggqyvpeilmga…'),
ProteinSequence(id=None, seq='mkgyfgpyggqyvpeilmga…'),
ProteinSequence(id=None, seq='mkgyfgpyggqyvpeilmga…'),
ProteinSequence(id=None, seq='mkgyfgpyggqyvpeilmga…'),
ProteinSequence(id=None, seq='mkgyfgpyggqyvpeilmga…'),
ProteinSequence(id=None, seq='mkgyfgpyggqyvpeilmga…'),
ProteinSequence(id=None, seq='mkgyfgpyggqyvpeilmga…'),
ProteinSequence(id=None, seq='mkgyfgpyggqyvpeilmga…'),
ProteinSequence(id=None, seq='mkgyfgpyggqyvpeilmga…'),
ProteinSequence(id=None, seq='mkgyfgpyggqyvpeilmga…'),
ProteinSequence(id=None, seq='mkgyfgpyggqyvpeilmga…'),
ProteinSequence(id=None, seq='mkgyfgpyggqyvpeilmga…'),
ProteinSequence(id=None, seq='mkgyfgpyggqyvpeilmga…'),
ProteinSequence(id=None, seq='mkgyfgpyggqyvpeilmga…'),
ProteinSequence(id=None, seq='mkgyfgpyggqyvpeilmga…'),
```

```
ProteinSequence(id=None, seq='mkgyfgpyggqyvpeilmga…'),
ProteinSequence(id=None, seq='mkgyfgpyggqyvpeilmga…'),
ProteinSequence(id=None, seq='mkgyfgpyggqyvpeilmga…'),
ProteinSequence(id=None, seq='mkgyfgpyggqyvpeilmga…'),
ProteinSequence(id=None, seq='mkgyfgpyggqyvpeilmga…'),
ProteinSequence(id=None, seq='mkgyfgpyggqyvpeilmga…'),
ProteinSequence(id=None, seq='mkgyfgpyggqyvpeilmga…'),
ProteinSequence(id=None, seq='mkgyfgpyggqyvpeilmga…'),
ProteinSequence(id=None, seq='mkgyfgpyggqyvpeilmga…'),
ProteinSequence(id=None, seq='mkgyfgpyggqyvpeilmga…'),
ProteinSequence(id=None, seq='mkgyfgpyggqyvpeilmga…'),
ProteinSequence(id=None, seq='mkgyfgpyggqyvpeilmga…'),
ProteinSequence(id=None, seq='mkgyfgpyggqyvpeilmga…'),
ProteinSequence(id=None, seq='mkgyfgpyggqyvpeilmga…'),
ProteinSequence(id=None, seq='mkgyfgpyggqyvpeilmga…'),
ProteinSequence(id=None, seq='mkgyfgpyggqyvpeilmga…'),
ProteinSequence(id=None, seq='mkgyfgpyggqyvpeilmga…'),
ProteinSequence(id=None, seq='mkgyfgpyggqyvpeilmga…'),
ProteinSequence(id=None, seq='mkgyfgpyggqyvpeilmga…'),
ProteinSequence(id=None, seq='mkgyfgpyggqyvpeilmga…'),
ProteinSequence(id=None, seq='mkgyfgpyggqyvpeilmga…'),
ProteinSequence(id=None, seq='mkgyfgpyggqyvpeilmga…'),
ProteinSequence(id=None, seq='mkgyfgpyggqyvpeilmga…'),
ProteinSequence(id=None, seq='mkgyfgpyggqyvpeilmga…'),
ProteinSequence(id=None, seq='mkgyfgpyggqyvpeilmga…'),
ProteinSequence(id=None, seq='mkgyfgpyggqyvpeilmga…'),
ProteinSequence(id=None, seq='mkgyfgpyggqyvpeilmga…'),
ProteinSequence(id=None, seq='mkgyfgpyggqyvpeilmga…'),
ProteinSequence(id=None, seq='mkgyfgpyggqyvpeilmga…'),
ProteinSequence(id=None, seq='mkgyfgpyggqyvpeilmga…'),
ProteinSequence(id=None, seq='mkgyfgpyggqyvpeilmga…'),
ProteinSequence(id=None, seq='mkgyfgpyggqyvpeilmga…'),
ProteinSequence(id=None, seq='mkgyfgpyggqyvpeilmga…'),
ProteinSequence(id=None, seq='mkgyfgpyggqyvpeilmga…'),
ProteinSequence(id=None, seq='mkgyfgpyggqyvpeilmga…'),
ProteinSequence(id=None, seq='mkgyfgpyggqyvpeilmga…'),
ProteinSequence(id=None, seq='mkgyfgpyggqyvpeilmga…'),
ProteinSequence(id=None, seq='mkgyfgpyggqyvpeilmga…'),
ProteinSequence(id=None, seq='mkgyfgpyggqyvpeilmga…'),
ProteinSequence(id=None, seq='mkgyfgpyggqyvpeilmga…'),
ProteinSequence(id=None, seq='mkgyfgpyggqyvpeilmga…'),
ProteinSequence(id=None, seq='mkgyfgpyggqyvpeilmga…'),
ProteinSequence(id=None, seq='mkgyfgpyggqyvpeilmga…'),
ProteinSequence(id=None, seq='mkgyfgpyggqyvpeilmga…'),
ProteinSequence(id=None, seq='mkgyfgpyggqyvpeilmga…'),
ProteinSequence(id=None, seq='mkgyfgpyggqyvpeilmga…'),
ProteinSequence(id=None, seq='mkgyfgpyggqyvpeilmga…'),
ProteinSequence(id=None, seq='mkgyfgpyggqyvpeilmga…'),
```

```
ProteinSequence(id=None, seq='mkgyfgpyggqyvpeilmga…'),
ProteinSequence(id=None, seq='mkgyfgpyggqyvpeilmga…'),
ProteinSequence(id=None, seq='mkgyfgpyggqyvpeilmga…'),
ProteinSequence(id=None, seq='mkgyfgpyggqyvpeilmga…'),
ProteinSequence(id=None, seq='mkgyfgpyggqyvpeilmga…'),
ProteinSequence(id=None, seq='mkgyfgpyggqyvpeilmga…'),
ProteinSequence(id=None, seq='mkgyfgpyggqyvpeilmga…'),
ProteinSequence(id=None, seq='mkgyfgpyggqyvpeilmga…'),
ProteinSequence(id=None, seq='mkgyfgpyggqyvpeilmga…'),
ProteinSequence(id=None, seq='mkgyfgpyggqyvpeilmga…'),
ProteinSequence(id=None, seq='mkgyfgpyggqyvpeilmga…'),
ProteinSequence(id=None, seq='mkgyfgpyggqyvpeilmga…'),
ProteinSequence(id=None, seq='mkgyfgpyggqyvpeilmga…'),
ProteinSequence(id=None, seq='mkgyfgpyggqyvpeilmga…'),
ProteinSequence(id=None, seq='mkgyfgpyggqyvpeilmga…'),
ProteinSequence(id=None, seq='mkgyfgpyggqyvpeilmga…'),
ProteinSequence(id=None, seq='mkgyfgpyggqyvpeilmga…'),
ProteinSequence(id=None, seq='mkgyfgpyggqyvpeilmga…'),
ProteinSequence(id=None, seq='mkgyfgpyggqyvpeilmga…'),
ProteinSequence(id=None, seq='mkgyfgpyggqyvpeilmga…'),
ProteinSequence(id=None, seq='mkgyfgpyggqyvpeilmga…'),
ProteinSequence(id=None, seq='mkgyfgpyggqyvpeilmga…'),
ProteinSequence(id=None, seq='mkgyfgpyggqyvpeilmga…'),
ProteinSequence(id=None, seq='mkgyfgpyggqyvpeilmga…'),
ProteinSequence(id=None, seq='mkgyfgpyggqyvpeilmga…'),
ProteinSequence(id=None, seq='mkgyfgpyggqyvpeilmga…'),
ProteinSequence(id=None, seq='mkgyfgpyggqyvpeilmga…'),
ProteinSequence(id=None, seq='mkgyfgpyggqyvpeilmga…'),
ProteinSequence(id=None, seq='mkgyfgpyggqyvpeilmga…'),
ProteinSequence(id=None, seq='mkgyfgpyggqyvpeilmga…'),
ProteinSequence(id=None, seq='mkgyfgpyggqyvpeilmga…'),
ProteinSequence(id=None, seq='mkgyfgpyggqyvpeilmga…'),
ProteinSequence(id=None, seq='mkgyfgpyggqyvpeilmga…'),
ProteinSequence(id=None, seq='mkgyfgpyggqyvpeilmga…'),
ProteinSequence(id=None, seq='mkgyfgpyggqyvpeilmga…'),
ProteinSequence(id=None, seq='mkgyfgpyggqyvpeilmga…'),
ProteinSequence(id=None, seq='mkgyfgpyggqyvpeilmga…'),
ProteinSequence(id=None, seq='mkgyfgpyggqyvpeilmga…'),
ProteinSequence(id=None, seq='mkgyfgpyggqyvpeilmga…'),
ProteinSequence(id=None, seq='mkgyfgpyggqyvpeilmga…'),
ProteinSequence(id=None, seq='mkgyfgpyggqyvpeilmga…'),
ProteinSequence(id=None, seq='mkgyfgpyggqyvpeilmga…'),
ProteinSequence(id=None, seq='mkgyfgpyggqyvpeilmga…'),
ProteinSequence(id=None, seq='mkgyfgpyggqyvpeilmga…'),
ProteinSequence(id=None, seq='mkgyfgpyggqyvpeilmga…'),
ProteinSequence(id=None, seq='mkgyfgpyggqyvpeilmga…'),
ProteinSequence(id=None, seq='mkgyfgpyggqyvpeilmga…'),
ProteinSequence(id=None, seq='mkgyfgpyggqyvpeilmga…'),
```

```
ProteinSequence(id=None, seq='mkgyfgpyggqyvpeilmga…'),
ProteinSequence(id=None, seq='mkgyfgpyggqyvpeilmga…'),
ProteinSequence(id=None, seq='mkgyfgpyggqyvpeilmga…'),
ProteinSequence(id=None, seq='mkgyfgpyggqyvpeilmga…'),
ProteinSequence(id=None, seq='mkgyfgpyggqyvpeilmga…'),
ProteinSequence(id=None, seq='mkgyfgpyggqyvpeilmga…'),
ProteinSequence(id=None, seq='mkgyfgpyggqyvpeilmga…'),
ProteinSequence(id=None, seq='mkgyfgpyggqyvpeilmga…'),
ProteinSequence(id=None, seq='mkgyfgpyggqyvpeilmga…'),
ProteinSequence(id=None, seq='mkgyfgpyggqyvpeilmga…'),
ProteinSequence(id=None, seq='mkgyfgpyggqyvpeilmga…'),
ProteinSequence(id=None, seq='mkgyfgpyggqyvpeilmga…'),
ProteinSequence(id=None, seq='mkgyfgpyggqyvpeilmga…'),
ProteinSequence(id=None, seq='mkgyfgpyggqyvpeilmga…'),
ProteinSequence(id=None, seq='mkgyfgpyggqyvpeilmga…'),
ProteinSequence(id=None, seq='mkgyfgpyggqyvpeilmga…'),
ProteinSequence(id=None, seq='mkgyfgpyggqyvpeilmga…'),
ProteinSequence(id=None, seq='mkgyfgpyggqyvpeilmga…'),
ProteinSequence(id=None, seq='mkgyfgpyggqyvpeilmga…'),
ProteinSequence(id=None, seq='mkgyfgpyggqyvpeilmga…'),
ProteinSequence(id=None, seq='mkgyfgpyggqyvpeilmga…'),
ProteinSequence(id=None, seq='mkgyfgpyggqyvpeilmga…'),
ProteinSequence(id=None, seq='mkgyfgpyggqyvpeilmga…'),
ProteinSequence(id=None, seq='mkgyfgpyggqyvpeilmga…'),
ProteinSequence(id=None, seq='mkgyfgpyggqyvpeilmga…'),
ProteinSequence(id=None, seq='mkgyfgpyggqyvpeilmga…'),
ProteinSequence(id=None, seq='mkgyfgpyggqyvpeilmga…'),
ProteinSequence(id=None, seq='mkgyfgpyggqyvpeilmga…'),
ProteinSequence(id=None, seq='mkgyfgpyggqyvpeilmga…'),
ProteinSequence(id=None, seq='mkgyfgpyggqyvpeilmga…'),
ProteinSequence(id=None, seq='mkgyfgpyggqyvpeilmga…'),
ProteinSequence(id=None, seq='mkgyfgpyggqyvpeilmga…'),
ProteinSequence(id=None, seq='mkgyfgpyggqyvpeilmga…'),
ProteinSequence(id=None, seq='mkgyfgpyggqyvpeilmga…'),
ProteinSequence(id=None, seq='mkgyfgpyggqyvpeilmga…'),
ProteinSequence(id=None, seq='mkgyfgpyggqyvpeilmga…'),
ProteinSequence(id=None, seq='mkgyfgpyggqyvpeilmga…'),
ProteinSequence(id=None, seq='mkgyfgpyggqyvpeilmga…'),
ProteinSequence(id=None, seq='mkgyfgpyggqyvpeilmga…'),
ProteinSequence(id=None, seq='mkgyfgpyggqyvpeilmga…'),
ProteinSequence(id=None, seq='mkgyfgpyggqyvpeilmga…'),
ProteinSequence(id=None, seq='mkgyfgpyggqyvpeilmga…'),
ProteinSequence(id=None, seq='mkgyfgpyggqyvpeilmga…'),
ProteinSequence(id=None, seq='mkgyfgpyggqyvpeilmga…'),
ProteinSequence(id=None, seq='mkgyfgpyggqyvpeilmga…'),
ProteinSequence(id=None, seq='mkgyfgpyggqyvpeilmga…'),
ProteinSequence(id=None, seq='mkgyfgpyggqyvpeilmga…'),
ProteinSequence(id=None, seq='mkgyfgpyggqyvpeilmga…'),
```

```
ProteinSequence(id=None, seq='mkgyfgpyggqyvpeilmga…'),
ProteinSequence(id=None, seq='mkgyfgpyggqyvpeilmga…'),
ProteinSequence(id=None, seq='mkgyfgpyggqyvpeilmga…'),
ProteinSequence(id=None, seq='mkgyfgpyggqyvpeilmga…'),
ProteinSequence(id=None, seq='mkgyfgpyggqyvpeilmga…'),
ProteinSequence(id=None, seq='mkgyfgpyggqyvpeilmga…'),
ProteinSequence(id=None, seq='mkgyfgpyggqyvpeilmga…'),
ProteinSequence(id=None, seq='mkgyfgpyggqyvpeilmga…'),
ProteinSequence(id=None, seq='mkgyfgpyggqyvpeilmga…'),
ProteinSequence(id=None, seq='mkgyfgpyggqyvpeilmga…'),
ProteinSequence(id=None, seq='mkgyfgpyggqyvpeilmga…'),
ProteinSequence(id=None, seq='mkgyfgpyggqyvpeilmga…'),
ProteinSequence(id=None, seq='mkgyfgpyggqyvpeilmga…'),
ProteinSequence(id=None, seq='mkgyfgpyggqyvpeilmga…'),
ProteinSequence(id=None, seq='mkgyfgpyggqyvpeilmga…'),
ProteinSequence(id=None, seq='mkgyfgpyggqyvpeilmga…'),
ProteinSequence(id=None, seq='mkgyfgpyggqyvpeilmga…'),
ProteinSequence(id=None, seq='mkgyfgpyggqyvpeilmga…'),
ProteinSequence(id=None, seq='mkgyfgpyggqyvpeilmga…'),
ProteinSequence(id=None, seq='mkgyfgpyggqyvpeilmga…'),
ProteinSequence(id=None, seq='mkgyfgpyggqyvpeilmga…'),
ProteinSequence(id=None, seq='mkgyfgpyggqyvpeilmga…'),
ProteinSequence(id=None, seq='mkgyfgpyggqyvpeilmga…'),
ProteinSequence(id=None, seq='mkgyfgpyggqyvpeilmga…'),
ProteinSequence(id=None, seq='mkgyfgpyggqyvpeilmga…'),
ProteinSequence(id=None, seq='mkgyfgpyggqyvpeilmga…'),
ProteinSequence(id=None, seq='mkgyfgpyggqyvpeilmga…'),
ProteinSequence(id=None, seq='mkgyfgpyggqyvpeilmga…'),
ProteinSequence(id=None, seq='mkgyfgpyggqyvpeilmga…'),
ProteinSequence(id=None, seq='mkgyfgpyggqyvpeilmga…'),
ProteinSequence(id=None, seq='mkgyfgpyggqyvpeilmga…'),
ProteinSequence(id=None, seq='mkgyfgpyggqyvpeilmga…'),
ProteinSequence(id=None, seq='mkgyfgpyggqyvpeilmga…'),
ProteinSequence(id=None, seq='mkgyfgpyggqyvpeilmga…'),
ProteinSequence(id=None, seq='mkgyfgpyggqyvpeilmga…'),
ProteinSequence(id=None, seq='mkgyfgpyggqyvpeilmga…'),
ProteinSequence(id=None, seq='mkgyfgpyggqyvpeilmga…'),
ProteinSequence(id=None, seq='mkgyfgpyggqyvpeilmga…'),
ProteinSequence(id=None, seq='mkgyfgpyggqyvpeilmga…'),
ProteinSequence(id=None, seq='mkgyfgpyggqyvpeilmga…'),
ProteinSequence(id=None, seq='mkgyfgpyggqyvpeilmga…'),
ProteinSequence(id=None, seq='mkgyfgpyggqyvpeilmga…'),
ProteinSequence(id=None, seq='mkgyfgpyggqyvpeilmga…'),
ProteinSequence(id=None, seq='mkgyfgpyggqyvpeilmga…'),
ProteinSequence(id=None, seq='mkgyfgpyggqyvpeilmga…'),
ProteinSequence(id=None, seq='mkgyfgpyggqyvpeilmga…'),
ProteinSequence(id=None, seq='mkgyfgpyggqyvpeilmga…'),
ProteinSequence(id=None, seq='mkgyfgpyggqyvpeilmga…'),
```

```
ProteinSequence(id=None, seq='mkgyfgpyggqyvpeilmga…'),
ProteinSequence(id=None, seq='mkgyfgpyggqyvpeilmga…'),
ProteinSequence(id=None, seq='mkgyfgpyggqyvpeilmga…'),
ProteinSequence(id=None, seq='mkgyfgpyggqyvpeilmga…'),
ProteinSequence(id=None, seq='mkgyfgpyggqyvpeilmga…'),
ProteinSequence(id=None, seq='mkgyfgpyggqyvpeilmga…'),
ProteinSequence(id=None, seq='mkgyfgpyggqyvpeilmga…'),
ProteinSequence(id=None, seq='mkgyfgpyggqyvpeilmga…'),
ProteinSequence(id=None, seq='mkgyfgpyggqyvpeilmga…'),
ProteinSequence(id=None, seq='mkgyfgpyggqyvpeilmga…'),
ProteinSequence(id=None, seq='mkgyfgpyggqyvpeilmga…'),
ProteinSequence(id=None, seq='mkgyfgpyggqyvpeilmga…'),
ProteinSequence(id=None, seq='mkgyfgpyggqyvpeilmga…'),
ProteinSequence(id=None, seq='mkgyfgpyggqyvpeilmga…'),
ProteinSequence(id=None, seq='mkgyfgpyggqyvpeilmga…'),
ProteinSequence(id=None, seq='mkgyfgpyggqyvpeilmga…'),
ProteinSequence(id=None, seq='mkgyfgpyggqyvpeilmga…'),
ProteinSequence(id=None, seq='mkgyfgpyggqyvpeilmga…'),
ProteinSequence(id=None, seq='mkgyfgpyggqyvpeilmga…'),
ProteinSequence(id=None, seq='mkgyfgpyggqyvpeilmga…'),
ProteinSequence(id=None, seq='mkgyfgpyggqyvpeilmga…'),
ProteinSequence(id=None, seq='mkgyfgpyggqyvpeilmga…'),
ProteinSequence(id=None, seq='mkgyfgpyggqyvpeilmga…'),
ProteinSequence(id=None, seq='mkgyfgpyggqyvpeilmga…'),
ProteinSequence(id=None, seq='mkgyfgpyggqyvpeilmga…'),
ProteinSequence(id=None, seq='mkgyfgpyggqyvpeilmga…'),
ProteinSequence(id=None, seq='mkgyfgpyggqyvpeilmga…'),
ProteinSequence(id=None, seq='mkgyfgpyggqyvpeilmga…'),
ProteinSequence(id=None, seq='mkgyfgpyggqyvpeilmga…'),
ProteinSequence(id=None, seq='mkgyfgpyggqyvpeilmga…'),
ProteinSequence(id=None, seq='mkgyfgpyggqyvpeilmga…'),
ProteinSequence(id=None, seq='mkgyfgpyggqyvpeilmga…'),
ProteinSequence(id=None, seq='mkgyfgpyggqyvpeilmga…'),
ProteinSequence(id=None, seq='mkgyfgpyggqyvpeilmga…'),
ProteinSequence(id=None, seq='mkgyfgpyggqyvpeilmga…'),
ProteinSequence(id=None, seq='mkgyfgpyggqyvpeilmga…'),
ProteinSequence(id=None, seq='mkgyfgpyggqyvpeilmga…'),
ProteinSequence(id=None, seq='mkgyfgpyggqyvpeilmga…'),
ProteinSequence(id=None, seq='mkgyfgpyggqyvpeilmga…'),
ProteinSequence(id=None, seq='mkgyfgpyggqyvpeilmga…'),
ProteinSequence(id=None, seq='mkgyfgpyggqyvpeilmga…'),
ProteinSequence(id=None, seq='mkgyfgpyggqyvpeilmga…'),
ProteinSequence(id=None, seq='mkgyfgpyggqyvpeilmga…'),
ProteinSequence(id=None, seq='mkgyfgpyggqyvpeilmga…'),
ProteinSequence(id=None, seq='mkgyfgpyggqyvpeilmga…'),
ProteinSequence(id=None, seq='mkgyfgpyggqyvpeilmga…'),
ProteinSequence(id=None, seq='mkgyfgpyggqyvpeilmga…'),
ProteinSequence(id=None, seq='mkgyfgpyggqyvpeilmga…'),
```

```
ProteinSequence(id=None, seq='mkgyfgpyggqyvpeilmga…'),
ProteinSequence(id=None, seq='mkgyfgpyggqyvpeilmga…'),
ProteinSequence(id=None, seq='mkgyfgpyggqyvpeilmga…'),
ProteinSequence(id=None, seq='mkgyfgpyggqyvpeilmga…'),
ProteinSequence(id=None, seq='mkgyfgpyggqyvpeilmga…'),
ProteinSequence(id=None, seq='mkgyfgpyggqyvpeilmga…'),
ProteinSequence(id=None, seq='mkgyfgpyggqyvpeilmga…'),
ProteinSequence(id=None, seq='mkgyfgpyggqyvpeilmga…'),
ProteinSequence(id=None, seq='mkgyfgpyggqyvpeilmga…'),
ProteinSequence(id=None, seq='mkgyfgpyggqyvpeilmga…'),
ProteinSequence(id=None, seq='mkgyfgpyggqyvpeilmga…'),
ProteinSequence(id=None, seq='mkgyfgpyggqyvpeilmga…'),
ProteinSequence(id=None, seq='mkgyfgpyggqyvpeilmga…'),
ProteinSequence(id=None, seq='mkgyfgpyggqyvpeilmga…'),
ProteinSequence(id=None, seq='mkgyfgpyggqyvpeilmga…'),
ProteinSequence(id=None, seq='mkgyfgpyggqyvpeilmga…'),
ProteinSequence(id=None, seq='mkgyfgpyggqyvpeilmga…'),
ProteinSequence(id=None, seq='mkgyfgpyggqyvpeilmga…'),
ProteinSequence(id=None, seq='mkgyfgpyggqyvpeilmga…'),
ProteinSequence(id=None, seq='mkgyfgpyggqyvpeilmga…'),
ProteinSequence(id=None, seq='mkgyfgpyggqyvpeilmga…'),
ProteinSequence(id=None, seq='mkgyfgpyggqyvpeilmga…'),
ProteinSequence(id=None, seq='mkgyfgpyggqyvpeilmga…'),
ProteinSequence(id=None, seq='mkgyfgpyggqyvpeilmga…'),
ProteinSequence(id=None, seq='mkgyfgpyggqyvpeilmga…'),
ProteinSequence(id=None, seq='mkgyfgpyggqyvpeilmga…'),
ProteinSequence(id=None, seq='mkgyfgpyggqyvpeilmga…'),
ProteinSequence(id=None, seq='mkgyfgpyggqyvpeilmga…'),
ProteinSequence(id=None, seq='mkgyfgpyggqyvpeilmga…'),
ProteinSequence(id=None, seq='mkgyfgpyggqyvpeilmga…'),
ProteinSequence(id=None, seq='mkgyfgpyggqyvpeilmga…'),
ProteinSequence(id=None, seq='mkgyfgpyggqyvpeilmga…'),
ProteinSequence(id=None, seq='mkgyfgpyggqyvpeilmga…'),
ProteinSequence(id=None, seq='mkgyfgpyggqyvpeilmga…'),
ProteinSequence(id=None, seq='mkgyfgpyggqyvpeilmga…'),
ProteinSequence(id=None, seq='mkgyfgpyggqyvpeilmga…'),
ProteinSequence(id=None, seq='mkgyfgpyggqyvpeilmga…'),
ProteinSequence(id=None, seq='mkgyfgpyggqyvpeilmga…'),
ProteinSequence(id=None, seq='mkgyfgpyggqyvpeilmga…'),
ProteinSequence(id=None, seq='mkgyfgpyggqyvpeilmga…'),
ProteinSequence(id=None, seq='mkgyfgpyggqyvpeilmga…'),
ProteinSequence(id=None, seq='mkgyfgpyggqyvpeilmga…'),
ProteinSequence(id=None, seq='mkgyfgpyggqyvpeilmga…'),
ProteinSequence(id=None, seq='mkgyfgpyggqyvpeilmga…'),
ProteinSequence(id=None, seq='mkgyfgpyggqyvpeilmga…'),
ProteinSequence(id=None, seq='mkgyfgpyggqyvpeilmga…'),
ProteinSequence(id=None, seq='mkgyfgpyggqyvpeilmga…'),
ProteinSequence(id=None, seq='mkgyfgpyggqyvpeilmga…'),
```

```
ProteinSequence(id=None, seq='mkgyfgpyggqyvpeilmga…'),
ProteinSequence(id=None, seq='mkgyfgpyggqyvpeilmga…'),
ProteinSequence(id=None, seq='mkgyfgpyggqyvpeilmga…'),
ProteinSequence(id=None, seq='mkgyfgpyggqyvpeilmga…'),
ProteinSequence(id=None, seq='mkgyfgpyggqyvpeilmga…'),
ProteinSequence(id=None, seq='mkgyfgpyggqyvpeilmga…'),
ProteinSequence(id=None, seq='mkgyfgpyggqyvpeilmga…'),
ProteinSequence(id=None, seq='mkgyfgpyggqyvpeilmga…'),
ProteinSequence(id=None, seq='mkgyfgpyggqyvpeilmga…'),
ProteinSequence(id=None, seq='mkgyfgpyggqyvpeilmga…'),
ProteinSequence(id=None, seq='mkgyfgpyggqyvpeilmga…'),
ProteinSequence(id=None, seq='mkgyfgpyggqyvpeilmga…'),
ProteinSequence(id=None, seq='mkgyfgpyggqyvpeilmga…'),
ProteinSequence(id=None, seq='mkgyfgpyggqyvpeilmga…'),
ProteinSequence(id=None, seq='mkgyfgpyggqyvpeilmga…'),
ProteinSequence(id=None, seq='mkgyfgpyggqyvpeilmga…'),
ProteinSequence(id=None, seq='mkgyfgpyggqyvpeilmga…'),
ProteinSequence(id=None, seq='mkgyfgpyggqyvpeilmga…'),
ProteinSequence(id=None, seq='mkgyfgpyggqyvpeilmga…'),
ProteinSequence(id=None, seq='mkgyfgpyggqyvpeilmga…'),
ProteinSequence(id=None, seq='mkgyfgpyggqyvpeilmga…'),
ProteinSequence(id=None, seq='mkgyfgpyggqyvpeilmga…'),
ProteinSequence(id=None, seq='mkgyfgpyggqyvpeilmga…'),
ProteinSequence(id=None, seq='mkgyfgpyggqyvpeilmga…'),
ProteinSequence(id=None, seq='mkgyfgpyggqyvpeilmga…'),
ProteinSequence(id=None, seq='mkgyfgpyggqyvpeilmga…'),
ProteinSequence(id=None, seq='mkgyfgpyggqyvpeilmga…'),
ProteinSequence(id=None, seq='mkgyfgpyggqyvpeilmga…'),
ProteinSequence(id=None, seq='mkgyfgpyggqyvpeilmga…'),
ProteinSequence(id=None, seq='mkgyfgpyggqyvpeilmga…'),
ProteinSequence(id=None, seq='mkgyfgpyggqyvpeilmga…'),
ProteinSequence(id=None, seq='mkgyfgpyggqyvpeilmga…'),
ProteinSequence(id=None, seq='mkgyfgpyggqyvpeilmga…'),
ProteinSequence(id=None, seq='mkgyfgpyggqyvpeilmga…'),
ProteinSequence(id=None, seq='mkgyfgpyggqyvpeilmga…'),
ProteinSequence(id=None, seq='mkgyfgpyggqyvpeilmga…'),
ProteinSequence(id=None, seq='mkgyfgpyggqyvpeilmga…'),
ProteinSequence(id=None, seq='mkgyfgpyggqyvpeilmga…'),
ProteinSequence(id=None, seq='mkgyfgpyggqyvpeilmga…'),
ProteinSequence(id=None, seq='mkgyfgpyggqyvpeilmga…'),
ProteinSequence(id=None, seq='mkgyfgpyggqyvpeilmga…'),
ProteinSequence(id=None, seq='mkgyfgpyggqyvpeilmga…'),
ProteinSequence(id=None, seq='mkgyfgpyggqyvpeilmga…'),
ProteinSequence(id=None, seq='mkgyfgpyggqyvpeilmga…'),
ProteinSequence(id=None, seq='mkgyfgpyggqyvpeilmga…'),
ProteinSequence(id=None, seq='mkgyfgpyggqyvpeilmga…'),
ProteinSequence(id=None, seq='mkgyfgpyggqyvpeilmga…'),
ProteinSequence(id=None, seq='mkgyfgpyggqyvpeilmga…'),
```

```
ProteinSequence(id=None, seq='mkgyfgpyggqyvpeilmga…'),
ProteinSequence(id=None, seq='mkgyfgpyggqyvpeilmga…')]
  warnings.warn(f"This model expects no fit, but received input. Ignoring input:
{X}")
INFO:aide_predict.bespoke_models.base:Transforming input using
ESM2LikelihoodWrapper
INFO:aide_predict.bespoke_models.base:Found 0 cached results, running on 127303
uncached sequences.
Computing log likelihoods: 100%|      | 1/1 [00:05<00:00,  5.01s/sequence]
INFO:aide_predict.bespoke_models.base:Transforming input using
ESM2LikelihoodWrapper
INFO:aide_predict.bespoke_models.base:Found 95477 cached results, running on
31826 uncached sequences.
Computing log likelihoods: 100%|      | 1/1 [00:00<00:00, 34.71sequence/s]
INFO:aide_predict.bespoke_models.base:Transforming input using
ESM2LikelihoodWrapper
INFO:aide_predict.bespoke_models.base:Found 127303 cached results, running on 0
uncached sequences.
INFO:aide_predict.bespoke_models.base:Transforming input using
ESM2LikelihoodWrapper
INFO:aide_predict.bespoke_models.base:Found 127303 cached results, running on 0
uncached sequences.
INFO:aide_predict.bespoke_models.base:Transforming input using
ESM2LikelihoodWrapper
INFO:aide_predict.bespoke_models.base:Found 127304 cached results, running on 0
uncached sequences.
```

[36]:
```
joblib.dump(esm_none_none_scores, 'esm_none_none_scores.joblib')
```

[36]:
```
['esm_none_none_scores.joblib']
```

1.4.2 2.2 EVC

[16]:
```
evc = ap.EVMutationWrapper(
    wt=wt,
    metadata_folder=os.path.join('.', 'data', 'epistatic', 'evmutation'),
)
evc.fit()
```

```
INFO:aide_predict.bespoke_models.base:Fitting EVMutationWrapper
```

[16]:
```
EVMutationWrapper(metadata_folder='./data/epistatic/evmutation',
                  wt=ProteinSequence(id='TARGET/1-397',
seq='mkgyfgpyggqyvpeilmga…'))
```

[43]:
```
evc_none_none_scores = cv_1(evc, X, y)
```

```
WARNING:aide_predict.bespoke_models.base:Model is already fitted. Skipping
```

```
INFO:aide_predict.bespoke_models.base:Transforming input using EVMutationWrapper
  0%|          | 0/127303 [00:00<?, ?it/s]/kfs2/projects/proteinml/repos/aide_pr
edict/aide_predict/bespoke_models/predictors/evmutation.py:186: UserWarning:
Mutation M1m is not supported by EVCouplings statistics and will be ignored for
scoring.
  warnings.warn(f"Mutation {subs_from}{subs_pos}{subs_to} is not supported by
EVCouplings statistics and will be ignored for scoring.")
/kfs2/projects/proteinml/repos/aide_predict/aide_predict/bespoke_models/predicto
rs/evmutation.py:186: UserWarning: Mutation K2k is not supported by EVCouplings
statistics and will be ignored for scoring.
  warnings.warn(f"Mutation {subs_from}{subs_pos}{subs_to} is not supported by
EVCouplings statistics and will be ignored for scoring.")
/kfs2/projects/proteinml/repos/aide_predict/aide_predict/bespoke_models/predicto
rs/evmutation.py:186: UserWarning: Mutation G3g is not supported by EVCouplings
statistics and will be ignored for scoring.
  warnings.warn(f"Mutation {subs_from}{subs_pos}{subs_to} is not supported by
EVCouplings statistics and will be ignored for scoring.")
/kfs2/projects/proteinml/repos/aide_predict/aide_predict/bespoke_models/predicto
rs/evmutation.py:186: UserWarning: Mutation Y4y is not supported by EVCouplings
statistics and will be ignored for scoring.
  warnings.warn(f"Mutation {subs_from}{subs_pos}{subs_to} is not supported by
EVCouplings statistics and will be ignored for scoring.")
/kfs2/projects/proteinml/repos/aide_predict/aide_predict/bespoke_models/predicto
rs/evmutation.py:186: UserWarning: Mutation F5f is not supported by EVCouplings
statistics and will be ignored for scoring.
  warnings.warn(f"Mutation {subs_from}{subs_pos}{subs_to} is not supported by
EVCouplings statistics and will be ignored for scoring.")
/kfs2/projects/proteinml/repos/aide_predict/aide_predict/bespoke_models/predicto
rs/evmutation.py:186: UserWarning: Mutation G6g is not supported by EVCouplings
statistics and will be ignored for scoring.
  warnings.warn(f"Mutation {subs_from}{subs_pos}{subs_to} is not supported by
EVCouplings statistics and will be ignored for scoring.")
/kfs2/projects/proteinml/repos/aide_predict/aide_predict/bespoke_models/predicto
rs/evmutation.py:186: UserWarning: Mutation P7p is not supported by EVCouplings
statistics and will be ignored for scoring.
  warnings.warn(f"Mutation {subs_from}{subs_pos}{subs_to} is not supported by
EVCouplings statistics and will be ignored for scoring.")
/kfs2/projects/proteinml/repos/aide_predict/aide_predict/bespoke_models/predicto
rs/evmutation.py:186: UserWarning: Mutation Y8y is not supported by EVCouplings
statistics and will be ignored for scoring.
  warnings.warn(f"Mutation {subs_from}{subs_pos}{subs_to} is not supported by
EVCouplings statistics and will be ignored for scoring.")
/kfs2/projects/proteinml/repos/aide_predict/aide_predict/bespoke_models/predicto
rs/evmutation.py:186: UserWarning: Mutation G9g is not supported by EVCouplings
statistics and will be ignored for scoring.
  warnings.warn(f"Mutation {subs_from}{subs_pos}{subs_to} is not supported by
EVCouplings statistics and will be ignored for scoring.")
/kfs2/projects/proteinml/repos/aide_predict/aide_predict/bespoke_models/predicto
```

```
rs/evmutation.py:186: UserWarning: Mutation G10g is not supported by EVCouplings
statistics and will be ignored for scoring.
  warnings.warn(f"Mutation {subs_from}{subs_pos}{subs_to} is not supported by
EVCouplings statistics and will be ignored for scoring.")
/kfs2/projects/proteinml/repos/aide_predict/aide_predict/bespoke_models/predicto
rs/evmutation.py:186: UserWarning: Mutation Q11q is not supported by EVCouplings
statistics and will be ignored for scoring.
  warnings.warn(f"Mutation {subs_from}{subs_pos}{subs_to} is not supported by
EVCouplings statistics and will be ignored for scoring.")
/kfs2/projects/proteinml/repos/aide_predict/aide_predict/bespoke_models/predicto
rs/evmutation.py:186: UserWarning: Mutation Y12y is not supported by EVCouplings
statistics and will be ignored for scoring.
  warnings.warn(f"Mutation {subs_from}{subs_pos}{subs_to} is not supported by
EVCouplings statistics and will be ignored for scoring.")
/kfs2/projects/proteinml/repos/aide_predict/aide_predict/bespoke_models/predicto
rs/evmutation.py:186: UserWarning: Mutation V13v is not supported by EVCouplings
statistics and will be ignored for scoring.
  warnings.warn(f"Mutation {subs_from}{subs_pos}{subs_to} is not supported by
EVCouplings statistics and will be ignored for scoring.")
/kfs2/projects/proteinml/repos/aide_predict/aide_predict/bespoke_models/predicto
rs/evmutation.py:186: UserWarning: Mutation P14p is not supported by EVCouplings
statistics and will be ignored for scoring.
  warnings.warn(f"Mutation {subs_from}{subs_pos}{subs_to} is not supported by
EVCouplings statistics and will be ignored for scoring.")
/kfs2/projects/proteinml/repos/aide_predict/aide_predict/bespoke_models/predicto
rs/evmutation.py:186: UserWarning: Mutation E15e is not supported by EVCouplings
statistics and will be ignored for scoring.
  warnings.warn(f"Mutation {subs_from}{subs_pos}{subs_to} is not supported by
EVCouplings statistics and will be ignored for scoring.")
/kfs2/projects/proteinml/repos/aide_predict/aide_predict/bespoke_models/predicto
rs/evmutation.py:186: UserWarning: Mutation I16i is not supported by EVCouplings
statistics and will be ignored for scoring.
  warnings.warn(f"Mutation {subs_from}{subs_pos}{subs_to} is not supported by
EVCouplings statistics and will be ignored for scoring.")
/kfs2/projects/proteinml/repos/aide_predict/aide_predict/bespoke_models/predicto
rs/evmutation.py:186: UserWarning: Mutation L17l is not supported by EVCouplings
statistics and will be ignored for scoring.
  warnings.warn(f"Mutation {subs_from}{subs_pos}{subs_to} is not supported by
EVCouplings statistics and will be ignored for scoring.")
/kfs2/projects/proteinml/repos/aide_predict/aide_predict/bespoke_models/predicto
rs/evmutation.py:186: UserWarning: Mutation M18m is not supported by EVCouplings
statistics and will be ignored for scoring.
  warnings.warn(f"Mutation {subs_from}{subs_pos}{subs_to} is not supported by
EVCouplings statistics and will be ignored for scoring.")
/kfs2/projects/proteinml/repos/aide_predict/aide_predict/bespoke_models/predicto
rs/evmutation.py:186: UserWarning: Mutation G19g is not supported by EVCouplings
statistics and will be ignored for scoring.
  warnings.warn(f"Mutation {subs_from}{subs_pos}{subs_to} is not supported by
```

```
EVCouplings statistics and will be ignored for scoring.")
/kfs2/projects/proteinml/repos/aide_predict/aide_predict/bespoke_models/predicto
rs/evmutation.py:186: UserWarning: Mutation A20a is not supported by EVCouplings
statistics and will be ignored for scoring.
  warnings.warn(f"Mutation {subs_from}{subs_pos}{subs_to} is not supported by
EVCouplings statistics and will be ignored for scoring.")
/kfs2/projects/proteinml/repos/aide_predict/aide_predict/bespoke_models/predicto
rs/evmutation.py:186: UserWarning: Mutation L21l is not supported by EVCouplings
statistics and will be ignored for scoring.
  warnings.warn(f"Mutation {subs_from}{subs_pos}{subs_to} is not supported by
EVCouplings statistics and will be ignored for scoring.")
/kfs2/projects/proteinml/repos/aide_predict/aide_predict/bespoke_models/predicto
rs/evmutation.py:186: UserWarning: Mutation E22e is not supported by EVCouplings
statistics and will be ignored for scoring.
  warnings.warn(f"Mutation {subs_from}{subs_pos}{subs_to} is not supported by
EVCouplings statistics and will be ignored for scoring.")
/kfs2/projects/proteinml/repos/aide_predict/aide_predict/bespoke_models/predicto
rs/evmutation.py:186: UserWarning: Mutation E23e is not supported by EVCouplings
statistics and will be ignored for scoring.
  warnings.warn(f"Mutation {subs_from}{subs_pos}{subs_to} is not supported by
EVCouplings statistics and will be ignored for scoring.")
/kfs2/projects/proteinml/repos/aide_predict/aide_predict/bespoke_models/predicto
rs/evmutation.py:186: UserWarning: Mutation L24l is not supported by EVCouplings
statistics and will be ignored for scoring.
  warnings.warn(f"Mutation {subs_from}{subs_pos}{subs_to} is not supported by
EVCouplings statistics and will be ignored for scoring.")
/kfs2/projects/proteinml/repos/aide_predict/aide_predict/bespoke_models/predicto
rs/evmutation.py:186: UserWarning: Mutation E25e is not supported by EVCouplings
statistics and will be ignored for scoring.
  warnings.warn(f"Mutation {subs_from}{subs_pos}{subs_to} is not supported by
EVCouplings statistics and will be ignored for scoring.")
/kfs2/projects/proteinml/repos/aide_predict/aide_predict/bespoke_models/predicto
rs/evmutation.py:186: UserWarning: Mutation A26a is not supported by EVCouplings
statistics and will be ignored for scoring.
  warnings.warn(f"Mutation {subs_from}{subs_pos}{subs_to} is not supported by
EVCouplings statistics and will be ignored for scoring.")
/kfs2/projects/proteinml/repos/aide_predict/aide_predict/bespoke_models/predicto
rs/evmutation.py:186: UserWarning: Mutation A27a is not supported by EVCouplings
statistics and will be ignored for scoring.
  warnings.warn(f"Mutation {subs_from}{subs_pos}{subs_to} is not supported by
EVCouplings statistics and will be ignored for scoring.")
/kfs2/projects/proteinml/repos/aide_predict/aide_predict/bespoke_models/predicto
rs/evmutation.py:186: UserWarning: Mutation Y28y is not supported by EVCouplings
statistics and will be ignored for scoring.
  warnings.warn(f"Mutation {subs_from}{subs_pos}{subs_to} is not supported by
EVCouplings statistics and will be ignored for scoring.")
/kfs2/projects/proteinml/repos/aide_predict/aide_predict/bespoke_models/predicto
rs/evmutation.py:186: UserWarning: Mutation E29e is not supported by EVCouplings
```

```
statistics and will be ignored for scoring.
  warnings.warn(f"Mutation {subs_from}{subs_pos}{subs_to} is not supported by
EVCouplings statistics and will be ignored for scoring.")
/kfs2/projects/proteinml/repos/aide_predict/aide_predict/bespoke_models/predicto
rs/evmutation.py:186: UserWarning: Mutation G30g is not supported by EVCouplings
statistics and will be ignored for scoring.
  warnings.warn(f"Mutation {subs_from}{subs_pos}{subs_to} is not supported by
EVCouplings statistics and will be ignored for scoring.")
/kfs2/projects/proteinml/repos/aide_predict/aide_predict/bespoke_models/predicto
rs/evmutation.py:186: UserWarning: Mutation I31i is not supported by EVCouplings
statistics and will be ignored for scoring.
  warnings.warn(f"Mutation {subs_from}{subs_pos}{subs_to} is not supported by
EVCouplings statistics and will be ignored for scoring.")
/kfs2/projects/proteinml/repos/aide_predict/aide_predict/bespoke_models/predicto
rs/evmutation.py:186: UserWarning: Mutation M32m is not supported by EVCouplings
statistics and will be ignored for scoring.
  warnings.warn(f"Mutation {subs_from}{subs_pos}{subs_to} is not supported by
EVCouplings statistics and will be ignored for scoring.")
/kfs2/projects/proteinml/repos/aide_predict/aide_predict/bespoke_models/predicto
rs/evmutation.py:186: UserWarning: Mutation K33k is not supported by EVCouplings
statistics and will be ignored for scoring.
  warnings.warn(f"Mutation {subs_from}{subs_pos}{subs_to} is not supported by
EVCouplings statistics and will be ignored for scoring.")
/kfs2/projects/proteinml/repos/aide_predict/aide_predict/bespoke_models/predicto
rs/evmutation.py:186: UserWarning: Mutation D34d is not supported by EVCouplings
statistics and will be ignored for scoring.
  warnings.warn(f"Mutation {subs_from}{subs_pos}{subs_to} is not supported by
EVCouplings statistics and will be ignored for scoring.")
/kfs2/projects/proteinml/repos/aide_predict/aide_predict/bespoke_models/predicto
rs/evmutation.py:186: UserWarning: Mutation E35e is not supported by EVCouplings
statistics and will be ignored for scoring.
  warnings.warn(f"Mutation {subs_from}{subs_pos}{subs_to} is not supported by
EVCouplings statistics and will be ignored for scoring.")
/kfs2/projects/proteinml/repos/aide_predict/aide_predict/bespoke_models/predicto
rs/evmutation.py:186: UserWarning: Mutation S36s is not supported by EVCouplings
statistics and will be ignored for scoring.
  warnings.warn(f"Mutation {subs_from}{subs_pos}{subs_to} is not supported by
EVCouplings statistics and will be ignored for scoring.")
/kfs2/projects/proteinml/repos/aide_predict/aide_predict/bespoke_models/predicto
rs/evmutation.py:186: UserWarning: Mutation F37f is not supported by EVCouplings
statistics and will be ignored for scoring.
  warnings.warn(f"Mutation {subs_from}{subs_pos}{subs_to} is not supported by
EVCouplings statistics and will be ignored for scoring.")
/kfs2/projects/proteinml/repos/aide_predict/aide_predict/bespoke_models/predicto
rs/evmutation.py:186: UserWarning: Mutation W38w is not supported by EVCouplings
statistics and will be ignored for scoring.
  warnings.warn(f"Mutation {subs_from}{subs_pos}{subs_to} is not supported by
EVCouplings statistics and will be ignored for scoring.")
```

```
/kfs2/projects/proteinml/repos/aide_predict/aide_predict/bespoke_models/predicto
rs/evmutation.py:186: UserWarning: Mutation K39k is not supported by EVCouplings
statistics and will be ignored for scoring.
  warnings.warn(f"Mutation {subs_from}{subs_pos}{subs_to} is not supported by
EVCouplings statistics and will be ignored for scoring.")
/kfs2/projects/proteinml/repos/aide_predict/aide_predict/bespoke_models/predicto
rs/evmutation.py:186: UserWarning: Mutation E40e is not supported by EVCouplings
statistics and will be ignored for scoring.
  warnings.warn(f"Mutation {subs_from}{subs_pos}{subs_to} is not supported by
EVCouplings statistics and will be ignored for scoring.")
/kfs2/projects/proteinml/repos/aide_predict/aide_predict/bespoke_models/predicto
rs/evmutation.py:186: UserWarning: Mutation F41f is not supported by EVCouplings
statistics and will be ignored for scoring.
  warnings.warn(f"Mutation {subs_from}{subs_pos}{subs_to} is not supported by
EVCouplings statistics and will be ignored for scoring.")
/kfs2/projects/proteinml/repos/aide_predict/aide_predict/bespoke_models/predicto
rs/evmutation.py:186: UserWarning: Mutation N42n is not supported by EVCouplings
statistics and will be ignored for scoring.
  warnings.warn(f"Mutation {subs_from}{subs_pos}{subs_to} is not supported by
EVCouplings statistics and will be ignored for scoring.")
/kfs2/projects/proteinml/repos/aide_predict/aide_predict/bespoke_models/predicto
rs/evmutation.py:186: UserWarning: Mutation D43d is not supported by EVCouplings
statistics and will be ignored for scoring.
  warnings.warn(f"Mutation {subs_from}{subs_pos}{subs_to} is not supported by
EVCouplings statistics and will be ignored for scoring.")
/kfs2/projects/proteinml/repos/aide_predict/aide_predict/bespoke_models/predicto
rs/evmutation.py:186: UserWarning: Mutation L44l is not supported by EVCouplings
statistics and will be ignored for scoring.
  warnings.warn(f"Mutation {subs_from}{subs_pos}{subs_to} is not supported by
EVCouplings statistics and will be ignored for scoring.")
/kfs2/projects/proteinml/repos/aide_predict/aide_predict/bespoke_models/predicto
rs/evmutation.py:186: UserWarning: Mutation L45l is not supported by EVCouplings
statistics and will be ignored for scoring.
  warnings.warn(f"Mutation {subs_from}{subs_pos}{subs_to} is not supported by
EVCouplings statistics and will be ignored for scoring.")
/kfs2/projects/proteinml/repos/aide_predict/aide_predict/bespoke_models/predicto
rs/evmutation.py:186: UserWarning: Mutation R46r is not supported by EVCouplings
statistics and will be ignored for scoring.
  warnings.warn(f"Mutation {subs_from}{subs_pos}{subs_to} is not supported by
EVCouplings statistics and will be ignored for scoring.")
/kfs2/projects/proteinml/repos/aide_predict/aide_predict/bespoke_models/predicto
rs/evmutation.py:186: UserWarning: Mutation D47d is not supported by EVCouplings
statistics and will be ignored for scoring.
  warnings.warn(f"Mutation {subs_from}{subs_pos}{subs_to} is not supported by
EVCouplings statistics and will be ignored for scoring.")
/kfs2/projects/proteinml/repos/aide_predict/aide_predict/bespoke_models/predicto
rs/evmutation.py:186: UserWarning: Mutation Y48y is not supported by EVCouplings
statistics and will be ignored for scoring.
```

```
  warnings.warn(f"Mutation {subs_from}{subs_pos}{subs_to} is not supported by
EVCouplings statistics and will be ignored for scoring.")
/kfs2/projects/proteinml/repos/aide_predict/aide_predict/bespoke_models/predicto
rs/evmutation.py:186: UserWarning: Mutation D134d is not supported by
EVCouplings statistics and will be ignored for scoring.
  warnings.warn(f"Mutation {subs_from}{subs_pos}{subs_to} is not supported by
EVCouplings statistics and will be ignored for scoring.")
/kfs2/projects/proteinml/repos/aide_predict/aide_predict/bespoke_models/predicto
rs/evmutation.py:186: UserWarning: Mutation I136i is not supported by
EVCouplings statistics and will be ignored for scoring.
  warnings.warn(f"Mutation {subs_from}{subs_pos}{subs_to} is not supported by
EVCouplings statistics and will be ignored for scoring.")
/kfs2/projects/proteinml/repos/aide_predict/aide_predict/bespoke_models/predicto
rs/evmutation.py:186: UserWarning: Mutation R137r is not supported by
EVCouplings statistics and will be ignored for scoring.
  warnings.warn(f"Mutation {subs_from}{subs_pos}{subs_to} is not supported by
EVCouplings statistics and will be ignored for scoring.")
/kfs2/projects/proteinml/repos/aide_predict/aide_predict/bespoke_models/predicto
rs/evmutation.py:186: UserWarning: Mutation Q138q is not supported by
EVCouplings statistics and will be ignored for scoring.
  warnings.warn(f"Mutation {subs_from}{subs_pos}{subs_to} is not supported by
EVCouplings statistics and will be ignored for scoring.")
/kfs2/projects/proteinml/repos/aide_predict/aide_predict/bespoke_models/predicto
rs/evmutation.py:186: UserWarning: Mutation S157s is not supported by
EVCouplings statistics and will be ignored for scoring.
  warnings.warn(f"Mutation {subs_from}{subs_pos}{subs_to} is not supported by
EVCouplings statistics and will be ignored for scoring.")
/kfs2/projects/proteinml/repos/aide_predict/aide_predict/bespoke_models/predicto
rs/evmutation.py:186: UserWarning: Mutation G158g is not supported by
EVCouplings statistics and will be ignored for scoring.
  warnings.warn(f"Mutation {subs_from}{subs_pos}{subs_to} is not supported by
EVCouplings statistics and will be ignored for scoring.")
/kfs2/projects/proteinml/repos/aide_predict/aide_predict/bespoke_models/predicto
rs/evmutation.py:186: UserWarning: Mutation S159s is not supported by
EVCouplings statistics and will be ignored for scoring.
  warnings.warn(f"Mutation {subs_from}{subs_pos}{subs_to} is not supported by
EVCouplings statistics and will be ignored for scoring.")
/kfs2/projects/proteinml/repos/aide_predict/aide_predict/bespoke_models/predicto
rs/evmutation.py:186: UserWarning: Mutation L177l is not supported by
EVCouplings statistics and will be ignored for scoring.
  warnings.warn(f"Mutation {subs_from}{subs_pos}{subs_to} is not supported by
EVCouplings statistics and will be ignored for scoring.")
/kfs2/projects/proteinml/repos/aide_predict/aide_predict/bespoke_models/predicto
rs/evmutation.py:186: UserWarning: Mutation T179t is not supported by
EVCouplings statistics and will be ignored for scoring.
  warnings.warn(f"Mutation {subs_from}{subs_pos}{subs_to} is not supported by
EVCouplings statistics and will be ignored for scoring.")
/kfs2/projects/proteinml/repos/aide_predict/aide_predict/bespoke_models/predicto
```

```
rs/evmutation.py:186: UserWarning: Mutation T180t is not supported by
EVCouplings statistics and will be ignored for scoring.
  warnings.warn(f"Mutation {subs_from}{subs_pos}{subs_to} is not supported by
EVCouplings statistics and will be ignored for scoring.")
/kfs2/projects/proteinml/repos/aide_predict/aide_predict/bespoke_models/predicto
rs/evmutation.py:186: UserWarning: Mutation Y181y is not supported by
EVCouplings statistics and will be ignored for scoring.
  warnings.warn(f"Mutation {subs_from}{subs_pos}{subs_to} is not supported by
EVCouplings statistics and will be ignored for scoring.")
/kfs2/projects/proteinml/repos/aide_predict/aide_predict/bespoke_models/predicto
rs/evmutation.py:186: UserWarning: Mutation V183F is not supported by
EVCouplings statistics and will be ignored for scoring.
  warnings.warn(f"Mutation {subs_from}{subs_pos}{subs_to} is not supported by
EVCouplings statistics and will be ignored for scoring.")
/kfs2/projects/proteinml/repos/aide_predict/aide_predict/bespoke_models/predicto
rs/evmutation.py:186: UserWarning: Mutation P192p is not supported by
EVCouplings statistics and will be ignored for scoring.
  warnings.warn(f"Mutation {subs_from}{subs_pos}{subs_to} is not supported by
EVCouplings statistics and will be ignored for scoring.")
/kfs2/projects/proteinml/repos/aide_predict/aide_predict/bespoke_models/predicto
rs/evmutation.py:186: UserWarning: Mutation I196i is not supported by
EVCouplings statistics and will be ignored for scoring.
  warnings.warn(f"Mutation {subs_from}{subs_pos}{subs_to} is not supported by
EVCouplings statistics and will be ignored for scoring.")
/kfs2/projects/proteinml/repos/aide_predict/aide_predict/bespoke_models/predicto
rs/evmutation.py:186: UserWarning: Mutation V197v is not supported by
EVCouplings statistics and will be ignored for scoring.
  warnings.warn(f"Mutation {subs_from}{subs_pos}{subs_to} is not supported by
EVCouplings statistics and will be ignored for scoring.")
/kfs2/projects/proteinml/repos/aide_predict/aide_predict/bespoke_models/predicto
rs/evmutation.py:186: UserWarning: Mutation P213p is not supported by
EVCouplings statistics and will be ignored for scoring.
  warnings.warn(f"Mutation {subs_from}{subs_pos}{subs_to} is not supported by
EVCouplings statistics and will be ignored for scoring.")
/kfs2/projects/proteinml/repos/aide_predict/aide_predict/bespoke_models/predicto
rs/evmutation.py:186: UserWarning: Mutation E214e is not supported by
EVCouplings statistics and will be ignored for scoring.
  warnings.warn(f"Mutation {subs_from}{subs_pos}{subs_to} is not supported by
EVCouplings statistics and will be ignored for scoring.")
/kfs2/projects/proteinml/repos/aide_predict/aide_predict/bespoke_models/predicto
rs/evmutation.py:186: UserWarning: Mutation K215k is not supported by
EVCouplings statistics and will be ignored for scoring.
  warnings.warn(f"Mutation {subs_from}{subs_pos}{subs_to} is not supported by
EVCouplings statistics and will be ignored for scoring.")
/kfs2/projects/proteinml/repos/aide_predict/aide_predict/bespoke_models/predicto
rs/evmutation.py:186: UserWarning: Mutation E216e is not supported by
EVCouplings statistics and will be ignored for scoring.
  warnings.warn(f"Mutation {subs_from}{subs_pos}{subs_to} is not supported by
```

```
EVCouplings statistics and will be ignored for scoring.")
/kfs2/projects/proteinml/repos/aide_predict/aide_predict/bespoke_models/predicto
rs/evmutation.py:186: UserWarning: Mutation G217g is not supported by
EVCouplings statistics and will be ignored for scoring.
  warnings.warn(f"Mutation {subs_from}{subs_pos}{subs_to} is not supported by
EVCouplings statistics and will be ignored for scoring.")
/kfs2/projects/proteinml/repos/aide_predict/aide_predict/bespoke_models/predicto
rs/evmutation.py:186: UserWarning: Mutation G260g is not supported by
EVCouplings statistics and will be ignored for scoring.
  warnings.warn(f"Mutation {subs_from}{subs_pos}{subs_to} is not supported by
EVCouplings statistics and will be ignored for scoring.")
/kfs2/projects/proteinml/repos/aide_predict/aide_predict/bespoke_models/predicto
rs/evmutation.py:186: UserWarning: Mutation K261k is not supported by
EVCouplings statistics and will be ignored for scoring.
  warnings.warn(f"Mutation {subs_from}{subs_pos}{subs_to} is not supported by
EVCouplings statistics and will be ignored for scoring.")
/kfs2/projects/proteinml/repos/aide_predict/aide_predict/bespoke_models/predicto
rs/evmutation.py:186: UserWarning: Mutation H262h is not supported by
EVCouplings statistics and will be ignored for scoring.
  warnings.warn(f"Mutation {subs_from}{subs_pos}{subs_to} is not supported by
EVCouplings statistics and will be ignored for scoring.")
/kfs2/projects/proteinml/repos/aide_predict/aide_predict/bespoke_models/predicto
rs/evmutation.py:186: UserWarning: Mutation A263a is not supported by
EVCouplings statistics and will be ignored for scoring.
  warnings.warn(f"Mutation {subs_from}{subs_pos}{subs_to} is not supported by
EVCouplings statistics and will be ignored for scoring.")
/kfs2/projects/proteinml/repos/aide_predict/aide_predict/bespoke_models/predicto
rs/evmutation.py:186: UserWarning: Mutation A264a is not supported by
EVCouplings statistics and will be ignored for scoring.
  warnings.warn(f"Mutation {subs_from}{subs_pos}{subs_to} is not supported by
EVCouplings statistics and will be ignored for scoring.")
/kfs2/projects/proteinml/repos/aide_predict/aide_predict/bespoke_models/predicto
rs/evmutation.py:186: UserWarning: Mutation H275h is not supported by
EVCouplings statistics and will be ignored for scoring.
  warnings.warn(f"Mutation {subs_from}{subs_pos}{subs_to} is not supported by
EVCouplings statistics and will be ignored for scoring.")
/kfs2/projects/proteinml/repos/aide_predict/aide_predict/bespoke_models/predicto
rs/evmutation.py:186: UserWarning: Mutation G276g is not supported by
EVCouplings statistics and will be ignored for scoring.
  warnings.warn(f"Mutation {subs_from}{subs_pos}{subs_to} is not supported by
EVCouplings statistics and will be ignored for scoring.")
/kfs2/projects/proteinml/repos/aide_predict/aide_predict/bespoke_models/predicto
rs/evmutation.py:186: UserWarning: Mutation S277s is not supported by
EVCouplings statistics and will be ignored for scoring.
  warnings.warn(f"Mutation {subs_from}{subs_pos}{subs_to} is not supported by
EVCouplings statistics and will be ignored for scoring.")
/kfs2/projects/proteinml/repos/aide_predict/aide_predict/bespoke_models/predicto
rs/evmutation.py:186: UserWarning: Mutation K278k is not supported by
```

```
EVCouplings statistics and will be ignored for scoring.
  warnings.warn(f"Mutation {subs_from}{subs_pos}{subs_to} is not supported by
EVCouplings statistics and will be ignored for scoring.")
/kfs2/projects/proteinml/repos/aide_predict/aide_predict/bespoke_models/predicto
rs/evmutation.py:186: UserWarning: Mutation T279t is not supported by
EVCouplings statistics and will be ignored for scoring.
  warnings.warn(f"Mutation {subs_from}{subs_pos}{subs_to} is not supported by
EVCouplings statistics and will be ignored for scoring.")
/kfs2/projects/proteinml/repos/aide_predict/aide_predict/bespoke_models/predicto
rs/evmutation.py:186: UserWarning: Mutation F280f is not supported by
EVCouplings statistics and will be ignored for scoring.
  warnings.warn(f"Mutation {subs_from}{subs_pos}{subs_to} is not supported by
EVCouplings statistics and will be ignored for scoring.")
/kfs2/projects/proteinml/repos/aide_predict/aide_predict/bespoke_models/predicto
rs/evmutation.py:186: UserWarning: Mutation V281v is not supported by
EVCouplings statistics and will be ignored for scoring.
  warnings.warn(f"Mutation {subs_from}{subs_pos}{subs_to} is not supported by
EVCouplings statistics and will be ignored for scoring.")
/kfs2/projects/proteinml/repos/aide_predict/aide_predict/bespoke_models/predicto
rs/evmutation.py:186: UserWarning: Mutation L282l is not supported by
EVCouplings statistics and will be ignored for scoring.
  warnings.warn(f"Mutation {subs_from}{subs_pos}{subs_to} is not supported by
EVCouplings statistics and will be ignored for scoring.")
/kfs2/projects/proteinml/repos/aide_predict/aide_predict/bespoke_models/predicto
rs/evmutation.py:186: UserWarning: Mutation Q283q is not supported by
EVCouplings statistics and will be ignored for scoring.
  warnings.warn(f"Mutation {subs_from}{subs_pos}{subs_to} is not supported by
EVCouplings statistics and will be ignored for scoring.")
/kfs2/projects/proteinml/repos/aide_predict/aide_predict/bespoke_models/predicto
rs/evmutation.py:186: UserWarning: Mutation D284d is not supported by
EVCouplings statistics and will be ignored for scoring.
  warnings.warn(f"Mutation {subs_from}{subs_pos}{subs_to} is not supported by
EVCouplings statistics and will be ignored for scoring.")
/kfs2/projects/proteinml/repos/aide_predict/aide_predict/bespoke_models/predicto
rs/evmutation.py:186: UserWarning: Mutation D285d is not supported by
EVCouplings statistics and will be ignored for scoring.
  warnings.warn(f"Mutation {subs_from}{subs_pos}{subs_to} is not supported by
EVCouplings statistics and will be ignored for scoring.")
/kfs2/projects/proteinml/repos/aide_predict/aide_predict/bespoke_models/predicto
rs/evmutation.py:186: UserWarning: Mutation W286w is not supported by
EVCouplings statistics and will be ignored for scoring.
  warnings.warn(f"Mutation {subs_from}{subs_pos}{subs_to} is not supported by
EVCouplings statistics and will be ignored for scoring.")
/kfs2/projects/proteinml/repos/aide_predict/aide_predict/bespoke_models/predicto
rs/evmutation.py:186: UserWarning: Mutation G287g is not supported by
EVCouplings statistics and will be ignored for scoring.
  warnings.warn(f"Mutation {subs_from}{subs_pos}{subs_to} is not supported by
EVCouplings statistics and will be ignored for scoring.")
```

```
/kfs2/projects/proteinml/repos/aide_predict/aide_predict/bespoke_models/predicto
rs/evmutation.py:186: UserWarning: Mutation Q288q is not supported by
EVCouplings statistics and will be ignored for scoring.
  warnings.warn(f"Mutation {subs_from}{subs_pos}{subs_to} is not supported by
EVCouplings statistics and will be ignored for scoring.")
/kfs2/projects/proteinml/repos/aide_predict/aide_predict/bespoke_models/predicto
rs/evmutation.py:186: UserWarning: Mutation V289v is not supported by
EVCouplings statistics and will be ignored for scoring.
  warnings.warn(f"Mutation {subs_from}{subs_pos}{subs_to} is not supported by
EVCouplings statistics and will be ignored for scoring.")
/kfs2/projects/proteinml/repos/aide_predict/aide_predict/bespoke_models/predicto
rs/evmutation.py:186: UserWarning: Mutation Q290q is not supported by
EVCouplings statistics and will be ignored for scoring.
  warnings.warn(f"Mutation {subs_from}{subs_pos}{subs_to} is not supported by
EVCouplings statistics and will be ignored for scoring.")
/kfs2/projects/proteinml/repos/aide_predict/aide_predict/bespoke_models/predicto
rs/evmutation.py:186: UserWarning: Mutation V291v is not supported by
EVCouplings statistics and will be ignored for scoring.
  warnings.warn(f"Mutation {subs_from}{subs_pos}{subs_to} is not supported by
EVCouplings statistics and will be ignored for scoring.")
/kfs2/projects/proteinml/repos/aide_predict/aide_predict/bespoke_models/predicto
rs/evmutation.py:186: UserWarning: Mutation S292s is not supported by
EVCouplings statistics and will be ignored for scoring.
  warnings.warn(f"Mutation {subs_from}{subs_pos}{subs_to} is not supported by
EVCouplings statistics and will be ignored for scoring.")
/kfs2/projects/proteinml/repos/aide_predict/aide_predict/bespoke_models/predicto
rs/evmutation.py:186: UserWarning: Mutation V378v is not supported by
EVCouplings statistics and will be ignored for scoring.
  warnings.warn(f"Mutation {subs_from}{subs_pos}{subs_to} is not supported by
EVCouplings statistics and will be ignored for scoring.")
/kfs2/projects/proteinml/repos/aide_predict/aide_predict/bespoke_models/predicto
rs/evmutation.py:186: UserWarning: Mutation L379l is not supported by
EVCouplings statistics and will be ignored for scoring.
  warnings.warn(f"Mutation {subs_from}{subs_pos}{subs_to} is not supported by
EVCouplings statistics and will be ignored for scoring.")
/kfs2/projects/proteinml/repos/aide_predict/aide_predict/bespoke_models/predicto
rs/evmutation.py:186: UserWarning: Mutation N380n is not supported by
EVCouplings statistics and will be ignored for scoring.
  warnings.warn(f"Mutation {subs_from}{subs_pos}{subs_to} is not supported by
EVCouplings statistics and will be ignored for scoring.")
/kfs2/projects/proteinml/repos/aide_predict/aide_predict/bespoke_models/predicto
rs/evmutation.py:186: UserWarning: Mutation H381h is not supported by
EVCouplings statistics and will be ignored for scoring.
  warnings.warn(f"Mutation {subs_from}{subs_pos}{subs_to} is not supported by
EVCouplings statistics and will be ignored for scoring.")
/kfs2/projects/proteinml/repos/aide_predict/aide_predict/bespoke_models/predicto
rs/evmutation.py:186: UserWarning: Mutation P382p is not supported by
EVCouplings statistics and will be ignored for scoring.
```

```
  warnings.warn(f"Mutation {subs_from}{subs_pos}{subs_to} is not supported by
EVCouplings statistics and will be ignored for scoring.")
/kfs2/projects/proteinml/repos/aide_predict/aide_predict/bespoke_models/predicto
rs/evmutation.py:186: UserWarning: Mutation Y383y is not supported by
EVCouplings statistics and will be ignored for scoring.
  warnings.warn(f"Mutation {subs_from}{subs_pos}{subs_to} is not supported by
EVCouplings statistics and will be ignored for scoring.")
/kfs2/projects/proteinml/repos/aide_predict/aide_predict/bespoke_models/predicto
rs/evmutation.py:186: UserWarning: Mutation V384v is not supported by
EVCouplings statistics and will be ignored for scoring.
  warnings.warn(f"Mutation {subs_from}{subs_pos}{subs_to} is not supported by
EVCouplings statistics and will be ignored for scoring.")
/kfs2/projects/proteinml/repos/aide_predict/aide_predict/bespoke_models/predicto
rs/evmutation.py:186: UserWarning: Mutation R385r is not supported by
EVCouplings statistics and will be ignored for scoring.
  warnings.warn(f"Mutation {subs_from}{subs_pos}{subs_to} is not supported by
EVCouplings statistics and will be ignored for scoring.")
/kfs2/projects/proteinml/repos/aide_predict/aide_predict/bespoke_models/predicto
rs/evmutation.py:186: UserWarning: Mutation E386e is not supported by
EVCouplings statistics and will be ignored for scoring.
  warnings.warn(f"Mutation {subs_from}{subs_pos}{subs_to} is not supported by
EVCouplings statistics and will be ignored for scoring.")
/kfs2/projects/proteinml/repos/aide_predict/aide_predict/bespoke_models/predicto
rs/evmutation.py:186: UserWarning: Mutation R387r is not supported by
EVCouplings statistics and will be ignored for scoring.
  warnings.warn(f"Mutation {subs_from}{subs_pos}{subs_to} is not supported by
EVCouplings statistics and will be ignored for scoring.")
/kfs2/projects/proteinml/repos/aide_predict/aide_predict/bespoke_models/predicto
rs/evmutation.py:186: UserWarning: Mutation I388i is not supported by
EVCouplings statistics and will be ignored for scoring.
  warnings.warn(f"Mutation {subs_from}{subs_pos}{subs_to} is not supported by
EVCouplings statistics and will be ignored for scoring.")
/kfs2/projects/proteinml/repos/aide_predict/aide_predict/bespoke_models/predicto
rs/evmutation.py:186: UserWarning: Mutation R389r is not supported by
EVCouplings statistics and will be ignored for scoring.
  warnings.warn(f"Mutation {subs_from}{subs_pos}{subs_to} is not supported by
EVCouplings statistics and will be ignored for scoring.")
/kfs2/projects/proteinml/repos/aide_predict/aide_predict/bespoke_models/predicto
rs/evmutation.py:186: UserWarning: Mutation L390l is not supported by
EVCouplings statistics and will be ignored for scoring.
  warnings.warn(f"Mutation {subs_from}{subs_pos}{subs_to} is not supported by
EVCouplings statistics and will be ignored for scoring.")
/kfs2/projects/proteinml/repos/aide_predict/aide_predict/bespoke_models/predicto
rs/evmutation.py:186: UserWarning: Mutation E391e is not supported by
EVCouplings statistics and will be ignored for scoring.
  warnings.warn(f"Mutation {subs_from}{subs_pos}{subs_to} is not supported by
EVCouplings statistics and will be ignored for scoring.")
/kfs2/projects/proteinml/repos/aide_predict/aide_predict/bespoke_models/predicto
```

```
rs/evmutation.py:186: UserWarning: Mutation H392h is not supported by
EVCouplings statistics and will be ignored for scoring.
  warnings.warn(f"Mutation {subs_from}{subs_pos}{subs_to} is not supported by
EVCouplings statistics and will be ignored for scoring.")
/kfs2/projects/proteinml/repos/aide_predict/aide_predict/bespoke_models/predicto
rs/evmutation.py:186: UserWarning: Mutation H393h is not supported by
EVCouplings statistics and will be ignored for scoring.
  warnings.warn(f"Mutation {subs_from}{subs_pos}{subs_to} is not supported by
EVCouplings statistics and will be ignored for scoring.")
/kfs2/projects/proteinml/repos/aide_predict/aide_predict/bespoke_models/predicto
rs/evmutation.py:186: UserWarning: Mutation H394h is not supported by
EVCouplings statistics and will be ignored for scoring.
  warnings.warn(f"Mutation {subs_from}{subs_pos}{subs_to} is not supported by
EVCouplings statistics and will be ignored for scoring.")
/kfs2/projects/proteinml/repos/aide_predict/aide_predict/bespoke_models/predicto
rs/evmutation.py:186: UserWarning: Mutation H395h is not supported by
EVCouplings statistics and will be ignored for scoring.
  warnings.warn(f"Mutation {subs_from}{subs_pos}{subs_to} is not supported by
EVCouplings statistics and will be ignored for scoring.")
/kfs2/projects/proteinml/repos/aide_predict/aide_predict/bespoke_models/predicto
rs/evmutation.py:186: UserWarning: Mutation H396h is not supported by
EVCouplings statistics and will be ignored for scoring.
  warnings.warn(f"Mutation {subs_from}{subs_pos}{subs_to} is not supported by
EVCouplings statistics and will be ignored for scoring.")
/kfs2/projects/proteinml/repos/aide_predict/aide_predict/bespoke_models/predicto
rs/evmutation.py:186: UserWarning: Mutation H397h is not supported by
EVCouplings statistics and will be ignored for scoring.
  warnings.warn(f"Mutation {subs_from}{subs_pos}{subs_to} is not supported by
EVCouplings statistics and will be ignored for scoring.")
  0%|          | 1/127303 [00:00<11:16:43,  3.14it/s]/kfs2/projects/proteinml/re
pos/aide_predict/aide_predict/bespoke_models/predictors/evmutation.py:186:
UserWarning: Mutation M1m is not supported by EVCouplings statistics and will be
ignored for scoring.
  warnings.warn(f"Mutation {subs_from}{subs_pos}{subs_to} is not supported by
EVCouplings statistics and will be ignored for scoring.")
/kfs2/projects/proteinml/repos/aide_predict/aide_predict/bespoke_models/predicto
rs/evmutation.py:186: UserWarning: Mutation K2k is not supported by EVCouplings
statistics and will be ignored for scoring.
  warnings.warn(f"Mutation {subs_from}{subs_pos}{subs_to} is not supported by
EVCouplings statistics and will be ignored for scoring.")
/kfs2/projects/proteinml/repos/aide_predict/aide_predict/bespoke_models/predicto
rs/evmutation.py:186: UserWarning: Mutation G3g is not supported by EVCouplings
statistics and will be ignored for scoring.
  warnings.warn(f"Mutation {subs_from}{subs_pos}{subs_to} is not supported by
EVCouplings statistics and will be ignored for scoring.")
/kfs2/projects/proteinml/repos/aide_predict/aide_predict/bespoke_models/predicto
rs/evmutation.py:186: UserWarning: Mutation Y4y is not supported by EVCouplings
statistics and will be ignored for scoring.
```

```
  warnings.warn(f"Mutation {subs_from}{subs_pos}{subs_to} is not supported by
EVCouplings statistics and will be ignored for scoring.")
/kfs2/projects/proteinml/repos/aide_predict/aide_predict/bespoke_models/predicto
rs/evmutation.py:186: UserWarning: Mutation F5f is not supported by EVCouplings
statistics and will be ignored for scoring.
  warnings.warn(f"Mutation {subs_from}{subs_pos}{subs_to} is not supported by
EVCouplings statistics and will be ignored for scoring.")
/kfs2/projects/proteinml/repos/aide_predict/aide_predict/bespoke_models/predicto
rs/evmutation.py:186: UserWarning: Mutation G6g is not supported by EVCouplings
statistics and will be ignored for scoring.
  warnings.warn(f"Mutation {subs_from}{subs_pos}{subs_to} is not supported by
EVCouplings statistics and will be ignored for scoring.")
/kfs2/projects/proteinml/repos/aide_predict/aide_predict/bespoke_models/predicto
rs/evmutation.py:186: UserWarning: Mutation P7p is not supported by EVCouplings
statistics and will be ignored for scoring.
  warnings.warn(f"Mutation {subs_from}{subs_pos}{subs_to} is not supported by
EVCouplings statistics and will be ignored for scoring.")
/kfs2/projects/proteinml/repos/aide_predict/aide_predict/bespoke_models/predicto
rs/evmutation.py:186: UserWarning: Mutation Y8y is not supported by EVCouplings
statistics and will be ignored for scoring.
  warnings.warn(f"Mutation {subs_from}{subs_pos}{subs_to} is not supported by
EVCouplings statistics and will be ignored for scoring.")
/kfs2/projects/proteinml/repos/aide_predict/aide_predict/bespoke_models/predicto
rs/evmutation.py:186: UserWarning: Mutation G9g is not supported by EVCouplings
statistics and will be ignored for scoring.
  warnings.warn(f"Mutation {subs_from}{subs_pos}{subs_to} is not supported by
EVCouplings statistics and will be ignored for scoring.")
/kfs2/projects/proteinml/repos/aide_predict/aide_predict/bespoke_models/predicto
rs/evmutation.py:186: UserWarning: Mutation G10g is not supported by EVCouplings
statistics and will be ignored for scoring.
  warnings.warn(f"Mutation {subs_from}{subs_pos}{subs_to} is not supported by
EVCouplings statistics and will be ignored for scoring.")
/kfs2/projects/proteinml/repos/aide_predict/aide_predict/bespoke_models/predicto
rs/evmutation.py:186: UserWarning: Mutation Q11q is not supported by EVCouplings
statistics and will be ignored for scoring.
  warnings.warn(f"Mutation {subs_from}{subs_pos}{subs_to} is not supported by
EVCouplings statistics and will be ignored for scoring.")
/kfs2/projects/proteinml/repos/aide_predict/aide_predict/bespoke_models/predicto
rs/evmutation.py:186: UserWarning: Mutation Y12y is not supported by EVCouplings
statistics and will be ignored for scoring.
  warnings.warn(f"Mutation {subs_from}{subs_pos}{subs_to} is not supported by
EVCouplings statistics and will be ignored for scoring.")
/kfs2/projects/proteinml/repos/aide_predict/aide_predict/bespoke_models/predicto
rs/evmutation.py:186: UserWarning: Mutation V13v is not supported by EVCouplings
statistics and will be ignored for scoring.
  warnings.warn(f"Mutation {subs_from}{subs_pos}{subs_to} is not supported by
EVCouplings statistics and will be ignored for scoring.")
/kfs2/projects/proteinml/repos/aide_predict/aide_predict/bespoke_models/predicto
```

```
rs/evmutation.py:186: UserWarning: Mutation P14p is not supported by EVCouplings
statistics and will be ignored for scoring.
  warnings.warn(f"Mutation {subs_from}{subs_pos}{subs_to} is not supported by
EVCouplings statistics and will be ignored for scoring.")
/kfs2/projects/proteinml/repos/aide_predict/aide_predict/bespoke_models/predicto
rs/evmutation.py:186: UserWarning: Mutation E15e is not supported by EVCouplings
statistics and will be ignored for scoring.
  warnings.warn(f"Mutation {subs_from}{subs_pos}{subs_to} is not supported by
EVCouplings statistics and will be ignored for scoring.")
/kfs2/projects/proteinml/repos/aide_predict/aide_predict/bespoke_models/predicto
rs/evmutation.py:186: UserWarning: Mutation I16i is not supported by EVCouplings
statistics and will be ignored for scoring.
  warnings.warn(f"Mutation {subs_from}{subs_pos}{subs_to} is not supported by
EVCouplings statistics and will be ignored for scoring.")
/kfs2/projects/proteinml/repos/aide_predict/aide_predict/bespoke_models/predicto
rs/evmutation.py:186: UserWarning: Mutation L17l is not supported by EVCouplings
statistics and will be ignored for scoring.
  warnings.warn(f"Mutation {subs_from}{subs_pos}{subs_to} is not supported by
EVCouplings statistics and will be ignored for scoring.")
/kfs2/projects/proteinml/repos/aide_predict/aide_predict/bespoke_models/predicto
rs/evmutation.py:186: UserWarning: Mutation M18m is not supported by EVCouplings
statistics and will be ignored for scoring.
  warnings.warn(f"Mutation {subs_from}{subs_pos}{subs_to} is not supported by
EVCouplings statistics and will be ignored for scoring.")
/kfs2/projects/proteinml/repos/aide_predict/aide_predict/bespoke_models/predicto
rs/evmutation.py:186: UserWarning: Mutation G19g is not supported by EVCouplings
statistics and will be ignored for scoring.
  warnings.warn(f"Mutation {subs_from}{subs_pos}{subs_to} is not supported by
EVCouplings statistics and will be ignored for scoring.")
/kfs2/projects/proteinml/repos/aide_predict/aide_predict/bespoke_models/predicto
rs/evmutation.py:186: UserWarning: Mutation A20a is not supported by EVCouplings
statistics and will be ignored for scoring.
  warnings.warn(f"Mutation {subs_from}{subs_pos}{subs_to} is not supported by
EVCouplings statistics and will be ignored for scoring.")
/kfs2/projects/proteinml/repos/aide_predict/aide_predict/bespoke_models/predicto
rs/evmutation.py:186: UserWarning: Mutation L21l is not supported by EVCouplings
statistics and will be ignored for scoring.
  warnings.warn(f"Mutation {subs_from}{subs_pos}{subs_to} is not supported by
EVCouplings statistics and will be ignored for scoring.")
/kfs2/projects/proteinml/repos/aide_predict/aide_predict/bespoke_models/predicto
rs/evmutation.py:186: UserWarning: Mutation E22e is not supported by EVCouplings
statistics and will be ignored for scoring.
  warnings.warn(f"Mutation {subs_from}{subs_pos}{subs_to} is not supported by
EVCouplings statistics and will be ignored for scoring.")
/kfs2/projects/proteinml/repos/aide_predict/aide_predict/bespoke_models/predicto
rs/evmutation.py:186: UserWarning: Mutation E23e is not supported by EVCouplings
statistics and will be ignored for scoring.
  warnings.warn(f"Mutation {subs_from}{subs_pos}{subs_to} is not supported by
```

```
EVCouplings statistics and will be ignored for scoring.")
/kfs2/projects/proteinml/repos/aide_predict/aide_predict/bespoke_models/predicto
rs/evmutation.py:186: UserWarning: Mutation L24l is not supported by EVCouplings
statistics and will be ignored for scoring.
  warnings.warn(f"Mutation {subs_from}{subs_pos}{subs_to} is not supported by
EVCouplings statistics and will be ignored for scoring.")
/kfs2/projects/proteinml/repos/aide_predict/aide_predict/bespoke_models/predicto
rs/evmutation.py:186: UserWarning: Mutation E25e is not supported by EVCouplings
statistics and will be ignored for scoring.
  warnings.warn(f"Mutation {subs_from}{subs_pos}{subs_to} is not supported by
EVCouplings statistics and will be ignored for scoring.")
/kfs2/projects/proteinml/repos/aide_predict/aide_predict/bespoke_models/predicto
rs/evmutation.py:186: UserWarning: Mutation A26a is not supported by EVCouplings
statistics and will be ignored for scoring.
  warnings.warn(f"Mutation {subs_from}{subs_pos}{subs_to} is not supported by
EVCouplings statistics and will be ignored for scoring.")
/kfs2/projects/proteinml/repos/aide_predict/aide_predict/bespoke_models/predicto
rs/evmutation.py:186: UserWarning: Mutation A27a is not supported by EVCouplings
statistics and will be ignored for scoring.
  warnings.warn(f"Mutation {subs_from}{subs_pos}{subs_to} is not supported by
EVCouplings statistics and will be ignored for scoring.")
/kfs2/projects/proteinml/repos/aide_predict/aide_predict/bespoke_models/predicto
rs/evmutation.py:186: UserWarning: Mutation Y28y is not supported by EVCouplings
statistics and will be ignored for scoring.
  warnings.warn(f"Mutation {subs_from}{subs_pos}{subs_to} is not supported by
EVCouplings statistics and will be ignored for scoring.")
/kfs2/projects/proteinml/repos/aide_predict/aide_predict/bespoke_models/predicto
rs/evmutation.py:186: UserWarning: Mutation E29e is not supported by EVCouplings
statistics and will be ignored for scoring.
  warnings.warn(f"Mutation {subs_from}{subs_pos}{subs_to} is not supported by
EVCouplings statistics and will be ignored for scoring.")
/kfs2/projects/proteinml/repos/aide_predict/aide_predict/bespoke_models/predicto
rs/evmutation.py:186: UserWarning: Mutation G30g is not supported by EVCouplings
statistics and will be ignored for scoring.
  warnings.warn(f"Mutation {subs_from}{subs_pos}{subs_to} is not supported by
EVCouplings statistics and will be ignored for scoring.")
/kfs2/projects/proteinml/repos/aide_predict/aide_predict/bespoke_models/predicto
rs/evmutation.py:186: UserWarning: Mutation I31i is not supported by EVCouplings
statistics and will be ignored for scoring.
  warnings.warn(f"Mutation {subs_from}{subs_pos}{subs_to} is not supported by
EVCouplings statistics and will be ignored for scoring.")
/kfs2/projects/proteinml/repos/aide_predict/aide_predict/bespoke_models/predicto
rs/evmutation.py:186: UserWarning: Mutation M32m is not supported by EVCouplings
statistics and will be ignored for scoring.
  warnings.warn(f"Mutation {subs_from}{subs_pos}{subs_to} is not supported by
EVCouplings statistics and will be ignored for scoring.")
/kfs2/projects/proteinml/repos/aide_predict/aide_predict/bespoke_models/predicto
rs/evmutation.py:186: UserWarning: Mutation K33k is not supported by EVCouplings
```

```
statistics and will be ignored for scoring.
  warnings.warn(f"Mutation {subs_from}{subs_pos}{subs_to} is not supported by
EVCouplings statistics and will be ignored for scoring.")
/kfs2/projects/proteinml/repos/aide_predict/aide_predict/bespoke_models/predicto
rs/evmutation.py:186: UserWarning: Mutation D34d is not supported by EVCouplings
statistics and will be ignored for scoring.
  warnings.warn(f"Mutation {subs_from}{subs_pos}{subs_to} is not supported by
EVCouplings statistics and will be ignored for scoring.")
/kfs2/projects/proteinml/repos/aide_predict/aide_predict/bespoke_models/predicto
rs/evmutation.py:186: UserWarning: Mutation E35e is not supported by EVCouplings
statistics and will be ignored for scoring.
  warnings.warn(f"Mutation {subs_from}{subs_pos}{subs_to} is not supported by
EVCouplings statistics and will be ignored for scoring.")
/kfs2/projects/proteinml/repos/aide_predict/aide_predict/bespoke_models/predicto
rs/evmutation.py:186: UserWarning: Mutation S36s is not supported by EVCouplings
statistics and will be ignored for scoring.
  warnings.warn(f"Mutation {subs_from}{subs_pos}{subs_to} is not supported by
EVCouplings statistics and will be ignored for scoring.")
/kfs2/projects/proteinml/repos/aide_predict/aide_predict/bespoke_models/predicto
rs/evmutation.py:186: UserWarning: Mutation F37f is not supported by EVCouplings
statistics and will be ignored for scoring.
  warnings.warn(f"Mutation {subs_from}{subs_pos}{subs_to} is not supported by
EVCouplings statistics and will be ignored for scoring.")
/kfs2/projects/proteinml/repos/aide_predict/aide_predict/bespoke_models/predicto
rs/evmutation.py:186: UserWarning: Mutation W38w is not supported by EVCouplings
statistics and will be ignored for scoring.
  warnings.warn(f"Mutation {subs_from}{subs_pos}{subs_to} is not supported by
EVCouplings statistics and will be ignored for scoring.")
/kfs2/projects/proteinml/repos/aide_predict/aide_predict/bespoke_models/predicto
rs/evmutation.py:186: UserWarning: Mutation K39k is not supported by EVCouplings
statistics and will be ignored for scoring.
  warnings.warn(f"Mutation {subs_from}{subs_pos}{subs_to} is not supported by
EVCouplings statistics and will be ignored for scoring.")
/kfs2/projects/proteinml/repos/aide_predict/aide_predict/bespoke_models/predicto
rs/evmutation.py:186: UserWarning: Mutation E40e is not supported by EVCouplings
statistics and will be ignored for scoring.
  warnings.warn(f"Mutation {subs_from}{subs_pos}{subs_to} is not supported by
EVCouplings statistics and will be ignored for scoring.")
/kfs2/projects/proteinml/repos/aide_predict/aide_predict/bespoke_models/predicto
rs/evmutation.py:186: UserWarning: Mutation F41f is not supported by EVCouplings
statistics and will be ignored for scoring.
  warnings.warn(f"Mutation {subs_from}{subs_pos}{subs_to} is not supported by
EVCouplings statistics and will be ignored for scoring.")
/kfs2/projects/proteinml/repos/aide_predict/aide_predict/bespoke_models/predicto
rs/evmutation.py:186: UserWarning: Mutation N42n is not supported by EVCouplings
statistics and will be ignored for scoring.
  warnings.warn(f"Mutation {subs_from}{subs_pos}{subs_to} is not supported by
EVCouplings statistics and will be ignored for scoring.")
```

```
/kfs2/projects/proteinml/repos/aide_predict/aide_predict/bespoke_models/predicto
rs/evmutation.py:186: UserWarning: Mutation D43d is not supported by EVCouplings
statistics and will be ignored for scoring.
  warnings.warn(f"Mutation {subs_from}{subs_pos}{subs_to} is not supported by
EVCouplings statistics and will be ignored for scoring.")
/kfs2/projects/proteinml/repos/aide_predict/aide_predict/bespoke_models/predicto
rs/evmutation.py:186: UserWarning: Mutation L44l is not supported by EVCouplings
statistics and will be ignored for scoring.
  warnings.warn(f"Mutation {subs_from}{subs_pos}{subs_to} is not supported by
EVCouplings statistics and will be ignored for scoring.")
/kfs2/projects/proteinml/repos/aide_predict/aide_predict/bespoke_models/predicto
rs/evmutation.py:186: UserWarning: Mutation L45l is not supported by EVCouplings
statistics and will be ignored for scoring.
  warnings.warn(f"Mutation {subs_from}{subs_pos}{subs_to} is not supported by
EVCouplings statistics and will be ignored for scoring.")
/kfs2/projects/proteinml/repos/aide_predict/aide_predict/bespoke_models/predicto
rs/evmutation.py:186: UserWarning: Mutation R46r is not supported by EVCouplings
statistics and will be ignored for scoring.
  warnings.warn(f"Mutation {subs_from}{subs_pos}{subs_to} is not supported by
EVCouplings statistics and will be ignored for scoring.")
/kfs2/projects/proteinml/repos/aide_predict/aide_predict/bespoke_models/predicto
rs/evmutation.py:186: UserWarning: Mutation D47d is not supported by EVCouplings
statistics and will be ignored for scoring.
  warnings.warn(f"Mutation {subs_from}{subs_pos}{subs_to} is not supported by
EVCouplings statistics and will be ignored for scoring.")
/kfs2/projects/proteinml/repos/aide_predict/aide_predict/bespoke_models/predicto
rs/evmutation.py:186: UserWarning: Mutation Y48y is not supported by EVCouplings
statistics and will be ignored for scoring.
  warnings.warn(f"Mutation {subs_from}{subs_pos}{subs_to} is not supported by
EVCouplings statistics and will be ignored for scoring.")
/kfs2/projects/proteinml/repos/aide_predict/aide_predict/bespoke_models/predicto
rs/evmutation.py:186: UserWarning: Mutation D134d is not supported by
EVCouplings statistics and will be ignored for scoring.
  warnings.warn(f"Mutation {subs_from}{subs_pos}{subs_to} is not supported by
EVCouplings statistics and will be ignored for scoring.")
/kfs2/projects/proteinml/repos/aide_predict/aide_predict/bespoke_models/predicto
rs/evmutation.py:186: UserWarning: Mutation I136i is not supported by
EVCouplings statistics and will be ignored for scoring.
  warnings.warn(f"Mutation {subs_from}{subs_pos}{subs_to} is not supported by
EVCouplings statistics and will be ignored for scoring.")
/kfs2/projects/proteinml/repos/aide_predict/aide_predict/bespoke_models/predicto
rs/evmutation.py:186: UserWarning: Mutation R137r is not supported by
EVCouplings statistics and will be ignored for scoring.
  warnings.warn(f"Mutation {subs_from}{subs_pos}{subs_to} is not supported by
EVCouplings statistics and will be ignored for scoring.")
/kfs2/projects/proteinml/repos/aide_predict/aide_predict/bespoke_models/predicto
rs/evmutation.py:186: UserWarning: Mutation Q138q is not supported by
EVCouplings statistics and will be ignored for scoring.
```

```
  warnings.warn(f"Mutation {subs_from}{subs_pos}{subs_to} is not supported by
EVCouplings statistics and will be ignored for scoring.")
/kfs2/projects/proteinml/repos/aide_predict/aide_predict/bespoke_models/predicto
rs/evmutation.py:186: UserWarning: Mutation S157s is not supported by
EVCouplings statistics and will be ignored for scoring.
  warnings.warn(f"Mutation {subs_from}{subs_pos}{subs_to} is not supported by
EVCouplings statistics and will be ignored for scoring.")
/kfs2/projects/proteinml/repos/aide_predict/aide_predict/bespoke_models/predicto
rs/evmutation.py:186: UserWarning: Mutation G158g is not supported by
EVCouplings statistics and will be ignored for scoring.
  warnings.warn(f"Mutation {subs_from}{subs_pos}{subs_to} is not supported by
EVCouplings statistics and will be ignored for scoring.")
/kfs2/projects/proteinml/repos/aide_predict/aide_predict/bespoke_models/predicto
rs/evmutation.py:186: UserWarning: Mutation S159s is not supported by
EVCouplings statistics and will be ignored for scoring.
  warnings.warn(f"Mutation {subs_from}{subs_pos}{subs_to} is not supported by
EVCouplings statistics and will be ignored for scoring.")
/kfs2/projects/proteinml/repos/aide_predict/aide_predict/bespoke_models/predicto
rs/evmutation.py:186: UserWarning: Mutation L177l is not supported by
EVCouplings statistics and will be ignored for scoring.
  warnings.warn(f"Mutation {subs_from}{subs_pos}{subs_to} is not supported by
EVCouplings statistics and will be ignored for scoring.")
/kfs2/projects/proteinml/repos/aide_predict/aide_predict/bespoke_models/predicto
rs/evmutation.py:186: UserWarning: Mutation T179t is not supported by
EVCouplings statistics and will be ignored for scoring.
  warnings.warn(f"Mutation {subs_from}{subs_pos}{subs_to} is not supported by
EVCouplings statistics and will be ignored for scoring.")
/kfs2/projects/proteinml/repos/aide_predict/aide_predict/bespoke_models/predicto
rs/evmutation.py:186: UserWarning: Mutation T180t is not supported by
EVCouplings statistics and will be ignored for scoring.
  warnings.warn(f"Mutation {subs_from}{subs_pos}{subs_to} is not supported by
EVCouplings statistics and will be ignored for scoring.")
/kfs2/projects/proteinml/repos/aide_predict/aide_predict/bespoke_models/predicto
rs/evmutation.py:186: UserWarning: Mutation Y181y is not supported by
EVCouplings statistics and will be ignored for scoring.
  warnings.warn(f"Mutation {subs_from}{subs_pos}{subs_to} is not supported by
EVCouplings statistics and will be ignored for scoring.")
/kfs2/projects/proteinml/repos/aide_predict/aide_predict/bespoke_models/predicto
rs/evmutation.py:186: UserWarning: Mutation V183D is not supported by
EVCouplings statistics and will be ignored for scoring.
  warnings.warn(f"Mutation {subs_from}{subs_pos}{subs_to} is not supported by
EVCouplings statistics and will be ignored for scoring.")
/kfs2/projects/proteinml/repos/aide_predict/aide_predict/bespoke_models/predicto
rs/evmutation.py:186: UserWarning: Mutation F184G is not supported by
EVCouplings statistics and will be ignored for scoring.
  warnings.warn(f"Mutation {subs_from}{subs_pos}{subs_to} is not supported by
EVCouplings statistics and will be ignored for scoring.")
/kfs2/projects/proteinml/repos/aide_predict/aide_predict/bespoke_models/predicto
```

```
rs/evmutation.py:186: UserWarning: Mutation P192p is not supported by
EVCouplings statistics and will be ignored for scoring.
  warnings.warn(f"Mutation {subs_from}{subs_pos}{subs_to} is not supported by
EVCouplings statistics and will be ignored for scoring.")
/kfs2/projects/proteinml/repos/aide_predict/aide_predict/bespoke_models/predicto
rs/evmutation.py:186: UserWarning: Mutation I196i is not supported by
EVCouplings statistics and will be ignored for scoring.
  warnings.warn(f"Mutation {subs_from}{subs_pos}{subs_to} is not supported by
EVCouplings statistics and will be ignored for scoring.")
/kfs2/projects/proteinml/repos/aide_predict/aide_predict/bespoke_models/predicto
rs/evmutation.py:186: UserWarning: Mutation V197v is not supported by
EVCouplings statistics and will be ignored for scoring.
  warnings.warn(f"Mutation {subs_from}{subs_pos}{subs_to} is not supported by
EVCouplings statistics and will be ignored for scoring.")
/kfs2/projects/proteinml/repos/aide_predict/aide_predict/bespoke_models/predicto
rs/evmutation.py:186: UserWarning: Mutation P213p is not supported by
EVCouplings statistics and will be ignored for scoring.
  warnings.warn(f"Mutation {subs_from}{subs_pos}{subs_to} is not supported by
EVCouplings statistics and will be ignored for scoring.")
/kfs2/projects/proteinml/repos/aide_predict/aide_predict/bespoke_models/predicto
rs/evmutation.py:186: UserWarning: Mutation E214e is not supported by
EVCouplings statistics and will be ignored for scoring.
  warnings.warn(f"Mutation {subs_from}{subs_pos}{subs_to} is not supported by
EVCouplings statistics and will be ignored for scoring.")
/kfs2/projects/proteinml/repos/aide_predict/aide_predict/bespoke_models/predicto
rs/evmutation.py:186: UserWarning: Mutation K215k is not supported by
EVCouplings statistics and will be ignored for scoring.
  warnings.warn(f"Mutation {subs_from}{subs_pos}{subs_to} is not supported by
EVCouplings statistics and will be ignored for scoring.")
/kfs2/projects/proteinml/repos/aide_predict/aide_predict/bespoke_models/predicto
rs/evmutation.py:186: UserWarning: Mutation E216e is not supported by
EVCouplings statistics and will be ignored for scoring.
  warnings.warn(f"Mutation {subs_from}{subs_pos}{subs_to} is not supported by
EVCouplings statistics and will be ignored for scoring.")
/kfs2/projects/proteinml/repos/aide_predict/aide_predict/bespoke_models/predicto
rs/evmutation.py:186: UserWarning: Mutation G217g is not supported by
EVCouplings statistics and will be ignored for scoring.
  warnings.warn(f"Mutation {subs_from}{subs_pos}{subs_to} is not supported by
EVCouplings statistics and will be ignored for scoring.")
/kfs2/projects/proteinml/repos/aide_predict/aide_predict/bespoke_models/predicto
rs/evmutation.py:186: UserWarning: Mutation G260g is not supported by
EVCouplings statistics and will be ignored for scoring.
  warnings.warn(f"Mutation {subs_from}{subs_pos}{subs_to} is not supported by
EVCouplings statistics and will be ignored for scoring.")
/kfs2/projects/proteinml/repos/aide_predict/aide_predict/bespoke_models/predicto
rs/evmutation.py:186: UserWarning: Mutation K261k is not supported by
EVCouplings statistics and will be ignored for scoring.
  warnings.warn(f"Mutation {subs_from}{subs_pos}{subs_to} is not supported by
```

```
EVCouplings statistics and will be ignored for scoring.")
/kfs2/projects/proteinml/repos/aide_predict/aide_predict/bespoke_models/predicto
rs/evmutation.py:186: UserWarning: Mutation H262h is not supported by
EVCouplings statistics and will be ignored for scoring.
  warnings.warn(f"Mutation {subs_from}{subs_pos}{subs_to} is not supported by
EVCouplings statistics and will be ignored for scoring.")
/kfs2/projects/proteinml/repos/aide_predict/aide_predict/bespoke_models/predicto
rs/evmutation.py:186: UserWarning: Mutation A263a is not supported by
EVCouplings statistics and will be ignored for scoring.
  warnings.warn(f"Mutation {subs_from}{subs_pos}{subs_to} is not supported by
EVCouplings statistics and will be ignored for scoring.")
/kfs2/projects/proteinml/repos/aide_predict/aide_predict/bespoke_models/predicto
rs/evmutation.py:186: UserWarning: Mutation A264a is not supported by
EVCouplings statistics and will be ignored for scoring.
  warnings.warn(f"Mutation {subs_from}{subs_pos}{subs_to} is not supported by
EVCouplings statistics and will be ignored for scoring.")
/kfs2/projects/proteinml/repos/aide_predict/aide_predict/bespoke_models/predicto
rs/evmutation.py:186: UserWarning: Mutation H275h is not supported by
EVCouplings statistics and will be ignored for scoring.
  warnings.warn(f"Mutation {subs_from}{subs_pos}{subs_to} is not supported by
EVCouplings statistics and will be ignored for scoring.")
/kfs2/projects/proteinml/repos/aide_predict/aide_predict/bespoke_models/predicto
rs/evmutation.py:186: UserWarning: Mutation G276g is not supported by
EVCouplings statistics and will be ignored for scoring.
  warnings.warn(f"Mutation {subs_from}{subs_pos}{subs_to} is not supported by
EVCouplings statistics and will be ignored for scoring.")
/kfs2/projects/proteinml/repos/aide_predict/aide_predict/bespoke_models/predicto
rs/evmutation.py:186: UserWarning: Mutation S277s is not supported by
EVCouplings statistics and will be ignored for scoring.
  warnings.warn(f"Mutation {subs_from}{subs_pos}{subs_to} is not supported by
EVCouplings statistics and will be ignored for scoring.")
/kfs2/projects/proteinml/repos/aide_predict/aide_predict/bespoke_models/predicto
rs/evmutation.py:186: UserWarning: Mutation K278k is not supported by
EVCouplings statistics and will be ignored for scoring.
  warnings.warn(f"Mutation {subs_from}{subs_pos}{subs_to} is not supported by
EVCouplings statistics and will be ignored for scoring.")
/kfs2/projects/proteinml/repos/aide_predict/aide_predict/bespoke_models/predicto
rs/evmutation.py:186: UserWarning: Mutation T279t is not supported by
EVCouplings statistics and will be ignored for scoring.
  warnings.warn(f"Mutation {subs_from}{subs_pos}{subs_to} is not supported by
EVCouplings statistics and will be ignored for scoring.")
/kfs2/projects/proteinml/repos/aide_predict/aide_predict/bespoke_models/predicto
rs/evmutation.py:186: UserWarning: Mutation F280f is not supported by
EVCouplings statistics and will be ignored for scoring.
  warnings.warn(f"Mutation {subs_from}{subs_pos}{subs_to} is not supported by
EVCouplings statistics and will be ignored for scoring.")
/kfs2/projects/proteinml/repos/aide_predict/aide_predict/bespoke_models/predicto
rs/evmutation.py:186: UserWarning: Mutation V281v is not supported by
```

```
EVCouplings statistics and will be ignored for scoring.
  warnings.warn(f"Mutation {subs_from}{subs_pos}{subs_to} is not supported by
EVCouplings statistics and will be ignored for scoring.")
/kfs2/projects/proteinml/repos/aide_predict/aide_predict/bespoke_models/predicto
rs/evmutation.py:186: UserWarning: Mutation L282l is not supported by
EVCouplings statistics and will be ignored for scoring.
  warnings.warn(f"Mutation {subs_from}{subs_pos}{subs_to} is not supported by
EVCouplings statistics and will be ignored for scoring.")
/kfs2/projects/proteinml/repos/aide_predict/aide_predict/bespoke_models/predicto
rs/evmutation.py:186: UserWarning: Mutation Q283q is not supported by
EVCouplings statistics and will be ignored for scoring.
  warnings.warn(f"Mutation {subs_from}{subs_pos}{subs_to} is not supported by
EVCouplings statistics and will be ignored for scoring.")
/kfs2/projects/proteinml/repos/aide_predict/aide_predict/bespoke_models/predicto
rs/evmutation.py:186: UserWarning: Mutation D284d is not supported by
EVCouplings statistics and will be ignored for scoring.
  warnings.warn(f"Mutation {subs_from}{subs_pos}{subs_to} is not supported by
EVCouplings statistics and will be ignored for scoring.")
/kfs2/projects/proteinml/repos/aide_predict/aide_predict/bespoke_models/predicto
rs/evmutation.py:186: UserWarning: Mutation D285d is not supported by
EVCouplings statistics and will be ignored for scoring.
  warnings.warn(f"Mutation {subs_from}{subs_pos}{subs_to} is not supported by
EVCouplings statistics and will be ignored for scoring.")
/kfs2/projects/proteinml/repos/aide_predict/aide_predict/bespoke_models/predicto
rs/evmutation.py:186: UserWarning: Mutation W286w is not supported by
EVCouplings statistics and will be ignored for scoring.
  warnings.warn(f"Mutation {subs_from}{subs_pos}{subs_to} is not supported by
EVCouplings statistics and will be ignored for scoring.")
/kfs2/projects/proteinml/repos/aide_predict/aide_predict/bespoke_models/predicto
rs/evmutation.py:186: UserWarning: Mutation G287g is not supported by
EVCouplings statistics and will be ignored for scoring.
  warnings.warn(f"Mutation {subs_from}{subs_pos}{subs_to} is not supported by
EVCouplings statistics and will be ignored for scoring.")
/kfs2/projects/proteinml/repos/aide_predict/aide_predict/bespoke_models/predicto
rs/evmutation.py:186: UserWarning: Mutation Q288q is not supported by
EVCouplings statistics and will be ignored for scoring.
  warnings.warn(f"Mutation {subs_from}{subs_pos}{subs_to} is not supported by
EVCouplings statistics and will be ignored for scoring.")
/kfs2/projects/proteinml/repos/aide_predict/aide_predict/bespoke_models/predicto
rs/evmutation.py:186: UserWarning: Mutation V289v is not supported by
EVCouplings statistics and will be ignored for scoring.
  warnings.warn(f"Mutation {subs_from}{subs_pos}{subs_to} is not supported by
EVCouplings statistics and will be ignored for scoring.")
/kfs2/projects/proteinml/repos/aide_predict/aide_predict/bespoke_models/predicto
rs/evmutation.py:186: UserWarning: Mutation Q290q is not supported by
EVCouplings statistics and will be ignored for scoring.
  warnings.warn(f"Mutation {subs_from}{subs_pos}{subs_to} is not supported by
EVCouplings statistics and will be ignored for scoring.")
```

```
/kfs2/projects/proteinml/repos/aide_predict/aide_predict/bespoke_models/predicto
rs/evmutation.py:186: UserWarning: Mutation V291v is not supported by
EVCouplings statistics and will be ignored for scoring.
  warnings.warn(f"Mutation {subs_from}{subs_pos}{subs_to} is not supported by
EVCouplings statistics and will be ignored for scoring.")
/kfs2/projects/proteinml/repos/aide_predict/aide_predict/bespoke_models/predicto
rs/evmutation.py:186: UserWarning: Mutation S292s is not supported by
EVCouplings statistics and will be ignored for scoring.
  warnings.warn(f"Mutation {subs_from}{subs_pos}{subs_to} is not supported by
EVCouplings statistics and will be ignored for scoring.")
/kfs2/projects/proteinml/repos/aide_predict/aide_predict/bespoke_models/predicto
rs/evmutation.py:186: UserWarning: Mutation V378v is not supported by
EVCouplings statistics and will be ignored for scoring.
  warnings.warn(f"Mutation {subs_from}{subs_pos}{subs_to} is not supported by
EVCouplings statistics and will be ignored for scoring.")
/kfs2/projects/proteinml/repos/aide_predict/aide_predict/bespoke_models/predicto
rs/evmutation.py:186: UserWarning: Mutation L379l is not supported by
EVCouplings statistics and will be ignored for scoring.
  warnings.warn(f"Mutation {subs_from}{subs_pos}{subs_to} is not supported by
EVCouplings statistics and will be ignored for scoring.")
/kfs2/projects/proteinml/repos/aide_predict/aide_predict/bespoke_models/predicto
rs/evmutation.py:186: UserWarning: Mutation N380n is not supported by
EVCouplings statistics and will be ignored for scoring.
  warnings.warn(f"Mutation {subs_from}{subs_pos}{subs_to} is not supported by
EVCouplings statistics and will be ignored for scoring.")
/kfs2/projects/proteinml/repos/aide_predict/aide_predict/bespoke_models/predicto
rs/evmutation.py:186: UserWarning: Mutation H381h is not supported by
EVCouplings statistics and will be ignored for scoring.
  warnings.warn(f"Mutation {subs_from}{subs_pos}{subs_to} is not supported by
EVCouplings statistics and will be ignored for scoring.")
/kfs2/projects/proteinml/repos/aide_predict/aide_predict/bespoke_models/predicto
rs/evmutation.py:186: UserWarning: Mutation P382p is not supported by
EVCouplings statistics and will be ignored for scoring.
  warnings.warn(f"Mutation {subs_from}{subs_pos}{subs_to} is not supported by
EVCouplings statistics and will be ignored for scoring.")
/kfs2/projects/proteinml/repos/aide_predict/aide_predict/bespoke_models/predicto
rs/evmutation.py:186: UserWarning: Mutation Y383y is not supported by
EVCouplings statistics and will be ignored for scoring.
  warnings.warn(f"Mutation {subs_from}{subs_pos}{subs_to} is not supported by
EVCouplings statistics and will be ignored for scoring.")
/kfs2/projects/proteinml/repos/aide_predict/aide_predict/bespoke_models/predicto
rs/evmutation.py:186: UserWarning: Mutation V384v is not supported by
EVCouplings statistics and will be ignored for scoring.
  warnings.warn(f"Mutation {subs_from}{subs_pos}{subs_to} is not supported by
EVCouplings statistics and will be ignored for scoring.")
/kfs2/projects/proteinml/repos/aide_predict/aide_predict/bespoke_models/predicto
rs/evmutation.py:186: UserWarning: Mutation R385r is not supported by
EVCouplings statistics and will be ignored for scoring.
```

```
  warnings.warn(f"Mutation {subs_from}{subs_pos}{subs_to} is not supported by
EVCouplings statistics and will be ignored for scoring.")
/kfs2/projects/proteinml/repos/aide_predict/aide_predict/bespoke_models/predicto
rs/evmutation.py:186: UserWarning: Mutation E386e is not supported by
EVCouplings statistics and will be ignored for scoring.
  warnings.warn(f"Mutation {subs_from}{subs_pos}{subs_to} is not supported by
EVCouplings statistics and will be ignored for scoring.")
/kfs2/projects/proteinml/repos/aide_predict/aide_predict/bespoke_models/predicto
rs/evmutation.py:186: UserWarning: Mutation R387r is not supported by
EVCouplings statistics and will be ignored for scoring.
  warnings.warn(f"Mutation {subs_from}{subs_pos}{subs_to} is not supported by
EVCouplings statistics and will be ignored for scoring.")
/kfs2/projects/proteinml/repos/aide_predict/aide_predict/bespoke_models/predicto
rs/evmutation.py:186: UserWarning: Mutation I388i is not supported by
EVCouplings statistics and will be ignored for scoring.
  warnings.warn(f"Mutation {subs_from}{subs_pos}{subs_to} is not supported by
EVCouplings statistics and will be ignored for scoring.")
/kfs2/projects/proteinml/repos/aide_predict/aide_predict/bespoke_models/predicto
rs/evmutation.py:186: UserWarning: Mutation R389r is not supported by
EVCouplings statistics and will be ignored for scoring.
  warnings.warn(f"Mutation {subs_from}{subs_pos}{subs_to} is not supported by
EVCouplings statistics and will be ignored for scoring.")
/kfs2/projects/proteinml/repos/aide_predict/aide_predict/bespoke_models/predicto
rs/evmutation.py:186: UserWarning: Mutation L390l is not supported by
EVCouplings statistics and will be ignored for scoring.
  warnings.warn(f"Mutation {subs_from}{subs_pos}{subs_to} is not supported by
EVCouplings statistics and will be ignored for scoring.")
/kfs2/projects/proteinml/repos/aide_predict/aide_predict/bespoke_models/predicto
rs/evmutation.py:186: UserWarning: Mutation E391e is not supported by
EVCouplings statistics and will be ignored for scoring.
  warnings.warn(f"Mutation {subs_from}{subs_pos}{subs_to} is not supported by
EVCouplings statistics and will be ignored for scoring.")
/kfs2/projects/proteinml/repos/aide_predict/aide_predict/bespoke_models/predicto
rs/evmutation.py:186: UserWarning: Mutation H392h is not supported by
EVCouplings statistics and will be ignored for scoring.
  warnings.warn(f"Mutation {subs_from}{subs_pos}{subs_to} is not supported by
EVCouplings statistics and will be ignored for scoring.")
/kfs2/projects/proteinml/repos/aide_predict/aide_predict/bespoke_models/predicto
rs/evmutation.py:186: UserWarning: Mutation H393h is not supported by
EVCouplings statistics and will be ignored for scoring.
  warnings.warn(f"Mutation {subs_from}{subs_pos}{subs_to} is not supported by
EVCouplings statistics and will be ignored for scoring.")
/kfs2/projects/proteinml/repos/aide_predict/aide_predict/bespoke_models/predicto
rs/evmutation.py:186: UserWarning: Mutation H394h is not supported by
EVCouplings statistics and will be ignored for scoring.
  warnings.warn(f"Mutation {subs_from}{subs_pos}{subs_to} is not supported by
EVCouplings statistics and will be ignored for scoring.")
/kfs2/projects/proteinml/repos/aide_predict/aide_predict/bespoke_models/predicto
```

```
rs/evmutation.py:186: UserWarning: Mutation H395h is not supported by
EVCouplings statistics and will be ignored for scoring.
  warnings.warn(f"Mutation {subs_from}{subs_pos}{subs_to} is not supported by
EVCouplings statistics and will be ignored for scoring.")
/kfs2/projects/proteinml/repos/aide_predict/aide_predict/bespoke_models/predicto
rs/evmutation.py:186: UserWarning: Mutation H396h is not supported by
EVCouplings statistics and will be ignored for scoring.
  warnings.warn(f"Mutation {subs_from}{subs_pos}{subs_to} is not supported by
EVCouplings statistics and will be ignored for scoring.")
/kfs2/projects/proteinml/repos/aide_predict/aide_predict/bespoke_models/predicto
rs/evmutation.py:186: UserWarning: Mutation H397h is not supported by
EVCouplings statistics and will be ignored for scoring.
  warnings.warn(f"Mutation {subs_from}{subs_pos}{subs_to} is not supported by
EVCouplings statistics and will be ignored for scoring.")
/kfs2/projects/proteinml/repos/aide_predict/aide_predict/bespoke_models/predicto
rs/evmutation.py:186: UserWarning: Mutation V183Q is not supported by
EVCouplings statistics and will be ignored for scoring.
  warnings.warn(f"Mutation {subs_from}{subs_pos}{subs_to} is not supported by
EVCouplings statistics and will be ignored for scoring.")
/kfs2/projects/proteinml/repos/aide_predict/aide_predict/bespoke_models/predicto
rs/evmutation.py:186: UserWarning: Mutation F184I is not supported by
EVCouplings statistics and will be ignored for scoring.
  warnings.warn(f"Mutation {subs_from}{subs_pos}{subs_to} is not supported by
EVCouplings statistics and will be ignored for scoring.")
/kfs2/projects/proteinml/repos/aide_predict/aide_predict/bespoke_models/predicto
rs/evmutation.py:186: UserWarning: Mutation V183C is not supported by
EVCouplings statistics and will be ignored for scoring.
  warnings.warn(f"Mutation {subs_from}{subs_pos}{subs_to} is not supported by
EVCouplings statistics and will be ignored for scoring.")
/kfs2/projects/proteinml/repos/aide_predict/aide_predict/bespoke_models/predicto
rs/evmutation.py:186: UserWarning: Mutation V183A is not supported by
EVCouplings statistics and will be ignored for scoring.
  warnings.warn(f"Mutation {subs_from}{subs_pos}{subs_to} is not supported by
EVCouplings statistics and will be ignored for scoring.")
/kfs2/projects/proteinml/repos/aide_predict/aide_predict/bespoke_models/predicto
rs/evmutation.py:186: UserWarning: Mutation F184E is not supported by
EVCouplings statistics and will be ignored for scoring.
  warnings.warn(f"Mutation {subs_from}{subs_pos}{subs_to} is not supported by
EVCouplings statistics and will be ignored for scoring.")
/kfs2/projects/proteinml/repos/aide_predict/aide_predict/bespoke_models/predicto
rs/evmutation.py:186: UserWarning: Mutation V183T is not supported by
EVCouplings statistics and will be ignored for scoring.
  warnings.warn(f"Mutation {subs_from}{subs_pos}{subs_to} is not supported by
EVCouplings statistics and will be ignored for scoring.")
/kfs2/projects/proteinml/repos/aide_predict/aide_predict/bespoke_models/predicto
rs/evmutation.py:186: UserWarning: Mutation F184T is not supported by
EVCouplings statistics and will be ignored for scoring.
  warnings.warn(f"Mutation {subs_from}{subs_pos}{subs_to} is not supported by
```

```
EVCouplings statistics and will be ignored for scoring.")
/kfs2/projects/proteinml/repos/aide_predict/aide_predict/bespoke_models/predicto
rs/evmutation.py:186: UserWarning: Mutation V183M is not supported by
EVCouplings statistics and will be ignored for scoring.
  warnings.warn(f"Mutation {subs_from}{subs_pos}{subs_to} is not supported by
EVCouplings statistics and will be ignored for scoring.")
/kfs2/projects/proteinml/repos/aide_predict/aide_predict/bespoke_models/predicto
rs/evmutation.py:186: UserWarning: Mutation F184D is not supported by
EVCouplings statistics and will be ignored for scoring.
  warnings.warn(f"Mutation {subs_from}{subs_pos}{subs_to} is not supported by
EVCouplings statistics and will be ignored for scoring.")
/kfs2/projects/proteinml/repos/aide_predict/aide_predict/bespoke_models/predicto
rs/evmutation.py:186: UserWarning: Mutation V183F is not supported by
EVCouplings statistics and will be ignored for scoring.
  warnings.warn(f"Mutation {subs_from}{subs_pos}{subs_to} is not supported by
EVCouplings statistics and will be ignored for scoring.")
/kfs2/projects/proteinml/repos/aide_predict/aide_predict/bespoke_models/predicto
rs/evmutation.py:186: UserWarning: Mutation F184A is not supported by
EVCouplings statistics and will be ignored for scoring.
  warnings.warn(f"Mutation {subs_from}{subs_pos}{subs_to} is not supported by
EVCouplings statistics and will be ignored for scoring.")
/kfs2/projects/proteinml/repos/aide_predict/aide_predict/bespoke_models/predicto
rs/evmutation.py:186: UserWarning: Mutation F184W is not supported by
EVCouplings statistics and will be ignored for scoring.
  warnings.warn(f"Mutation {subs_from}{subs_pos}{subs_to} is not supported by
EVCouplings statistics and will be ignored for scoring.")
/kfs2/projects/proteinml/repos/aide_predict/aide_predict/bespoke_models/predicto
rs/evmutation.py:186: UserWarning: Mutation V183E is not supported by
EVCouplings statistics and will be ignored for scoring.
  warnings.warn(f"Mutation {subs_from}{subs_pos}{subs_to} is not supported by
EVCouplings statistics and will be ignored for scoring.")
/kfs2/projects/proteinml/repos/aide_predict/aide_predict/bespoke_models/predicto
rs/evmutation.py:186: UserWarning: Mutation V183G is not supported by
EVCouplings statistics and will be ignored for scoring.
  warnings.warn(f"Mutation {subs_from}{subs_pos}{subs_to} is not supported by
EVCouplings statistics and will be ignored for scoring.")
/kfs2/projects/proteinml/repos/aide_predict/aide_predict/bespoke_models/predicto
rs/evmutation.py:186: UserWarning: Mutation V183W is not supported by
EVCouplings statistics and will be ignored for scoring.
  warnings.warn(f"Mutation {subs_from}{subs_pos}{subs_to} is not supported by
EVCouplings statistics and will be ignored for scoring.")
/kfs2/projects/proteinml/repos/aide_predict/aide_predict/bespoke_models/predicto
rs/evmutation.py:186: UserWarning: Mutation F184Q is not supported by
EVCouplings statistics and will be ignored for scoring.
  warnings.warn(f"Mutation {subs_from}{subs_pos}{subs_to} is not supported by
EVCouplings statistics and will be ignored for scoring.")
/kfs2/projects/proteinml/repos/aide_predict/aide_predict/bespoke_models/predicto
rs/evmutation.py:186: UserWarning: Mutation V183S is not supported by
```

```
EVCouplings statistics and will be ignored for scoring.
  warnings.warn(f"Mutation {subs_from}{subs_pos}{subs_to} is not supported by
EVCouplings statistics and will be ignored for scoring.")
/kfs2/projects/proteinml/repos/aide_predict/aide_predict/bespoke_models/predicto
rs/evmutation.py:186: UserWarning: Mutation F184V is not supported by
EVCouplings statistics and will be ignored for scoring.
  warnings.warn(f"Mutation {subs_from}{subs_pos}{subs_to} is not supported by
EVCouplings statistics and will be ignored for scoring.")
/kfs2/projects/proteinml/repos/aide_predict/aide_predict/bespoke_models/predicto
rs/evmutation.py:186: UserWarning: Mutation F184S is not supported by
EVCouplings statistics and will be ignored for scoring.
  warnings.warn(f"Mutation {subs_from}{subs_pos}{subs_to} is not supported by
EVCouplings statistics and will be ignored for scoring.")
/kfs2/projects/proteinml/repos/aide_predict/aide_predict/bespoke_models/predicto
rs/evmutation.py:186: UserWarning: Mutation F184N is not supported by
EVCouplings statistics and will be ignored for scoring.
  warnings.warn(f"Mutation {subs_from}{subs_pos}{subs_to} is not supported by
EVCouplings statistics and will be ignored for scoring.")
/kfs2/projects/proteinml/repos/aide_predict/aide_predict/bespoke_models/predicto
rs/evmutation.py:186: UserWarning: Mutation V183L is not supported by
EVCouplings statistics and will be ignored for scoring.
  warnings.warn(f"Mutation {subs_from}{subs_pos}{subs_to} is not supported by
EVCouplings statistics and will be ignored for scoring.")
/kfs2/projects/proteinml/repos/aide_predict/aide_predict/bespoke_models/predicto
rs/evmutation.py:186: UserWarning: Mutation F184K is not supported by
EVCouplings statistics and will be ignored for scoring.
  warnings.warn(f"Mutation {subs_from}{subs_pos}{subs_to} is not supported by
EVCouplings statistics and will be ignored for scoring.")
/kfs2/projects/proteinml/repos/aide_predict/aide_predict/bespoke_models/predicto
rs/evmutation.py:186: UserWarning: Mutation V183I is not supported by
EVCouplings statistics and will be ignored for scoring.
  warnings.warn(f"Mutation {subs_from}{subs_pos}{subs_to} is not supported by
EVCouplings statistics and will be ignored for scoring.")
/kfs2/projects/proteinml/repos/aide_predict/aide_predict/bespoke_models/predicto
rs/evmutation.py:186: UserWarning: Mutation V183K is not supported by
EVCouplings statistics and will be ignored for scoring.
  warnings.warn(f"Mutation {subs_from}{subs_pos}{subs_to} is not supported by
EVCouplings statistics and will be ignored for scoring.")
/kfs2/projects/proteinml/repos/aide_predict/aide_predict/bespoke_models/predicto
rs/evmutation.py:186: UserWarning: Mutation V183Y is not supported by
EVCouplings statistics and will be ignored for scoring.
  warnings.warn(f"Mutation {subs_from}{subs_pos}{subs_to} is not supported by
EVCouplings statistics and will be ignored for scoring.")
/kfs2/projects/proteinml/repos/aide_predict/aide_predict/bespoke_models/predicto
rs/evmutation.py:186: UserWarning: Mutation V183H is not supported by
EVCouplings statistics and will be ignored for scoring.
  warnings.warn(f"Mutation {subs_from}{subs_pos}{subs_to} is not supported by
EVCouplings statistics and will be ignored for scoring.")
```

```
/kfs2/projects/proteinml/repos/aide_predict/aide_predict/bespoke_models/predicto
rs/evmutation.py:186: UserWarning: Mutation F184R is not supported by
EVCouplings statistics and will be ignored for scoring.
  warnings.warn(f"Mutation {subs_from}{subs_pos}{subs_to} is not supported by
EVCouplings statistics and will be ignored for scoring.")
/kfs2/projects/proteinml/repos/aide_predict/aide_predict/bespoke_models/predicto
rs/evmutation.py:186: UserWarning: Mutation F184H is not supported by
EVCouplings statistics and will be ignored for scoring.
  warnings.warn(f"Mutation {subs_from}{subs_pos}{subs_to} is not supported by
EVCouplings statistics and will be ignored for scoring.")
/kfs2/projects/proteinml/repos/aide_predict/aide_predict/bespoke_models/predicto
rs/evmutation.py:186: UserWarning: Mutation V183P is not supported by
EVCouplings statistics and will be ignored for scoring.
  warnings.warn(f"Mutation {subs_from}{subs_pos}{subs_to} is not supported by
EVCouplings statistics and will be ignored for scoring.")
/kfs2/projects/proteinml/repos/aide_predict/aide_predict/bespoke_models/predicto
rs/evmutation.py:186: UserWarning: Mutation F184C is not supported by
EVCouplings statistics and will be ignored for scoring.
  warnings.warn(f"Mutation {subs_from}{subs_pos}{subs_to} is not supported by
EVCouplings statistics and will be ignored for scoring.")
/kfs2/projects/proteinml/repos/aide_predict/aide_predict/bespoke_models/predicto
rs/evmutation.py:186: UserWarning: Mutation F184Y is not supported by
EVCouplings statistics and will be ignored for scoring.
  warnings.warn(f"Mutation {subs_from}{subs_pos}{subs_to} is not supported by
EVCouplings statistics and will be ignored for scoring.")
/kfs2/projects/proteinml/repos/aide_predict/aide_predict/bespoke_models/predicto
rs/evmutation.py:186: UserWarning: Mutation V183R is not supported by
EVCouplings statistics and will be ignored for scoring.
  warnings.warn(f"Mutation {subs_from}{subs_pos}{subs_to} is not supported by
EVCouplings statistics and will be ignored for scoring.")
/kfs2/projects/proteinml/repos/aide_predict/aide_predict/bespoke_models/predicto
rs/evmutation.py:186: UserWarning: Mutation F184P is not supported by
EVCouplings statistics and will be ignored for scoring.
  warnings.warn(f"Mutation {subs_from}{subs_pos}{subs_to} is not supported by
EVCouplings statistics and will be ignored for scoring.")
/kfs2/projects/proteinml/repos/aide_predict/aide_predict/bespoke_models/predicto
rs/evmutation.py:186: UserWarning: Mutation F184L is not supported by
EVCouplings statistics and will be ignored for scoring.
  warnings.warn(f"Mutation {subs_from}{subs_pos}{subs_to} is not supported by
EVCouplings statistics and will be ignored for scoring.")
/kfs2/projects/proteinml/repos/aide_predict/aide_predict/bespoke_models/predicto
rs/evmutation.py:186: UserWarning: Mutation V183N is not supported by
EVCouplings statistics and will be ignored for scoring.
  warnings.warn(f"Mutation {subs_from}{subs_pos}{subs_to} is not supported by
EVCouplings statistics and will be ignored for scoring.")
/kfs2/projects/proteinml/repos/aide_predict/aide_predict/bespoke_models/predicto
rs/evmutation.py:186: UserWarning: Mutation F184M is not supported by
EVCouplings statistics and will be ignored for scoring.
```

```
  warnings.warn(f"Mutation {subs_from}{subs_pos}{subs_to} is not supported by
EVCouplings statistics and will be ignored for scoring.")
100%|      | 127303/127303 [00:13<00:00, 9646.80it/s]
100%|      | 1/1 [00:00<00:00, 13443.28it/s]
WARNING:aide_predict.bespoke_models.base:Model is already fitted. Skipping
INFO:aide_predict.bespoke_models.base:Transforming input using EVMutationWrapper
100%|      | 127303/127303 [00:12<00:00, 9989.78it/s]
100%|      | 1/1 [00:00<00:00, 14979.66it/s]
WARNING:aide_predict.bespoke_models.base:Model is already fitted. Skipping
INFO:aide_predict.bespoke_models.base:Transforming input using EVMutationWrapper
100%|      | 127303/127303 [00:12<00:00, 9947.29it/s]
100%|      | 1/1 [00:00<00:00, 13888.42it/s]
WARNING:aide_predict.bespoke_models.base:Model is already fitted. Skipping
INFO:aide_predict.bespoke_models.base:Transforming input using EVMutationWrapper
100%|      | 127303/127303 [00:12<00:00, 10061.22it/s]
100%|      | 1/1 [00:00<00:00, 9822.73it/s]
WARNING:aide_predict.bespoke_models.base:Model is already fitted. Skipping
INFO:aide_predict.bespoke_models.base:Transforming input using EVMutationWrapper
100%|      | 127304/127304 [00:12<00:00, 10092.08it/s]
100%|      | 1/1 [00:00<00:00, 12905.55it/s]
```

[45]:
```
joblib.dump(evc_none_none_scores, 'evc_none_none_scores.joblib')
```

[45]:
```
['evc_none_none_scores.joblib']
```

1.4.3 2.3 MSATransformer

[17]:
```
msa_likelihood = ap.MSATransformerLikelihoodWrapper(
    wt=wt,
    metadata_folder=os.path.join('.', 'data', 'epistatic', 'msalikelihood'),
    device='cuda:1',
    marginal_method='wildtype_marginal',
)
msa_likelihood.fit()
```

[17]:
```
MSATransformerLikelihoodWrapper(device='cuda:1',
                                marginal_method='wildtype_marginal',
metadata_folder='./data/epistatic/msalikelihood',
                                use_cache=True,
                                wt=ProteinSequence(id='TARGET/1-397',
seq='mkgyfgpyggqyvpeilmga…'))
```

[47]:
```
msa_none_none_scores = cv_1(msa_likelihood, X, y)
```

```
/kfs2/projects/proteinml/repos/aide_predict/aide_predict/bespoke_models/base.py:
388: UserWarning: This model expects no fit, but received input. Ignoring input:
[ProteinSequence(id=None, seq='mkgyfgpyggqyvpeilmga…'),
ProteinSequence(id=None, seq='mkgyfgpyggqyvpeilmga…'),
```

```
ProteinSequence(id=None, seq='mkgyfgpyggqyvpeilmga…'),
ProteinSequence(id=None, seq='mkgyfgpyggqyvpeilmga…'),
ProteinSequence(id=None, seq='mkgyfgpyggqyvpeilmga…'),
ProteinSequence(id=None, seq='mkgyfgpyggqyvpeilmga…'),
ProteinSequence(id=None, seq='mkgyfgpyggqyvpeilmga…'),
ProteinSequence(id=None, seq='mkgyfgpyggqyvpeilmga…'),
ProteinSequence(id=None, seq='mkgyfgpyggqyvpeilmga…'),
ProteinSequence(id=None, seq='mkgyfgpyggqyvpeilmga…'),
ProteinSequence(id=None, seq='mkgyfgpyggqyvpeilmga…'),
ProteinSequence(id=None, seq='mkgyfgpyggqyvpeilmga…'),
ProteinSequence(id=None, seq='mkgyfgpyggqyvpeilmga…'),
ProteinSequence(id=None, seq='mkgyfgpyggqyvpeilmga…'),
ProteinSequence(id=None, seq='mkgyfgpyggqyvpeilmga…'),
ProteinSequence(id=None, seq='mkgyfgpyggqyvpeilmga…'),
ProteinSequence(id=None, seq='mkgyfgpyggqyvpeilmga…'),
ProteinSequence(id=None, seq='mkgyfgpyggqyvpeilmga…'),
ProteinSequence(id=None, seq='mkgyfgpyggqyvpeilmga…'),
ProteinSequence(id=None, seq='mkgyfgpyggqyvpeilmga…'),
ProteinSequence(id=None, seq='mkgyfgpyggqyvpeilmga…'),
ProteinSequence(id=None, seq='mkgyfgpyggqyvpeilmga…'),
ProteinSequence(id=None, seq='mkgyfgpyggqyvpeilmga…'),
ProteinSequence(id=None, seq='mkgyfgpyggqyvpeilmga…'),
ProteinSequence(id=None, seq='mkgyfgpyggqyvpeilmga…'),
ProteinSequence(id=None, seq='mkgyfgpyggqyvpeilmga…'),
ProteinSequence(id=None, seq='mkgyfgpyggqyvpeilmga…'),
ProteinSequence(id=None, seq='mkgyfgpyggqyvpeilmga…'),
ProteinSequence(id=None, seq='mkgyfgpyggqyvpeilmga…'),
ProteinSequence(id=None, seq='mkgyfgpyggqyvpeilmga…'),
ProteinSequence(id=None, seq='mkgyfgpyggqyvpeilmga…'),
ProteinSequence(id=None, seq='mkgyfgpyggqyvpeilmga…'),
ProteinSequence(id=None, seq='mkgyfgpyggqyvpeilmga…'),
ProteinSequence(id=None, seq='mkgyfgpyggqyvpeilmga…'),
ProteinSequence(id=None, seq='mkgyfgpyggqyvpeilmga…'),
ProteinSequence(id=None, seq='mkgyfgpyggqyvpeilmga…'),
ProteinSequence(id=None, seq='mkgyfgpyggqyvpeilmga…'),
ProteinSequence(id=None, seq='mkgyfgpyggqyvpeilmga…'),
ProteinSequence(id=None, seq='mkgyfgpyggqyvpeilmga…'),
ProteinSequence(id=None, seq='mkgyfgpyggqyvpeilmga…'),
ProteinSequence(id=None, seq='mkgyfgpyggqyvpeilmga…'),
ProteinSequence(id=None, seq='mkgyfgpyggqyvpeilmga…'),
ProteinSequence(id=None, seq='mkgyfgpyggqyvpeilmga…'),
ProteinSequence(id=None, seq='mkgyfgpyggqyvpeilmga…'),
ProteinSequence(id=None, seq='mkgyfgpyggqyvpeilmga…'),
ProteinSequence(id=None, seq='mkgyfgpyggqyvpeilmga…'),
ProteinSequence(id=None, seq='mkgyfgpyggqyvpeilmga…'),
ProteinSequence(id=None, seq='mkgyfgpyggqyvpeilmga…'),
ProteinSequence(id=None, seq='mkgyfgpyggqyvpeilmga…'),
ProteinSequence(id=None, seq='mkgyfgpyggqyvpeilmga…'),
```

```
ProteinSequence(id=None, seq='mkgyfgpyggqyvpeilmga…'),
ProteinSequence(id=None, seq='mkgyfgpyggqyvpeilmga…'),
ProteinSequence(id=None, seq='mkgyfgpyggqyvpeilmga…'),
ProteinSequence(id=None, seq='mkgyfgpyggqyvpeilmga…'),
ProteinSequence(id=None, seq='mkgyfgpyggqyvpeilmga…'),
ProteinSequence(id=None, seq='mkgyfgpyggqyvpeilmga…'),
ProteinSequence(id=None, seq='mkgyfgpyggqyvpeilmga…'),
ProteinSequence(id=None, seq='mkgyfgpyggqyvpeilmga…'),
ProteinSequence(id=None, seq='mkgyfgpyggqyvpeilmga…'),
ProteinSequence(id=None, seq='mkgyfgpyggqyvpeilmga…'),
ProteinSequence(id=None, seq='mkgyfgpyggqyvpeilmga…'),
ProteinSequence(id=None, seq='mkgyfgpyggqyvpeilmga…'),
ProteinSequence(id=None, seq='mkgyfgpyggqyvpeilmga…'),
ProteinSequence(id=None, seq='mkgyfgpyggqyvpeilmga…'),
ProteinSequence(id=None, seq='mkgyfgpyggqyvpeilmga…'),
ProteinSequence(id=None, seq='mkgyfgpyggqyvpeilmga…'),
ProteinSequence(id=None, seq='mkgyfgpyggqyvpeilmga…'),
ProteinSequence(id=None, seq='mkgyfgpyggqyvpeilmga…'),
ProteinSequence(id=None, seq='mkgyfgpyggqyvpeilmga…'),
ProteinSequence(id=None, seq='mkgyfgpyggqyvpeilmga…'),
ProteinSequence(id=None, seq='mkgyfgpyggqyvpeilmga…'),
ProteinSequence(id=None, seq='mkgyfgpyggqyvpeilmga…'),
ProteinSequence(id=None, seq='mkgyfgpyggqyvpeilmga…'),
ProteinSequence(id=None, seq='mkgyfgpyggqyvpeilmga…'),
ProteinSequence(id=None, seq='mkgyfgpyggqyvpeilmga…'),
ProteinSequence(id=None, seq='mkgyfgpyggqyvpeilmga…'),
ProteinSequence(id=None, seq='mkgyfgpyggqyvpeilmga…'),
ProteinSequence(id=None, seq='mkgyfgpyggqyvpeilmga…'),
ProteinSequence(id=None, seq='mkgyfgpyggqyvpeilmga…'),
ProteinSequence(id=None, seq='mkgyfgpyggqyvpeilmga…'),
ProteinSequence(id=None, seq='mkgyfgpyggqyvpeilmga…'),
ProteinSequence(id=None, seq='mkgyfgpyggqyvpeilmga…'),
ProteinSequence(id=None, seq='mkgyfgpyggqyvpeilmga…'),
ProteinSequence(id=None, seq='mkgyfgpyggqyvpeilmga…'),
ProteinSequence(id=None, seq='mkgyfgpyggqyvpeilmga…'),
ProteinSequence(id=None, seq='mkgyfgpyggqyvpeilmga…'),
ProteinSequence(id=None, seq='mkgyfgpyggqyvpeilmga…'),
ProteinSequence(id=None, seq='mkgyfgpyggqyvpeilmga…'),
ProteinSequence(id=None, seq='mkgyfgpyggqyvpeilmga…'),
ProteinSequence(id=None, seq='mkgyfgpyggqyvpeilmga…'),
ProteinSequence(id=None, seq='mkgyfgpyggqyvpeilmga…'),
ProteinSequence(id=None, seq='mkgyfgpyggqyvpeilmga…'),
ProteinSequence(id=None, seq='mkgyfgpyggqyvpeilmga…'),
ProteinSequence(id=None, seq='mkgyfgpyggqyvpeilmga…'),
ProteinSequence(id=None, seq='mkgyfgpyggqyvpeilmga…'),
ProteinSequence(id=None, seq='mkgyfgpyggqyvpeilmga…'),
ProteinSequence(id=None, seq='mkgyfgpyggqyvpeilmga…'),
ProteinSequence(id=None, seq='mkgyfgpyggqyvpeilmga…'),
```

```
ProteinSequence(id=None, seq='mkgyfgpyggqyvpeilmga…'),
ProteinSequence(id=None, seq='mkgyfgpyggqyvpeilmga…'),
ProteinSequence(id=None, seq='mkgyfgpyggqyvpeilmga…'),
ProteinSequence(id=None, seq='mkgyfgpyggqyvpeilmga…'),
ProteinSequence(id=None, seq='mkgyfgpyggqyvpeilmga…'),
ProteinSequence(id=None, seq='mkgyfgpyggqyvpeilmga…'),
ProteinSequence(id=None, seq='mkgyfgpyggqyvpeilmga…'),
ProteinSequence(id=None, seq='mkgyfgpyggqyvpeilmga…'),
ProteinSequence(id=None, seq='mkgyfgpyggqyvpeilmga…'),
ProteinSequence(id=None, seq='mkgyfgpyggqyvpeilmga…'),
ProteinSequence(id=None, seq='mkgyfgpyggqyvpeilmga…'),
ProteinSequence(id=None, seq='mkgyfgpyggqyvpeilmga…'),
ProteinSequence(id=None, seq='mkgyfgpyggqyvpeilmga…'),
ProteinSequence(id=None, seq='mkgyfgpyggqyvpeilmga…'),
ProteinSequence(id=None, seq='mkgyfgpyggqyvpeilmga…'),
ProteinSequence(id=None, seq='mkgyfgpyggqyvpeilmga…'),
ProteinSequence(id=None, seq='mkgyfgpyggqyvpeilmga…'),
ProteinSequence(id=None, seq='mkgyfgpyggqyvpeilmga…'),
ProteinSequence(id=None, seq='mkgyfgpyggqyvpeilmga…'),
ProteinSequence(id=None, seq='mkgyfgpyggqyvpeilmga…'),
ProteinSequence(id=None, seq='mkgyfgpyggqyvpeilmga…'),
ProteinSequence(id=None, seq='mkgyfgpyggqyvpeilmga…'),
ProteinSequence(id=None, seq='mkgyfgpyggqyvpeilmga…'),
ProteinSequence(id=None, seq='mkgyfgpyggqyvpeilmga…'),
ProteinSequence(id=None, seq='mkgyfgpyggqyvpeilmga…'),
ProteinSequence(id=None, seq='mkgyfgpyggqyvpeilmga…'),
ProteinSequence(id=None, seq='mkgyfgpyggqyvpeilmga…'),
ProteinSequence(id=None, seq='mkgyfgpyggqyvpeilmga…'),
ProteinSequence(id=None, seq='mkgyfgpyggqyvpeilmga…'),
ProteinSequence(id=None, seq='mkgyfgpyggqyvpeilmga…'),
ProteinSequence(id=None, seq='mkgyfgpyggqyvpeilmga…'),
ProteinSequence(id=None, seq='mkgyfgpyggqyvpeilmga…'),
ProteinSequence(id=None, seq='mkgyfgpyggqyvpeilmga…'),
ProteinSequence(id=None, seq='mkgyfgpyggqyvpeilmga…'),
ProteinSequence(id=None, seq='mkgyfgpyggqyvpeilmga…'),
ProteinSequence(id=None, seq='mkgyfgpyggqyvpeilmga…'),
ProteinSequence(id=None, seq='mkgyfgpyggqyvpeilmga…'),
ProteinSequence(id=None, seq='mkgyfgpyggqyvpeilmga…'),
ProteinSequence(id=None, seq='mkgyfgpyggqyvpeilmga…'),
ProteinSequence(id=None, seq='mkgyfgpyggqyvpeilmga…'),
ProteinSequence(id=None, seq='mkgyfgpyggqyvpeilmga…'),
ProteinSequence(id=None, seq='mkgyfgpyggqyvpeilmga…'),
ProteinSequence(id=None, seq='mkgyfgpyggqyvpeilmga…'),
ProteinSequence(id=None, seq='mkgyfgpyggqyvpeilmga…'),
ProteinSequence(id=None, seq='mkgyfgpyggqyvpeilmga…'),
ProteinSequence(id=None, seq='mkgyfgpyggqyvpeilmga…'),
ProteinSequence(id=None, seq='mkgyfgpyggqyvpeilmga…'),
ProteinSequence(id=None, seq='mkgyfgpyggqyvpeilmga…'),
```

```
ProteinSequence(id=None, seq='mkgyfgpyggqyvpeilmga…'),
ProteinSequence(id=None, seq='mkgyfgpyggqyvpeilmga…'),
ProteinSequence(id=None, seq='mkgyfgpyggqyvpeilmga…'),
ProteinSequence(id=None, seq='mkgyfgpyggqyvpeilmga…'),
ProteinSequence(id=None, seq='mkgyfgpyggqyvpeilmga…'),
ProteinSequence(id=None, seq='mkgyfgpyggqyvpeilmga…'),
ProteinSequence(id=None, seq='mkgyfgpyggqyvpeilmga…'),
ProteinSequence(id=None, seq='mkgyfgpyggqyvpeilmga…'),
ProteinSequence(id=None, seq='mkgyfgpyggqyvpeilmga…'),
ProteinSequence(id=None, seq='mkgyfgpyggqyvpeilmga…'),
ProteinSequence(id=None, seq='mkgyfgpyggqyvpeilmga…'),
ProteinSequence(id=None, seq='mkgyfgpyggqyvpeilmga…'),
ProteinSequence(id=None, seq='mkgyfgpyggqyvpeilmga…'),
ProteinSequence(id=None, seq='mkgyfgpyggqyvpeilmga…'),
ProteinSequence(id=None, seq='mkgyfgpyggqyvpeilmga…'),
ProteinSequence(id=None, seq='mkgyfgpyggqyvpeilmga…'),
ProteinSequence(id=None, seq='mkgyfgpyggqyvpeilmga…'),
ProteinSequence(id=None, seq='mkgyfgpyggqyvpeilmga…'),
ProteinSequence(id=None, seq='mkgyfgpyggqyvpeilmga…'),
ProteinSequence(id=None, seq='mkgyfgpyggqyvpeilmga…'),
ProteinSequence(id=None, seq='mkgyfgpyggqyvpeilmga…'),
ProteinSequence(id=None, seq='mkgyfgpyggqyvpeilmga…'),
ProteinSequence(id=None, seq='mkgyfgpyggqyvpeilmga…'),
ProteinSequence(id=None, seq='mkgyfgpyggqyvpeilmga…'),
ProteinSequence(id=None, seq='mkgyfgpyggqyvpeilmga…'),
ProteinSequence(id=None, seq='mkgyfgpyggqyvpeilmga…'),
ProteinSequence(id=None, seq='mkgyfgpyggqyvpeilmga…'),
ProteinSequence(id=None, seq='mkgyfgpyggqyvpeilmga…'),
ProteinSequence(id=None, seq='mkgyfgpyggqyvpeilmga…'),
ProteinSequence(id=None, seq='mkgyfgpyggqyvpeilmga…'),
ProteinSequence(id=None, seq='mkgyfgpyggqyvpeilmga…'),
ProteinSequence(id=None, seq='mkgyfgpyggqyvpeilmga…'),
ProteinSequence(id=None, seq='mkgyfgpyggqyvpeilmga…'),
ProteinSequence(id=None, seq='mkgyfgpyggqyvpeilmga…'),
ProteinSequence(id=None, seq='mkgyfgpyggqyvpeilmga…'),
ProteinSequence(id=None, seq='mkgyfgpyggqyvpeilmga…'),
ProteinSequence(id=None, seq='mkgyfgpyggqyvpeilmga…'),
ProteinSequence(id=None, seq='mkgyfgpyggqyvpeilmga…'),
ProteinSequence(id=None, seq='mkgyfgpyggqyvpeilmga…'),
ProteinSequence(id=None, seq='mkgyfgpyggqyvpeilmga…'),
ProteinSequence(id=None, seq='mkgyfgpyggqyvpeilmga…'),
ProteinSequence(id=None, seq='mkgyfgpyggqyvpeilmga…'),
ProteinSequence(id=None, seq='mkgyfgpyggqyvpeilmga…'),
ProteinSequence(id=None, seq='mkgyfgpyggqyvpeilmga…'),
ProteinSequence(id=None, seq='mkgyfgpyggqyvpeilmga…'),
ProteinSequence(id=None, seq='mkgyfgpyggqyvpeilmga…'),
ProteinSequence(id=None, seq='mkgyfgpyggqyvpeilmga…'),
ProteinSequence(id=None, seq='mkgyfgpyggqyvpeilmga…'),
```

```
ProteinSequence(id=None, seq='mkgyfgpyggqyvpeilmga…'),
ProteinSequence(id=None, seq='mkgyfgpyggqyvpeilmga…'),
ProteinSequence(id=None, seq='mkgyfgpyggqyvpeilmga…'),
ProteinSequence(id=None, seq='mkgyfgpyggqyvpeilmga…'),
ProteinSequence(id=None, seq='mkgyfgpyggqyvpeilmga…'),
ProteinSequence(id=None, seq='mkgyfgpyggqyvpeilmga…'),
ProteinSequence(id=None, seq='mkgyfgpyggqyvpeilmga…'),
ProteinSequence(id=None, seq='mkgyfgpyggqyvpeilmga…'),
ProteinSequence(id=None, seq='mkgyfgpyggqyvpeilmga…'),
ProteinSequence(id=None, seq='mkgyfgpyggqyvpeilmga…'),
ProteinSequence(id=None, seq='mkgyfgpyggqyvpeilmga…'),
ProteinSequence(id=None, seq='mkgyfgpyggqyvpeilmga…'),
ProteinSequence(id=None, seq='mkgyfgpyggqyvpeilmga…'),
ProteinSequence(id=None, seq='mkgyfgpyggqyvpeilmga…'),
ProteinSequence(id=None, seq='mkgyfgpyggqyvpeilmga…'),
ProteinSequence(id=None, seq='mkgyfgpyggqyvpeilmga…'),
ProteinSequence(id=None, seq='mkgyfgpyggqyvpeilmga…'),
ProteinSequence(id=None, seq='mkgyfgpyggqyvpeilmga…'),
ProteinSequence(id=None, seq='mkgyfgpyggqyvpeilmga…'),
ProteinSequence(id=None, seq='mkgyfgpyggqyvpeilmga…'),
ProteinSequence(id=None, seq='mkgyfgpyggqyvpeilmga…'),
ProteinSequence(id=None, seq='mkgyfgpyggqyvpeilmga…'),
ProteinSequence(id=None, seq='mkgyfgpyggqyvpeilmga…'),
ProteinSequence(id=None, seq='mkgyfgpyggqyvpeilmga…'),
ProteinSequence(id=None, seq='mkgyfgpyggqyvpeilmga…'),
ProteinSequence(id=None, seq='mkgyfgpyggqyvpeilmga…'),
ProteinSequence(id=None, seq='mkgyfgpyggqyvpeilmga…'),
ProteinSequence(id=None, seq='mkgyfgpyggqyvpeilmga…'),
ProteinSequence(id=None, seq='mkgyfgpyggqyvpeilmga…'),
ProteinSequence(id=None, seq='mkgyfgpyggqyvpeilmga…'),
ProteinSequence(id=None, seq='mkgyfgpyggqyvpeilmga…'),
ProteinSequence(id=None, seq='mkgyfgpyggqyvpeilmga…'),
ProteinSequence(id=None, seq='mkgyfgpyggqyvpeilmga…'),
ProteinSequence(id=None, seq='mkgyfgpyggqyvpeilmga…'),
ProteinSequence(id=None, seq='mkgyfgpyggqyvpeilmga…'),
ProteinSequence(id=None, seq='mkgyfgpyggqyvpeilmga…'),
ProteinSequence(id=None, seq='mkgyfgpyggqyvpeilmga…'),
ProteinSequence(id=None, seq='mkgyfgpyggqyvpeilmga…'),
ProteinSequence(id=None, seq='mkgyfgpyggqyvpeilmga…'),
ProteinSequence(id=None, seq='mkgyfgpyggqyvpeilmga…'),
ProteinSequence(id=None, seq='mkgyfgpyggqyvpeilmga…'),
ProteinSequence(id=None, seq='mkgyfgpyggqyvpeilmga…'),
ProteinSequence(id=None, seq='mkgyfgpyggqyvpeilmga…'),
ProteinSequence(id=None, seq='mkgyfgpyggqyvpeilmga…'),
ProteinSequence(id=None, seq='mkgyfgpyggqyvpeilmga…'),
ProteinSequence(id=None, seq='mkgyfgpyggqyvpeilmga…'),
ProteinSequence(id=None, seq='mkgyfgpyggqyvpeilmga…'),
ProteinSequence(id=None, seq='mkgyfgpyggqyvpeilmga…'),
```

```
ProteinSequence(id=None, seq='mkgyfgpyggqyvpeilmga…'),
ProteinSequence(id=None, seq='mkgyfgpyggqyvpeilmga…'),
ProteinSequence(id=None, seq='mkgyfgpyggqyvpeilmga…'),
ProteinSequence(id=None, seq='mkgyfgpyggqyvpeilmga…'),
ProteinSequence(id=None, seq='mkgyfgpyggqyvpeilmga…'),
ProteinSequence(id=None, seq='mkgyfgpyggqyvpeilmga…'),
ProteinSequence(id=None, seq='mkgyfgpyggqyvpeilmga…'),
ProteinSequence(id=None, seq='mkgyfgpyggqyvpeilmga…'),
ProteinSequence(id=None, seq='mkgyfgpyggqyvpeilmga…'),
ProteinSequence(id=None, seq='mkgyfgpyggqyvpeilmga…'),
ProteinSequence(id=None, seq='mkgyfgpyggqyvpeilmga…'),
ProteinSequence(id=None, seq='mkgyfgpyggqyvpeilmga…'),
ProteinSequence(id=None, seq='mkgyfgpyggqyvpeilmga…'),
ProteinSequence(id=None, seq='mkgyfgpyggqyvpeilmga…'),
ProteinSequence(id=None, seq='mkgyfgpyggqyvpeilmga…'),
ProteinSequence(id=None, seq='mkgyfgpyggqyvpeilmga…'),
ProteinSequence(id=None, seq='mkgyfgpyggqyvpeilmga…'),
ProteinSequence(id=None, seq='mkgyfgpyggqyvpeilmga…'),
ProteinSequence(id=None, seq='mkgyfgpyggqyvpeilmga…'),
ProteinSequence(id=None, seq='mkgyfgpyggqyvpeilmga…'),
ProteinSequence(id=None, seq='mkgyfgpyggqyvpeilmga…'),
ProteinSequence(id=None, seq='mkgyfgpyggqyvpeilmga…'),
ProteinSequence(id=None, seq='mkgyfgpyggqyvpeilmga…'),
ProteinSequence(id=None, seq='mkgyfgpyggqyvpeilmga…'),
ProteinSequence(id=None, seq='mkgyfgpyggqyvpeilmga…'),
ProteinSequence(id=None, seq='mkgyfgpyggqyvpeilmga…'),
ProteinSequence(id=None, seq='mkgyfgpyggqyvpeilmga…'),
ProteinSequence(id=None, seq='mkgyfgpyggqyvpeilmga…'),
ProteinSequence(id=None, seq='mkgyfgpyggqyvpeilmga…'),
ProteinSequence(id=None, seq='mkgyfgpyggqyvpeilmga…'),
ProteinSequence(id=None, seq='mkgyfgpyggqyvpeilmga…'),
ProteinSequence(id=None, seq='mkgyfgpyggqyvpeilmga…'),
ProteinSequence(id=None, seq='mkgyfgpyggqyvpeilmga…'),
ProteinSequence(id=None, seq='mkgyfgpyggqyvpeilmga…'),
ProteinSequence(id=None, seq='mkgyfgpyggqyvpeilmga…'),
ProteinSequence(id=None, seq='mkgyfgpyggqyvpeilmga…'),
ProteinSequence(id=None, seq='mkgyfgpyggqyvpeilmga…'),
ProteinSequence(id=None, seq='mkgyfgpyggqyvpeilmga…'),
ProteinSequence(id=None, seq='mkgyfgpyggqyvpeilmga…'),
ProteinSequence(id=None, seq='mkgyfgpyggqyvpeilmga…'),
ProteinSequence(id=None, seq='mkgyfgpyggqyvpeilmga…'),
ProteinSequence(id=None, seq='mkgyfgpyggqyvpeilmga…'),
ProteinSequence(id=None, seq='mkgyfgpyggqyvpeilmga…'),
ProteinSequence(id=None, seq='mkgyfgpyggqyvpeilmga…'),
ProteinSequence(id=None, seq='mkgyfgpyggqyvpeilmga…'),
ProteinSequence(id=None, seq='mkgyfgpyggqyvpeilmga…'),
ProteinSequence(id=None, seq='mkgyfgpyggqyvpeilmga…'),
ProteinSequence(id=None, seq='mkgyfgpyggqyvpeilmga…'),
```

```
ProteinSequence(id=None, seq='mkgyfgpyggqyvpeilmga…'),
ProteinSequence(id=None, seq='mkgyfgpyggqyvpeilmga…'),
ProteinSequence(id=None, seq='mkgyfgpyggqyvpeilmga…'),
ProteinSequence(id=None, seq='mkgyfgpyggqyvpeilmga…'),
ProteinSequence(id=None, seq='mkgyfgpyggqyvpeilmga…'),
ProteinSequence(id=None, seq='mkgyfgpyggqyvpeilmga…'),
ProteinSequence(id=None, seq='mkgyfgpyggqyvpeilmga…'),
ProteinSequence(id=None, seq='mkgyfgpyggqyvpeilmga…'),
ProteinSequence(id=None, seq='mkgyfgpyggqyvpeilmga…'),
ProteinSequence(id=None, seq='mkgyfgpyggqyvpeilmga…'),
ProteinSequence(id=None, seq='mkgyfgpyggqyvpeilmga…'),
ProteinSequence(id=None, seq='mkgyfgpyggqyvpeilmga…'),
ProteinSequence(id=None, seq='mkgyfgpyggqyvpeilmga…'),
ProteinSequence(id=None, seq='mkgyfgpyggqyvpeilmga…'),
ProteinSequence(id=None, seq='mkgyfgpyggqyvpeilmga…'),
ProteinSequence(id=None, seq='mkgyfgpyggqyvpeilmga…'),
ProteinSequence(id=None, seq='mkgyfgpyggqyvpeilmga…'),
ProteinSequence(id=None, seq='mkgyfgpyggqyvpeilmga…'),
ProteinSequence(id=None, seq='mkgyfgpyggqyvpeilmga…'),
ProteinSequence(id=None, seq='mkgyfgpyggqyvpeilmga…'),
ProteinSequence(id=None, seq='mkgyfgpyggqyvpeilmga…'),
ProteinSequence(id=None, seq='mkgyfgpyggqyvpeilmga…'),
ProteinSequence(id=None, seq='mkgyfgpyggqyvpeilmga…'),
ProteinSequence(id=None, seq='mkgyfgpyggqyvpeilmga…'),
ProteinSequence(id=None, seq='mkgyfgpyggqyvpeilmga…'),
ProteinSequence(id=None, seq='mkgyfgpyggqyvpeilmga…'),
ProteinSequence(id=None, seq='mkgyfgpyggqyvpeilmga…'),
ProteinSequence(id=None, seq='mkgyfgpyggqyvpeilmga…'),
ProteinSequence(id=None, seq='mkgyfgpyggqyvpeilmga…'),
ProteinSequence(id=None, seq='mkgyfgpyggqyvpeilmga…'),
ProteinSequence(id=None, seq='mkgyfgpyggqyvpeilmga…'),
ProteinSequence(id=None, seq='mkgyfgpyggqyvpeilmga…'),
ProteinSequence(id=None, seq='mkgyfgpyggqyvpeilmga…'),
ProteinSequence(id=None, seq='mkgyfgpyggqyvpeilmga…'),
ProteinSequence(id=None, seq='mkgyfgpyggqyvpeilmga…'),
ProteinSequence(id=None, seq='mkgyfgpyggqyvpeilmga…'),
ProteinSequence(id=None, seq='mkgyfgpyggqyvpeilmga…'),
ProteinSequence(id=None, seq='mkgyfgpyggqyvpeilmga…'),
ProteinSequence(id=None, seq='mkgyfgpyggqyvpeilmga…'),
ProteinSequence(id=None, seq='mkgyfgpyggqyvpeilmga…'),
ProteinSequence(id=None, seq='mkgyfgpyggqyvpeilmga…'),
ProteinSequence(id=None, seq='mkgyfgpyggqyvpeilmga…'),
ProteinSequence(id=None, seq='mkgyfgpyggqyvpeilmga…'),
ProteinSequence(id=None, seq='mkgyfgpyggqyvpeilmga…'),
ProteinSequence(id=None, seq='mkgyfgpyggqyvpeilmga…'),
ProteinSequence(id=None, seq='mkgyfgpyggqyvpeilmga…'),
ProteinSequence(id=None, seq='mkgyfgpyggqyvpeilmga…'),
ProteinSequence(id=None, seq='mkgyfgpyggqyvpeilmga…'),
```

```
ProteinSequence(id=None, seq='mkgyfgpyggqyvpeilmga…'),
ProteinSequence(id=None, seq='mkgyfgpyggqyvpeilmga…'),
ProteinSequence(id=None, seq='mkgyfgpyggqyvpeilmga…'),
ProteinSequence(id=None, seq='mkgyfgpyggqyvpeilmga…'),
ProteinSequence(id=None, seq='mkgyfgpyggqyvpeilmga…'),
ProteinSequence(id=None, seq='mkgyfgpyggqyvpeilmga…'),
ProteinSequence(id=None, seq='mkgyfgpyggqyvpeilmga…'),
ProteinSequence(id=None, seq='mkgyfgpyggqyvpeilmga…'),
ProteinSequence(id=None, seq='mkgyfgpyggqyvpeilmga…'),
ProteinSequence(id=None, seq='mkgyfgpyggqyvpeilmga…'),
ProteinSequence(id=None, seq='mkgyfgpyggqyvpeilmga…'),
ProteinSequence(id=None, seq='mkgyfgpyggqyvpeilmga…'),
ProteinSequence(id=None, seq='mkgyfgpyggqyvpeilmga…'),
ProteinSequence(id=None, seq='mkgyfgpyggqyvpeilmga…'),
ProteinSequence(id=None, seq='mkgyfgpyggqyvpeilmga…'),
ProteinSequence(id=None, seq='mkgyfgpyggqyvpeilmga…'),
ProteinSequence(id=None, seq='mkgyfgpyggqyvpeilmga…'),
ProteinSequence(id=None, seq='mkgyfgpyggqyvpeilmga…'),
ProteinSequence(id=None, seq='mkgyfgpyggqyvpeilmga…'),
ProteinSequence(id=None, seq='mkgyfgpyggqyvpeilmga…'),
ProteinSequence(id=None, seq='mkgyfgpyggqyvpeilmga…'),
ProteinSequence(id=None, seq='mkgyfgpyggqyvpeilmga…'),
ProteinSequence(id=None, seq='mkgyfgpyggqyvpeilmga…'),
ProteinSequence(id=None, seq='mkgyfgpyggqyvpeilmga…'),
ProteinSequence(id=None, seq='mkgyfgpyggqyvpeilmga…'),
ProteinSequence(id=None, seq='mkgyfgpyggqyvpeilmga…'),
ProteinSequence(id=None, seq='mkgyfgpyggqyvpeilmga…'),
ProteinSequence(id=None, seq='mkgyfgpyggqyvpeilmga…'),
ProteinSequence(id=None, seq='mkgyfgpyggqyvpeilmga…'),
ProteinSequence(id=None, seq='mkgyfgpyggqyvpeilmga…'),
ProteinSequence(id=None, seq='mkgyfgpyggqyvpeilmga…'),
ProteinSequence(id=None, seq='mkgyfgpyggqyvpeilmga…'),
ProteinSequence(id=None, seq='mkgyfgpyggqyvpeilmga…'),
ProteinSequence(id=None, seq='mkgyfgpyggqyvpeilmga…'),
ProteinSequence(id=None, seq='mkgyfgpyggqyvpeilmga…'),
ProteinSequence(id=None, seq='mkgyfgpyggqyvpeilmga…'),
ProteinSequence(id=None, seq='mkgyfgpyggqyvpeilmga…'),
ProteinSequence(id=None, seq='mkgyfgpyggqyvpeilmga…'),
ProteinSequence(id=None, seq='mkgyfgpyggqyvpeilmga…'),
ProteinSequence(id=None, seq='mkgyfgpyggqyvpeilmga…'),
ProteinSequence(id=None, seq='mkgyfgpyggqyvpeilmga…'),
ProteinSequence(id=None, seq='mkgyfgpyggqyvpeilmga…'),
ProteinSequence(id=None, seq='mkgyfgpyggqyvpeilmga…'),
ProteinSequence(id=None, seq='mkgyfgpyggqyvpeilmga…'),
ProteinSequence(id=None, seq='mkgyfgpyggqyvpeilmga…'),
ProteinSequence(id=None, seq='mkgyfgpyggqyvpeilmga…'),
ProteinSequence(id=None, seq='mkgyfgpyggqyvpeilmga…'),
ProteinSequence(id=None, seq='mkgyfgpyggqyvpeilmga…'),
```

```
ProteinSequence(id=None, seq='mkgyfgpyggqyvpeilmga…'),
ProteinSequence(id=None, seq='mkgyfgpyggqyvpeilmga…'),
ProteinSequence(id=None, seq='mkgyfgpyggqyvpeilmga…'),
ProteinSequence(id=None, seq='mkgyfgpyggqyvpeilmga…'),
ProteinSequence(id=None, seq='mkgyfgpyggqyvpeilmga…'),
ProteinSequence(id=None, seq='mkgyfgpyggqyvpeilmga…'),
ProteinSequence(id=None, seq='mkgyfgpyggqyvpeilmga…'),
ProteinSequence(id=None, seq='mkgyfgpyggqyvpeilmga…'),
ProteinSequence(id=None, seq='mkgyfgpyggqyvpeilmga…'),
ProteinSequence(id=None, seq='mkgyfgpyggqyvpeilmga…'),
ProteinSequence(id=None, seq='mkgyfgpyggqyvpeilmga…'),
ProteinSequence(id=None, seq='mkgyfgpyggqyvpeilmga…'),
ProteinSequence(id=None, seq='mkgyfgpyggqyvpeilmga…'),
ProteinSequence(id=None, seq='mkgyfgpyggqyvpeilmga…'),
ProteinSequence(id=None, seq='mkgyfgpyggqyvpeilmga…'),
ProteinSequence(id=None, seq='mkgyfgpyggqyvpeilmga…'),
ProteinSequence(id=None, seq='mkgyfgpyggqyvpeilmga…'),
ProteinSequence(id=None, seq='mkgyfgpyggqyvpeilmga…'),
ProteinSequence(id=None, seq='mkgyfgpyggqyvpeilmga…'),
ProteinSequence(id=None, seq='mkgyfgpyggqyvpeilmga…'),
ProteinSequence(id=None, seq='mkgyfgpyggqyvpeilmga…'),
ProteinSequence(id=None, seq='mkgyfgpyggqyvpeilmga…'),
ProteinSequence(id=None, seq='mkgyfgpyggqyvpeilmga…'),
ProteinSequence(id=None, seq='mkgyfgpyggqyvpeilmga…'),
ProteinSequence(id=None, seq='mkgyfgpyggqyvpeilmga…'),
ProteinSequence(id=None, seq='mkgyfgpyggqyvpeilmga…'),
ProteinSequence(id=None, seq='mkgyfgpyggqyvpeilmga…'),
ProteinSequence(id=None, seq='mkgyfgpyggqyvpeilmga…'),
ProteinSequence(id=None, seq='mkgyfgpyggqyvpeilmga…'),
ProteinSequence(id=None, seq='mkgyfgpyggqyvpeilmga…'),
ProteinSequence(id=None, seq='mkgyfgpyggqyvpeilmga…'),
ProteinSequence(id=None, seq='mkgyfgpyggqyvpeilmga…'),
ProteinSequence(id=None, seq='mkgyfgpyggqyvpeilmga…'),
ProteinSequence(id=None, seq='mkgyfgpyggqyvpeilmga…'),
ProteinSequence(id=None, seq='mkgyfgpyggqyvpeilmga…'),
ProteinSequence(id=None, seq='mkgyfgpyggqyvpeilmga…'),
ProteinSequence(id=None, seq='mkgyfgpyggqyvpeilmga…'),
ProteinSequence(id=None, seq='mkgyfgpyggqyvpeilmga…'),
ProteinSequence(id=None, seq='mkgyfgpyggqyvpeilmga…'),
ProteinSequence(id=None, seq='mkgyfgpyggqyvpeilmga…'),
ProteinSequence(id=None, seq='mkgyfgpyggqyvpeilmga…'),
ProteinSequence(id=None, seq='mkgyfgpyggqyvpeilmga…'),
ProteinSequence(id=None, seq='mkgyfgpyggqyvpeilmga…'),
ProteinSequence(id=None, seq='mkgyfgpyggqyvpeilmga…'),
ProteinSequence(id=None, seq='mkgyfgpyggqyvpeilmga…'),
ProteinSequence(id=None, seq='mkgyfgpyggqyvpeilmga…'),
ProteinSequence(id=None, seq='mkgyfgpyggqyvpeilmga…'),
ProteinSequence(id=None, seq='mkgyfgpyggqyvpeilmga…'),
```

```
ProteinSequence(id=None, seq='mkgyfgpyggqyvpeilmga…'),
ProteinSequence(id=None, seq='mkgyfgpyggqyvpeilmga…'),
ProteinSequence(id=None, seq='mkgyfgpyggqyvpeilmga…'),
ProteinSequence(id=None, seq='mkgyfgpyggqyvpeilmga…'),
ProteinSequence(id=None, seq='mkgyfgpyggqyvpeilmga…'),
ProteinSequence(id=None, seq='mkgyfgpyggqyvpeilmga…'),
ProteinSequence(id=None, seq='mkgyfgpyggqyvpeilmga…'),
ProteinSequence(id=None, seq='mkgyfgpyggqyvpeilmga…'),
ProteinSequence(id=None, seq='mkgyfgpyggqyvpeilmga…'),
ProteinSequence(id=None, seq='mkgyfgpyggqyvpeilmga…'),
ProteinSequence(id=None, seq='mkgyfgpyggqyvpeilmga…'),
ProteinSequence(id=None, seq='mkgyfgpyggqyvpeilmga…'),
ProteinSequence(id=None, seq='mkgyfgpyggqyvpeilmga…'),
ProteinSequence(id=None, seq='mkgyfgpyggqyvpeilmga…'),
ProteinSequence(id=None, seq='mkgyfgpyggqyvpeilmga…'),
ProteinSequence(id=None, seq='mkgyfgpyggqyvpeilmga…'),
ProteinSequence(id=None, seq='mkgyfgpyggqyvpeilmga…'),
ProteinSequence(id=None, seq='mkgyfgpyggqyvpeilmga…'),
ProteinSequence(id=None, seq='mkgyfgpyggqyvpeilmga…'),
ProteinSequence(id=None, seq='mkgyfgpyggqyvpeilmga…'),
ProteinSequence(id=None, seq='mkgyfgpyggqyvpeilmga…'),
ProteinSequence(id=None, seq='mkgyfgpyggqyvpeilmga…'),
ProteinSequence(id=None, seq='mkgyfgpyggqyvpeilmga…'),
ProteinSequence(id=None, seq='mkgyfgpyggqyvpeilmga…'),
ProteinSequence(id=None, seq='mkgyfgpyggqyvpeilmga…'),
ProteinSequence(id=None, seq='mkgyfgpyggqyvpeilmga…'),
ProteinSequence(id=None, seq='mkgyfgpyggqyvpeilmga…'),
ProteinSequence(id=None, seq='mkgyfgpyggqyvpeilmga…'),
ProteinSequence(id=None, seq='mkgyfgpyggqyvpeilmga…'),
ProteinSequence(id=None, seq='mkgyfgpyggqyvpeilmga…'),
ProteinSequence(id=None, seq='mkgyfgpyggqyvpeilmga…'),
ProteinSequence(id=None, seq='mkgyfgpyggqyvpeilmga…'),
ProteinSequence(id=None, seq='mkgyfgpyggqyvpeilmga…'),
ProteinSequence(id=None, seq='mkgyfgpyggqyvpeilmga…'),
ProteinSequence(id=None, seq='mkgyfgpyggqyvpeilmga…'),
ProteinSequence(id=None, seq='mkgyfgpyggqyvpeilmga…'),
ProteinSequence(id=None, seq='mkgyfgpyggqyvpeilmga…'),
ProteinSequence(id=None, seq='mkgyfgpyggqyvpeilmga…'),
ProteinSequence(id=None, seq='mkgyfgpyggqyvpeilmga…'),
ProteinSequence(id=None, seq='mkgyfgpyggqyvpeilmga…'),
ProteinSequence(id=None, seq='mkgyfgpyggqyvpeilmga…'),
ProteinSequence(id=None, seq='mkgyfgpyggqyvpeilmga…'),
ProteinSequence(id=None, seq='mkgyfgpyggqyvpeilmga…'),
ProteinSequence(id=None, seq='mkgyfgpyggqyvpeilmga…'),
ProteinSequence(id=None, seq='mkgyfgpyggqyvpeilmga…'),
ProteinSequence(id=None, seq='mkgyfgpyggqyvpeilmga…'),
ProteinSequence(id=None, seq='mkgyfgpyggqyvpeilmga…'),
ProteinSequence(id=None, seq='mkgyfgpyggqyvpeilmga…'),
```

```
ProteinSequence(id=None, seq='mkgyfgpyggqyvpeilmga…'),
ProteinSequence(id=None, seq='mkgyfgpyggqyvpeilmga…'),
ProteinSequence(id=None, seq='mkgyfgpyggqyvpeilmga…'),
ProteinSequence(id=None, seq='mkgyfgpyggqyvpeilmga…'),
ProteinSequence(id=None, seq='mkgyfgpyggqyvpeilmga…'),
ProteinSequence(id=None, seq='mkgyfgpyggqyvpeilmga…'),
ProteinSequence(id=None, seq='mkgyfgpyggqyvpeilmga…'),
ProteinSequence(id=None, seq='mkgyfgpyggqyvpeilmga…'),
ProteinSequence(id=None, seq='mkgyfgpyggqyvpeilmga…'),
ProteinSequence(id=None, seq='mkgyfgpyggqyvpeilmga…'),
ProteinSequence(id=None, seq='mkgyfgpyggqyvpeilmga…'),
ProteinSequence(id=None, seq='mkgyfgpyggqyvpeilmga…'),
ProteinSequence(id=None, seq='mkgyfgpyggqyvpeilmga…'),
ProteinSequence(id=None, seq='mkgyfgpyggqyvpeilmga…'),
ProteinSequence(id=None, seq='mkgyfgpyggqyvpeilmga…'),
ProteinSequence(id=None, seq='mkgyfgpyggqyvpeilmga…'),
ProteinSequence(id=None, seq='mkgyfgpyggqyvpeilmga…'),
ProteinSequence(id=None, seq='mkgyfgpyggqyvpeilmga…'),
ProteinSequence(id=None, seq='mkgyfgpyggqyvpeilmga…'),
ProteinSequence(id=None, seq='mkgyfgpyggqyvpeilmga…'),
ProteinSequence(id=None, seq='mkgyfgpyggqyvpeilmga…'),
ProteinSequence(id=None, seq='mkgyfgpyggqyvpeilmga…'),
ProteinSequence(id=None, seq='mkgyfgpyggqyvpeilmga…'),
ProteinSequence(id=None, seq='mkgyfgpyggqyvpeilmga…'),
ProteinSequence(id=None, seq='mkgyfgpyggqyvpeilmga…'),
ProteinSequence(id=None, seq='mkgyfgpyggqyvpeilmga…'),
ProteinSequence(id=None, seq='mkgyfgpyggqyvpeilmga…'),
ProteinSequence(id=None, seq='mkgyfgpyggqyvpeilmga…'),
ProteinSequence(id=None, seq='mkgyfgpyggqyvpeilmga…'),
ProteinSequence(id=None, seq='mkgyfgpyggqyvpeilmga…'),
ProteinSequence(id=None, seq='mkgyfgpyggqyvpeilmga…'),
ProteinSequence(id=None, seq='mkgyfgpyggqyvpeilmga…'),
ProteinSequence(id=None, seq='mkgyfgpyggqyvpeilmga…'),
ProteinSequence(id=None, seq='mkgyfgpyggqyvpeilmga…'),
ProteinSequence(id=None, seq='mkgyfgpyggqyvpeilmga…'),
ProteinSequence(id=None, seq='mkgyfgpyggqyvpeilmga…'),
ProteinSequence(id=None, seq='mkgyfgpyggqyvpeilmga…'),
ProteinSequence(id=None, seq='mkgyfgpyggqyvpeilmga…'),
ProteinSequence(id=None, seq='mkgyfgpyggqyvpeilmga…'),
ProteinSequence(id=None, seq='mkgyfgpyggqyvpeilmga…'),
ProteinSequence(id=None, seq='mkgyfgpyggqyvpeilmga…'),
ProteinSequence(id=None, seq='mkgyfgpyggqyvpeilmga…'),
ProteinSequence(id=None, seq='mkgyfgpyggqyvpeilmga…'),
ProteinSequence(id=None, seq='mkgyfgpyggqyvpeilmga…'),
ProteinSequence(id=None, seq='mkgyfgpyggqyvpeilmga…'),
ProteinSequence(id=None, seq='mkgyfgpyggqyvpeilmga…'),
ProteinSequence(id=None, seq='mkgyfgpyggqyvpeilmga…'),
ProteinSequence(id=None, seq='mkgyfgpyggqyvpeilmga…'),
```

```
ProteinSequence(id=None, seq='mkgyfgpyggqyvpeilmga…'),
ProteinSequence(id=None, seq='mkgyfgpyggqyvpeilmga…'),
ProteinSequence(id=None, seq='mkgyfgpyggqyvpeilmga…'),
ProteinSequence(id=None, seq='mkgyfgpyggqyvpeilmga…'),
ProteinSequence(id=None, seq='mkgyfgpyggqyvpeilmga…'),
ProteinSequence(id=None, seq='mkgyfgpyggqyvpeilmga…'),
ProteinSequence(id=None, seq='mkgyfgpyggqyvpeilmga…'),
ProteinSequence(id=None, seq='mkgyfgpyggqyvpeilmga…'),
ProteinSequence(id=None, seq='mkgyfgpyggqyvpeilmga…'),
ProteinSequence(id=None, seq='mkgyfgpyggqyvpeilmga…'),
ProteinSequence(id=None, seq='mkgyfgpyggqyvpeilmga…'),
ProteinSequence(id=None, seq='mkgyfgpyggqyvpeilmga…'),
ProteinSequence(id=None, seq='mkgyfgpyggqyvpeilmga…'),
ProteinSequence(id=None, seq='mkgyfgpyggqyvpeilmga…'),
ProteinSequence(id=None, seq='mkgyfgpyggqyvpeilmga…'),
ProteinSequence(id=None, seq='mkgyfgpyggqyvpeilmga…'),
ProteinSequence(id=None, seq='mkgyfgpyggqyvpeilmga…'),
ProteinSequence(id=None, seq='mkgyfgpyggqyvpeilmga…'),
ProteinSequence(id=None, seq='mkgyfgpyggqyvpeilmga…'),
ProteinSequence(id=None, seq='mkgyfgpyggqyvpeilmga…'),
ProteinSequence(id=None, seq='mkgyfgpyggqyvpeilmga…'),
ProteinSequence(id=None, seq='mkgyfgpyggqyvpeilmga…'),
ProteinSequence(id=None, seq='mkgyfgpyggqyvpeilmga…'),
ProteinSequence(id=None, seq='mkgyfgpyggqyvpeilmga…'),
ProteinSequence(id=None, seq='mkgyfgpyggqyvpeilmga…'),
ProteinSequence(id=None, seq='mkgyfgpyggqyvpeilmga…'),
ProteinSequence(id=None, seq='mkgyfgpyggqyvpeilmga…'),
ProteinSequence(id=None, seq='mkgyfgpyggqyvpeilmga…'),
ProteinSequence(id=None, seq='mkgyfgpyggqyvpeilmga…'),
ProteinSequence(id=None, seq='mkgyfgpyggqyvpeilmga…'),
ProteinSequence(id=None, seq='mkgyfgpyggqyvpeilmga…'),
ProteinSequence(id=None, seq='mkgyfgpyggqyvpeilmga…'),
ProteinSequence(id=None, seq='mkgyfgpyggqyvpeilmga…'),
ProteinSequence(id=None, seq='mkgyfgpyggqyvpeilmga…'),
ProteinSequence(id=None, seq='mkgyfgpyggqyvpeilmga…'),
ProteinSequence(id=None, seq='mkgyfgpyggqyvpeilmga…'),
ProteinSequence(id=None, seq='mkgyfgpyggqyvpeilmga…'),
ProteinSequence(id=None, seq='mkgyfgpyggqyvpeilmga…'),
ProteinSequence(id=None, seq='mkgyfgpyggqyvpeilmga…'),
ProteinSequence(id=None, seq='mkgyfgpyggqyvpeilmga…'),
ProteinSequence(id=None, seq='mkgyfgpyggqyvpeilmga…'),
ProteinSequence(id=None, seq='mkgyfgpyggqyvpeilmga…'),
ProteinSequence(id=None, seq='mkgyfgpyggqyvpeilmga…'),
ProteinSequence(id=None, seq='mkgyfgpyggqyvpeilmga…'),
ProteinSequence(id=None, seq='mkgyfgpyggqyvpeilmga…'),
ProteinSequence(id=None, seq='mkgyfgpyggqyvpeilmga…'),
ProteinSequence(id=None, seq='mkgyfgpyggqyvpeilmga…'),
ProteinSequence(id=None, seq='mkgyfgpyggqyvpeilmga…'),
```

```
ProteinSequence(id=None, seq='mkgyfgpyggqyvpeilmga…'),
ProteinSequence(id=None, seq='mkgyfgpyggqyvpeilmga…'),
ProteinSequence(id=None, seq='mkgyfgpyggqyvpeilmga…'),
ProteinSequence(id=None, seq='mkgyfgpyggqyvpeilmga…'),
ProteinSequence(id=None, seq='mkgyfgpyggqyvpeilmga…'),
ProteinSequence(id=None, seq='mkgyfgpyggqyvpeilmga…'),
ProteinSequence(id=None, seq='mkgyfgpyggqyvpeilmga…'),
ProteinSequence(id=None, seq='mkgyfgpyggqyvpeilmga…'),
ProteinSequence(id=None, seq='mkgyfgpyggqyvpeilmga…'),
ProteinSequence(id=None, seq='mkgyfgpyggqyvpeilmga…'),
ProteinSequence(id=None, seq='mkgyfgpyggqyvpeilmga…'),
ProteinSequence(id=None, seq='mkgyfgpyggqyvpeilmga…'),
ProteinSequence(id=None, seq='mkgyfgpyggqyvpeilmga…'),
ProteinSequence(id=None, seq='mkgyfgpyggqyvpeilmga…'),
ProteinSequence(id=None, seq='mkgyfgpyggqyvpeilmga…'),
ProteinSequence(id=None, seq='mkgyfgpyggqyvpeilmga…'),
ProteinSequence(id=None, seq='mkgyfgpyggqyvpeilmga…'),
ProteinSequence(id=None, seq='mkgyfgpyggqyvpeilmga…'),
ProteinSequence(id=None, seq='mkgyfgpyggqyvpeilmga…'),
ProteinSequence(id=None, seq='mkgyfgpyggqyvpeilmga…'),
ProteinSequence(id=None, seq='mkgyfgpyggqyvpeilmga…'),
ProteinSequence(id=None, seq='mkgyfgpyggqyvpeilmga…'),
ProteinSequence(id=None, seq='mkgyfgpyggqyvpeilmga…'),
ProteinSequence(id=None, seq='mkgyfgpyggqyvpeilmga…'),
ProteinSequence(id=None, seq='mkgyfgpyggqyvpeilmga…'),
ProteinSequence(id=None, seq='mkgyfgpyggqyvpeilmga…'),
ProteinSequence(id=None, seq='mkgyfgpyggqyvpeilmga…'),
ProteinSequence(id=None, seq='mkgyfgpyggqyvpeilmga…'),
ProteinSequence(id=None, seq='mkgyfgpyggqyvpeilmga…'),
ProteinSequence(id=None, seq='mkgyfgpyggqyvpeilmga…'),
ProteinSequence(id=None, seq='mkgyfgpyggqyvpeilmga…'),
ProteinSequence(id=None, seq='mkgyfgpyggqyvpeilmga…'),
ProteinSequence(id=None, seq='mkgyfgpyggqyvpeilmga…'),
ProteinSequence(id=None, seq='mkgyfgpyggqyvpeilmga…'),
ProteinSequence(id=None, seq='mkgyfgpyggqyvpeilmga…'),
ProteinSequence(id=None, seq='mkgyfgpyggqyvpeilmga…'),
ProteinSequence(id=None, seq='mkgyfgpyggqyvpeilmga…'),
ProteinSequence(id=None, seq='mkgyfgpyggqyvpeilmga…'),
ProteinSequence(id=None, seq='mkgyfgpyggqyvpeilmga…'),
ProteinSequence(id=None, seq='mkgyfgpyggqyvpeilmga…'),
ProteinSequence(id=None, seq='mkgyfgpyggqyvpeilmga…'),
ProteinSequence(id=None, seq='mkgyfgpyggqyvpeilmga…'),
ProteinSequence(id=None, seq='mkgyfgpyggqyvpeilmga…'),
ProteinSequence(id=None, seq='mkgyfgpyggqyvpeilmga…'),
ProteinSequence(id=None, seq='mkgyfgpyggqyvpeilmga…'),
ProteinSequence(id=None, seq='mkgyfgpyggqyvpeilmga…'),
ProteinSequence(id=None, seq='mkgyfgpyggqyvpeilmga…'),
ProteinSequence(id=None, seq='mkgyfgpyggqyvpeilmga…'),
```

```
ProteinSequence(id=None, seq='mkgyfgpyggqyvpeilmga…'),
ProteinSequence(id=None, seq='mkgyfgpyggqyvpeilmga…'),
ProteinSequence(id=None, seq='mkgyfgpyggqyvpeilmga…'),
ProteinSequence(id=None, seq='mkgyfgpyggqyvpeilmga…'),
ProteinSequence(id=None, seq='mkgyfgpyggqyvpeilmga…'),
ProteinSequence(id=None, seq='mkgyfgpyggqyvpeilmga…'),
ProteinSequence(id=None, seq='mkgyfgpyggqyvpeilmga…'),
ProteinSequence(id=None, seq='mkgyfgpyggqyvpeilmga…'),
ProteinSequence(id=None, seq='mkgyfgpyggqyvpeilmga…'),
ProteinSequence(id=None, seq='mkgyfgpyggqyvpeilmga…'),
ProteinSequence(id=None, seq='mkgyfgpyggqyvpeilmga…'),
ProteinSequence(id=None, seq='mkgyfgpyggqyvpeilmga…'),
ProteinSequence(id=None, seq='mkgyfgpyggqyvpeilmga…'),
ProteinSequence(id=None, seq='mkgyfgpyggqyvpeilmga…'),
ProteinSequence(id=None, seq='mkgyfgpyggqyvpeilmga…'),
ProteinSequence(id=None, seq='mkgyfgpyggqyvpeilmga…'),
ProteinSequence(id=None, seq='mkgyfgpyggqyvpeilmga…'),
ProteinSequence(id=None, seq='mkgyfgpyggqyvpeilmga…'),
ProteinSequence(id=None, seq='mkgyfgpyggqyvpeilmga…'),
ProteinSequence(id=None, seq='mkgyfgpyggqyvpeilmga…'),
ProteinSequence(id=None, seq='mkgyfgpyggqyvpeilmga…'),
ProteinSequence(id=None, seq='mkgyfgpyggqyvpeilmga…'),
ProteinSequence(id=None, seq='mkgyfgpyggqyvpeilmga…'),
ProteinSequence(id=None, seq='mkgyfgpyggqyvpeilmga…'),
ProteinSequence(id=None, seq='mkgyfgpyggqyvpeilmga…'),
ProteinSequence(id=None, seq='mkgyfgpyggqyvpeilmga…'),
ProteinSequence(id=None, seq='mkgyfgpyggqyvpeilmga…'),
ProteinSequence(id=None, seq='mkgyfgpyggqyvpeilmga…'),
ProteinSequence(id=None, seq='mkgyfgpyggqyvpeilmga…'),
ProteinSequence(id=None, seq='mkgyfgpyggqyvpeilmga…'),
ProteinSequence(id=None, seq='mkgyfgpyggqyvpeilmga…'),
ProteinSequence(id=None, seq='mkgyfgpyggqyvpeilmga…'),
ProteinSequence(id=None, seq='mkgyfgpyggqyvpeilmga…'),
ProteinSequence(id=None, seq='mkgyfgpyggqyvpeilmga…'),
ProteinSequence(id=None, seq='mkgyfgpyggqyvpeilmga…'),
ProteinSequence(id=None, seq='mkgyfgpyggqyvpeilmga…'),
ProteinSequence(id=None, seq='mkgyfgpyggqyvpeilmga…'),
ProteinSequence(id=None, seq='mkgyfgpyggqyvpeilmga…'),
ProteinSequence(id=None, seq='mkgyfgpyggqyvpeilmga…'),
ProteinSequence(id=None, seq='mkgyfgpyggqyvpeilmga…'),
ProteinSequence(id=None, seq='mkgyfgpyggqyvpeilmga…'),
ProteinSequence(id=None, seq='mkgyfgpyggqyvpeilmga…'),
ProteinSequence(id=None, seq='mkgyfgpyggqyvpeilmga…'),
ProteinSequence(id=None, seq='mkgyfgpyggqyvpeilmga…'),
ProteinSequence(id=None, seq='mkgyfgpyggqyvpeilmga…'),
ProteinSequence(id=None, seq='mkgyfgpyggqyvpeilmga…'),
ProteinSequence(id=None, seq='mkgyfgpyggqyvpeilmga…'),
ProteinSequence(id=None, seq='mkgyfgpyggqyvpeilmga…'),
```

```
ProteinSequence(id=None, seq='mkgyfgpyggqyvpeilmga…'),
ProteinSequence(id=None, seq='mkgyfgpyggqyvpeilmga…'),
ProteinSequence(id=None, seq='mkgyfgpyggqyvpeilmga…'),
ProteinSequence(id=None, seq='mkgyfgpyggqyvpeilmga…'),
ProteinSequence(id=None, seq='mkgyfgpyggqyvpeilmga…'),
ProteinSequence(id=None, seq='mkgyfgpyggqyvpeilmga…'),
ProteinSequence(id=None, seq='mkgyfgpyggqyvpeilmga…'),
ProteinSequence(id=None, seq='mkgyfgpyggqyvpeilmga…'),
ProteinSequence(id=None, seq='mkgyfgpyggqyvpeilmga…'),
ProteinSequence(id=None, seq='mkgyfgpyggqyvpeilmga…'),
ProteinSequence(id=None, seq='mkgyfgpyggqyvpeilmga…'),
ProteinSequence(id=None, seq='mkgyfgpyggqyvpeilmga…'),
ProteinSequence(id=None, seq='mkgyfgpyggqyvpeilmga…'),
ProteinSequence(id=None, seq='mkgyfgpyggqyvpeilmga…'),
ProteinSequence(id=None, seq='mkgyfgpyggqyvpeilmga…'),
ProteinSequence(id=None, seq='mkgyfgpyggqyvpeilmga…'),
ProteinSequence(id=None, seq='mkgyfgpyggqyvpeilmga…'),
ProteinSequence(id=None, seq='mkgyfgpyggqyvpeilmga…'),
ProteinSequence(id=None, seq='mkgyfgpyggqyvpeilmga…'),
ProteinSequence(id=None, seq='mkgyfgpyggqyvpeilmga…'),
ProteinSequence(id=None, seq='mkgyfgpyggqyvpeilmga…'),
ProteinSequence(id=None, seq='mkgyfgpyggqyvpeilmga…'),
ProteinSequence(id=None, seq='mkgyfgpyggqyvpeilmga…'),
ProteinSequence(id=None, seq='mkgyfgpyggqyvpeilmga…'),
ProteinSequence(id=None, seq='mkgyfgpyggqyvpeilmga…'),
ProteinSequence(id=None, seq='mkgyfgpyggqyvpeilmga…'),
ProteinSequence(id=None, seq='mkgyfgpyggqyvpeilmga…'),
ProteinSequence(id=None, seq='mkgyfgpyggqyvpeilmga…'),
ProteinSequence(id=None, seq='mkgyfgpyggqyvpeilmga…'),
ProteinSequence(id=None, seq='mkgyfgpyggqyvpeilmga…'),
ProteinSequence(id=None, seq='mkgyfgpyggqyvpeilmga…'),
ProteinSequence(id=None, seq='mkgyfgpyggqyvpeilmga…'),
ProteinSequence(id=None, seq='mkgyfgpyggqyvpeilmga…'),
ProteinSequence(id=None, seq='mkgyfgpyggqyvpeilmga…'),
ProteinSequence(id=None, seq='mkgyfgpyggqyvpeilmga…'),
ProteinSequence(id=None, seq='mkgyfgpyggqyvpeilmga…'),
ProteinSequence(id=None, seq='mkgyfgpyggqyvpeilmga…'),
ProteinSequence(id=None, seq='mkgyfgpyggqyvpeilmga…'),
ProteinSequence(id=None, seq='mkgyfgpyggqyvpeilmga…'),
ProteinSequence(id=None, seq='mkgyfgpyggqyvpeilmga…'),
ProteinSequence(id=None, seq='mkgyfgpyggqyvpeilmga…'),
ProteinSequence(id=None, seq='mkgyfgpyggqyvpeilmga…'),
ProteinSequence(id=None, seq='mkgyfgpyggqyvpeilmga…'),
ProteinSequence(id=None, seq='mkgyfgpyggqyvpeilmga…'),
ProteinSequence(id=None, seq='mkgyfgpyggqyvpeilmga…'),
ProteinSequence(id=None, seq='mkgyfgpyggqyvpeilmga…'),
ProteinSequence(id=None, seq='mkgyfgpyggqyvpeilmga…'),
ProteinSequence(id=None, seq='mkgyfgpyggqyvpeilmga…'),
```

```
ProteinSequence(id=None, seq='mkgyfgpyggqyvpeilmga…'),
ProteinSequence(id=None, seq='mkgyfgpyggqyvpeilmga…'),
ProteinSequence(id=None, seq='mkgyfgpyggqyvpeilmga…'),
ProteinSequence(id=None, seq='mkgyfgpyggqyvpeilmga…'),
ProteinSequence(id=None, seq='mkgyfgpyggqyvpeilmga…'),
ProteinSequence(id=None, seq='mkgyfgpyggqyvpeilmga…'),
ProteinSequence(id=None, seq='mkgyfgpyggqyvpeilmga…'),
ProteinSequence(id=None, seq='mkgyfgpyggqyvpeilmga…'),
ProteinSequence(id=None, seq='mkgyfgpyggqyvpeilmga…'),
ProteinSequence(id=None, seq='mkgyfgpyggqyvpeilmga…'),
ProteinSequence(id=None, seq='mkgyfgpyggqyvpeilmga…'),
ProteinSequence(id=None, seq='mkgyfgpyggqyvpeilmga…'),
ProteinSequence(id=None, seq='mkgyfgpyggqyvpeilmga…'),
ProteinSequence(id=None, seq='mkgyfgpyggqyvpeilmga…'),
ProteinSequence(id=None, seq='mkgyfgpyggqyvpeilmga…'),
ProteinSequence(id=None, seq='mkgyfgpyggqyvpeilmga…'),
ProteinSequence(id=None, seq='mkgyfgpyggqyvpeilmga…'),
ProteinSequence(id=None, seq='mkgyfgpyggqyvpeilmga…'),
ProteinSequence(id=None, seq='mkgyfgpyggqyvpeilmga…'),
ProteinSequence(id=None, seq='mkgyfgpyggqyvpeilmga…'),
ProteinSequence(id=None, seq='mkgyfgpyggqyvpeilmga…'),
ProteinSequence(id=None, seq='mkgyfgpyggqyvpeilmga…'),
ProteinSequence(id=None, seq='mkgyfgpyggqyvpeilmga…'),
ProteinSequence(id=None, seq='mkgyfgpyggqyvpeilmga…'),
ProteinSequence(id=None, seq='mkgyfgpyggqyvpeilmga…'),
ProteinSequence(id=None, seq='mkgyfgpyggqyvpeilmga…'),
ProteinSequence(id=None, seq='mkgyfgpyggqyvpeilmga…'),
ProteinSequence(id=None, seq='mkgyfgpyggqyvpeilmga…'),
ProteinSequence(id=None, seq='mkgyfgpyggqyvpeilmga…'),
ProteinSequence(id=None, seq='mkgyfgpyggqyvpeilmga…'),
ProteinSequence(id=None, seq='mkgyfgpyggqyvpeilmga…'),
ProteinSequence(id=None, seq='mkgyfgpyggqyvpeilmga…'),
ProteinSequence(id=None, seq='mkgyfgpyggqyvpeilmga…'),
ProteinSequence(id=None, seq='mkgyfgpyggqyvpeilmga…'),
ProteinSequence(id=None, seq='mkgyfgpyggqyvpeilmga…'),
ProteinSequence(id=None, seq='mkgyfgpyggqyvpeilmga…'),
ProteinSequence(id=None, seq='mkgyfgpyggqyvpeilmga…'),
ProteinSequence(id=None, seq='mkgyfgpyggqyvpeilmga…'),
ProteinSequence(id=None, seq='mkgyfgpyggqyvpeilmga…'),
ProteinSequence(id=None, seq='mkgyfgpyggqyvpeilmga…'),
ProteinSequence(id=None, seq='mkgyfgpyggqyvpeilmga…'),
ProteinSequence(id=None, seq='mkgyfgpyggqyvpeilmga…'),
ProteinSequence(id=None, seq='mkgyfgpyggqyvpeilmga…'),
ProteinSequence(id=None, seq='mkgyfgpyggqyvpeilmga…'),
ProteinSequence(id=None, seq='mkgyfgpyggqyvpeilmga…'),
ProteinSequence(id=None, seq='mkgyfgpyggqyvpeilmga…'),
ProteinSequence(id=None, seq='mkgyfgpyggqyvpeilmga…'),
ProteinSequence(id=None, seq='mkgyfgpyggqyvpeilmga…'),
```

```
ProteinSequence(id=None, seq='mkgyfgpyggqyvpeilmga…'),
ProteinSequence(id=None, seq='mkgyfgpyggqyvpeilmga…'),
ProteinSequence(id=None, seq='mkgyfgpyggqyvpeilmga…'),
ProteinSequence(id=None, seq='mkgyfgpyggqyvpeilmga…'),
ProteinSequence(id=None, seq='mkgyfgpyggqyvpeilmga…'),
ProteinSequence(id=None, seq='mkgyfgpyggqyvpeilmga…'),
ProteinSequence(id=None, seq='mkgyfgpyggqyvpeilmga…'),
ProteinSequence(id=None, seq='mkgyfgpyggqyvpeilmga…'),
ProteinSequence(id=None, seq='mkgyfgpyggqyvpeilmga…'),
ProteinSequence(id=None, seq='mkgyfgpyggqyvpeilmga…'),
ProteinSequence(id=None, seq='mkgyfgpyggqyvpeilmga…'),
ProteinSequence(id=None, seq='mkgyfgpyggqyvpeilmga…'),
ProteinSequence(id=None, seq='mkgyfgpyggqyvpeilmga…'),
ProteinSequence(id=None, seq='mkgyfgpyggqyvpeilmga…'),
ProteinSequence(id=None, seq='mkgyfgpyggqyvpeilmga…'),
ProteinSequence(id=None, seq='mkgyfgpyggqyvpeilmga…'),
ProteinSequence(id=None, seq='mkgyfgpyggqyvpeilmga…'),
ProteinSequence(id=None, seq='mkgyfgpyggqyvpeilmga…'),
ProteinSequence(id=None, seq='mkgyfgpyggqyvpeilmga…'),
ProteinSequence(id=None, seq='mkgyfgpyggqyvpeilmga…'),
ProteinSequence(id=None, seq='mkgyfgpyggqyvpeilmga…'),
ProteinSequence(id=None, seq='mkgyfgpyggqyvpeilmga…'),
ProteinSequence(id=None, seq='mkgyfgpyggqyvpeilmga…'),
ProteinSequence(id=None, seq='mkgyfgpyggqyvpeilmga…'),
ProteinSequence(id=None, seq='mkgyfgpyggqyvpeilmga…'),
ProteinSequence(id=None, seq='mkgyfgpyggqyvpeilmga…'),
ProteinSequence(id=None, seq='mkgyfgpyggqyvpeilmga…'),
ProteinSequence(id=None, seq='mkgyfgpyggqyvpeilmga…'),
ProteinSequence(id=None, seq='mkgyfgpyggqyvpeilmga…'),
ProteinSequence(id=None, seq='mkgyfgpyggqyvpeilmga…'),
ProteinSequence(id=None, seq='mkgyfgpyggqyvpeilmga…'),
ProteinSequence(id=None, seq='mkgyfgpyggqyvpeilmga…'),
ProteinSequence(id=None, seq='mkgyfgpyggqyvpeilmga…'),
ProteinSequence(id=None, seq='mkgyfgpyggqyvpeilmga…'),
ProteinSequence(id=None, seq='mkgyfgpyggqyvpeilmga…'),
ProteinSequence(id=None, seq='mkgyfgpyggqyvpeilmga…'),
ProteinSequence(id=None, seq='mkgyfgpyggqyvpeilmga…'),
ProteinSequence(id=None, seq='mkgyfgpyggqyvpeilmga…'),
ProteinSequence(id=None, seq='mkgyfgpyggqyvpeilmga…'),
ProteinSequence(id=None, seq='mkgyfgpyggqyvpeilmga…'),
ProteinSequence(id=None, seq='mkgyfgpyggqyvpeilmga…'),
ProteinSequence(id=None, seq='mkgyfgpyggqyvpeilmga…'),
ProteinSequence(id=None, seq='mkgyfgpyggqyvpeilmga…'),
ProteinSequence(id=None, seq='mkgyfgpyggqyvpeilmga…'),
ProteinSequence(id=None, seq='mkgyfgpyggqyvpeilmga…'),
ProteinSequence(id=None, seq='mkgyfgpyggqyvpeilmga…'),
ProteinSequence(id=None, seq='mkgyfgpyggqyvpeilmga…'),
ProteinSequence(id=None, seq='mkgyfgpyggqyvpeilmga…'),
```

```
ProteinSequence(id=None, seq='mkgyfgpyggqyvpeilmga…'),
ProteinSequence(id=None, seq='mkgyfgpyggqyvpeilmga…'),
ProteinSequence(id=None, seq='mkgyfgpyggqyvpeilmga…'),
ProteinSequence(id=None, seq='mkgyfgpyggqyvpeilmga…'),
ProteinSequence(id=None, seq='mkgyfgpyggqyvpeilmga…'),
ProteinSequence(id=None, seq='mkgyfgpyggqyvpeilmga…'),
ProteinSequence(id=None, seq='mkgyfgpyggqyvpeilmga…'),
ProteinSequence(id=None, seq='mkgyfgpyggqyvpeilmga…'),
ProteinSequence(id=None, seq='mkgyfgpyggqyvpeilmga…'),
ProteinSequence(id=None, seq='mkgyfgpyggqyvpeilmga…'),
ProteinSequence(id=None, seq='mkgyfgpyggqyvpeilmga…'),
ProteinSequence(id=None, seq='mkgyfgpyggqyvpeilmga…'),
ProteinSequence(id=None, seq='mkgyfgpyggqyvpeilmga…'),
ProteinSequence(id=None, seq='mkgyfgpyggqyvpeilmga…'),
ProteinSequence(id=None, seq='mkgyfgpyggqyvpeilmga…'),
ProteinSequence(id=None, seq='mkgyfgpyggqyvpeilmga…'),
ProteinSequence(id=None, seq='mkgyfgpyggqyvpeilmga…'),
ProteinSequence(id=None, seq='mkgyfgpyggqyvpeilmga…'),
ProteinSequence(id=None, seq='mkgyfgpyggqyvpeilmga…'),
ProteinSequence(id=None, seq='mkgyfgpyggqyvpeilmga…'),
ProteinSequence(id=None, seq='mkgyfgpyggqyvpeilmga…'),
ProteinSequence(id=None, seq='mkgyfgpyggqyvpeilmga…'),
ProteinSequence(id=None, seq='mkgyfgpyggqyvpeilmga…'),
ProteinSequence(id=None, seq='mkgyfgpyggqyvpeilmga…'),
ProteinSequence(id=None, seq='mkgyfgpyggqyvpeilmga…'),
ProteinSequence(id=None, seq='mkgyfgpyggqyvpeilmga…'),
ProteinSequence(id=None, seq='mkgyfgpyggqyvpeilmga…'),
ProteinSequence(id=None, seq='mkgyfgpyggqyvpeilmga…'),
ProteinSequence(id=None, seq='mkgyfgpyggqyvpeilmga…'),
ProteinSequence(id=None, seq='mkgyfgpyggqyvpeilmga…'),
ProteinSequence(id=None, seq='mkgyfgpyggqyvpeilmga…'),
ProteinSequence(id=None, seq='mkgyfgpyggqyvpeilmga…'),
ProteinSequence(id=None, seq='mkgyfgpyggqyvpeilmga…'),
ProteinSequence(id=None, seq='mkgyfgpyggqyvpeilmga…'),
ProteinSequence(id=None, seq='mkgyfgpyggqyvpeilmga…'),
ProteinSequence(id=None, seq='mkgyfgpyggqyvpeilmga…'),
ProteinSequence(id=None, seq='mkgyfgpyggqyvpeilmga…'),
ProteinSequence(id=None, seq='mkgyfgpyggqyvpeilmga…'),
ProteinSequence(id=None, seq='mkgyfgpyggqyvpeilmga…'),
ProteinSequence(id=None, seq='mkgyfgpyggqyvpeilmga…'),
ProteinSequence(id=None, seq='mkgyfgpyggqyvpeilmga…'),
ProteinSequence(id=None, seq='mkgyfgpyggqyvpeilmga…'),
ProteinSequence(id=None, seq='mkgyfgpyggqyvpeilmga…'),
ProteinSequence(id=None, seq='mkgyfgpyggqyvpeilmga…'),
ProteinSequence(id=None, seq='mkgyfgpyggqyvpeilmga…'),
ProteinSequence(id=None, seq='mkgyfgpyggqyvpeilmga…'),
ProteinSequence(id=None, seq='mkgyfgpyggqyvpeilmga…'),
ProteinSequence(id=None, seq='mkgyfgpyggqyvpeilmga…'),
```

```
ProteinSequence(id=None, seq='mkgyfgpyggqyvpeilmga…'),
ProteinSequence(id=None, seq='mkgyfgpyggqyvpeilmga…'),
ProteinSequence(id=None, seq='mkgyfgpyggqyvpeilmga…'),
ProteinSequence(id=None, seq='mkgyfgpyggqyvpeilmga…'),
ProteinSequence(id=None, seq='mkgyfgpyggqyvpeilmga…'),
ProteinSequence(id=None, seq='mkgyfgpyggqyvpeilmga…'),
ProteinSequence(id=None, seq='mkgyfgpyggqyvpeilmga…'),
ProteinSequence(id=None, seq='mkgyfgpyggqyvpeilmga…'),
ProteinSequence(id=None, seq='mkgyfgpyggqyvpeilmga…'),
ProteinSequence(id=None, seq='mkgyfgpyggqyvpeilmga…'),
ProteinSequence(id=None, seq='mkgyfgpyggqyvpeilmga…'),
ProteinSequence(id=None, seq='mkgyfgpyggqyvpeilmga…'),
ProteinSequence(id=None, seq='mkgyfgpyggqyvpeilmga…'),
ProteinSequence(id=None, seq='mkgyfgpyggqyvpeilmga…'),
ProteinSequence(id=None, seq='mkgyfgpyggqyvpeilmga…'),
ProteinSequence(id=None, seq='mkgyfgpyggqyvpeilmga…'),
ProteinSequence(id=None, seq='mkgyfgpyggqyvpeilmga…'),
ProteinSequence(id=None, seq='mkgyfgpyggqyvpeilmga…'),
ProteinSequence(id=None, seq='mkgyfgpyggqyvpeilmga…'),
ProteinSequence(id=None, seq='mkgyfgpyggqyvpeilmga…'),
ProteinSequence(id=None, seq='mkgyfgpyggqyvpeilmga…'),
ProteinSequence(id=None, seq='mkgyfgpyggqyvpeilmga…'),
ProteinSequence(id=None, seq='mkgyfgpyggqyvpeilmga…'),
ProteinSequence(id=None, seq='mkgyfgpyggqyvpeilmga…'),
ProteinSequence(id=None, seq='mkgyfgpyggqyvpeilmga…'),
ProteinSequence(id=None, seq='mkgyfgpyggqyvpeilmga…'),
ProteinSequence(id=None, seq='mkgyfgpyggqyvpeilmga…'),
ProteinSequence(id=None, seq='mkgyfgpyggqyvpeilmga…'),
ProteinSequence(id=None, seq='mkgyfgpyggqyvpeilmga…'),
ProteinSequence(id=None, seq='mkgyfgpyggqyvpeilmga…'),
ProteinSequence(id=None, seq='mkgyfgpyggqyvpeilmga…'),
ProteinSequence(id=None, seq='mkgyfgpyggqyvpeilmga…'),
ProteinSequence(id=None, seq='mkgyfgpyggqyvpeilmga…'),
ProteinSequence(id=None, seq='mkgyfgpyggqyvpeilmga…'),
ProteinSequence(id=None, seq='mkgyfgpyggqyvpeilmga…'),
ProteinSequence(id=None, seq='mkgyfgpyggqyvpeilmga…'),
ProteinSequence(id=None, seq='mkgyfgpyggqyvpeilmga…'),
ProteinSequence(id=None, seq='mkgyfgpyggqyvpeilmga…'),
ProteinSequence(id=None, seq='mkgyfgpyggqyvpeilmga…'),
ProteinSequence(id=None, seq='mkgyfgpyggqyvpeilmga…'),
ProteinSequence(id=None, seq='mkgyfgpyggqyvpeilmga…'),
ProteinSequence(id=None, seq='mkgyfgpyggqyvpeilmga…'),
ProteinSequence(id=None, seq='mkgyfgpyggqyvpeilmga…'),
ProteinSequence(id=None, seq='mkgyfgpyggqyvpeilmga…'),
ProteinSequence(id=None, seq='mkgyfgpyggqyvpeilmga…'),
ProteinSequence(id=None, seq='mkgyfgpyggqyvpeilmga…'),
ProteinSequence(id=None, seq='mkgyfgpyggqyvpeilmga…'),
ProteinSequence(id=None, seq='mkgyfgpyggqyvpeilmga…'),
```

```
ProteinSequence(id=None, seq='mkgyfgpyggqyvpeilmga…'),
ProteinSequence(id=None, seq='mkgyfgpyggqyvpeilmga…'),
ProteinSequence(id=None, seq='mkgyfgpyggqyvpeilmga…'),
ProteinSequence(id=None, seq='mkgyfgpyggqyvpeilmga…'),
ProteinSequence(id=None, seq='mkgyfgpyggqyvpeilmga…'),
ProteinSequence(id=None, seq='mkgyfgpyggqyvpeilmga…'),
ProteinSequence(id=None, seq='mkgyfgpyggqyvpeilmga…'),
ProteinSequence(id=None, seq='mkgyfgpyggqyvpeilmga…'),
ProteinSequence(id=None, seq='mkgyfgpyggqyvpeilmga…'),
ProteinSequence(id=None, seq='mkgyfgpyggqyvpeilmga…'),
ProteinSequence(id=None, seq='mkgyfgpyggqyvpeilmga…'),
ProteinSequence(id=None, seq='mkgyfgpyggqyvpeilmga…'),
ProteinSequence(id=None, seq='mkgyfgpyggqyvpeilmga…'),
ProteinSequence(id=None, seq='mkgyfgpyggqyvpeilmga…'),
ProteinSequence(id=None, seq='mkgyfgpyggqyvpeilmga…'),
ProteinSequence(id=None, seq='mkgyfgpyggqyvpeilmga…'),
ProteinSequence(id=None, seq='mkgyfgpyggqyvpeilmga…'),
ProteinSequence(id=None, seq='mkgyfgpyggqyvpeilmga…'),
ProteinSequence(id=None, seq='mkgyfgpyggqyvpeilmga…'),
ProteinSequence(id=None, seq='mkgyfgpyggqyvpeilmga…'),
ProteinSequence(id=None, seq='mkgyfgpyggqyvpeilmga…'),
ProteinSequence(id=None, seq='mkgyfgpyggqyvpeilmga…'),
ProteinSequence(id=None, seq='mkgyfgpyggqyvpeilmga…'),
ProteinSequence(id=None, seq='mkgyfgpyggqyvpeilmga…'),
ProteinSequence(id=None, seq='mkgyfgpyggqyvpeilmga…'),
ProteinSequence(id=None, seq='mkgyfgpyggqyvpeilmga…'),
ProteinSequence(id=None, seq='mkgyfgpyggqyvpeilmga…'),
ProteinSequence(id=None, seq='mkgyfgpyggqyvpeilmga…'),
ProteinSequence(id=None, seq='mkgyfgpyggqyvpeilmga…'),
ProteinSequence(id=None, seq='mkgyfgpyggqyvpeilmga…'),
ProteinSequence(id=None, seq='mkgyfgpyggqyvpeilmga…'),
ProteinSequence(id=None, seq='mkgyfgpyggqyvpeilmga…'),
ProteinSequence(id=None, seq='mkgyfgpyggqyvpeilmga…'),
ProteinSequence(id=None, seq='mkgyfgpyggqyvpeilmga…'),
ProteinSequence(id=None, seq='mkgyfgpyggqyvpeilmga…'),
ProteinSequence(id=None, seq='mkgyfgpyggqyvpeilmga…'),
ProteinSequence(id=None, seq='mkgyfgpyggqyvpeilmga…'),
ProteinSequence(id=None, seq='mkgyfgpyggqyvpeilmga…'),
ProteinSequence(id=None, seq='mkgyfgpyggqyvpeilmga…'),
ProteinSequence(id=None, seq='mkgyfgpyggqyvpeilmga…'),
ProteinSequence(id=None, seq='mkgyfgpyggqyvpeilmga…'),
ProteinSequence(id=None, seq='mkgyfgpyggqyvpeilmga…'),
ProteinSequence(id=None, seq='mkgyfgpyggqyvpeilmga…'),
ProteinSequence(id=None, seq='mkgyfgpyggqyvpeilmga…'),
ProteinSequence(id=None, seq='mkgyfgpyggqyvpeilmga…'),
ProteinSequence(id=None, seq='mkgyfgpyggqyvpeilmga…')]
  warnings.warn(f"This model expects no fit, but received input. Ignoring input:
{X}")
```

```
INFO:aide_predict.bespoke_models.base:Transforming input using
MSATransformerLikelihoodWrapper
INFO:aide_predict.bespoke_models.base:Found 0 cached results, running on 127303
uncached sequences.
/kfs2/projects/proteinml/repos/aide_predict/aide_predict/bespoke_models/base.py:
573: UserWarning: Some sequences do not have an MSA, but the wild type sequence
does. Attempting to use the wild type sequence MSA.
  warnings.warn("Some sequences do not have an MSA, but the wild type sequence
does. Attempting to use the wild type sequence MSA.")
MSA batches for sequence 0: 12it [00:04,  2.82it/s]:00<?, ?sequence/s]
Computing log likelihoods: 100%|      | 1/1 [00:04<00:00,  4.41s/sequence]
INFO:aide_predict.bespoke_models.base:Transforming input using
MSATransformerLikelihoodWrapper
INFO:aide_predict.bespoke_models.base:Found 95477 cached results, running on
31826 uncached sequences.
MSA batches for sequence 0: 12it [00:05,  2.10it/s]:00<?, ?sequence/s]
Computing log likelihoods: 100%|      | 1/1 [00:06<00:00,  6.01s/sequence]
INFO:aide_predict.bespoke_models.base:Transforming input using
MSATransformerLikelihoodWrapper
INFO:aide_predict.bespoke_models.base:Found 127303 cached results, running on 0
uncached sequences.
INFO:aide_predict.bespoke_models.base:Transforming input using
MSATransformerLikelihoodWrapper
INFO:aide_predict.bespoke_models.base:Found 127303 cached results, running on 0
uncached sequences.
INFO:aide_predict.bespoke_models.base:Transforming input using
MSATransformerLikelihoodWrapper
INFO:aide_predict.bespoke_models.base:Found 127304 cached results, running on 0
uncached sequences.
```

[49]:
```
joblib.dump(msa_none_none_scores, 'msa_none_none_scores.joblib')
```

[49]:
```
['msa_none_none_scores.joblib']
```

1.5 3. Supervised learning

1.5.1 3.0 downstream pipelines

We can specify embedders with AIDE into a scikitlearn pipeline, but to avoid recomputation of embeddings we will precompute them and pass them into specified pipelines. Many models leverage `CacheMixin` which helps significantly, but still requires IO - so here we just keep the X embeddings in memory.

[18]:
```
linear_nopca = Pipeline([
    ('scaler', StandardScaler()),
    ('model', TransformedTargetRegressor(
        regressor=Ridge(
            alpha=1.0,
```

```
        )))])
linear_pca = Pipeline([
    ('scaler', StandardScaler()),
    ('pca', PCA(n_components=0.9)),
    ('model', TransformedTargetRegressor(
        regressor=Ridge(
            alpha=1.0,
        )))])
linear_param_space = {
    'model__regressor__alpha': loguniform(1e-3, 1e3),
}
```

[19]:
```
mlp_nopca = Pipeline([
    ('scaler', StandardScaler()),
    ('model', TransformedTargetRegressor(
        regressor=MLPRegressor(
            hidden_layer_sizes=(20, 20),
            max_iter=1000,
            early_stopping=True
        )))])
mlp_pca = Pipeline([
    ('scaler', StandardScaler()),
    ('pca', PCA(n_components=0.9)),
    ('model', TransformedTargetRegressor(
        regressor=MLPRegressor(
            hidden_layer_sizes=(20, 20),
            max_iter=1000,
            early_stopping=True
        )))])
mlp_param_space = {
    'model__regressor__hidden_layer_sizes': [(20,), (20, 20), (20, 20, 20),␣
↪(20, 20, 20, 20)],
    'model__regressor__alpha': loguniform(1e-6, 1e-1),
    'model__regressor__activation': ['relu', 'tanh'],
}
```

1.5.2 3.1 OHE linear

[20]:
```
ohe = ap.OneHotProteinEmbedding(
    positions=library_positions,
    metadata_folder=os.path.join('.', 'data', 'epistatic', 'onehot'))
X_ohe = ohe.fit_transform(X)
```

```
INFO:aide_predict.bespoke_models.base:Fitting OneHotProteinEmbedding
INFO:aide_predict.bespoke_models.base:Transforming input using
OneHotProteinEmbedding
```

[53]:
```
none_ohe_linear_scores = hyperopt_and_scoring(linear_nopca, X_ohe, y,␣
↪linear_param_space)
joblib.dump(none_ohe_linear_scores, 'none_ohe_linear_scores.joblib')
```

[53]: ['none_ohe_linear_scores.joblib']

1.5.3 3.2 OHE MLP

[54]:
```
none_ohe_mlp_scores = hyperopt_and_scoring(mlp_nopca, X_ohe, y, mlp_param_space)
joblib.dump(none_ohe_mlp_scores, 'none_ohe_mlp_scores.joblib')
```

```
/projects/proteinml/.links/miniconda3/envs/aidep/lib/python3.10/site-
packages/sklearn/neural_network/_multilayer_perceptron.py:690:
ConvergenceWarning: Stochastic Optimizer: Maximum iterations (1000) reached and
the optimization hasn't converged yet.
  warnings.warn(
/projects/proteinml/.links/miniconda3/envs/aidep/lib/python3.10/site-
packages/sklearn/neural_network/_multilayer_perceptron.py:690:
ConvergenceWarning: Stochastic Optimizer: Maximum iterations (1000) reached and
the optimization hasn't converged yet.
  warnings.warn(
/projects/proteinml/.links/miniconda3/envs/aidep/lib/python3.10/site-
packages/sklearn/neural_network/_multilayer_perceptron.py:690:
ConvergenceWarning: Stochastic Optimizer: Maximum iterations (1000) reached and
the optimization hasn't converged yet.
  warnings.warn(
/projects/proteinml/.links/miniconda3/envs/aidep/lib/python3.10/site-
packages/sklearn/neural_network/_multilayer_perceptron.py:690:
ConvergenceWarning: Stochastic Optimizer: Maximum iterations (1000) reached and
the optimization hasn't converged yet.
  warnings.warn(
/projects/proteinml/.links/miniconda3/envs/aidep/lib/python3.10/site-
packages/sklearn/neural_network/_multilayer_perceptron.py:690:
ConvergenceWarning: Stochastic Optimizer: Maximum iterations (1000) reached and
the optimization hasn't converged yet.
  warnings.warn(
/projects/proteinml/.links/miniconda3/envs/aidep/lib/python3.10/site-
packages/sklearn/neural_network/_multilayer_perceptron.py:690:
ConvergenceWarning: Stochastic Optimizer: Maximum iterations (1000) reached and
the optimization hasn't converged yet.
  warnings.warn(
/projects/proteinml/.links/miniconda3/envs/aidep/lib/python3.10/site-
packages/sklearn/neural_network/_multilayer_perceptron.py:690:
ConvergenceWarning: Stochastic Optimizer: Maximum iterations (1000) reached and
the optimization hasn't converged yet.
  warnings.warn(
/projects/proteinml/.links/miniconda3/envs/aidep/lib/python3.10/site-
```

```
packages/sklearn/neural_network/_multilayer_perceptron.py:690:
ConvergenceWarning: Stochastic Optimizer: Maximum iterations (1000) reached and
the optimization hasn't converged yet.
  warnings.warn(
/projects/proteinml/.links/miniconda3/envs/aidep/lib/python3.10/site-
packages/sklearn/neural_network/_multilayer_perceptron.py:690:
ConvergenceWarning: Stochastic Optimizer: Maximum iterations (1000) reached and
the optimization hasn't converged yet.
  warnings.warn(
/projects/proteinml/.links/miniconda3/envs/aidep/lib/python3.10/site-
packages/sklearn/neural_network/_multilayer_perceptron.py:690:
ConvergenceWarning: Stochastic Optimizer: Maximum iterations (1000) reached and
the optimization hasn't converged yet.
  warnings.warn(
/projects/proteinml/.links/miniconda3/envs/aidep/lib/python3.10/site-
packages/sklearn/neural_network/_multilayer_perceptron.py:690:
ConvergenceWarning: Stochastic Optimizer: Maximum iterations (1000) reached and
the optimization hasn't converged yet.
  warnings.warn(
/projects/proteinml/.links/miniconda3/envs/aidep/lib/python3.10/site-
packages/sklearn/neural_network/_multilayer_perceptron.py:690:
ConvergenceWarning: Stochastic Optimizer: Maximum iterations (1000) reached and
the optimization hasn't converged yet.
  warnings.warn(
/projects/proteinml/.links/miniconda3/envs/aidep/lib/python3.10/site-
packages/sklearn/neural_network/_multilayer_perceptron.py:690:
ConvergenceWarning: Stochastic Optimizer: Maximum iterations (1000) reached and
the optimization hasn't converged yet.
  warnings.warn(
/projects/proteinml/.links/miniconda3/envs/aidep/lib/python3.10/site-
packages/sklearn/neural_network/_multilayer_perceptron.py:690:
ConvergenceWarning: Stochastic Optimizer: Maximum iterations (1000) reached and
the optimization hasn't converged yet.
  warnings.warn(
/projects/proteinml/.links/miniconda3/envs/aidep/lib/python3.10/site-
packages/sklearn/neural_network/_multilayer_perceptron.py:690:
ConvergenceWarning: Stochastic Optimizer: Maximum iterations (1000) reached and
the optimization hasn't converged yet.
  warnings.warn(
/projects/proteinml/.links/miniconda3/envs/aidep/lib/python3.10/site-
packages/sklearn/neural_network/_multilayer_perceptron.py:690:
ConvergenceWarning: Stochastic Optimizer: Maximum iterations (1000) reached and
the optimization hasn't converged yet.
  warnings.warn(
/projects/proteinml/.links/miniconda3/envs/aidep/lib/python3.10/site-
packages/sklearn/neural_network/_multilayer_perceptron.py:690:
ConvergenceWarning: Stochastic Optimizer: Maximum iterations (1000) reached and
the optimization hasn't converged yet.
```

```
  warnings.warn(
/projects/proteinml/.links/miniconda3/envs/aidep/lib/python3.10/site-
packages/sklearn/neural_network/_multilayer_perceptron.py:690:
ConvergenceWarning: Stochastic Optimizer: Maximum iterations (1000) reached and
the optimization hasn't converged yet.
  warnings.warn(
/projects/proteinml/.links/miniconda3/envs/aidep/lib/python3.10/site-
packages/sklearn/neural_network/_multilayer_perceptron.py:690:
ConvergenceWarning: Stochastic Optimizer: Maximum iterations (1000) reached and
the optimization hasn't converged yet.
  warnings.warn(
/projects/proteinml/.links/miniconda3/envs/aidep/lib/python3.10/site-
packages/sklearn/neural_network/_multilayer_perceptron.py:690:
ConvergenceWarning: Stochastic Optimizer: Maximum iterations (1000) reached and
the optimization hasn't converged yet.
  warnings.warn(
/projects/proteinml/.links/miniconda3/envs/aidep/lib/python3.10/site-
packages/sklearn/neural_network/_multilayer_perceptron.py:690:
ConvergenceWarning: Stochastic Optimizer: Maximum iterations (1000) reached and
the optimization hasn't converged yet.
  warnings.warn(
/projects/proteinml/.links/miniconda3/envs/aidep/lib/python3.10/site-
packages/sklearn/neural_network/_multilayer_perceptron.py:690:
ConvergenceWarning: Stochastic Optimizer: Maximum iterations (1000) reached and
the optimization hasn't converged yet.
  warnings.warn(
/projects/proteinml/.links/miniconda3/envs/aidep/lib/python3.10/site-
packages/sklearn/neural_network/_multilayer_perceptron.py:690:
ConvergenceWarning: Stochastic Optimizer: Maximum iterations (1000) reached and
the optimization hasn't converged yet.
  warnings.warn(
/projects/proteinml/.links/miniconda3/envs/aidep/lib/python3.10/site-
packages/sklearn/neural_network/_multilayer_perceptron.py:690:
ConvergenceWarning: Stochastic Optimizer: Maximum iterations (1000) reached and
the optimization hasn't converged yet.
  warnings.warn(
/projects/proteinml/.links/miniconda3/envs/aidep/lib/python3.10/site-
packages/sklearn/neural_network/_multilayer_perceptron.py:690:
ConvergenceWarning: Stochastic Optimizer: Maximum iterations (1000) reached and
the optimization hasn't converged yet.
  warnings.warn(
/projects/proteinml/.links/miniconda3/envs/aidep/lib/python3.10/site-
packages/sklearn/neural_network/_multilayer_perceptron.py:690:
ConvergenceWarning: Stochastic Optimizer: Maximum iterations (1000) reached and
the optimization hasn't converged yet.
  warnings.warn(
/projects/proteinml/.links/miniconda3/envs/aidep/lib/python3.10/site-
packages/sklearn/neural_network/_multilayer_perceptron.py:690:
```

```
ConvergenceWarning: Stochastic Optimizer: Maximum iterations (1000) reached and
the optimization hasn't converged yet.
  warnings.warn(
/projects/proteinml/.links/miniconda3/envs/aidep/lib/python3.10/site-
packages/sklearn/neural_network/_multilayer_perceptron.py:690:
ConvergenceWarning: Stochastic Optimizer: Maximum iterations (1000) reached and
the optimization hasn't converged yet.
  warnings.warn(
/projects/proteinml/.links/miniconda3/envs/aidep/lib/python3.10/site-
packages/sklearn/neural_network/_multilayer_perceptron.py:690:
ConvergenceWarning: Stochastic Optimizer: Maximum iterations (1000) reached and
the optimization hasn't converged yet.
  warnings.warn(
/projects/proteinml/.links/miniconda3/envs/aidep/lib/python3.10/site-
packages/sklearn/neural_network/_multilayer_perceptron.py:690:
ConvergenceWarning: Stochastic Optimizer: Maximum iterations (1000) reached and
the optimization hasn't converged yet.
  warnings.warn(
/projects/proteinml/.links/miniconda3/envs/aidep/lib/python3.10/site-
packages/sklearn/neural_network/_multilayer_perceptron.py:690:
ConvergenceWarning: Stochastic Optimizer: Maximum iterations (1000) reached and
the optimization hasn't converged yet.
  warnings.warn(
/projects/proteinml/.links/miniconda3/envs/aidep/lib/python3.10/site-
packages/sklearn/neural_network/_multilayer_perceptron.py:690:
ConvergenceWarning: Stochastic Optimizer: Maximum iterations (1000) reached and
the optimization hasn't converged yet.
  warnings.warn(
/projects/proteinml/.links/miniconda3/envs/aidep/lib/python3.10/site-
packages/sklearn/neural_network/_multilayer_perceptron.py:690:
ConvergenceWarning: Stochastic Optimizer: Maximum iterations (1000) reached and
the optimization hasn't converged yet.
  warnings.warn(
/projects/proteinml/.links/miniconda3/envs/aidep/lib/python3.10/site-
packages/sklearn/neural_network/_multilayer_perceptron.py:690:
ConvergenceWarning: Stochastic Optimizer: Maximum iterations (1000) reached and
the optimization hasn't converged yet.
  warnings.warn(
/projects/proteinml/.links/miniconda3/envs/aidep/lib/python3.10/site-
packages/sklearn/neural_network/_multilayer_perceptron.py:690:
ConvergenceWarning: Stochastic Optimizer: Maximum iterations (1000) reached and
the optimization hasn't converged yet.
  warnings.warn(
/projects/proteinml/.links/miniconda3/envs/aidep/lib/python3.10/site-
packages/sklearn/neural_network/_multilayer_perceptron.py:690:
ConvergenceWarning: Stochastic Optimizer: Maximum iterations (1000) reached and
the optimization hasn't converged yet.
  warnings.warn(
```

```
/projects/proteinml/.links/miniconda3/envs/aidep/lib/python3.10/site-
packages/sklearn/neural_network/_multilayer_perceptron.py:690:
ConvergenceWarning: Stochastic Optimizer: Maximum iterations (1000) reached and
the optimization hasn't converged yet.
  warnings.warn(
/projects/proteinml/.links/miniconda3/envs/aidep/lib/python3.10/site-
packages/sklearn/neural_network/_multilayer_perceptron.py:690:
ConvergenceWarning: Stochastic Optimizer: Maximum iterations (1000) reached and
the optimization hasn't converged yet.
  warnings.warn(
/projects/proteinml/.links/miniconda3/envs/aidep/lib/python3.10/site-
packages/sklearn/neural_network/_multilayer_perceptron.py:690:
ConvergenceWarning: Stochastic Optimizer: Maximum iterations (1000) reached and
the optimization hasn't converged yet.
  warnings.warn(
/projects/proteinml/.links/miniconda3/envs/aidep/lib/python3.10/site-
packages/sklearn/neural_network/_multilayer_perceptron.py:690:
ConvergenceWarning: Stochastic Optimizer: Maximum iterations (1000) reached and
the optimization hasn't converged yet.
  warnings.warn(
/projects/proteinml/.links/miniconda3/envs/aidep/lib/python3.10/site-
packages/sklearn/neural_network/_multilayer_perceptron.py:690:
ConvergenceWarning: Stochastic Optimizer: Maximum iterations (1000) reached and
the optimization hasn't converged yet.
  warnings.warn(
/projects/proteinml/.links/miniconda3/envs/aidep/lib/python3.10/site-
packages/sklearn/neural_network/_multilayer_perceptron.py:690:
ConvergenceWarning: Stochastic Optimizer: Maximum iterations (1000) reached and
the optimization hasn't converged yet.
  warnings.warn(
/projects/proteinml/.links/miniconda3/envs/aidep/lib/python3.10/site-
packages/sklearn/neural_network/_multilayer_perceptron.py:690:
ConvergenceWarning: Stochastic Optimizer: Maximum iterations (1000) reached and
the optimization hasn't converged yet.
  warnings.warn(
/projects/proteinml/.links/miniconda3/envs/aidep/lib/python3.10/site-
packages/sklearn/neural_network/_multilayer_perceptron.py:690:
ConvergenceWarning: Stochastic Optimizer: Maximum iterations (1000) reached and
the optimization hasn't converged yet.
  warnings.warn(
/projects/proteinml/.links/miniconda3/envs/aidep/lib/python3.10/site-
packages/sklearn/neural_network/_multilayer_perceptron.py:690:
ConvergenceWarning: Stochastic Optimizer: Maximum iterations (1000) reached and
the optimization hasn't converged yet.
  warnings.warn(
/projects/proteinml/.links/miniconda3/envs/aidep/lib/python3.10/site-
packages/sklearn/neural_network/_multilayer_perceptron.py:690:
ConvergenceWarning: Stochastic Optimizer: Maximum iterations (1000) reached and
```

```
the optimization hasn't converged yet.
  warnings.warn(
/projects/proteinml/.links/miniconda3/envs/aidep/lib/python3.10/site-
packages/sklearn/neural_network/_multilayer_perceptron.py:690:
ConvergenceWarning: Stochastic Optimizer: Maximum iterations (1000) reached and
the optimization hasn't converged yet.
  warnings.warn(
/projects/proteinml/.links/miniconda3/envs/aidep/lib/python3.10/site-
packages/sklearn/neural_network/_multilayer_perceptron.py:690:
ConvergenceWarning: Stochastic Optimizer: Maximum iterations (1000) reached and
the optimization hasn't converged yet.
  warnings.warn(
/projects/proteinml/.links/miniconda3/envs/aidep/lib/python3.10/site-
packages/sklearn/neural_network/_multilayer_perceptron.py:690:
ConvergenceWarning: Stochastic Optimizer: Maximum iterations (1000) reached and
the optimization hasn't converged yet.
  warnings.warn(
/projects/proteinml/.links/miniconda3/envs/aidep/lib/python3.10/site-
packages/sklearn/neural_network/_multilayer_perceptron.py:690:
ConvergenceWarning: Stochastic Optimizer: Maximum iterations (1000) reached and
the optimization hasn't converged yet.
  warnings.warn(
/projects/proteinml/.links/miniconda3/envs/aidep/lib/python3.10/site-
packages/sklearn/neural_network/_multilayer_perceptron.py:690:
ConvergenceWarning: Stochastic Optimizer: Maximum iterations (1000) reached and
the optimization hasn't converged yet.
  warnings.warn(
/projects/proteinml/.links/miniconda3/envs/aidep/lib/python3.10/site-
packages/sklearn/neural_network/_multilayer_perceptron.py:690:
ConvergenceWarning: Stochastic Optimizer: Maximum iterations (1000) reached and
the optimization hasn't converged yet.
  warnings.warn(
/projects/proteinml/.links/miniconda3/envs/aidep/lib/python3.10/site-
packages/sklearn/neural_network/_multilayer_perceptron.py:690:
ConvergenceWarning: Stochastic Optimizer: Maximum iterations (1000) reached and
the optimization hasn't converged yet.
  warnings.warn(
/projects/proteinml/.links/miniconda3/envs/aidep/lib/python3.10/site-
packages/sklearn/neural_network/_multilayer_perceptron.py:690:
ConvergenceWarning: Stochastic Optimizer: Maximum iterations (1000) reached and
the optimization hasn't converged yet.
  warnings.warn(
/projects/proteinml/.links/miniconda3/envs/aidep/lib/python3.10/site-
packages/sklearn/neural_network/_multilayer_perceptron.py:690:
ConvergenceWarning: Stochastic Optimizer: Maximum iterations (1000) reached and
the optimization hasn't converged yet.
  warnings.warn(
/projects/proteinml/.links/miniconda3/envs/aidep/lib/python3.10/site-
```

```
packages/sklearn/neural_network/_multilayer_perceptron.py:690:
ConvergenceWarning: Stochastic Optimizer: Maximum iterations (1000) reached and
the optimization hasn't converged yet.
  warnings.warn(
/projects/proteinml/.links/miniconda3/envs/aidep/lib/python3.10/site-
packages/sklearn/neural_network/_multilayer_perceptron.py:690:
ConvergenceWarning: Stochastic Optimizer: Maximum iterations (1000) reached and
the optimization hasn't converged yet.
  warnings.warn(
/projects/proteinml/.links/miniconda3/envs/aidep/lib/python3.10/site-
packages/sklearn/neural_network/_multilayer_perceptron.py:690:
ConvergenceWarning: Stochastic Optimizer: Maximum iterations (1000) reached and
the optimization hasn't converged yet.
  warnings.warn(
/projects/proteinml/.links/miniconda3/envs/aidep/lib/python3.10/site-
packages/sklearn/neural_network/_multilayer_perceptron.py:690:
ConvergenceWarning: Stochastic Optimizer: Maximum iterations (1000) reached and
the optimization hasn't converged yet.
  warnings.warn(
/projects/proteinml/.links/miniconda3/envs/aidep/lib/python3.10/site-
packages/sklearn/neural_network/_multilayer_perceptron.py:690:
ConvergenceWarning: Stochastic Optimizer: Maximum iterations (1000) reached and
the optimization hasn't converged yet.
  warnings.warn(
/projects/proteinml/.links/miniconda3/envs/aidep/lib/python3.10/site-
packages/sklearn/neural_network/_multilayer_perceptron.py:690:
ConvergenceWarning: Stochastic Optimizer: Maximum iterations (1000) reached and
the optimization hasn't converged yet.
  warnings.warn(
/projects/proteinml/.links/miniconda3/envs/aidep/lib/python3.10/site-
packages/sklearn/neural_network/_multilayer_perceptron.py:690:
ConvergenceWarning: Stochastic Optimizer: Maximum iterations (1000) reached and
the optimization hasn't converged yet.
  warnings.warn(
/projects/proteinml/.links/miniconda3/envs/aidep/lib/python3.10/site-
packages/sklearn/neural_network/_multilayer_perceptron.py:690:
ConvergenceWarning: Stochastic Optimizer: Maximum iterations (1000) reached and
the optimization hasn't converged yet.
  warnings.warn(
/projects/proteinml/.links/miniconda3/envs/aidep/lib/python3.10/site-
packages/sklearn/neural_network/_multilayer_perceptron.py:690:
ConvergenceWarning: Stochastic Optimizer: Maximum iterations (1000) reached and
the optimization hasn't converged yet.
  warnings.warn(
/projects/proteinml/.links/miniconda3/envs/aidep/lib/python3.10/site-
packages/sklearn/neural_network/_multilayer_perceptron.py:690:
ConvergenceWarning: Stochastic Optimizer: Maximum iterations (1000) reached and
the optimization hasn't converged yet.
```

```
  warnings.warn(
/projects/proteinml/.links/miniconda3/envs/aidep/lib/python3.10/site-
packages/sklearn/neural_network/_multilayer_perceptron.py:690:
ConvergenceWarning: Stochastic Optimizer: Maximum iterations (1000) reached and
the optimization hasn't converged yet.
  warnings.warn(
/projects/proteinml/.links/miniconda3/envs/aidep/lib/python3.10/site-
packages/sklearn/neural_network/_multilayer_perceptron.py:690:
ConvergenceWarning: Stochastic Optimizer: Maximum iterations (1000) reached and
the optimization hasn't converged yet.
  warnings.warn(
/projects/proteinml/.links/miniconda3/envs/aidep/lib/python3.10/site-
packages/sklearn/neural_network/_multilayer_perceptron.py:690:
ConvergenceWarning: Stochastic Optimizer: Maximum iterations (1000) reached and
the optimization hasn't converged yet.
  warnings.warn(
/projects/proteinml/.links/miniconda3/envs/aidep/lib/python3.10/site-
packages/sklearn/neural_network/_multilayer_perceptron.py:690:
ConvergenceWarning: Stochastic Optimizer: Maximum iterations (1000) reached and
the optimization hasn't converged yet.
  warnings.warn(
/projects/proteinml/.links/miniconda3/envs/aidep/lib/python3.10/site-
packages/sklearn/neural_network/_multilayer_perceptron.py:690:
ConvergenceWarning: Stochastic Optimizer: Maximum iterations (1000) reached and
the optimization hasn't converged yet.
  warnings.warn(
/projects/proteinml/.links/miniconda3/envs/aidep/lib/python3.10/site-
packages/sklearn/neural_network/_multilayer_perceptron.py:690:
ConvergenceWarning: Stochastic Optimizer: Maximum iterations (1000) reached and
the optimization hasn't converged yet.
  warnings.warn(
/projects/proteinml/.links/miniconda3/envs/aidep/lib/python3.10/site-
packages/sklearn/neural_network/_multilayer_perceptron.py:690:
ConvergenceWarning: Stochastic Optimizer: Maximum iterations (1000) reached and
the optimization hasn't converged yet.
  warnings.warn(
/projects/proteinml/.links/miniconda3/envs/aidep/lib/python3.10/site-
packages/sklearn/neural_network/_multilayer_perceptron.py:690:
ConvergenceWarning: Stochastic Optimizer: Maximum iterations (1000) reached and
the optimization hasn't converged yet.
  warnings.warn(
/projects/proteinml/.links/miniconda3/envs/aidep/lib/python3.10/site-
packages/sklearn/neural_network/_multilayer_perceptron.py:690:
ConvergenceWarning: Stochastic Optimizer: Maximum iterations (1000) reached and
the optimization hasn't converged yet.
  warnings.warn(
/projects/proteinml/.links/miniconda3/envs/aidep/lib/python3.10/site-
packages/sklearn/neural_network/_multilayer_perceptron.py:690:
```

```
ConvergenceWarning: Stochastic Optimizer: Maximum iterations (1000) reached and
the optimization hasn't converged yet.
  warnings.warn(
/projects/proteinml/.links/miniconda3/envs/aidep/lib/python3.10/site-
packages/sklearn/neural_network/_multilayer_perceptron.py:690:
ConvergenceWarning: Stochastic Optimizer: Maximum iterations (1000) reached and
the optimization hasn't converged yet.
  warnings.warn(
/projects/proteinml/.links/miniconda3/envs/aidep/lib/python3.10/site-
packages/sklearn/neural_network/_multilayer_perceptron.py:690:
ConvergenceWarning: Stochastic Optimizer: Maximum iterations (1000) reached and
the optimization hasn't converged yet.
  warnings.warn(
/projects/proteinml/.links/miniconda3/envs/aidep/lib/python3.10/site-
packages/sklearn/neural_network/_multilayer_perceptron.py:690:
ConvergenceWarning: Stochastic Optimizer: Maximum iterations (1000) reached and
the optimization hasn't converged yet.
  warnings.warn(
/projects/proteinml/.links/miniconda3/envs/aidep/lib/python3.10/site-
packages/sklearn/neural_network/_multilayer_perceptron.py:690:
ConvergenceWarning: Stochastic Optimizer: Maximum iterations (1000) reached and
the optimization hasn't converged yet.
  warnings.warn(
/projects/proteinml/.links/miniconda3/envs/aidep/lib/python3.10/site-
packages/sklearn/neural_network/_multilayer_perceptron.py:690:
ConvergenceWarning: Stochastic Optimizer: Maximum iterations (1000) reached and
the optimization hasn't converged yet.
  warnings.warn(
/projects/proteinml/.links/miniconda3/envs/aidep/lib/python3.10/site-
packages/sklearn/neural_network/_multilayer_perceptron.py:690:
ConvergenceWarning: Stochastic Optimizer: Maximum iterations (1000) reached and
the optimization hasn't converged yet.
  warnings.warn(
/projects/proteinml/.links/miniconda3/envs/aidep/lib/python3.10/site-
packages/sklearn/neural_network/_multilayer_perceptron.py:690:
ConvergenceWarning: Stochastic Optimizer: Maximum iterations (1000) reached and
the optimization hasn't converged yet.
  warnings.warn(
/projects/proteinml/.links/miniconda3/envs/aidep/lib/python3.10/site-
packages/sklearn/neural_network/_multilayer_perceptron.py:690:
ConvergenceWarning: Stochastic Optimizer: Maximum iterations (1000) reached and
the optimization hasn't converged yet.
  warnings.warn(
/projects/proteinml/.links/miniconda3/envs/aidep/lib/python3.10/site-
packages/sklearn/neural_network/_multilayer_perceptron.py:690:
ConvergenceWarning: Stochastic Optimizer: Maximum iterations (1000) reached and
the optimization hasn't converged yet.
  warnings.warn(
```

```
/projects/proteinml/.links/miniconda3/envs/aidep/lib/python3.10/site-
packages/sklearn/neural_network/_multilayer_perceptron.py:690:
ConvergenceWarning: Stochastic Optimizer: Maximum iterations (1000) reached and
the optimization hasn't converged yet.
  warnings.warn(
/projects/proteinml/.links/miniconda3/envs/aidep/lib/python3.10/site-
packages/sklearn/neural_network/_multilayer_perceptron.py:690:
ConvergenceWarning: Stochastic Optimizer: Maximum iterations (1000) reached and
the optimization hasn't converged yet.
  warnings.warn(
/projects/proteinml/.links/miniconda3/envs/aidep/lib/python3.10/site-
packages/sklearn/neural_network/_multilayer_perceptron.py:690:
ConvergenceWarning: Stochastic Optimizer: Maximum iterations (1000) reached and
the optimization hasn't converged yet.
  warnings.warn(
/projects/proteinml/.links/miniconda3/envs/aidep/lib/python3.10/site-
packages/sklearn/neural_network/_multilayer_perceptron.py:690:
ConvergenceWarning: Stochastic Optimizer: Maximum iterations (1000) reached and
the optimization hasn't converged yet.
  warnings.warn(
/projects/proteinml/.links/miniconda3/envs/aidep/lib/python3.10/site-
packages/sklearn/neural_network/_multilayer_perceptron.py:690:
ConvergenceWarning: Stochastic Optimizer: Maximum iterations (1000) reached and
the optimization hasn't converged yet.
  warnings.warn(
/projects/proteinml/.links/miniconda3/envs/aidep/lib/python3.10/site-
packages/sklearn/neural_network/_multilayer_perceptron.py:690:
ConvergenceWarning: Stochastic Optimizer: Maximum iterations (1000) reached and
the optimization hasn't converged yet.
  warnings.warn(
/projects/proteinml/.links/miniconda3/envs/aidep/lib/python3.10/site-
packages/sklearn/neural_network/_multilayer_perceptron.py:690:
ConvergenceWarning: Stochastic Optimizer: Maximum iterations (1000) reached and
the optimization hasn't converged yet.
  warnings.warn(
/projects/proteinml/.links/miniconda3/envs/aidep/lib/python3.10/site-
packages/sklearn/neural_network/_multilayer_perceptron.py:690:
ConvergenceWarning: Stochastic Optimizer: Maximum iterations (1000) reached and
the optimization hasn't converged yet.
  warnings.warn(
/projects/proteinml/.links/miniconda3/envs/aidep/lib/python3.10/site-
packages/sklearn/neural_network/_multilayer_perceptron.py:690:
ConvergenceWarning: Stochastic Optimizer: Maximum iterations (1000) reached and
the optimization hasn't converged yet.
  warnings.warn(
/projects/proteinml/.links/miniconda3/envs/aidep/lib/python3.10/site-
packages/sklearn/neural_network/_multilayer_perceptron.py:690:
ConvergenceWarning: Stochastic Optimizer: Maximum iterations (1000) reached and
```

```
the optimization hasn't converged yet.
  warnings.warn(
/projects/proteinml/.links/miniconda3/envs/aidep/lib/python3.10/site-
packages/sklearn/neural_network/_multilayer_perceptron.py:690:
ConvergenceWarning: Stochastic Optimizer: Maximum iterations (1000) reached and
the optimization hasn't converged yet.
  warnings.warn(
/projects/proteinml/.links/miniconda3/envs/aidep/lib/python3.10/site-
packages/sklearn/neural_network/_multilayer_perceptron.py:690:
ConvergenceWarning: Stochastic Optimizer: Maximum iterations (1000) reached and
the optimization hasn't converged yet.
  warnings.warn(
/projects/proteinml/.links/miniconda3/envs/aidep/lib/python3.10/site-
packages/sklearn/neural_network/_multilayer_perceptron.py:690:
ConvergenceWarning: Stochastic Optimizer: Maximum iterations (1000) reached and
the optimization hasn't converged yet.
  warnings.warn(
/projects/proteinml/.links/miniconda3/envs/aidep/lib/python3.10/site-
packages/sklearn/neural_network/_multilayer_perceptron.py:690:
ConvergenceWarning: Stochastic Optimizer: Maximum iterations (1000) reached and
the optimization hasn't converged yet.
  warnings.warn(
/projects/proteinml/.links/miniconda3/envs/aidep/lib/python3.10/site-
packages/sklearn/neural_network/_multilayer_perceptron.py:690:
ConvergenceWarning: Stochastic Optimizer: Maximum iterations (1000) reached and
the optimization hasn't converged yet.
  warnings.warn(
/projects/proteinml/.links/miniconda3/envs/aidep/lib/python3.10/site-
packages/sklearn/neural_network/_multilayer_perceptron.py:690:
ConvergenceWarning: Stochastic Optimizer: Maximum iterations (1000) reached and
the optimization hasn't converged yet.
  warnings.warn(
/projects/proteinml/.links/miniconda3/envs/aidep/lib/python3.10/site-
packages/sklearn/neural_network/_multilayer_perceptron.py:690:
ConvergenceWarning: Stochastic Optimizer: Maximum iterations (1000) reached and
the optimization hasn't converged yet.
  warnings.warn(
/projects/proteinml/.links/miniconda3/envs/aidep/lib/python3.10/site-
packages/sklearn/neural_network/_multilayer_perceptron.py:690:
ConvergenceWarning: Stochastic Optimizer: Maximum iterations (1000) reached and
the optimization hasn't converged yet.
  warnings.warn(
/projects/proteinml/.links/miniconda3/envs/aidep/lib/python3.10/site-
packages/sklearn/neural_network/_multilayer_perceptron.py:690:
ConvergenceWarning: Stochastic Optimizer: Maximum iterations (1000) reached and
the optimization hasn't converged yet.
  warnings.warn(
/projects/proteinml/.links/miniconda3/envs/aidep/lib/python3.10/site-
```

```
packages/sklearn/neural_network/_multilayer_perceptron.py:690:
ConvergenceWarning: Stochastic Optimizer: Maximum iterations (1000) reached and
the optimization hasn't converged yet.
  warnings.warn(
/projects/proteinml/.links/miniconda3/envs/aidep/lib/python3.10/site-
packages/sklearn/neural_network/_multilayer_perceptron.py:690:
ConvergenceWarning: Stochastic Optimizer: Maximum iterations (1000) reached and
the optimization hasn't converged yet.
  warnings.warn(
/projects/proteinml/.links/miniconda3/envs/aidep/lib/python3.10/site-
packages/sklearn/neural_network/_multilayer_perceptron.py:690:
ConvergenceWarning: Stochastic Optimizer: Maximum iterations (1000) reached and
the optimization hasn't converged yet.
  warnings.warn(
/projects/proteinml/.links/miniconda3/envs/aidep/lib/python3.10/site-
packages/sklearn/neural_network/_multilayer_perceptron.py:690:
ConvergenceWarning: Stochastic Optimizer: Maximum iterations (1000) reached and
the optimization hasn't converged yet.
  warnings.warn(
/projects/proteinml/.links/miniconda3/envs/aidep/lib/python3.10/site-
packages/sklearn/neural_network/_multilayer_perceptron.py:690:
ConvergenceWarning: Stochastic Optimizer: Maximum iterations (1000) reached and
the optimization hasn't converged yet.
  warnings.warn(
/projects/proteinml/.links/miniconda3/envs/aidep/lib/python3.10/site-
packages/sklearn/neural_network/_multilayer_perceptron.py:690:
ConvergenceWarning: Stochastic Optimizer: Maximum iterations (1000) reached and
the optimization hasn't converged yet.
  warnings.warn(
/projects/proteinml/.links/miniconda3/envs/aidep/lib/python3.10/site-
packages/sklearn/neural_network/_multilayer_perceptron.py:690:
ConvergenceWarning: Stochastic Optimizer: Maximum iterations (1000) reached and
the optimization hasn't converged yet.
  warnings.warn(
/projects/proteinml/.links/miniconda3/envs/aidep/lib/python3.10/site-
packages/sklearn/neural_network/_multilayer_perceptron.py:690:
ConvergenceWarning: Stochastic Optimizer: Maximum iterations (1000) reached and
the optimization hasn't converged yet.
  warnings.warn(
/projects/proteinml/.links/miniconda3/envs/aidep/lib/python3.10/site-
packages/sklearn/neural_network/_multilayer_perceptron.py:690:
ConvergenceWarning: Stochastic Optimizer: Maximum iterations (1000) reached and
the optimization hasn't converged yet.
  warnings.warn(
/projects/proteinml/.links/miniconda3/envs/aidep/lib/python3.10/site-
packages/sklearn/neural_network/_multilayer_perceptron.py:690:
ConvergenceWarning: Stochastic Optimizer: Maximum iterations (1000) reached and
the optimization hasn't converged yet.
```

```
  warnings.warn(
/projects/proteinml/.links/miniconda3/envs/aidep/lib/python3.10/site-
packages/sklearn/neural_network/_multilayer_perceptron.py:690:
ConvergenceWarning: Stochastic Optimizer: Maximum iterations (1000) reached and
the optimization hasn't converged yet.
  warnings.warn(
/projects/proteinml/.links/miniconda3/envs/aidep/lib/python3.10/site-
packages/sklearn/neural_network/_multilayer_perceptron.py:690:
ConvergenceWarning: Stochastic Optimizer: Maximum iterations (1000) reached and
the optimization hasn't converged yet.
  warnings.warn(
/projects/proteinml/.links/miniconda3/envs/aidep/lib/python3.10/site-
packages/sklearn/neural_network/_multilayer_perceptron.py:690:
ConvergenceWarning: Stochastic Optimizer: Maximum iterations (1000) reached and
the optimization hasn't converged yet.
  warnings.warn(
/projects/proteinml/.links/miniconda3/envs/aidep/lib/python3.10/site-
packages/sklearn/neural_network/_multilayer_perceptron.py:690:
ConvergenceWarning: Stochastic Optimizer: Maximum iterations (1000) reached and
the optimization hasn't converged yet.
  warnings.warn(
/projects/proteinml/.links/miniconda3/envs/aidep/lib/python3.10/site-
packages/sklearn/neural_network/_multilayer_perceptron.py:690:
ConvergenceWarning: Stochastic Optimizer: Maximum iterations (1000) reached and
the optimization hasn't converged yet.
  warnings.warn(
/projects/proteinml/.links/miniconda3/envs/aidep/lib/python3.10/site-
packages/sklearn/neural_network/_multilayer_perceptron.py:690:
ConvergenceWarning: Stochastic Optimizer: Maximum iterations (1000) reached and
the optimization hasn't converged yet.
  warnings.warn(
/projects/proteinml/.links/miniconda3/envs/aidep/lib/python3.10/site-
packages/sklearn/neural_network/_multilayer_perceptron.py:690:
ConvergenceWarning: Stochastic Optimizer: Maximum iterations (1000) reached and
the optimization hasn't converged yet.
  warnings.warn(
/projects/proteinml/.links/miniconda3/envs/aidep/lib/python3.10/site-
packages/sklearn/neural_network/_multilayer_perceptron.py:690:
ConvergenceWarning: Stochastic Optimizer: Maximum iterations (1000) reached and
the optimization hasn't converged yet.
  warnings.warn(
/projects/proteinml/.links/miniconda3/envs/aidep/lib/python3.10/site-
packages/sklearn/neural_network/_multilayer_perceptron.py:690:
ConvergenceWarning: Stochastic Optimizer: Maximum iterations (1000) reached and
the optimization hasn't converged yet.
  warnings.warn(
/projects/proteinml/.links/miniconda3/envs/aidep/lib/python3.10/site-
packages/sklearn/neural_network/_multilayer_perceptron.py:690:
```

```
ConvergenceWarning: Stochastic Optimizer: Maximum iterations (1000) reached and
the optimization hasn't converged yet.
  warnings.warn(
/projects/proteinml/.links/miniconda3/envs/aidep/lib/python3.10/site-
packages/sklearn/neural_network/_multilayer_perceptron.py:690:
ConvergenceWarning: Stochastic Optimizer: Maximum iterations (1000) reached and
the optimization hasn't converged yet.
  warnings.warn(
/projects/proteinml/.links/miniconda3/envs/aidep/lib/python3.10/site-
packages/sklearn/neural_network/_multilayer_perceptron.py:690:
ConvergenceWarning: Stochastic Optimizer: Maximum iterations (1000) reached and
the optimization hasn't converged yet.
  warnings.warn(
/projects/proteinml/.links/miniconda3/envs/aidep/lib/python3.10/site-
packages/sklearn/neural_network/_multilayer_perceptron.py:690:
ConvergenceWarning: Stochastic Optimizer: Maximum iterations (1000) reached and
the optimization hasn't converged yet.
  warnings.warn(
/projects/proteinml/.links/miniconda3/envs/aidep/lib/python3.10/site-
packages/sklearn/neural_network/_multilayer_perceptron.py:690:
ConvergenceWarning: Stochastic Optimizer: Maximum iterations (1000) reached and
the optimization hasn't converged yet.
  warnings.warn(
/projects/proteinml/.links/miniconda3/envs/aidep/lib/python3.10/site-
packages/sklearn/neural_network/_multilayer_perceptron.py:690:
ConvergenceWarning: Stochastic Optimizer: Maximum iterations (1000) reached and
the optimization hasn't converged yet.
  warnings.warn(
/projects/proteinml/.links/miniconda3/envs/aidep/lib/python3.10/site-
packages/sklearn/neural_network/_multilayer_perceptron.py:690:
ConvergenceWarning: Stochastic Optimizer: Maximum iterations (1000) reached and
the optimization hasn't converged yet.
  warnings.warn(
/projects/proteinml/.links/miniconda3/envs/aidep/lib/python3.10/site-
packages/sklearn/neural_network/_multilayer_perceptron.py:690:
ConvergenceWarning: Stochastic Optimizer: Maximum iterations (1000) reached and
the optimization hasn't converged yet.
  warnings.warn(
/projects/proteinml/.links/miniconda3/envs/aidep/lib/python3.10/site-
packages/sklearn/neural_network/_multilayer_perceptron.py:690:
ConvergenceWarning: Stochastic Optimizer: Maximum iterations (1000) reached and
the optimization hasn't converged yet.
  warnings.warn(
/projects/proteinml/.links/miniconda3/envs/aidep/lib/python3.10/site-
packages/sklearn/neural_network/_multilayer_perceptron.py:690:
ConvergenceWarning: Stochastic Optimizer: Maximum iterations (1000) reached and
the optimization hasn't converged yet.
  warnings.warn(
```

```
/projects/proteinml/.links/miniconda3/envs/aidep/lib/python3.10/site-
packages/sklearn/neural_network/_multilayer_perceptron.py:690:
ConvergenceWarning: Stochastic Optimizer: Maximum iterations (1000) reached and
the optimization hasn't converged yet.
  warnings.warn(
/projects/proteinml/.links/miniconda3/envs/aidep/lib/python3.10/site-
packages/sklearn/neural_network/_multilayer_perceptron.py:690:
ConvergenceWarning: Stochastic Optimizer: Maximum iterations (1000) reached and
the optimization hasn't converged yet.
  warnings.warn(
/projects/proteinml/.links/miniconda3/envs/aidep/lib/python3.10/site-
packages/sklearn/neural_network/_multilayer_perceptron.py:690:
ConvergenceWarning: Stochastic Optimizer: Maximum iterations (1000) reached and
the optimization hasn't converged yet.
  warnings.warn(
/projects/proteinml/.links/miniconda3/envs/aidep/lib/python3.10/site-
packages/sklearn/neural_network/_multilayer_perceptron.py:690:
ConvergenceWarning: Stochastic Optimizer: Maximum iterations (1000) reached and
the optimization hasn't converged yet.
  warnings.warn(
/projects/proteinml/.links/miniconda3/envs/aidep/lib/python3.10/site-
packages/sklearn/neural_network/_multilayer_perceptron.py:690:
ConvergenceWarning: Stochastic Optimizer: Maximum iterations (1000) reached and
the optimization hasn't converged yet.
  warnings.warn(
/projects/proteinml/.links/miniconda3/envs/aidep/lib/python3.10/site-
packages/sklearn/neural_network/_multilayer_perceptron.py:690:
ConvergenceWarning: Stochastic Optimizer: Maximum iterations (1000) reached and
the optimization hasn't converged yet.
  warnings.warn(
/projects/proteinml/.links/miniconda3/envs/aidep/lib/python3.10/site-
packages/sklearn/neural_network/_multilayer_perceptron.py:690:
ConvergenceWarning: Stochastic Optimizer: Maximum iterations (1000) reached and
the optimization hasn't converged yet.
  warnings.warn(
/projects/proteinml/.links/miniconda3/envs/aidep/lib/python3.10/site-
packages/sklearn/neural_network/_multilayer_perceptron.py:690:
ConvergenceWarning: Stochastic Optimizer: Maximum iterations (1000) reached and
the optimization hasn't converged yet.
  warnings.warn(
/projects/proteinml/.links/miniconda3/envs/aidep/lib/python3.10/site-
packages/sklearn/neural_network/_multilayer_perceptron.py:690:
ConvergenceWarning: Stochastic Optimizer: Maximum iterations (1000) reached and
the optimization hasn't converged yet.
  warnings.warn(
/projects/proteinml/.links/miniconda3/envs/aidep/lib/python3.10/site-
packages/sklearn/neural_network/_multilayer_perceptron.py:690:
ConvergenceWarning: Stochastic Optimizer: Maximum iterations (1000) reached and
```

```
the optimization hasn't converged yet.
  warnings.warn(
/projects/proteinml/.links/miniconda3/envs/aidep/lib/python3.10/site-
packages/sklearn/neural_network/_multilayer_perceptron.py:690:
ConvergenceWarning: Stochastic Optimizer: Maximum iterations (1000) reached and
the optimization hasn't converged yet.
  warnings.warn(
/projects/proteinml/.links/miniconda3/envs/aidep/lib/python3.10/site-
packages/sklearn/neural_network/_multilayer_perceptron.py:690:
ConvergenceWarning: Stochastic Optimizer: Maximum iterations (1000) reached and
the optimization hasn't converged yet.
  warnings.warn(
/projects/proteinml/.links/miniconda3/envs/aidep/lib/python3.10/site-
packages/sklearn/neural_network/_multilayer_perceptron.py:690:
ConvergenceWarning: Stochastic Optimizer: Maximum iterations (1000) reached and
the optimization hasn't converged yet.
  warnings.warn(
/projects/proteinml/.links/miniconda3/envs/aidep/lib/python3.10/site-
packages/sklearn/neural_network/_multilayer_perceptron.py:690:
ConvergenceWarning: Stochastic Optimizer: Maximum iterations (1000) reached and
the optimization hasn't converged yet.
  warnings.warn(
/projects/proteinml/.links/miniconda3/envs/aidep/lib/python3.10/site-
packages/sklearn/neural_network/_multilayer_perceptron.py:690:
ConvergenceWarning: Stochastic Optimizer: Maximum iterations (1000) reached and
the optimization hasn't converged yet.
  warnings.warn(
/projects/proteinml/.links/miniconda3/envs/aidep/lib/python3.10/site-
packages/sklearn/neural_network/_multilayer_perceptron.py:690:
ConvergenceWarning: Stochastic Optimizer: Maximum iterations (1000) reached and
the optimization hasn't converged yet.
  warnings.warn(
/projects/proteinml/.links/miniconda3/envs/aidep/lib/python3.10/site-
packages/sklearn/neural_network/_multilayer_perceptron.py:690:
ConvergenceWarning: Stochastic Optimizer: Maximum iterations (1000) reached and
the optimization hasn't converged yet.
  warnings.warn(
/projects/proteinml/.links/miniconda3/envs/aidep/lib/python3.10/site-
packages/sklearn/neural_network/_multilayer_perceptron.py:690:
ConvergenceWarning: Stochastic Optimizer: Maximum iterations (1000) reached and
the optimization hasn't converged yet.
  warnings.warn(
/projects/proteinml/.links/miniconda3/envs/aidep/lib/python3.10/site-
packages/sklearn/neural_network/_multilayer_perceptron.py:690:
ConvergenceWarning: Stochastic Optimizer: Maximum iterations (1000) reached and
the optimization hasn't converged yet.
  warnings.warn(
/projects/proteinml/.links/miniconda3/envs/aidep/lib/python3.10/site-
```

```
packages/sklearn/neural_network/_multilayer_perceptron.py:690:
ConvergenceWarning: Stochastic Optimizer: Maximum iterations (1000) reached and
the optimization hasn't converged yet.
  warnings.warn(
/projects/proteinml/.links/miniconda3/envs/aidep/lib/python3.10/site-
packages/sklearn/neural_network/_multilayer_perceptron.py:690:
ConvergenceWarning: Stochastic Optimizer: Maximum iterations (1000) reached and
the optimization hasn't converged yet.
  warnings.warn(
/projects/proteinml/.links/miniconda3/envs/aidep/lib/python3.10/site-
packages/sklearn/neural_network/_multilayer_perceptron.py:690:
ConvergenceWarning: Stochastic Optimizer: Maximum iterations (1000) reached and
the optimization hasn't converged yet.
  warnings.warn(
/projects/proteinml/.links/miniconda3/envs/aidep/lib/python3.10/site-
packages/sklearn/neural_network/_multilayer_perceptron.py:690:
ConvergenceWarning: Stochastic Optimizer: Maximum iterations (1000) reached and
the optimization hasn't converged yet.
  warnings.warn(
/projects/proteinml/.links/miniconda3/envs/aidep/lib/python3.10/site-
packages/sklearn/neural_network/_multilayer_perceptron.py:690:
ConvergenceWarning: Stochastic Optimizer: Maximum iterations (1000) reached and
the optimization hasn't converged yet.
  warnings.warn(
/projects/proteinml/.links/miniconda3/envs/aidep/lib/python3.10/site-
packages/sklearn/neural_network/_multilayer_perceptron.py:690:
ConvergenceWarning: Stochastic Optimizer: Maximum iterations (1000) reached and
the optimization hasn't converged yet.
  warnings.warn(
/projects/proteinml/.links/miniconda3/envs/aidep/lib/python3.10/site-
packages/sklearn/neural_network/_multilayer_perceptron.py:690:
ConvergenceWarning: Stochastic Optimizer: Maximum iterations (1000) reached and
the optimization hasn't converged yet.
  warnings.warn(
/projects/proteinml/.links/miniconda3/envs/aidep/lib/python3.10/site-
packages/sklearn/neural_network/_multilayer_perceptron.py:690:
ConvergenceWarning: Stochastic Optimizer: Maximum iterations (1000) reached and
the optimization hasn't converged yet.
  warnings.warn(
/projects/proteinml/.links/miniconda3/envs/aidep/lib/python3.10/site-
packages/sklearn/neural_network/_multilayer_perceptron.py:690:
ConvergenceWarning: Stochastic Optimizer: Maximum iterations (1000) reached and
the optimization hasn't converged yet.
  warnings.warn(
/projects/proteinml/.links/miniconda3/envs/aidep/lib/python3.10/site-
packages/sklearn/neural_network/_multilayer_perceptron.py:690:
ConvergenceWarning: Stochastic Optimizer: Maximum iterations (1000) reached and
the optimization hasn't converged yet.
```

```
  warnings.warn(
/projects/proteinml/.links/miniconda3/envs/aidep/lib/python3.10/site-
packages/sklearn/neural_network/_multilayer_perceptron.py:690:
ConvergenceWarning: Stochastic Optimizer: Maximum iterations (1000) reached and
the optimization hasn't converged yet.
  warnings.warn(
/projects/proteinml/.links/miniconda3/envs/aidep/lib/python3.10/site-
packages/sklearn/neural_network/_multilayer_perceptron.py:690:
ConvergenceWarning: Stochastic Optimizer: Maximum iterations (1000) reached and
the optimization hasn't converged yet.
  warnings.warn(
/projects/proteinml/.links/miniconda3/envs/aidep/lib/python3.10/site-
packages/sklearn/neural_network/_multilayer_perceptron.py:690:
ConvergenceWarning: Stochastic Optimizer: Maximum iterations (1000) reached and
the optimization hasn't converged yet.
  warnings.warn(
/projects/proteinml/.links/miniconda3/envs/aidep/lib/python3.10/site-
packages/sklearn/neural_network/_multilayer_perceptron.py:690:
ConvergenceWarning: Stochastic Optimizer: Maximum iterations (1000) reached and
the optimization hasn't converged yet.
  warnings.warn(
/projects/proteinml/.links/miniconda3/envs/aidep/lib/python3.10/site-
packages/sklearn/neural_network/_multilayer_perceptron.py:690:
ConvergenceWarning: Stochastic Optimizer: Maximum iterations (1000) reached and
the optimization hasn't converged yet.
  warnings.warn(
/projects/proteinml/.links/miniconda3/envs/aidep/lib/python3.10/site-
packages/sklearn/neural_network/_multilayer_perceptron.py:690:
ConvergenceWarning: Stochastic Optimizer: Maximum iterations (1000) reached and
the optimization hasn't converged yet.
  warnings.warn(
/projects/proteinml/.links/miniconda3/envs/aidep/lib/python3.10/site-
packages/sklearn/neural_network/_multilayer_perceptron.py:690:
ConvergenceWarning: Stochastic Optimizer: Maximum iterations (1000) reached and
the optimization hasn't converged yet.
  warnings.warn(
/projects/proteinml/.links/miniconda3/envs/aidep/lib/python3.10/site-
packages/sklearn/neural_network/_multilayer_perceptron.py:690:
ConvergenceWarning: Stochastic Optimizer: Maximum iterations (1000) reached and
the optimization hasn't converged yet.
  warnings.warn(
/projects/proteinml/.links/miniconda3/envs/aidep/lib/python3.10/site-
packages/sklearn/neural_network/_multilayer_perceptron.py:690:
ConvergenceWarning: Stochastic Optimizer: Maximum iterations (1000) reached and
the optimization hasn't converged yet.
  warnings.warn(
/projects/proteinml/.links/miniconda3/envs/aidep/lib/python3.10/site-
packages/sklearn/neural_network/_multilayer_perceptron.py:690:
```

```
ConvergenceWarning: Stochastic Optimizer: Maximum iterations (1000) reached and
the optimization hasn't converged yet.
  warnings.warn(
/projects/proteinml/.links/miniconda3/envs/aidep/lib/python3.10/site-
packages/sklearn/neural_network/_multilayer_perceptron.py:690:
ConvergenceWarning: Stochastic Optimizer: Maximum iterations (1000) reached and
the optimization hasn't converged yet.
  warnings.warn(
/projects/proteinml/.links/miniconda3/envs/aidep/lib/python3.10/site-
packages/sklearn/neural_network/_multilayer_perceptron.py:690:
ConvergenceWarning: Stochastic Optimizer: Maximum iterations (1000) reached and
the optimization hasn't converged yet.
  warnings.warn(
/projects/proteinml/.links/miniconda3/envs/aidep/lib/python3.10/site-
packages/sklearn/neural_network/_multilayer_perceptron.py:690:
ConvergenceWarning: Stochastic Optimizer: Maximum iterations (1000) reached and
the optimization hasn't converged yet.
  warnings.warn(
/projects/proteinml/.links/miniconda3/envs/aidep/lib/python3.10/site-
packages/sklearn/neural_network/_multilayer_perceptron.py:690:
ConvergenceWarning: Stochastic Optimizer: Maximum iterations (1000) reached and
the optimization hasn't converged yet.
  warnings.warn(
/projects/proteinml/.links/miniconda3/envs/aidep/lib/python3.10/site-
packages/sklearn/neural_network/_multilayer_perceptron.py:690:
ConvergenceWarning: Stochastic Optimizer: Maximum iterations (1000) reached and
the optimization hasn't converged yet.
  warnings.warn(
/projects/proteinml/.links/miniconda3/envs/aidep/lib/python3.10/site-
packages/sklearn/neural_network/_multilayer_perceptron.py:690:
ConvergenceWarning: Stochastic Optimizer: Maximum iterations (1000) reached and
the optimization hasn't converged yet.
  warnings.warn(
/projects/proteinml/.links/miniconda3/envs/aidep/lib/python3.10/site-
packages/sklearn/neural_network/_multilayer_perceptron.py:690:
ConvergenceWarning: Stochastic Optimizer: Maximum iterations (1000) reached and
the optimization hasn't converged yet.
  warnings.warn(
/projects/proteinml/.links/miniconda3/envs/aidep/lib/python3.10/site-
packages/sklearn/neural_network/_multilayer_perceptron.py:690:
ConvergenceWarning: Stochastic Optimizer: Maximum iterations (1000) reached and
the optimization hasn't converged yet.
  warnings.warn(
/projects/proteinml/.links/miniconda3/envs/aidep/lib/python3.10/site-
packages/sklearn/neural_network/_multilayer_perceptron.py:690:
ConvergenceWarning: Stochastic Optimizer: Maximum iterations (1000) reached and
the optimization hasn't converged yet.
  warnings.warn(
```

```
/projects/proteinml/.links/miniconda3/envs/aidep/lib/python3.10/site-
packages/sklearn/neural_network/_multilayer_perceptron.py:690:
ConvergenceWarning: Stochastic Optimizer: Maximum iterations (1000) reached and
the optimization hasn't converged yet.
  warnings.warn(
/projects/proteinml/.links/miniconda3/envs/aidep/lib/python3.10/site-
packages/sklearn/neural_network/_multilayer_perceptron.py:690:
ConvergenceWarning: Stochastic Optimizer: Maximum iterations (1000) reached and
the optimization hasn't converged yet.
  warnings.warn(
/projects/proteinml/.links/miniconda3/envs/aidep/lib/python3.10/site-
packages/sklearn/neural_network/_multilayer_perceptron.py:690:
ConvergenceWarning: Stochastic Optimizer: Maximum iterations (1000) reached and
the optimization hasn't converged yet.
  warnings.warn(
/projects/proteinml/.links/miniconda3/envs/aidep/lib/python3.10/site-
packages/sklearn/neural_network/_multilayer_perceptron.py:690:
ConvergenceWarning: Stochastic Optimizer: Maximum iterations (1000) reached and
the optimization hasn't converged yet.
  warnings.warn(
/projects/proteinml/.links/miniconda3/envs/aidep/lib/python3.10/site-
packages/sklearn/neural_network/_multilayer_perceptron.py:690:
ConvergenceWarning: Stochastic Optimizer: Maximum iterations (1000) reached and
the optimization hasn't converged yet.
  warnings.warn(
/projects/proteinml/.links/miniconda3/envs/aidep/lib/python3.10/site-
packages/sklearn/neural_network/_multilayer_perceptron.py:690:
ConvergenceWarning: Stochastic Optimizer: Maximum iterations (1000) reached and
the optimization hasn't converged yet.
  warnings.warn(
/projects/proteinml/.links/miniconda3/envs/aidep/lib/python3.10/site-
packages/sklearn/neural_network/_multilayer_perceptron.py:690:
ConvergenceWarning: Stochastic Optimizer: Maximum iterations (1000) reached and
the optimization hasn't converged yet.
  warnings.warn(
/projects/proteinml/.links/miniconda3/envs/aidep/lib/python3.10/site-
packages/sklearn/neural_network/_multilayer_perceptron.py:690:
ConvergenceWarning: Stochastic Optimizer: Maximum iterations (1000) reached and
the optimization hasn't converged yet.
  warnings.warn(
/projects/proteinml/.links/miniconda3/envs/aidep/lib/python3.10/site-
packages/sklearn/neural_network/_multilayer_perceptron.py:690:
ConvergenceWarning: Stochastic Optimizer: Maximum iterations (1000) reached and
the optimization hasn't converged yet.
  warnings.warn(
/projects/proteinml/.links/miniconda3/envs/aidep/lib/python3.10/site-
packages/sklearn/neural_network/_multilayer_perceptron.py:690:
ConvergenceWarning: Stochastic Optimizer: Maximum iterations (1000) reached and
```

```
the optimization hasn't converged yet.
  warnings.warn(
/projects/proteinml/.links/miniconda3/envs/aidep/lib/python3.10/site-
packages/sklearn/neural_network/_multilayer_perceptron.py:690:
ConvergenceWarning: Stochastic Optimizer: Maximum iterations (1000) reached and
the optimization hasn't converged yet.
  warnings.warn(
/projects/proteinml/.links/miniconda3/envs/aidep/lib/python3.10/site-
packages/sklearn/neural_network/_multilayer_perceptron.py:690:
ConvergenceWarning: Stochastic Optimizer: Maximum iterations (1000) reached and
the optimization hasn't converged yet.
  warnings.warn(
/projects/proteinml/.links/miniconda3/envs/aidep/lib/python3.10/site-
packages/sklearn/neural_network/_multilayer_perceptron.py:690:
ConvergenceWarning: Stochastic Optimizer: Maximum iterations (1000) reached and
the optimization hasn't converged yet.
  warnings.warn(
/projects/proteinml/.links/miniconda3/envs/aidep/lib/python3.10/site-
packages/sklearn/neural_network/_multilayer_perceptron.py:690:
ConvergenceWarning: Stochastic Optimizer: Maximum iterations (1000) reached and
the optimization hasn't converged yet.
  warnings.warn(
/projects/proteinml/.links/miniconda3/envs/aidep/lib/python3.10/site-
packages/sklearn/neural_network/_multilayer_perceptron.py:690:
ConvergenceWarning: Stochastic Optimizer: Maximum iterations (1000) reached and
the optimization hasn't converged yet.
  warnings.warn(
/projects/proteinml/.links/miniconda3/envs/aidep/lib/python3.10/site-
packages/sklearn/neural_network/_multilayer_perceptron.py:690:
ConvergenceWarning: Stochastic Optimizer: Maximum iterations (1000) reached and
the optimization hasn't converged yet.
  warnings.warn(
/projects/proteinml/.links/miniconda3/envs/aidep/lib/python3.10/site-
packages/sklearn/neural_network/_multilayer_perceptron.py:690:
ConvergenceWarning: Stochastic Optimizer: Maximum iterations (1000) reached and
the optimization hasn't converged yet.
  warnings.warn(
/projects/proteinml/.links/miniconda3/envs/aidep/lib/python3.10/site-
packages/sklearn/neural_network/_multilayer_perceptron.py:690:
ConvergenceWarning: Stochastic Optimizer: Maximum iterations (1000) reached and
the optimization hasn't converged yet.
  warnings.warn(
/projects/proteinml/.links/miniconda3/envs/aidep/lib/python3.10/site-
packages/sklearn/neural_network/_multilayer_perceptron.py:690:
ConvergenceWarning: Stochastic Optimizer: Maximum iterations (1000) reached and
the optimization hasn't converged yet.
  warnings.warn(
/projects/proteinml/.links/miniconda3/envs/aidep/lib/python3.10/site-
```

```
packages/sklearn/neural_network/_multilayer_perceptron.py:690:
ConvergenceWarning: Stochastic Optimizer: Maximum iterations (1000) reached and
the optimization hasn't converged yet.
  warnings.warn(
/projects/proteinml/.links/miniconda3/envs/aidep/lib/python3.10/site-
packages/sklearn/neural_network/_multilayer_perceptron.py:690:
ConvergenceWarning: Stochastic Optimizer: Maximum iterations (1000) reached and
the optimization hasn't converged yet.
  warnings.warn(
/projects/proteinml/.links/miniconda3/envs/aidep/lib/python3.10/site-
packages/sklearn/neural_network/_multilayer_perceptron.py:690:
ConvergenceWarning: Stochastic Optimizer: Maximum iterations (1000) reached and
the optimization hasn't converged yet.
  warnings.warn(
/projects/proteinml/.links/miniconda3/envs/aidep/lib/python3.10/site-
packages/sklearn/neural_network/_multilayer_perceptron.py:690:
ConvergenceWarning: Stochastic Optimizer: Maximum iterations (1000) reached and
the optimization hasn't converged yet.
  warnings.warn(
/projects/proteinml/.links/miniconda3/envs/aidep/lib/python3.10/site-
packages/sklearn/neural_network/_multilayer_perceptron.py:690:
ConvergenceWarning: Stochastic Optimizer: Maximum iterations (1000) reached and
the optimization hasn't converged yet.
  warnings.warn(
/projects/proteinml/.links/miniconda3/envs/aidep/lib/python3.10/site-
packages/sklearn/neural_network/_multilayer_perceptron.py:690:
ConvergenceWarning: Stochastic Optimizer: Maximum iterations (1000) reached and
the optimization hasn't converged yet.
  warnings.warn(
/projects/proteinml/.links/miniconda3/envs/aidep/lib/python3.10/site-
packages/sklearn/neural_network/_multilayer_perceptron.py:690:
ConvergenceWarning: Stochastic Optimizer: Maximum iterations (1000) reached and
the optimization hasn't converged yet.
  warnings.warn(
/projects/proteinml/.links/miniconda3/envs/aidep/lib/python3.10/site-
packages/sklearn/neural_network/_multilayer_perceptron.py:690:
ConvergenceWarning: Stochastic Optimizer: Maximum iterations (1000) reached and
the optimization hasn't converged yet.
  warnings.warn(
/projects/proteinml/.links/miniconda3/envs/aidep/lib/python3.10/site-
packages/sklearn/neural_network/_multilayer_perceptron.py:690:
ConvergenceWarning: Stochastic Optimizer: Maximum iterations (1000) reached and
the optimization hasn't converged yet.
  warnings.warn(
/projects/proteinml/.links/miniconda3/envs/aidep/lib/python3.10/site-
packages/sklearn/neural_network/_multilayer_perceptron.py:690:
ConvergenceWarning: Stochastic Optimizer: Maximum iterations (1000) reached and
the optimization hasn't converged yet.
```

```
  warnings.warn(
/projects/proteinml/.links/miniconda3/envs/aidep/lib/python3.10/site-
packages/sklearn/neural_network/_multilayer_perceptron.py:690:
ConvergenceWarning: Stochastic Optimizer: Maximum iterations (1000) reached and
the optimization hasn't converged yet.
  warnings.warn(
/projects/proteinml/.links/miniconda3/envs/aidep/lib/python3.10/site-
packages/sklearn/neural_network/_multilayer_perceptron.py:690:
ConvergenceWarning: Stochastic Optimizer: Maximum iterations (1000) reached and
the optimization hasn't converged yet.
  warnings.warn(
/projects/proteinml/.links/miniconda3/envs/aidep/lib/python3.10/site-
packages/sklearn/neural_network/_multilayer_perceptron.py:690:
ConvergenceWarning: Stochastic Optimizer: Maximum iterations (1000) reached and
the optimization hasn't converged yet.
  warnings.warn(
/projects/proteinml/.links/miniconda3/envs/aidep/lib/python3.10/site-
packages/sklearn/neural_network/_multilayer_perceptron.py:690:
ConvergenceWarning: Stochastic Optimizer: Maximum iterations (1000) reached and
the optimization hasn't converged yet.
  warnings.warn(
/projects/proteinml/.links/miniconda3/envs/aidep/lib/python3.10/site-
packages/sklearn/neural_network/_multilayer_perceptron.py:690:
ConvergenceWarning: Stochastic Optimizer: Maximum iterations (1000) reached and
the optimization hasn't converged yet.
  warnings.warn(
/projects/proteinml/.links/miniconda3/envs/aidep/lib/python3.10/site-
packages/sklearn/neural_network/_multilayer_perceptron.py:690:
ConvergenceWarning: Stochastic Optimizer: Maximum iterations (1000) reached and
the optimization hasn't converged yet.
  warnings.warn(
/projects/proteinml/.links/miniconda3/envs/aidep/lib/python3.10/site-
packages/sklearn/neural_network/_multilayer_perceptron.py:690:
ConvergenceWarning: Stochastic Optimizer: Maximum iterations (1000) reached and
the optimization hasn't converged yet.
  warnings.warn(
/projects/proteinml/.links/miniconda3/envs/aidep/lib/python3.10/site-
packages/sklearn/neural_network/_multilayer_perceptron.py:690:
ConvergenceWarning: Stochastic Optimizer: Maximum iterations (1000) reached and
the optimization hasn't converged yet.
  warnings.warn(
/projects/proteinml/.links/miniconda3/envs/aidep/lib/python3.10/site-
packages/sklearn/neural_network/_multilayer_perceptron.py:690:
ConvergenceWarning: Stochastic Optimizer: Maximum iterations (1000) reached and
the optimization hasn't converged yet.
  warnings.warn(
/projects/proteinml/.links/miniconda3/envs/aidep/lib/python3.10/site-
packages/sklearn/neural_network/_multilayer_perceptron.py:690:
```

```
ConvergenceWarning: Stochastic Optimizer: Maximum iterations (1000) reached and
the optimization hasn't converged yet.
  warnings.warn(
/projects/proteinml/.links/miniconda3/envs/aidep/lib/python3.10/site-
packages/sklearn/neural_network/_multilayer_perceptron.py:690:
ConvergenceWarning: Stochastic Optimizer: Maximum iterations (1000) reached and
the optimization hasn't converged yet.
  warnings.warn(
/projects/proteinml/.links/miniconda3/envs/aidep/lib/python3.10/site-
packages/sklearn/neural_network/_multilayer_perceptron.py:690:
ConvergenceWarning: Stochastic Optimizer: Maximum iterations (1000) reached and
the optimization hasn't converged yet.
  warnings.warn(
/projects/proteinml/.links/miniconda3/envs/aidep/lib/python3.10/site-
packages/sklearn/neural_network/_multilayer_perceptron.py:690:
ConvergenceWarning: Stochastic Optimizer: Maximum iterations (1000) reached and
the optimization hasn't converged yet.
  warnings.warn(
/projects/proteinml/.links/miniconda3/envs/aidep/lib/python3.10/site-
packages/sklearn/neural_network/_multilayer_perceptron.py:690:
ConvergenceWarning: Stochastic Optimizer: Maximum iterations (1000) reached and
the optimization hasn't converged yet.
  warnings.warn(
/projects/proteinml/.links/miniconda3/envs/aidep/lib/python3.10/site-
packages/sklearn/neural_network/_multilayer_perceptron.py:690:
ConvergenceWarning: Stochastic Optimizer: Maximum iterations (1000) reached and
the optimization hasn't converged yet.
  warnings.warn(
/projects/proteinml/.links/miniconda3/envs/aidep/lib/python3.10/site-
packages/sklearn/neural_network/_multilayer_perceptron.py:690:
ConvergenceWarning: Stochastic Optimizer: Maximum iterations (1000) reached and
the optimization hasn't converged yet.
  warnings.warn(
/projects/proteinml/.links/miniconda3/envs/aidep/lib/python3.10/site-
packages/sklearn/neural_network/_multilayer_perceptron.py:690:
ConvergenceWarning: Stochastic Optimizer: Maximum iterations (1000) reached and
the optimization hasn't converged yet.
  warnings.warn(
/projects/proteinml/.links/miniconda3/envs/aidep/lib/python3.10/site-
packages/sklearn/neural_network/_multilayer_perceptron.py:690:
ConvergenceWarning: Stochastic Optimizer: Maximum iterations (1000) reached and
the optimization hasn't converged yet.
  warnings.warn(
/projects/proteinml/.links/miniconda3/envs/aidep/lib/python3.10/site-
packages/sklearn/neural_network/_multilayer_perceptron.py:690:
ConvergenceWarning: Stochastic Optimizer: Maximum iterations (1000) reached and
the optimization hasn't converged yet.
  warnings.warn(
```

```
/projects/proteinml/.links/miniconda3/envs/aidep/lib/python3.10/site-
packages/sklearn/neural_network/_multilayer_perceptron.py:690:
ConvergenceWarning: Stochastic Optimizer: Maximum iterations (1000) reached and
the optimization hasn't converged yet.
  warnings.warn(
/projects/proteinml/.links/miniconda3/envs/aidep/lib/python3.10/site-
packages/sklearn/neural_network/_multilayer_perceptron.py:690:
ConvergenceWarning: Stochastic Optimizer: Maximum iterations (1000) reached and
the optimization hasn't converged yet.
  warnings.warn(
/projects/proteinml/.links/miniconda3/envs/aidep/lib/python3.10/site-
packages/sklearn/neural_network/_multilayer_perceptron.py:690:
ConvergenceWarning: Stochastic Optimizer: Maximum iterations (1000) reached and
the optimization hasn't converged yet.
  warnings.warn(
/projects/proteinml/.links/miniconda3/envs/aidep/lib/python3.10/site-
packages/sklearn/neural_network/_multilayer_perceptron.py:690:
ConvergenceWarning: Stochastic Optimizer: Maximum iterations (1000) reached and
the optimization hasn't converged yet.
  warnings.warn(
/projects/proteinml/.links/miniconda3/envs/aidep/lib/python3.10/site-
packages/sklearn/neural_network/_multilayer_perceptron.py:690:
ConvergenceWarning: Stochastic Optimizer: Maximum iterations (1000) reached and
the optimization hasn't converged yet.
  warnings.warn(
/projects/proteinml/.links/miniconda3/envs/aidep/lib/python3.10/site-
packages/sklearn/neural_network/_multilayer_perceptron.py:690:
ConvergenceWarning: Stochastic Optimizer: Maximum iterations (1000) reached and
the optimization hasn't converged yet.
  warnings.warn(
/projects/proteinml/.links/miniconda3/envs/aidep/lib/python3.10/site-
packages/sklearn/neural_network/_multilayer_perceptron.py:690:
ConvergenceWarning: Stochastic Optimizer: Maximum iterations (1000) reached and
the optimization hasn't converged yet.
  warnings.warn(
/projects/proteinml/.links/miniconda3/envs/aidep/lib/python3.10/site-
packages/sklearn/neural_network/_multilayer_perceptron.py:690:
ConvergenceWarning: Stochastic Optimizer: Maximum iterations (1000) reached and
the optimization hasn't converged yet.
  warnings.warn(
/projects/proteinml/.links/miniconda3/envs/aidep/lib/python3.10/site-
packages/sklearn/neural_network/_multilayer_perceptron.py:690:
ConvergenceWarning: Stochastic Optimizer: Maximum iterations (1000) reached and
the optimization hasn't converged yet.
  warnings.warn(
/projects/proteinml/.links/miniconda3/envs/aidep/lib/python3.10/site-
packages/sklearn/neural_network/_multilayer_perceptron.py:690:
ConvergenceWarning: Stochastic Optimizer: Maximum iterations (1000) reached and
```

```
the optimization hasn't converged yet.
  warnings.warn(
/projects/proteinml/.links/miniconda3/envs/aidep/lib/python3.10/site-
packages/sklearn/neural_network/_multilayer_perceptron.py:690:
ConvergenceWarning: Stochastic Optimizer: Maximum iterations (1000) reached and
the optimization hasn't converged yet.
  warnings.warn(
/projects/proteinml/.links/miniconda3/envs/aidep/lib/python3.10/site-
packages/sklearn/neural_network/_multilayer_perceptron.py:690:
ConvergenceWarning: Stochastic Optimizer: Maximum iterations (1000) reached and
the optimization hasn't converged yet.
  warnings.warn(
/projects/proteinml/.links/miniconda3/envs/aidep/lib/python3.10/site-
packages/sklearn/neural_network/_multilayer_perceptron.py:690:
ConvergenceWarning: Stochastic Optimizer: Maximum iterations (1000) reached and
the optimization hasn't converged yet.
  warnings.warn(
/projects/proteinml/.links/miniconda3/envs/aidep/lib/python3.10/site-
packages/sklearn/neural_network/_multilayer_perceptron.py:690:
ConvergenceWarning: Stochastic Optimizer: Maximum iterations (1000) reached and
the optimization hasn't converged yet.
  warnings.warn(
/projects/proteinml/.links/miniconda3/envs/aidep/lib/python3.10/site-
packages/sklearn/neural_network/_multilayer_perceptron.py:690:
ConvergenceWarning: Stochastic Optimizer: Maximum iterations (1000) reached and
the optimization hasn't converged yet.
  warnings.warn(
/projects/proteinml/.links/miniconda3/envs/aidep/lib/python3.10/site-
packages/sklearn/neural_network/_multilayer_perceptron.py:690:
ConvergenceWarning: Stochastic Optimizer: Maximum iterations (1000) reached and
the optimization hasn't converged yet.
  warnings.warn(
/projects/proteinml/.links/miniconda3/envs/aidep/lib/python3.10/site-
packages/sklearn/neural_network/_multilayer_perceptron.py:690:
ConvergenceWarning: Stochastic Optimizer: Maximum iterations (1000) reached and
the optimization hasn't converged yet.
  warnings.warn(
/projects/proteinml/.links/miniconda3/envs/aidep/lib/python3.10/site-
packages/sklearn/neural_network/_multilayer_perceptron.py:690:
ConvergenceWarning: Stochastic Optimizer: Maximum iterations (1000) reached and
the optimization hasn't converged yet.
  warnings.warn(
/projects/proteinml/.links/miniconda3/envs/aidep/lib/python3.10/site-
packages/sklearn/neural_network/_multilayer_perceptron.py:690:
ConvergenceWarning: Stochastic Optimizer: Maximum iterations (1000) reached and
the optimization hasn't converged yet.
  warnings.warn(
/projects/proteinml/.links/miniconda3/envs/aidep/lib/python3.10/site-
```

```
packages/sklearn/neural_network/_multilayer_perceptron.py:690:
ConvergenceWarning: Stochastic Optimizer: Maximum iterations (1000) reached and
the optimization hasn't converged yet.
  warnings.warn(
/projects/proteinml/.links/miniconda3/envs/aidep/lib/python3.10/site-
packages/sklearn/neural_network/_multilayer_perceptron.py:690:
ConvergenceWarning: Stochastic Optimizer: Maximum iterations (1000) reached and
the optimization hasn't converged yet.
  warnings.warn(
/projects/proteinml/.links/miniconda3/envs/aidep/lib/python3.10/site-
packages/sklearn/neural_network/_multilayer_perceptron.py:690:
ConvergenceWarning: Stochastic Optimizer: Maximum iterations (1000) reached and
the optimization hasn't converged yet.
  warnings.warn(
/projects/proteinml/.links/miniconda3/envs/aidep/lib/python3.10/site-
packages/sklearn/neural_network/_multilayer_perceptron.py:690:
ConvergenceWarning: Stochastic Optimizer: Maximum iterations (1000) reached and
the optimization hasn't converged yet.
  warnings.warn(
/projects/proteinml/.links/miniconda3/envs/aidep/lib/python3.10/site-
packages/sklearn/neural_network/_multilayer_perceptron.py:690:
ConvergenceWarning: Stochastic Optimizer: Maximum iterations (1000) reached and
the optimization hasn't converged yet.
  warnings.warn(
/projects/proteinml/.links/miniconda3/envs/aidep/lib/python3.10/site-
packages/sklearn/neural_network/_multilayer_perceptron.py:690:
ConvergenceWarning: Stochastic Optimizer: Maximum iterations (1000) reached and
the optimization hasn't converged yet.
  warnings.warn(
/projects/proteinml/.links/miniconda3/envs/aidep/lib/python3.10/site-
packages/sklearn/neural_network/_multilayer_perceptron.py:690:
ConvergenceWarning: Stochastic Optimizer: Maximum iterations (1000) reached and
the optimization hasn't converged yet.
  warnings.warn(
/projects/proteinml/.links/miniconda3/envs/aidep/lib/python3.10/site-
packages/sklearn/neural_network/_multilayer_perceptron.py:690:
ConvergenceWarning: Stochastic Optimizer: Maximum iterations (1000) reached and
the optimization hasn't converged yet.
  warnings.warn(
/projects/proteinml/.links/miniconda3/envs/aidep/lib/python3.10/site-
packages/sklearn/neural_network/_multilayer_perceptron.py:690:
ConvergenceWarning: Stochastic Optimizer: Maximum iterations (1000) reached and
the optimization hasn't converged yet.
  warnings.warn(
/projects/proteinml/.links/miniconda3/envs/aidep/lib/python3.10/site-
packages/sklearn/neural_network/_multilayer_perceptron.py:690:
ConvergenceWarning: Stochastic Optimizer: Maximum iterations (1000) reached and
the optimization hasn't converged yet.
```

```
  warnings.warn(
/projects/proteinml/.links/miniconda3/envs/aidep/lib/python3.10/site-
packages/sklearn/neural_network/_multilayer_perceptron.py:690:
ConvergenceWarning: Stochastic Optimizer: Maximum iterations (1000) reached and
the optimization hasn't converged yet.
  warnings.warn(
/projects/proteinml/.links/miniconda3/envs/aidep/lib/python3.10/site-
packages/sklearn/neural_network/_multilayer_perceptron.py:690:
ConvergenceWarning: Stochastic Optimizer: Maximum iterations (1000) reached and
the optimization hasn't converged yet.
  warnings.warn(
/projects/proteinml/.links/miniconda3/envs/aidep/lib/python3.10/site-
packages/sklearn/neural_network/_multilayer_perceptron.py:690:
ConvergenceWarning: Stochastic Optimizer: Maximum iterations (1000) reached and
the optimization hasn't converged yet.
  warnings.warn(
/projects/proteinml/.links/miniconda3/envs/aidep/lib/python3.10/site-
packages/sklearn/neural_network/_multilayer_perceptron.py:690:
ConvergenceWarning: Stochastic Optimizer: Maximum iterations (1000) reached and
the optimization hasn't converged yet.
  warnings.warn(
/projects/proteinml/.links/miniconda3/envs/aidep/lib/python3.10/site-
packages/sklearn/neural_network/_multilayer_perceptron.py:690:
ConvergenceWarning: Stochastic Optimizer: Maximum iterations (1000) reached and
the optimization hasn't converged yet.
  warnings.warn(
/projects/proteinml/.links/miniconda3/envs/aidep/lib/python3.10/site-
packages/sklearn/neural_network/_multilayer_perceptron.py:690:
ConvergenceWarning: Stochastic Optimizer: Maximum iterations (1000) reached and
the optimization hasn't converged yet.
  warnings.warn(
/projects/proteinml/.links/miniconda3/envs/aidep/lib/python3.10/site-
packages/sklearn/neural_network/_multilayer_perceptron.py:690:
ConvergenceWarning: Stochastic Optimizer: Maximum iterations (1000) reached and
the optimization hasn't converged yet.
  warnings.warn(
/projects/proteinml/.links/miniconda3/envs/aidep/lib/python3.10/site-
packages/sklearn/neural_network/_multilayer_perceptron.py:690:
ConvergenceWarning: Stochastic Optimizer: Maximum iterations (1000) reached and
the optimization hasn't converged yet.
  warnings.warn(
/projects/proteinml/.links/miniconda3/envs/aidep/lib/python3.10/site-
packages/sklearn/neural_network/_multilayer_perceptron.py:690:
ConvergenceWarning: Stochastic Optimizer: Maximum iterations (1000) reached and
the optimization hasn't converged yet.
  warnings.warn(
/projects/proteinml/.links/miniconda3/envs/aidep/lib/python3.10/site-
packages/sklearn/neural_network/_multilayer_perceptron.py:690:
```

```
ConvergenceWarning: Stochastic Optimizer: Maximum iterations (1000) reached and
the optimization hasn't converged yet.
  warnings.warn(
/projects/proteinml/.links/miniconda3/envs/aidep/lib/python3.10/site-
packages/sklearn/neural_network/_multilayer_perceptron.py:690:
ConvergenceWarning: Stochastic Optimizer: Maximum iterations (1000) reached and
the optimization hasn't converged yet.
  warnings.warn(
/projects/proteinml/.links/miniconda3/envs/aidep/lib/python3.10/site-
packages/sklearn/neural_network/_multilayer_perceptron.py:690:
ConvergenceWarning: Stochastic Optimizer: Maximum iterations (1000) reached and
the optimization hasn't converged yet.
  warnings.warn(
/projects/proteinml/.links/miniconda3/envs/aidep/lib/python3.10/site-
packages/sklearn/neural_network/_multilayer_perceptron.py:690:
ConvergenceWarning: Stochastic Optimizer: Maximum iterations (1000) reached and
the optimization hasn't converged yet.
  warnings.warn(
/projects/proteinml/.links/miniconda3/envs/aidep/lib/python3.10/site-
packages/sklearn/neural_network/_multilayer_perceptron.py:690:
ConvergenceWarning: Stochastic Optimizer: Maximum iterations (1000) reached and
the optimization hasn't converged yet.
  warnings.warn(
/projects/proteinml/.links/miniconda3/envs/aidep/lib/python3.10/site-
packages/sklearn/neural_network/_multilayer_perceptron.py:690:
ConvergenceWarning: Stochastic Optimizer: Maximum iterations (1000) reached and
the optimization hasn't converged yet.
  warnings.warn(
/projects/proteinml/.links/miniconda3/envs/aidep/lib/python3.10/site-
packages/sklearn/neural_network/_multilayer_perceptron.py:690:
ConvergenceWarning: Stochastic Optimizer: Maximum iterations (1000) reached and
the optimization hasn't converged yet.
  warnings.warn(
/projects/proteinml/.links/miniconda3/envs/aidep/lib/python3.10/site-
packages/sklearn/neural_network/_multilayer_perceptron.py:690:
ConvergenceWarning: Stochastic Optimizer: Maximum iterations (1000) reached and
the optimization hasn't converged yet.
  warnings.warn(
/projects/proteinml/.links/miniconda3/envs/aidep/lib/python3.10/site-
packages/sklearn/neural_network/_multilayer_perceptron.py:690:
ConvergenceWarning: Stochastic Optimizer: Maximum iterations (1000) reached and
the optimization hasn't converged yet.
  warnings.warn(
/projects/proteinml/.links/miniconda3/envs/aidep/lib/python3.10/site-
packages/sklearn/neural_network/_multilayer_perceptron.py:690:
ConvergenceWarning: Stochastic Optimizer: Maximum iterations (1000) reached and
the optimization hasn't converged yet.
  warnings.warn(
```

```
/projects/proteinml/.links/miniconda3/envs/aidep/lib/python3.10/site-
packages/sklearn/neural_network/_multilayer_perceptron.py:690:
ConvergenceWarning: Stochastic Optimizer: Maximum iterations (1000) reached and
the optimization hasn't converged yet.
  warnings.warn(
/projects/proteinml/.links/miniconda3/envs/aidep/lib/python3.10/site-
packages/sklearn/neural_network/_multilayer_perceptron.py:690:
ConvergenceWarning: Stochastic Optimizer: Maximum iterations (1000) reached and
the optimization hasn't converged yet.
  warnings.warn(
/projects/proteinml/.links/miniconda3/envs/aidep/lib/python3.10/site-
packages/sklearn/neural_network/_multilayer_perceptron.py:690:
ConvergenceWarning: Stochastic Optimizer: Maximum iterations (1000) reached and
the optimization hasn't converged yet.
  warnings.warn(
/projects/proteinml/.links/miniconda3/envs/aidep/lib/python3.10/site-
packages/sklearn/neural_network/_multilayer_perceptron.py:690:
ConvergenceWarning: Stochastic Optimizer: Maximum iterations (1000) reached and
the optimization hasn't converged yet.
  warnings.warn(
/projects/proteinml/.links/miniconda3/envs/aidep/lib/python3.10/site-
packages/sklearn/neural_network/_multilayer_perceptron.py:690:
ConvergenceWarning: Stochastic Optimizer: Maximum iterations (1000) reached and
the optimization hasn't converged yet.
  warnings.warn(
/projects/proteinml/.links/miniconda3/envs/aidep/lib/python3.10/site-
packages/sklearn/neural_network/_multilayer_perceptron.py:690:
ConvergenceWarning: Stochastic Optimizer: Maximum iterations (1000) reached and
the optimization hasn't converged yet.
  warnings.warn(
/projects/proteinml/.links/miniconda3/envs/aidep/lib/python3.10/site-
packages/sklearn/neural_network/_multilayer_perceptron.py:690:
ConvergenceWarning: Stochastic Optimizer: Maximum iterations (1000) reached and
the optimization hasn't converged yet.
  warnings.warn(
/projects/proteinml/.links/miniconda3/envs/aidep/lib/python3.10/site-
packages/sklearn/neural_network/_multilayer_perceptron.py:690:
ConvergenceWarning: Stochastic Optimizer: Maximum iterations (1000) reached and
the optimization hasn't converged yet.
  warnings.warn(
/projects/proteinml/.links/miniconda3/envs/aidep/lib/python3.10/site-
packages/sklearn/neural_network/_multilayer_perceptron.py:690:
ConvergenceWarning: Stochastic Optimizer: Maximum iterations (1000) reached and
the optimization hasn't converged yet.
  warnings.warn(
/projects/proteinml/.links/miniconda3/envs/aidep/lib/python3.10/site-
packages/sklearn/neural_network/_multilayer_perceptron.py:690:
ConvergenceWarning: Stochastic Optimizer: Maximum iterations (1000) reached and
```

```
the optimization hasn't converged yet.
  warnings.warn(
/projects/proteinml/.links/miniconda3/envs/aidep/lib/python3.10/site-
packages/sklearn/neural_network/_multilayer_perceptron.py:690:
ConvergenceWarning: Stochastic Optimizer: Maximum iterations (1000) reached and
the optimization hasn't converged yet.
  warnings.warn(
/projects/proteinml/.links/miniconda3/envs/aidep/lib/python3.10/site-
packages/sklearn/neural_network/_multilayer_perceptron.py:690:
ConvergenceWarning: Stochastic Optimizer: Maximum iterations (1000) reached and
the optimization hasn't converged yet.
  warnings.warn(
/projects/proteinml/.links/miniconda3/envs/aidep/lib/python3.10/site-
packages/sklearn/neural_network/_multilayer_perceptron.py:690:
ConvergenceWarning: Stochastic Optimizer: Maximum iterations (1000) reached and
the optimization hasn't converged yet.
  warnings.warn(
/projects/proteinml/.links/miniconda3/envs/aidep/lib/python3.10/site-
packages/sklearn/neural_network/_multilayer_perceptron.py:690:
ConvergenceWarning: Stochastic Optimizer: Maximum iterations (1000) reached and
the optimization hasn't converged yet.
  warnings.warn(
/projects/proteinml/.links/miniconda3/envs/aidep/lib/python3.10/site-
packages/sklearn/neural_network/_multilayer_perceptron.py:690:
ConvergenceWarning: Stochastic Optimizer: Maximum iterations (1000) reached and
the optimization hasn't converged yet.
  warnings.warn(
/projects/proteinml/.links/miniconda3/envs/aidep/lib/python3.10/site-
packages/sklearn/neural_network/_multilayer_perceptron.py:690:
ConvergenceWarning: Stochastic Optimizer: Maximum iterations (1000) reached and
the optimization hasn't converged yet.
  warnings.warn(
/projects/proteinml/.links/miniconda3/envs/aidep/lib/python3.10/site-
packages/sklearn/neural_network/_multilayer_perceptron.py:690:
ConvergenceWarning: Stochastic Optimizer: Maximum iterations (1000) reached and
the optimization hasn't converged yet.
  warnings.warn(
/projects/proteinml/.links/miniconda3/envs/aidep/lib/python3.10/site-
packages/sklearn/neural_network/_multilayer_perceptron.py:690:
ConvergenceWarning: Stochastic Optimizer: Maximum iterations (1000) reached and
the optimization hasn't converged yet.
  warnings.warn(
/projects/proteinml/.links/miniconda3/envs/aidep/lib/python3.10/site-
packages/sklearn/neural_network/_multilayer_perceptron.py:690:
ConvergenceWarning: Stochastic Optimizer: Maximum iterations (1000) reached and
the optimization hasn't converged yet.
  warnings.warn(
/projects/proteinml/.links/miniconda3/envs/aidep/lib/python3.10/site-
```

```
packages/sklearn/neural_network/_multilayer_perceptron.py:690:
ConvergenceWarning: Stochastic Optimizer: Maximum iterations (1000) reached and
the optimization hasn't converged yet.
  warnings.warn(
/projects/proteinml/.links/miniconda3/envs/aidep/lib/python3.10/site-
packages/sklearn/neural_network/_multilayer_perceptron.py:690:
ConvergenceWarning: Stochastic Optimizer: Maximum iterations (1000) reached and
the optimization hasn't converged yet.
  warnings.warn(
/projects/proteinml/.links/miniconda3/envs/aidep/lib/python3.10/site-
packages/sklearn/neural_network/_multilayer_perceptron.py:690:
ConvergenceWarning: Stochastic Optimizer: Maximum iterations (1000) reached and
the optimization hasn't converged yet.
  warnings.warn(
/projects/proteinml/.links/miniconda3/envs/aidep/lib/python3.10/site-
packages/sklearn/neural_network/_multilayer_perceptron.py:690:
ConvergenceWarning: Stochastic Optimizer: Maximum iterations (1000) reached and
the optimization hasn't converged yet.
  warnings.warn(
/projects/proteinml/.links/miniconda3/envs/aidep/lib/python3.10/site-
packages/sklearn/neural_network/_multilayer_perceptron.py:690:
ConvergenceWarning: Stochastic Optimizer: Maximum iterations (1000) reached and
the optimization hasn't converged yet.
  warnings.warn(
/projects/proteinml/.links/miniconda3/envs/aidep/lib/python3.10/site-
packages/sklearn/neural_network/_multilayer_perceptron.py:690:
ConvergenceWarning: Stochastic Optimizer: Maximum iterations (1000) reached and
the optimization hasn't converged yet.
  warnings.warn(
/projects/proteinml/.links/miniconda3/envs/aidep/lib/python3.10/site-
packages/sklearn/neural_network/_multilayer_perceptron.py:690:
ConvergenceWarning: Stochastic Optimizer: Maximum iterations (1000) reached and
the optimization hasn't converged yet.
  warnings.warn(
/projects/proteinml/.links/miniconda3/envs/aidep/lib/python3.10/site-
packages/sklearn/neural_network/_multilayer_perceptron.py:690:
ConvergenceWarning: Stochastic Optimizer: Maximum iterations (1000) reached and
the optimization hasn't converged yet.
  warnings.warn(
/projects/proteinml/.links/miniconda3/envs/aidep/lib/python3.10/site-
packages/sklearn/neural_network/_multilayer_perceptron.py:690:
ConvergenceWarning: Stochastic Optimizer: Maximum iterations (1000) reached and
the optimization hasn't converged yet.
  warnings.warn(
/projects/proteinml/.links/miniconda3/envs/aidep/lib/python3.10/site-
packages/sklearn/neural_network/_multilayer_perceptron.py:690:
ConvergenceWarning: Stochastic Optimizer: Maximum iterations (1000) reached and
the optimization hasn't converged yet.
```

```
  warnings.warn(
/projects/proteinml/.links/miniconda3/envs/aidep/lib/python3.10/site-
packages/sklearn/neural_network/_multilayer_perceptron.py:690:
ConvergenceWarning: Stochastic Optimizer: Maximum iterations (1000) reached and
the optimization hasn't converged yet.
  warnings.warn(
/projects/proteinml/.links/miniconda3/envs/aidep/lib/python3.10/site-
packages/sklearn/neural_network/_multilayer_perceptron.py:690:
ConvergenceWarning: Stochastic Optimizer: Maximum iterations (1000) reached and
the optimization hasn't converged yet.
  warnings.warn(
/projects/proteinml/.links/miniconda3/envs/aidep/lib/python3.10/site-
packages/sklearn/neural_network/_multilayer_perceptron.py:690:
ConvergenceWarning: Stochastic Optimizer: Maximum iterations (1000) reached and
the optimization hasn't converged yet.
  warnings.warn(
/projects/proteinml/.links/miniconda3/envs/aidep/lib/python3.10/site-
packages/sklearn/neural_network/_multilayer_perceptron.py:690:
ConvergenceWarning: Stochastic Optimizer: Maximum iterations (1000) reached and
the optimization hasn't converged yet.
  warnings.warn(
/projects/proteinml/.links/miniconda3/envs/aidep/lib/python3.10/site-
packages/sklearn/neural_network/_multilayer_perceptron.py:690:
ConvergenceWarning: Stochastic Optimizer: Maximum iterations (1000) reached and
the optimization hasn't converged yet.
  warnings.warn(
/projects/proteinml/.links/miniconda3/envs/aidep/lib/python3.10/site-
packages/sklearn/neural_network/_multilayer_perceptron.py:690:
ConvergenceWarning: Stochastic Optimizer: Maximum iterations (1000) reached and
the optimization hasn't converged yet.
  warnings.warn(
/projects/proteinml/.links/miniconda3/envs/aidep/lib/python3.10/site-
packages/sklearn/neural_network/_multilayer_perceptron.py:690:
ConvergenceWarning: Stochastic Optimizer: Maximum iterations (1000) reached and
the optimization hasn't converged yet.
  warnings.warn(
/projects/proteinml/.links/miniconda3/envs/aidep/lib/python3.10/site-
packages/sklearn/neural_network/_multilayer_perceptron.py:690:
ConvergenceWarning: Stochastic Optimizer: Maximum iterations (1000) reached and
the optimization hasn't converged yet.
  warnings.warn(
/projects/proteinml/.links/miniconda3/envs/aidep/lib/python3.10/site-
packages/sklearn/neural_network/_multilayer_perceptron.py:690:
ConvergenceWarning: Stochastic Optimizer: Maximum iterations (1000) reached and
the optimization hasn't converged yet.
  warnings.warn(
/projects/proteinml/.links/miniconda3/envs/aidep/lib/python3.10/site-
packages/sklearn/neural_network/_multilayer_perceptron.py:690:
```

```
ConvergenceWarning: Stochastic Optimizer: Maximum iterations (1000) reached and
the optimization hasn't converged yet.
  warnings.warn(
/projects/proteinml/.links/miniconda3/envs/aidep/lib/python3.10/site-
packages/sklearn/neural_network/_multilayer_perceptron.py:690:
ConvergenceWarning: Stochastic Optimizer: Maximum iterations (1000) reached and
the optimization hasn't converged yet.
  warnings.warn(
/projects/proteinml/.links/miniconda3/envs/aidep/lib/python3.10/site-
packages/sklearn/neural_network/_multilayer_perceptron.py:690:
ConvergenceWarning: Stochastic Optimizer: Maximum iterations (1000) reached and
the optimization hasn't converged yet.
  warnings.warn(
/projects/proteinml/.links/miniconda3/envs/aidep/lib/python3.10/site-
packages/sklearn/neural_network/_multilayer_perceptron.py:690:
ConvergenceWarning: Stochastic Optimizer: Maximum iterations (1000) reached and
the optimization hasn't converged yet.
  warnings.warn(
/projects/proteinml/.links/miniconda3/envs/aidep/lib/python3.10/site-
packages/sklearn/neural_network/_multilayer_perceptron.py:690:
ConvergenceWarning: Stochastic Optimizer: Maximum iterations (1000) reached and
the optimization hasn't converged yet.
  warnings.warn(
/projects/proteinml/.links/miniconda3/envs/aidep/lib/python3.10/site-
packages/sklearn/neural_network/_multilayer_perceptron.py:690:
ConvergenceWarning: Stochastic Optimizer: Maximum iterations (1000) reached and
the optimization hasn't converged yet.
  warnings.warn(
/projects/proteinml/.links/miniconda3/envs/aidep/lib/python3.10/site-
packages/sklearn/neural_network/_multilayer_perceptron.py:690:
ConvergenceWarning: Stochastic Optimizer: Maximum iterations (1000) reached and
the optimization hasn't converged yet.
  warnings.warn(
/projects/proteinml/.links/miniconda3/envs/aidep/lib/python3.10/site-
packages/sklearn/neural_network/_multilayer_perceptron.py:690:
ConvergenceWarning: Stochastic Optimizer: Maximum iterations (1000) reached and
the optimization hasn't converged yet.
  warnings.warn(
/projects/proteinml/.links/miniconda3/envs/aidep/lib/python3.10/site-
packages/sklearn/neural_network/_multilayer_perceptron.py:690:
ConvergenceWarning: Stochastic Optimizer: Maximum iterations (1000) reached and
the optimization hasn't converged yet.
  warnings.warn(
/projects/proteinml/.links/miniconda3/envs/aidep/lib/python3.10/site-
packages/sklearn/neural_network/_multilayer_perceptron.py:690:
ConvergenceWarning: Stochastic Optimizer: Maximum iterations (1000) reached and
the optimization hasn't converged yet.
  warnings.warn(
```

```
/projects/proteinml/.links/miniconda3/envs/aidep/lib/python3.10/site-
packages/sklearn/neural_network/_multilayer_perceptron.py:690:
ConvergenceWarning: Stochastic Optimizer: Maximum iterations (1000) reached and
the optimization hasn't converged yet.
  warnings.warn(
/projects/proteinml/.links/miniconda3/envs/aidep/lib/python3.10/site-
packages/sklearn/neural_network/_multilayer_perceptron.py:690:
ConvergenceWarning: Stochastic Optimizer: Maximum iterations (1000) reached and
the optimization hasn't converged yet.
  warnings.warn(
/projects/proteinml/.links/miniconda3/envs/aidep/lib/python3.10/site-
packages/sklearn/neural_network/_multilayer_perceptron.py:690:
ConvergenceWarning: Stochastic Optimizer: Maximum iterations (1000) reached and
the optimization hasn't converged yet.
  warnings.warn(
/projects/proteinml/.links/miniconda3/envs/aidep/lib/python3.10/site-
packages/sklearn/neural_network/_multilayer_perceptron.py:690:
ConvergenceWarning: Stochastic Optimizer: Maximum iterations (1000) reached and
the optimization hasn't converged yet.
  warnings.warn(
/projects/proteinml/.links/miniconda3/envs/aidep/lib/python3.10/site-
packages/sklearn/neural_network/_multilayer_perceptron.py:690:
ConvergenceWarning: Stochastic Optimizer: Maximum iterations (1000) reached and
the optimization hasn't converged yet.
  warnings.warn(
/projects/proteinml/.links/miniconda3/envs/aidep/lib/python3.10/site-
packages/sklearn/neural_network/_multilayer_perceptron.py:690:
ConvergenceWarning: Stochastic Optimizer: Maximum iterations (1000) reached and
the optimization hasn't converged yet.
  warnings.warn(
/projects/proteinml/.links/miniconda3/envs/aidep/lib/python3.10/site-
packages/sklearn/neural_network/_multilayer_perceptron.py:690:
ConvergenceWarning: Stochastic Optimizer: Maximum iterations (1000) reached and
the optimization hasn't converged yet.
  warnings.warn(
/projects/proteinml/.links/miniconda3/envs/aidep/lib/python3.10/site-
packages/sklearn/neural_network/_multilayer_perceptron.py:690:
ConvergenceWarning: Stochastic Optimizer: Maximum iterations (1000) reached and
the optimization hasn't converged yet.
  warnings.warn(
/projects/proteinml/.links/miniconda3/envs/aidep/lib/python3.10/site-
packages/sklearn/neural_network/_multilayer_perceptron.py:690:
ConvergenceWarning: Stochastic Optimizer: Maximum iterations (1000) reached and
the optimization hasn't converged yet.
  warnings.warn(
/projects/proteinml/.links/miniconda3/envs/aidep/lib/python3.10/site-
packages/sklearn/neural_network/_multilayer_perceptron.py:690:
ConvergenceWarning: Stochastic Optimizer: Maximum iterations (1000) reached and
```

```
the optimization hasn't converged yet.
  warnings.warn(
/projects/proteinml/.links/miniconda3/envs/aidep/lib/python3.10/site-
packages/sklearn/neural_network/_multilayer_perceptron.py:690:
ConvergenceWarning: Stochastic Optimizer: Maximum iterations (1000) reached and
the optimization hasn't converged yet.
  warnings.warn(
/projects/proteinml/.links/miniconda3/envs/aidep/lib/python3.10/site-
packages/sklearn/neural_network/_multilayer_perceptron.py:690:
ConvergenceWarning: Stochastic Optimizer: Maximum iterations (1000) reached and
the optimization hasn't converged yet.
  warnings.warn(
/projects/proteinml/.links/miniconda3/envs/aidep/lib/python3.10/site-
packages/sklearn/neural_network/_multilayer_perceptron.py:690:
ConvergenceWarning: Stochastic Optimizer: Maximum iterations (1000) reached and
the optimization hasn't converged yet.
  warnings.warn(
/projects/proteinml/.links/miniconda3/envs/aidep/lib/python3.10/site-
packages/sklearn/neural_network/_multilayer_perceptron.py:690:
ConvergenceWarning: Stochastic Optimizer: Maximum iterations (1000) reached and
the optimization hasn't converged yet.
  warnings.warn(
/projects/proteinml/.links/miniconda3/envs/aidep/lib/python3.10/site-
packages/sklearn/neural_network/_multilayer_perceptron.py:690:
ConvergenceWarning: Stochastic Optimizer: Maximum iterations (1000) reached and
the optimization hasn't converged yet.
  warnings.warn(
/projects/proteinml/.links/miniconda3/envs/aidep/lib/python3.10/site-
packages/sklearn/neural_network/_multilayer_perceptron.py:690:
ConvergenceWarning: Stochastic Optimizer: Maximum iterations (1000) reached and
the optimization hasn't converged yet.
  warnings.warn(
/projects/proteinml/.links/miniconda3/envs/aidep/lib/python3.10/site-
packages/sklearn/neural_network/_multilayer_perceptron.py:690:
ConvergenceWarning: Stochastic Optimizer: Maximum iterations (1000) reached and
the optimization hasn't converged yet.
  warnings.warn(
/projects/proteinml/.links/miniconda3/envs/aidep/lib/python3.10/site-
packages/sklearn/neural_network/_multilayer_perceptron.py:690:
ConvergenceWarning: Stochastic Optimizer: Maximum iterations (1000) reached and
the optimization hasn't converged yet.
  warnings.warn(
/projects/proteinml/.links/miniconda3/envs/aidep/lib/python3.10/site-
packages/sklearn/neural_network/_multilayer_perceptron.py:690:
ConvergenceWarning: Stochastic Optimizer: Maximum iterations (1000) reached and
the optimization hasn't converged yet.
  warnings.warn(
/projects/proteinml/.links/miniconda3/envs/aidep/lib/python3.10/site-
```

```
packages/sklearn/neural_network/_multilayer_perceptron.py:690:
ConvergenceWarning: Stochastic Optimizer: Maximum iterations (1000) reached and
the optimization hasn't converged yet.
  warnings.warn(
/projects/proteinml/.links/miniconda3/envs/aidep/lib/python3.10/site-
packages/sklearn/neural_network/_multilayer_perceptron.py:690:
ConvergenceWarning: Stochastic Optimizer: Maximum iterations (1000) reached and
the optimization hasn't converged yet.
  warnings.warn(
/projects/proteinml/.links/miniconda3/envs/aidep/lib/python3.10/site-
packages/sklearn/neural_network/_multilayer_perceptron.py:690:
ConvergenceWarning: Stochastic Optimizer: Maximum iterations (1000) reached and
the optimization hasn't converged yet.
  warnings.warn(
/projects/proteinml/.links/miniconda3/envs/aidep/lib/python3.10/site-
packages/sklearn/neural_network/_multilayer_perceptron.py:690:
ConvergenceWarning: Stochastic Optimizer: Maximum iterations (1000) reached and
the optimization hasn't converged yet.
  warnings.warn(
/projects/proteinml/.links/miniconda3/envs/aidep/lib/python3.10/site-
packages/sklearn/neural_network/_multilayer_perceptron.py:690:
ConvergenceWarning: Stochastic Optimizer: Maximum iterations (1000) reached and
the optimization hasn't converged yet.
  warnings.warn(
/projects/proteinml/.links/miniconda3/envs/aidep/lib/python3.10/site-
packages/sklearn/neural_network/_multilayer_perceptron.py:690:
ConvergenceWarning: Stochastic Optimizer: Maximum iterations (1000) reached and
the optimization hasn't converged yet.
  warnings.warn(
/projects/proteinml/.links/miniconda3/envs/aidep/lib/python3.10/site-
packages/sklearn/neural_network/_multilayer_perceptron.py:690:
ConvergenceWarning: Stochastic Optimizer: Maximum iterations (1000) reached and
the optimization hasn't converged yet.
  warnings.warn(
/projects/proteinml/.links/miniconda3/envs/aidep/lib/python3.10/site-
packages/sklearn/neural_network/_multilayer_perceptron.py:690:
ConvergenceWarning: Stochastic Optimizer: Maximum iterations (1000) reached and
the optimization hasn't converged yet.
  warnings.warn(
/projects/proteinml/.links/miniconda3/envs/aidep/lib/python3.10/site-
packages/sklearn/neural_network/_multilayer_perceptron.py:690:
ConvergenceWarning: Stochastic Optimizer: Maximum iterations (1000) reached and
the optimization hasn't converged yet.
  warnings.warn(
/projects/proteinml/.links/miniconda3/envs/aidep/lib/python3.10/site-
packages/sklearn/neural_network/_multilayer_perceptron.py:690:
ConvergenceWarning: Stochastic Optimizer: Maximum iterations (1000) reached and
the optimization hasn't converged yet.
```

```
  warnings.warn(
/projects/proteinml/.links/miniconda3/envs/aidep/lib/python3.10/site-
packages/sklearn/neural_network/_multilayer_perceptron.py:690:
ConvergenceWarning: Stochastic Optimizer: Maximum iterations (1000) reached and
the optimization hasn't converged yet.
  warnings.warn(
/projects/proteinml/.links/miniconda3/envs/aidep/lib/python3.10/site-
packages/sklearn/neural_network/_multilayer_perceptron.py:690:
ConvergenceWarning: Stochastic Optimizer: Maximum iterations (1000) reached and
the optimization hasn't converged yet.
  warnings.warn(
/projects/proteinml/.links/miniconda3/envs/aidep/lib/python3.10/site-
packages/sklearn/neural_network/_multilayer_perceptron.py:690:
ConvergenceWarning: Stochastic Optimizer: Maximum iterations (1000) reached and
the optimization hasn't converged yet.
  warnings.warn(
/projects/proteinml/.links/miniconda3/envs/aidep/lib/python3.10/site-
packages/sklearn/neural_network/_multilayer_perceptron.py:690:
ConvergenceWarning: Stochastic Optimizer: Maximum iterations (1000) reached and
the optimization hasn't converged yet.
  warnings.warn(
/projects/proteinml/.links/miniconda3/envs/aidep/lib/python3.10/site-
packages/sklearn/neural_network/_multilayer_perceptron.py:690:
ConvergenceWarning: Stochastic Optimizer: Maximum iterations (1000) reached and
the optimization hasn't converged yet.
  warnings.warn(
/projects/proteinml/.links/miniconda3/envs/aidep/lib/python3.10/site-
packages/sklearn/neural_network/_multilayer_perceptron.py:690:
ConvergenceWarning: Stochastic Optimizer: Maximum iterations (1000) reached and
the optimization hasn't converged yet.
  warnings.warn(
/projects/proteinml/.links/miniconda3/envs/aidep/lib/python3.10/site-
packages/sklearn/neural_network/_multilayer_perceptron.py:690:
ConvergenceWarning: Stochastic Optimizer: Maximum iterations (1000) reached and
the optimization hasn't converged yet.
  warnings.warn(
/projects/proteinml/.links/miniconda3/envs/aidep/lib/python3.10/site-
packages/sklearn/neural_network/_multilayer_perceptron.py:690:
ConvergenceWarning: Stochastic Optimizer: Maximum iterations (1000) reached and
the optimization hasn't converged yet.
  warnings.warn(
/projects/proteinml/.links/miniconda3/envs/aidep/lib/python3.10/site-
packages/sklearn/neural_network/_multilayer_perceptron.py:690:
ConvergenceWarning: Stochastic Optimizer: Maximum iterations (1000) reached and
the optimization hasn't converged yet.
  warnings.warn(
/projects/proteinml/.links/miniconda3/envs/aidep/lib/python3.10/site-
packages/sklearn/neural_network/_multilayer_perceptron.py:690:
```

```
ConvergenceWarning: Stochastic Optimizer: Maximum iterations (1000) reached and
the optimization hasn't converged yet.
  warnings.warn(
/projects/proteinml/.links/miniconda3/envs/aidep/lib/python3.10/site-
packages/sklearn/neural_network/_multilayer_perceptron.py:690:
ConvergenceWarning: Stochastic Optimizer: Maximum iterations (1000) reached and
the optimization hasn't converged yet.
  warnings.warn(
/projects/proteinml/.links/miniconda3/envs/aidep/lib/python3.10/site-
packages/sklearn/neural_network/_multilayer_perceptron.py:690:
ConvergenceWarning: Stochastic Optimizer: Maximum iterations (1000) reached and
the optimization hasn't converged yet.
  warnings.warn(
/projects/proteinml/.links/miniconda3/envs/aidep/lib/python3.10/site-
packages/sklearn/neural_network/_multilayer_perceptron.py:690:
ConvergenceWarning: Stochastic Optimizer: Maximum iterations (1000) reached and
the optimization hasn't converged yet.
  warnings.warn(
/projects/proteinml/.links/miniconda3/envs/aidep/lib/python3.10/site-
packages/sklearn/neural_network/_multilayer_perceptron.py:690:
ConvergenceWarning: Stochastic Optimizer: Maximum iterations (1000) reached and
the optimization hasn't converged yet.
  warnings.warn(
/projects/proteinml/.links/miniconda3/envs/aidep/lib/python3.10/site-
packages/sklearn/neural_network/_multilayer_perceptron.py:690:
ConvergenceWarning: Stochastic Optimizer: Maximum iterations (1000) reached and
the optimization hasn't converged yet.
  warnings.warn(
/projects/proteinml/.links/miniconda3/envs/aidep/lib/python3.10/site-
packages/sklearn/neural_network/_multilayer_perceptron.py:690:
ConvergenceWarning: Stochastic Optimizer: Maximum iterations (1000) reached and
the optimization hasn't converged yet.
  warnings.warn(
/projects/proteinml/.links/miniconda3/envs/aidep/lib/python3.10/site-
packages/sklearn/neural_network/_multilayer_perceptron.py:690:
ConvergenceWarning: Stochastic Optimizer: Maximum iterations (1000) reached and
the optimization hasn't converged yet.
  warnings.warn(
/projects/proteinml/.links/miniconda3/envs/aidep/lib/python3.10/site-
packages/sklearn/neural_network/_multilayer_perceptron.py:690:
ConvergenceWarning: Stochastic Optimizer: Maximum iterations (1000) reached and
the optimization hasn't converged yet.
  warnings.warn(
/projects/proteinml/.links/miniconda3/envs/aidep/lib/python3.10/site-
packages/sklearn/neural_network/_multilayer_perceptron.py:690:
ConvergenceWarning: Stochastic Optimizer: Maximum iterations (1000) reached and
the optimization hasn't converged yet.
  warnings.warn(
```

```
/projects/proteinml/.links/miniconda3/envs/aidep/lib/python3.10/site-
packages/sklearn/neural_network/_multilayer_perceptron.py:690:
ConvergenceWarning: Stochastic Optimizer: Maximum iterations (1000) reached and
the optimization hasn't converged yet.
  warnings.warn(
/projects/proteinml/.links/miniconda3/envs/aidep/lib/python3.10/site-
packages/sklearn/neural_network/_multilayer_perceptron.py:690:
ConvergenceWarning: Stochastic Optimizer: Maximum iterations (1000) reached and
the optimization hasn't converged yet.
  warnings.warn(
/projects/proteinml/.links/miniconda3/envs/aidep/lib/python3.10/site-
packages/sklearn/neural_network/_multilayer_perceptron.py:690:
ConvergenceWarning: Stochastic Optimizer: Maximum iterations (1000) reached and
the optimization hasn't converged yet.
  warnings.warn(
/projects/proteinml/.links/miniconda3/envs/aidep/lib/python3.10/site-
packages/sklearn/neural_network/_multilayer_perceptron.py:690:
ConvergenceWarning: Stochastic Optimizer: Maximum iterations (1000) reached and
the optimization hasn't converged yet.
  warnings.warn(
/projects/proteinml/.links/miniconda3/envs/aidep/lib/python3.10/site-
packages/sklearn/neural_network/_multilayer_perceptron.py:690:
ConvergenceWarning: Stochastic Optimizer: Maximum iterations (1000) reached and
the optimization hasn't converged yet.
  warnings.warn(
/projects/proteinml/.links/miniconda3/envs/aidep/lib/python3.10/site-
packages/sklearn/neural_network/_multilayer_perceptron.py:690:
ConvergenceWarning: Stochastic Optimizer: Maximum iterations (1000) reached and
the optimization hasn't converged yet.
  warnings.warn(
/projects/proteinml/.links/miniconda3/envs/aidep/lib/python3.10/site-
packages/sklearn/neural_network/_multilayer_perceptron.py:690:
ConvergenceWarning: Stochastic Optimizer: Maximum iterations (1000) reached and
the optimization hasn't converged yet.
  warnings.warn(
/projects/proteinml/.links/miniconda3/envs/aidep/lib/python3.10/site-
packages/sklearn/neural_network/_multilayer_perceptron.py:690:
ConvergenceWarning: Stochastic Optimizer: Maximum iterations (1000) reached and
the optimization hasn't converged yet.
  warnings.warn(
/projects/proteinml/.links/miniconda3/envs/aidep/lib/python3.10/site-
packages/sklearn/neural_network/_multilayer_perceptron.py:690:
ConvergenceWarning: Stochastic Optimizer: Maximum iterations (1000) reached and
the optimization hasn't converged yet.
  warnings.warn(
/projects/proteinml/.links/miniconda3/envs/aidep/lib/python3.10/site-
packages/sklearn/neural_network/_multilayer_perceptron.py:690:
ConvergenceWarning: Stochastic Optimizer: Maximum iterations (1000) reached and
```

```
the optimization hasn't converged yet.
  warnings.warn(
/projects/proteinml/.links/miniconda3/envs/aidep/lib/python3.10/site-
packages/sklearn/neural_network/_multilayer_perceptron.py:690:
ConvergenceWarning: Stochastic Optimizer: Maximum iterations (1000) reached and
the optimization hasn't converged yet.
  warnings.warn(
/projects/proteinml/.links/miniconda3/envs/aidep/lib/python3.10/site-
packages/sklearn/neural_network/_multilayer_perceptron.py:690:
ConvergenceWarning: Stochastic Optimizer: Maximum iterations (1000) reached and
the optimization hasn't converged yet.
  warnings.warn(
/projects/proteinml/.links/miniconda3/envs/aidep/lib/python3.10/site-
packages/sklearn/neural_network/_multilayer_perceptron.py:690:
ConvergenceWarning: Stochastic Optimizer: Maximum iterations (1000) reached and
the optimization hasn't converged yet.
  warnings.warn(
/projects/proteinml/.links/miniconda3/envs/aidep/lib/python3.10/site-
packages/sklearn/neural_network/_multilayer_perceptron.py:690:
ConvergenceWarning: Stochastic Optimizer: Maximum iterations (1000) reached and
the optimization hasn't converged yet.
  warnings.warn(
/projects/proteinml/.links/miniconda3/envs/aidep/lib/python3.10/site-
packages/sklearn/neural_network/_multilayer_perceptron.py:690:
ConvergenceWarning: Stochastic Optimizer: Maximum iterations (1000) reached and
the optimization hasn't converged yet.
  warnings.warn(
/projects/proteinml/.links/miniconda3/envs/aidep/lib/python3.10/site-
packages/sklearn/neural_network/_multilayer_perceptron.py:690:
ConvergenceWarning: Stochastic Optimizer: Maximum iterations (1000) reached and
the optimization hasn't converged yet.
  warnings.warn(
/projects/proteinml/.links/miniconda3/envs/aidep/lib/python3.10/site-
packages/sklearn/neural_network/_multilayer_perceptron.py:690:
ConvergenceWarning: Stochastic Optimizer: Maximum iterations (1000) reached and
the optimization hasn't converged yet.
  warnings.warn(
/projects/proteinml/.links/miniconda3/envs/aidep/lib/python3.10/site-
packages/sklearn/neural_network/_multilayer_perceptron.py:690:
ConvergenceWarning: Stochastic Optimizer: Maximum iterations (1000) reached and
the optimization hasn't converged yet.
  warnings.warn(
/projects/proteinml/.links/miniconda3/envs/aidep/lib/python3.10/site-
packages/sklearn/neural_network/_multilayer_perceptron.py:690:
ConvergenceWarning: Stochastic Optimizer: Maximum iterations (1000) reached and
the optimization hasn't converged yet.
  warnings.warn(
/projects/proteinml/.links/miniconda3/envs/aidep/lib/python3.10/site-
```

```
packages/sklearn/neural_network/_multilayer_perceptron.py:690:
ConvergenceWarning: Stochastic Optimizer: Maximum iterations (1000) reached and
the optimization hasn't converged yet.
  warnings.warn(
/projects/proteinml/.links/miniconda3/envs/aidep/lib/python3.10/site-
packages/sklearn/neural_network/_multilayer_perceptron.py:690:
ConvergenceWarning: Stochastic Optimizer: Maximum iterations (1000) reached and
the optimization hasn't converged yet.
  warnings.warn(
/projects/proteinml/.links/miniconda3/envs/aidep/lib/python3.10/site-
packages/sklearn/neural_network/_multilayer_perceptron.py:690:
ConvergenceWarning: Stochastic Optimizer: Maximum iterations (1000) reached and
the optimization hasn't converged yet.
  warnings.warn(
/projects/proteinml/.links/miniconda3/envs/aidep/lib/python3.10/site-
packages/sklearn/neural_network/_multilayer_perceptron.py:690:
ConvergenceWarning: Stochastic Optimizer: Maximum iterations (1000) reached and
the optimization hasn't converged yet.
  warnings.warn(
/projects/proteinml/.links/miniconda3/envs/aidep/lib/python3.10/site-
packages/sklearn/neural_network/_multilayer_perceptron.py:690:
ConvergenceWarning: Stochastic Optimizer: Maximum iterations (1000) reached and
the optimization hasn't converged yet.
  warnings.warn(
/projects/proteinml/.links/miniconda3/envs/aidep/lib/python3.10/site-
packages/sklearn/neural_network/_multilayer_perceptron.py:690:
ConvergenceWarning: Stochastic Optimizer: Maximum iterations (1000) reached and
the optimization hasn't converged yet.
  warnings.warn(
/projects/proteinml/.links/miniconda3/envs/aidep/lib/python3.10/site-
packages/sklearn/neural_network/_multilayer_perceptron.py:690:
ConvergenceWarning: Stochastic Optimizer: Maximum iterations (1000) reached and
the optimization hasn't converged yet.
  warnings.warn(
/projects/proteinml/.links/miniconda3/envs/aidep/lib/python3.10/site-
packages/sklearn/neural_network/_multilayer_perceptron.py:690:
ConvergenceWarning: Stochastic Optimizer: Maximum iterations (1000) reached and
the optimization hasn't converged yet.
  warnings.warn(
/projects/proteinml/.links/miniconda3/envs/aidep/lib/python3.10/site-
packages/sklearn/neural_network/_multilayer_perceptron.py:690:
ConvergenceWarning: Stochastic Optimizer: Maximum iterations (1000) reached and
the optimization hasn't converged yet.
  warnings.warn(
/projects/proteinml/.links/miniconda3/envs/aidep/lib/python3.10/site-
packages/sklearn/neural_network/_multilayer_perceptron.py:690:
ConvergenceWarning: Stochastic Optimizer: Maximum iterations (1000) reached and
the optimization hasn't converged yet.
```

```
  warnings.warn(
/projects/proteinml/.links/miniconda3/envs/aidep/lib/python3.10/site-
packages/sklearn/neural_network/_multilayer_perceptron.py:690:
ConvergenceWarning: Stochastic Optimizer: Maximum iterations (1000) reached and
the optimization hasn't converged yet.
  warnings.warn(
/projects/proteinml/.links/miniconda3/envs/aidep/lib/python3.10/site-
packages/sklearn/neural_network/_multilayer_perceptron.py:690:
ConvergenceWarning: Stochastic Optimizer: Maximum iterations (1000) reached and
the optimization hasn't converged yet.
  warnings.warn(
/projects/proteinml/.links/miniconda3/envs/aidep/lib/python3.10/site-
packages/sklearn/neural_network/_multilayer_perceptron.py:690:
ConvergenceWarning: Stochastic Optimizer: Maximum iterations (1000) reached and
the optimization hasn't converged yet.
  warnings.warn(
/projects/proteinml/.links/miniconda3/envs/aidep/lib/python3.10/site-
packages/sklearn/neural_network/_multilayer_perceptron.py:690:
ConvergenceWarning: Stochastic Optimizer: Maximum iterations (1000) reached and
the optimization hasn't converged yet.
  warnings.warn(
/projects/proteinml/.links/miniconda3/envs/aidep/lib/python3.10/site-
packages/sklearn/neural_network/_multilayer_perceptron.py:690:
ConvergenceWarning: Stochastic Optimizer: Maximum iterations (1000) reached and
the optimization hasn't converged yet.
  warnings.warn(
/projects/proteinml/.links/miniconda3/envs/aidep/lib/python3.10/site-
packages/sklearn/neural_network/_multilayer_perceptron.py:690:
ConvergenceWarning: Stochastic Optimizer: Maximum iterations (1000) reached and
the optimization hasn't converged yet.
  warnings.warn(
/projects/proteinml/.links/miniconda3/envs/aidep/lib/python3.10/site-
packages/sklearn/neural_network/_multilayer_perceptron.py:690:
ConvergenceWarning: Stochastic Optimizer: Maximum iterations (1000) reached and
the optimization hasn't converged yet.
  warnings.warn(
/projects/proteinml/.links/miniconda3/envs/aidep/lib/python3.10/site-
packages/sklearn/neural_network/_multilayer_perceptron.py:690:
ConvergenceWarning: Stochastic Optimizer: Maximum iterations (1000) reached and
the optimization hasn't converged yet.
  warnings.warn(
/projects/proteinml/.links/miniconda3/envs/aidep/lib/python3.10/site-
packages/sklearn/neural_network/_multilayer_perceptron.py:690:
ConvergenceWarning: Stochastic Optimizer: Maximum iterations (1000) reached and
the optimization hasn't converged yet.
  warnings.warn(
/projects/proteinml/.links/miniconda3/envs/aidep/lib/python3.10/site-
packages/sklearn/neural_network/_multilayer_perceptron.py:690:
```

```
ConvergenceWarning: Stochastic Optimizer: Maximum iterations (1000) reached and
the optimization hasn't converged yet.
  warnings.warn(
/projects/proteinml/.links/miniconda3/envs/aidep/lib/python3.10/site-
packages/sklearn/neural_network/_multilayer_perceptron.py:690:
ConvergenceWarning: Stochastic Optimizer: Maximum iterations (1000) reached and
the optimization hasn't converged yet.
  warnings.warn(
/projects/proteinml/.links/miniconda3/envs/aidep/lib/python3.10/site-
packages/sklearn/neural_network/_multilayer_perceptron.py:690:
ConvergenceWarning: Stochastic Optimizer: Maximum iterations (1000) reached and
the optimization hasn't converged yet.
  warnings.warn(
/projects/proteinml/.links/miniconda3/envs/aidep/lib/python3.10/site-
packages/sklearn/neural_network/_multilayer_perceptron.py:690:
ConvergenceWarning: Stochastic Optimizer: Maximum iterations (1000) reached and
the optimization hasn't converged yet.
  warnings.warn(
/projects/proteinml/.links/miniconda3/envs/aidep/lib/python3.10/site-
packages/sklearn/neural_network/_multilayer_perceptron.py:690:
ConvergenceWarning: Stochastic Optimizer: Maximum iterations (1000) reached and
the optimization hasn't converged yet.
  warnings.warn(
/projects/proteinml/.links/miniconda3/envs/aidep/lib/python3.10/site-
packages/sklearn/neural_network/_multilayer_perceptron.py:690:
ConvergenceWarning: Stochastic Optimizer: Maximum iterations (1000) reached and
the optimization hasn't converged yet.
  warnings.warn(
/projects/proteinml/.links/miniconda3/envs/aidep/lib/python3.10/site-
packages/sklearn/neural_network/_multilayer_perceptron.py:690:
ConvergenceWarning: Stochastic Optimizer: Maximum iterations (1000) reached and
the optimization hasn't converged yet.
  warnings.warn(
/projects/proteinml/.links/miniconda3/envs/aidep/lib/python3.10/site-
packages/sklearn/neural_network/_multilayer_perceptron.py:690:
ConvergenceWarning: Stochastic Optimizer: Maximum iterations (1000) reached and
the optimization hasn't converged yet.
  warnings.warn(
/projects/proteinml/.links/miniconda3/envs/aidep/lib/python3.10/site-
packages/sklearn/neural_network/_multilayer_perceptron.py:690:
ConvergenceWarning: Stochastic Optimizer: Maximum iterations (1000) reached and
the optimization hasn't converged yet.
  warnings.warn(
/projects/proteinml/.links/miniconda3/envs/aidep/lib/python3.10/site-
packages/sklearn/neural_network/_multilayer_perceptron.py:690:
ConvergenceWarning: Stochastic Optimizer: Maximum iterations (1000) reached and
the optimization hasn't converged yet.
  warnings.warn(
```

```
/projects/proteinml/.links/miniconda3/envs/aidep/lib/python3.10/site-
packages/sklearn/neural_network/_multilayer_perceptron.py:690:
ConvergenceWarning: Stochastic Optimizer: Maximum iterations (1000) reached and
the optimization hasn't converged yet.
  warnings.warn(
/projects/proteinml/.links/miniconda3/envs/aidep/lib/python3.10/site-
packages/sklearn/neural_network/_multilayer_perceptron.py:690:
ConvergenceWarning: Stochastic Optimizer: Maximum iterations (1000) reached and
the optimization hasn't converged yet.
  warnings.warn(
/projects/proteinml/.links/miniconda3/envs/aidep/lib/python3.10/site-
packages/sklearn/neural_network/_multilayer_perceptron.py:690:
ConvergenceWarning: Stochastic Optimizer: Maximum iterations (1000) reached and
the optimization hasn't converged yet.
  warnings.warn(
/projects/proteinml/.links/miniconda3/envs/aidep/lib/python3.10/site-
packages/sklearn/neural_network/_multilayer_perceptron.py:690:
ConvergenceWarning: Stochastic Optimizer: Maximum iterations (1000) reached and
the optimization hasn't converged yet.
  warnings.warn(
/projects/proteinml/.links/miniconda3/envs/aidep/lib/python3.10/site-
packages/sklearn/neural_network/_multilayer_perceptron.py:690:
ConvergenceWarning: Stochastic Optimizer: Maximum iterations (1000) reached and
the optimization hasn't converged yet.
  warnings.warn(
/projects/proteinml/.links/miniconda3/envs/aidep/lib/python3.10/site-
packages/sklearn/neural_network/_multilayer_perceptron.py:690:
ConvergenceWarning: Stochastic Optimizer: Maximum iterations (1000) reached and
the optimization hasn't converged yet.
  warnings.warn(
```

[54]:
```
['none_ohe_mlp_scores.joblib']
```

1.5.4 3.3 ESM2 embeddings, full sequence mean pool, linear model

Include scaling and PCA to reduce dims.

[22]:
```
esm2_embedder = ap.ESM2Embedding(
    pool='mean',
    metadata_folder=os.path.join('.', 'data', 'epistatic', 'esm2emb_full'),
    device='cuda:1',
    model_checkpoint='esm2_t12_35M_UR50D',
    use_cache=True,
    batch_size=180
)
esm2_embedder.fit()
X_esm = esm2_embedder.transform(X)
```

```
Some weights of EsmModel were not initialized from the model checkpoint at
facebook/esm2_t12_35M_UR50D and are newly initialized: ['pooler.dense.bias',
'pooler.dense.weight']
You should probably TRAIN this model on a down-stream task to be able to use it
for predictions and inference.
INFO:aide_predict.bespoke_models.base:Transforming input using ESM2Embedding
INFO:aide_predict.bespoke_models.base:Found 159129 cached results, running on 0
uncached sequences.
```

[20]:
```
none_esmfull_linear_scores = hyperopt_and_scoring(linear_pca, X_esm, y,␣
↪linear_param_space)
joblib.dump(none_esmfull_linear_scores, 'none_esmfull_linear_scores.joblib')
```

[20]:
```
['none_esmfull_linear_scores.joblib']
```

1.5.5 3.4 ESM2 embeddings, full sequence mean pooled, MLP

[21]:
```
none_esmfull_mlp_scores = hyperopt_and_scoring(mlp_pca, X_esm, y,␣
↪mlp_param_space)
joblib.dump(none_esmfull_mlp_scores, 'none_esmfull_mlp_scores.joblib')
```

```
/projects/proteinml/.links/miniconda3/envs/aidep/lib/python3.10/site-
packages/sklearn/neural_network/_multilayer_perceptron.py:690:
ConvergenceWarning: Stochastic Optimizer: Maximum iterations (1000) reached and
the optimization hasn't converged yet.
  warnings.warn(
/projects/proteinml/.links/miniconda3/envs/aidep/lib/python3.10/site-
packages/sklearn/neural_network/_multilayer_perceptron.py:690:
ConvergenceWarning: Stochastic Optimizer: Maximum iterations (1000) reached and
the optimization hasn't converged yet.
  warnings.warn(
/projects/proteinml/.links/miniconda3/envs/aidep/lib/python3.10/site-
packages/sklearn/neural_network/_multilayer_perceptron.py:690:
ConvergenceWarning: Stochastic Optimizer: Maximum iterations (1000) reached and
the optimization hasn't converged yet.
  warnings.warn(
/projects/proteinml/.links/miniconda3/envs/aidep/lib/python3.10/site-
packages/sklearn/neural_network/_multilayer_perceptron.py:690:
ConvergenceWarning: Stochastic Optimizer: Maximum iterations (1000) reached and
the optimization hasn't converged yet.
  warnings.warn(
/projects/proteinml/.links/miniconda3/envs/aidep/lib/python3.10/site-
packages/sklearn/neural_network/_multilayer_perceptron.py:690:
ConvergenceWarning: Stochastic Optimizer: Maximum iterations (1000) reached and
the optimization hasn't converged yet.
  warnings.warn(
/projects/proteinml/.links/miniconda3/envs/aidep/lib/python3.10/site-
```

```
packages/sklearn/neural_network/_multilayer_perceptron.py:690:
ConvergenceWarning: Stochastic Optimizer: Maximum iterations (1000) reached and
the optimization hasn't converged yet.
  warnings.warn(
/projects/proteinml/.links/miniconda3/envs/aidep/lib/python3.10/site-
packages/sklearn/neural_network/_multilayer_perceptron.py:690:
ConvergenceWarning: Stochastic Optimizer: Maximum iterations (1000) reached and
the optimization hasn't converged yet.
  warnings.warn(
/projects/proteinml/.links/miniconda3/envs/aidep/lib/python3.10/site-
packages/sklearn/neural_network/_multilayer_perceptron.py:690:
ConvergenceWarning: Stochastic Optimizer: Maximum iterations (1000) reached and
the optimization hasn't converged yet.
  warnings.warn(
/projects/proteinml/.links/miniconda3/envs/aidep/lib/python3.10/site-
packages/sklearn/neural_network/_multilayer_perceptron.py:690:
ConvergenceWarning: Stochastic Optimizer: Maximum iterations (1000) reached and
the optimization hasn't converged yet.
  warnings.warn(
/projects/proteinml/.links/miniconda3/envs/aidep/lib/python3.10/site-
packages/sklearn/neural_network/_multilayer_perceptron.py:690:
ConvergenceWarning: Stochastic Optimizer: Maximum iterations (1000) reached and
the optimization hasn't converged yet.
  warnings.warn(
/projects/proteinml/.links/miniconda3/envs/aidep/lib/python3.10/site-
packages/sklearn/neural_network/_multilayer_perceptron.py:690:
ConvergenceWarning: Stochastic Optimizer: Maximum iterations (1000) reached and
the optimization hasn't converged yet.
  warnings.warn(
/projects/proteinml/.links/miniconda3/envs/aidep/lib/python3.10/site-
packages/sklearn/neural_network/_multilayer_perceptron.py:690:
ConvergenceWarning: Stochastic Optimizer: Maximum iterations (1000) reached and
the optimization hasn't converged yet.
  warnings.warn(
/projects/proteinml/.links/miniconda3/envs/aidep/lib/python3.10/site-
packages/sklearn/neural_network/_multilayer_perceptron.py:690:
ConvergenceWarning: Stochastic Optimizer: Maximum iterations (1000) reached and
the optimization hasn't converged yet.
  warnings.warn(
/projects/proteinml/.links/miniconda3/envs/aidep/lib/python3.10/site-
packages/sklearn/neural_network/_multilayer_perceptron.py:690:
ConvergenceWarning: Stochastic Optimizer: Maximum iterations (1000) reached and
the optimization hasn't converged yet.
  warnings.warn(
/projects/proteinml/.links/miniconda3/envs/aidep/lib/python3.10/site-
packages/sklearn/neural_network/_multilayer_perceptron.py:690:
ConvergenceWarning: Stochastic Optimizer: Maximum iterations (1000) reached and
the optimization hasn't converged yet.
```

```
  warnings.warn(
/projects/proteinml/.links/miniconda3/envs/aidep/lib/python3.10/site-
packages/sklearn/neural_network/_multilayer_perceptron.py:690:
ConvergenceWarning: Stochastic Optimizer: Maximum iterations (1000) reached and
the optimization hasn't converged yet.
  warnings.warn(
/projects/proteinml/.links/miniconda3/envs/aidep/lib/python3.10/site-
packages/sklearn/neural_network/_multilayer_perceptron.py:690:
ConvergenceWarning: Stochastic Optimizer: Maximum iterations (1000) reached and
the optimization hasn't converged yet.
  warnings.warn(
/projects/proteinml/.links/miniconda3/envs/aidep/lib/python3.10/site-
packages/sklearn/neural_network/_multilayer_perceptron.py:690:
ConvergenceWarning: Stochastic Optimizer: Maximum iterations (1000) reached and
the optimization hasn't converged yet.
  warnings.warn(
/projects/proteinml/.links/miniconda3/envs/aidep/lib/python3.10/site-
packages/sklearn/neural_network/_multilayer_perceptron.py:690:
ConvergenceWarning: Stochastic Optimizer: Maximum iterations (1000) reached and
the optimization hasn't converged yet.
  warnings.warn(
/projects/proteinml/.links/miniconda3/envs/aidep/lib/python3.10/site-
packages/sklearn/neural_network/_multilayer_perceptron.py:690:
ConvergenceWarning: Stochastic Optimizer: Maximum iterations (1000) reached and
the optimization hasn't converged yet.
  warnings.warn(
/projects/proteinml/.links/miniconda3/envs/aidep/lib/python3.10/site-
packages/sklearn/neural_network/_multilayer_perceptron.py:690:
ConvergenceWarning: Stochastic Optimizer: Maximum iterations (1000) reached and
the optimization hasn't converged yet.
  warnings.warn(
/projects/proteinml/.links/miniconda3/envs/aidep/lib/python3.10/site-
packages/sklearn/neural_network/_multilayer_perceptron.py:690:
ConvergenceWarning: Stochastic Optimizer: Maximum iterations (1000) reached and
the optimization hasn't converged yet.
  warnings.warn(
/projects/proteinml/.links/miniconda3/envs/aidep/lib/python3.10/site-
packages/sklearn/neural_network/_multilayer_perceptron.py:690:
ConvergenceWarning: Stochastic Optimizer: Maximum iterations (1000) reached and
the optimization hasn't converged yet.
  warnings.warn(
/projects/proteinml/.links/miniconda3/envs/aidep/lib/python3.10/site-
packages/sklearn/neural_network/_multilayer_perceptron.py:690:
ConvergenceWarning: Stochastic Optimizer: Maximum iterations (1000) reached and
the optimization hasn't converged yet.
  warnings.warn(
/projects/proteinml/.links/miniconda3/envs/aidep/lib/python3.10/site-
packages/sklearn/neural_network/_multilayer_perceptron.py:690:
```

```
ConvergenceWarning: Stochastic Optimizer: Maximum iterations (1000) reached and
the optimization hasn't converged yet.
  warnings.warn(
/projects/proteinml/.links/miniconda3/envs/aidep/lib/python3.10/site-
packages/sklearn/neural_network/_multilayer_perceptron.py:690:
ConvergenceWarning: Stochastic Optimizer: Maximum iterations (1000) reached and
the optimization hasn't converged yet.
  warnings.warn(
/projects/proteinml/.links/miniconda3/envs/aidep/lib/python3.10/site-
packages/sklearn/neural_network/_multilayer_perceptron.py:690:
ConvergenceWarning: Stochastic Optimizer: Maximum iterations (1000) reached and
the optimization hasn't converged yet.
  warnings.warn(
/projects/proteinml/.links/miniconda3/envs/aidep/lib/python3.10/site-
packages/sklearn/neural_network/_multilayer_perceptron.py:690:
ConvergenceWarning: Stochastic Optimizer: Maximum iterations (1000) reached and
the optimization hasn't converged yet.
  warnings.warn(
/projects/proteinml/.links/miniconda3/envs/aidep/lib/python3.10/site-
packages/sklearn/neural_network/_multilayer_perceptron.py:690:
ConvergenceWarning: Stochastic Optimizer: Maximum iterations (1000) reached and
the optimization hasn't converged yet.
  warnings.warn(
/projects/proteinml/.links/miniconda3/envs/aidep/lib/python3.10/site-
packages/sklearn/neural_network/_multilayer_perceptron.py:690:
ConvergenceWarning: Stochastic Optimizer: Maximum iterations (1000) reached and
the optimization hasn't converged yet.
  warnings.warn(
/projects/proteinml/.links/miniconda3/envs/aidep/lib/python3.10/site-
packages/sklearn/neural_network/_multilayer_perceptron.py:690:
ConvergenceWarning: Stochastic Optimizer: Maximum iterations (1000) reached and
the optimization hasn't converged yet.
  warnings.warn(
/projects/proteinml/.links/miniconda3/envs/aidep/lib/python3.10/site-
packages/sklearn/neural_network/_multilayer_perceptron.py:690:
ConvergenceWarning: Stochastic Optimizer: Maximum iterations (1000) reached and
the optimization hasn't converged yet.
  warnings.warn(
/projects/proteinml/.links/miniconda3/envs/aidep/lib/python3.10/site-
packages/sklearn/neural_network/_multilayer_perceptron.py:690:
ConvergenceWarning: Stochastic Optimizer: Maximum iterations (1000) reached and
the optimization hasn't converged yet.
  warnings.warn(
/projects/proteinml/.links/miniconda3/envs/aidep/lib/python3.10/site-
packages/sklearn/neural_network/_multilayer_perceptron.py:690:
ConvergenceWarning: Stochastic Optimizer: Maximum iterations (1000) reached and
the optimization hasn't converged yet.
  warnings.warn(
```

```
/projects/proteinml/.links/miniconda3/envs/aidep/lib/python3.10/site-
packages/sklearn/neural_network/_multilayer_perceptron.py:690:
ConvergenceWarning: Stochastic Optimizer: Maximum iterations (1000) reached and
the optimization hasn't converged yet.
  warnings.warn(
/projects/proteinml/.links/miniconda3/envs/aidep/lib/python3.10/site-
packages/sklearn/neural_network/_multilayer_perceptron.py:690:
ConvergenceWarning: Stochastic Optimizer: Maximum iterations (1000) reached and
the optimization hasn't converged yet.
  warnings.warn(
/projects/proteinml/.links/miniconda3/envs/aidep/lib/python3.10/site-
packages/sklearn/neural_network/_multilayer_perceptron.py:690:
ConvergenceWarning: Stochastic Optimizer: Maximum iterations (1000) reached and
the optimization hasn't converged yet.
  warnings.warn(
/projects/proteinml/.links/miniconda3/envs/aidep/lib/python3.10/site-
packages/sklearn/neural_network/_multilayer_perceptron.py:690:
ConvergenceWarning: Stochastic Optimizer: Maximum iterations (1000) reached and
the optimization hasn't converged yet.
  warnings.warn(
/projects/proteinml/.links/miniconda3/envs/aidep/lib/python3.10/site-
packages/sklearn/neural_network/_multilayer_perceptron.py:690:
ConvergenceWarning: Stochastic Optimizer: Maximum iterations (1000) reached and
the optimization hasn't converged yet.
  warnings.warn(
/projects/proteinml/.links/miniconda3/envs/aidep/lib/python3.10/site-
packages/sklearn/neural_network/_multilayer_perceptron.py:690:
ConvergenceWarning: Stochastic Optimizer: Maximum iterations (1000) reached and
the optimization hasn't converged yet.
  warnings.warn(
/projects/proteinml/.links/miniconda3/envs/aidep/lib/python3.10/site-
packages/sklearn/neural_network/_multilayer_perceptron.py:690:
ConvergenceWarning: Stochastic Optimizer: Maximum iterations (1000) reached and
the optimization hasn't converged yet.
  warnings.warn(
/projects/proteinml/.links/miniconda3/envs/aidep/lib/python3.10/site-
packages/sklearn/neural_network/_multilayer_perceptron.py:690:
ConvergenceWarning: Stochastic Optimizer: Maximum iterations (1000) reached and
the optimization hasn't converged yet.
  warnings.warn(
/projects/proteinml/.links/miniconda3/envs/aidep/lib/python3.10/site-
packages/sklearn/neural_network/_multilayer_perceptron.py:690:
ConvergenceWarning: Stochastic Optimizer: Maximum iterations (1000) reached and
the optimization hasn't converged yet.
  warnings.warn(
/projects/proteinml/.links/miniconda3/envs/aidep/lib/python3.10/site-
packages/sklearn/neural_network/_multilayer_perceptron.py:690:
ConvergenceWarning: Stochastic Optimizer: Maximum iterations (1000) reached and
```

```
the optimization hasn't converged yet.
  warnings.warn(
/projects/proteinml/.links/miniconda3/envs/aidep/lib/python3.10/site-
packages/sklearn/neural_network/_multilayer_perceptron.py:690:
ConvergenceWarning: Stochastic Optimizer: Maximum iterations (1000) reached and
the optimization hasn't converged yet.
  warnings.warn(
/projects/proteinml/.links/miniconda3/envs/aidep/lib/python3.10/site-
packages/sklearn/neural_network/_multilayer_perceptron.py:690:
ConvergenceWarning: Stochastic Optimizer: Maximum iterations (1000) reached and
the optimization hasn't converged yet.
  warnings.warn(
/projects/proteinml/.links/miniconda3/envs/aidep/lib/python3.10/site-
packages/sklearn/neural_network/_multilayer_perceptron.py:690:
ConvergenceWarning: Stochastic Optimizer: Maximum iterations (1000) reached and
the optimization hasn't converged yet.
  warnings.warn(
/projects/proteinml/.links/miniconda3/envs/aidep/lib/python3.10/site-
packages/sklearn/neural_network/_multilayer_perceptron.py:690:
ConvergenceWarning: Stochastic Optimizer: Maximum iterations (1000) reached and
the optimization hasn't converged yet.
  warnings.warn(
/projects/proteinml/.links/miniconda3/envs/aidep/lib/python3.10/site-
packages/sklearn/neural_network/_multilayer_perceptron.py:690:
ConvergenceWarning: Stochastic Optimizer: Maximum iterations (1000) reached and
the optimization hasn't converged yet.
  warnings.warn(
/projects/proteinml/.links/miniconda3/envs/aidep/lib/python3.10/site-
packages/sklearn/neural_network/_multilayer_perceptron.py:690:
ConvergenceWarning: Stochastic Optimizer: Maximum iterations (1000) reached and
the optimization hasn't converged yet.
  warnings.warn(
/projects/proteinml/.links/miniconda3/envs/aidep/lib/python3.10/site-
packages/sklearn/neural_network/_multilayer_perceptron.py:690:
ConvergenceWarning: Stochastic Optimizer: Maximum iterations (1000) reached and
the optimization hasn't converged yet.
  warnings.warn(
/projects/proteinml/.links/miniconda3/envs/aidep/lib/python3.10/site-
packages/sklearn/neural_network/_multilayer_perceptron.py:690:
ConvergenceWarning: Stochastic Optimizer: Maximum iterations (1000) reached and
the optimization hasn't converged yet.
  warnings.warn(
/projects/proteinml/.links/miniconda3/envs/aidep/lib/python3.10/site-
packages/sklearn/neural_network/_multilayer_perceptron.py:690:
ConvergenceWarning: Stochastic Optimizer: Maximum iterations (1000) reached and
the optimization hasn't converged yet.
  warnings.warn(
/projects/proteinml/.links/miniconda3/envs/aidep/lib/python3.10/site-
```

```
packages/sklearn/neural_network/_multilayer_perceptron.py:690:
ConvergenceWarning: Stochastic Optimizer: Maximum iterations (1000) reached and
the optimization hasn't converged yet.
  warnings.warn(
/projects/proteinml/.links/miniconda3/envs/aidep/lib/python3.10/site-
packages/sklearn/neural_network/_multilayer_perceptron.py:690:
ConvergenceWarning: Stochastic Optimizer: Maximum iterations (1000) reached and
the optimization hasn't converged yet.
  warnings.warn(
/projects/proteinml/.links/miniconda3/envs/aidep/lib/python3.10/site-
packages/sklearn/neural_network/_multilayer_perceptron.py:690:
ConvergenceWarning: Stochastic Optimizer: Maximum iterations (1000) reached and
the optimization hasn't converged yet.
  warnings.warn(
/projects/proteinml/.links/miniconda3/envs/aidep/lib/python3.10/site-
packages/sklearn/neural_network/_multilayer_perceptron.py:690:
ConvergenceWarning: Stochastic Optimizer: Maximum iterations (1000) reached and
the optimization hasn't converged yet.
  warnings.warn(
/projects/proteinml/.links/miniconda3/envs/aidep/lib/python3.10/site-
packages/sklearn/neural_network/_multilayer_perceptron.py:690:
ConvergenceWarning: Stochastic Optimizer: Maximum iterations (1000) reached and
the optimization hasn't converged yet.
  warnings.warn(
/projects/proteinml/.links/miniconda3/envs/aidep/lib/python3.10/site-
packages/sklearn/neural_network/_multilayer_perceptron.py:690:
ConvergenceWarning: Stochastic Optimizer: Maximum iterations (1000) reached and
the optimization hasn't converged yet.
  warnings.warn(
/projects/proteinml/.links/miniconda3/envs/aidep/lib/python3.10/site-
packages/sklearn/neural_network/_multilayer_perceptron.py:690:
ConvergenceWarning: Stochastic Optimizer: Maximum iterations (1000) reached and
the optimization hasn't converged yet.
  warnings.warn(
/projects/proteinml/.links/miniconda3/envs/aidep/lib/python3.10/site-
packages/sklearn/neural_network/_multilayer_perceptron.py:690:
ConvergenceWarning: Stochastic Optimizer: Maximum iterations (1000) reached and
the optimization hasn't converged yet.
  warnings.warn(
/projects/proteinml/.links/miniconda3/envs/aidep/lib/python3.10/site-
packages/sklearn/neural_network/_multilayer_perceptron.py:690:
ConvergenceWarning: Stochastic Optimizer: Maximum iterations (1000) reached and
the optimization hasn't converged yet.
  warnings.warn(
/projects/proteinml/.links/miniconda3/envs/aidep/lib/python3.10/site-
packages/sklearn/neural_network/_multilayer_perceptron.py:690:
ConvergenceWarning: Stochastic Optimizer: Maximum iterations (1000) reached and
the optimization hasn't converged yet.
```

```
  warnings.warn(
/projects/proteinml/.links/miniconda3/envs/aidep/lib/python3.10/site-
packages/sklearn/neural_network/_multilayer_perceptron.py:690:
ConvergenceWarning: Stochastic Optimizer: Maximum iterations (1000) reached and
the optimization hasn't converged yet.
  warnings.warn(
/projects/proteinml/.links/miniconda3/envs/aidep/lib/python3.10/site-
packages/sklearn/neural_network/_multilayer_perceptron.py:690:
ConvergenceWarning: Stochastic Optimizer: Maximum iterations (1000) reached and
the optimization hasn't converged yet.
  warnings.warn(
/projects/proteinml/.links/miniconda3/envs/aidep/lib/python3.10/site-
packages/sklearn/neural_network/_multilayer_perceptron.py:690:
ConvergenceWarning: Stochastic Optimizer: Maximum iterations (1000) reached and
the optimization hasn't converged yet.
  warnings.warn(
/projects/proteinml/.links/miniconda3/envs/aidep/lib/python3.10/site-
packages/sklearn/neural_network/_multilayer_perceptron.py:690:
ConvergenceWarning: Stochastic Optimizer: Maximum iterations (1000) reached and
the optimization hasn't converged yet.
  warnings.warn(
/projects/proteinml/.links/miniconda3/envs/aidep/lib/python3.10/site-
packages/sklearn/neural_network/_multilayer_perceptron.py:690:
ConvergenceWarning: Stochastic Optimizer: Maximum iterations (1000) reached and
the optimization hasn't converged yet.
  warnings.warn(
/projects/proteinml/.links/miniconda3/envs/aidep/lib/python3.10/site-
packages/sklearn/neural_network/_multilayer_perceptron.py:690:
ConvergenceWarning: Stochastic Optimizer: Maximum iterations (1000) reached and
the optimization hasn't converged yet.
  warnings.warn(
/projects/proteinml/.links/miniconda3/envs/aidep/lib/python3.10/site-
packages/sklearn/neural_network/_multilayer_perceptron.py:690:
ConvergenceWarning: Stochastic Optimizer: Maximum iterations (1000) reached and
the optimization hasn't converged yet.
  warnings.warn(
/projects/proteinml/.links/miniconda3/envs/aidep/lib/python3.10/site-
packages/sklearn/neural_network/_multilayer_perceptron.py:690:
ConvergenceWarning: Stochastic Optimizer: Maximum iterations (1000) reached and
the optimization hasn't converged yet.
  warnings.warn(
/projects/proteinml/.links/miniconda3/envs/aidep/lib/python3.10/site-
packages/sklearn/neural_network/_multilayer_perceptron.py:690:
ConvergenceWarning: Stochastic Optimizer: Maximum iterations (1000) reached and
the optimization hasn't converged yet.
  warnings.warn(
/projects/proteinml/.links/miniconda3/envs/aidep/lib/python3.10/site-
packages/sklearn/neural_network/_multilayer_perceptron.py:690:
```

```
ConvergenceWarning: Stochastic Optimizer: Maximum iterations (1000) reached and
the optimization hasn't converged yet.
  warnings.warn(
/projects/proteinml/.links/miniconda3/envs/aidep/lib/python3.10/site-
packages/sklearn/neural_network/_multilayer_perceptron.py:690:
ConvergenceWarning: Stochastic Optimizer: Maximum iterations (1000) reached and
the optimization hasn't converged yet.
  warnings.warn(
/projects/proteinml/.links/miniconda3/envs/aidep/lib/python3.10/site-
packages/sklearn/neural_network/_multilayer_perceptron.py:690:
ConvergenceWarning: Stochastic Optimizer: Maximum iterations (1000) reached and
the optimization hasn't converged yet.
  warnings.warn(
/projects/proteinml/.links/miniconda3/envs/aidep/lib/python3.10/site-
packages/sklearn/neural_network/_multilayer_perceptron.py:690:
ConvergenceWarning: Stochastic Optimizer: Maximum iterations (1000) reached and
the optimization hasn't converged yet.
  warnings.warn(
/projects/proteinml/.links/miniconda3/envs/aidep/lib/python3.10/site-
packages/sklearn/neural_network/_multilayer_perceptron.py:690:
ConvergenceWarning: Stochastic Optimizer: Maximum iterations (1000) reached and
the optimization hasn't converged yet.
  warnings.warn(
/projects/proteinml/.links/miniconda3/envs/aidep/lib/python3.10/site-
packages/sklearn/neural_network/_multilayer_perceptron.py:690:
ConvergenceWarning: Stochastic Optimizer: Maximum iterations (1000) reached and
the optimization hasn't converged yet.
  warnings.warn(
/projects/proteinml/.links/miniconda3/envs/aidep/lib/python3.10/site-
packages/sklearn/neural_network/_multilayer_perceptron.py:690:
ConvergenceWarning: Stochastic Optimizer: Maximum iterations (1000) reached and
the optimization hasn't converged yet.
  warnings.warn(
/projects/proteinml/.links/miniconda3/envs/aidep/lib/python3.10/site-
packages/sklearn/neural_network/_multilayer_perceptron.py:690:
ConvergenceWarning: Stochastic Optimizer: Maximum iterations (1000) reached and
the optimization hasn't converged yet.
  warnings.warn(
/projects/proteinml/.links/miniconda3/envs/aidep/lib/python3.10/site-
packages/sklearn/neural_network/_multilayer_perceptron.py:690:
ConvergenceWarning: Stochastic Optimizer: Maximum iterations (1000) reached and
the optimization hasn't converged yet.
  warnings.warn(
/projects/proteinml/.links/miniconda3/envs/aidep/lib/python3.10/site-
packages/sklearn/neural_network/_multilayer_perceptron.py:690:
ConvergenceWarning: Stochastic Optimizer: Maximum iterations (1000) reached and
the optimization hasn't converged yet.
  warnings.warn(
```

```
/projects/proteinml/.links/miniconda3/envs/aidep/lib/python3.10/site-
packages/sklearn/neural_network/_multilayer_perceptron.py:690:
ConvergenceWarning: Stochastic Optimizer: Maximum iterations (1000) reached and
the optimization hasn't converged yet.
  warnings.warn(
/projects/proteinml/.links/miniconda3/envs/aidep/lib/python3.10/site-
packages/sklearn/neural_network/_multilayer_perceptron.py:690:
ConvergenceWarning: Stochastic Optimizer: Maximum iterations (1000) reached and
the optimization hasn't converged yet.
  warnings.warn(
/projects/proteinml/.links/miniconda3/envs/aidep/lib/python3.10/site-
packages/sklearn/neural_network/_multilayer_perceptron.py:690:
ConvergenceWarning: Stochastic Optimizer: Maximum iterations (1000) reached and
the optimization hasn't converged yet.
  warnings.warn(
/projects/proteinml/.links/miniconda3/envs/aidep/lib/python3.10/site-
packages/sklearn/neural_network/_multilayer_perceptron.py:690:
ConvergenceWarning: Stochastic Optimizer: Maximum iterations (1000) reached and
the optimization hasn't converged yet.
  warnings.warn(
/projects/proteinml/.links/miniconda3/envs/aidep/lib/python3.10/site-
packages/sklearn/neural_network/_multilayer_perceptron.py:690:
ConvergenceWarning: Stochastic Optimizer: Maximum iterations (1000) reached and
the optimization hasn't converged yet.
  warnings.warn(
/projects/proteinml/.links/miniconda3/envs/aidep/lib/python3.10/site-
packages/sklearn/neural_network/_multilayer_perceptron.py:690:
ConvergenceWarning: Stochastic Optimizer: Maximum iterations (1000) reached and
the optimization hasn't converged yet.
  warnings.warn(
/projects/proteinml/.links/miniconda3/envs/aidep/lib/python3.10/site-
packages/sklearn/neural_network/_multilayer_perceptron.py:690:
ConvergenceWarning: Stochastic Optimizer: Maximum iterations (1000) reached and
the optimization hasn't converged yet.
  warnings.warn(
/projects/proteinml/.links/miniconda3/envs/aidep/lib/python3.10/site-
packages/sklearn/neural_network/_multilayer_perceptron.py:690:
ConvergenceWarning: Stochastic Optimizer: Maximum iterations (1000) reached and
the optimization hasn't converged yet.
  warnings.warn(
/projects/proteinml/.links/miniconda3/envs/aidep/lib/python3.10/site-
packages/sklearn/neural_network/_multilayer_perceptron.py:690:
ConvergenceWarning: Stochastic Optimizer: Maximum iterations (1000) reached and
the optimization hasn't converged yet.
  warnings.warn(
/projects/proteinml/.links/miniconda3/envs/aidep/lib/python3.10/site-
packages/sklearn/neural_network/_multilayer_perceptron.py:690:
ConvergenceWarning: Stochastic Optimizer: Maximum iterations (1000) reached and
```

```
the optimization hasn't converged yet.
  warnings.warn(
/projects/proteinml/.links/miniconda3/envs/aidep/lib/python3.10/site-
packages/sklearn/neural_network/_multilayer_perceptron.py:690:
ConvergenceWarning: Stochastic Optimizer: Maximum iterations (1000) reached and
the optimization hasn't converged yet.
  warnings.warn(
/projects/proteinml/.links/miniconda3/envs/aidep/lib/python3.10/site-
packages/sklearn/neural_network/_multilayer_perceptron.py:690:
ConvergenceWarning: Stochastic Optimizer: Maximum iterations (1000) reached and
the optimization hasn't converged yet.
  warnings.warn(
/projects/proteinml/.links/miniconda3/envs/aidep/lib/python3.10/site-
packages/sklearn/neural_network/_multilayer_perceptron.py:690:
ConvergenceWarning: Stochastic Optimizer: Maximum iterations (1000) reached and
the optimization hasn't converged yet.
  warnings.warn(
/projects/proteinml/.links/miniconda3/envs/aidep/lib/python3.10/site-
packages/sklearn/neural_network/_multilayer_perceptron.py:690:
ConvergenceWarning: Stochastic Optimizer: Maximum iterations (1000) reached and
the optimization hasn't converged yet.
  warnings.warn(
/projects/proteinml/.links/miniconda3/envs/aidep/lib/python3.10/site-
packages/sklearn/neural_network/_multilayer_perceptron.py:690:
ConvergenceWarning: Stochastic Optimizer: Maximum iterations (1000) reached and
the optimization hasn't converged yet.
  warnings.warn(
/projects/proteinml/.links/miniconda3/envs/aidep/lib/python3.10/site-
packages/sklearn/neural_network/_multilayer_perceptron.py:690:
ConvergenceWarning: Stochastic Optimizer: Maximum iterations (1000) reached and
the optimization hasn't converged yet.
  warnings.warn(
/projects/proteinml/.links/miniconda3/envs/aidep/lib/python3.10/site-
packages/sklearn/neural_network/_multilayer_perceptron.py:690:
ConvergenceWarning: Stochastic Optimizer: Maximum iterations (1000) reached and
the optimization hasn't converged yet.
  warnings.warn(
/projects/proteinml/.links/miniconda3/envs/aidep/lib/python3.10/site-
packages/sklearn/neural_network/_multilayer_perceptron.py:690:
ConvergenceWarning: Stochastic Optimizer: Maximum iterations (1000) reached and
the optimization hasn't converged yet.
  warnings.warn(
/projects/proteinml/.links/miniconda3/envs/aidep/lib/python3.10/site-
packages/sklearn/neural_network/_multilayer_perceptron.py:690:
ConvergenceWarning: Stochastic Optimizer: Maximum iterations (1000) reached and
the optimization hasn't converged yet.
  warnings.warn(
/projects/proteinml/.links/miniconda3/envs/aidep/lib/python3.10/site-
```

```
packages/sklearn/neural_network/_multilayer_perceptron.py:690:
ConvergenceWarning: Stochastic Optimizer: Maximum iterations (1000) reached and
the optimization hasn't converged yet.
  warnings.warn(
/projects/proteinml/.links/miniconda3/envs/aidep/lib/python3.10/site-
packages/sklearn/neural_network/_multilayer_perceptron.py:690:
ConvergenceWarning: Stochastic Optimizer: Maximum iterations (1000) reached and
the optimization hasn't converged yet.
  warnings.warn(
/projects/proteinml/.links/miniconda3/envs/aidep/lib/python3.10/site-
packages/sklearn/neural_network/_multilayer_perceptron.py:690:
ConvergenceWarning: Stochastic Optimizer: Maximum iterations (1000) reached and
the optimization hasn't converged yet.
  warnings.warn(
/projects/proteinml/.links/miniconda3/envs/aidep/lib/python3.10/site-
packages/sklearn/neural_network/_multilayer_perceptron.py:690:
ConvergenceWarning: Stochastic Optimizer: Maximum iterations (1000) reached and
the optimization hasn't converged yet.
  warnings.warn(
/projects/proteinml/.links/miniconda3/envs/aidep/lib/python3.10/site-
packages/sklearn/neural_network/_multilayer_perceptron.py:690:
ConvergenceWarning: Stochastic Optimizer: Maximum iterations (1000) reached and
the optimization hasn't converged yet.
  warnings.warn(
/projects/proteinml/.links/miniconda3/envs/aidep/lib/python3.10/site-
packages/sklearn/neural_network/_multilayer_perceptron.py:690:
ConvergenceWarning: Stochastic Optimizer: Maximum iterations (1000) reached and
the optimization hasn't converged yet.
  warnings.warn(
/projects/proteinml/.links/miniconda3/envs/aidep/lib/python3.10/site-
packages/sklearn/neural_network/_multilayer_perceptron.py:690:
ConvergenceWarning: Stochastic Optimizer: Maximum iterations (1000) reached and
the optimization hasn't converged yet.
  warnings.warn(
/projects/proteinml/.links/miniconda3/envs/aidep/lib/python3.10/site-
packages/sklearn/neural_network/_multilayer_perceptron.py:690:
ConvergenceWarning: Stochastic Optimizer: Maximum iterations (1000) reached and
the optimization hasn't converged yet.
  warnings.warn(
/projects/proteinml/.links/miniconda3/envs/aidep/lib/python3.10/site-
packages/sklearn/neural_network/_multilayer_perceptron.py:690:
ConvergenceWarning: Stochastic Optimizer: Maximum iterations (1000) reached and
the optimization hasn't converged yet.
  warnings.warn(
/projects/proteinml/.links/miniconda3/envs/aidep/lib/python3.10/site-
packages/sklearn/neural_network/_multilayer_perceptron.py:690:
ConvergenceWarning: Stochastic Optimizer: Maximum iterations (1000) reached and
the optimization hasn't converged yet.
```

```
  warnings.warn(
/projects/proteinml/.links/miniconda3/envs/aidep/lib/python3.10/site-
packages/sklearn/neural_network/_multilayer_perceptron.py:690:
ConvergenceWarning: Stochastic Optimizer: Maximum iterations (1000) reached and
the optimization hasn't converged yet.
  warnings.warn(
/projects/proteinml/.links/miniconda3/envs/aidep/lib/python3.10/site-
packages/sklearn/neural_network/_multilayer_perceptron.py:690:
ConvergenceWarning: Stochastic Optimizer: Maximum iterations (1000) reached and
the optimization hasn't converged yet.
  warnings.warn(
/projects/proteinml/.links/miniconda3/envs/aidep/lib/python3.10/site-
packages/sklearn/neural_network/_multilayer_perceptron.py:690:
ConvergenceWarning: Stochastic Optimizer: Maximum iterations (1000) reached and
the optimization hasn't converged yet.
  warnings.warn(
/projects/proteinml/.links/miniconda3/envs/aidep/lib/python3.10/site-
packages/sklearn/neural_network/_multilayer_perceptron.py:690:
ConvergenceWarning: Stochastic Optimizer: Maximum iterations (1000) reached and
the optimization hasn't converged yet.
  warnings.warn(
/projects/proteinml/.links/miniconda3/envs/aidep/lib/python3.10/site-
packages/sklearn/neural_network/_multilayer_perceptron.py:690:
ConvergenceWarning: Stochastic Optimizer: Maximum iterations (1000) reached and
the optimization hasn't converged yet.
  warnings.warn(
/projects/proteinml/.links/miniconda3/envs/aidep/lib/python3.10/site-
packages/sklearn/neural_network/_multilayer_perceptron.py:690:
ConvergenceWarning: Stochastic Optimizer: Maximum iterations (1000) reached and
the optimization hasn't converged yet.
  warnings.warn(
/projects/proteinml/.links/miniconda3/envs/aidep/lib/python3.10/site-
packages/sklearn/neural_network/_multilayer_perceptron.py:690:
ConvergenceWarning: Stochastic Optimizer: Maximum iterations (1000) reached and
the optimization hasn't converged yet.
  warnings.warn(
/projects/proteinml/.links/miniconda3/envs/aidep/lib/python3.10/site-
packages/sklearn/neural_network/_multilayer_perceptron.py:690:
ConvergenceWarning: Stochastic Optimizer: Maximum iterations (1000) reached and
the optimization hasn't converged yet.
  warnings.warn(
/projects/proteinml/.links/miniconda3/envs/aidep/lib/python3.10/site-
packages/sklearn/neural_network/_multilayer_perceptron.py:690:
ConvergenceWarning: Stochastic Optimizer: Maximum iterations (1000) reached and
the optimization hasn't converged yet.
  warnings.warn(
/projects/proteinml/.links/miniconda3/envs/aidep/lib/python3.10/site-
packages/sklearn/neural_network/_multilayer_perceptron.py:690:
```

```
ConvergenceWarning: Stochastic Optimizer: Maximum iterations (1000) reached and
the optimization hasn't converged yet.
  warnings.warn(
/projects/proteinml/.links/miniconda3/envs/aidep/lib/python3.10/site-
packages/sklearn/neural_network/_multilayer_perceptron.py:690:
ConvergenceWarning: Stochastic Optimizer: Maximum iterations (1000) reached and
the optimization hasn't converged yet.
  warnings.warn(
/projects/proteinml/.links/miniconda3/envs/aidep/lib/python3.10/site-
packages/sklearn/neural_network/_multilayer_perceptron.py:690:
ConvergenceWarning: Stochastic Optimizer: Maximum iterations (1000) reached and
the optimization hasn't converged yet.
  warnings.warn(
/projects/proteinml/.links/miniconda3/envs/aidep/lib/python3.10/site-
packages/sklearn/neural_network/_multilayer_perceptron.py:690:
ConvergenceWarning: Stochastic Optimizer: Maximum iterations (1000) reached and
the optimization hasn't converged yet.
  warnings.warn(
/projects/proteinml/.links/miniconda3/envs/aidep/lib/python3.10/site-
packages/sklearn/neural_network/_multilayer_perceptron.py:690:
ConvergenceWarning: Stochastic Optimizer: Maximum iterations (1000) reached and
the optimization hasn't converged yet.
  warnings.warn(
/projects/proteinml/.links/miniconda3/envs/aidep/lib/python3.10/site-
packages/sklearn/neural_network/_multilayer_perceptron.py:690:
ConvergenceWarning: Stochastic Optimizer: Maximum iterations (1000) reached and
the optimization hasn't converged yet.
  warnings.warn(
/projects/proteinml/.links/miniconda3/envs/aidep/lib/python3.10/site-
packages/sklearn/neural_network/_multilayer_perceptron.py:690:
ConvergenceWarning: Stochastic Optimizer: Maximum iterations (1000) reached and
the optimization hasn't converged yet.
  warnings.warn(
/projects/proteinml/.links/miniconda3/envs/aidep/lib/python3.10/site-
packages/sklearn/neural_network/_multilayer_perceptron.py:690:
ConvergenceWarning: Stochastic Optimizer: Maximum iterations (1000) reached and
the optimization hasn't converged yet.
  warnings.warn(
/projects/proteinml/.links/miniconda3/envs/aidep/lib/python3.10/site-
packages/sklearn/neural_network/_multilayer_perceptron.py:690:
ConvergenceWarning: Stochastic Optimizer: Maximum iterations (1000) reached and
the optimization hasn't converged yet.
  warnings.warn(
/projects/proteinml/.links/miniconda3/envs/aidep/lib/python3.10/site-
packages/sklearn/neural_network/_multilayer_perceptron.py:690:
ConvergenceWarning: Stochastic Optimizer: Maximum iterations (1000) reached and
the optimization hasn't converged yet.
  warnings.warn(
```

```
/projects/proteinml/.links/miniconda3/envs/aidep/lib/python3.10/site-
packages/sklearn/neural_network/_multilayer_perceptron.py:690:
ConvergenceWarning: Stochastic Optimizer: Maximum iterations (1000) reached and
the optimization hasn't converged yet.
  warnings.warn(
/projects/proteinml/.links/miniconda3/envs/aidep/lib/python3.10/site-
packages/sklearn/neural_network/_multilayer_perceptron.py:690:
ConvergenceWarning: Stochastic Optimizer: Maximum iterations (1000) reached and
the optimization hasn't converged yet.
  warnings.warn(
/projects/proteinml/.links/miniconda3/envs/aidep/lib/python3.10/site-
packages/sklearn/neural_network/_multilayer_perceptron.py:690:
ConvergenceWarning: Stochastic Optimizer: Maximum iterations (1000) reached and
the optimization hasn't converged yet.
  warnings.warn(
/projects/proteinml/.links/miniconda3/envs/aidep/lib/python3.10/site-
packages/sklearn/neural_network/_multilayer_perceptron.py:690:
ConvergenceWarning: Stochastic Optimizer: Maximum iterations (1000) reached and
the optimization hasn't converged yet.
  warnings.warn(
/projects/proteinml/.links/miniconda3/envs/aidep/lib/python3.10/site-
packages/sklearn/neural_network/_multilayer_perceptron.py:690:
ConvergenceWarning: Stochastic Optimizer: Maximum iterations (1000) reached and
the optimization hasn't converged yet.
  warnings.warn(
/projects/proteinml/.links/miniconda3/envs/aidep/lib/python3.10/site-
packages/sklearn/neural_network/_multilayer_perceptron.py:690:
ConvergenceWarning: Stochastic Optimizer: Maximum iterations (1000) reached and
the optimization hasn't converged yet.
  warnings.warn(
/projects/proteinml/.links/miniconda3/envs/aidep/lib/python3.10/site-
packages/sklearn/neural_network/_multilayer_perceptron.py:690:
ConvergenceWarning: Stochastic Optimizer: Maximum iterations (1000) reached and
the optimization hasn't converged yet.
  warnings.warn(
/projects/proteinml/.links/miniconda3/envs/aidep/lib/python3.10/site-
packages/sklearn/neural_network/_multilayer_perceptron.py:690:
ConvergenceWarning: Stochastic Optimizer: Maximum iterations (1000) reached and
the optimization hasn't converged yet.
  warnings.warn(
/projects/proteinml/.links/miniconda3/envs/aidep/lib/python3.10/site-
packages/sklearn/neural_network/_multilayer_perceptron.py:690:
ConvergenceWarning: Stochastic Optimizer: Maximum iterations (1000) reached and
the optimization hasn't converged yet.
  warnings.warn(
/projects/proteinml/.links/miniconda3/envs/aidep/lib/python3.10/site-
packages/sklearn/neural_network/_multilayer_perceptron.py:690:
ConvergenceWarning: Stochastic Optimizer: Maximum iterations (1000) reached and
```

```
the optimization hasn't converged yet.
  warnings.warn(
/projects/proteinml/.links/miniconda3/envs/aidep/lib/python3.10/site-
packages/sklearn/neural_network/_multilayer_perceptron.py:690:
ConvergenceWarning: Stochastic Optimizer: Maximum iterations (1000) reached and
the optimization hasn't converged yet.
  warnings.warn(
/projects/proteinml/.links/miniconda3/envs/aidep/lib/python3.10/site-
packages/sklearn/neural_network/_multilayer_perceptron.py:690:
ConvergenceWarning: Stochastic Optimizer: Maximum iterations (1000) reached and
the optimization hasn't converged yet.
  warnings.warn(
/projects/proteinml/.links/miniconda3/envs/aidep/lib/python3.10/site-
packages/sklearn/neural_network/_multilayer_perceptron.py:690:
ConvergenceWarning: Stochastic Optimizer: Maximum iterations (1000) reached and
the optimization hasn't converged yet.
  warnings.warn(
/projects/proteinml/.links/miniconda3/envs/aidep/lib/python3.10/site-
packages/sklearn/neural_network/_multilayer_perceptron.py:690:
ConvergenceWarning: Stochastic Optimizer: Maximum iterations (1000) reached and
the optimization hasn't converged yet.
  warnings.warn(
/projects/proteinml/.links/miniconda3/envs/aidep/lib/python3.10/site-
packages/sklearn/neural_network/_multilayer_perceptron.py:690:
ConvergenceWarning: Stochastic Optimizer: Maximum iterations (1000) reached and
the optimization hasn't converged yet.
  warnings.warn(
/projects/proteinml/.links/miniconda3/envs/aidep/lib/python3.10/site-
packages/sklearn/neural_network/_multilayer_perceptron.py:690:
ConvergenceWarning: Stochastic Optimizer: Maximum iterations (1000) reached and
the optimization hasn't converged yet.
  warnings.warn(
/projects/proteinml/.links/miniconda3/envs/aidep/lib/python3.10/site-
packages/sklearn/neural_network/_multilayer_perceptron.py:690:
ConvergenceWarning: Stochastic Optimizer: Maximum iterations (1000) reached and
the optimization hasn't converged yet.
  warnings.warn(
/projects/proteinml/.links/miniconda3/envs/aidep/lib/python3.10/site-
packages/sklearn/neural_network/_multilayer_perceptron.py:690:
ConvergenceWarning: Stochastic Optimizer: Maximum iterations (1000) reached and
the optimization hasn't converged yet.
  warnings.warn(
/projects/proteinml/.links/miniconda3/envs/aidep/lib/python3.10/site-
packages/sklearn/neural_network/_multilayer_perceptron.py:690:
ConvergenceWarning: Stochastic Optimizer: Maximum iterations (1000) reached and
the optimization hasn't converged yet.
  warnings.warn(
/projects/proteinml/.links/miniconda3/envs/aidep/lib/python3.10/site-
```

```
packages/sklearn/neural_network/_multilayer_perceptron.py:690:
ConvergenceWarning: Stochastic Optimizer: Maximum iterations (1000) reached and
the optimization hasn't converged yet.
  warnings.warn(
/projects/proteinml/.links/miniconda3/envs/aidep/lib/python3.10/site-
packages/sklearn/neural_network/_multilayer_perceptron.py:690:
ConvergenceWarning: Stochastic Optimizer: Maximum iterations (1000) reached and
the optimization hasn't converged yet.
  warnings.warn(
/projects/proteinml/.links/miniconda3/envs/aidep/lib/python3.10/site-
packages/sklearn/neural_network/_multilayer_perceptron.py:690:
ConvergenceWarning: Stochastic Optimizer: Maximum iterations (1000) reached and
the optimization hasn't converged yet.
  warnings.warn(
/projects/proteinml/.links/miniconda3/envs/aidep/lib/python3.10/site-
packages/sklearn/neural_network/_multilayer_perceptron.py:690:
ConvergenceWarning: Stochastic Optimizer: Maximum iterations (1000) reached and
the optimization hasn't converged yet.
  warnings.warn(
/projects/proteinml/.links/miniconda3/envs/aidep/lib/python3.10/site-
packages/sklearn/neural_network/_multilayer_perceptron.py:690:
ConvergenceWarning: Stochastic Optimizer: Maximum iterations (1000) reached and
the optimization hasn't converged yet.
  warnings.warn(
/projects/proteinml/.links/miniconda3/envs/aidep/lib/python3.10/site-
packages/sklearn/neural_network/_multilayer_perceptron.py:690:
ConvergenceWarning: Stochastic Optimizer: Maximum iterations (1000) reached and
the optimization hasn't converged yet.
  warnings.warn(
/projects/proteinml/.links/miniconda3/envs/aidep/lib/python3.10/site-
packages/sklearn/neural_network/_multilayer_perceptron.py:690:
ConvergenceWarning: Stochastic Optimizer: Maximum iterations (1000) reached and
the optimization hasn't converged yet.
  warnings.warn(
/projects/proteinml/.links/miniconda3/envs/aidep/lib/python3.10/site-
packages/sklearn/neural_network/_multilayer_perceptron.py:690:
ConvergenceWarning: Stochastic Optimizer: Maximum iterations (1000) reached and
the optimization hasn't converged yet.
  warnings.warn(
/projects/proteinml/.links/miniconda3/envs/aidep/lib/python3.10/site-
packages/sklearn/neural_network/_multilayer_perceptron.py:690:
ConvergenceWarning: Stochastic Optimizer: Maximum iterations (1000) reached and
the optimization hasn't converged yet.
  warnings.warn(
/projects/proteinml/.links/miniconda3/envs/aidep/lib/python3.10/site-
packages/sklearn/neural_network/_multilayer_perceptron.py:690:
ConvergenceWarning: Stochastic Optimizer: Maximum iterations (1000) reached and
the optimization hasn't converged yet.
```

```
  warnings.warn(
/projects/proteinml/.links/miniconda3/envs/aidep/lib/python3.10/site-
packages/sklearn/neural_network/_multilayer_perceptron.py:690:
ConvergenceWarning: Stochastic Optimizer: Maximum iterations (1000) reached and
the optimization hasn't converged yet.
  warnings.warn(
/projects/proteinml/.links/miniconda3/envs/aidep/lib/python3.10/site-
packages/sklearn/neural_network/_multilayer_perceptron.py:690:
ConvergenceWarning: Stochastic Optimizer: Maximum iterations (1000) reached and
the optimization hasn't converged yet.
  warnings.warn(
/projects/proteinml/.links/miniconda3/envs/aidep/lib/python3.10/site-
packages/sklearn/neural_network/_multilayer_perceptron.py:690:
ConvergenceWarning: Stochastic Optimizer: Maximum iterations (1000) reached and
the optimization hasn't converged yet.
  warnings.warn(
/projects/proteinml/.links/miniconda3/envs/aidep/lib/python3.10/site-
packages/sklearn/neural_network/_multilayer_perceptron.py:690:
ConvergenceWarning: Stochastic Optimizer: Maximum iterations (1000) reached and
the optimization hasn't converged yet.
  warnings.warn(
/projects/proteinml/.links/miniconda3/envs/aidep/lib/python3.10/site-
packages/sklearn/neural_network/_multilayer_perceptron.py:690:
ConvergenceWarning: Stochastic Optimizer: Maximum iterations (1000) reached and
the optimization hasn't converged yet.
  warnings.warn(
/projects/proteinml/.links/miniconda3/envs/aidep/lib/python3.10/site-
packages/sklearn/neural_network/_multilayer_perceptron.py:690:
ConvergenceWarning: Stochastic Optimizer: Maximum iterations (1000) reached and
the optimization hasn't converged yet.
  warnings.warn(
/projects/proteinml/.links/miniconda3/envs/aidep/lib/python3.10/site-
packages/sklearn/neural_network/_multilayer_perceptron.py:690:
ConvergenceWarning: Stochastic Optimizer: Maximum iterations (1000) reached and
the optimization hasn't converged yet.
  warnings.warn(
/projects/proteinml/.links/miniconda3/envs/aidep/lib/python3.10/site-
packages/sklearn/neural_network/_multilayer_perceptron.py:690:
ConvergenceWarning: Stochastic Optimizer: Maximum iterations (1000) reached and
the optimization hasn't converged yet.
  warnings.warn(
/projects/proteinml/.links/miniconda3/envs/aidep/lib/python3.10/site-
packages/sklearn/neural_network/_multilayer_perceptron.py:690:
ConvergenceWarning: Stochastic Optimizer: Maximum iterations (1000) reached and
the optimization hasn't converged yet.
  warnings.warn(
/projects/proteinml/.links/miniconda3/envs/aidep/lib/python3.10/site-
packages/sklearn/neural_network/_multilayer_perceptron.py:690:
```

```
ConvergenceWarning: Stochastic Optimizer: Maximum iterations (1000) reached and
the optimization hasn't converged yet.
  warnings.warn(
/projects/proteinml/.links/miniconda3/envs/aidep/lib/python3.10/site-
packages/sklearn/neural_network/_multilayer_perceptron.py:690:
ConvergenceWarning: Stochastic Optimizer: Maximum iterations (1000) reached and
the optimization hasn't converged yet.
  warnings.warn(
/projects/proteinml/.links/miniconda3/envs/aidep/lib/python3.10/site-
packages/sklearn/neural_network/_multilayer_perceptron.py:690:
ConvergenceWarning: Stochastic Optimizer: Maximum iterations (1000) reached and
the optimization hasn't converged yet.
  warnings.warn(
/projects/proteinml/.links/miniconda3/envs/aidep/lib/python3.10/site-
packages/sklearn/neural_network/_multilayer_perceptron.py:690:
ConvergenceWarning: Stochastic Optimizer: Maximum iterations (1000) reached and
the optimization hasn't converged yet.
  warnings.warn(
/projects/proteinml/.links/miniconda3/envs/aidep/lib/python3.10/site-
packages/sklearn/neural_network/_multilayer_perceptron.py:690:
ConvergenceWarning: Stochastic Optimizer: Maximum iterations (1000) reached and
the optimization hasn't converged yet.
  warnings.warn(
/projects/proteinml/.links/miniconda3/envs/aidep/lib/python3.10/site-
packages/sklearn/neural_network/_multilayer_perceptron.py:690:
ConvergenceWarning: Stochastic Optimizer: Maximum iterations (1000) reached and
the optimization hasn't converged yet.
  warnings.warn(
/projects/proteinml/.links/miniconda3/envs/aidep/lib/python3.10/site-
packages/sklearn/neural_network/_multilayer_perceptron.py:690:
ConvergenceWarning: Stochastic Optimizer: Maximum iterations (1000) reached and
the optimization hasn't converged yet.
  warnings.warn(
/projects/proteinml/.links/miniconda3/envs/aidep/lib/python3.10/site-
packages/sklearn/neural_network/_multilayer_perceptron.py:690:
ConvergenceWarning: Stochastic Optimizer: Maximum iterations (1000) reached and
the optimization hasn't converged yet.
  warnings.warn(
/projects/proteinml/.links/miniconda3/envs/aidep/lib/python3.10/site-
packages/sklearn/neural_network/_multilayer_perceptron.py:690:
ConvergenceWarning: Stochastic Optimizer: Maximum iterations (1000) reached and
the optimization hasn't converged yet.
  warnings.warn(
/projects/proteinml/.links/miniconda3/envs/aidep/lib/python3.10/site-
packages/sklearn/neural_network/_multilayer_perceptron.py:690:
ConvergenceWarning: Stochastic Optimizer: Maximum iterations (1000) reached and
the optimization hasn't converged yet.
  warnings.warn(
```

```
/projects/proteinml/.links/miniconda3/envs/aidep/lib/python3.10/site-
packages/sklearn/neural_network/_multilayer_perceptron.py:690:
ConvergenceWarning: Stochastic Optimizer: Maximum iterations (1000) reached and
the optimization hasn't converged yet.
  warnings.warn(
/projects/proteinml/.links/miniconda3/envs/aidep/lib/python3.10/site-
packages/sklearn/neural_network/_multilayer_perceptron.py:690:
ConvergenceWarning: Stochastic Optimizer: Maximum iterations (1000) reached and
the optimization hasn't converged yet.
  warnings.warn(
/projects/proteinml/.links/miniconda3/envs/aidep/lib/python3.10/site-
packages/sklearn/neural_network/_multilayer_perceptron.py:690:
ConvergenceWarning: Stochastic Optimizer: Maximum iterations (1000) reached and
the optimization hasn't converged yet.
  warnings.warn(
/projects/proteinml/.links/miniconda3/envs/aidep/lib/python3.10/site-
packages/sklearn/neural_network/_multilayer_perceptron.py:690:
ConvergenceWarning: Stochastic Optimizer: Maximum iterations (1000) reached and
the optimization hasn't converged yet.
  warnings.warn(
/projects/proteinml/.links/miniconda3/envs/aidep/lib/python3.10/site-
packages/sklearn/neural_network/_multilayer_perceptron.py:690:
ConvergenceWarning: Stochastic Optimizer: Maximum iterations (1000) reached and
the optimization hasn't converged yet.
  warnings.warn(
/projects/proteinml/.links/miniconda3/envs/aidep/lib/python3.10/site-
packages/sklearn/neural_network/_multilayer_perceptron.py:690:
ConvergenceWarning: Stochastic Optimizer: Maximum iterations (1000) reached and
the optimization hasn't converged yet.
  warnings.warn(
/projects/proteinml/.links/miniconda3/envs/aidep/lib/python3.10/site-
packages/sklearn/neural_network/_multilayer_perceptron.py:690:
ConvergenceWarning: Stochastic Optimizer: Maximum iterations (1000) reached and
the optimization hasn't converged yet.
  warnings.warn(
/projects/proteinml/.links/miniconda3/envs/aidep/lib/python3.10/site-
packages/sklearn/neural_network/_multilayer_perceptron.py:690:
ConvergenceWarning: Stochastic Optimizer: Maximum iterations (1000) reached and
the optimization hasn't converged yet.
  warnings.warn(
/projects/proteinml/.links/miniconda3/envs/aidep/lib/python3.10/site-
packages/sklearn/neural_network/_multilayer_perceptron.py:690:
ConvergenceWarning: Stochastic Optimizer: Maximum iterations (1000) reached and
the optimization hasn't converged yet.
  warnings.warn(
/projects/proteinml/.links/miniconda3/envs/aidep/lib/python3.10/site-
packages/sklearn/neural_network/_multilayer_perceptron.py:690:
ConvergenceWarning: Stochastic Optimizer: Maximum iterations (1000) reached and
```

```
the optimization hasn't converged yet.
  warnings.warn(
/projects/proteinml/.links/miniconda3/envs/aidep/lib/python3.10/site-
packages/sklearn/neural_network/_multilayer_perceptron.py:690:
ConvergenceWarning: Stochastic Optimizer: Maximum iterations (1000) reached and
the optimization hasn't converged yet.
  warnings.warn(
/projects/proteinml/.links/miniconda3/envs/aidep/lib/python3.10/site-
packages/sklearn/neural_network/_multilayer_perceptron.py:690:
ConvergenceWarning: Stochastic Optimizer: Maximum iterations (1000) reached and
the optimization hasn't converged yet.
  warnings.warn(
/projects/proteinml/.links/miniconda3/envs/aidep/lib/python3.10/site-
packages/sklearn/neural_network/_multilayer_perceptron.py:690:
ConvergenceWarning: Stochastic Optimizer: Maximum iterations (1000) reached and
the optimization hasn't converged yet.
  warnings.warn(
/projects/proteinml/.links/miniconda3/envs/aidep/lib/python3.10/site-
packages/sklearn/neural_network/_multilayer_perceptron.py:690:
ConvergenceWarning: Stochastic Optimizer: Maximum iterations (1000) reached and
the optimization hasn't converged yet.
  warnings.warn(
/projects/proteinml/.links/miniconda3/envs/aidep/lib/python3.10/site-
packages/sklearn/neural_network/_multilayer_perceptron.py:690:
ConvergenceWarning: Stochastic Optimizer: Maximum iterations (1000) reached and
the optimization hasn't converged yet.
  warnings.warn(
/projects/proteinml/.links/miniconda3/envs/aidep/lib/python3.10/site-
packages/sklearn/neural_network/_multilayer_perceptron.py:690:
ConvergenceWarning: Stochastic Optimizer: Maximum iterations (1000) reached and
the optimization hasn't converged yet.
  warnings.warn(
/projects/proteinml/.links/miniconda3/envs/aidep/lib/python3.10/site-
packages/sklearn/neural_network/_multilayer_perceptron.py:690:
ConvergenceWarning: Stochastic Optimizer: Maximum iterations (1000) reached and
the optimization hasn't converged yet.
  warnings.warn(
/projects/proteinml/.links/miniconda3/envs/aidep/lib/python3.10/site-
packages/sklearn/neural_network/_multilayer_perceptron.py:690:
ConvergenceWarning: Stochastic Optimizer: Maximum iterations (1000) reached and
the optimization hasn't converged yet.
  warnings.warn(
/projects/proteinml/.links/miniconda3/envs/aidep/lib/python3.10/site-
packages/sklearn/neural_network/_multilayer_perceptron.py:690:
ConvergenceWarning: Stochastic Optimizer: Maximum iterations (1000) reached and
the optimization hasn't converged yet.
  warnings.warn(
/projects/proteinml/.links/miniconda3/envs/aidep/lib/python3.10/site-
```

```
packages/sklearn/neural_network/_multilayer_perceptron.py:690:
ConvergenceWarning: Stochastic Optimizer: Maximum iterations (1000) reached and
the optimization hasn't converged yet.
  warnings.warn(
/projects/proteinml/.links/miniconda3/envs/aidep/lib/python3.10/site-
packages/sklearn/neural_network/_multilayer_perceptron.py:690:
ConvergenceWarning: Stochastic Optimizer: Maximum iterations (1000) reached and
the optimization hasn't converged yet.
  warnings.warn(
/projects/proteinml/.links/miniconda3/envs/aidep/lib/python3.10/site-
packages/sklearn/neural_network/_multilayer_perceptron.py:690:
ConvergenceWarning: Stochastic Optimizer: Maximum iterations (1000) reached and
the optimization hasn't converged yet.
  warnings.warn(
/projects/proteinml/.links/miniconda3/envs/aidep/lib/python3.10/site-
packages/sklearn/neural_network/_multilayer_perceptron.py:690:
ConvergenceWarning: Stochastic Optimizer: Maximum iterations (1000) reached and
the optimization hasn't converged yet.
  warnings.warn(
/projects/proteinml/.links/miniconda3/envs/aidep/lib/python3.10/site-
packages/sklearn/neural_network/_multilayer_perceptron.py:690:
ConvergenceWarning: Stochastic Optimizer: Maximum iterations (1000) reached and
the optimization hasn't converged yet.
  warnings.warn(
/projects/proteinml/.links/miniconda3/envs/aidep/lib/python3.10/site-
packages/sklearn/neural_network/_multilayer_perceptron.py:690:
ConvergenceWarning: Stochastic Optimizer: Maximum iterations (1000) reached and
the optimization hasn't converged yet.
  warnings.warn(
/projects/proteinml/.links/miniconda3/envs/aidep/lib/python3.10/site-
packages/sklearn/neural_network/_multilayer_perceptron.py:690:
ConvergenceWarning: Stochastic Optimizer: Maximum iterations (1000) reached and
the optimization hasn't converged yet.
  warnings.warn(
/projects/proteinml/.links/miniconda3/envs/aidep/lib/python3.10/site-
packages/sklearn/neural_network/_multilayer_perceptron.py:690:
ConvergenceWarning: Stochastic Optimizer: Maximum iterations (1000) reached and
the optimization hasn't converged yet.
  warnings.warn(
/projects/proteinml/.links/miniconda3/envs/aidep/lib/python3.10/site-
packages/sklearn/neural_network/_multilayer_perceptron.py:690:
ConvergenceWarning: Stochastic Optimizer: Maximum iterations (1000) reached and
the optimization hasn't converged yet.
  warnings.warn(
/projects/proteinml/.links/miniconda3/envs/aidep/lib/python3.10/site-
packages/sklearn/neural_network/_multilayer_perceptron.py:690:
ConvergenceWarning: Stochastic Optimizer: Maximum iterations (1000) reached and
the optimization hasn't converged yet.
```

```
  warnings.warn(
/projects/proteinml/.links/miniconda3/envs/aidep/lib/python3.10/site-
packages/sklearn/neural_network/_multilayer_perceptron.py:690:
ConvergenceWarning: Stochastic Optimizer: Maximum iterations (1000) reached and
the optimization hasn't converged yet.
  warnings.warn(
/projects/proteinml/.links/miniconda3/envs/aidep/lib/python3.10/site-
packages/sklearn/neural_network/_multilayer_perceptron.py:690:
ConvergenceWarning: Stochastic Optimizer: Maximum iterations (1000) reached and
the optimization hasn't converged yet.
  warnings.warn(
/projects/proteinml/.links/miniconda3/envs/aidep/lib/python3.10/site-
packages/sklearn/neural_network/_multilayer_perceptron.py:690:
ConvergenceWarning: Stochastic Optimizer: Maximum iterations (1000) reached and
the optimization hasn't converged yet.
  warnings.warn(
/projects/proteinml/.links/miniconda3/envs/aidep/lib/python3.10/site-
packages/sklearn/neural_network/_multilayer_perceptron.py:690:
ConvergenceWarning: Stochastic Optimizer: Maximum iterations (1000) reached and
the optimization hasn't converged yet.
  warnings.warn(
/projects/proteinml/.links/miniconda3/envs/aidep/lib/python3.10/site-
packages/sklearn/neural_network/_multilayer_perceptron.py:690:
ConvergenceWarning: Stochastic Optimizer: Maximum iterations (1000) reached and
the optimization hasn't converged yet.
  warnings.warn(
/projects/proteinml/.links/miniconda3/envs/aidep/lib/python3.10/site-
packages/sklearn/neural_network/_multilayer_perceptron.py:690:
ConvergenceWarning: Stochastic Optimizer: Maximum iterations (1000) reached and
the optimization hasn't converged yet.
  warnings.warn(
/projects/proteinml/.links/miniconda3/envs/aidep/lib/python3.10/site-
packages/sklearn/neural_network/_multilayer_perceptron.py:690:
ConvergenceWarning: Stochastic Optimizer: Maximum iterations (1000) reached and
the optimization hasn't converged yet.
  warnings.warn(
/projects/proteinml/.links/miniconda3/envs/aidep/lib/python3.10/site-
packages/sklearn/neural_network/_multilayer_perceptron.py:690:
ConvergenceWarning: Stochastic Optimizer: Maximum iterations (1000) reached and
the optimization hasn't converged yet.
  warnings.warn(
/projects/proteinml/.links/miniconda3/envs/aidep/lib/python3.10/site-
packages/sklearn/neural_network/_multilayer_perceptron.py:690:
ConvergenceWarning: Stochastic Optimizer: Maximum iterations (1000) reached and
the optimization hasn't converged yet.
  warnings.warn(
/projects/proteinml/.links/miniconda3/envs/aidep/lib/python3.10/site-
packages/sklearn/neural_network/_multilayer_perceptron.py:690:
```

```
ConvergenceWarning: Stochastic Optimizer: Maximum iterations (1000) reached and
the optimization hasn't converged yet.
  warnings.warn(
/projects/proteinml/.links/miniconda3/envs/aidep/lib/python3.10/site-
packages/sklearn/neural_network/_multilayer_perceptron.py:690:
ConvergenceWarning: Stochastic Optimizer: Maximum iterations (1000) reached and
the optimization hasn't converged yet.
  warnings.warn(
/projects/proteinml/.links/miniconda3/envs/aidep/lib/python3.10/site-
packages/sklearn/neural_network/_multilayer_perceptron.py:690:
ConvergenceWarning: Stochastic Optimizer: Maximum iterations (1000) reached and
the optimization hasn't converged yet.
  warnings.warn(
/projects/proteinml/.links/miniconda3/envs/aidep/lib/python3.10/site-
packages/sklearn/neural_network/_multilayer_perceptron.py:690:
ConvergenceWarning: Stochastic Optimizer: Maximum iterations (1000) reached and
the optimization hasn't converged yet.
  warnings.warn(
/projects/proteinml/.links/miniconda3/envs/aidep/lib/python3.10/site-
packages/sklearn/neural_network/_multilayer_perceptron.py:690:
ConvergenceWarning: Stochastic Optimizer: Maximum iterations (1000) reached and
the optimization hasn't converged yet.
  warnings.warn(
/projects/proteinml/.links/miniconda3/envs/aidep/lib/python3.10/site-
packages/sklearn/neural_network/_multilayer_perceptron.py:690:
ConvergenceWarning: Stochastic Optimizer: Maximum iterations (1000) reached and
the optimization hasn't converged yet.
  warnings.warn(
/projects/proteinml/.links/miniconda3/envs/aidep/lib/python3.10/site-
packages/sklearn/neural_network/_multilayer_perceptron.py:690:
ConvergenceWarning: Stochastic Optimizer: Maximum iterations (1000) reached and
the optimization hasn't converged yet.
  warnings.warn(
/projects/proteinml/.links/miniconda3/envs/aidep/lib/python3.10/site-
packages/sklearn/neural_network/_multilayer_perceptron.py:690:
ConvergenceWarning: Stochastic Optimizer: Maximum iterations (1000) reached and
the optimization hasn't converged yet.
  warnings.warn(
/projects/proteinml/.links/miniconda3/envs/aidep/lib/python3.10/site-
packages/sklearn/neural_network/_multilayer_perceptron.py:690:
ConvergenceWarning: Stochastic Optimizer: Maximum iterations (1000) reached and
the optimization hasn't converged yet.
  warnings.warn(
/projects/proteinml/.links/miniconda3/envs/aidep/lib/python3.10/site-
packages/sklearn/neural_network/_multilayer_perceptron.py:690:
ConvergenceWarning: Stochastic Optimizer: Maximum iterations (1000) reached and
the optimization hasn't converged yet.
  warnings.warn(
```

```
/projects/proteinml/.links/miniconda3/envs/aidep/lib/python3.10/site-
packages/sklearn/neural_network/_multilayer_perceptron.py:690:
ConvergenceWarning: Stochastic Optimizer: Maximum iterations (1000) reached and
the optimization hasn't converged yet.
  warnings.warn(
/projects/proteinml/.links/miniconda3/envs/aidep/lib/python3.10/site-
packages/sklearn/neural_network/_multilayer_perceptron.py:690:
ConvergenceWarning: Stochastic Optimizer: Maximum iterations (1000) reached and
the optimization hasn't converged yet.
  warnings.warn(
/projects/proteinml/.links/miniconda3/envs/aidep/lib/python3.10/site-
packages/sklearn/neural_network/_multilayer_perceptron.py:690:
ConvergenceWarning: Stochastic Optimizer: Maximum iterations (1000) reached and
the optimization hasn't converged yet.
  warnings.warn(
/projects/proteinml/.links/miniconda3/envs/aidep/lib/python3.10/site-
packages/sklearn/neural_network/_multilayer_perceptron.py:690:
ConvergenceWarning: Stochastic Optimizer: Maximum iterations (1000) reached and
the optimization hasn't converged yet.
  warnings.warn(
/projects/proteinml/.links/miniconda3/envs/aidep/lib/python3.10/site-
packages/sklearn/neural_network/_multilayer_perceptron.py:690:
ConvergenceWarning: Stochastic Optimizer: Maximum iterations (1000) reached and
the optimization hasn't converged yet.
  warnings.warn(
/projects/proteinml/.links/miniconda3/envs/aidep/lib/python3.10/site-
packages/sklearn/neural_network/_multilayer_perceptron.py:690:
ConvergenceWarning: Stochastic Optimizer: Maximum iterations (1000) reached and
the optimization hasn't converged yet.
  warnings.warn(
/projects/proteinml/.links/miniconda3/envs/aidep/lib/python3.10/site-
packages/sklearn/neural_network/_multilayer_perceptron.py:690:
ConvergenceWarning: Stochastic Optimizer: Maximum iterations (1000) reached and
the optimization hasn't converged yet.
  warnings.warn(
/projects/proteinml/.links/miniconda3/envs/aidep/lib/python3.10/site-
packages/sklearn/neural_network/_multilayer_perceptron.py:690:
ConvergenceWarning: Stochastic Optimizer: Maximum iterations (1000) reached and
the optimization hasn't converged yet.
  warnings.warn(
/projects/proteinml/.links/miniconda3/envs/aidep/lib/python3.10/site-
packages/sklearn/neural_network/_multilayer_perceptron.py:690:
ConvergenceWarning: Stochastic Optimizer: Maximum iterations (1000) reached and
the optimization hasn't converged yet.
  warnings.warn(
/projects/proteinml/.links/miniconda3/envs/aidep/lib/python3.10/site-
packages/sklearn/neural_network/_multilayer_perceptron.py:690:
ConvergenceWarning: Stochastic Optimizer: Maximum iterations (1000) reached and
```

```
the optimization hasn't converged yet.
  warnings.warn(
/projects/proteinml/.links/miniconda3/envs/aidep/lib/python3.10/site-
packages/sklearn/neural_network/_multilayer_perceptron.py:690:
ConvergenceWarning: Stochastic Optimizer: Maximum iterations (1000) reached and
the optimization hasn't converged yet.
  warnings.warn(
/projects/proteinml/.links/miniconda3/envs/aidep/lib/python3.10/site-
packages/sklearn/neural_network/_multilayer_perceptron.py:690:
ConvergenceWarning: Stochastic Optimizer: Maximum iterations (1000) reached and
the optimization hasn't converged yet.
  warnings.warn(
/projects/proteinml/.links/miniconda3/envs/aidep/lib/python3.10/site-
packages/sklearn/neural_network/_multilayer_perceptron.py:690:
ConvergenceWarning: Stochastic Optimizer: Maximum iterations (1000) reached and
the optimization hasn't converged yet.
  warnings.warn(
/projects/proteinml/.links/miniconda3/envs/aidep/lib/python3.10/site-
packages/sklearn/neural_network/_multilayer_perceptron.py:690:
ConvergenceWarning: Stochastic Optimizer: Maximum iterations (1000) reached and
the optimization hasn't converged yet.
  warnings.warn(
/projects/proteinml/.links/miniconda3/envs/aidep/lib/python3.10/site-
packages/sklearn/neural_network/_multilayer_perceptron.py:690:
ConvergenceWarning: Stochastic Optimizer: Maximum iterations (1000) reached and
the optimization hasn't converged yet.
  warnings.warn(
/projects/proteinml/.links/miniconda3/envs/aidep/lib/python3.10/site-
packages/sklearn/neural_network/_multilayer_perceptron.py:690:
ConvergenceWarning: Stochastic Optimizer: Maximum iterations (1000) reached and
the optimization hasn't converged yet.
  warnings.warn(
/projects/proteinml/.links/miniconda3/envs/aidep/lib/python3.10/site-
packages/sklearn/neural_network/_multilayer_perceptron.py:690:
ConvergenceWarning: Stochastic Optimizer: Maximum iterations (1000) reached and
the optimization hasn't converged yet.
  warnings.warn(
/projects/proteinml/.links/miniconda3/envs/aidep/lib/python3.10/site-
packages/sklearn/neural_network/_multilayer_perceptron.py:690:
ConvergenceWarning: Stochastic Optimizer: Maximum iterations (1000) reached and
the optimization hasn't converged yet.
  warnings.warn(
/projects/proteinml/.links/miniconda3/envs/aidep/lib/python3.10/site-
packages/sklearn/neural_network/_multilayer_perceptron.py:690:
ConvergenceWarning: Stochastic Optimizer: Maximum iterations (1000) reached and
the optimization hasn't converged yet.
  warnings.warn(
/projects/proteinml/.links/miniconda3/envs/aidep/lib/python3.10/site-
```

```
packages/sklearn/neural_network/_multilayer_perceptron.py:690:
ConvergenceWarning: Stochastic Optimizer: Maximum iterations (1000) reached and
the optimization hasn't converged yet.
  warnings.warn(
/projects/proteinml/.links/miniconda3/envs/aidep/lib/python3.10/site-
packages/sklearn/neural_network/_multilayer_perceptron.py:690:
ConvergenceWarning: Stochastic Optimizer: Maximum iterations (1000) reached and
the optimization hasn't converged yet.
  warnings.warn(
/projects/proteinml/.links/miniconda3/envs/aidep/lib/python3.10/site-
packages/sklearn/neural_network/_multilayer_perceptron.py:690:
ConvergenceWarning: Stochastic Optimizer: Maximum iterations (1000) reached and
the optimization hasn't converged yet.
  warnings.warn(
/projects/proteinml/.links/miniconda3/envs/aidep/lib/python3.10/site-
packages/sklearn/neural_network/_multilayer_perceptron.py:690:
ConvergenceWarning: Stochastic Optimizer: Maximum iterations (1000) reached and
the optimization hasn't converged yet.
  warnings.warn(
/projects/proteinml/.links/miniconda3/envs/aidep/lib/python3.10/site-
packages/sklearn/neural_network/_multilayer_perceptron.py:690:
ConvergenceWarning: Stochastic Optimizer: Maximum iterations (1000) reached and
the optimization hasn't converged yet.
  warnings.warn(
/projects/proteinml/.links/miniconda3/envs/aidep/lib/python3.10/site-
packages/sklearn/neural_network/_multilayer_perceptron.py:690:
ConvergenceWarning: Stochastic Optimizer: Maximum iterations (1000) reached and
the optimization hasn't converged yet.
  warnings.warn(
/projects/proteinml/.links/miniconda3/envs/aidep/lib/python3.10/site-
packages/sklearn/neural_network/_multilayer_perceptron.py:690:
ConvergenceWarning: Stochastic Optimizer: Maximum iterations (1000) reached and
the optimization hasn't converged yet.
  warnings.warn(
/projects/proteinml/.links/miniconda3/envs/aidep/lib/python3.10/site-
packages/sklearn/neural_network/_multilayer_perceptron.py:690:
ConvergenceWarning: Stochastic Optimizer: Maximum iterations (1000) reached and
the optimization hasn't converged yet.
  warnings.warn(
/projects/proteinml/.links/miniconda3/envs/aidep/lib/python3.10/site-
packages/sklearn/neural_network/_multilayer_perceptron.py:690:
ConvergenceWarning: Stochastic Optimizer: Maximum iterations (1000) reached and
the optimization hasn't converged yet.
  warnings.warn(
/projects/proteinml/.links/miniconda3/envs/aidep/lib/python3.10/site-
packages/sklearn/neural_network/_multilayer_perceptron.py:690:
ConvergenceWarning: Stochastic Optimizer: Maximum iterations (1000) reached and
the optimization hasn't converged yet.
```

```
  warnings.warn(
/projects/proteinml/.links/miniconda3/envs/aidep/lib/python3.10/site-
packages/sklearn/neural_network/_multilayer_perceptron.py:690:
ConvergenceWarning: Stochastic Optimizer: Maximum iterations (1000) reached and
the optimization hasn't converged yet.
  warnings.warn(
/projects/proteinml/.links/miniconda3/envs/aidep/lib/python3.10/site-
packages/sklearn/neural_network/_multilayer_perceptron.py:690:
ConvergenceWarning: Stochastic Optimizer: Maximum iterations (1000) reached and
the optimization hasn't converged yet.
  warnings.warn(
/projects/proteinml/.links/miniconda3/envs/aidep/lib/python3.10/site-
packages/sklearn/neural_network/_multilayer_perceptron.py:690:
ConvergenceWarning: Stochastic Optimizer: Maximum iterations (1000) reached and
the optimization hasn't converged yet.
  warnings.warn(
/projects/proteinml/.links/miniconda3/envs/aidep/lib/python3.10/site-
packages/sklearn/neural_network/_multilayer_perceptron.py:690:
ConvergenceWarning: Stochastic Optimizer: Maximum iterations (1000) reached and
the optimization hasn't converged yet.
  warnings.warn(
/projects/proteinml/.links/miniconda3/envs/aidep/lib/python3.10/site-
packages/sklearn/neural_network/_multilayer_perceptron.py:690:
ConvergenceWarning: Stochastic Optimizer: Maximum iterations (1000) reached and
the optimization hasn't converged yet.
  warnings.warn(
/projects/proteinml/.links/miniconda3/envs/aidep/lib/python3.10/site-
packages/sklearn/neural_network/_multilayer_perceptron.py:690:
ConvergenceWarning: Stochastic Optimizer: Maximum iterations (1000) reached and
the optimization hasn't converged yet.
  warnings.warn(
/projects/proteinml/.links/miniconda3/envs/aidep/lib/python3.10/site-
packages/sklearn/neural_network/_multilayer_perceptron.py:690:
ConvergenceWarning: Stochastic Optimizer: Maximum iterations (1000) reached and
the optimization hasn't converged yet.
  warnings.warn(
/projects/proteinml/.links/miniconda3/envs/aidep/lib/python3.10/site-
packages/sklearn/neural_network/_multilayer_perceptron.py:690:
ConvergenceWarning: Stochastic Optimizer: Maximum iterations (1000) reached and
the optimization hasn't converged yet.
  warnings.warn(
/projects/proteinml/.links/miniconda3/envs/aidep/lib/python3.10/site-
packages/sklearn/neural_network/_multilayer_perceptron.py:690:
ConvergenceWarning: Stochastic Optimizer: Maximum iterations (1000) reached and
the optimization hasn't converged yet.
  warnings.warn(
/projects/proteinml/.links/miniconda3/envs/aidep/lib/python3.10/site-
packages/sklearn/neural_network/_multilayer_perceptron.py:690:
```

```
ConvergenceWarning: Stochastic Optimizer: Maximum iterations (1000) reached and
the optimization hasn't converged yet.
  warnings.warn(
/projects/proteinml/.links/miniconda3/envs/aidep/lib/python3.10/site-
packages/sklearn/neural_network/_multilayer_perceptron.py:690:
ConvergenceWarning: Stochastic Optimizer: Maximum iterations (1000) reached and
the optimization hasn't converged yet.
  warnings.warn(
/projects/proteinml/.links/miniconda3/envs/aidep/lib/python3.10/site-
packages/sklearn/neural_network/_multilayer_perceptron.py:690:
ConvergenceWarning: Stochastic Optimizer: Maximum iterations (1000) reached and
the optimization hasn't converged yet.
  warnings.warn(
/projects/proteinml/.links/miniconda3/envs/aidep/lib/python3.10/site-
packages/sklearn/neural_network/_multilayer_perceptron.py:690:
ConvergenceWarning: Stochastic Optimizer: Maximum iterations (1000) reached and
the optimization hasn't converged yet.
  warnings.warn(
/projects/proteinml/.links/miniconda3/envs/aidep/lib/python3.10/site-
packages/sklearn/neural_network/_multilayer_perceptron.py:690:
ConvergenceWarning: Stochastic Optimizer: Maximum iterations (1000) reached and
the optimization hasn't converged yet.
  warnings.warn(
/projects/proteinml/.links/miniconda3/envs/aidep/lib/python3.10/site-
packages/sklearn/neural_network/_multilayer_perceptron.py:690:
ConvergenceWarning: Stochastic Optimizer: Maximum iterations (1000) reached and
the optimization hasn't converged yet.
  warnings.warn(
/projects/proteinml/.links/miniconda3/envs/aidep/lib/python3.10/site-
packages/sklearn/neural_network/_multilayer_perceptron.py:690:
ConvergenceWarning: Stochastic Optimizer: Maximum iterations (1000) reached and
the optimization hasn't converged yet.
  warnings.warn(
/projects/proteinml/.links/miniconda3/envs/aidep/lib/python3.10/site-
packages/sklearn/neural_network/_multilayer_perceptron.py:690:
ConvergenceWarning: Stochastic Optimizer: Maximum iterations (1000) reached and
the optimization hasn't converged yet.
  warnings.warn(
/projects/proteinml/.links/miniconda3/envs/aidep/lib/python3.10/site-
packages/sklearn/neural_network/_multilayer_perceptron.py:690:
ConvergenceWarning: Stochastic Optimizer: Maximum iterations (1000) reached and
the optimization hasn't converged yet.
  warnings.warn(
/projects/proteinml/.links/miniconda3/envs/aidep/lib/python3.10/site-
packages/sklearn/neural_network/_multilayer_perceptron.py:690:
ConvergenceWarning: Stochastic Optimizer: Maximum iterations (1000) reached and
the optimization hasn't converged yet.
  warnings.warn(
```

[21]: ['none_esmfull_mlp_scores.joblib']

1.5.6 3.5 ESM2 embeddings, only changing residues flattened features, linear

[23]:
```
esm2_embedder_only4 = ap.ESM2Embedding(
    pool=False,
    flatten=True,
    metadata_folder=os.path.join('.', 'data', 'epistatic',␣
↪'esm2emb_design_space'),
    device='cuda:1',
    model_checkpoint='esm2_t12_35M_UR50D',
    use_cache=True,
    batch_size=180,
    positions=library_positions
)
esm2_embedder_only4.fit()
X_esm_only4 = esm2_embedder_only4.transform(X)
```

```
Some weights of EsmModel were not initialized from the model checkpoint at
facebook/esm2_t12_35M_UR50D and are newly initialized: ['pooler.dense.bias',
'pooler.dense.weight']
You should probably TRAIN this model on a down-stream task to be able to use it
for predictions and inference.
INFO:aide_predict.bespoke_models.base:Transforming input using ESM2Embedding
INFO:aide_predict.bespoke_models.base:Found 159129 cached results, running on 0
uncached sequences.
```

[26]:
```
none_esm4sites_linear_scores = hyperopt_and_scoring(linear_pca, X_esm_only4, y,␣
↪linear_param_space)
joblib.dump(none_esm4sites_linear_scores, 'none_esm4sites_linear_scores.joblib')
```

[26]: ['none_esm4sites_linear_scores.joblib']

1.5.7 3.6 ESM2 embeddings, only changing residues flattened, MLP

[27]:
```
none_esm4sites_mlp_scores = hyperopt_and_scoring(mlp_pca, X_esm_only4, y,␣
↪mlp_param_space)
joblib.dump(none_esm4sites_mlp_scores, 'none_esm4sites_mlp_scores.joblib')
```

```
/projects/proteinml/.links/miniconda3/envs/aidep/lib/python3.10/site-
packages/sklearn/neural_network/_multilayer_perceptron.py:690:
ConvergenceWarning: Stochastic Optimizer: Maximum iterations (1000) reached and
the optimization hasn't converged yet.
  warnings.warn(
/projects/proteinml/.links/miniconda3/envs/aidep/lib/python3.10/site-
packages/sklearn/neural_network/_multilayer_perceptron.py:690:
ConvergenceWarning: Stochastic Optimizer: Maximum iterations (1000) reached and
```

```
the optimization hasn't converged yet.
  warnings.warn(
/projects/proteinml/.links/miniconda3/envs/aidep/lib/python3.10/site-
packages/sklearn/neural_network/_multilayer_perceptron.py:690:
ConvergenceWarning: Stochastic Optimizer: Maximum iterations (1000) reached and
the optimization hasn't converged yet.
  warnings.warn(
/projects/proteinml/.links/miniconda3/envs/aidep/lib/python3.10/site-
packages/sklearn/neural_network/_multilayer_perceptron.py:690:
ConvergenceWarning: Stochastic Optimizer: Maximum iterations (1000) reached and
the optimization hasn't converged yet.
  warnings.warn(
/projects/proteinml/.links/miniconda3/envs/aidep/lib/python3.10/site-
packages/sklearn/neural_network/_multilayer_perceptron.py:690:
ConvergenceWarning: Stochastic Optimizer: Maximum iterations (1000) reached and
the optimization hasn't converged yet.
  warnings.warn(
/projects/proteinml/.links/miniconda3/envs/aidep/lib/python3.10/site-
packages/sklearn/neural_network/_multilayer_perceptron.py:690:
ConvergenceWarning: Stochastic Optimizer: Maximum iterations (1000) reached and
the optimization hasn't converged yet.
  warnings.warn(
/projects/proteinml/.links/miniconda3/envs/aidep/lib/python3.10/site-
packages/sklearn/neural_network/_multilayer_perceptron.py:690:
ConvergenceWarning: Stochastic Optimizer: Maximum iterations (1000) reached and
the optimization hasn't converged yet.
  warnings.warn(
/projects/proteinml/.links/miniconda3/envs/aidep/lib/python3.10/site-
packages/sklearn/neural_network/_multilayer_perceptron.py:690:
ConvergenceWarning: Stochastic Optimizer: Maximum iterations (1000) reached and
the optimization hasn't converged yet.
  warnings.warn(
/projects/proteinml/.links/miniconda3/envs/aidep/lib/python3.10/site-
packages/sklearn/neural_network/_multilayer_perceptron.py:690:
ConvergenceWarning: Stochastic Optimizer: Maximum iterations (1000) reached and
the optimization hasn't converged yet.
  warnings.warn(
/projects/proteinml/.links/miniconda3/envs/aidep/lib/python3.10/site-
packages/sklearn/neural_network/_multilayer_perceptron.py:690:
ConvergenceWarning: Stochastic Optimizer: Maximum iterations (1000) reached and
the optimization hasn't converged yet.
  warnings.warn(
/projects/proteinml/.links/miniconda3/envs/aidep/lib/python3.10/site-
packages/sklearn/neural_network/_multilayer_perceptron.py:690:
ConvergenceWarning: Stochastic Optimizer: Maximum iterations (1000) reached and
the optimization hasn't converged yet.
  warnings.warn(
/projects/proteinml/.links/miniconda3/envs/aidep/lib/python3.10/site-
```

```
packages/sklearn/neural_network/_multilayer_perceptron.py:690:
ConvergenceWarning: Stochastic Optimizer: Maximum iterations (1000) reached and
the optimization hasn't converged yet.
  warnings.warn(
/projects/proteinml/.links/miniconda3/envs/aidep/lib/python3.10/site-
packages/sklearn/neural_network/_multilayer_perceptron.py:690:
ConvergenceWarning: Stochastic Optimizer: Maximum iterations (1000) reached and
the optimization hasn't converged yet.
  warnings.warn(
/projects/proteinml/.links/miniconda3/envs/aidep/lib/python3.10/site-
packages/sklearn/neural_network/_multilayer_perceptron.py:690:
ConvergenceWarning: Stochastic Optimizer: Maximum iterations (1000) reached and
the optimization hasn't converged yet.
  warnings.warn(
/projects/proteinml/.links/miniconda3/envs/aidep/lib/python3.10/site-
packages/sklearn/neural_network/_multilayer_perceptron.py:690:
ConvergenceWarning: Stochastic Optimizer: Maximum iterations (1000) reached and
the optimization hasn't converged yet.
  warnings.warn(
/projects/proteinml/.links/miniconda3/envs/aidep/lib/python3.10/site-
packages/sklearn/neural_network/_multilayer_perceptron.py:690:
ConvergenceWarning: Stochastic Optimizer: Maximum iterations (1000) reached and
the optimization hasn't converged yet.
  warnings.warn(
/projects/proteinml/.links/miniconda3/envs/aidep/lib/python3.10/site-
packages/sklearn/neural_network/_multilayer_perceptron.py:690:
ConvergenceWarning: Stochastic Optimizer: Maximum iterations (1000) reached and
the optimization hasn't converged yet.
  warnings.warn(
/projects/proteinml/.links/miniconda3/envs/aidep/lib/python3.10/site-
packages/sklearn/neural_network/_multilayer_perceptron.py:690:
ConvergenceWarning: Stochastic Optimizer: Maximum iterations (1000) reached and
the optimization hasn't converged yet.
  warnings.warn(
/projects/proteinml/.links/miniconda3/envs/aidep/lib/python3.10/site-
packages/sklearn/neural_network/_multilayer_perceptron.py:690:
ConvergenceWarning: Stochastic Optimizer: Maximum iterations (1000) reached and
the optimization hasn't converged yet.
  warnings.warn(
/projects/proteinml/.links/miniconda3/envs/aidep/lib/python3.10/site-
packages/sklearn/neural_network/_multilayer_perceptron.py:690:
ConvergenceWarning: Stochastic Optimizer: Maximum iterations (1000) reached and
the optimization hasn't converged yet.
  warnings.warn(
/projects/proteinml/.links/miniconda3/envs/aidep/lib/python3.10/site-
packages/sklearn/neural_network/_multilayer_perceptron.py:690:
ConvergenceWarning: Stochastic Optimizer: Maximum iterations (1000) reached and
the optimization hasn't converged yet.
```

```
  warnings.warn(
/projects/proteinml/.links/miniconda3/envs/aidep/lib/python3.10/site-
packages/sklearn/neural_network/_multilayer_perceptron.py:690:
ConvergenceWarning: Stochastic Optimizer: Maximum iterations (1000) reached and
the optimization hasn't converged yet.
  warnings.warn(
/projects/proteinml/.links/miniconda3/envs/aidep/lib/python3.10/site-
packages/sklearn/neural_network/_multilayer_perceptron.py:690:
ConvergenceWarning: Stochastic Optimizer: Maximum iterations (1000) reached and
the optimization hasn't converged yet.
  warnings.warn(
/projects/proteinml/.links/miniconda3/envs/aidep/lib/python3.10/site-
packages/sklearn/neural_network/_multilayer_perceptron.py:690:
ConvergenceWarning: Stochastic Optimizer: Maximum iterations (1000) reached and
the optimization hasn't converged yet.
  warnings.warn(
/projects/proteinml/.links/miniconda3/envs/aidep/lib/python3.10/site-
packages/sklearn/neural_network/_multilayer_perceptron.py:690:
ConvergenceWarning: Stochastic Optimizer: Maximum iterations (1000) reached and
the optimization hasn't converged yet.
  warnings.warn(
/projects/proteinml/.links/miniconda3/envs/aidep/lib/python3.10/site-
packages/sklearn/neural_network/_multilayer_perceptron.py:690:
ConvergenceWarning: Stochastic Optimizer: Maximum iterations (1000) reached and
the optimization hasn't converged yet.
  warnings.warn(
/projects/proteinml/.links/miniconda3/envs/aidep/lib/python3.10/site-
packages/sklearn/neural_network/_multilayer_perceptron.py:690:
ConvergenceWarning: Stochastic Optimizer: Maximum iterations (1000) reached and
the optimization hasn't converged yet.
  warnings.warn(
/projects/proteinml/.links/miniconda3/envs/aidep/lib/python3.10/site-
packages/sklearn/neural_network/_multilayer_perceptron.py:690:
ConvergenceWarning: Stochastic Optimizer: Maximum iterations (1000) reached and
the optimization hasn't converged yet.
  warnings.warn(
/projects/proteinml/.links/miniconda3/envs/aidep/lib/python3.10/site-
packages/sklearn/neural_network/_multilayer_perceptron.py:690:
ConvergenceWarning: Stochastic Optimizer: Maximum iterations (1000) reached and
the optimization hasn't converged yet.
  warnings.warn(
/projects/proteinml/.links/miniconda3/envs/aidep/lib/python3.10/site-
packages/sklearn/neural_network/_multilayer_perceptron.py:690:
ConvergenceWarning: Stochastic Optimizer: Maximum iterations (1000) reached and
the optimization hasn't converged yet.
  warnings.warn(
/projects/proteinml/.links/miniconda3/envs/aidep/lib/python3.10/site-
packages/sklearn/neural_network/_multilayer_perceptron.py:690:
```

```
ConvergenceWarning: Stochastic Optimizer: Maximum iterations (1000) reached and
the optimization hasn't converged yet.
  warnings.warn(
/projects/proteinml/.links/miniconda3/envs/aidep/lib/python3.10/site-
packages/sklearn/neural_network/_multilayer_perceptron.py:690:
ConvergenceWarning: Stochastic Optimizer: Maximum iterations (1000) reached and
the optimization hasn't converged yet.
  warnings.warn(
/projects/proteinml/.links/miniconda3/envs/aidep/lib/python3.10/site-
packages/sklearn/neural_network/_multilayer_perceptron.py:690:
ConvergenceWarning: Stochastic Optimizer: Maximum iterations (1000) reached and
the optimization hasn't converged yet.
  warnings.warn(
/projects/proteinml/.links/miniconda3/envs/aidep/lib/python3.10/site-
packages/sklearn/neural_network/_multilayer_perceptron.py:690:
ConvergenceWarning: Stochastic Optimizer: Maximum iterations (1000) reached and
the optimization hasn't converged yet.
  warnings.warn(
/projects/proteinml/.links/miniconda3/envs/aidep/lib/python3.10/site-
packages/sklearn/neural_network/_multilayer_perceptron.py:690:
ConvergenceWarning: Stochastic Optimizer: Maximum iterations (1000) reached and
the optimization hasn't converged yet.
  warnings.warn(
/projects/proteinml/.links/miniconda3/envs/aidep/lib/python3.10/site-
packages/sklearn/neural_network/_multilayer_perceptron.py:690:
ConvergenceWarning: Stochastic Optimizer: Maximum iterations (1000) reached and
the optimization hasn't converged yet.
  warnings.warn(
/projects/proteinml/.links/miniconda3/envs/aidep/lib/python3.10/site-
packages/sklearn/neural_network/_multilayer_perceptron.py:690:
ConvergenceWarning: Stochastic Optimizer: Maximum iterations (1000) reached and
the optimization hasn't converged yet.
  warnings.warn(
/projects/proteinml/.links/miniconda3/envs/aidep/lib/python3.10/site-
packages/sklearn/neural_network/_multilayer_perceptron.py:690:
ConvergenceWarning: Stochastic Optimizer: Maximum iterations (1000) reached and
the optimization hasn't converged yet.
  warnings.warn(
/projects/proteinml/.links/miniconda3/envs/aidep/lib/python3.10/site-
packages/sklearn/neural_network/_multilayer_perceptron.py:690:
ConvergenceWarning: Stochastic Optimizer: Maximum iterations (1000) reached and
the optimization hasn't converged yet.
  warnings.warn(
/projects/proteinml/.links/miniconda3/envs/aidep/lib/python3.10/site-
packages/sklearn/neural_network/_multilayer_perceptron.py:690:
ConvergenceWarning: Stochastic Optimizer: Maximum iterations (1000) reached and
the optimization hasn't converged yet.
  warnings.warn(
```

```
/projects/proteinml/.links/miniconda3/envs/aidep/lib/python3.10/site-
packages/sklearn/neural_network/_multilayer_perceptron.py:690:
ConvergenceWarning: Stochastic Optimizer: Maximum iterations (1000) reached and
the optimization hasn't converged yet.
  warnings.warn(
/projects/proteinml/.links/miniconda3/envs/aidep/lib/python3.10/site-
packages/sklearn/neural_network/_multilayer_perceptron.py:690:
ConvergenceWarning: Stochastic Optimizer: Maximum iterations (1000) reached and
the optimization hasn't converged yet.
  warnings.warn(
/projects/proteinml/.links/miniconda3/envs/aidep/lib/python3.10/site-
packages/sklearn/neural_network/_multilayer_perceptron.py:690:
ConvergenceWarning: Stochastic Optimizer: Maximum iterations (1000) reached and
the optimization hasn't converged yet.
  warnings.warn(
/projects/proteinml/.links/miniconda3/envs/aidep/lib/python3.10/site-
packages/sklearn/neural_network/_multilayer_perceptron.py:690:
ConvergenceWarning: Stochastic Optimizer: Maximum iterations (1000) reached and
the optimization hasn't converged yet.
  warnings.warn(
/projects/proteinml/.links/miniconda3/envs/aidep/lib/python3.10/site-
packages/sklearn/neural_network/_multilayer_perceptron.py:690:
ConvergenceWarning: Stochastic Optimizer: Maximum iterations (1000) reached and
the optimization hasn't converged yet.
  warnings.warn(
/projects/proteinml/.links/miniconda3/envs/aidep/lib/python3.10/site-
packages/sklearn/neural_network/_multilayer_perceptron.py:690:
ConvergenceWarning: Stochastic Optimizer: Maximum iterations (1000) reached and
the optimization hasn't converged yet.
  warnings.warn(
/projects/proteinml/.links/miniconda3/envs/aidep/lib/python3.10/site-
packages/sklearn/neural_network/_multilayer_perceptron.py:690:
ConvergenceWarning: Stochastic Optimizer: Maximum iterations (1000) reached and
the optimization hasn't converged yet.
  warnings.warn(
/projects/proteinml/.links/miniconda3/envs/aidep/lib/python3.10/site-
packages/sklearn/neural_network/_multilayer_perceptron.py:690:
ConvergenceWarning: Stochastic Optimizer: Maximum iterations (1000) reached and
the optimization hasn't converged yet.
  warnings.warn(
/projects/proteinml/.links/miniconda3/envs/aidep/lib/python3.10/site-
packages/sklearn/neural_network/_multilayer_perceptron.py:690:
ConvergenceWarning: Stochastic Optimizer: Maximum iterations (1000) reached and
the optimization hasn't converged yet.
  warnings.warn(
/projects/proteinml/.links/miniconda3/envs/aidep/lib/python3.10/site-
packages/sklearn/neural_network/_multilayer_perceptron.py:690:
ConvergenceWarning: Stochastic Optimizer: Maximum iterations (1000) reached and
```

```
the optimization hasn't converged yet.
  warnings.warn(
/projects/proteinml/.links/miniconda3/envs/aidep/lib/python3.10/site-
packages/sklearn/neural_network/_multilayer_perceptron.py:690:
ConvergenceWarning: Stochastic Optimizer: Maximum iterations (1000) reached and
the optimization hasn't converged yet.
  warnings.warn(
/projects/proteinml/.links/miniconda3/envs/aidep/lib/python3.10/site-
packages/sklearn/neural_network/_multilayer_perceptron.py:690:
ConvergenceWarning: Stochastic Optimizer: Maximum iterations (1000) reached and
the optimization hasn't converged yet.
  warnings.warn(
/projects/proteinml/.links/miniconda3/envs/aidep/lib/python3.10/site-
packages/sklearn/neural_network/_multilayer_perceptron.py:690:
ConvergenceWarning: Stochastic Optimizer: Maximum iterations (1000) reached and
the optimization hasn't converged yet.
  warnings.warn(
/projects/proteinml/.links/miniconda3/envs/aidep/lib/python3.10/site-
packages/sklearn/neural_network/_multilayer_perceptron.py:690:
ConvergenceWarning: Stochastic Optimizer: Maximum iterations (1000) reached and
the optimization hasn't converged yet.
  warnings.warn(
/projects/proteinml/.links/miniconda3/envs/aidep/lib/python3.10/site-
packages/sklearn/neural_network/_multilayer_perceptron.py:690:
ConvergenceWarning: Stochastic Optimizer: Maximum iterations (1000) reached and
the optimization hasn't converged yet.
  warnings.warn(
/projects/proteinml/.links/miniconda3/envs/aidep/lib/python3.10/site-
packages/sklearn/neural_network/_multilayer_perceptron.py:690:
ConvergenceWarning: Stochastic Optimizer: Maximum iterations (1000) reached and
the optimization hasn't converged yet.
  warnings.warn(
/projects/proteinml/.links/miniconda3/envs/aidep/lib/python3.10/site-
packages/sklearn/neural_network/_multilayer_perceptron.py:690:
ConvergenceWarning: Stochastic Optimizer: Maximum iterations (1000) reached and
the optimization hasn't converged yet.
  warnings.warn(
/projects/proteinml/.links/miniconda3/envs/aidep/lib/python3.10/site-
packages/sklearn/neural_network/_multilayer_perceptron.py:690:
ConvergenceWarning: Stochastic Optimizer: Maximum iterations (1000) reached and
the optimization hasn't converged yet.
  warnings.warn(
/projects/proteinml/.links/miniconda3/envs/aidep/lib/python3.10/site-
packages/sklearn/neural_network/_multilayer_perceptron.py:690:
ConvergenceWarning: Stochastic Optimizer: Maximum iterations (1000) reached and
the optimization hasn't converged yet.
  warnings.warn(
/projects/proteinml/.links/miniconda3/envs/aidep/lib/python3.10/site-
```

```
packages/sklearn/neural_network/_multilayer_perceptron.py:690:
ConvergenceWarning: Stochastic Optimizer: Maximum iterations (1000) reached and
the optimization hasn't converged yet.
  warnings.warn(
/projects/proteinml/.links/miniconda3/envs/aidep/lib/python3.10/site-
packages/sklearn/neural_network/_multilayer_perceptron.py:690:
ConvergenceWarning: Stochastic Optimizer: Maximum iterations (1000) reached and
the optimization hasn't converged yet.
  warnings.warn(
/projects/proteinml/.links/miniconda3/envs/aidep/lib/python3.10/site-
packages/sklearn/neural_network/_multilayer_perceptron.py:690:
ConvergenceWarning: Stochastic Optimizer: Maximum iterations (1000) reached and
the optimization hasn't converged yet.
  warnings.warn(
/projects/proteinml/.links/miniconda3/envs/aidep/lib/python3.10/site-
packages/sklearn/neural_network/_multilayer_perceptron.py:690:
ConvergenceWarning: Stochastic Optimizer: Maximum iterations (1000) reached and
the optimization hasn't converged yet.
  warnings.warn(
/projects/proteinml/.links/miniconda3/envs/aidep/lib/python3.10/site-
packages/sklearn/neural_network/_multilayer_perceptron.py:690:
ConvergenceWarning: Stochastic Optimizer: Maximum iterations (1000) reached and
the optimization hasn't converged yet.
  warnings.warn(
/projects/proteinml/.links/miniconda3/envs/aidep/lib/python3.10/site-
packages/sklearn/neural_network/_multilayer_perceptron.py:690:
ConvergenceWarning: Stochastic Optimizer: Maximum iterations (1000) reached and
the optimization hasn't converged yet.
  warnings.warn(
/projects/proteinml/.links/miniconda3/envs/aidep/lib/python3.10/site-
packages/sklearn/neural_network/_multilayer_perceptron.py:690:
ConvergenceWarning: Stochastic Optimizer: Maximum iterations (1000) reached and
the optimization hasn't converged yet.
  warnings.warn(
/projects/proteinml/.links/miniconda3/envs/aidep/lib/python3.10/site-
packages/sklearn/neural_network/_multilayer_perceptron.py:690:
ConvergenceWarning: Stochastic Optimizer: Maximum iterations (1000) reached and
the optimization hasn't converged yet.
  warnings.warn(
/projects/proteinml/.links/miniconda3/envs/aidep/lib/python3.10/site-
packages/sklearn/neural_network/_multilayer_perceptron.py:690:
ConvergenceWarning: Stochastic Optimizer: Maximum iterations (1000) reached and
the optimization hasn't converged yet.
  warnings.warn(
/projects/proteinml/.links/miniconda3/envs/aidep/lib/python3.10/site-
packages/sklearn/neural_network/_multilayer_perceptron.py:690:
ConvergenceWarning: Stochastic Optimizer: Maximum iterations (1000) reached and
the optimization hasn't converged yet.
```

```
  warnings.warn(
/projects/proteinml/.links/miniconda3/envs/aidep/lib/python3.10/site-
packages/sklearn/neural_network/_multilayer_perceptron.py:690:
ConvergenceWarning: Stochastic Optimizer: Maximum iterations (1000) reached and
the optimization hasn't converged yet.
  warnings.warn(
/projects/proteinml/.links/miniconda3/envs/aidep/lib/python3.10/site-
packages/sklearn/neural_network/_multilayer_perceptron.py:690:
ConvergenceWarning: Stochastic Optimizer: Maximum iterations (1000) reached and
the optimization hasn't converged yet.
  warnings.warn(
/projects/proteinml/.links/miniconda3/envs/aidep/lib/python3.10/site-
packages/sklearn/neural_network/_multilayer_perceptron.py:690:
ConvergenceWarning: Stochastic Optimizer: Maximum iterations (1000) reached and
the optimization hasn't converged yet.
  warnings.warn(
/projects/proteinml/.links/miniconda3/envs/aidep/lib/python3.10/site-
packages/sklearn/neural_network/_multilayer_perceptron.py:690:
ConvergenceWarning: Stochastic Optimizer: Maximum iterations (1000) reached and
the optimization hasn't converged yet.
  warnings.warn(
/projects/proteinml/.links/miniconda3/envs/aidep/lib/python3.10/site-
packages/sklearn/neural_network/_multilayer_perceptron.py:690:
ConvergenceWarning: Stochastic Optimizer: Maximum iterations (1000) reached and
the optimization hasn't converged yet.
  warnings.warn(
/projects/proteinml/.links/miniconda3/envs/aidep/lib/python3.10/site-
packages/sklearn/neural_network/_multilayer_perceptron.py:690:
ConvergenceWarning: Stochastic Optimizer: Maximum iterations (1000) reached and
the optimization hasn't converged yet.
  warnings.warn(
/projects/proteinml/.links/miniconda3/envs/aidep/lib/python3.10/site-
packages/sklearn/neural_network/_multilayer_perceptron.py:690:
ConvergenceWarning: Stochastic Optimizer: Maximum iterations (1000) reached and
the optimization hasn't converged yet.
  warnings.warn(
/projects/proteinml/.links/miniconda3/envs/aidep/lib/python3.10/site-
packages/sklearn/neural_network/_multilayer_perceptron.py:690:
ConvergenceWarning: Stochastic Optimizer: Maximum iterations (1000) reached and
the optimization hasn't converged yet.
  warnings.warn(
/projects/proteinml/.links/miniconda3/envs/aidep/lib/python3.10/site-
packages/sklearn/neural_network/_multilayer_perceptron.py:690:
ConvergenceWarning: Stochastic Optimizer: Maximum iterations (1000) reached and
the optimization hasn't converged yet.
  warnings.warn(
/projects/proteinml/.links/miniconda3/envs/aidep/lib/python3.10/site-
packages/sklearn/neural_network/_multilayer_perceptron.py:690:
```

```
ConvergenceWarning: Stochastic Optimizer: Maximum iterations (1000) reached and
the optimization hasn't converged yet.
  warnings.warn(
/projects/proteinml/.links/miniconda3/envs/aidep/lib/python3.10/site-
packages/sklearn/neural_network/_multilayer_perceptron.py:690:
ConvergenceWarning: Stochastic Optimizer: Maximum iterations (1000) reached and
the optimization hasn't converged yet.
  warnings.warn(
/projects/proteinml/.links/miniconda3/envs/aidep/lib/python3.10/site-
packages/sklearn/neural_network/_multilayer_perceptron.py:690:
ConvergenceWarning: Stochastic Optimizer: Maximum iterations (1000) reached and
the optimization hasn't converged yet.
  warnings.warn(
/projects/proteinml/.links/miniconda3/envs/aidep/lib/python3.10/site-
packages/sklearn/neural_network/_multilayer_perceptron.py:690:
ConvergenceWarning: Stochastic Optimizer: Maximum iterations (1000) reached and
the optimization hasn't converged yet.
  warnings.warn(
/projects/proteinml/.links/miniconda3/envs/aidep/lib/python3.10/site-
packages/sklearn/neural_network/_multilayer_perceptron.py:690:
ConvergenceWarning: Stochastic Optimizer: Maximum iterations (1000) reached and
the optimization hasn't converged yet.
  warnings.warn(
/projects/proteinml/.links/miniconda3/envs/aidep/lib/python3.10/site-
packages/sklearn/neural_network/_multilayer_perceptron.py:690:
ConvergenceWarning: Stochastic Optimizer: Maximum iterations (1000) reached and
the optimization hasn't converged yet.
  warnings.warn(
/projects/proteinml/.links/miniconda3/envs/aidep/lib/python3.10/site-
packages/sklearn/neural_network/_multilayer_perceptron.py:690:
ConvergenceWarning: Stochastic Optimizer: Maximum iterations (1000) reached and
the optimization hasn't converged yet.
  warnings.warn(
/projects/proteinml/.links/miniconda3/envs/aidep/lib/python3.10/site-
packages/sklearn/neural_network/_multilayer_perceptron.py:690:
ConvergenceWarning: Stochastic Optimizer: Maximum iterations (1000) reached and
the optimization hasn't converged yet.
  warnings.warn(
/projects/proteinml/.links/miniconda3/envs/aidep/lib/python3.10/site-
packages/sklearn/neural_network/_multilayer_perceptron.py:690:
ConvergenceWarning: Stochastic Optimizer: Maximum iterations (1000) reached and
the optimization hasn't converged yet.
  warnings.warn(
/projects/proteinml/.links/miniconda3/envs/aidep/lib/python3.10/site-
packages/sklearn/neural_network/_multilayer_perceptron.py:690:
ConvergenceWarning: Stochastic Optimizer: Maximum iterations (1000) reached and
the optimization hasn't converged yet.
  warnings.warn(
```

```
/projects/proteinml/.links/miniconda3/envs/aidep/lib/python3.10/site-
packages/sklearn/neural_network/_multilayer_perceptron.py:690:
ConvergenceWarning: Stochastic Optimizer: Maximum iterations (1000) reached and
the optimization hasn't converged yet.
  warnings.warn(
/projects/proteinml/.links/miniconda3/envs/aidep/lib/python3.10/site-
packages/sklearn/neural_network/_multilayer_perceptron.py:690:
ConvergenceWarning: Stochastic Optimizer: Maximum iterations (1000) reached and
the optimization hasn't converged yet.
  warnings.warn(
/projects/proteinml/.links/miniconda3/envs/aidep/lib/python3.10/site-
packages/sklearn/neural_network/_multilayer_perceptron.py:690:
ConvergenceWarning: Stochastic Optimizer: Maximum iterations (1000) reached and
the optimization hasn't converged yet.
  warnings.warn(
/projects/proteinml/.links/miniconda3/envs/aidep/lib/python3.10/site-
packages/sklearn/neural_network/_multilayer_perceptron.py:690:
ConvergenceWarning: Stochastic Optimizer: Maximum iterations (1000) reached and
the optimization hasn't converged yet.
  warnings.warn(
/projects/proteinml/.links/miniconda3/envs/aidep/lib/python3.10/site-
packages/sklearn/neural_network/_multilayer_perceptron.py:690:
ConvergenceWarning: Stochastic Optimizer: Maximum iterations (1000) reached and
the optimization hasn't converged yet.
  warnings.warn(
/projects/proteinml/.links/miniconda3/envs/aidep/lib/python3.10/site-
packages/sklearn/neural_network/_multilayer_perceptron.py:690:
ConvergenceWarning: Stochastic Optimizer: Maximum iterations (1000) reached and
the optimization hasn't converged yet.
  warnings.warn(
/projects/proteinml/.links/miniconda3/envs/aidep/lib/python3.10/site-
packages/sklearn/neural_network/_multilayer_perceptron.py:690:
ConvergenceWarning: Stochastic Optimizer: Maximum iterations (1000) reached and
the optimization hasn't converged yet.
  warnings.warn(
/projects/proteinml/.links/miniconda3/envs/aidep/lib/python3.10/site-
packages/sklearn/neural_network/_multilayer_perceptron.py:690:
ConvergenceWarning: Stochastic Optimizer: Maximum iterations (1000) reached and
the optimization hasn't converged yet.
  warnings.warn(
/projects/proteinml/.links/miniconda3/envs/aidep/lib/python3.10/site-
packages/sklearn/neural_network/_multilayer_perceptron.py:690:
ConvergenceWarning: Stochastic Optimizer: Maximum iterations (1000) reached and
the optimization hasn't converged yet.
  warnings.warn(
/projects/proteinml/.links/miniconda3/envs/aidep/lib/python3.10/site-
packages/sklearn/neural_network/_multilayer_perceptron.py:690:
ConvergenceWarning: Stochastic Optimizer: Maximum iterations (1000) reached and
```

```
the optimization hasn't converged yet.
  warnings.warn(
/projects/proteinml/.links/miniconda3/envs/aidep/lib/python3.10/site-
packages/sklearn/neural_network/_multilayer_perceptron.py:690:
ConvergenceWarning: Stochastic Optimizer: Maximum iterations (1000) reached and
the optimization hasn't converged yet.
  warnings.warn(
/projects/proteinml/.links/miniconda3/envs/aidep/lib/python3.10/site-
packages/sklearn/neural_network/_multilayer_perceptron.py:690:
ConvergenceWarning: Stochastic Optimizer: Maximum iterations (1000) reached and
the optimization hasn't converged yet.
  warnings.warn(
/projects/proteinml/.links/miniconda3/envs/aidep/lib/python3.10/site-
packages/sklearn/neural_network/_multilayer_perceptron.py:690:
ConvergenceWarning: Stochastic Optimizer: Maximum iterations (1000) reached and
the optimization hasn't converged yet.
  warnings.warn(
/projects/proteinml/.links/miniconda3/envs/aidep/lib/python3.10/site-
packages/sklearn/neural_network/_multilayer_perceptron.py:690:
ConvergenceWarning: Stochastic Optimizer: Maximum iterations (1000) reached and
the optimization hasn't converged yet.
  warnings.warn(
/projects/proteinml/.links/miniconda3/envs/aidep/lib/python3.10/site-
packages/sklearn/neural_network/_multilayer_perceptron.py:690:
ConvergenceWarning: Stochastic Optimizer: Maximum iterations (1000) reached and
the optimization hasn't converged yet.
  warnings.warn(
/projects/proteinml/.links/miniconda3/envs/aidep/lib/python3.10/site-
packages/sklearn/neural_network/_multilayer_perceptron.py:690:
ConvergenceWarning: Stochastic Optimizer: Maximum iterations (1000) reached and
the optimization hasn't converged yet.
  warnings.warn(
/projects/proteinml/.links/miniconda3/envs/aidep/lib/python3.10/site-
packages/sklearn/neural_network/_multilayer_perceptron.py:690:
ConvergenceWarning: Stochastic Optimizer: Maximum iterations (1000) reached and
the optimization hasn't converged yet.
  warnings.warn(
/projects/proteinml/.links/miniconda3/envs/aidep/lib/python3.10/site-
packages/sklearn/neural_network/_multilayer_perceptron.py:690:
ConvergenceWarning: Stochastic Optimizer: Maximum iterations (1000) reached and
the optimization hasn't converged yet.
  warnings.warn(
/projects/proteinml/.links/miniconda3/envs/aidep/lib/python3.10/site-
packages/sklearn/neural_network/_multilayer_perceptron.py:690:
ConvergenceWarning: Stochastic Optimizer: Maximum iterations (1000) reached and
the optimization hasn't converged yet.
  warnings.warn(
/projects/proteinml/.links/miniconda3/envs/aidep/lib/python3.10/site-
```

```
packages/sklearn/neural_network/_multilayer_perceptron.py:690:
ConvergenceWarning: Stochastic Optimizer: Maximum iterations (1000) reached and
the optimization hasn't converged yet.
  warnings.warn(
/projects/proteinml/.links/miniconda3/envs/aidep/lib/python3.10/site-
packages/sklearn/neural_network/_multilayer_perceptron.py:690:
ConvergenceWarning: Stochastic Optimizer: Maximum iterations (1000) reached and
the optimization hasn't converged yet.
  warnings.warn(
/projects/proteinml/.links/miniconda3/envs/aidep/lib/python3.10/site-
packages/sklearn/neural_network/_multilayer_perceptron.py:690:
ConvergenceWarning: Stochastic Optimizer: Maximum iterations (1000) reached and
the optimization hasn't converged yet.
  warnings.warn(
/projects/proteinml/.links/miniconda3/envs/aidep/lib/python3.10/site-
packages/sklearn/neural_network/_multilayer_perceptron.py:690:
ConvergenceWarning: Stochastic Optimizer: Maximum iterations (1000) reached and
the optimization hasn't converged yet.
  warnings.warn(
/projects/proteinml/.links/miniconda3/envs/aidep/lib/python3.10/site-
packages/sklearn/neural_network/_multilayer_perceptron.py:690:
ConvergenceWarning: Stochastic Optimizer: Maximum iterations (1000) reached and
the optimization hasn't converged yet.
  warnings.warn(
/projects/proteinml/.links/miniconda3/envs/aidep/lib/python3.10/site-
packages/sklearn/neural_network/_multilayer_perceptron.py:690:
ConvergenceWarning: Stochastic Optimizer: Maximum iterations (1000) reached and
the optimization hasn't converged yet.
  warnings.warn(
/projects/proteinml/.links/miniconda3/envs/aidep/lib/python3.10/site-
packages/sklearn/neural_network/_multilayer_perceptron.py:690:
ConvergenceWarning: Stochastic Optimizer: Maximum iterations (1000) reached and
the optimization hasn't converged yet.
  warnings.warn(
/projects/proteinml/.links/miniconda3/envs/aidep/lib/python3.10/site-
packages/sklearn/neural_network/_multilayer_perceptron.py:690:
ConvergenceWarning: Stochastic Optimizer: Maximum iterations (1000) reached and
the optimization hasn't converged yet.
  warnings.warn(
/projects/proteinml/.links/miniconda3/envs/aidep/lib/python3.10/site-
packages/sklearn/neural_network/_multilayer_perceptron.py:690:
ConvergenceWarning: Stochastic Optimizer: Maximum iterations (1000) reached and
the optimization hasn't converged yet.
  warnings.warn(
/projects/proteinml/.links/miniconda3/envs/aidep/lib/python3.10/site-
packages/sklearn/neural_network/_multilayer_perceptron.py:690:
ConvergenceWarning: Stochastic Optimizer: Maximum iterations (1000) reached and
the optimization hasn't converged yet.
```

```
  warnings.warn(
/projects/proteinml/.links/miniconda3/envs/aidep/lib/python3.10/site-
packages/sklearn/neural_network/_multilayer_perceptron.py:690:
ConvergenceWarning: Stochastic Optimizer: Maximum iterations (1000) reached and
the optimization hasn't converged yet.
  warnings.warn(
/projects/proteinml/.links/miniconda3/envs/aidep/lib/python3.10/site-
packages/sklearn/neural_network/_multilayer_perceptron.py:690:
ConvergenceWarning: Stochastic Optimizer: Maximum iterations (1000) reached and
the optimization hasn't converged yet.
  warnings.warn(
/projects/proteinml/.links/miniconda3/envs/aidep/lib/python3.10/site-
packages/sklearn/neural_network/_multilayer_perceptron.py:690:
ConvergenceWarning: Stochastic Optimizer: Maximum iterations (1000) reached and
the optimization hasn't converged yet.
  warnings.warn(
/projects/proteinml/.links/miniconda3/envs/aidep/lib/python3.10/site-
packages/sklearn/neural_network/_multilayer_perceptron.py:690:
ConvergenceWarning: Stochastic Optimizer: Maximum iterations (1000) reached and
the optimization hasn't converged yet.
  warnings.warn(
/projects/proteinml/.links/miniconda3/envs/aidep/lib/python3.10/site-
packages/sklearn/neural_network/_multilayer_perceptron.py:690:
ConvergenceWarning: Stochastic Optimizer: Maximum iterations (1000) reached and
the optimization hasn't converged yet.
  warnings.warn(
/projects/proteinml/.links/miniconda3/envs/aidep/lib/python3.10/site-
packages/sklearn/neural_network/_multilayer_perceptron.py:690:
ConvergenceWarning: Stochastic Optimizer: Maximum iterations (1000) reached and
the optimization hasn't converged yet.
  warnings.warn(
/projects/proteinml/.links/miniconda3/envs/aidep/lib/python3.10/site-
packages/sklearn/neural_network/_multilayer_perceptron.py:690:
ConvergenceWarning: Stochastic Optimizer: Maximum iterations (1000) reached and
the optimization hasn't converged yet.
  warnings.warn(
/projects/proteinml/.links/miniconda3/envs/aidep/lib/python3.10/site-
packages/sklearn/neural_network/_multilayer_perceptron.py:690:
ConvergenceWarning: Stochastic Optimizer: Maximum iterations (1000) reached and
the optimization hasn't converged yet.
  warnings.warn(
/projects/proteinml/.links/miniconda3/envs/aidep/lib/python3.10/site-
packages/sklearn/neural_network/_multilayer_perceptron.py:690:
ConvergenceWarning: Stochastic Optimizer: Maximum iterations (1000) reached and
the optimization hasn't converged yet.
  warnings.warn(
/projects/proteinml/.links/miniconda3/envs/aidep/lib/python3.10/site-
packages/sklearn/neural_network/_multilayer_perceptron.py:690:
```

```
ConvergenceWarning: Stochastic Optimizer: Maximum iterations (1000) reached and
the optimization hasn't converged yet.
  warnings.warn(
/projects/proteinml/.links/miniconda3/envs/aidep/lib/python3.10/site-
packages/sklearn/neural_network/_multilayer_perceptron.py:690:
ConvergenceWarning: Stochastic Optimizer: Maximum iterations (1000) reached and
the optimization hasn't converged yet.
  warnings.warn(
/projects/proteinml/.links/miniconda3/envs/aidep/lib/python3.10/site-
packages/sklearn/neural_network/_multilayer_perceptron.py:690:
ConvergenceWarning: Stochastic Optimizer: Maximum iterations (1000) reached and
the optimization hasn't converged yet.
  warnings.warn(
/projects/proteinml/.links/miniconda3/envs/aidep/lib/python3.10/site-
packages/sklearn/neural_network/_multilayer_perceptron.py:690:
ConvergenceWarning: Stochastic Optimizer: Maximum iterations (1000) reached and
the optimization hasn't converged yet.
  warnings.warn(
/projects/proteinml/.links/miniconda3/envs/aidep/lib/python3.10/site-
packages/sklearn/neural_network/_multilayer_perceptron.py:690:
ConvergenceWarning: Stochastic Optimizer: Maximum iterations (1000) reached and
the optimization hasn't converged yet.
  warnings.warn(
/projects/proteinml/.links/miniconda3/envs/aidep/lib/python3.10/site-
packages/sklearn/neural_network/_multilayer_perceptron.py:690:
ConvergenceWarning: Stochastic Optimizer: Maximum iterations (1000) reached and
the optimization hasn't converged yet.
  warnings.warn(
/projects/proteinml/.links/miniconda3/envs/aidep/lib/python3.10/site-
packages/sklearn/neural_network/_multilayer_perceptron.py:690:
ConvergenceWarning: Stochastic Optimizer: Maximum iterations (1000) reached and
the optimization hasn't converged yet.
  warnings.warn(
/projects/proteinml/.links/miniconda3/envs/aidep/lib/python3.10/site-
packages/sklearn/neural_network/_multilayer_perceptron.py:690:
ConvergenceWarning: Stochastic Optimizer: Maximum iterations (1000) reached and
the optimization hasn't converged yet.
  warnings.warn(
/projects/proteinml/.links/miniconda3/envs/aidep/lib/python3.10/site-
packages/sklearn/neural_network/_multilayer_perceptron.py:690:
ConvergenceWarning: Stochastic Optimizer: Maximum iterations (1000) reached and
the optimization hasn't converged yet.
  warnings.warn(
/projects/proteinml/.links/miniconda3/envs/aidep/lib/python3.10/site-
packages/sklearn/neural_network/_multilayer_perceptron.py:690:
ConvergenceWarning: Stochastic Optimizer: Maximum iterations (1000) reached and
the optimization hasn't converged yet.
  warnings.warn(
```

```
/projects/proteinml/.links/miniconda3/envs/aidep/lib/python3.10/site-
packages/sklearn/neural_network/_multilayer_perceptron.py:690:
ConvergenceWarning: Stochastic Optimizer: Maximum iterations (1000) reached and
the optimization hasn't converged yet.
  warnings.warn(
/projects/proteinml/.links/miniconda3/envs/aidep/lib/python3.10/site-
packages/sklearn/neural_network/_multilayer_perceptron.py:690:
ConvergenceWarning: Stochastic Optimizer: Maximum iterations (1000) reached and
the optimization hasn't converged yet.
  warnings.warn(
/projects/proteinml/.links/miniconda3/envs/aidep/lib/python3.10/site-
packages/sklearn/neural_network/_multilayer_perceptron.py:690:
ConvergenceWarning: Stochastic Optimizer: Maximum iterations (1000) reached and
the optimization hasn't converged yet.
  warnings.warn(
/projects/proteinml/.links/miniconda3/envs/aidep/lib/python3.10/site-
packages/sklearn/neural_network/_multilayer_perceptron.py:690:
ConvergenceWarning: Stochastic Optimizer: Maximum iterations (1000) reached and
the optimization hasn't converged yet.
  warnings.warn(
/projects/proteinml/.links/miniconda3/envs/aidep/lib/python3.10/site-
packages/sklearn/neural_network/_multilayer_perceptron.py:690:
ConvergenceWarning: Stochastic Optimizer: Maximum iterations (1000) reached and
the optimization hasn't converged yet.
  warnings.warn(
/projects/proteinml/.links/miniconda3/envs/aidep/lib/python3.10/site-
packages/sklearn/neural_network/_multilayer_perceptron.py:690:
ConvergenceWarning: Stochastic Optimizer: Maximum iterations (1000) reached and
the optimization hasn't converged yet.
  warnings.warn(
/projects/proteinml/.links/miniconda3/envs/aidep/lib/python3.10/site-
packages/sklearn/neural_network/_multilayer_perceptron.py:690:
ConvergenceWarning: Stochastic Optimizer: Maximum iterations (1000) reached and
the optimization hasn't converged yet.
  warnings.warn(
/projects/proteinml/.links/miniconda3/envs/aidep/lib/python3.10/site-
packages/sklearn/neural_network/_multilayer_perceptron.py:690:
ConvergenceWarning: Stochastic Optimizer: Maximum iterations (1000) reached and
the optimization hasn't converged yet.
  warnings.warn(
/projects/proteinml/.links/miniconda3/envs/aidep/lib/python3.10/site-
packages/sklearn/neural_network/_multilayer_perceptron.py:690:
ConvergenceWarning: Stochastic Optimizer: Maximum iterations (1000) reached and
the optimization hasn't converged yet.
  warnings.warn(
/projects/proteinml/.links/miniconda3/envs/aidep/lib/python3.10/site-
packages/sklearn/neural_network/_multilayer_perceptron.py:690:
ConvergenceWarning: Stochastic Optimizer: Maximum iterations (1000) reached and
```

```
the optimization hasn't converged yet.
  warnings.warn(
/projects/proteinml/.links/miniconda3/envs/aidep/lib/python3.10/site-
packages/sklearn/neural_network/_multilayer_perceptron.py:690:
ConvergenceWarning: Stochastic Optimizer: Maximum iterations (1000) reached and
the optimization hasn't converged yet.
  warnings.warn(
/projects/proteinml/.links/miniconda3/envs/aidep/lib/python3.10/site-
packages/sklearn/neural_network/_multilayer_perceptron.py:690:
ConvergenceWarning: Stochastic Optimizer: Maximum iterations (1000) reached and
the optimization hasn't converged yet.
  warnings.warn(
/projects/proteinml/.links/miniconda3/envs/aidep/lib/python3.10/site-
packages/sklearn/neural_network/_multilayer_perceptron.py:690:
ConvergenceWarning: Stochastic Optimizer: Maximum iterations (1000) reached and
the optimization hasn't converged yet.
  warnings.warn(
/projects/proteinml/.links/miniconda3/envs/aidep/lib/python3.10/site-
packages/sklearn/neural_network/_multilayer_perceptron.py:690:
ConvergenceWarning: Stochastic Optimizer: Maximum iterations (1000) reached and
the optimization hasn't converged yet.
  warnings.warn(
/projects/proteinml/.links/miniconda3/envs/aidep/lib/python3.10/site-
packages/sklearn/neural_network/_multilayer_perceptron.py:690:
ConvergenceWarning: Stochastic Optimizer: Maximum iterations (1000) reached and
the optimization hasn't converged yet.
  warnings.warn(
/projects/proteinml/.links/miniconda3/envs/aidep/lib/python3.10/site-
packages/sklearn/neural_network/_multilayer_perceptron.py:690:
ConvergenceWarning: Stochastic Optimizer: Maximum iterations (1000) reached and
the optimization hasn't converged yet.
  warnings.warn(
/projects/proteinml/.links/miniconda3/envs/aidep/lib/python3.10/site-
packages/sklearn/neural_network/_multilayer_perceptron.py:690:
ConvergenceWarning: Stochastic Optimizer: Maximum iterations (1000) reached and
the optimization hasn't converged yet.
  warnings.warn(
/projects/proteinml/.links/miniconda3/envs/aidep/lib/python3.10/site-
packages/sklearn/neural_network/_multilayer_perceptron.py:690:
ConvergenceWarning: Stochastic Optimizer: Maximum iterations (1000) reached and
the optimization hasn't converged yet.
  warnings.warn(
/projects/proteinml/.links/miniconda3/envs/aidep/lib/python3.10/site-
packages/sklearn/neural_network/_multilayer_perceptron.py:690:
ConvergenceWarning: Stochastic Optimizer: Maximum iterations (1000) reached and
the optimization hasn't converged yet.
  warnings.warn(
/projects/proteinml/.links/miniconda3/envs/aidep/lib/python3.10/site-
```

```
packages/sklearn/neural_network/_multilayer_perceptron.py:690:
ConvergenceWarning: Stochastic Optimizer: Maximum iterations (1000) reached and
the optimization hasn't converged yet.
  warnings.warn(
/projects/proteinml/.links/miniconda3/envs/aidep/lib/python3.10/site-
packages/sklearn/neural_network/_multilayer_perceptron.py:690:
ConvergenceWarning: Stochastic Optimizer: Maximum iterations (1000) reached and
the optimization hasn't converged yet.
  warnings.warn(
/projects/proteinml/.links/miniconda3/envs/aidep/lib/python3.10/site-
packages/sklearn/neural_network/_multilayer_perceptron.py:690:
ConvergenceWarning: Stochastic Optimizer: Maximum iterations (1000) reached and
the optimization hasn't converged yet.
  warnings.warn(
/projects/proteinml/.links/miniconda3/envs/aidep/lib/python3.10/site-
packages/sklearn/neural_network/_multilayer_perceptron.py:690:
ConvergenceWarning: Stochastic Optimizer: Maximum iterations (1000) reached and
the optimization hasn't converged yet.
  warnings.warn(
/projects/proteinml/.links/miniconda3/envs/aidep/lib/python3.10/site-
packages/sklearn/neural_network/_multilayer_perceptron.py:690:
ConvergenceWarning: Stochastic Optimizer: Maximum iterations (1000) reached and
the optimization hasn't converged yet.
  warnings.warn(
/projects/proteinml/.links/miniconda3/envs/aidep/lib/python3.10/site-
packages/sklearn/neural_network/_multilayer_perceptron.py:690:
ConvergenceWarning: Stochastic Optimizer: Maximum iterations (1000) reached and
the optimization hasn't converged yet.
  warnings.warn(
/projects/proteinml/.links/miniconda3/envs/aidep/lib/python3.10/site-
packages/sklearn/neural_network/_multilayer_perceptron.py:690:
ConvergenceWarning: Stochastic Optimizer: Maximum iterations (1000) reached and
the optimization hasn't converged yet.
  warnings.warn(
/projects/proteinml/.links/miniconda3/envs/aidep/lib/python3.10/site-
packages/sklearn/neural_network/_multilayer_perceptron.py:690:
ConvergenceWarning: Stochastic Optimizer: Maximum iterations (1000) reached and
the optimization hasn't converged yet.
  warnings.warn(
/projects/proteinml/.links/miniconda3/envs/aidep/lib/python3.10/site-
packages/sklearn/neural_network/_multilayer_perceptron.py:690:
ConvergenceWarning: Stochastic Optimizer: Maximum iterations (1000) reached and
the optimization hasn't converged yet.
  warnings.warn(
/projects/proteinml/.links/miniconda3/envs/aidep/lib/python3.10/site-
packages/sklearn/neural_network/_multilayer_perceptron.py:690:
ConvergenceWarning: Stochastic Optimizer: Maximum iterations (1000) reached and
the optimization hasn't converged yet.
```

```
  warnings.warn(
/projects/proteinml/.links/miniconda3/envs/aidep/lib/python3.10/site-
packages/sklearn/neural_network/_multilayer_perceptron.py:690:
ConvergenceWarning: Stochastic Optimizer: Maximum iterations (1000) reached and
the optimization hasn't converged yet.
  warnings.warn(
/projects/proteinml/.links/miniconda3/envs/aidep/lib/python3.10/site-
packages/sklearn/neural_network/_multilayer_perceptron.py:690:
ConvergenceWarning: Stochastic Optimizer: Maximum iterations (1000) reached and
the optimization hasn't converged yet.
  warnings.warn(
/projects/proteinml/.links/miniconda3/envs/aidep/lib/python3.10/site-
packages/sklearn/neural_network/_multilayer_perceptron.py:690:
ConvergenceWarning: Stochastic Optimizer: Maximum iterations (1000) reached and
the optimization hasn't converged yet.
  warnings.warn(
/projects/proteinml/.links/miniconda3/envs/aidep/lib/python3.10/site-
packages/sklearn/neural_network/_multilayer_perceptron.py:690:
ConvergenceWarning: Stochastic Optimizer: Maximum iterations (1000) reached and
the optimization hasn't converged yet.
  warnings.warn(
/projects/proteinml/.links/miniconda3/envs/aidep/lib/python3.10/site-
packages/sklearn/neural_network/_multilayer_perceptron.py:690:
ConvergenceWarning: Stochastic Optimizer: Maximum iterations (1000) reached and
the optimization hasn't converged yet.
  warnings.warn(
/projects/proteinml/.links/miniconda3/envs/aidep/lib/python3.10/site-
packages/sklearn/neural_network/_multilayer_perceptron.py:690:
ConvergenceWarning: Stochastic Optimizer: Maximum iterations (1000) reached and
the optimization hasn't converged yet.
  warnings.warn(
/projects/proteinml/.links/miniconda3/envs/aidep/lib/python3.10/site-
packages/sklearn/neural_network/_multilayer_perceptron.py:690:
ConvergenceWarning: Stochastic Optimizer: Maximum iterations (1000) reached and
the optimization hasn't converged yet.
  warnings.warn(
/projects/proteinml/.links/miniconda3/envs/aidep/lib/python3.10/site-
packages/sklearn/neural_network/_multilayer_perceptron.py:690:
ConvergenceWarning: Stochastic Optimizer: Maximum iterations (1000) reached and
the optimization hasn't converged yet.
  warnings.warn(
/projects/proteinml/.links/miniconda3/envs/aidep/lib/python3.10/site-
packages/sklearn/neural_network/_multilayer_perceptron.py:690:
ConvergenceWarning: Stochastic Optimizer: Maximum iterations (1000) reached and
the optimization hasn't converged yet.
  warnings.warn(
/projects/proteinml/.links/miniconda3/envs/aidep/lib/python3.10/site-
packages/sklearn/neural_network/_multilayer_perceptron.py:690:
```

```
ConvergenceWarning: Stochastic Optimizer: Maximum iterations (1000) reached and
the optimization hasn't converged yet.
  warnings.warn(
/projects/proteinml/.links/miniconda3/envs/aidep/lib/python3.10/site-
packages/sklearn/neural_network/_multilayer_perceptron.py:690:
ConvergenceWarning: Stochastic Optimizer: Maximum iterations (1000) reached and
the optimization hasn't converged yet.
  warnings.warn(
/projects/proteinml/.links/miniconda3/envs/aidep/lib/python3.10/site-
packages/sklearn/neural_network/_multilayer_perceptron.py:690:
ConvergenceWarning: Stochastic Optimizer: Maximum iterations (1000) reached and
the optimization hasn't converged yet.
  warnings.warn(
/projects/proteinml/.links/miniconda3/envs/aidep/lib/python3.10/site-
packages/sklearn/neural_network/_multilayer_perceptron.py:690:
ConvergenceWarning: Stochastic Optimizer: Maximum iterations (1000) reached and
the optimization hasn't converged yet.
  warnings.warn(
/projects/proteinml/.links/miniconda3/envs/aidep/lib/python3.10/site-
packages/sklearn/neural_network/_multilayer_perceptron.py:690:
ConvergenceWarning: Stochastic Optimizer: Maximum iterations (1000) reached and
the optimization hasn't converged yet.
  warnings.warn(
/projects/proteinml/.links/miniconda3/envs/aidep/lib/python3.10/site-
packages/sklearn/neural_network/_multilayer_perceptron.py:690:
ConvergenceWarning: Stochastic Optimizer: Maximum iterations (1000) reached and
the optimization hasn't converged yet.
  warnings.warn(
/projects/proteinml/.links/miniconda3/envs/aidep/lib/python3.10/site-
packages/sklearn/neural_network/_multilayer_perceptron.py:690:
ConvergenceWarning: Stochastic Optimizer: Maximum iterations (1000) reached and
the optimization hasn't converged yet.
  warnings.warn(
/projects/proteinml/.links/miniconda3/envs/aidep/lib/python3.10/site-
packages/sklearn/neural_network/_multilayer_perceptron.py:690:
ConvergenceWarning: Stochastic Optimizer: Maximum iterations (1000) reached and
the optimization hasn't converged yet.
  warnings.warn(
/projects/proteinml/.links/miniconda3/envs/aidep/lib/python3.10/site-
packages/sklearn/neural_network/_multilayer_perceptron.py:690:
ConvergenceWarning: Stochastic Optimizer: Maximum iterations (1000) reached and
the optimization hasn't converged yet.
  warnings.warn(
/projects/proteinml/.links/miniconda3/envs/aidep/lib/python3.10/site-
packages/sklearn/neural_network/_multilayer_perceptron.py:690:
ConvergenceWarning: Stochastic Optimizer: Maximum iterations (1000) reached and
the optimization hasn't converged yet.
  warnings.warn(
```

```
/projects/proteinml/.links/miniconda3/envs/aidep/lib/python3.10/site-
packages/sklearn/neural_network/_multilayer_perceptron.py:690:
ConvergenceWarning: Stochastic Optimizer: Maximum iterations (1000) reached and
the optimization hasn't converged yet.
  warnings.warn(
/projects/proteinml/.links/miniconda3/envs/aidep/lib/python3.10/site-
packages/sklearn/neural_network/_multilayer_perceptron.py:690:
ConvergenceWarning: Stochastic Optimizer: Maximum iterations (1000) reached and
the optimization hasn't converged yet.
  warnings.warn(
/projects/proteinml/.links/miniconda3/envs/aidep/lib/python3.10/site-
packages/sklearn/neural_network/_multilayer_perceptron.py:690:
ConvergenceWarning: Stochastic Optimizer: Maximum iterations (1000) reached and
the optimization hasn't converged yet.
  warnings.warn(
/projects/proteinml/.links/miniconda3/envs/aidep/lib/python3.10/site-
packages/sklearn/neural_network/_multilayer_perceptron.py:690:
ConvergenceWarning: Stochastic Optimizer: Maximum iterations (1000) reached and
the optimization hasn't converged yet.
  warnings.warn(
/projects/proteinml/.links/miniconda3/envs/aidep/lib/python3.10/site-
packages/sklearn/neural_network/_multilayer_perceptron.py:690:
ConvergenceWarning: Stochastic Optimizer: Maximum iterations (1000) reached and
the optimization hasn't converged yet.
  warnings.warn(
/projects/proteinml/.links/miniconda3/envs/aidep/lib/python3.10/site-
packages/sklearn/neural_network/_multilayer_perceptron.py:690:
ConvergenceWarning: Stochastic Optimizer: Maximum iterations (1000) reached and
the optimization hasn't converged yet.
  warnings.warn(
/projects/proteinml/.links/miniconda3/envs/aidep/lib/python3.10/site-
packages/sklearn/neural_network/_multilayer_perceptron.py:690:
ConvergenceWarning: Stochastic Optimizer: Maximum iterations (1000) reached and
the optimization hasn't converged yet.
  warnings.warn(
/projects/proteinml/.links/miniconda3/envs/aidep/lib/python3.10/site-
packages/sklearn/neural_network/_multilayer_perceptron.py:690:
ConvergenceWarning: Stochastic Optimizer: Maximum iterations (1000) reached and
the optimization hasn't converged yet.
  warnings.warn(
/projects/proteinml/.links/miniconda3/envs/aidep/lib/python3.10/site-
packages/sklearn/neural_network/_multilayer_perceptron.py:690:
ConvergenceWarning: Stochastic Optimizer: Maximum iterations (1000) reached and
the optimization hasn't converged yet.
  warnings.warn(
/projects/proteinml/.links/miniconda3/envs/aidep/lib/python3.10/site-
packages/sklearn/neural_network/_multilayer_perceptron.py:690:
ConvergenceWarning: Stochastic Optimizer: Maximum iterations (1000) reached and
```

```
the optimization hasn't converged yet.
  warnings.warn(
/projects/proteinml/.links/miniconda3/envs/aidep/lib/python3.10/site-
packages/sklearn/neural_network/_multilayer_perceptron.py:690:
ConvergenceWarning: Stochastic Optimizer: Maximum iterations (1000) reached and
the optimization hasn't converged yet.
  warnings.warn(
/projects/proteinml/.links/miniconda3/envs/aidep/lib/python3.10/site-
packages/sklearn/neural_network/_multilayer_perceptron.py:690:
ConvergenceWarning: Stochastic Optimizer: Maximum iterations (1000) reached and
the optimization hasn't converged yet.
  warnings.warn(
/projects/proteinml/.links/miniconda3/envs/aidep/lib/python3.10/site-
packages/sklearn/neural_network/_multilayer_perceptron.py:690:
ConvergenceWarning: Stochastic Optimizer: Maximum iterations (1000) reached and
the optimization hasn't converged yet.
  warnings.warn(
/projects/proteinml/.links/miniconda3/envs/aidep/lib/python3.10/site-
packages/sklearn/neural_network/_multilayer_perceptron.py:690:
ConvergenceWarning: Stochastic Optimizer: Maximum iterations (1000) reached and
the optimization hasn't converged yet.
  warnings.warn(
/projects/proteinml/.links/miniconda3/envs/aidep/lib/python3.10/site-
packages/sklearn/neural_network/_multilayer_perceptron.py:690:
ConvergenceWarning: Stochastic Optimizer: Maximum iterations (1000) reached and
the optimization hasn't converged yet.
  warnings.warn(
/projects/proteinml/.links/miniconda3/envs/aidep/lib/python3.10/site-
packages/sklearn/neural_network/_multilayer_perceptron.py:690:
ConvergenceWarning: Stochastic Optimizer: Maximum iterations (1000) reached and
the optimization hasn't converged yet.
  warnings.warn(
/projects/proteinml/.links/miniconda3/envs/aidep/lib/python3.10/site-
packages/sklearn/neural_network/_multilayer_perceptron.py:690:
ConvergenceWarning: Stochastic Optimizer: Maximum iterations (1000) reached and
the optimization hasn't converged yet.
  warnings.warn(
/projects/proteinml/.links/miniconda3/envs/aidep/lib/python3.10/site-
packages/sklearn/neural_network/_multilayer_perceptron.py:690:
ConvergenceWarning: Stochastic Optimizer: Maximum iterations (1000) reached and
the optimization hasn't converged yet.
  warnings.warn(
/projects/proteinml/.links/miniconda3/envs/aidep/lib/python3.10/site-
packages/sklearn/neural_network/_multilayer_perceptron.py:690:
ConvergenceWarning: Stochastic Optimizer: Maximum iterations (1000) reached and
the optimization hasn't converged yet.
  warnings.warn(
/projects/proteinml/.links/miniconda3/envs/aidep/lib/python3.10/site-
```

```
packages/sklearn/neural_network/_multilayer_perceptron.py:690:
ConvergenceWarning: Stochastic Optimizer: Maximum iterations (1000) reached and
the optimization hasn't converged yet.
  warnings.warn(
/projects/proteinml/.links/miniconda3/envs/aidep/lib/python3.10/site-
packages/sklearn/neural_network/_multilayer_perceptron.py:690:
ConvergenceWarning: Stochastic Optimizer: Maximum iterations (1000) reached and
the optimization hasn't converged yet.
  warnings.warn(
/projects/proteinml/.links/miniconda3/envs/aidep/lib/python3.10/site-
packages/sklearn/neural_network/_multilayer_perceptron.py:690:
ConvergenceWarning: Stochastic Optimizer: Maximum iterations (1000) reached and
the optimization hasn't converged yet.
  warnings.warn(
/projects/proteinml/.links/miniconda3/envs/aidep/lib/python3.10/site-
packages/sklearn/neural_network/_multilayer_perceptron.py:690:
ConvergenceWarning: Stochastic Optimizer: Maximum iterations (1000) reached and
the optimization hasn't converged yet.
  warnings.warn(
/projects/proteinml/.links/miniconda3/envs/aidep/lib/python3.10/site-
packages/sklearn/neural_network/_multilayer_perceptron.py:690:
ConvergenceWarning: Stochastic Optimizer: Maximum iterations (1000) reached and
the optimization hasn't converged yet.
  warnings.warn(
/projects/proteinml/.links/miniconda3/envs/aidep/lib/python3.10/site-
packages/sklearn/neural_network/_multilayer_perceptron.py:690:
ConvergenceWarning: Stochastic Optimizer: Maximum iterations (1000) reached and
the optimization hasn't converged yet.
  warnings.warn(
/projects/proteinml/.links/miniconda3/envs/aidep/lib/python3.10/site-
packages/sklearn/neural_network/_multilayer_perceptron.py:690:
ConvergenceWarning: Stochastic Optimizer: Maximum iterations (1000) reached and
the optimization hasn't converged yet.
  warnings.warn(
/projects/proteinml/.links/miniconda3/envs/aidep/lib/python3.10/site-
packages/sklearn/neural_network/_multilayer_perceptron.py:690:
ConvergenceWarning: Stochastic Optimizer: Maximum iterations (1000) reached and
the optimization hasn't converged yet.
  warnings.warn(
/projects/proteinml/.links/miniconda3/envs/aidep/lib/python3.10/site-
packages/sklearn/neural_network/_multilayer_perceptron.py:690:
ConvergenceWarning: Stochastic Optimizer: Maximum iterations (1000) reached and
the optimization hasn't converged yet.
  warnings.warn(
/projects/proteinml/.links/miniconda3/envs/aidep/lib/python3.10/site-
packages/sklearn/neural_network/_multilayer_perceptron.py:690:
ConvergenceWarning: Stochastic Optimizer: Maximum iterations (1000) reached and
the optimization hasn't converged yet.
```

```
  warnings.warn(
/projects/proteinml/.links/miniconda3/envs/aidep/lib/python3.10/site-
packages/sklearn/neural_network/_multilayer_perceptron.py:690:
ConvergenceWarning: Stochastic Optimizer: Maximum iterations (1000) reached and
the optimization hasn't converged yet.
  warnings.warn(
/projects/proteinml/.links/miniconda3/envs/aidep/lib/python3.10/site-
packages/sklearn/neural_network/_multilayer_perceptron.py:690:
ConvergenceWarning: Stochastic Optimizer: Maximum iterations (1000) reached and
the optimization hasn't converged yet.
  warnings.warn(
/projects/proteinml/.links/miniconda3/envs/aidep/lib/python3.10/site-
packages/sklearn/neural_network/_multilayer_perceptron.py:690:
ConvergenceWarning: Stochastic Optimizer: Maximum iterations (1000) reached and
the optimization hasn't converged yet.
  warnings.warn(
/projects/proteinml/.links/miniconda3/envs/aidep/lib/python3.10/site-
packages/sklearn/neural_network/_multilayer_perceptron.py:690:
ConvergenceWarning: Stochastic Optimizer: Maximum iterations (1000) reached and
the optimization hasn't converged yet.
  warnings.warn(
/projects/proteinml/.links/miniconda3/envs/aidep/lib/python3.10/site-
packages/sklearn/neural_network/_multilayer_perceptron.py:690:
ConvergenceWarning: Stochastic Optimizer: Maximum iterations (1000) reached and
the optimization hasn't converged yet.
  warnings.warn(
/projects/proteinml/.links/miniconda3/envs/aidep/lib/python3.10/site-
packages/sklearn/neural_network/_multilayer_perceptron.py:690:
ConvergenceWarning: Stochastic Optimizer: Maximum iterations (1000) reached and
the optimization hasn't converged yet.
  warnings.warn(
/projects/proteinml/.links/miniconda3/envs/aidep/lib/python3.10/site-
packages/sklearn/neural_network/_multilayer_perceptron.py:690:
ConvergenceWarning: Stochastic Optimizer: Maximum iterations (1000) reached and
the optimization hasn't converged yet.
  warnings.warn(
/projects/proteinml/.links/miniconda3/envs/aidep/lib/python3.10/site-
packages/sklearn/neural_network/_multilayer_perceptron.py:690:
ConvergenceWarning: Stochastic Optimizer: Maximum iterations (1000) reached and
the optimization hasn't converged yet.
  warnings.warn(
/projects/proteinml/.links/miniconda3/envs/aidep/lib/python3.10/site-
packages/sklearn/neural_network/_multilayer_perceptron.py:690:
ConvergenceWarning: Stochastic Optimizer: Maximum iterations (1000) reached and
the optimization hasn't converged yet.
  warnings.warn(
/projects/proteinml/.links/miniconda3/envs/aidep/lib/python3.10/site-
packages/sklearn/neural_network/_multilayer_perceptron.py:690:
```

```
ConvergenceWarning: Stochastic Optimizer: Maximum iterations (1000) reached and
the optimization hasn't converged yet.
  warnings.warn(
/projects/proteinml/.links/miniconda3/envs/aidep/lib/python3.10/site-
packages/sklearn/neural_network/_multilayer_perceptron.py:690:
ConvergenceWarning: Stochastic Optimizer: Maximum iterations (1000) reached and
the optimization hasn't converged yet.
  warnings.warn(
/projects/proteinml/.links/miniconda3/envs/aidep/lib/python3.10/site-
packages/sklearn/neural_network/_multilayer_perceptron.py:690:
ConvergenceWarning: Stochastic Optimizer: Maximum iterations (1000) reached and
the optimization hasn't converged yet.
  warnings.warn(
/projects/proteinml/.links/miniconda3/envs/aidep/lib/python3.10/site-
packages/sklearn/neural_network/_multilayer_perceptron.py:690:
ConvergenceWarning: Stochastic Optimizer: Maximum iterations (1000) reached and
the optimization hasn't converged yet.
  warnings.warn(
/projects/proteinml/.links/miniconda3/envs/aidep/lib/python3.10/site-
packages/sklearn/neural_network/_multilayer_perceptron.py:690:
ConvergenceWarning: Stochastic Optimizer: Maximum iterations (1000) reached and
the optimization hasn't converged yet.
  warnings.warn(
/projects/proteinml/.links/miniconda3/envs/aidep/lib/python3.10/site-
packages/sklearn/neural_network/_multilayer_perceptron.py:690:
ConvergenceWarning: Stochastic Optimizer: Maximum iterations (1000) reached and
the optimization hasn't converged yet.
  warnings.warn(
/projects/proteinml/.links/miniconda3/envs/aidep/lib/python3.10/site-
packages/sklearn/neural_network/_multilayer_perceptron.py:690:
ConvergenceWarning: Stochastic Optimizer: Maximum iterations (1000) reached and
the optimization hasn't converged yet.
  warnings.warn(
/projects/proteinml/.links/miniconda3/envs/aidep/lib/python3.10/site-
packages/sklearn/neural_network/_multilayer_perceptron.py:690:
ConvergenceWarning: Stochastic Optimizer: Maximum iterations (1000) reached and
the optimization hasn't converged yet.
  warnings.warn(
/projects/proteinml/.links/miniconda3/envs/aidep/lib/python3.10/site-
packages/sklearn/neural_network/_multilayer_perceptron.py:690:
ConvergenceWarning: Stochastic Optimizer: Maximum iterations (1000) reached and
the optimization hasn't converged yet.
  warnings.warn(
/projects/proteinml/.links/miniconda3/envs/aidep/lib/python3.10/site-
packages/sklearn/neural_network/_multilayer_perceptron.py:690:
ConvergenceWarning: Stochastic Optimizer: Maximum iterations (1000) reached and
the optimization hasn't converged yet.
  warnings.warn(
```

```
/projects/proteinml/.links/miniconda3/envs/aidep/lib/python3.10/site-
packages/sklearn/neural_network/_multilayer_perceptron.py:690:
ConvergenceWarning: Stochastic Optimizer: Maximum iterations (1000) reached and
the optimization hasn't converged yet.
  warnings.warn(
/projects/proteinml/.links/miniconda3/envs/aidep/lib/python3.10/site-
packages/sklearn/neural_network/_multilayer_perceptron.py:690:
ConvergenceWarning: Stochastic Optimizer: Maximum iterations (1000) reached and
the optimization hasn't converged yet.
  warnings.warn(
/projects/proteinml/.links/miniconda3/envs/aidep/lib/python3.10/site-
packages/sklearn/neural_network/_multilayer_perceptron.py:690:
ConvergenceWarning: Stochastic Optimizer: Maximum iterations (1000) reached and
the optimization hasn't converged yet.
  warnings.warn(
/projects/proteinml/.links/miniconda3/envs/aidep/lib/python3.10/site-
packages/sklearn/neural_network/_multilayer_perceptron.py:690:
ConvergenceWarning: Stochastic Optimizer: Maximum iterations (1000) reached and
the optimization hasn't converged yet.
  warnings.warn(
/projects/proteinml/.links/miniconda3/envs/aidep/lib/python3.10/site-
packages/sklearn/neural_network/_multilayer_perceptron.py:690:
ConvergenceWarning: Stochastic Optimizer: Maximum iterations (1000) reached and
the optimization hasn't converged yet.
  warnings.warn(
/projects/proteinml/.links/miniconda3/envs/aidep/lib/python3.10/site-
packages/sklearn/neural_network/_multilayer_perceptron.py:690:
ConvergenceWarning: Stochastic Optimizer: Maximum iterations (1000) reached and
the optimization hasn't converged yet.
  warnings.warn(
/projects/proteinml/.links/miniconda3/envs/aidep/lib/python3.10/site-
packages/sklearn/neural_network/_multilayer_perceptron.py:690:
ConvergenceWarning: Stochastic Optimizer: Maximum iterations (1000) reached and
the optimization hasn't converged yet.
  warnings.warn(
/projects/proteinml/.links/miniconda3/envs/aidep/lib/python3.10/site-
packages/sklearn/neural_network/_multilayer_perceptron.py:690:
ConvergenceWarning: Stochastic Optimizer: Maximum iterations (1000) reached and
the optimization hasn't converged yet.
  warnings.warn(
/projects/proteinml/.links/miniconda3/envs/aidep/lib/python3.10/site-
packages/sklearn/neural_network/_multilayer_perceptron.py:690:
ConvergenceWarning: Stochastic Optimizer: Maximum iterations (1000) reached and
the optimization hasn't converged yet.
  warnings.warn(
/projects/proteinml/.links/miniconda3/envs/aidep/lib/python3.10/site-
packages/sklearn/neural_network/_multilayer_perceptron.py:690:
ConvergenceWarning: Stochastic Optimizer: Maximum iterations (1000) reached and
```

```
the optimization hasn't converged yet.
  warnings.warn(
/projects/proteinml/.links/miniconda3/envs/aidep/lib/python3.10/site-
packages/sklearn/neural_network/_multilayer_perceptron.py:690:
ConvergenceWarning: Stochastic Optimizer: Maximum iterations (1000) reached and
the optimization hasn't converged yet.
  warnings.warn(
/projects/proteinml/.links/miniconda3/envs/aidep/lib/python3.10/site-
packages/sklearn/neural_network/_multilayer_perceptron.py:690:
ConvergenceWarning: Stochastic Optimizer: Maximum iterations (1000) reached and
the optimization hasn't converged yet.
  warnings.warn(
/projects/proteinml/.links/miniconda3/envs/aidep/lib/python3.10/site-
packages/sklearn/neural_network/_multilayer_perceptron.py:690:
ConvergenceWarning: Stochastic Optimizer: Maximum iterations (1000) reached and
the optimization hasn't converged yet.
  warnings.warn(
/projects/proteinml/.links/miniconda3/envs/aidep/lib/python3.10/site-
packages/sklearn/neural_network/_multilayer_perceptron.py:690:
ConvergenceWarning: Stochastic Optimizer: Maximum iterations (1000) reached and
the optimization hasn't converged yet.
  warnings.warn(
/projects/proteinml/.links/miniconda3/envs/aidep/lib/python3.10/site-
packages/sklearn/neural_network/_multilayer_perceptron.py:690:
ConvergenceWarning: Stochastic Optimizer: Maximum iterations (1000) reached and
the optimization hasn't converged yet.
  warnings.warn(
/projects/proteinml/.links/miniconda3/envs/aidep/lib/python3.10/site-
packages/sklearn/neural_network/_multilayer_perceptron.py:690:
ConvergenceWarning: Stochastic Optimizer: Maximum iterations (1000) reached and
the optimization hasn't converged yet.
  warnings.warn(
/projects/proteinml/.links/miniconda3/envs/aidep/lib/python3.10/site-
packages/sklearn/neural_network/_multilayer_perceptron.py:690:
ConvergenceWarning: Stochastic Optimizer: Maximum iterations (1000) reached and
the optimization hasn't converged yet.
  warnings.warn(
/projects/proteinml/.links/miniconda3/envs/aidep/lib/python3.10/site-
packages/sklearn/neural_network/_multilayer_perceptron.py:690:
ConvergenceWarning: Stochastic Optimizer: Maximum iterations (1000) reached and
the optimization hasn't converged yet.
  warnings.warn(
/projects/proteinml/.links/miniconda3/envs/aidep/lib/python3.10/site-
packages/sklearn/neural_network/_multilayer_perceptron.py:690:
ConvergenceWarning: Stochastic Optimizer: Maximum iterations (1000) reached and
the optimization hasn't converged yet.
  warnings.warn(
/projects/proteinml/.links/miniconda3/envs/aidep/lib/python3.10/site-
```

```
packages/sklearn/neural_network/_multilayer_perceptron.py:690:
ConvergenceWarning: Stochastic Optimizer: Maximum iterations (1000) reached and
the optimization hasn't converged yet.
  warnings.warn(
/projects/proteinml/.links/miniconda3/envs/aidep/lib/python3.10/site-
packages/sklearn/neural_network/_multilayer_perceptron.py:690:
ConvergenceWarning: Stochastic Optimizer: Maximum iterations (1000) reached and
the optimization hasn't converged yet.
  warnings.warn(
/projects/proteinml/.links/miniconda3/envs/aidep/lib/python3.10/site-
packages/sklearn/neural_network/_multilayer_perceptron.py:690:
ConvergenceWarning: Stochastic Optimizer: Maximum iterations (1000) reached and
the optimization hasn't converged yet.
  warnings.warn(
/projects/proteinml/.links/miniconda3/envs/aidep/lib/python3.10/site-
packages/sklearn/neural_network/_multilayer_perceptron.py:690:
ConvergenceWarning: Stochastic Optimizer: Maximum iterations (1000) reached and
the optimization hasn't converged yet.
  warnings.warn(
/projects/proteinml/.links/miniconda3/envs/aidep/lib/python3.10/site-
packages/sklearn/neural_network/_multilayer_perceptron.py:690:
ConvergenceWarning: Stochastic Optimizer: Maximum iterations (1000) reached and
the optimization hasn't converged yet.
  warnings.warn(
/projects/proteinml/.links/miniconda3/envs/aidep/lib/python3.10/site-
packages/sklearn/neural_network/_multilayer_perceptron.py:690:
ConvergenceWarning: Stochastic Optimizer: Maximum iterations (1000) reached and
the optimization hasn't converged yet.
  warnings.warn(
/projects/proteinml/.links/miniconda3/envs/aidep/lib/python3.10/site-
packages/sklearn/neural_network/_multilayer_perceptron.py:690:
ConvergenceWarning: Stochastic Optimizer: Maximum iterations (1000) reached and
the optimization hasn't converged yet.
  warnings.warn(
/projects/proteinml/.links/miniconda3/envs/aidep/lib/python3.10/site-
packages/sklearn/neural_network/_multilayer_perceptron.py:690:
ConvergenceWarning: Stochastic Optimizer: Maximum iterations (1000) reached and
the optimization hasn't converged yet.
  warnings.warn(
/projects/proteinml/.links/miniconda3/envs/aidep/lib/python3.10/site-
packages/sklearn/neural_network/_multilayer_perceptron.py:690:
ConvergenceWarning: Stochastic Optimizer: Maximum iterations (1000) reached and
the optimization hasn't converged yet.
  warnings.warn(
/projects/proteinml/.links/miniconda3/envs/aidep/lib/python3.10/site-
packages/sklearn/neural_network/_multilayer_perceptron.py:690:
ConvergenceWarning: Stochastic Optimizer: Maximum iterations (1000) reached and
the optimization hasn't converged yet.
```

```
  warnings.warn(
/projects/proteinml/.links/miniconda3/envs/aidep/lib/python3.10/site-
packages/sklearn/neural_network/_multilayer_perceptron.py:690:
ConvergenceWarning: Stochastic Optimizer: Maximum iterations (1000) reached and
the optimization hasn't converged yet.
  warnings.warn(
/projects/proteinml/.links/miniconda3/envs/aidep/lib/python3.10/site-
packages/sklearn/neural_network/_multilayer_perceptron.py:690:
ConvergenceWarning: Stochastic Optimizer: Maximum iterations (1000) reached and
the optimization hasn't converged yet.
  warnings.warn(
/projects/proteinml/.links/miniconda3/envs/aidep/lib/python3.10/site-
packages/sklearn/neural_network/_multilayer_perceptron.py:690:
ConvergenceWarning: Stochastic Optimizer: Maximum iterations (1000) reached and
the optimization hasn't converged yet.
  warnings.warn(
/projects/proteinml/.links/miniconda3/envs/aidep/lib/python3.10/site-
packages/sklearn/neural_network/_multilayer_perceptron.py:690:
ConvergenceWarning: Stochastic Optimizer: Maximum iterations (1000) reached and
the optimization hasn't converged yet.
  warnings.warn(
/projects/proteinml/.links/miniconda3/envs/aidep/lib/python3.10/site-
packages/sklearn/neural_network/_multilayer_perceptron.py:690:
ConvergenceWarning: Stochastic Optimizer: Maximum iterations (1000) reached and
the optimization hasn't converged yet.
  warnings.warn(
/projects/proteinml/.links/miniconda3/envs/aidep/lib/python3.10/site-
packages/sklearn/neural_network/_multilayer_perceptron.py:690:
ConvergenceWarning: Stochastic Optimizer: Maximum iterations (1000) reached and
the optimization hasn't converged yet.
  warnings.warn(
/projects/proteinml/.links/miniconda3/envs/aidep/lib/python3.10/site-
packages/sklearn/neural_network/_multilayer_perceptron.py:690:
ConvergenceWarning: Stochastic Optimizer: Maximum iterations (1000) reached and
the optimization hasn't converged yet.
  warnings.warn(
/projects/proteinml/.links/miniconda3/envs/aidep/lib/python3.10/site-
packages/sklearn/neural_network/_multilayer_perceptron.py:690:
ConvergenceWarning: Stochastic Optimizer: Maximum iterations (1000) reached and
the optimization hasn't converged yet.
  warnings.warn(
/projects/proteinml/.links/miniconda3/envs/aidep/lib/python3.10/site-
packages/sklearn/neural_network/_multilayer_perceptron.py:690:
ConvergenceWarning: Stochastic Optimizer: Maximum iterations (1000) reached and
the optimization hasn't converged yet.
  warnings.warn(
/projects/proteinml/.links/miniconda3/envs/aidep/lib/python3.10/site-
packages/sklearn/neural_network/_multilayer_perceptron.py:690:
```

```
ConvergenceWarning: Stochastic Optimizer: Maximum iterations (1000) reached and
the optimization hasn't converged yet.
  warnings.warn(
/projects/proteinml/.links/miniconda3/envs/aidep/lib/python3.10/site-
packages/sklearn/neural_network/_multilayer_perceptron.py:690:
ConvergenceWarning: Stochastic Optimizer: Maximum iterations (1000) reached and
the optimization hasn't converged yet.
  warnings.warn(
/projects/proteinml/.links/miniconda3/envs/aidep/lib/python3.10/site-
packages/sklearn/neural_network/_multilayer_perceptron.py:690:
ConvergenceWarning: Stochastic Optimizer: Maximum iterations (1000) reached and
the optimization hasn't converged yet.
  warnings.warn(
/projects/proteinml/.links/miniconda3/envs/aidep/lib/python3.10/site-
packages/sklearn/neural_network/_multilayer_perceptron.py:690:
ConvergenceWarning: Stochastic Optimizer: Maximum iterations (1000) reached and
the optimization hasn't converged yet.
  warnings.warn(
/projects/proteinml/.links/miniconda3/envs/aidep/lib/python3.10/site-
packages/sklearn/neural_network/_multilayer_perceptron.py:690:
ConvergenceWarning: Stochastic Optimizer: Maximum iterations (1000) reached and
the optimization hasn't converged yet.
  warnings.warn(
/projects/proteinml/.links/miniconda3/envs/aidep/lib/python3.10/site-
packages/sklearn/neural_network/_multilayer_perceptron.py:690:
ConvergenceWarning: Stochastic Optimizer: Maximum iterations (1000) reached and
the optimization hasn't converged yet.
  warnings.warn(
/projects/proteinml/.links/miniconda3/envs/aidep/lib/python3.10/site-
packages/sklearn/neural_network/_multilayer_perceptron.py:690:
ConvergenceWarning: Stochastic Optimizer: Maximum iterations (1000) reached and
the optimization hasn't converged yet.
  warnings.warn(
/projects/proteinml/.links/miniconda3/envs/aidep/lib/python3.10/site-
packages/sklearn/neural_network/_multilayer_perceptron.py:690:
ConvergenceWarning: Stochastic Optimizer: Maximum iterations (1000) reached and
the optimization hasn't converged yet.
  warnings.warn(
/projects/proteinml/.links/miniconda3/envs/aidep/lib/python3.10/site-
packages/sklearn/neural_network/_multilayer_perceptron.py:690:
ConvergenceWarning: Stochastic Optimizer: Maximum iterations (1000) reached and
the optimization hasn't converged yet.
  warnings.warn(
/projects/proteinml/.links/miniconda3/envs/aidep/lib/python3.10/site-
packages/sklearn/neural_network/_multilayer_perceptron.py:690:
ConvergenceWarning: Stochastic Optimizer: Maximum iterations (1000) reached and
the optimization hasn't converged yet.
  warnings.warn(
```

```
/projects/proteinml/.links/miniconda3/envs/aidep/lib/python3.10/site-
packages/sklearn/neural_network/_multilayer_perceptron.py:690:
ConvergenceWarning: Stochastic Optimizer: Maximum iterations (1000) reached and
the optimization hasn't converged yet.
  warnings.warn(
/projects/proteinml/.links/miniconda3/envs/aidep/lib/python3.10/site-
packages/sklearn/neural_network/_multilayer_perceptron.py:690:
ConvergenceWarning: Stochastic Optimizer: Maximum iterations (1000) reached and
the optimization hasn't converged yet.
  warnings.warn(
/projects/proteinml/.links/miniconda3/envs/aidep/lib/python3.10/site-
packages/sklearn/neural_network/_multilayer_perceptron.py:690:
ConvergenceWarning: Stochastic Optimizer: Maximum iterations (1000) reached and
the optimization hasn't converged yet.
  warnings.warn(
/projects/proteinml/.links/miniconda3/envs/aidep/lib/python3.10/site-
packages/sklearn/neural_network/_multilayer_perceptron.py:690:
ConvergenceWarning: Stochastic Optimizer: Maximum iterations (1000) reached and
the optimization hasn't converged yet.
  warnings.warn(
/projects/proteinml/.links/miniconda3/envs/aidep/lib/python3.10/site-
packages/sklearn/neural_network/_multilayer_perceptron.py:690:
ConvergenceWarning: Stochastic Optimizer: Maximum iterations (1000) reached and
the optimization hasn't converged yet.
  warnings.warn(
/projects/proteinml/.links/miniconda3/envs/aidep/lib/python3.10/site-
packages/sklearn/neural_network/_multilayer_perceptron.py:690:
ConvergenceWarning: Stochastic Optimizer: Maximum iterations (1000) reached and
the optimization hasn't converged yet.
  warnings.warn(
/projects/proteinml/.links/miniconda3/envs/aidep/lib/python3.10/site-
packages/sklearn/neural_network/_multilayer_perceptron.py:690:
ConvergenceWarning: Stochastic Optimizer: Maximum iterations (1000) reached and
the optimization hasn't converged yet.
  warnings.warn(
/projects/proteinml/.links/miniconda3/envs/aidep/lib/python3.10/site-
packages/sklearn/neural_network/_multilayer_perceptron.py:690:
ConvergenceWarning: Stochastic Optimizer: Maximum iterations (1000) reached and
the optimization hasn't converged yet.
  warnings.warn(
/projects/proteinml/.links/miniconda3/envs/aidep/lib/python3.10/site-
packages/sklearn/neural_network/_multilayer_perceptron.py:690:
ConvergenceWarning: Stochastic Optimizer: Maximum iterations (1000) reached and
the optimization hasn't converged yet.
  warnings.warn(
/projects/proteinml/.links/miniconda3/envs/aidep/lib/python3.10/site-
packages/sklearn/neural_network/_multilayer_perceptron.py:690:
ConvergenceWarning: Stochastic Optimizer: Maximum iterations (1000) reached and
```

```
the optimization hasn't converged yet.
  warnings.warn(
/projects/proteinml/.links/miniconda3/envs/aidep/lib/python3.10/site-
packages/sklearn/neural_network/_multilayer_perceptron.py:690:
ConvergenceWarning: Stochastic Optimizer: Maximum iterations (1000) reached and
the optimization hasn't converged yet.
  warnings.warn(
/projects/proteinml/.links/miniconda3/envs/aidep/lib/python3.10/site-
packages/sklearn/neural_network/_multilayer_perceptron.py:690:
ConvergenceWarning: Stochastic Optimizer: Maximum iterations (1000) reached and
the optimization hasn't converged yet.
  warnings.warn(
/projects/proteinml/.links/miniconda3/envs/aidep/lib/python3.10/site-
packages/sklearn/neural_network/_multilayer_perceptron.py:690:
ConvergenceWarning: Stochastic Optimizer: Maximum iterations (1000) reached and
the optimization hasn't converged yet.
  warnings.warn(
/projects/proteinml/.links/miniconda3/envs/aidep/lib/python3.10/site-
packages/sklearn/neural_network/_multilayer_perceptron.py:690:
ConvergenceWarning: Stochastic Optimizer: Maximum iterations (1000) reached and
the optimization hasn't converged yet.
  warnings.warn(
/projects/proteinml/.links/miniconda3/envs/aidep/lib/python3.10/site-
packages/sklearn/neural_network/_multilayer_perceptron.py:690:
ConvergenceWarning: Stochastic Optimizer: Maximum iterations (1000) reached and
the optimization hasn't converged yet.
  warnings.warn(
/projects/proteinml/.links/miniconda3/envs/aidep/lib/python3.10/site-
packages/sklearn/neural_network/_multilayer_perceptron.py:690:
ConvergenceWarning: Stochastic Optimizer: Maximum iterations (1000) reached and
the optimization hasn't converged yet.
  warnings.warn(
/projects/proteinml/.links/miniconda3/envs/aidep/lib/python3.10/site-
packages/sklearn/neural_network/_multilayer_perceptron.py:690:
ConvergenceWarning: Stochastic Optimizer: Maximum iterations (1000) reached and
the optimization hasn't converged yet.
  warnings.warn(
/projects/proteinml/.links/miniconda3/envs/aidep/lib/python3.10/site-
packages/sklearn/neural_network/_multilayer_perceptron.py:690:
ConvergenceWarning: Stochastic Optimizer: Maximum iterations (1000) reached and
the optimization hasn't converged yet.
  warnings.warn(
/projects/proteinml/.links/miniconda3/envs/aidep/lib/python3.10/site-
packages/sklearn/neural_network/_multilayer_perceptron.py:690:
ConvergenceWarning: Stochastic Optimizer: Maximum iterations (1000) reached and
the optimization hasn't converged yet.
  warnings.warn(
/projects/proteinml/.links/miniconda3/envs/aidep/lib/python3.10/site-
```

```
packages/sklearn/neural_network/_multilayer_perceptron.py:690:
ConvergenceWarning: Stochastic Optimizer: Maximum iterations (1000) reached and
the optimization hasn't converged yet.
  warnings.warn(
/projects/proteinml/.links/miniconda3/envs/aidep/lib/python3.10/site-
packages/sklearn/neural_network/_multilayer_perceptron.py:690:
ConvergenceWarning: Stochastic Optimizer: Maximum iterations (1000) reached and
the optimization hasn't converged yet.
  warnings.warn(
/projects/proteinml/.links/miniconda3/envs/aidep/lib/python3.10/site-
packages/sklearn/neural_network/_multilayer_perceptron.py:690:
ConvergenceWarning: Stochastic Optimizer: Maximum iterations (1000) reached and
the optimization hasn't converged yet.
  warnings.warn(
/projects/proteinml/.links/miniconda3/envs/aidep/lib/python3.10/site-
packages/sklearn/neural_network/_multilayer_perceptron.py:690:
ConvergenceWarning: Stochastic Optimizer: Maximum iterations (1000) reached and
the optimization hasn't converged yet.
  warnings.warn(
/projects/proteinml/.links/miniconda3/envs/aidep/lib/python3.10/site-
packages/sklearn/neural_network/_multilayer_perceptron.py:690:
ConvergenceWarning: Stochastic Optimizer: Maximum iterations (1000) reached and
the optimization hasn't converged yet.
  warnings.warn(
/projects/proteinml/.links/miniconda3/envs/aidep/lib/python3.10/site-
packages/sklearn/neural_network/_multilayer_perceptron.py:690:
ConvergenceWarning: Stochastic Optimizer: Maximum iterations (1000) reached and
the optimization hasn't converged yet.
  warnings.warn(
/projects/proteinml/.links/miniconda3/envs/aidep/lib/python3.10/site-
packages/sklearn/neural_network/_multilayer_perceptron.py:690:
ConvergenceWarning: Stochastic Optimizer: Maximum iterations (1000) reached and
the optimization hasn't converged yet.
  warnings.warn(
/projects/proteinml/.links/miniconda3/envs/aidep/lib/python3.10/site-
packages/sklearn/neural_network/_multilayer_perceptron.py:690:
ConvergenceWarning: Stochastic Optimizer: Maximum iterations (1000) reached and
the optimization hasn't converged yet.
  warnings.warn(
/projects/proteinml/.links/miniconda3/envs/aidep/lib/python3.10/site-
packages/sklearn/neural_network/_multilayer_perceptron.py:690:
ConvergenceWarning: Stochastic Optimizer: Maximum iterations (1000) reached and
the optimization hasn't converged yet.
  warnings.warn(
/projects/proteinml/.links/miniconda3/envs/aidep/lib/python3.10/site-
packages/sklearn/neural_network/_multilayer_perceptron.py:690:
ConvergenceWarning: Stochastic Optimizer: Maximum iterations (1000) reached and
the optimization hasn't converged yet.
```

```
  warnings.warn(
/projects/proteinml/.links/miniconda3/envs/aidep/lib/python3.10/site-
packages/sklearn/neural_network/_multilayer_perceptron.py:690:
ConvergenceWarning: Stochastic Optimizer: Maximum iterations (1000) reached and
the optimization hasn't converged yet.
  warnings.warn(
/projects/proteinml/.links/miniconda3/envs/aidep/lib/python3.10/site-
packages/sklearn/neural_network/_multilayer_perceptron.py:690:
ConvergenceWarning: Stochastic Optimizer: Maximum iterations (1000) reached and
the optimization hasn't converged yet.
  warnings.warn(
/projects/proteinml/.links/miniconda3/envs/aidep/lib/python3.10/site-
packages/sklearn/neural_network/_multilayer_perceptron.py:690:
ConvergenceWarning: Stochastic Optimizer: Maximum iterations (1000) reached and
the optimization hasn't converged yet.
  warnings.warn(
/projects/proteinml/.links/miniconda3/envs/aidep/lib/python3.10/site-
packages/sklearn/neural_network/_multilayer_perceptron.py:690:
ConvergenceWarning: Stochastic Optimizer: Maximum iterations (1000) reached and
the optimization hasn't converged yet.
  warnings.warn(
/projects/proteinml/.links/miniconda3/envs/aidep/lib/python3.10/site-
packages/sklearn/neural_network/_multilayer_perceptron.py:690:
ConvergenceWarning: Stochastic Optimizer: Maximum iterations (1000) reached and
the optimization hasn't converged yet.
  warnings.warn(
/projects/proteinml/.links/miniconda3/envs/aidep/lib/python3.10/site-
packages/sklearn/neural_network/_multilayer_perceptron.py:690:
ConvergenceWarning: Stochastic Optimizer: Maximum iterations (1000) reached and
the optimization hasn't converged yet.
  warnings.warn(
/projects/proteinml/.links/miniconda3/envs/aidep/lib/python3.10/site-
packages/sklearn/neural_network/_multilayer_perceptron.py:690:
ConvergenceWarning: Stochastic Optimizer: Maximum iterations (1000) reached and
the optimization hasn't converged yet.
  warnings.warn(
/projects/proteinml/.links/miniconda3/envs/aidep/lib/python3.10/site-
packages/sklearn/neural_network/_multilayer_perceptron.py:690:
ConvergenceWarning: Stochastic Optimizer: Maximum iterations (1000) reached and
the optimization hasn't converged yet.
  warnings.warn(
/projects/proteinml/.links/miniconda3/envs/aidep/lib/python3.10/site-
packages/sklearn/neural_network/_multilayer_perceptron.py:690:
ConvergenceWarning: Stochastic Optimizer: Maximum iterations (1000) reached and
the optimization hasn't converged yet.
  warnings.warn(
/projects/proteinml/.links/miniconda3/envs/aidep/lib/python3.10/site-
packages/sklearn/neural_network/_multilayer_perceptron.py:690:
```

```
ConvergenceWarning: Stochastic Optimizer: Maximum iterations (1000) reached and
the optimization hasn't converged yet.
  warnings.warn(
/projects/proteinml/.links/miniconda3/envs/aidep/lib/python3.10/site-
packages/sklearn/neural_network/_multilayer_perceptron.py:690:
ConvergenceWarning: Stochastic Optimizer: Maximum iterations (1000) reached and
the optimization hasn't converged yet.
  warnings.warn(
/projects/proteinml/.links/miniconda3/envs/aidep/lib/python3.10/site-
packages/sklearn/neural_network/_multilayer_perceptron.py:690:
ConvergenceWarning: Stochastic Optimizer: Maximum iterations (1000) reached and
the optimization hasn't converged yet.
  warnings.warn(
/projects/proteinml/.links/miniconda3/envs/aidep/lib/python3.10/site-
packages/sklearn/neural_network/_multilayer_perceptron.py:690:
ConvergenceWarning: Stochastic Optimizer: Maximum iterations (1000) reached and
the optimization hasn't converged yet.
  warnings.warn(
/projects/proteinml/.links/miniconda3/envs/aidep/lib/python3.10/site-
packages/sklearn/neural_network/_multilayer_perceptron.py:690:
ConvergenceWarning: Stochastic Optimizer: Maximum iterations (1000) reached and
the optimization hasn't converged yet.
  warnings.warn(
/projects/proteinml/.links/miniconda3/envs/aidep/lib/python3.10/site-
packages/sklearn/neural_network/_multilayer_perceptron.py:690:
ConvergenceWarning: Stochastic Optimizer: Maximum iterations (1000) reached and
the optimization hasn't converged yet.
  warnings.warn(
/projects/proteinml/.links/miniconda3/envs/aidep/lib/python3.10/site-
packages/sklearn/neural_network/_multilayer_perceptron.py:690:
ConvergenceWarning: Stochastic Optimizer: Maximum iterations (1000) reached and
the optimization hasn't converged yet.
  warnings.warn(
/projects/proteinml/.links/miniconda3/envs/aidep/lib/python3.10/site-
packages/sklearn/neural_network/_multilayer_perceptron.py:690:
ConvergenceWarning: Stochastic Optimizer: Maximum iterations (1000) reached and
the optimization hasn't converged yet.
  warnings.warn(
/projects/proteinml/.links/miniconda3/envs/aidep/lib/python3.10/site-
packages/sklearn/neural_network/_multilayer_perceptron.py:690:
ConvergenceWarning: Stochastic Optimizer: Maximum iterations (1000) reached and
the optimization hasn't converged yet.
  warnings.warn(
/projects/proteinml/.links/miniconda3/envs/aidep/lib/python3.10/site-
packages/sklearn/neural_network/_multilayer_perceptron.py:690:
ConvergenceWarning: Stochastic Optimizer: Maximum iterations (1000) reached and
the optimization hasn't converged yet.
  warnings.warn(
```

```
/projects/proteinml/.links/miniconda3/envs/aidep/lib/python3.10/site-
packages/sklearn/neural_network/_multilayer_perceptron.py:690:
ConvergenceWarning: Stochastic Optimizer: Maximum iterations (1000) reached and
the optimization hasn't converged yet.
  warnings.warn(
/projects/proteinml/.links/miniconda3/envs/aidep/lib/python3.10/site-
packages/sklearn/neural_network/_multilayer_perceptron.py:690:
ConvergenceWarning: Stochastic Optimizer: Maximum iterations (1000) reached and
the optimization hasn't converged yet.
  warnings.warn(
/projects/proteinml/.links/miniconda3/envs/aidep/lib/python3.10/site-
packages/sklearn/neural_network/_multilayer_perceptron.py:690:
ConvergenceWarning: Stochastic Optimizer: Maximum iterations (1000) reached and
the optimization hasn't converged yet.
  warnings.warn(
/projects/proteinml/.links/miniconda3/envs/aidep/lib/python3.10/site-
packages/sklearn/neural_network/_multilayer_perceptron.py:690:
ConvergenceWarning: Stochastic Optimizer: Maximum iterations (1000) reached and
the optimization hasn't converged yet.
  warnings.warn(
/projects/proteinml/.links/miniconda3/envs/aidep/lib/python3.10/site-
packages/sklearn/neural_network/_multilayer_perceptron.py:690:
ConvergenceWarning: Stochastic Optimizer: Maximum iterations (1000) reached and
the optimization hasn't converged yet.
  warnings.warn(
/projects/proteinml/.links/miniconda3/envs/aidep/lib/python3.10/site-
packages/sklearn/neural_network/_multilayer_perceptron.py:690:
ConvergenceWarning: Stochastic Optimizer: Maximum iterations (1000) reached and
the optimization hasn't converged yet.
  warnings.warn(
/projects/proteinml/.links/miniconda3/envs/aidep/lib/python3.10/site-
packages/sklearn/neural_network/_multilayer_perceptron.py:690:
ConvergenceWarning: Stochastic Optimizer: Maximum iterations (1000) reached and
the optimization hasn't converged yet.
  warnings.warn(
/projects/proteinml/.links/miniconda3/envs/aidep/lib/python3.10/site-
packages/sklearn/neural_network/_multilayer_perceptron.py:690:
ConvergenceWarning: Stochastic Optimizer: Maximum iterations (1000) reached and
the optimization hasn't converged yet.
  warnings.warn(
/projects/proteinml/.links/miniconda3/envs/aidep/lib/python3.10/site-
packages/sklearn/neural_network/_multilayer_perceptron.py:690:
ConvergenceWarning: Stochastic Optimizer: Maximum iterations (1000) reached and
the optimization hasn't converged yet.
  warnings.warn(
/projects/proteinml/.links/miniconda3/envs/aidep/lib/python3.10/site-
packages/sklearn/neural_network/_multilayer_perceptron.py:690:
ConvergenceWarning: Stochastic Optimizer: Maximum iterations (1000) reached and
```

```
the optimization hasn't converged yet.
  warnings.warn(
/projects/proteinml/.links/miniconda3/envs/aidep/lib/python3.10/site-
packages/sklearn/neural_network/_multilayer_perceptron.py:690:
ConvergenceWarning: Stochastic Optimizer: Maximum iterations (1000) reached and
the optimization hasn't converged yet.
  warnings.warn(
/projects/proteinml/.links/miniconda3/envs/aidep/lib/python3.10/site-
packages/sklearn/neural_network/_multilayer_perceptron.py:690:
ConvergenceWarning: Stochastic Optimizer: Maximum iterations (1000) reached and
the optimization hasn't converged yet.
  warnings.warn(
/projects/proteinml/.links/miniconda3/envs/aidep/lib/python3.10/site-
packages/sklearn/neural_network/_multilayer_perceptron.py:690:
ConvergenceWarning: Stochastic Optimizer: Maximum iterations (1000) reached and
the optimization hasn't converged yet.
  warnings.warn(
/projects/proteinml/.links/miniconda3/envs/aidep/lib/python3.10/site-
packages/sklearn/neural_network/_multilayer_perceptron.py:690:
ConvergenceWarning: Stochastic Optimizer: Maximum iterations (1000) reached and
the optimization hasn't converged yet.
  warnings.warn(
/projects/proteinml/.links/miniconda3/envs/aidep/lib/python3.10/site-
packages/sklearn/neural_network/_multilayer_perceptron.py:690:
ConvergenceWarning: Stochastic Optimizer: Maximum iterations (1000) reached and
the optimization hasn't converged yet.
  warnings.warn(
/projects/proteinml/.links/miniconda3/envs/aidep/lib/python3.10/site-
packages/sklearn/neural_network/_multilayer_perceptron.py:690:
ConvergenceWarning: Stochastic Optimizer: Maximum iterations (1000) reached and
the optimization hasn't converged yet.
  warnings.warn(
/projects/proteinml/.links/miniconda3/envs/aidep/lib/python3.10/site-
packages/sklearn/neural_network/_multilayer_perceptron.py:690:
ConvergenceWarning: Stochastic Optimizer: Maximum iterations (1000) reached and
the optimization hasn't converged yet.
  warnings.warn(
/projects/proteinml/.links/miniconda3/envs/aidep/lib/python3.10/site-
packages/sklearn/neural_network/_multilayer_perceptron.py:690:
ConvergenceWarning: Stochastic Optimizer: Maximum iterations (1000) reached and
the optimization hasn't converged yet.
  warnings.warn(
/projects/proteinml/.links/miniconda3/envs/aidep/lib/python3.10/site-
packages/sklearn/neural_network/_multilayer_perceptron.py:690:
ConvergenceWarning: Stochastic Optimizer: Maximum iterations (1000) reached and
the optimization hasn't converged yet.
  warnings.warn(
/projects/proteinml/.links/miniconda3/envs/aidep/lib/python3.10/site-
```

```
packages/sklearn/neural_network/_multilayer_perceptron.py:690:
ConvergenceWarning: Stochastic Optimizer: Maximum iterations (1000) reached and
the optimization hasn't converged yet.
  warnings.warn(
/projects/proteinml/.links/miniconda3/envs/aidep/lib/python3.10/site-
packages/sklearn/neural_network/_multilayer_perceptron.py:690:
ConvergenceWarning: Stochastic Optimizer: Maximum iterations (1000) reached and
the optimization hasn't converged yet.
  warnings.warn(
/projects/proteinml/.links/miniconda3/envs/aidep/lib/python3.10/site-
packages/sklearn/neural_network/_multilayer_perceptron.py:690:
ConvergenceWarning: Stochastic Optimizer: Maximum iterations (1000) reached and
the optimization hasn't converged yet.
  warnings.warn(
/projects/proteinml/.links/miniconda3/envs/aidep/lib/python3.10/site-
packages/sklearn/neural_network/_multilayer_perceptron.py:690:
ConvergenceWarning: Stochastic Optimizer: Maximum iterations (1000) reached and
the optimization hasn't converged yet.
  warnings.warn(
/projects/proteinml/.links/miniconda3/envs/aidep/lib/python3.10/site-
packages/sklearn/neural_network/_multilayer_perceptron.py:690:
ConvergenceWarning: Stochastic Optimizer: Maximum iterations (1000) reached and
the optimization hasn't converged yet.
  warnings.warn(
/projects/proteinml/.links/miniconda3/envs/aidep/lib/python3.10/site-
packages/sklearn/neural_network/_multilayer_perceptron.py:690:
ConvergenceWarning: Stochastic Optimizer: Maximum iterations (1000) reached and
the optimization hasn't converged yet.
  warnings.warn(
/projects/proteinml/.links/miniconda3/envs/aidep/lib/python3.10/site-
packages/sklearn/neural_network/_multilayer_perceptron.py:690:
ConvergenceWarning: Stochastic Optimizer: Maximum iterations (1000) reached and
the optimization hasn't converged yet.
  warnings.warn(
/projects/proteinml/.links/miniconda3/envs/aidep/lib/python3.10/site-
packages/sklearn/neural_network/_multilayer_perceptron.py:690:
ConvergenceWarning: Stochastic Optimizer: Maximum iterations (1000) reached and
the optimization hasn't converged yet.
  warnings.warn(
/projects/proteinml/.links/miniconda3/envs/aidep/lib/python3.10/site-
packages/sklearn/neural_network/_multilayer_perceptron.py:690:
ConvergenceWarning: Stochastic Optimizer: Maximum iterations (1000) reached and
the optimization hasn't converged yet.
  warnings.warn(
/projects/proteinml/.links/miniconda3/envs/aidep/lib/python3.10/site-
packages/sklearn/neural_network/_multilayer_perceptron.py:690:
ConvergenceWarning: Stochastic Optimizer: Maximum iterations (1000) reached and
the optimization hasn't converged yet.
```

```
  warnings.warn(
/projects/proteinml/.links/miniconda3/envs/aidep/lib/python3.10/site-
packages/sklearn/neural_network/_multilayer_perceptron.py:690:
ConvergenceWarning: Stochastic Optimizer: Maximum iterations (1000) reached and
the optimization hasn't converged yet.
  warnings.warn(
/projects/proteinml/.links/miniconda3/envs/aidep/lib/python3.10/site-
packages/sklearn/neural_network/_multilayer_perceptron.py:690:
ConvergenceWarning: Stochastic Optimizer: Maximum iterations (1000) reached and
the optimization hasn't converged yet.
  warnings.warn(
/projects/proteinml/.links/miniconda3/envs/aidep/lib/python3.10/site-
packages/sklearn/neural_network/_multilayer_perceptron.py:690:
ConvergenceWarning: Stochastic Optimizer: Maximum iterations (1000) reached and
the optimization hasn't converged yet.
  warnings.warn(
/projects/proteinml/.links/miniconda3/envs/aidep/lib/python3.10/site-
packages/sklearn/neural_network/_multilayer_perceptron.py:690:
ConvergenceWarning: Stochastic Optimizer: Maximum iterations (1000) reached and
the optimization hasn't converged yet.
  warnings.warn(
/projects/proteinml/.links/miniconda3/envs/aidep/lib/python3.10/site-
packages/sklearn/neural_network/_multilayer_perceptron.py:690:
ConvergenceWarning: Stochastic Optimizer: Maximum iterations (1000) reached and
the optimization hasn't converged yet.
  warnings.warn(
/projects/proteinml/.links/miniconda3/envs/aidep/lib/python3.10/site-
packages/sklearn/neural_network/_multilayer_perceptron.py:690:
ConvergenceWarning: Stochastic Optimizer: Maximum iterations (1000) reached and
the optimization hasn't converged yet.
  warnings.warn(
/projects/proteinml/.links/miniconda3/envs/aidep/lib/python3.10/site-
packages/sklearn/neural_network/_multilayer_perceptron.py:690:
ConvergenceWarning: Stochastic Optimizer: Maximum iterations (1000) reached and
the optimization hasn't converged yet.
  warnings.warn(
/projects/proteinml/.links/miniconda3/envs/aidep/lib/python3.10/site-
packages/sklearn/neural_network/_multilayer_perceptron.py:690:
ConvergenceWarning: Stochastic Optimizer: Maximum iterations (1000) reached and
the optimization hasn't converged yet.
  warnings.warn(
/projects/proteinml/.links/miniconda3/envs/aidep/lib/python3.10/site-
packages/sklearn/neural_network/_multilayer_perceptron.py:690:
ConvergenceWarning: Stochastic Optimizer: Maximum iterations (1000) reached and
the optimization hasn't converged yet.
  warnings.warn(
/projects/proteinml/.links/miniconda3/envs/aidep/lib/python3.10/site-
packages/sklearn/neural_network/_multilayer_perceptron.py:690:
```

```
ConvergenceWarning: Stochastic Optimizer: Maximum iterations (1000) reached and
the optimization hasn't converged yet.
  warnings.warn(
/projects/proteinml/.links/miniconda3/envs/aidep/lib/python3.10/site-
packages/sklearn/neural_network/_multilayer_perceptron.py:690:
ConvergenceWarning: Stochastic Optimizer: Maximum iterations (1000) reached and
the optimization hasn't converged yet.
  warnings.warn(
/projects/proteinml/.links/miniconda3/envs/aidep/lib/python3.10/site-
packages/sklearn/neural_network/_multilayer_perceptron.py:690:
ConvergenceWarning: Stochastic Optimizer: Maximum iterations (1000) reached and
the optimization hasn't converged yet.
  warnings.warn(
/projects/proteinml/.links/miniconda3/envs/aidep/lib/python3.10/site-
packages/sklearn/neural_network/_multilayer_perceptron.py:690:
ConvergenceWarning: Stochastic Optimizer: Maximum iterations (1000) reached and
the optimization hasn't converged yet.
  warnings.warn(
/projects/proteinml/.links/miniconda3/envs/aidep/lib/python3.10/site-
packages/sklearn/neural_network/_multilayer_perceptron.py:690:
ConvergenceWarning: Stochastic Optimizer: Maximum iterations (1000) reached and
the optimization hasn't converged yet.
  warnings.warn(
/projects/proteinml/.links/miniconda3/envs/aidep/lib/python3.10/site-
packages/sklearn/neural_network/_multilayer_perceptron.py:690:
ConvergenceWarning: Stochastic Optimizer: Maximum iterations (1000) reached and
the optimization hasn't converged yet.
  warnings.warn(
/projects/proteinml/.links/miniconda3/envs/aidep/lib/python3.10/site-
packages/sklearn/neural_network/_multilayer_perceptron.py:690:
ConvergenceWarning: Stochastic Optimizer: Maximum iterations (1000) reached and
the optimization hasn't converged yet.
  warnings.warn(
/projects/proteinml/.links/miniconda3/envs/aidep/lib/python3.10/site-
packages/sklearn/neural_network/_multilayer_perceptron.py:690:
ConvergenceWarning: Stochastic Optimizer: Maximum iterations (1000) reached and
the optimization hasn't converged yet.
  warnings.warn(
/projects/proteinml/.links/miniconda3/envs/aidep/lib/python3.10/site-
packages/sklearn/neural_network/_multilayer_perceptron.py:690:
ConvergenceWarning: Stochastic Optimizer: Maximum iterations (1000) reached and
the optimization hasn't converged yet.
  warnings.warn(
/projects/proteinml/.links/miniconda3/envs/aidep/lib/python3.10/site-
packages/sklearn/neural_network/_multilayer_perceptron.py:690:
ConvergenceWarning: Stochastic Optimizer: Maximum iterations (1000) reached and
the optimization hasn't converged yet.
  warnings.warn(
```

```
/projects/proteinml/.links/miniconda3/envs/aidep/lib/python3.10/site-
packages/sklearn/neural_network/_multilayer_perceptron.py:690:
ConvergenceWarning: Stochastic Optimizer: Maximum iterations (1000) reached and
the optimization hasn't converged yet.
  warnings.warn(
/projects/proteinml/.links/miniconda3/envs/aidep/lib/python3.10/site-
packages/sklearn/neural_network/_multilayer_perceptron.py:690:
ConvergenceWarning: Stochastic Optimizer: Maximum iterations (1000) reached and
the optimization hasn't converged yet.
  warnings.warn(
/projects/proteinml/.links/miniconda3/envs/aidep/lib/python3.10/site-
packages/sklearn/neural_network/_multilayer_perceptron.py:690:
ConvergenceWarning: Stochastic Optimizer: Maximum iterations (1000) reached and
the optimization hasn't converged yet.
  warnings.warn(
/projects/proteinml/.links/miniconda3/envs/aidep/lib/python3.10/site-
packages/sklearn/neural_network/_multilayer_perceptron.py:690:
ConvergenceWarning: Stochastic Optimizer: Maximum iterations (1000) reached and
the optimization hasn't converged yet.
  warnings.warn(
/projects/proteinml/.links/miniconda3/envs/aidep/lib/python3.10/site-
packages/sklearn/neural_network/_multilayer_perceptron.py:690:
ConvergenceWarning: Stochastic Optimizer: Maximum iterations (1000) reached and
the optimization hasn't converged yet.
  warnings.warn(
/projects/proteinml/.links/miniconda3/envs/aidep/lib/python3.10/site-
packages/sklearn/neural_network/_multilayer_perceptron.py:690:
ConvergenceWarning: Stochastic Optimizer: Maximum iterations (1000) reached and
the optimization hasn't converged yet.
  warnings.warn(
/projects/proteinml/.links/miniconda3/envs/aidep/lib/python3.10/site-
packages/sklearn/neural_network/_multilayer_perceptron.py:690:
ConvergenceWarning: Stochastic Optimizer: Maximum iterations (1000) reached and
the optimization hasn't converged yet.
  warnings.warn(
/projects/proteinml/.links/miniconda3/envs/aidep/lib/python3.10/site-
packages/sklearn/neural_network/_multilayer_perceptron.py:690:
ConvergenceWarning: Stochastic Optimizer: Maximum iterations (1000) reached and
the optimization hasn't converged yet.
  warnings.warn(
/projects/proteinml/.links/miniconda3/envs/aidep/lib/python3.10/site-
packages/sklearn/neural_network/_multilayer_perceptron.py:690:
ConvergenceWarning: Stochastic Optimizer: Maximum iterations (1000) reached and
the optimization hasn't converged yet.
  warnings.warn(
/projects/proteinml/.links/miniconda3/envs/aidep/lib/python3.10/site-
packages/sklearn/neural_network/_multilayer_perceptron.py:690:
ConvergenceWarning: Stochastic Optimizer: Maximum iterations (1000) reached and
```

```
the optimization hasn't converged yet.
  warnings.warn(
/projects/proteinml/.links/miniconda3/envs/aidep/lib/python3.10/site-
packages/sklearn/neural_network/_multilayer_perceptron.py:690:
ConvergenceWarning: Stochastic Optimizer: Maximum iterations (1000) reached and
the optimization hasn't converged yet.
  warnings.warn(
/projects/proteinml/.links/miniconda3/envs/aidep/lib/python3.10/site-
packages/sklearn/neural_network/_multilayer_perceptron.py:690:
ConvergenceWarning: Stochastic Optimizer: Maximum iterations (1000) reached and
the optimization hasn't converged yet.
  warnings.warn(
/projects/proteinml/.links/miniconda3/envs/aidep/lib/python3.10/site-
packages/sklearn/neural_network/_multilayer_perceptron.py:690:
ConvergenceWarning: Stochastic Optimizer: Maximum iterations (1000) reached and
the optimization hasn't converged yet.
  warnings.warn(
/projects/proteinml/.links/miniconda3/envs/aidep/lib/python3.10/site-
packages/sklearn/neural_network/_multilayer_perceptron.py:690:
ConvergenceWarning: Stochastic Optimizer: Maximum iterations (1000) reached and
the optimization hasn't converged yet.
  warnings.warn(
/projects/proteinml/.links/miniconda3/envs/aidep/lib/python3.10/site-
packages/sklearn/neural_network/_multilayer_perceptron.py:690:
ConvergenceWarning: Stochastic Optimizer: Maximum iterations (1000) reached and
the optimization hasn't converged yet.
  warnings.warn(
/projects/proteinml/.links/miniconda3/envs/aidep/lib/python3.10/site-
packages/sklearn/neural_network/_multilayer_perceptron.py:690:
ConvergenceWarning: Stochastic Optimizer: Maximum iterations (1000) reached and
the optimization hasn't converged yet.
  warnings.warn(
/projects/proteinml/.links/miniconda3/envs/aidep/lib/python3.10/site-
packages/sklearn/neural_network/_multilayer_perceptron.py:690:
ConvergenceWarning: Stochastic Optimizer: Maximum iterations (1000) reached and
the optimization hasn't converged yet.
  warnings.warn(
/projects/proteinml/.links/miniconda3/envs/aidep/lib/python3.10/site-
packages/sklearn/neural_network/_multilayer_perceptron.py:690:
ConvergenceWarning: Stochastic Optimizer: Maximum iterations (1000) reached and
the optimization hasn't converged yet.
  warnings.warn(
/projects/proteinml/.links/miniconda3/envs/aidep/lib/python3.10/site-
packages/sklearn/neural_network/_multilayer_perceptron.py:690:
ConvergenceWarning: Stochastic Optimizer: Maximum iterations (1000) reached and
the optimization hasn't converged yet.
  warnings.warn(
/projects/proteinml/.links/miniconda3/envs/aidep/lib/python3.10/site-
```

```
packages/sklearn/neural_network/_multilayer_perceptron.py:690:
ConvergenceWarning: Stochastic Optimizer: Maximum iterations (1000) reached and
the optimization hasn't converged yet.
  warnings.warn(
/projects/proteinml/.links/miniconda3/envs/aidep/lib/python3.10/site-
packages/sklearn/neural_network/_multilayer_perceptron.py:690:
ConvergenceWarning: Stochastic Optimizer: Maximum iterations (1000) reached and
the optimization hasn't converged yet.
  warnings.warn(
/projects/proteinml/.links/miniconda3/envs/aidep/lib/python3.10/site-
packages/sklearn/neural_network/_multilayer_perceptron.py:690:
ConvergenceWarning: Stochastic Optimizer: Maximum iterations (1000) reached and
the optimization hasn't converged yet.
  warnings.warn(
/projects/proteinml/.links/miniconda3/envs/aidep/lib/python3.10/site-
packages/sklearn/neural_network/_multilayer_perceptron.py:690:
ConvergenceWarning: Stochastic Optimizer: Maximum iterations (1000) reached and
the optimization hasn't converged yet.
  warnings.warn(
/projects/proteinml/.links/miniconda3/envs/aidep/lib/python3.10/site-
packages/sklearn/neural_network/_multilayer_perceptron.py:690:
ConvergenceWarning: Stochastic Optimizer: Maximum iterations (1000) reached and
the optimization hasn't converged yet.
  warnings.warn(
/projects/proteinml/.links/miniconda3/envs/aidep/lib/python3.10/site-
packages/sklearn/neural_network/_multilayer_perceptron.py:690:
ConvergenceWarning: Stochastic Optimizer: Maximum iterations (1000) reached and
the optimization hasn't converged yet.
  warnings.warn(
/projects/proteinml/.links/miniconda3/envs/aidep/lib/python3.10/site-
packages/sklearn/neural_network/_multilayer_perceptron.py:690:
ConvergenceWarning: Stochastic Optimizer: Maximum iterations (1000) reached and
the optimization hasn't converged yet.
  warnings.warn(
/projects/proteinml/.links/miniconda3/envs/aidep/lib/python3.10/site-
packages/sklearn/neural_network/_multilayer_perceptron.py:690:
ConvergenceWarning: Stochastic Optimizer: Maximum iterations (1000) reached and
the optimization hasn't converged yet.
  warnings.warn(
/projects/proteinml/.links/miniconda3/envs/aidep/lib/python3.10/site-
packages/sklearn/neural_network/_multilayer_perceptron.py:690:
ConvergenceWarning: Stochastic Optimizer: Maximum iterations (1000) reached and
the optimization hasn't converged yet.
  warnings.warn(
/projects/proteinml/.links/miniconda3/envs/aidep/lib/python3.10/site-
packages/sklearn/neural_network/_multilayer_perceptron.py:690:
ConvergenceWarning: Stochastic Optimizer: Maximum iterations (1000) reached and
the optimization hasn't converged yet.
```

```
  warnings.warn(
/projects/proteinml/.links/miniconda3/envs/aidep/lib/python3.10/site-
packages/sklearn/neural_network/_multilayer_perceptron.py:690:
ConvergenceWarning: Stochastic Optimizer: Maximum iterations (1000) reached and
the optimization hasn't converged yet.
  warnings.warn(
/projects/proteinml/.links/miniconda3/envs/aidep/lib/python3.10/site-
packages/sklearn/neural_network/_multilayer_perceptron.py:690:
ConvergenceWarning: Stochastic Optimizer: Maximum iterations (1000) reached and
the optimization hasn't converged yet.
  warnings.warn(
/projects/proteinml/.links/miniconda3/envs/aidep/lib/python3.10/site-
packages/sklearn/neural_network/_multilayer_perceptron.py:690:
ConvergenceWarning: Stochastic Optimizer: Maximum iterations (1000) reached and
the optimization hasn't converged yet.
  warnings.warn(
/projects/proteinml/.links/miniconda3/envs/aidep/lib/python3.10/site-
packages/sklearn/neural_network/_multilayer_perceptron.py:690:
ConvergenceWarning: Stochastic Optimizer: Maximum iterations (1000) reached and
the optimization hasn't converged yet.
  warnings.warn(
/projects/proteinml/.links/miniconda3/envs/aidep/lib/python3.10/site-
packages/sklearn/neural_network/_multilayer_perceptron.py:690:
ConvergenceWarning: Stochastic Optimizer: Maximum iterations (1000) reached and
the optimization hasn't converged yet.
  warnings.warn(
/projects/proteinml/.links/miniconda3/envs/aidep/lib/python3.10/site-
packages/sklearn/neural_network/_multilayer_perceptron.py:690:
ConvergenceWarning: Stochastic Optimizer: Maximum iterations (1000) reached and
the optimization hasn't converged yet.
  warnings.warn(
/projects/proteinml/.links/miniconda3/envs/aidep/lib/python3.10/site-
packages/sklearn/neural_network/_multilayer_perceptron.py:690:
ConvergenceWarning: Stochastic Optimizer: Maximum iterations (1000) reached and
the optimization hasn't converged yet.
  warnings.warn(
/projects/proteinml/.links/miniconda3/envs/aidep/lib/python3.10/site-
packages/sklearn/neural_network/_multilayer_perceptron.py:690:
ConvergenceWarning: Stochastic Optimizer: Maximum iterations (1000) reached and
the optimization hasn't converged yet.
  warnings.warn(
/projects/proteinml/.links/miniconda3/envs/aidep/lib/python3.10/site-
packages/sklearn/neural_network/_multilayer_perceptron.py:690:
ConvergenceWarning: Stochastic Optimizer: Maximum iterations (1000) reached and
the optimization hasn't converged yet.
  warnings.warn(
/projects/proteinml/.links/miniconda3/envs/aidep/lib/python3.10/site-
packages/sklearn/neural_network/_multilayer_perceptron.py:690:
```

```
ConvergenceWarning: Stochastic Optimizer: Maximum iterations (1000) reached and
the optimization hasn't converged yet.
  warnings.warn(
/projects/proteinml/.links/miniconda3/envs/aidep/lib/python3.10/site-
packages/sklearn/neural_network/_multilayer_perceptron.py:690:
ConvergenceWarning: Stochastic Optimizer: Maximum iterations (1000) reached and
the optimization hasn't converged yet.
  warnings.warn(
/projects/proteinml/.links/miniconda3/envs/aidep/lib/python3.10/site-
packages/sklearn/neural_network/_multilayer_perceptron.py:690:
ConvergenceWarning: Stochastic Optimizer: Maximum iterations (1000) reached and
the optimization hasn't converged yet.
  warnings.warn(
/projects/proteinml/.links/miniconda3/envs/aidep/lib/python3.10/site-
packages/sklearn/neural_network/_multilayer_perceptron.py:690:
ConvergenceWarning: Stochastic Optimizer: Maximum iterations (1000) reached and
the optimization hasn't converged yet.
  warnings.warn(
/projects/proteinml/.links/miniconda3/envs/aidep/lib/python3.10/site-
packages/sklearn/neural_network/_multilayer_perceptron.py:690:
ConvergenceWarning: Stochastic Optimizer: Maximum iterations (1000) reached and
the optimization hasn't converged yet.
  warnings.warn(
/projects/proteinml/.links/miniconda3/envs/aidep/lib/python3.10/site-
packages/sklearn/neural_network/_multilayer_perceptron.py:690:
ConvergenceWarning: Stochastic Optimizer: Maximum iterations (1000) reached and
the optimization hasn't converged yet.
  warnings.warn(
/projects/proteinml/.links/miniconda3/envs/aidep/lib/python3.10/site-
packages/sklearn/neural_network/_multilayer_perceptron.py:690:
ConvergenceWarning: Stochastic Optimizer: Maximum iterations (1000) reached and
the optimization hasn't converged yet.
  warnings.warn(
/projects/proteinml/.links/miniconda3/envs/aidep/lib/python3.10/site-
packages/sklearn/neural_network/_multilayer_perceptron.py:690:
ConvergenceWarning: Stochastic Optimizer: Maximum iterations (1000) reached and
the optimization hasn't converged yet.
  warnings.warn(
/projects/proteinml/.links/miniconda3/envs/aidep/lib/python3.10/site-
packages/sklearn/neural_network/_multilayer_perceptron.py:690:
ConvergenceWarning: Stochastic Optimizer: Maximum iterations (1000) reached and
the optimization hasn't converged yet.
  warnings.warn(
/projects/proteinml/.links/miniconda3/envs/aidep/lib/python3.10/site-
packages/sklearn/neural_network/_multilayer_perceptron.py:690:
ConvergenceWarning: Stochastic Optimizer: Maximum iterations (1000) reached and
the optimization hasn't converged yet.
  warnings.warn(
```

```
/projects/proteinml/.links/miniconda3/envs/aidep/lib/python3.10/site-
packages/sklearn/neural_network/_multilayer_perceptron.py:690:
ConvergenceWarning: Stochastic Optimizer: Maximum iterations (1000) reached and
the optimization hasn't converged yet.
  warnings.warn(
/projects/proteinml/.links/miniconda3/envs/aidep/lib/python3.10/site-
packages/sklearn/neural_network/_multilayer_perceptron.py:690:
ConvergenceWarning: Stochastic Optimizer: Maximum iterations (1000) reached and
the optimization hasn't converged yet.
  warnings.warn(
/projects/proteinml/.links/miniconda3/envs/aidep/lib/python3.10/site-
packages/sklearn/neural_network/_multilayer_perceptron.py:690:
ConvergenceWarning: Stochastic Optimizer: Maximum iterations (1000) reached and
the optimization hasn't converged yet.
  warnings.warn(
/projects/proteinml/.links/miniconda3/envs/aidep/lib/python3.10/site-
packages/sklearn/neural_network/_multilayer_perceptron.py:690:
ConvergenceWarning: Stochastic Optimizer: Maximum iterations (1000) reached and
the optimization hasn't converged yet.
  warnings.warn(
/projects/proteinml/.links/miniconda3/envs/aidep/lib/python3.10/site-
packages/sklearn/neural_network/_multilayer_perceptron.py:690:
ConvergenceWarning: Stochastic Optimizer: Maximum iterations (1000) reached and
the optimization hasn't converged yet.
  warnings.warn(
/projects/proteinml/.links/miniconda3/envs/aidep/lib/python3.10/site-
packages/sklearn/neural_network/_multilayer_perceptron.py:690:
ConvergenceWarning: Stochastic Optimizer: Maximum iterations (1000) reached and
the optimization hasn't converged yet.
  warnings.warn(
/projects/proteinml/.links/miniconda3/envs/aidep/lib/python3.10/site-
packages/sklearn/neural_network/_multilayer_perceptron.py:690:
ConvergenceWarning: Stochastic Optimizer: Maximum iterations (1000) reached and
the optimization hasn't converged yet.
  warnings.warn(
/projects/proteinml/.links/miniconda3/envs/aidep/lib/python3.10/site-
packages/sklearn/neural_network/_multilayer_perceptron.py:690:
ConvergenceWarning: Stochastic Optimizer: Maximum iterations (1000) reached and
the optimization hasn't converged yet.
  warnings.warn(
/projects/proteinml/.links/miniconda3/envs/aidep/lib/python3.10/site-
packages/sklearn/neural_network/_multilayer_perceptron.py:690:
ConvergenceWarning: Stochastic Optimizer: Maximum iterations (1000) reached and
the optimization hasn't converged yet.
  warnings.warn(
/projects/proteinml/.links/miniconda3/envs/aidep/lib/python3.10/site-
packages/sklearn/neural_network/_multilayer_perceptron.py:690:
ConvergenceWarning: Stochastic Optimizer: Maximum iterations (1000) reached and
```

```
the optimization hasn't converged yet.
  warnings.warn(
/projects/proteinml/.links/miniconda3/envs/aidep/lib/python3.10/site-
packages/sklearn/neural_network/_multilayer_perceptron.py:690:
ConvergenceWarning: Stochastic Optimizer: Maximum iterations (1000) reached and
the optimization hasn't converged yet.
  warnings.warn(
/projects/proteinml/.links/miniconda3/envs/aidep/lib/python3.10/site-
packages/sklearn/neural_network/_multilayer_perceptron.py:690:
ConvergenceWarning: Stochastic Optimizer: Maximum iterations (1000) reached and
the optimization hasn't converged yet.
  warnings.warn(
/projects/proteinml/.links/miniconda3/envs/aidep/lib/python3.10/site-
packages/sklearn/neural_network/_multilayer_perceptron.py:690:
ConvergenceWarning: Stochastic Optimizer: Maximum iterations (1000) reached and
the optimization hasn't converged yet.
  warnings.warn(
/projects/proteinml/.links/miniconda3/envs/aidep/lib/python3.10/site-
packages/sklearn/neural_network/_multilayer_perceptron.py:690:
ConvergenceWarning: Stochastic Optimizer: Maximum iterations (1000) reached and
the optimization hasn't converged yet.
  warnings.warn(
/projects/proteinml/.links/miniconda3/envs/aidep/lib/python3.10/site-
packages/sklearn/neural_network/_multilayer_perceptron.py:690:
ConvergenceWarning: Stochastic Optimizer: Maximum iterations (1000) reached and
the optimization hasn't converged yet.
  warnings.warn(
/projects/proteinml/.links/miniconda3/envs/aidep/lib/python3.10/site-
packages/sklearn/neural_network/_multilayer_perceptron.py:690:
ConvergenceWarning: Stochastic Optimizer: Maximum iterations (1000) reached and
the optimization hasn't converged yet.
  warnings.warn(
/projects/proteinml/.links/miniconda3/envs/aidep/lib/python3.10/site-
packages/sklearn/neural_network/_multilayer_perceptron.py:690:
ConvergenceWarning: Stochastic Optimizer: Maximum iterations (1000) reached and
the optimization hasn't converged yet.
  warnings.warn(
/projects/proteinml/.links/miniconda3/envs/aidep/lib/python3.10/site-
packages/sklearn/neural_network/_multilayer_perceptron.py:690:
ConvergenceWarning: Stochastic Optimizer: Maximum iterations (1000) reached and
the optimization hasn't converged yet.
  warnings.warn(
/projects/proteinml/.links/miniconda3/envs/aidep/lib/python3.10/site-
packages/sklearn/neural_network/_multilayer_perceptron.py:690:
ConvergenceWarning: Stochastic Optimizer: Maximum iterations (1000) reached and
the optimization hasn't converged yet.
  warnings.warn(
/projects/proteinml/.links/miniconda3/envs/aidep/lib/python3.10/site-
```

```
packages/sklearn/neural_network/_multilayer_perceptron.py:690:
ConvergenceWarning: Stochastic Optimizer: Maximum iterations (1000) reached and
the optimization hasn't converged yet.
  warnings.warn(
/projects/proteinml/.links/miniconda3/envs/aidep/lib/python3.10/site-
packages/sklearn/neural_network/_multilayer_perceptron.py:690:
ConvergenceWarning: Stochastic Optimizer: Maximum iterations (1000) reached and
the optimization hasn't converged yet.
  warnings.warn(
/projects/proteinml/.links/miniconda3/envs/aidep/lib/python3.10/site-
packages/sklearn/neural_network/_multilayer_perceptron.py:690:
ConvergenceWarning: Stochastic Optimizer: Maximum iterations (1000) reached and
the optimization hasn't converged yet.
  warnings.warn(
/projects/proteinml/.links/miniconda3/envs/aidep/lib/python3.10/site-
packages/sklearn/neural_network/_multilayer_perceptron.py:690:
ConvergenceWarning: Stochastic Optimizer: Maximum iterations (1000) reached and
the optimization hasn't converged yet.
  warnings.warn(
/projects/proteinml/.links/miniconda3/envs/aidep/lib/python3.10/site-
packages/sklearn/neural_network/_multilayer_perceptron.py:690:
ConvergenceWarning: Stochastic Optimizer: Maximum iterations (1000) reached and
the optimization hasn't converged yet.
  warnings.warn(
/projects/proteinml/.links/miniconda3/envs/aidep/lib/python3.10/site-
packages/sklearn/neural_network/_multilayer_perceptron.py:690:
ConvergenceWarning: Stochastic Optimizer: Maximum iterations (1000) reached and
the optimization hasn't converged yet.
  warnings.warn(
/projects/proteinml/.links/miniconda3/envs/aidep/lib/python3.10/site-
packages/sklearn/neural_network/_multilayer_perceptron.py:690:
ConvergenceWarning: Stochastic Optimizer: Maximum iterations (1000) reached and
the optimization hasn't converged yet.
  warnings.warn(
/projects/proteinml/.links/miniconda3/envs/aidep/lib/python3.10/site-
packages/sklearn/neural_network/_multilayer_perceptron.py:690:
ConvergenceWarning: Stochastic Optimizer: Maximum iterations (1000) reached and
the optimization hasn't converged yet.
  warnings.warn(
/projects/proteinml/.links/miniconda3/envs/aidep/lib/python3.10/site-
packages/sklearn/neural_network/_multilayer_perceptron.py:690:
ConvergenceWarning: Stochastic Optimizer: Maximum iterations (1000) reached and
the optimization hasn't converged yet.
  warnings.warn(
/projects/proteinml/.links/miniconda3/envs/aidep/lib/python3.10/site-
packages/sklearn/neural_network/_multilayer_perceptron.py:690:
ConvergenceWarning: Stochastic Optimizer: Maximum iterations (1000) reached and
the optimization hasn't converged yet.
```

```
  warnings.warn(
/projects/proteinml/.links/miniconda3/envs/aidep/lib/python3.10/site-
packages/sklearn/neural_network/_multilayer_perceptron.py:690:
ConvergenceWarning: Stochastic Optimizer: Maximum iterations (1000) reached and
the optimization hasn't converged yet.
  warnings.warn(
/projects/proteinml/.links/miniconda3/envs/aidep/lib/python3.10/site-
packages/sklearn/neural_network/_multilayer_perceptron.py:690:
ConvergenceWarning: Stochastic Optimizer: Maximum iterations (1000) reached and
the optimization hasn't converged yet.
  warnings.warn(
/projects/proteinml/.links/miniconda3/envs/aidep/lib/python3.10/site-
packages/sklearn/neural_network/_multilayer_perceptron.py:690:
ConvergenceWarning: Stochastic Optimizer: Maximum iterations (1000) reached and
the optimization hasn't converged yet.
  warnings.warn(
/projects/proteinml/.links/miniconda3/envs/aidep/lib/python3.10/site-
packages/sklearn/neural_network/_multilayer_perceptron.py:690:
ConvergenceWarning: Stochastic Optimizer: Maximum iterations (1000) reached and
the optimization hasn't converged yet.
  warnings.warn(
/projects/proteinml/.links/miniconda3/envs/aidep/lib/python3.10/site-
packages/sklearn/neural_network/_multilayer_perceptron.py:690:
ConvergenceWarning: Stochastic Optimizer: Maximum iterations (1000) reached and
the optimization hasn't converged yet.
  warnings.warn(
/projects/proteinml/.links/miniconda3/envs/aidep/lib/python3.10/site-
packages/sklearn/neural_network/_multilayer_perceptron.py:690:
ConvergenceWarning: Stochastic Optimizer: Maximum iterations (1000) reached and
the optimization hasn't converged yet.
  warnings.warn(
/projects/proteinml/.links/miniconda3/envs/aidep/lib/python3.10/site-
packages/sklearn/neural_network/_multilayer_perceptron.py:690:
ConvergenceWarning: Stochastic Optimizer: Maximum iterations (1000) reached and
the optimization hasn't converged yet.
  warnings.warn(
/projects/proteinml/.links/miniconda3/envs/aidep/lib/python3.10/site-
packages/sklearn/neural_network/_multilayer_perceptron.py:690:
ConvergenceWarning: Stochastic Optimizer: Maximum iterations (1000) reached and
the optimization hasn't converged yet.
  warnings.warn(
/projects/proteinml/.links/miniconda3/envs/aidep/lib/python3.10/site-
packages/sklearn/neural_network/_multilayer_perceptron.py:690:
ConvergenceWarning: Stochastic Optimizer: Maximum iterations (1000) reached and
the optimization hasn't converged yet.
  warnings.warn(
/projects/proteinml/.links/miniconda3/envs/aidep/lib/python3.10/site-
packages/sklearn/neural_network/_multilayer_perceptron.py:690:
```

```
ConvergenceWarning: Stochastic Optimizer: Maximum iterations (1000) reached and
the optimization hasn't converged yet.
  warnings.warn(
/projects/proteinml/.links/miniconda3/envs/aidep/lib/python3.10/site-
packages/sklearn/neural_network/_multilayer_perceptron.py:690:
ConvergenceWarning: Stochastic Optimizer: Maximum iterations (1000) reached and
the optimization hasn't converged yet.
  warnings.warn(
/projects/proteinml/.links/miniconda3/envs/aidep/lib/python3.10/site-
packages/sklearn/neural_network/_multilayer_perceptron.py:690:
ConvergenceWarning: Stochastic Optimizer: Maximum iterations (1000) reached and
the optimization hasn't converged yet.
  warnings.warn(
/projects/proteinml/.links/miniconda3/envs/aidep/lib/python3.10/site-
packages/sklearn/neural_network/_multilayer_perceptron.py:690:
ConvergenceWarning: Stochastic Optimizer: Maximum iterations (1000) reached and
the optimization hasn't converged yet.
  warnings.warn(
/projects/proteinml/.links/miniconda3/envs/aidep/lib/python3.10/site-
packages/sklearn/neural_network/_multilayer_perceptron.py:690:
ConvergenceWarning: Stochastic Optimizer: Maximum iterations (1000) reached and
the optimization hasn't converged yet.
  warnings.warn(
/projects/proteinml/.links/miniconda3/envs/aidep/lib/python3.10/site-
packages/sklearn/neural_network/_multilayer_perceptron.py:690:
ConvergenceWarning: Stochastic Optimizer: Maximum iterations (1000) reached and
the optimization hasn't converged yet.
  warnings.warn(
/projects/proteinml/.links/miniconda3/envs/aidep/lib/python3.10/site-
packages/sklearn/neural_network/_multilayer_perceptron.py:690:
ConvergenceWarning: Stochastic Optimizer: Maximum iterations (1000) reached and
the optimization hasn't converged yet.
  warnings.warn(
/projects/proteinml/.links/miniconda3/envs/aidep/lib/python3.10/site-
packages/sklearn/neural_network/_multilayer_perceptron.py:690:
ConvergenceWarning: Stochastic Optimizer: Maximum iterations (1000) reached and
the optimization hasn't converged yet.
  warnings.warn(
/projects/proteinml/.links/miniconda3/envs/aidep/lib/python3.10/site-
packages/sklearn/neural_network/_multilayer_perceptron.py:690:
ConvergenceWarning: Stochastic Optimizer: Maximum iterations (1000) reached and
the optimization hasn't converged yet.
  warnings.warn(
/projects/proteinml/.links/miniconda3/envs/aidep/lib/python3.10/site-
packages/sklearn/neural_network/_multilayer_perceptron.py:690:
ConvergenceWarning: Stochastic Optimizer: Maximum iterations (1000) reached and
the optimization hasn't converged yet.
  warnings.warn(
```

```
/projects/proteinml/.links/miniconda3/envs/aidep/lib/python3.10/site-
packages/sklearn/neural_network/_multilayer_perceptron.py:690:
ConvergenceWarning: Stochastic Optimizer: Maximum iterations (1000) reached and
the optimization hasn't converged yet.
  warnings.warn(
/projects/proteinml/.links/miniconda3/envs/aidep/lib/python3.10/site-
packages/sklearn/neural_network/_multilayer_perceptron.py:690:
ConvergenceWarning: Stochastic Optimizer: Maximum iterations (1000) reached and
the optimization hasn't converged yet.
  warnings.warn(
/projects/proteinml/.links/miniconda3/envs/aidep/lib/python3.10/site-
packages/sklearn/neural_network/_multilayer_perceptron.py:690:
ConvergenceWarning: Stochastic Optimizer: Maximum iterations (1000) reached and
the optimization hasn't converged yet.
  warnings.warn(
/projects/proteinml/.links/miniconda3/envs/aidep/lib/python3.10/site-
packages/sklearn/neural_network/_multilayer_perceptron.py:690:
ConvergenceWarning: Stochastic Optimizer: Maximum iterations (1000) reached and
the optimization hasn't converged yet.
  warnings.warn(
/projects/proteinml/.links/miniconda3/envs/aidep/lib/python3.10/site-
packages/sklearn/neural_network/_multilayer_perceptron.py:690:
ConvergenceWarning: Stochastic Optimizer: Maximum iterations (1000) reached and
the optimization hasn't converged yet.
  warnings.warn(
/projects/proteinml/.links/miniconda3/envs/aidep/lib/python3.10/site-
packages/sklearn/neural_network/_multilayer_perceptron.py:690:
ConvergenceWarning: Stochastic Optimizer: Maximum iterations (1000) reached and
the optimization hasn't converged yet.
  warnings.warn(
/projects/proteinml/.links/miniconda3/envs/aidep/lib/python3.10/site-
packages/sklearn/neural_network/_multilayer_perceptron.py:690:
ConvergenceWarning: Stochastic Optimizer: Maximum iterations (1000) reached and
the optimization hasn't converged yet.
  warnings.warn(
/projects/proteinml/.links/miniconda3/envs/aidep/lib/python3.10/site-
packages/sklearn/neural_network/_multilayer_perceptron.py:690:
ConvergenceWarning: Stochastic Optimizer: Maximum iterations (1000) reached and
the optimization hasn't converged yet.
  warnings.warn(
/projects/proteinml/.links/miniconda3/envs/aidep/lib/python3.10/site-
packages/sklearn/neural_network/_multilayer_perceptron.py:690:
ConvergenceWarning: Stochastic Optimizer: Maximum iterations (1000) reached and
the optimization hasn't converged yet.
  warnings.warn(
/projects/proteinml/.links/miniconda3/envs/aidep/lib/python3.10/site-
packages/sklearn/neural_network/_multilayer_perceptron.py:690:
ConvergenceWarning: Stochastic Optimizer: Maximum iterations (1000) reached and
```

```
the optimization hasn't converged yet.
  warnings.warn(
/projects/proteinml/.links/miniconda3/envs/aidep/lib/python3.10/site-
packages/sklearn/neural_network/_multilayer_perceptron.py:690:
ConvergenceWarning: Stochastic Optimizer: Maximum iterations (1000) reached and
the optimization hasn't converged yet.
  warnings.warn(
/projects/proteinml/.links/miniconda3/envs/aidep/lib/python3.10/site-
packages/sklearn/neural_network/_multilayer_perceptron.py:690:
ConvergenceWarning: Stochastic Optimizer: Maximum iterations (1000) reached and
the optimization hasn't converged yet.
  warnings.warn(
/projects/proteinml/.links/miniconda3/envs/aidep/lib/python3.10/site-
packages/sklearn/neural_network/_multilayer_perceptron.py:690:
ConvergenceWarning: Stochastic Optimizer: Maximum iterations (1000) reached and
the optimization hasn't converged yet.
  warnings.warn(
/projects/proteinml/.links/miniconda3/envs/aidep/lib/python3.10/site-
packages/sklearn/neural_network/_multilayer_perceptron.py:690:
ConvergenceWarning: Stochastic Optimizer: Maximum iterations (1000) reached and
the optimization hasn't converged yet.
  warnings.warn(
/projects/proteinml/.links/miniconda3/envs/aidep/lib/python3.10/site-
packages/sklearn/neural_network/_multilayer_perceptron.py:690:
ConvergenceWarning: Stochastic Optimizer: Maximum iterations (1000) reached and
the optimization hasn't converged yet.
  warnings.warn(
/projects/proteinml/.links/miniconda3/envs/aidep/lib/python3.10/site-
packages/sklearn/neural_network/_multilayer_perceptron.py:690:
ConvergenceWarning: Stochastic Optimizer: Maximum iterations (1000) reached and
the optimization hasn't converged yet.
  warnings.warn(
/projects/proteinml/.links/miniconda3/envs/aidep/lib/python3.10/site-
packages/sklearn/neural_network/_multilayer_perceptron.py:690:
ConvergenceWarning: Stochastic Optimizer: Maximum iterations (1000) reached and
the optimization hasn't converged yet.
  warnings.warn(
/projects/proteinml/.links/miniconda3/envs/aidep/lib/python3.10/site-
packages/sklearn/neural_network/_multilayer_perceptron.py:690:
ConvergenceWarning: Stochastic Optimizer: Maximum iterations (1000) reached and
the optimization hasn't converged yet.
  warnings.warn(
/projects/proteinml/.links/miniconda3/envs/aidep/lib/python3.10/site-
packages/sklearn/neural_network/_multilayer_perceptron.py:690:
ConvergenceWarning: Stochastic Optimizer: Maximum iterations (1000) reached and
the optimization hasn't converged yet.
  warnings.warn(
/projects/proteinml/.links/miniconda3/envs/aidep/lib/python3.10/site-
```

```
packages/sklearn/neural_network/_multilayer_perceptron.py:690:
ConvergenceWarning: Stochastic Optimizer: Maximum iterations (1000) reached and
the optimization hasn't converged yet.
  warnings.warn(
/projects/proteinml/.links/miniconda3/envs/aidep/lib/python3.10/site-
packages/sklearn/neural_network/_multilayer_perceptron.py:690:
ConvergenceWarning: Stochastic Optimizer: Maximum iterations (1000) reached and
the optimization hasn't converged yet.
  warnings.warn(
/projects/proteinml/.links/miniconda3/envs/aidep/lib/python3.10/site-
packages/sklearn/neural_network/_multilayer_perceptron.py:690:
ConvergenceWarning: Stochastic Optimizer: Maximum iterations (1000) reached and
the optimization hasn't converged yet.
  warnings.warn(
/projects/proteinml/.links/miniconda3/envs/aidep/lib/python3.10/site-
packages/sklearn/neural_network/_multilayer_perceptron.py:690:
ConvergenceWarning: Stochastic Optimizer: Maximum iterations (1000) reached and
the optimization hasn't converged yet.
  warnings.warn(
/projects/proteinml/.links/miniconda3/envs/aidep/lib/python3.10/site-
packages/sklearn/neural_network/_multilayer_perceptron.py:690:
ConvergenceWarning: Stochastic Optimizer: Maximum iterations (1000) reached and
the optimization hasn't converged yet.
  warnings.warn(
/projects/proteinml/.links/miniconda3/envs/aidep/lib/python3.10/site-
packages/sklearn/neural_network/_multilayer_perceptron.py:690:
ConvergenceWarning: Stochastic Optimizer: Maximum iterations (1000) reached and
the optimization hasn't converged yet.
  warnings.warn(
/projects/proteinml/.links/miniconda3/envs/aidep/lib/python3.10/site-
packages/sklearn/neural_network/_multilayer_perceptron.py:690:
ConvergenceWarning: Stochastic Optimizer: Maximum iterations (1000) reached and
the optimization hasn't converged yet.
  warnings.warn(
/projects/proteinml/.links/miniconda3/envs/aidep/lib/python3.10/site-
packages/sklearn/neural_network/_multilayer_perceptron.py:690:
ConvergenceWarning: Stochastic Optimizer: Maximum iterations (1000) reached and
the optimization hasn't converged yet.
  warnings.warn(
/projects/proteinml/.links/miniconda3/envs/aidep/lib/python3.10/site-
packages/sklearn/neural_network/_multilayer_perceptron.py:690:
ConvergenceWarning: Stochastic Optimizer: Maximum iterations (1000) reached and
the optimization hasn't converged yet.
  warnings.warn(
/projects/proteinml/.links/miniconda3/envs/aidep/lib/python3.10/site-
packages/sklearn/neural_network/_multilayer_perceptron.py:690:
ConvergenceWarning: Stochastic Optimizer: Maximum iterations (1000) reached and
the optimization hasn't converged yet.
```

```
  warnings.warn(
/projects/proteinml/.links/miniconda3/envs/aidep/lib/python3.10/site-
packages/sklearn/neural_network/_multilayer_perceptron.py:690:
ConvergenceWarning: Stochastic Optimizer: Maximum iterations (1000) reached and
the optimization hasn't converged yet.
  warnings.warn(
/projects/proteinml/.links/miniconda3/envs/aidep/lib/python3.10/site-
packages/sklearn/neural_network/_multilayer_perceptron.py:690:
ConvergenceWarning: Stochastic Optimizer: Maximum iterations (1000) reached and
the optimization hasn't converged yet.
  warnings.warn(
/projects/proteinml/.links/miniconda3/envs/aidep/lib/python3.10/site-
packages/sklearn/neural_network/_multilayer_perceptron.py:690:
ConvergenceWarning: Stochastic Optimizer: Maximum iterations (1000) reached and
the optimization hasn't converged yet.
  warnings.warn(
/projects/proteinml/.links/miniconda3/envs/aidep/lib/python3.10/site-
packages/sklearn/neural_network/_multilayer_perceptron.py:690:
ConvergenceWarning: Stochastic Optimizer: Maximum iterations (1000) reached and
the optimization hasn't converged yet.
  warnings.warn(
/projects/proteinml/.links/miniconda3/envs/aidep/lib/python3.10/site-
packages/sklearn/neural_network/_multilayer_perceptron.py:690:
ConvergenceWarning: Stochastic Optimizer: Maximum iterations (1000) reached and
the optimization hasn't converged yet.
  warnings.warn(
/projects/proteinml/.links/miniconda3/envs/aidep/lib/python3.10/site-
packages/sklearn/neural_network/_multilayer_perceptron.py:690:
ConvergenceWarning: Stochastic Optimizer: Maximum iterations (1000) reached and
the optimization hasn't converged yet.
  warnings.warn(
/projects/proteinml/.links/miniconda3/envs/aidep/lib/python3.10/site-
packages/sklearn/neural_network/_multilayer_perceptron.py:690:
ConvergenceWarning: Stochastic Optimizer: Maximum iterations (1000) reached and
the optimization hasn't converged yet.
  warnings.warn(
/projects/proteinml/.links/miniconda3/envs/aidep/lib/python3.10/site-
packages/sklearn/neural_network/_multilayer_perceptron.py:690:
ConvergenceWarning: Stochastic Optimizer: Maximum iterations (1000) reached and
the optimization hasn't converged yet.
  warnings.warn(
/projects/proteinml/.links/miniconda3/envs/aidep/lib/python3.10/site-
packages/sklearn/neural_network/_multilayer_perceptron.py:690:
ConvergenceWarning: Stochastic Optimizer: Maximum iterations (1000) reached and
the optimization hasn't converged yet.
  warnings.warn(
/projects/proteinml/.links/miniconda3/envs/aidep/lib/python3.10/site-
packages/sklearn/neural_network/_multilayer_perceptron.py:690:
```

```
ConvergenceWarning: Stochastic Optimizer: Maximum iterations (1000) reached and
the optimization hasn't converged yet.
  warnings.warn(
/projects/proteinml/.links/miniconda3/envs/aidep/lib/python3.10/site-
packages/sklearn/neural_network/_multilayer_perceptron.py:690:
ConvergenceWarning: Stochastic Optimizer: Maximum iterations (1000) reached and
the optimization hasn't converged yet.
  warnings.warn(
/projects/proteinml/.links/miniconda3/envs/aidep/lib/python3.10/site-
packages/sklearn/neural_network/_multilayer_perceptron.py:690:
ConvergenceWarning: Stochastic Optimizer: Maximum iterations (1000) reached and
the optimization hasn't converged yet.
  warnings.warn(
/projects/proteinml/.links/miniconda3/envs/aidep/lib/python3.10/site-
packages/sklearn/neural_network/_multilayer_perceptron.py:690:
ConvergenceWarning: Stochastic Optimizer: Maximum iterations (1000) reached and
the optimization hasn't converged yet.
  warnings.warn(
/projects/proteinml/.links/miniconda3/envs/aidep/lib/python3.10/site-
packages/sklearn/neural_network/_multilayer_perceptron.py:690:
ConvergenceWarning: Stochastic Optimizer: Maximum iterations (1000) reached and
the optimization hasn't converged yet.
  warnings.warn(
/projects/proteinml/.links/miniconda3/envs/aidep/lib/python3.10/site-
packages/sklearn/neural_network/_multilayer_perceptron.py:690:
ConvergenceWarning: Stochastic Optimizer: Maximum iterations (1000) reached and
the optimization hasn't converged yet.
  warnings.warn(
/projects/proteinml/.links/miniconda3/envs/aidep/lib/python3.10/site-
packages/sklearn/neural_network/_multilayer_perceptron.py:690:
ConvergenceWarning: Stochastic Optimizer: Maximum iterations (1000) reached and
the optimization hasn't converged yet.
  warnings.warn(
/projects/proteinml/.links/miniconda3/envs/aidep/lib/python3.10/site-
packages/sklearn/neural_network/_multilayer_perceptron.py:690:
ConvergenceWarning: Stochastic Optimizer: Maximum iterations (1000) reached and
the optimization hasn't converged yet.
  warnings.warn(
/projects/proteinml/.links/miniconda3/envs/aidep/lib/python3.10/site-
packages/sklearn/neural_network/_multilayer_perceptron.py:690:
ConvergenceWarning: Stochastic Optimizer: Maximum iterations (1000) reached and
the optimization hasn't converged yet.
  warnings.warn(
/projects/proteinml/.links/miniconda3/envs/aidep/lib/python3.10/site-
packages/sklearn/neural_network/_multilayer_perceptron.py:690:
ConvergenceWarning: Stochastic Optimizer: Maximum iterations (1000) reached and
the optimization hasn't converged yet.
  warnings.warn(
```

```
/projects/proteinml/.links/miniconda3/envs/aidep/lib/python3.10/site-
packages/sklearn/neural_network/_multilayer_perceptron.py:690:
ConvergenceWarning: Stochastic Optimizer: Maximum iterations (1000) reached and
the optimization hasn't converged yet.
  warnings.warn(
/projects/proteinml/.links/miniconda3/envs/aidep/lib/python3.10/site-
packages/sklearn/neural_network/_multilayer_perceptron.py:690:
ConvergenceWarning: Stochastic Optimizer: Maximum iterations (1000) reached and
the optimization hasn't converged yet.
  warnings.warn(
/projects/proteinml/.links/miniconda3/envs/aidep/lib/python3.10/site-
packages/sklearn/neural_network/_multilayer_perceptron.py:690:
ConvergenceWarning: Stochastic Optimizer: Maximum iterations (1000) reached and
the optimization hasn't converged yet.
  warnings.warn(
/projects/proteinml/.links/miniconda3/envs/aidep/lib/python3.10/site-
packages/sklearn/neural_network/_multilayer_perceptron.py:690:
ConvergenceWarning: Stochastic Optimizer: Maximum iterations (1000) reached and
the optimization hasn't converged yet.
  warnings.warn(
/projects/proteinml/.links/miniconda3/envs/aidep/lib/python3.10/site-
packages/sklearn/neural_network/_multilayer_perceptron.py:690:
ConvergenceWarning: Stochastic Optimizer: Maximum iterations (1000) reached and
the optimization hasn't converged yet.
  warnings.warn(
/projects/proteinml/.links/miniconda3/envs/aidep/lib/python3.10/site-
packages/sklearn/neural_network/_multilayer_perceptron.py:690:
ConvergenceWarning: Stochastic Optimizer: Maximum iterations (1000) reached and
the optimization hasn't converged yet.
  warnings.warn(
/projects/proteinml/.links/miniconda3/envs/aidep/lib/python3.10/site-
packages/sklearn/neural_network/_multilayer_perceptron.py:690:
ConvergenceWarning: Stochastic Optimizer: Maximum iterations (1000) reached and
the optimization hasn't converged yet.
  warnings.warn(
/projects/proteinml/.links/miniconda3/envs/aidep/lib/python3.10/site-
packages/sklearn/neural_network/_multilayer_perceptron.py:690:
ConvergenceWarning: Stochastic Optimizer: Maximum iterations (1000) reached and
the optimization hasn't converged yet.
  warnings.warn(
/projects/proteinml/.links/miniconda3/envs/aidep/lib/python3.10/site-
packages/sklearn/neural_network/_multilayer_perceptron.py:690:
ConvergenceWarning: Stochastic Optimizer: Maximum iterations (1000) reached and
the optimization hasn't converged yet.
  warnings.warn(
/projects/proteinml/.links/miniconda3/envs/aidep/lib/python3.10/site-
packages/sklearn/neural_network/_multilayer_perceptron.py:690:
ConvergenceWarning: Stochastic Optimizer: Maximum iterations (1000) reached and
```

```
the optimization hasn't converged yet.
  warnings.warn(
/projects/proteinml/.links/miniconda3/envs/aidep/lib/python3.10/site-
packages/sklearn/neural_network/_multilayer_perceptron.py:690:
ConvergenceWarning: Stochastic Optimizer: Maximum iterations (1000) reached and
the optimization hasn't converged yet.
  warnings.warn(
/projects/proteinml/.links/miniconda3/envs/aidep/lib/python3.10/site-
packages/sklearn/neural_network/_multilayer_perceptron.py:690:
ConvergenceWarning: Stochastic Optimizer: Maximum iterations (1000) reached and
the optimization hasn't converged yet.
  warnings.warn(
/projects/proteinml/.links/miniconda3/envs/aidep/lib/python3.10/site-
packages/sklearn/neural_network/_multilayer_perceptron.py:690:
ConvergenceWarning: Stochastic Optimizer: Maximum iterations (1000) reached and
the optimization hasn't converged yet.
  warnings.warn(
/projects/proteinml/.links/miniconda3/envs/aidep/lib/python3.10/site-
packages/sklearn/neural_network/_multilayer_perceptron.py:690:
ConvergenceWarning: Stochastic Optimizer: Maximum iterations (1000) reached and
the optimization hasn't converged yet.
  warnings.warn(
/projects/proteinml/.links/miniconda3/envs/aidep/lib/python3.10/site-
packages/sklearn/neural_network/_multilayer_perceptron.py:690:
ConvergenceWarning: Stochastic Optimizer: Maximum iterations (1000) reached and
the optimization hasn't converged yet.
  warnings.warn(
/projects/proteinml/.links/miniconda3/envs/aidep/lib/python3.10/site-
packages/sklearn/neural_network/_multilayer_perceptron.py:690:
ConvergenceWarning: Stochastic Optimizer: Maximum iterations (1000) reached and
the optimization hasn't converged yet.
  warnings.warn(
/projects/proteinml/.links/miniconda3/envs/aidep/lib/python3.10/site-
packages/sklearn/neural_network/_multilayer_perceptron.py:690:
ConvergenceWarning: Stochastic Optimizer: Maximum iterations (1000) reached and
the optimization hasn't converged yet.
  warnings.warn(
/projects/proteinml/.links/miniconda3/envs/aidep/lib/python3.10/site-
packages/sklearn/neural_network/_multilayer_perceptron.py:690:
ConvergenceWarning: Stochastic Optimizer: Maximum iterations (1000) reached and
the optimization hasn't converged yet.
  warnings.warn(
/projects/proteinml/.links/miniconda3/envs/aidep/lib/python3.10/site-
packages/sklearn/neural_network/_multilayer_perceptron.py:690:
ConvergenceWarning: Stochastic Optimizer: Maximum iterations (1000) reached and
the optimization hasn't converged yet.
  warnings.warn(
/projects/proteinml/.links/miniconda3/envs/aidep/lib/python3.10/site-
```

```
packages/sklearn/neural_network/_multilayer_perceptron.py:690:
ConvergenceWarning: Stochastic Optimizer: Maximum iterations (1000) reached and
the optimization hasn't converged yet.
  warnings.warn(
/projects/proteinml/.links/miniconda3/envs/aidep/lib/python3.10/site-
packages/sklearn/neural_network/_multilayer_perceptron.py:690:
ConvergenceWarning: Stochastic Optimizer: Maximum iterations (1000) reached and
the optimization hasn't converged yet.
  warnings.warn(
/projects/proteinml/.links/miniconda3/envs/aidep/lib/python3.10/site-
packages/sklearn/neural_network/_multilayer_perceptron.py:690:
ConvergenceWarning: Stochastic Optimizer: Maximum iterations (1000) reached and
the optimization hasn't converged yet.
  warnings.warn(
/projects/proteinml/.links/miniconda3/envs/aidep/lib/python3.10/site-
packages/sklearn/neural_network/_multilayer_perceptron.py:690:
ConvergenceWarning: Stochastic Optimizer: Maximum iterations (1000) reached and
the optimization hasn't converged yet.
  warnings.warn(
/projects/proteinml/.links/miniconda3/envs/aidep/lib/python3.10/site-
packages/sklearn/neural_network/_multilayer_perceptron.py:690:
ConvergenceWarning: Stochastic Optimizer: Maximum iterations (1000) reached and
the optimization hasn't converged yet.
  warnings.warn(
/projects/proteinml/.links/miniconda3/envs/aidep/lib/python3.10/site-
packages/sklearn/neural_network/_multilayer_perceptron.py:690:
ConvergenceWarning: Stochastic Optimizer: Maximum iterations (1000) reached and
the optimization hasn't converged yet.
  warnings.warn(
/projects/proteinml/.links/miniconda3/envs/aidep/lib/python3.10/site-
packages/sklearn/neural_network/_multilayer_perceptron.py:690:
ConvergenceWarning: Stochastic Optimizer: Maximum iterations (1000) reached and
the optimization hasn't converged yet.
  warnings.warn(
/projects/proteinml/.links/miniconda3/envs/aidep/lib/python3.10/site-
packages/sklearn/neural_network/_multilayer_perceptron.py:690:
ConvergenceWarning: Stochastic Optimizer: Maximum iterations (1000) reached and
the optimization hasn't converged yet.
  warnings.warn(
/projects/proteinml/.links/miniconda3/envs/aidep/lib/python3.10/site-
packages/sklearn/neural_network/_multilayer_perceptron.py:690:
ConvergenceWarning: Stochastic Optimizer: Maximum iterations (1000) reached and
the optimization hasn't converged yet.
  warnings.warn(
/projects/proteinml/.links/miniconda3/envs/aidep/lib/python3.10/site-
packages/sklearn/neural_network/_multilayer_perceptron.py:690:
ConvergenceWarning: Stochastic Optimizer: Maximum iterations (1000) reached and
the optimization hasn't converged yet.
```

```
  warnings.warn(
/projects/proteinml/.links/miniconda3/envs/aidep/lib/python3.10/site-
packages/sklearn/neural_network/_multilayer_perceptron.py:690:
ConvergenceWarning: Stochastic Optimizer: Maximum iterations (1000) reached and
the optimization hasn't converged yet.
  warnings.warn(
/projects/proteinml/.links/miniconda3/envs/aidep/lib/python3.10/site-
packages/sklearn/neural_network/_multilayer_perceptron.py:690:
ConvergenceWarning: Stochastic Optimizer: Maximum iterations (1000) reached and
the optimization hasn't converged yet.
  warnings.warn(
/projects/proteinml/.links/miniconda3/envs/aidep/lib/python3.10/site-
packages/sklearn/neural_network/_multilayer_perceptron.py:690:
ConvergenceWarning: Stochastic Optimizer: Maximum iterations (1000) reached and
the optimization hasn't converged yet.
  warnings.warn(
/projects/proteinml/.links/miniconda3/envs/aidep/lib/python3.10/site-
packages/sklearn/neural_network/_multilayer_perceptron.py:690:
ConvergenceWarning: Stochastic Optimizer: Maximum iterations (1000) reached and
the optimization hasn't converged yet.
  warnings.warn(
/projects/proteinml/.links/miniconda3/envs/aidep/lib/python3.10/site-
packages/sklearn/neural_network/_multilayer_perceptron.py:690:
ConvergenceWarning: Stochastic Optimizer: Maximum iterations (1000) reached and
the optimization hasn't converged yet.
  warnings.warn(
/projects/proteinml/.links/miniconda3/envs/aidep/lib/python3.10/site-
packages/sklearn/neural_network/_multilayer_perceptron.py:690:
ConvergenceWarning: Stochastic Optimizer: Maximum iterations (1000) reached and
the optimization hasn't converged yet.
  warnings.warn(
/projects/proteinml/.links/miniconda3/envs/aidep/lib/python3.10/site-
packages/sklearn/neural_network/_multilayer_perceptron.py:690:
ConvergenceWarning: Stochastic Optimizer: Maximum iterations (1000) reached and
the optimization hasn't converged yet.
  warnings.warn(
/projects/proteinml/.links/miniconda3/envs/aidep/lib/python3.10/site-
packages/sklearn/neural_network/_multilayer_perceptron.py:690:
ConvergenceWarning: Stochastic Optimizer: Maximum iterations (1000) reached and
the optimization hasn't converged yet.
  warnings.warn(
/projects/proteinml/.links/miniconda3/envs/aidep/lib/python3.10/site-
packages/sklearn/neural_network/_multilayer_perceptron.py:690:
ConvergenceWarning: Stochastic Optimizer: Maximum iterations (1000) reached and
the optimization hasn't converged yet.
  warnings.warn(
/projects/proteinml/.links/miniconda3/envs/aidep/lib/python3.10/site-
packages/sklearn/neural_network/_multilayer_perceptron.py:690:
```

```
ConvergenceWarning: Stochastic Optimizer: Maximum iterations (1000) reached and
the optimization hasn't converged yet.
  warnings.warn(
/projects/proteinml/.links/miniconda3/envs/aidep/lib/python3.10/site-
packages/sklearn/neural_network/_multilayer_perceptron.py:690:
ConvergenceWarning: Stochastic Optimizer: Maximum iterations (1000) reached and
the optimization hasn't converged yet.
  warnings.warn(
/projects/proteinml/.links/miniconda3/envs/aidep/lib/python3.10/site-
packages/sklearn/neural_network/_multilayer_perceptron.py:690:
ConvergenceWarning: Stochastic Optimizer: Maximum iterations (1000) reached and
the optimization hasn't converged yet.
  warnings.warn(
/projects/proteinml/.links/miniconda3/envs/aidep/lib/python3.10/site-
packages/sklearn/neural_network/_multilayer_perceptron.py:690:
ConvergenceWarning: Stochastic Optimizer: Maximum iterations (1000) reached and
the optimization hasn't converged yet.
  warnings.warn(
/projects/proteinml/.links/miniconda3/envs/aidep/lib/python3.10/site-
packages/sklearn/neural_network/_multilayer_perceptron.py:690:
ConvergenceWarning: Stochastic Optimizer: Maximum iterations (1000) reached and
the optimization hasn't converged yet.
  warnings.warn(
/projects/proteinml/.links/miniconda3/envs/aidep/lib/python3.10/site-
packages/sklearn/neural_network/_multilayer_perceptron.py:690:
ConvergenceWarning: Stochastic Optimizer: Maximum iterations (1000) reached and
the optimization hasn't converged yet.
  warnings.warn(
/projects/proteinml/.links/miniconda3/envs/aidep/lib/python3.10/site-
packages/sklearn/neural_network/_multilayer_perceptron.py:690:
ConvergenceWarning: Stochastic Optimizer: Maximum iterations (1000) reached and
the optimization hasn't converged yet.
  warnings.warn(
/projects/proteinml/.links/miniconda3/envs/aidep/lib/python3.10/site-
packages/sklearn/neural_network/_multilayer_perceptron.py:690:
ConvergenceWarning: Stochastic Optimizer: Maximum iterations (1000) reached and
the optimization hasn't converged yet.
  warnings.warn(
/projects/proteinml/.links/miniconda3/envs/aidep/lib/python3.10/site-
packages/sklearn/neural_network/_multilayer_perceptron.py:690:
ConvergenceWarning: Stochastic Optimizer: Maximum iterations (1000) reached and
the optimization hasn't converged yet.
  warnings.warn(
/projects/proteinml/.links/miniconda3/envs/aidep/lib/python3.10/site-
packages/sklearn/neural_network/_multilayer_perceptron.py:690:
ConvergenceWarning: Stochastic Optimizer: Maximum iterations (1000) reached and
the optimization hasn't converged yet.
  warnings.warn(
```

```
/projects/proteinml/.links/miniconda3/envs/aidep/lib/python3.10/site-
packages/sklearn/neural_network/_multilayer_perceptron.py:690:
ConvergenceWarning: Stochastic Optimizer: Maximum iterations (1000) reached and
the optimization hasn't converged yet.
  warnings.warn(
/projects/proteinml/.links/miniconda3/envs/aidep/lib/python3.10/site-
packages/sklearn/neural_network/_multilayer_perceptron.py:690:
ConvergenceWarning: Stochastic Optimizer: Maximum iterations (1000) reached and
the optimization hasn't converged yet.
  warnings.warn(
/projects/proteinml/.links/miniconda3/envs/aidep/lib/python3.10/site-
packages/sklearn/neural_network/_multilayer_perceptron.py:690:
ConvergenceWarning: Stochastic Optimizer: Maximum iterations (1000) reached and
the optimization hasn't converged yet.
  warnings.warn(
/projects/proteinml/.links/miniconda3/envs/aidep/lib/python3.10/site-
packages/sklearn/neural_network/_multilayer_perceptron.py:690:
ConvergenceWarning: Stochastic Optimizer: Maximum iterations (1000) reached and
the optimization hasn't converged yet.
  warnings.warn(
```

[27]:
```
['none_esm4sites_mlp_scores.joblib']
```

1.6 4. Combined models

1.6.1 4.1 EVC plus one hot linear

[24]:
```
X_evc = evc.transform(X)
```

```
INFO:aide_predict.bespoke_models.base:Transforming input using EVMutationWrapper
  0%|          | 0/159129 [00:00<?, ?it/s]/kfs2/projects/proteinml/repos/aide_pr
edict/aide_predict/bespoke_models/predictors/evmutation.py:186: UserWarning:
Mutation M1m is not supported by EVCouplings statistics and will be ignored for
scoring.
  warnings.warn(f"Mutation {subs_from}{subs_pos}{subs_to} is not supported by
EVCouplings statistics and will be ignored for scoring.")
/kfs2/projects/proteinml/repos/aide_predict/aide_predict/bespoke_models/predicto
rs/evmutation.py:186: UserWarning: Mutation K2k is not supported by EVCouplings
statistics and will be ignored for scoring.
  warnings.warn(f"Mutation {subs_from}{subs_pos}{subs_to} is not supported by
EVCouplings statistics and will be ignored for scoring.")
/kfs2/projects/proteinml/repos/aide_predict/aide_predict/bespoke_models/predicto
rs/evmutation.py:186: UserWarning: Mutation G3g is not supported by EVCouplings
statistics and will be ignored for scoring.
  warnings.warn(f"Mutation {subs_from}{subs_pos}{subs_to} is not supported by
EVCouplings statistics and will be ignored for scoring.")
/kfs2/projects/proteinml/repos/aide_predict/aide_predict/bespoke_models/predicto
rs/evmutation.py:186: UserWarning: Mutation Y4y is not supported by EVCouplings
```

```
statistics and will be ignored for scoring.
  warnings.warn(f"Mutation {subs_from}{subs_pos}{subs_to} is not supported by
EVCouplings statistics and will be ignored for scoring.")
/kfs2/projects/proteinml/repos/aide_predict/aide_predict/bespoke_models/predicto
rs/evmutation.py:186: UserWarning: Mutation F5f is not supported by EVCouplings
statistics and will be ignored for scoring.
  warnings.warn(f"Mutation {subs_from}{subs_pos}{subs_to} is not supported by
EVCouplings statistics and will be ignored for scoring.")
/kfs2/projects/proteinml/repos/aide_predict/aide_predict/bespoke_models/predicto
rs/evmutation.py:186: UserWarning: Mutation G6g is not supported by EVCouplings
statistics and will be ignored for scoring.
  warnings.warn(f"Mutation {subs_from}{subs_pos}{subs_to} is not supported by
EVCouplings statistics and will be ignored for scoring.")
/kfs2/projects/proteinml/repos/aide_predict/aide_predict/bespoke_models/predicto
rs/evmutation.py:186: UserWarning: Mutation P7p is not supported by EVCouplings
statistics and will be ignored for scoring.
  warnings.warn(f"Mutation {subs_from}{subs_pos}{subs_to} is not supported by
EVCouplings statistics and will be ignored for scoring.")
/kfs2/projects/proteinml/repos/aide_predict/aide_predict/bespoke_models/predicto
rs/evmutation.py:186: UserWarning: Mutation Y8y is not supported by EVCouplings
statistics and will be ignored for scoring.
  warnings.warn(f"Mutation {subs_from}{subs_pos}{subs_to} is not supported by
EVCouplings statistics and will be ignored for scoring.")
/kfs2/projects/proteinml/repos/aide_predict/aide_predict/bespoke_models/predicto
rs/evmutation.py:186: UserWarning: Mutation G9g is not supported by EVCouplings
statistics and will be ignored for scoring.
  warnings.warn(f"Mutation {subs_from}{subs_pos}{subs_to} is not supported by
EVCouplings statistics and will be ignored for scoring.")
/kfs2/projects/proteinml/repos/aide_predict/aide_predict/bespoke_models/predicto
rs/evmutation.py:186: UserWarning: Mutation G10g is not supported by EVCouplings
statistics and will be ignored for scoring.
  warnings.warn(f"Mutation {subs_from}{subs_pos}{subs_to} is not supported by
EVCouplings statistics and will be ignored for scoring.")
/kfs2/projects/proteinml/repos/aide_predict/aide_predict/bespoke_models/predicto
rs/evmutation.py:186: UserWarning: Mutation Q11q is not supported by EVCouplings
statistics and will be ignored for scoring.
  warnings.warn(f"Mutation {subs_from}{subs_pos}{subs_to} is not supported by
EVCouplings statistics and will be ignored for scoring.")
/kfs2/projects/proteinml/repos/aide_predict/aide_predict/bespoke_models/predicto
rs/evmutation.py:186: UserWarning: Mutation Y12y is not supported by EVCouplings
statistics and will be ignored for scoring.
  warnings.warn(f"Mutation {subs_from}{subs_pos}{subs_to} is not supported by
EVCouplings statistics and will be ignored for scoring.")
/kfs2/projects/proteinml/repos/aide_predict/aide_predict/bespoke_models/predicto
rs/evmutation.py:186: UserWarning: Mutation V13v is not supported by EVCouplings
statistics and will be ignored for scoring.
  warnings.warn(f"Mutation {subs_from}{subs_pos}{subs_to} is not supported by
EVCouplings statistics and will be ignored for scoring.")
```

```
/kfs2/projects/proteinml/repos/aide_predict/aide_predict/bespoke_models/predicto
rs/evmutation.py:186: UserWarning: Mutation P14p is not supported by EVCouplings
statistics and will be ignored for scoring.
  warnings.warn(f"Mutation {subs_from}{subs_pos}{subs_to} is not supported by
EVCouplings statistics and will be ignored for scoring.")
/kfs2/projects/proteinml/repos/aide_predict/aide_predict/bespoke_models/predicto
rs/evmutation.py:186: UserWarning: Mutation E15e is not supported by EVCouplings
statistics and will be ignored for scoring.
  warnings.warn(f"Mutation {subs_from}{subs_pos}{subs_to} is not supported by
EVCouplings statistics and will be ignored for scoring.")
/kfs2/projects/proteinml/repos/aide_predict/aide_predict/bespoke_models/predicto
rs/evmutation.py:186: UserWarning: Mutation I16i is not supported by EVCouplings
statistics and will be ignored for scoring.
  warnings.warn(f"Mutation {subs_from}{subs_pos}{subs_to} is not supported by
EVCouplings statistics and will be ignored for scoring.")
/kfs2/projects/proteinml/repos/aide_predict/aide_predict/bespoke_models/predicto
rs/evmutation.py:186: UserWarning: Mutation L17l is not supported by EVCouplings
statistics and will be ignored for scoring.
  warnings.warn(f"Mutation {subs_from}{subs_pos}{subs_to} is not supported by
EVCouplings statistics and will be ignored for scoring.")
/kfs2/projects/proteinml/repos/aide_predict/aide_predict/bespoke_models/predicto
rs/evmutation.py:186: UserWarning: Mutation M18m is not supported by EVCouplings
statistics and will be ignored for scoring.
  warnings.warn(f"Mutation {subs_from}{subs_pos}{subs_to} is not supported by
EVCouplings statistics and will be ignored for scoring.")
/kfs2/projects/proteinml/repos/aide_predict/aide_predict/bespoke_models/predicto
rs/evmutation.py:186: UserWarning: Mutation G19g is not supported by EVCouplings
statistics and will be ignored for scoring.
  warnings.warn(f"Mutation {subs_from}{subs_pos}{subs_to} is not supported by
EVCouplings statistics and will be ignored for scoring.")
/kfs2/projects/proteinml/repos/aide_predict/aide_predict/bespoke_models/predicto
rs/evmutation.py:186: UserWarning: Mutation A20a is not supported by EVCouplings
statistics and will be ignored for scoring.
  warnings.warn(f"Mutation {subs_from}{subs_pos}{subs_to} is not supported by
EVCouplings statistics and will be ignored for scoring.")
/kfs2/projects/proteinml/repos/aide_predict/aide_predict/bespoke_models/predicto
rs/evmutation.py:186: UserWarning: Mutation L21l is not supported by EVCouplings
statistics and will be ignored for scoring.
  warnings.warn(f"Mutation {subs_from}{subs_pos}{subs_to} is not supported by
EVCouplings statistics and will be ignored for scoring.")
/kfs2/projects/proteinml/repos/aide_predict/aide_predict/bespoke_models/predicto
rs/evmutation.py:186: UserWarning: Mutation E22e is not supported by EVCouplings
statistics and will be ignored for scoring.
  warnings.warn(f"Mutation {subs_from}{subs_pos}{subs_to} is not supported by
EVCouplings statistics and will be ignored for scoring.")
/kfs2/projects/proteinml/repos/aide_predict/aide_predict/bespoke_models/predicto
rs/evmutation.py:186: UserWarning: Mutation E23e is not supported by EVCouplings
statistics and will be ignored for scoring.
```

```
  warnings.warn(f"Mutation {subs_from}{subs_pos}{subs_to} is not supported by
EVCouplings statistics and will be ignored for scoring.")
/kfs2/projects/proteinml/repos/aide_predict/aide_predict/bespoke_models/predicto
rs/evmutation.py:186: UserWarning: Mutation L24l is not supported by EVCouplings
statistics and will be ignored for scoring.
  warnings.warn(f"Mutation {subs_from}{subs_pos}{subs_to} is not supported by
EVCouplings statistics and will be ignored for scoring.")
/kfs2/projects/proteinml/repos/aide_predict/aide_predict/bespoke_models/predicto
rs/evmutation.py:186: UserWarning: Mutation E25e is not supported by EVCouplings
statistics and will be ignored for scoring.
  warnings.warn(f"Mutation {subs_from}{subs_pos}{subs_to} is not supported by
EVCouplings statistics and will be ignored for scoring.")
/kfs2/projects/proteinml/repos/aide_predict/aide_predict/bespoke_models/predicto
rs/evmutation.py:186: UserWarning: Mutation A26a is not supported by EVCouplings
statistics and will be ignored for scoring.
  warnings.warn(f"Mutation {subs_from}{subs_pos}{subs_to} is not supported by
EVCouplings statistics and will be ignored for scoring.")
/kfs2/projects/proteinml/repos/aide_predict/aide_predict/bespoke_models/predicto
rs/evmutation.py:186: UserWarning: Mutation A27a is not supported by EVCouplings
statistics and will be ignored for scoring.
  warnings.warn(f"Mutation {subs_from}{subs_pos}{subs_to} is not supported by
EVCouplings statistics and will be ignored for scoring.")
/kfs2/projects/proteinml/repos/aide_predict/aide_predict/bespoke_models/predicto
rs/evmutation.py:186: UserWarning: Mutation Y28y is not supported by EVCouplings
statistics and will be ignored for scoring.
  warnings.warn(f"Mutation {subs_from}{subs_pos}{subs_to} is not supported by
EVCouplings statistics and will be ignored for scoring.")
/kfs2/projects/proteinml/repos/aide_predict/aide_predict/bespoke_models/predicto
rs/evmutation.py:186: UserWarning: Mutation E29e is not supported by EVCouplings
statistics and will be ignored for scoring.
  warnings.warn(f"Mutation {subs_from}{subs_pos}{subs_to} is not supported by
EVCouplings statistics and will be ignored for scoring.")
/kfs2/projects/proteinml/repos/aide_predict/aide_predict/bespoke_models/predicto
rs/evmutation.py:186: UserWarning: Mutation G30g is not supported by EVCouplings
statistics and will be ignored for scoring.
  warnings.warn(f"Mutation {subs_from}{subs_pos}{subs_to} is not supported by
EVCouplings statistics and will be ignored for scoring.")
/kfs2/projects/proteinml/repos/aide_predict/aide_predict/bespoke_models/predicto
rs/evmutation.py:186: UserWarning: Mutation I31i is not supported by EVCouplings
statistics and will be ignored for scoring.
  warnings.warn(f"Mutation {subs_from}{subs_pos}{subs_to} is not supported by
EVCouplings statistics and will be ignored for scoring.")
/kfs2/projects/proteinml/repos/aide_predict/aide_predict/bespoke_models/predicto
rs/evmutation.py:186: UserWarning: Mutation M32m is not supported by EVCouplings
statistics and will be ignored for scoring.
  warnings.warn(f"Mutation {subs_from}{subs_pos}{subs_to} is not supported by
EVCouplings statistics and will be ignored for scoring.")
/kfs2/projects/proteinml/repos/aide_predict/aide_predict/bespoke_models/predicto
```

```
rs/evmutation.py:186: UserWarning: Mutation K33k is not supported by EVCouplings
statistics and will be ignored for scoring.
  warnings.warn(f"Mutation {subs_from}{subs_pos}{subs_to} is not supported by
EVCouplings statistics and will be ignored for scoring.")
/kfs2/projects/proteinml/repos/aide_predict/aide_predict/bespoke_models/predicto
rs/evmutation.py:186: UserWarning: Mutation D34d is not supported by EVCouplings
statistics and will be ignored for scoring.
  warnings.warn(f"Mutation {subs_from}{subs_pos}{subs_to} is not supported by
EVCouplings statistics and will be ignored for scoring.")
/kfs2/projects/proteinml/repos/aide_predict/aide_predict/bespoke_models/predicto
rs/evmutation.py:186: UserWarning: Mutation E35e is not supported by EVCouplings
statistics and will be ignored for scoring.
  warnings.warn(f"Mutation {subs_from}{subs_pos}{subs_to} is not supported by
EVCouplings statistics and will be ignored for scoring.")
/kfs2/projects/proteinml/repos/aide_predict/aide_predict/bespoke_models/predicto
rs/evmutation.py:186: UserWarning: Mutation S36s is not supported by EVCouplings
statistics and will be ignored for scoring.
  warnings.warn(f"Mutation {subs_from}{subs_pos}{subs_to} is not supported by
EVCouplings statistics and will be ignored for scoring.")
/kfs2/projects/proteinml/repos/aide_predict/aide_predict/bespoke_models/predicto
rs/evmutation.py:186: UserWarning: Mutation F37f is not supported by EVCouplings
statistics and will be ignored for scoring.
  warnings.warn(f"Mutation {subs_from}{subs_pos}{subs_to} is not supported by
EVCouplings statistics and will be ignored for scoring.")
/kfs2/projects/proteinml/repos/aide_predict/aide_predict/bespoke_models/predicto
rs/evmutation.py:186: UserWarning: Mutation W38w is not supported by EVCouplings
statistics and will be ignored for scoring.
  warnings.warn(f"Mutation {subs_from}{subs_pos}{subs_to} is not supported by
EVCouplings statistics and will be ignored for scoring.")
/kfs2/projects/proteinml/repos/aide_predict/aide_predict/bespoke_models/predicto
rs/evmutation.py:186: UserWarning: Mutation K39k is not supported by EVCouplings
statistics and will be ignored for scoring.
  warnings.warn(f"Mutation {subs_from}{subs_pos}{subs_to} is not supported by
EVCouplings statistics and will be ignored for scoring.")
/kfs2/projects/proteinml/repos/aide_predict/aide_predict/bespoke_models/predicto
rs/evmutation.py:186: UserWarning: Mutation E40e is not supported by EVCouplings
statistics and will be ignored for scoring.
  warnings.warn(f"Mutation {subs_from}{subs_pos}{subs_to} is not supported by
EVCouplings statistics and will be ignored for scoring.")
/kfs2/projects/proteinml/repos/aide_predict/aide_predict/bespoke_models/predicto
rs/evmutation.py:186: UserWarning: Mutation F41f is not supported by EVCouplings
statistics and will be ignored for scoring.
  warnings.warn(f"Mutation {subs_from}{subs_pos}{subs_to} is not supported by
EVCouplings statistics and will be ignored for scoring.")
/kfs2/projects/proteinml/repos/aide_predict/aide_predict/bespoke_models/predicto
rs/evmutation.py:186: UserWarning: Mutation N42n is not supported by EVCouplings
statistics and will be ignored for scoring.
  warnings.warn(f"Mutation {subs_from}{subs_pos}{subs_to} is not supported by
```

```
EVCouplings statistics and will be ignored for scoring.")
/kfs2/projects/proteinml/repos/aide_predict/aide_predict/bespoke_models/predicto
rs/evmutation.py:186: UserWarning: Mutation D43d is not supported by EVCouplings
statistics and will be ignored for scoring.
  warnings.warn(f"Mutation {subs_from}{subs_pos}{subs_to} is not supported by
EVCouplings statistics and will be ignored for scoring.")
/kfs2/projects/proteinml/repos/aide_predict/aide_predict/bespoke_models/predicto
rs/evmutation.py:186: UserWarning: Mutation L44l is not supported by EVCouplings
statistics and will be ignored for scoring.
  warnings.warn(f"Mutation {subs_from}{subs_pos}{subs_to} is not supported by
EVCouplings statistics and will be ignored for scoring.")
/kfs2/projects/proteinml/repos/aide_predict/aide_predict/bespoke_models/predicto
rs/evmutation.py:186: UserWarning: Mutation L45l is not supported by EVCouplings
statistics and will be ignored for scoring.
  warnings.warn(f"Mutation {subs_from}{subs_pos}{subs_to} is not supported by
EVCouplings statistics and will be ignored for scoring.")
/kfs2/projects/proteinml/repos/aide_predict/aide_predict/bespoke_models/predicto
rs/evmutation.py:186: UserWarning: Mutation R46r is not supported by EVCouplings
statistics and will be ignored for scoring.
  warnings.warn(f"Mutation {subs_from}{subs_pos}{subs_to} is not supported by
EVCouplings statistics and will be ignored for scoring.")
/kfs2/projects/proteinml/repos/aide_predict/aide_predict/bespoke_models/predicto
rs/evmutation.py:186: UserWarning: Mutation D47d is not supported by EVCouplings
statistics and will be ignored for scoring.
  warnings.warn(f"Mutation {subs_from}{subs_pos}{subs_to} is not supported by
EVCouplings statistics and will be ignored for scoring.")
/kfs2/projects/proteinml/repos/aide_predict/aide_predict/bespoke_models/predicto
rs/evmutation.py:186: UserWarning: Mutation Y48y is not supported by EVCouplings
statistics and will be ignored for scoring.
  warnings.warn(f"Mutation {subs_from}{subs_pos}{subs_to} is not supported by
EVCouplings statistics and will be ignored for scoring.")
/kfs2/projects/proteinml/repos/aide_predict/aide_predict/bespoke_models/predicto
rs/evmutation.py:186: UserWarning: Mutation D134d is not supported by
EVCouplings statistics and will be ignored for scoring.
  warnings.warn(f"Mutation {subs_from}{subs_pos}{subs_to} is not supported by
EVCouplings statistics and will be ignored for scoring.")
/kfs2/projects/proteinml/repos/aide_predict/aide_predict/bespoke_models/predicto
rs/evmutation.py:186: UserWarning: Mutation I136i is not supported by
EVCouplings statistics and will be ignored for scoring.
  warnings.warn(f"Mutation {subs_from}{subs_pos}{subs_to} is not supported by
EVCouplings statistics and will be ignored for scoring.")
/kfs2/projects/proteinml/repos/aide_predict/aide_predict/bespoke_models/predicto
rs/evmutation.py:186: UserWarning: Mutation R137r is not supported by
EVCouplings statistics and will be ignored for scoring.
  warnings.warn(f"Mutation {subs_from}{subs_pos}{subs_to} is not supported by
EVCouplings statistics and will be ignored for scoring.")
/kfs2/projects/proteinml/repos/aide_predict/aide_predict/bespoke_models/predicto
rs/evmutation.py:186: UserWarning: Mutation Q138q is not supported by
```

```
EVCouplings statistics and will be ignored for scoring.
  warnings.warn(f"Mutation {subs_from}{subs_pos}{subs_to} is not supported by
EVCouplings statistics and will be ignored for scoring.")
/kfs2/projects/proteinml/repos/aide_predict/aide_predict/bespoke_models/predicto
rs/evmutation.py:186: UserWarning: Mutation S157s is not supported by
EVCouplings statistics and will be ignored for scoring.
  warnings.warn(f"Mutation {subs_from}{subs_pos}{subs_to} is not supported by
EVCouplings statistics and will be ignored for scoring.")
/kfs2/projects/proteinml/repos/aide_predict/aide_predict/bespoke_models/predicto
rs/evmutation.py:186: UserWarning: Mutation G158g is not supported by
EVCouplings statistics and will be ignored for scoring.
  warnings.warn(f"Mutation {subs_from}{subs_pos}{subs_to} is not supported by
EVCouplings statistics and will be ignored for scoring.")
/kfs2/projects/proteinml/repos/aide_predict/aide_predict/bespoke_models/predicto
rs/evmutation.py:186: UserWarning: Mutation S159s is not supported by
EVCouplings statistics and will be ignored for scoring.
  warnings.warn(f"Mutation {subs_from}{subs_pos}{subs_to} is not supported by
EVCouplings statistics and will be ignored for scoring.")
/kfs2/projects/proteinml/repos/aide_predict/aide_predict/bespoke_models/predicto
rs/evmutation.py:186: UserWarning: Mutation L177l is not supported by
EVCouplings statistics and will be ignored for scoring.
  warnings.warn(f"Mutation {subs_from}{subs_pos}{subs_to} is not supported by
EVCouplings statistics and will be ignored for scoring.")
/kfs2/projects/proteinml/repos/aide_predict/aide_predict/bespoke_models/predicto
rs/evmutation.py:186: UserWarning: Mutation T179t is not supported by
EVCouplings statistics and will be ignored for scoring.
  warnings.warn(f"Mutation {subs_from}{subs_pos}{subs_to} is not supported by
EVCouplings statistics and will be ignored for scoring.")
/kfs2/projects/proteinml/repos/aide_predict/aide_predict/bespoke_models/predicto
rs/evmutation.py:186: UserWarning: Mutation T180t is not supported by
EVCouplings statistics and will be ignored for scoring.
  warnings.warn(f"Mutation {subs_from}{subs_pos}{subs_to} is not supported by
EVCouplings statistics and will be ignored for scoring.")
/kfs2/projects/proteinml/repos/aide_predict/aide_predict/bespoke_models/predicto
rs/evmutation.py:186: UserWarning: Mutation Y181y is not supported by
EVCouplings statistics and will be ignored for scoring.
  warnings.warn(f"Mutation {subs_from}{subs_pos}{subs_to} is not supported by
EVCouplings statistics and will be ignored for scoring.")
/kfs2/projects/proteinml/repos/aide_predict/aide_predict/bespoke_models/predicto
rs/evmutation.py:186: UserWarning: Mutation V183G is not supported by
EVCouplings statistics and will be ignored for scoring.
  warnings.warn(f"Mutation {subs_from}{subs_pos}{subs_to} is not supported by
EVCouplings statistics and will be ignored for scoring.")
/kfs2/projects/proteinml/repos/aide_predict/aide_predict/bespoke_models/predicto
rs/evmutation.py:186: UserWarning: Mutation F184N is not supported by
EVCouplings statistics and will be ignored for scoring.
  warnings.warn(f"Mutation {subs_from}{subs_pos}{subs_to} is not supported by
EVCouplings statistics and will be ignored for scoring.")
```

```
/kfs2/projects/proteinml/repos/aide_predict/aide_predict/bespoke_models/predicto
rs/evmutation.py:186: UserWarning: Mutation P192p is not supported by
EVCouplings statistics and will be ignored for scoring.
  warnings.warn(f"Mutation {subs_from}{subs_pos}{subs_to} is not supported by
EVCouplings statistics and will be ignored for scoring.")
/kfs2/projects/proteinml/repos/aide_predict/aide_predict/bespoke_models/predicto
rs/evmutation.py:186: UserWarning: Mutation I196i is not supported by
EVCouplings statistics and will be ignored for scoring.
  warnings.warn(f"Mutation {subs_from}{subs_pos}{subs_to} is not supported by
EVCouplings statistics and will be ignored for scoring.")
/kfs2/projects/proteinml/repos/aide_predict/aide_predict/bespoke_models/predicto
rs/evmutation.py:186: UserWarning: Mutation V197v is not supported by
EVCouplings statistics and will be ignored for scoring.
  warnings.warn(f"Mutation {subs_from}{subs_pos}{subs_to} is not supported by
EVCouplings statistics and will be ignored for scoring.")
/kfs2/projects/proteinml/repos/aide_predict/aide_predict/bespoke_models/predicto
rs/evmutation.py:186: UserWarning: Mutation P213p is not supported by
EVCouplings statistics and will be ignored for scoring.
  warnings.warn(f"Mutation {subs_from}{subs_pos}{subs_to} is not supported by
EVCouplings statistics and will be ignored for scoring.")
/kfs2/projects/proteinml/repos/aide_predict/aide_predict/bespoke_models/predicto
rs/evmutation.py:186: UserWarning: Mutation E214e is not supported by
EVCouplings statistics and will be ignored for scoring.
  warnings.warn(f"Mutation {subs_from}{subs_pos}{subs_to} is not supported by
EVCouplings statistics and will be ignored for scoring.")
/kfs2/projects/proteinml/repos/aide_predict/aide_predict/bespoke_models/predicto
rs/evmutation.py:186: UserWarning: Mutation K215k is not supported by
EVCouplings statistics and will be ignored for scoring.
  warnings.warn(f"Mutation {subs_from}{subs_pos}{subs_to} is not supported by
EVCouplings statistics and will be ignored for scoring.")
/kfs2/projects/proteinml/repos/aide_predict/aide_predict/bespoke_models/predicto
rs/evmutation.py:186: UserWarning: Mutation E216e is not supported by
EVCouplings statistics and will be ignored for scoring.
  warnings.warn(f"Mutation {subs_from}{subs_pos}{subs_to} is not supported by
EVCouplings statistics and will be ignored for scoring.")
/kfs2/projects/proteinml/repos/aide_predict/aide_predict/bespoke_models/predicto
rs/evmutation.py:186: UserWarning: Mutation G217g is not supported by
EVCouplings statistics and will be ignored for scoring.
  warnings.warn(f"Mutation {subs_from}{subs_pos}{subs_to} is not supported by
EVCouplings statistics and will be ignored for scoring.")
/kfs2/projects/proteinml/repos/aide_predict/aide_predict/bespoke_models/predicto
rs/evmutation.py:186: UserWarning: Mutation G260g is not supported by
EVCouplings statistics and will be ignored for scoring.
  warnings.warn(f"Mutation {subs_from}{subs_pos}{subs_to} is not supported by
EVCouplings statistics and will be ignored for scoring.")
/kfs2/projects/proteinml/repos/aide_predict/aide_predict/bespoke_models/predicto
rs/evmutation.py:186: UserWarning: Mutation K261k is not supported by
EVCouplings statistics and will be ignored for scoring.
```

```
  warnings.warn(f"Mutation {subs_from}{subs_pos}{subs_to} is not supported by
EVCouplings statistics and will be ignored for scoring.")
/kfs2/projects/proteinml/repos/aide_predict/aide_predict/bespoke_models/predicto
rs/evmutation.py:186: UserWarning: Mutation H262h is not supported by
EVCouplings statistics and will be ignored for scoring.
  warnings.warn(f"Mutation {subs_from}{subs_pos}{subs_to} is not supported by
EVCouplings statistics and will be ignored for scoring.")
/kfs2/projects/proteinml/repos/aide_predict/aide_predict/bespoke_models/predicto
rs/evmutation.py:186: UserWarning: Mutation A263a is not supported by
EVCouplings statistics and will be ignored for scoring.
  warnings.warn(f"Mutation {subs_from}{subs_pos}{subs_to} is not supported by
EVCouplings statistics and will be ignored for scoring.")
/kfs2/projects/proteinml/repos/aide_predict/aide_predict/bespoke_models/predicto
rs/evmutation.py:186: UserWarning: Mutation A264a is not supported by
EVCouplings statistics and will be ignored for scoring.
  warnings.warn(f"Mutation {subs_from}{subs_pos}{subs_to} is not supported by
EVCouplings statistics and will be ignored for scoring.")
/kfs2/projects/proteinml/repos/aide_predict/aide_predict/bespoke_models/predicto
rs/evmutation.py:186: UserWarning: Mutation H275h is not supported by
EVCouplings statistics and will be ignored for scoring.
  warnings.warn(f"Mutation {subs_from}{subs_pos}{subs_to} is not supported by
EVCouplings statistics and will be ignored for scoring.")
/kfs2/projects/proteinml/repos/aide_predict/aide_predict/bespoke_models/predicto
rs/evmutation.py:186: UserWarning: Mutation G276g is not supported by
EVCouplings statistics and will be ignored for scoring.
  warnings.warn(f"Mutation {subs_from}{subs_pos}{subs_to} is not supported by
EVCouplings statistics and will be ignored for scoring.")
/kfs2/projects/proteinml/repos/aide_predict/aide_predict/bespoke_models/predicto
rs/evmutation.py:186: UserWarning: Mutation S277s is not supported by
EVCouplings statistics and will be ignored for scoring.
  warnings.warn(f"Mutation {subs_from}{subs_pos}{subs_to} is not supported by
EVCouplings statistics and will be ignored for scoring.")
/kfs2/projects/proteinml/repos/aide_predict/aide_predict/bespoke_models/predicto
rs/evmutation.py:186: UserWarning: Mutation K278k is not supported by
EVCouplings statistics and will be ignored for scoring.
  warnings.warn(f"Mutation {subs_from}{subs_pos}{subs_to} is not supported by
EVCouplings statistics and will be ignored for scoring.")
/kfs2/projects/proteinml/repos/aide_predict/aide_predict/bespoke_models/predicto
rs/evmutation.py:186: UserWarning: Mutation T279t is not supported by
EVCouplings statistics and will be ignored for scoring.
  warnings.warn(f"Mutation {subs_from}{subs_pos}{subs_to} is not supported by
EVCouplings statistics and will be ignored for scoring.")
/kfs2/projects/proteinml/repos/aide_predict/aide_predict/bespoke_models/predicto
rs/evmutation.py:186: UserWarning: Mutation F280f is not supported by
EVCouplings statistics and will be ignored for scoring.
  warnings.warn(f"Mutation {subs_from}{subs_pos}{subs_to} is not supported by
EVCouplings statistics and will be ignored for scoring.")
/kfs2/projects/proteinml/repos/aide_predict/aide_predict/bespoke_models/predicto
```

```
rs/evmutation.py:186: UserWarning: Mutation V281v is not supported by
EVCouplings statistics and will be ignored for scoring.
  warnings.warn(f"Mutation {subs_from}{subs_pos}{subs_to} is not supported by
EVCouplings statistics and will be ignored for scoring.")
/kfs2/projects/proteinml/repos/aide_predict/aide_predict/bespoke_models/predicto
rs/evmutation.py:186: UserWarning: Mutation L282l is not supported by
EVCouplings statistics and will be ignored for scoring.
  warnings.warn(f"Mutation {subs_from}{subs_pos}{subs_to} is not supported by
EVCouplings statistics and will be ignored for scoring.")
/kfs2/projects/proteinml/repos/aide_predict/aide_predict/bespoke_models/predicto
rs/evmutation.py:186: UserWarning: Mutation Q283q is not supported by
EVCouplings statistics and will be ignored for scoring.
  warnings.warn(f"Mutation {subs_from}{subs_pos}{subs_to} is not supported by
EVCouplings statistics and will be ignored for scoring.")
/kfs2/projects/proteinml/repos/aide_predict/aide_predict/bespoke_models/predicto
rs/evmutation.py:186: UserWarning: Mutation D284d is not supported by
EVCouplings statistics and will be ignored for scoring.
  warnings.warn(f"Mutation {subs_from}{subs_pos}{subs_to} is not supported by
EVCouplings statistics and will be ignored for scoring.")
/kfs2/projects/proteinml/repos/aide_predict/aide_predict/bespoke_models/predicto
rs/evmutation.py:186: UserWarning: Mutation D285d is not supported by
EVCouplings statistics and will be ignored for scoring.
  warnings.warn(f"Mutation {subs_from}{subs_pos}{subs_to} is not supported by
EVCouplings statistics and will be ignored for scoring.")
/kfs2/projects/proteinml/repos/aide_predict/aide_predict/bespoke_models/predicto
rs/evmutation.py:186: UserWarning: Mutation W286w is not supported by
EVCouplings statistics and will be ignored for scoring.
  warnings.warn(f"Mutation {subs_from}{subs_pos}{subs_to} is not supported by
EVCouplings statistics and will be ignored for scoring.")
/kfs2/projects/proteinml/repos/aide_predict/aide_predict/bespoke_models/predicto
rs/evmutation.py:186: UserWarning: Mutation G287g is not supported by
EVCouplings statistics and will be ignored for scoring.
  warnings.warn(f"Mutation {subs_from}{subs_pos}{subs_to} is not supported by
EVCouplings statistics and will be ignored for scoring.")
/kfs2/projects/proteinml/repos/aide_predict/aide_predict/bespoke_models/predicto
rs/evmutation.py:186: UserWarning: Mutation Q288q is not supported by
EVCouplings statistics and will be ignored for scoring.
  warnings.warn(f"Mutation {subs_from}{subs_pos}{subs_to} is not supported by
EVCouplings statistics and will be ignored for scoring.")
/kfs2/projects/proteinml/repos/aide_predict/aide_predict/bespoke_models/predicto
rs/evmutation.py:186: UserWarning: Mutation V289v is not supported by
EVCouplings statistics and will be ignored for scoring.
  warnings.warn(f"Mutation {subs_from}{subs_pos}{subs_to} is not supported by
EVCouplings statistics and will be ignored for scoring.")
/kfs2/projects/proteinml/repos/aide_predict/aide_predict/bespoke_models/predicto
rs/evmutation.py:186: UserWarning: Mutation Q290q is not supported by
EVCouplings statistics and will be ignored for scoring.
  warnings.warn(f"Mutation {subs_from}{subs_pos}{subs_to} is not supported by
```

```
EVCouplings statistics and will be ignored for scoring.")
/kfs2/projects/proteinml/repos/aide_predict/aide_predict/bespoke_models/predicto
rs/evmutation.py:186: UserWarning: Mutation V291v is not supported by
EVCouplings statistics and will be ignored for scoring.
  warnings.warn(f"Mutation {subs_from}{subs_pos}{subs_to} is not supported by
EVCouplings statistics and will be ignored for scoring.")
/kfs2/projects/proteinml/repos/aide_predict/aide_predict/bespoke_models/predicto
rs/evmutation.py:186: UserWarning: Mutation S292s is not supported by
EVCouplings statistics and will be ignored for scoring.
  warnings.warn(f"Mutation {subs_from}{subs_pos}{subs_to} is not supported by
EVCouplings statistics and will be ignored for scoring.")
/kfs2/projects/proteinml/repos/aide_predict/aide_predict/bespoke_models/predicto
rs/evmutation.py:186: UserWarning: Mutation V378v is not supported by
EVCouplings statistics and will be ignored for scoring.
  warnings.warn(f"Mutation {subs_from}{subs_pos}{subs_to} is not supported by
EVCouplings statistics and will be ignored for scoring.")
/kfs2/projects/proteinml/repos/aide_predict/aide_predict/bespoke_models/predicto
rs/evmutation.py:186: UserWarning: Mutation L379l is not supported by
EVCouplings statistics and will be ignored for scoring.
  warnings.warn(f"Mutation {subs_from}{subs_pos}{subs_to} is not supported by
EVCouplings statistics and will be ignored for scoring.")
/kfs2/projects/proteinml/repos/aide_predict/aide_predict/bespoke_models/predicto
rs/evmutation.py:186: UserWarning: Mutation N380n is not supported by
EVCouplings statistics and will be ignored for scoring.
  warnings.warn(f"Mutation {subs_from}{subs_pos}{subs_to} is not supported by
EVCouplings statistics and will be ignored for scoring.")
/kfs2/projects/proteinml/repos/aide_predict/aide_predict/bespoke_models/predicto
rs/evmutation.py:186: UserWarning: Mutation H381h is not supported by
EVCouplings statistics and will be ignored for scoring.
  warnings.warn(f"Mutation {subs_from}{subs_pos}{subs_to} is not supported by
EVCouplings statistics and will be ignored for scoring.")
/kfs2/projects/proteinml/repos/aide_predict/aide_predict/bespoke_models/predicto
rs/evmutation.py:186: UserWarning: Mutation P382p is not supported by
EVCouplings statistics and will be ignored for scoring.
  warnings.warn(f"Mutation {subs_from}{subs_pos}{subs_to} is not supported by
EVCouplings statistics and will be ignored for scoring.")
/kfs2/projects/proteinml/repos/aide_predict/aide_predict/bespoke_models/predicto
rs/evmutation.py:186: UserWarning: Mutation Y383y is not supported by
EVCouplings statistics and will be ignored for scoring.
  warnings.warn(f"Mutation {subs_from}{subs_pos}{subs_to} is not supported by
EVCouplings statistics and will be ignored for scoring.")
/kfs2/projects/proteinml/repos/aide_predict/aide_predict/bespoke_models/predicto
rs/evmutation.py:186: UserWarning: Mutation V384v is not supported by
EVCouplings statistics and will be ignored for scoring.
  warnings.warn(f"Mutation {subs_from}{subs_pos}{subs_to} is not supported by
EVCouplings statistics and will be ignored for scoring.")
/kfs2/projects/proteinml/repos/aide_predict/aide_predict/bespoke_models/predicto
rs/evmutation.py:186: UserWarning: Mutation R385r is not supported by
```

```
EVCouplings statistics and will be ignored for scoring.
  warnings.warn(f"Mutation {subs_from}{subs_pos}{subs_to} is not supported by
EVCouplings statistics and will be ignored for scoring.")
/kfs2/projects/proteinml/repos/aide_predict/aide_predict/bespoke_models/predicto
rs/evmutation.py:186: UserWarning: Mutation E386e is not supported by
EVCouplings statistics and will be ignored for scoring.
  warnings.warn(f"Mutation {subs_from}{subs_pos}{subs_to} is not supported by
EVCouplings statistics and will be ignored for scoring.")
/kfs2/projects/proteinml/repos/aide_predict/aide_predict/bespoke_models/predicto
rs/evmutation.py:186: UserWarning: Mutation R387r is not supported by
EVCouplings statistics and will be ignored for scoring.
  warnings.warn(f"Mutation {subs_from}{subs_pos}{subs_to} is not supported by
EVCouplings statistics and will be ignored for scoring.")
/kfs2/projects/proteinml/repos/aide_predict/aide_predict/bespoke_models/predicto
rs/evmutation.py:186: UserWarning: Mutation I388i is not supported by
EVCouplings statistics and will be ignored for scoring.
  warnings.warn(f"Mutation {subs_from}{subs_pos}{subs_to} is not supported by
EVCouplings statistics and will be ignored for scoring.")
/kfs2/projects/proteinml/repos/aide_predict/aide_predict/bespoke_models/predicto
rs/evmutation.py:186: UserWarning: Mutation R389r is not supported by
EVCouplings statistics and will be ignored for scoring.
  warnings.warn(f"Mutation {subs_from}{subs_pos}{subs_to} is not supported by
EVCouplings statistics and will be ignored for scoring.")
/kfs2/projects/proteinml/repos/aide_predict/aide_predict/bespoke_models/predicto
rs/evmutation.py:186: UserWarning: Mutation L390l is not supported by
EVCouplings statistics and will be ignored for scoring.
  warnings.warn(f"Mutation {subs_from}{subs_pos}{subs_to} is not supported by
EVCouplings statistics and will be ignored for scoring.")
/kfs2/projects/proteinml/repos/aide_predict/aide_predict/bespoke_models/predicto
rs/evmutation.py:186: UserWarning: Mutation E391e is not supported by
EVCouplings statistics and will be ignored for scoring.
  warnings.warn(f"Mutation {subs_from}{subs_pos}{subs_to} is not supported by
EVCouplings statistics and will be ignored for scoring.")
/kfs2/projects/proteinml/repos/aide_predict/aide_predict/bespoke_models/predicto
rs/evmutation.py:186: UserWarning: Mutation H392h is not supported by
EVCouplings statistics and will be ignored for scoring.
  warnings.warn(f"Mutation {subs_from}{subs_pos}{subs_to} is not supported by
EVCouplings statistics and will be ignored for scoring.")
/kfs2/projects/proteinml/repos/aide_predict/aide_predict/bespoke_models/predicto
rs/evmutation.py:186: UserWarning: Mutation H393h is not supported by
EVCouplings statistics and will be ignored for scoring.
  warnings.warn(f"Mutation {subs_from}{subs_pos}{subs_to} is not supported by
EVCouplings statistics and will be ignored for scoring.")
/kfs2/projects/proteinml/repos/aide_predict/aide_predict/bespoke_models/predicto
rs/evmutation.py:186: UserWarning: Mutation H394h is not supported by
EVCouplings statistics and will be ignored for scoring.
  warnings.warn(f"Mutation {subs_from}{subs_pos}{subs_to} is not supported by
EVCouplings statistics and will be ignored for scoring.")
```

```
/kfs2/projects/proteinml/repos/aide_predict/aide_predict/bespoke_models/predicto
rs/evmutation.py:186: UserWarning: Mutation H395h is not supported by
EVCouplings statistics and will be ignored for scoring.
  warnings.warn(f"Mutation {subs_from}{subs_pos}{subs_to} is not supported by
EVCouplings statistics and will be ignored for scoring.")
/kfs2/projects/proteinml/repos/aide_predict/aide_predict/bespoke_models/predicto
rs/evmutation.py:186: UserWarning: Mutation H396h is not supported by
EVCouplings statistics and will be ignored for scoring.
  warnings.warn(f"Mutation {subs_from}{subs_pos}{subs_to} is not supported by
EVCouplings statistics and will be ignored for scoring.")
/kfs2/projects/proteinml/repos/aide_predict/aide_predict/bespoke_models/predicto
rs/evmutation.py:186: UserWarning: Mutation H397h is not supported by
EVCouplings statistics and will be ignored for scoring.
  warnings.warn(f"Mutation {subs_from}{subs_pos}{subs_to} is not supported by
EVCouplings statistics and will be ignored for scoring.")
  0%|          | 1/159129 [00:06<306:11:44,  6.93s/it]/kfs2/projects/proteinml/r
epos/aide_predict/aide_predict/bespoke_models/predictors/evmutation.py:186:
UserWarning: Mutation M1m is not supported by EVCouplings statistics and will be
ignored for scoring.
  warnings.warn(f"Mutation {subs_from}{subs_pos}{subs_to} is not supported by
EVCouplings statistics and will be ignored for scoring.")
/kfs2/projects/proteinml/repos/aide_predict/aide_predict/bespoke_models/predicto
rs/evmutation.py:186: UserWarning: Mutation K2k is not supported by EVCouplings
statistics and will be ignored for scoring.
  warnings.warn(f"Mutation {subs_from}{subs_pos}{subs_to} is not supported by
EVCouplings statistics and will be ignored for scoring.")
/kfs2/projects/proteinml/repos/aide_predict/aide_predict/bespoke_models/predicto
rs/evmutation.py:186: UserWarning: Mutation G3g is not supported by EVCouplings
statistics and will be ignored for scoring.
  warnings.warn(f"Mutation {subs_from}{subs_pos}{subs_to} is not supported by
EVCouplings statistics and will be ignored for scoring.")
/kfs2/projects/proteinml/repos/aide_predict/aide_predict/bespoke_models/predicto
rs/evmutation.py:186: UserWarning: Mutation Y4y is not supported by EVCouplings
statistics and will be ignored for scoring.
  warnings.warn(f"Mutation {subs_from}{subs_pos}{subs_to} is not supported by
EVCouplings statistics and will be ignored for scoring.")
/kfs2/projects/proteinml/repos/aide_predict/aide_predict/bespoke_models/predicto
rs/evmutation.py:186: UserWarning: Mutation F5f is not supported by EVCouplings
statistics and will be ignored for scoring.
  warnings.warn(f"Mutation {subs_from}{subs_pos}{subs_to} is not supported by
EVCouplings statistics and will be ignored for scoring.")
/kfs2/projects/proteinml/repos/aide_predict/aide_predict/bespoke_models/predicto
rs/evmutation.py:186: UserWarning: Mutation G6g is not supported by EVCouplings
statistics and will be ignored for scoring.
  warnings.warn(f"Mutation {subs_from}{subs_pos}{subs_to} is not supported by
EVCouplings statistics and will be ignored for scoring.")
/kfs2/projects/proteinml/repos/aide_predict/aide_predict/bespoke_models/predicto
rs/evmutation.py:186: UserWarning: Mutation P7p is not supported by EVCouplings
```

```
statistics and will be ignored for scoring.
  warnings.warn(f"Mutation {subs_from}{subs_pos}{subs_to} is not supported by
EVCouplings statistics and will be ignored for scoring.")
/kfs2/projects/proteinml/repos/aide_predict/aide_predict/bespoke_models/predicto
rs/evmutation.py:186: UserWarning: Mutation Y8y is not supported by EVCouplings
statistics and will be ignored for scoring.
  warnings.warn(f"Mutation {subs_from}{subs_pos}{subs_to} is not supported by
EVCouplings statistics and will be ignored for scoring.")
/kfs2/projects/proteinml/repos/aide_predict/aide_predict/bespoke_models/predicto
rs/evmutation.py:186: UserWarning: Mutation G9g is not supported by EVCouplings
statistics and will be ignored for scoring.
  warnings.warn(f"Mutation {subs_from}{subs_pos}{subs_to} is not supported by
EVCouplings statistics and will be ignored for scoring.")
/kfs2/projects/proteinml/repos/aide_predict/aide_predict/bespoke_models/predicto
rs/evmutation.py:186: UserWarning: Mutation G10g is not supported by EVCouplings
statistics and will be ignored for scoring.
  warnings.warn(f"Mutation {subs_from}{subs_pos}{subs_to} is not supported by
EVCouplings statistics and will be ignored for scoring.")
/kfs2/projects/proteinml/repos/aide_predict/aide_predict/bespoke_models/predicto
rs/evmutation.py:186: UserWarning: Mutation Q11q is not supported by EVCouplings
statistics and will be ignored for scoring.
  warnings.warn(f"Mutation {subs_from}{subs_pos}{subs_to} is not supported by
EVCouplings statistics and will be ignored for scoring.")
/kfs2/projects/proteinml/repos/aide_predict/aide_predict/bespoke_models/predicto
rs/evmutation.py:186: UserWarning: Mutation Y12y is not supported by EVCouplings
statistics and will be ignored for scoring.
  warnings.warn(f"Mutation {subs_from}{subs_pos}{subs_to} is not supported by
EVCouplings statistics and will be ignored for scoring.")
/kfs2/projects/proteinml/repos/aide_predict/aide_predict/bespoke_models/predicto
rs/evmutation.py:186: UserWarning: Mutation V13v is not supported by EVCouplings
statistics and will be ignored for scoring.
  warnings.warn(f"Mutation {subs_from}{subs_pos}{subs_to} is not supported by
EVCouplings statistics and will be ignored for scoring.")
/kfs2/projects/proteinml/repos/aide_predict/aide_predict/bespoke_models/predicto
rs/evmutation.py:186: UserWarning: Mutation P14p is not supported by EVCouplings
statistics and will be ignored for scoring.
  warnings.warn(f"Mutation {subs_from}{subs_pos}{subs_to} is not supported by
EVCouplings statistics and will be ignored for scoring.")
/kfs2/projects/proteinml/repos/aide_predict/aide_predict/bespoke_models/predicto
rs/evmutation.py:186: UserWarning: Mutation E15e is not supported by EVCouplings
statistics and will be ignored for scoring.
  warnings.warn(f"Mutation {subs_from}{subs_pos}{subs_to} is not supported by
EVCouplings statistics and will be ignored for scoring.")
/kfs2/projects/proteinml/repos/aide_predict/aide_predict/bespoke_models/predicto
rs/evmutation.py:186: UserWarning: Mutation I16i is not supported by EVCouplings
statistics and will be ignored for scoring.
  warnings.warn(f"Mutation {subs_from}{subs_pos}{subs_to} is not supported by
EVCouplings statistics and will be ignored for scoring.")
```

```
/kfs2/projects/proteinml/repos/aide_predict/aide_predict/bespoke_models/predicto
rs/evmutation.py:186: UserWarning: Mutation L17l is not supported by EVCouplings
statistics and will be ignored for scoring.
  warnings.warn(f"Mutation {subs_from}{subs_pos}{subs_to} is not supported by
EVCouplings statistics and will be ignored for scoring.")
/kfs2/projects/proteinml/repos/aide_predict/aide_predict/bespoke_models/predicto
rs/evmutation.py:186: UserWarning: Mutation M18m is not supported by EVCouplings
statistics and will be ignored for scoring.
  warnings.warn(f"Mutation {subs_from}{subs_pos}{subs_to} is not supported by
EVCouplings statistics and will be ignored for scoring.")
/kfs2/projects/proteinml/repos/aide_predict/aide_predict/bespoke_models/predicto
rs/evmutation.py:186: UserWarning: Mutation G19g is not supported by EVCouplings
statistics and will be ignored for scoring.
  warnings.warn(f"Mutation {subs_from}{subs_pos}{subs_to} is not supported by
EVCouplings statistics and will be ignored for scoring.")
/kfs2/projects/proteinml/repos/aide_predict/aide_predict/bespoke_models/predicto
rs/evmutation.py:186: UserWarning: Mutation A20a is not supported by EVCouplings
statistics and will be ignored for scoring.
  warnings.warn(f"Mutation {subs_from}{subs_pos}{subs_to} is not supported by
EVCouplings statistics and will be ignored for scoring.")
/kfs2/projects/proteinml/repos/aide_predict/aide_predict/bespoke_models/predicto
rs/evmutation.py:186: UserWarning: Mutation L21l is not supported by EVCouplings
statistics and will be ignored for scoring.
  warnings.warn(f"Mutation {subs_from}{subs_pos}{subs_to} is not supported by
EVCouplings statistics and will be ignored for scoring.")
/kfs2/projects/proteinml/repos/aide_predict/aide_predict/bespoke_models/predicto
rs/evmutation.py:186: UserWarning: Mutation E22e is not supported by EVCouplings
statistics and will be ignored for scoring.
  warnings.warn(f"Mutation {subs_from}{subs_pos}{subs_to} is not supported by
EVCouplings statistics and will be ignored for scoring.")
/kfs2/projects/proteinml/repos/aide_predict/aide_predict/bespoke_models/predicto
rs/evmutation.py:186: UserWarning: Mutation E23e is not supported by EVCouplings
statistics and will be ignored for scoring.
  warnings.warn(f"Mutation {subs_from}{subs_pos}{subs_to} is not supported by
EVCouplings statistics and will be ignored for scoring.")
/kfs2/projects/proteinml/repos/aide_predict/aide_predict/bespoke_models/predicto
rs/evmutation.py:186: UserWarning: Mutation L24l is not supported by EVCouplings
statistics and will be ignored for scoring.
  warnings.warn(f"Mutation {subs_from}{subs_pos}{subs_to} is not supported by
EVCouplings statistics and will be ignored for scoring.")
/kfs2/projects/proteinml/repos/aide_predict/aide_predict/bespoke_models/predicto
rs/evmutation.py:186: UserWarning: Mutation E25e is not supported by EVCouplings
statistics and will be ignored for scoring.
  warnings.warn(f"Mutation {subs_from}{subs_pos}{subs_to} is not supported by
EVCouplings statistics and will be ignored for scoring.")
/kfs2/projects/proteinml/repos/aide_predict/aide_predict/bespoke_models/predicto
rs/evmutation.py:186: UserWarning: Mutation A26a is not supported by EVCouplings
statistics and will be ignored for scoring.
```

```
  warnings.warn(f"Mutation {subs_from}{subs_pos}{subs_to} is not supported by
EVCouplings statistics and will be ignored for scoring.")
/kfs2/projects/proteinml/repos/aide_predict/aide_predict/bespoke_models/predicto
rs/evmutation.py:186: UserWarning: Mutation A27a is not supported by EVCouplings
statistics and will be ignored for scoring.
  warnings.warn(f"Mutation {subs_from}{subs_pos}{subs_to} is not supported by
EVCouplings statistics and will be ignored for scoring.")
/kfs2/projects/proteinml/repos/aide_predict/aide_predict/bespoke_models/predicto
rs/evmutation.py:186: UserWarning: Mutation Y28y is not supported by EVCouplings
statistics and will be ignored for scoring.
  warnings.warn(f"Mutation {subs_from}{subs_pos}{subs_to} is not supported by
EVCouplings statistics and will be ignored for scoring.")
/kfs2/projects/proteinml/repos/aide_predict/aide_predict/bespoke_models/predicto
rs/evmutation.py:186: UserWarning: Mutation E29e is not supported by EVCouplings
statistics and will be ignored for scoring.
  warnings.warn(f"Mutation {subs_from}{subs_pos}{subs_to} is not supported by
EVCouplings statistics and will be ignored for scoring.")
/kfs2/projects/proteinml/repos/aide_predict/aide_predict/bespoke_models/predicto
rs/evmutation.py:186: UserWarning: Mutation G30g is not supported by EVCouplings
statistics and will be ignored for scoring.
  warnings.warn(f"Mutation {subs_from}{subs_pos}{subs_to} is not supported by
EVCouplings statistics and will be ignored for scoring.")
/kfs2/projects/proteinml/repos/aide_predict/aide_predict/bespoke_models/predicto
rs/evmutation.py:186: UserWarning: Mutation I31i is not supported by EVCouplings
statistics and will be ignored for scoring.
  warnings.warn(f"Mutation {subs_from}{subs_pos}{subs_to} is not supported by
EVCouplings statistics and will be ignored for scoring.")
/kfs2/projects/proteinml/repos/aide_predict/aide_predict/bespoke_models/predicto
rs/evmutation.py:186: UserWarning: Mutation M32m is not supported by EVCouplings
statistics and will be ignored for scoring.
  warnings.warn(f"Mutation {subs_from}{subs_pos}{subs_to} is not supported by
EVCouplings statistics and will be ignored for scoring.")
/kfs2/projects/proteinml/repos/aide_predict/aide_predict/bespoke_models/predicto
rs/evmutation.py:186: UserWarning: Mutation K33k is not supported by EVCouplings
statistics and will be ignored for scoring.
  warnings.warn(f"Mutation {subs_from}{subs_pos}{subs_to} is not supported by
EVCouplings statistics and will be ignored for scoring.")
/kfs2/projects/proteinml/repos/aide_predict/aide_predict/bespoke_models/predicto
rs/evmutation.py:186: UserWarning: Mutation D34d is not supported by EVCouplings
statistics and will be ignored for scoring.
  warnings.warn(f"Mutation {subs_from}{subs_pos}{subs_to} is not supported by
EVCouplings statistics and will be ignored for scoring.")
/kfs2/projects/proteinml/repos/aide_predict/aide_predict/bespoke_models/predicto
rs/evmutation.py:186: UserWarning: Mutation E35e is not supported by EVCouplings
statistics and will be ignored for scoring.
  warnings.warn(f"Mutation {subs_from}{subs_pos}{subs_to} is not supported by
EVCouplings statistics and will be ignored for scoring.")
/kfs2/projects/proteinml/repos/aide_predict/aide_predict/bespoke_models/predicto
```

```
rs/evmutation.py:186: UserWarning: Mutation S36s is not supported by EVCouplings
statistics and will be ignored for scoring.
  warnings.warn(f"Mutation {subs_from}{subs_pos}{subs_to} is not supported by
EVCouplings statistics and will be ignored for scoring.")
/kfs2/projects/proteinml/repos/aide_predict/aide_predict/bespoke_models/predicto
rs/evmutation.py:186: UserWarning: Mutation F37f is not supported by EVCouplings
statistics and will be ignored for scoring.
  warnings.warn(f"Mutation {subs_from}{subs_pos}{subs_to} is not supported by
EVCouplings statistics and will be ignored for scoring.")
/kfs2/projects/proteinml/repos/aide_predict/aide_predict/bespoke_models/predicto
rs/evmutation.py:186: UserWarning: Mutation W38w is not supported by EVCouplings
statistics and will be ignored for scoring.
  warnings.warn(f"Mutation {subs_from}{subs_pos}{subs_to} is not supported by
EVCouplings statistics and will be ignored for scoring.")
/kfs2/projects/proteinml/repos/aide_predict/aide_predict/bespoke_models/predicto
rs/evmutation.py:186: UserWarning: Mutation K39k is not supported by EVCouplings
statistics and will be ignored for scoring.
  warnings.warn(f"Mutation {subs_from}{subs_pos}{subs_to} is not supported by
EVCouplings statistics and will be ignored for scoring.")
/kfs2/projects/proteinml/repos/aide_predict/aide_predict/bespoke_models/predicto
rs/evmutation.py:186: UserWarning: Mutation E40e is not supported by EVCouplings
statistics and will be ignored for scoring.
  warnings.warn(f"Mutation {subs_from}{subs_pos}{subs_to} is not supported by
EVCouplings statistics and will be ignored for scoring.")
/kfs2/projects/proteinml/repos/aide_predict/aide_predict/bespoke_models/predicto
rs/evmutation.py:186: UserWarning: Mutation F41f is not supported by EVCouplings
statistics and will be ignored for scoring.
  warnings.warn(f"Mutation {subs_from}{subs_pos}{subs_to} is not supported by
EVCouplings statistics and will be ignored for scoring.")
/kfs2/projects/proteinml/repos/aide_predict/aide_predict/bespoke_models/predicto
rs/evmutation.py:186: UserWarning: Mutation N42n is not supported by EVCouplings
statistics and will be ignored for scoring.
  warnings.warn(f"Mutation {subs_from}{subs_pos}{subs_to} is not supported by
EVCouplings statistics and will be ignored for scoring.")
/kfs2/projects/proteinml/repos/aide_predict/aide_predict/bespoke_models/predicto
rs/evmutation.py:186: UserWarning: Mutation D43d is not supported by EVCouplings
statistics and will be ignored for scoring.
  warnings.warn(f"Mutation {subs_from}{subs_pos}{subs_to} is not supported by
EVCouplings statistics and will be ignored for scoring.")
/kfs2/projects/proteinml/repos/aide_predict/aide_predict/bespoke_models/predicto
rs/evmutation.py:186: UserWarning: Mutation L44l is not supported by EVCouplings
statistics and will be ignored for scoring.
  warnings.warn(f"Mutation {subs_from}{subs_pos}{subs_to} is not supported by
EVCouplings statistics and will be ignored for scoring.")
/kfs2/projects/proteinml/repos/aide_predict/aide_predict/bespoke_models/predicto
rs/evmutation.py:186: UserWarning: Mutation L45l is not supported by EVCouplings
statistics and will be ignored for scoring.
  warnings.warn(f"Mutation {subs_from}{subs_pos}{subs_to} is not supported by
```

```
EVCouplings statistics and will be ignored for scoring.")
/kfs2/projects/proteinml/repos/aide_predict/aide_predict/bespoke_models/predicto
rs/evmutation.py:186: UserWarning: Mutation R46r is not supported by EVCouplings
statistics and will be ignored for scoring.
  warnings.warn(f"Mutation {subs_from}{subs_pos}{subs_to} is not supported by
EVCouplings statistics and will be ignored for scoring.")
/kfs2/projects/proteinml/repos/aide_predict/aide_predict/bespoke_models/predicto
rs/evmutation.py:186: UserWarning: Mutation D47d is not supported by EVCouplings
statistics and will be ignored for scoring.
  warnings.warn(f"Mutation {subs_from}{subs_pos}{subs_to} is not supported by
EVCouplings statistics and will be ignored for scoring.")
/kfs2/projects/proteinml/repos/aide_predict/aide_predict/bespoke_models/predicto
rs/evmutation.py:186: UserWarning: Mutation Y48y is not supported by EVCouplings
statistics and will be ignored for scoring.
  warnings.warn(f"Mutation {subs_from}{subs_pos}{subs_to} is not supported by
EVCouplings statistics and will be ignored for scoring.")
/kfs2/projects/proteinml/repos/aide_predict/aide_predict/bespoke_models/predicto
rs/evmutation.py:186: UserWarning: Mutation D134d is not supported by
EVCouplings statistics and will be ignored for scoring.
  warnings.warn(f"Mutation {subs_from}{subs_pos}{subs_to} is not supported by
EVCouplings statistics and will be ignored for scoring.")
/kfs2/projects/proteinml/repos/aide_predict/aide_predict/bespoke_models/predicto
rs/evmutation.py:186: UserWarning: Mutation I136i is not supported by
EVCouplings statistics and will be ignored for scoring.
  warnings.warn(f"Mutation {subs_from}{subs_pos}{subs_to} is not supported by
EVCouplings statistics and will be ignored for scoring.")
/kfs2/projects/proteinml/repos/aide_predict/aide_predict/bespoke_models/predicto
rs/evmutation.py:186: UserWarning: Mutation R137r is not supported by
EVCouplings statistics and will be ignored for scoring.
  warnings.warn(f"Mutation {subs_from}{subs_pos}{subs_to} is not supported by
EVCouplings statistics and will be ignored for scoring.")
/kfs2/projects/proteinml/repos/aide_predict/aide_predict/bespoke_models/predicto
rs/evmutation.py:186: UserWarning: Mutation Q138q is not supported by
EVCouplings statistics and will be ignored for scoring.
  warnings.warn(f"Mutation {subs_from}{subs_pos}{subs_to} is not supported by
EVCouplings statistics and will be ignored for scoring.")
/kfs2/projects/proteinml/repos/aide_predict/aide_predict/bespoke_models/predicto
rs/evmutation.py:186: UserWarning: Mutation S157s is not supported by
EVCouplings statistics and will be ignored for scoring.
  warnings.warn(f"Mutation {subs_from}{subs_pos}{subs_to} is not supported by
EVCouplings statistics and will be ignored for scoring.")
/kfs2/projects/proteinml/repos/aide_predict/aide_predict/bespoke_models/predicto
rs/evmutation.py:186: UserWarning: Mutation G158g is not supported by
EVCouplings statistics and will be ignored for scoring.
  warnings.warn(f"Mutation {subs_from}{subs_pos}{subs_to} is not supported by
EVCouplings statistics and will be ignored for scoring.")
/kfs2/projects/proteinml/repos/aide_predict/aide_predict/bespoke_models/predicto
rs/evmutation.py:186: UserWarning: Mutation S159s is not supported by
```

```
EVCouplings statistics and will be ignored for scoring.
  warnings.warn(f"Mutation {subs_from}{subs_pos}{subs_to} is not supported by
EVCouplings statistics and will be ignored for scoring.")
/kfs2/projects/proteinml/repos/aide_predict/aide_predict/bespoke_models/predicto
rs/evmutation.py:186: UserWarning: Mutation L177l is not supported by
EVCouplings statistics and will be ignored for scoring.
  warnings.warn(f"Mutation {subs_from}{subs_pos}{subs_to} is not supported by
EVCouplings statistics and will be ignored for scoring.")
/kfs2/projects/proteinml/repos/aide_predict/aide_predict/bespoke_models/predicto
rs/evmutation.py:186: UserWarning: Mutation T179t is not supported by
EVCouplings statistics and will be ignored for scoring.
  warnings.warn(f"Mutation {subs_from}{subs_pos}{subs_to} is not supported by
EVCouplings statistics and will be ignored for scoring.")
/kfs2/projects/proteinml/repos/aide_predict/aide_predict/bespoke_models/predicto
rs/evmutation.py:186: UserWarning: Mutation T180t is not supported by
EVCouplings statistics and will be ignored for scoring.
  warnings.warn(f"Mutation {subs_from}{subs_pos}{subs_to} is not supported by
EVCouplings statistics and will be ignored for scoring.")
/kfs2/projects/proteinml/repos/aide_predict/aide_predict/bespoke_models/predicto
rs/evmutation.py:186: UserWarning: Mutation Y181y is not supported by
EVCouplings statistics and will be ignored for scoring.
  warnings.warn(f"Mutation {subs_from}{subs_pos}{subs_to} is not supported by
EVCouplings statistics and will be ignored for scoring.")
/kfs2/projects/proteinml/repos/aide_predict/aide_predict/bespoke_models/predicto
rs/evmutation.py:186: UserWarning: Mutation V183G is not supported by
EVCouplings statistics and will be ignored for scoring.
  warnings.warn(f"Mutation {subs_from}{subs_pos}{subs_to} is not supported by
EVCouplings statistics and will be ignored for scoring.")
/kfs2/projects/proteinml/repos/aide_predict/aide_predict/bespoke_models/predicto
rs/evmutation.py:186: UserWarning: Mutation F184Y is not supported by
EVCouplings statistics and will be ignored for scoring.
  warnings.warn(f"Mutation {subs_from}{subs_pos}{subs_to} is not supported by
EVCouplings statistics and will be ignored for scoring.")
/kfs2/projects/proteinml/repos/aide_predict/aide_predict/bespoke_models/predicto
rs/evmutation.py:186: UserWarning: Mutation P192p is not supported by
EVCouplings statistics and will be ignored for scoring.
  warnings.warn(f"Mutation {subs_from}{subs_pos}{subs_to} is not supported by
EVCouplings statistics and will be ignored for scoring.")
/kfs2/projects/proteinml/repos/aide_predict/aide_predict/bespoke_models/predicto
rs/evmutation.py:186: UserWarning: Mutation I196i is not supported by
EVCouplings statistics and will be ignored for scoring.
  warnings.warn(f"Mutation {subs_from}{subs_pos}{subs_to} is not supported by
EVCouplings statistics and will be ignored for scoring.")
/kfs2/projects/proteinml/repos/aide_predict/aide_predict/bespoke_models/predicto
rs/evmutation.py:186: UserWarning: Mutation V197v is not supported by
EVCouplings statistics and will be ignored for scoring.
  warnings.warn(f"Mutation {subs_from}{subs_pos}{subs_to} is not supported by
EVCouplings statistics and will be ignored for scoring.")
```

```
/kfs2/projects/proteinml/repos/aide_predict/aide_predict/bespoke_models/predicto
rs/evmutation.py:186: UserWarning: Mutation P213p is not supported by
EVCouplings statistics and will be ignored for scoring.
  warnings.warn(f"Mutation {subs_from}{subs_pos}{subs_to} is not supported by
EVCouplings statistics and will be ignored for scoring.")
/kfs2/projects/proteinml/repos/aide_predict/aide_predict/bespoke_models/predicto
rs/evmutation.py:186: UserWarning: Mutation E214e is not supported by
EVCouplings statistics and will be ignored for scoring.
  warnings.warn(f"Mutation {subs_from}{subs_pos}{subs_to} is not supported by
EVCouplings statistics and will be ignored for scoring.")
/kfs2/projects/proteinml/repos/aide_predict/aide_predict/bespoke_models/predicto
rs/evmutation.py:186: UserWarning: Mutation K215k is not supported by
EVCouplings statistics and will be ignored for scoring.
  warnings.warn(f"Mutation {subs_from}{subs_pos}{subs_to} is not supported by
EVCouplings statistics and will be ignored for scoring.")
/kfs2/projects/proteinml/repos/aide_predict/aide_predict/bespoke_models/predicto
rs/evmutation.py:186: UserWarning: Mutation E216e is not supported by
EVCouplings statistics and will be ignored for scoring.
  warnings.warn(f"Mutation {subs_from}{subs_pos}{subs_to} is not supported by
EVCouplings statistics and will be ignored for scoring.")
/kfs2/projects/proteinml/repos/aide_predict/aide_predict/bespoke_models/predicto
rs/evmutation.py:186: UserWarning: Mutation G217g is not supported by
EVCouplings statistics and will be ignored for scoring.
  warnings.warn(f"Mutation {subs_from}{subs_pos}{subs_to} is not supported by
EVCouplings statistics and will be ignored for scoring.")
/kfs2/projects/proteinml/repos/aide_predict/aide_predict/bespoke_models/predicto
rs/evmutation.py:186: UserWarning: Mutation G260g is not supported by
EVCouplings statistics and will be ignored for scoring.
  warnings.warn(f"Mutation {subs_from}{subs_pos}{subs_to} is not supported by
EVCouplings statistics and will be ignored for scoring.")
/kfs2/projects/proteinml/repos/aide_predict/aide_predict/bespoke_models/predicto
rs/evmutation.py:186: UserWarning: Mutation K261k is not supported by
EVCouplings statistics and will be ignored for scoring.
  warnings.warn(f"Mutation {subs_from}{subs_pos}{subs_to} is not supported by
EVCouplings statistics and will be ignored for scoring.")
/kfs2/projects/proteinml/repos/aide_predict/aide_predict/bespoke_models/predicto
rs/evmutation.py:186: UserWarning: Mutation H262h is not supported by
EVCouplings statistics and will be ignored for scoring.
  warnings.warn(f"Mutation {subs_from}{subs_pos}{subs_to} is not supported by
EVCouplings statistics and will be ignored for scoring.")
/kfs2/projects/proteinml/repos/aide_predict/aide_predict/bespoke_models/predicto
rs/evmutation.py:186: UserWarning: Mutation A263a is not supported by
EVCouplings statistics and will be ignored for scoring.
  warnings.warn(f"Mutation {subs_from}{subs_pos}{subs_to} is not supported by
EVCouplings statistics and will be ignored for scoring.")
/kfs2/projects/proteinml/repos/aide_predict/aide_predict/bespoke_models/predicto
rs/evmutation.py:186: UserWarning: Mutation A264a is not supported by
EVCouplings statistics and will be ignored for scoring.
```

```
  warnings.warn(f"Mutation {subs_from}{subs_pos}{subs_to} is not supported by
EVCouplings statistics and will be ignored for scoring.")
/kfs2/projects/proteinml/repos/aide_predict/aide_predict/bespoke_models/predicto
rs/evmutation.py:186: UserWarning: Mutation H275h is not supported by
EVCouplings statistics and will be ignored for scoring.
  warnings.warn(f"Mutation {subs_from}{subs_pos}{subs_to} is not supported by
EVCouplings statistics and will be ignored for scoring.")
/kfs2/projects/proteinml/repos/aide_predict/aide_predict/bespoke_models/predicto
rs/evmutation.py:186: UserWarning: Mutation G276g is not supported by
EVCouplings statistics and will be ignored for scoring.
  warnings.warn(f"Mutation {subs_from}{subs_pos}{subs_to} is not supported by
EVCouplings statistics and will be ignored for scoring.")
/kfs2/projects/proteinml/repos/aide_predict/aide_predict/bespoke_models/predicto
rs/evmutation.py:186: UserWarning: Mutation S277s is not supported by
EVCouplings statistics and will be ignored for scoring.
  warnings.warn(f"Mutation {subs_from}{subs_pos}{subs_to} is not supported by
EVCouplings statistics and will be ignored for scoring.")
/kfs2/projects/proteinml/repos/aide_predict/aide_predict/bespoke_models/predicto
rs/evmutation.py:186: UserWarning: Mutation K278k is not supported by
EVCouplings statistics and will be ignored for scoring.
  warnings.warn(f"Mutation {subs_from}{subs_pos}{subs_to} is not supported by
EVCouplings statistics and will be ignored for scoring.")
/kfs2/projects/proteinml/repos/aide_predict/aide_predict/bespoke_models/predicto
rs/evmutation.py:186: UserWarning: Mutation T279t is not supported by
EVCouplings statistics and will be ignored for scoring.
  warnings.warn(f"Mutation {subs_from}{subs_pos}{subs_to} is not supported by
EVCouplings statistics and will be ignored for scoring.")
/kfs2/projects/proteinml/repos/aide_predict/aide_predict/bespoke_models/predicto
rs/evmutation.py:186: UserWarning: Mutation F280f is not supported by
EVCouplings statistics and will be ignored for scoring.
  warnings.warn(f"Mutation {subs_from}{subs_pos}{subs_to} is not supported by
EVCouplings statistics and will be ignored for scoring.")
/kfs2/projects/proteinml/repos/aide_predict/aide_predict/bespoke_models/predicto
rs/evmutation.py:186: UserWarning: Mutation V281v is not supported by
EVCouplings statistics and will be ignored for scoring.
  warnings.warn(f"Mutation {subs_from}{subs_pos}{subs_to} is not supported by
EVCouplings statistics and will be ignored for scoring.")
/kfs2/projects/proteinml/repos/aide_predict/aide_predict/bespoke_models/predicto
rs/evmutation.py:186: UserWarning: Mutation L282l is not supported by
EVCouplings statistics and will be ignored for scoring.
  warnings.warn(f"Mutation {subs_from}{subs_pos}{subs_to} is not supported by
EVCouplings statistics and will be ignored for scoring.")
/kfs2/projects/proteinml/repos/aide_predict/aide_predict/bespoke_models/predicto
rs/evmutation.py:186: UserWarning: Mutation Q283q is not supported by
EVCouplings statistics and will be ignored for scoring.
  warnings.warn(f"Mutation {subs_from}{subs_pos}{subs_to} is not supported by
EVCouplings statistics and will be ignored for scoring.")
/kfs2/projects/proteinml/repos/aide_predict/aide_predict/bespoke_models/predicto
```

```
rs/evmutation.py:186: UserWarning: Mutation D284d is not supported by
EVCouplings statistics and will be ignored for scoring.
  warnings.warn(f"Mutation {subs_from}{subs_pos}{subs_to} is not supported by
EVCouplings statistics and will be ignored for scoring.")
/kfs2/projects/proteinml/repos/aide_predict/aide_predict/bespoke_models/predicto
rs/evmutation.py:186: UserWarning: Mutation D285d is not supported by
EVCouplings statistics and will be ignored for scoring.
  warnings.warn(f"Mutation {subs_from}{subs_pos}{subs_to} is not supported by
EVCouplings statistics and will be ignored for scoring.")
/kfs2/projects/proteinml/repos/aide_predict/aide_predict/bespoke_models/predicto
rs/evmutation.py:186: UserWarning: Mutation W286w is not supported by
EVCouplings statistics and will be ignored for scoring.
  warnings.warn(f"Mutation {subs_from}{subs_pos}{subs_to} is not supported by
EVCouplings statistics and will be ignored for scoring.")
/kfs2/projects/proteinml/repos/aide_predict/aide_predict/bespoke_models/predicto
rs/evmutation.py:186: UserWarning: Mutation G287g is not supported by
EVCouplings statistics and will be ignored for scoring.
  warnings.warn(f"Mutation {subs_from}{subs_pos}{subs_to} is not supported by
EVCouplings statistics and will be ignored for scoring.")
/kfs2/projects/proteinml/repos/aide_predict/aide_predict/bespoke_models/predicto
rs/evmutation.py:186: UserWarning: Mutation Q288q is not supported by
EVCouplings statistics and will be ignored for scoring.
  warnings.warn(f"Mutation {subs_from}{subs_pos}{subs_to} is not supported by
EVCouplings statistics and will be ignored for scoring.")
/kfs2/projects/proteinml/repos/aide_predict/aide_predict/bespoke_models/predicto
rs/evmutation.py:186: UserWarning: Mutation V289v is not supported by
EVCouplings statistics and will be ignored for scoring.
  warnings.warn(f"Mutation {subs_from}{subs_pos}{subs_to} is not supported by
EVCouplings statistics and will be ignored for scoring.")
/kfs2/projects/proteinml/repos/aide_predict/aide_predict/bespoke_models/predicto
rs/evmutation.py:186: UserWarning: Mutation Q290q is not supported by
EVCouplings statistics and will be ignored for scoring.
  warnings.warn(f"Mutation {subs_from}{subs_pos}{subs_to} is not supported by
EVCouplings statistics and will be ignored for scoring.")
/kfs2/projects/proteinml/repos/aide_predict/aide_predict/bespoke_models/predicto
rs/evmutation.py:186: UserWarning: Mutation V291v is not supported by
EVCouplings statistics and will be ignored for scoring.
  warnings.warn(f"Mutation {subs_from}{subs_pos}{subs_to} is not supported by
EVCouplings statistics and will be ignored for scoring.")
/kfs2/projects/proteinml/repos/aide_predict/aide_predict/bespoke_models/predicto
rs/evmutation.py:186: UserWarning: Mutation S292s is not supported by
EVCouplings statistics and will be ignored for scoring.
  warnings.warn(f"Mutation {subs_from}{subs_pos}{subs_to} is not supported by
EVCouplings statistics and will be ignored for scoring.")
/kfs2/projects/proteinml/repos/aide_predict/aide_predict/bespoke_models/predicto
rs/evmutation.py:186: UserWarning: Mutation V378v is not supported by
EVCouplings statistics and will be ignored for scoring.
  warnings.warn(f"Mutation {subs_from}{subs_pos}{subs_to} is not supported by
```

```
EVCouplings statistics and will be ignored for scoring.")
/kfs2/projects/proteinml/repos/aide_predict/aide_predict/bespoke_models/predicto
rs/evmutation.py:186: UserWarning: Mutation L379l is not supported by
EVCouplings statistics and will be ignored for scoring.
  warnings.warn(f"Mutation {subs_from}{subs_pos}{subs_to} is not supported by
EVCouplings statistics and will be ignored for scoring.")
/kfs2/projects/proteinml/repos/aide_predict/aide_predict/bespoke_models/predicto
rs/evmutation.py:186: UserWarning: Mutation N380n is not supported by
EVCouplings statistics and will be ignored for scoring.
  warnings.warn(f"Mutation {subs_from}{subs_pos}{subs_to} is not supported by
EVCouplings statistics and will be ignored for scoring.")
/kfs2/projects/proteinml/repos/aide_predict/aide_predict/bespoke_models/predicto
rs/evmutation.py:186: UserWarning: Mutation H381h is not supported by
EVCouplings statistics and will be ignored for scoring.
  warnings.warn(f"Mutation {subs_from}{subs_pos}{subs_to} is not supported by
EVCouplings statistics and will be ignored for scoring.")
/kfs2/projects/proteinml/repos/aide_predict/aide_predict/bespoke_models/predicto
rs/evmutation.py:186: UserWarning: Mutation P382p is not supported by
EVCouplings statistics and will be ignored for scoring.
  warnings.warn(f"Mutation {subs_from}{subs_pos}{subs_to} is not supported by
EVCouplings statistics and will be ignored for scoring.")
/kfs2/projects/proteinml/repos/aide_predict/aide_predict/bespoke_models/predicto
rs/evmutation.py:186: UserWarning: Mutation Y383y is not supported by
EVCouplings statistics and will be ignored for scoring.
  warnings.warn(f"Mutation {subs_from}{subs_pos}{subs_to} is not supported by
EVCouplings statistics and will be ignored for scoring.")
/kfs2/projects/proteinml/repos/aide_predict/aide_predict/bespoke_models/predicto
rs/evmutation.py:186: UserWarning: Mutation V384v is not supported by
EVCouplings statistics and will be ignored for scoring.
  warnings.warn(f"Mutation {subs_from}{subs_pos}{subs_to} is not supported by
EVCouplings statistics and will be ignored for scoring.")
/kfs2/projects/proteinml/repos/aide_predict/aide_predict/bespoke_models/predicto
rs/evmutation.py:186: UserWarning: Mutation R385r is not supported by
EVCouplings statistics and will be ignored for scoring.
  warnings.warn(f"Mutation {subs_from}{subs_pos}{subs_to} is not supported by
EVCouplings statistics and will be ignored for scoring.")
/kfs2/projects/proteinml/repos/aide_predict/aide_predict/bespoke_models/predicto
rs/evmutation.py:186: UserWarning: Mutation E386e is not supported by
EVCouplings statistics and will be ignored for scoring.
  warnings.warn(f"Mutation {subs_from}{subs_pos}{subs_to} is not supported by
EVCouplings statistics and will be ignored for scoring.")
/kfs2/projects/proteinml/repos/aide_predict/aide_predict/bespoke_models/predicto
rs/evmutation.py:186: UserWarning: Mutation R387r is not supported by
EVCouplings statistics and will be ignored for scoring.
  warnings.warn(f"Mutation {subs_from}{subs_pos}{subs_to} is not supported by
EVCouplings statistics and will be ignored for scoring.")
/kfs2/projects/proteinml/repos/aide_predict/aide_predict/bespoke_models/predicto
rs/evmutation.py:186: UserWarning: Mutation I388i is not supported by
```

```
EVCouplings statistics and will be ignored for scoring.
  warnings.warn(f"Mutation {subs_from}{subs_pos}{subs_to} is not supported by
EVCouplings statistics and will be ignored for scoring.")
/kfs2/projects/proteinml/repos/aide_predict/aide_predict/bespoke_models/predicto
rs/evmutation.py:186: UserWarning: Mutation R389r is not supported by
EVCouplings statistics and will be ignored for scoring.
  warnings.warn(f"Mutation {subs_from}{subs_pos}{subs_to} is not supported by
EVCouplings statistics and will be ignored for scoring.")
/kfs2/projects/proteinml/repos/aide_predict/aide_predict/bespoke_models/predicto
rs/evmutation.py:186: UserWarning: Mutation L390l is not supported by
EVCouplings statistics and will be ignored for scoring.
  warnings.warn(f"Mutation {subs_from}{subs_pos}{subs_to} is not supported by
EVCouplings statistics and will be ignored for scoring.")
/kfs2/projects/proteinml/repos/aide_predict/aide_predict/bespoke_models/predicto
rs/evmutation.py:186: UserWarning: Mutation E391e is not supported by
EVCouplings statistics and will be ignored for scoring.
  warnings.warn(f"Mutation {subs_from}{subs_pos}{subs_to} is not supported by
EVCouplings statistics and will be ignored for scoring.")
/kfs2/projects/proteinml/repos/aide_predict/aide_predict/bespoke_models/predicto
rs/evmutation.py:186: UserWarning: Mutation H392h is not supported by
EVCouplings statistics and will be ignored for scoring.
  warnings.warn(f"Mutation {subs_from}{subs_pos}{subs_to} is not supported by
EVCouplings statistics and will be ignored for scoring.")
/kfs2/projects/proteinml/repos/aide_predict/aide_predict/bespoke_models/predicto
rs/evmutation.py:186: UserWarning: Mutation H393h is not supported by
EVCouplings statistics and will be ignored for scoring.
  warnings.warn(f"Mutation {subs_from}{subs_pos}{subs_to} is not supported by
EVCouplings statistics and will be ignored for scoring.")
/kfs2/projects/proteinml/repos/aide_predict/aide_predict/bespoke_models/predicto
rs/evmutation.py:186: UserWarning: Mutation H394h is not supported by
EVCouplings statistics and will be ignored for scoring.
  warnings.warn(f"Mutation {subs_from}{subs_pos}{subs_to} is not supported by
EVCouplings statistics and will be ignored for scoring.")
/kfs2/projects/proteinml/repos/aide_predict/aide_predict/bespoke_models/predicto
rs/evmutation.py:186: UserWarning: Mutation H395h is not supported by
EVCouplings statistics and will be ignored for scoring.
  warnings.warn(f"Mutation {subs_from}{subs_pos}{subs_to} is not supported by
EVCouplings statistics and will be ignored for scoring.")
/kfs2/projects/proteinml/repos/aide_predict/aide_predict/bespoke_models/predicto
rs/evmutation.py:186: UserWarning: Mutation H396h is not supported by
EVCouplings statistics and will be ignored for scoring.
  warnings.warn(f"Mutation {subs_from}{subs_pos}{subs_to} is not supported by
EVCouplings statistics and will be ignored for scoring.")
/kfs2/projects/proteinml/repos/aide_predict/aide_predict/bespoke_models/predicto
rs/evmutation.py:186: UserWarning: Mutation H397h is not supported by
EVCouplings statistics and will be ignored for scoring.
  warnings.warn(f"Mutation {subs_from}{subs_pos}{subs_to} is not supported by
EVCouplings statistics and will be ignored for scoring.")
```

```
/kfs2/projects/proteinml/repos/aide_predict/aide_predict/bespoke_models/predicto
rs/evmutation.py:186: UserWarning: Mutation V183P is not supported by
EVCouplings statistics and will be ignored for scoring.
  warnings.warn(f"Mutation {subs_from}{subs_pos}{subs_to} is not supported by
EVCouplings statistics and will be ignored for scoring.")
/kfs2/projects/proteinml/repos/aide_predict/aide_predict/bespoke_models/predicto
rs/evmutation.py:186: UserWarning: Mutation V183K is not supported by
EVCouplings statistics and will be ignored for scoring.
  warnings.warn(f"Mutation {subs_from}{subs_pos}{subs_to} is not supported by
EVCouplings statistics and will be ignored for scoring.")
/kfs2/projects/proteinml/repos/aide_predict/aide_predict/bespoke_models/predicto
rs/evmutation.py:186: UserWarning: Mutation F184W is not supported by
EVCouplings statistics and will be ignored for scoring.
  warnings.warn(f"Mutation {subs_from}{subs_pos}{subs_to} is not supported by
EVCouplings statistics and will be ignored for scoring.")
/kfs2/projects/proteinml/repos/aide_predict/aide_predict/bespoke_models/predicto
rs/evmutation.py:186: UserWarning: Mutation V183D is not supported by
EVCouplings statistics and will be ignored for scoring.
  warnings.warn(f"Mutation {subs_from}{subs_pos}{subs_to} is not supported by
EVCouplings statistics and will be ignored for scoring.")
/kfs2/projects/proteinml/repos/aide_predict/aide_predict/bespoke_models/predicto
rs/evmutation.py:186: UserWarning: Mutation F184H is not supported by
EVCouplings statistics and will be ignored for scoring.
  warnings.warn(f"Mutation {subs_from}{subs_pos}{subs_to} is not supported by
EVCouplings statistics and will be ignored for scoring.")
/kfs2/projects/proteinml/repos/aide_predict/aide_predict/bespoke_models/predicto
rs/evmutation.py:186: UserWarning: Mutation V183L is not supported by
EVCouplings statistics and will be ignored for scoring.
  warnings.warn(f"Mutation {subs_from}{subs_pos}{subs_to} is not supported by
EVCouplings statistics and will be ignored for scoring.")
/kfs2/projects/proteinml/repos/aide_predict/aide_predict/bespoke_models/predicto
rs/evmutation.py:186: UserWarning: Mutation F184R is not supported by
EVCouplings statistics and will be ignored for scoring.
  warnings.warn(f"Mutation {subs_from}{subs_pos}{subs_to} is not supported by
EVCouplings statistics and will be ignored for scoring.")
/kfs2/projects/proteinml/repos/aide_predict/aide_predict/bespoke_models/predicto
rs/evmutation.py:186: UserWarning: Mutation V183N is not supported by
EVCouplings statistics and will be ignored for scoring.
  warnings.warn(f"Mutation {subs_from}{subs_pos}{subs_to} is not supported by
EVCouplings statistics and will be ignored for scoring.")
/kfs2/projects/proteinml/repos/aide_predict/aide_predict/bespoke_models/predicto
rs/evmutation.py:186: UserWarning: Mutation F184V is not supported by
EVCouplings statistics and will be ignored for scoring.
  warnings.warn(f"Mutation {subs_from}{subs_pos}{subs_to} is not supported by
EVCouplings statistics and will be ignored for scoring.")
/kfs2/projects/proteinml/repos/aide_predict/aide_predict/bespoke_models/predicto
rs/evmutation.py:186: UserWarning: Mutation V183T is not supported by
EVCouplings statistics and will be ignored for scoring.
```

```
  warnings.warn(f"Mutation {subs_from}{subs_pos}{subs_to} is not supported by
EVCouplings statistics and will be ignored for scoring.")
/kfs2/projects/proteinml/repos/aide_predict/aide_predict/bespoke_models/predicto
rs/evmutation.py:186: UserWarning: Mutation F184E is not supported by
EVCouplings statistics and will be ignored for scoring.
  warnings.warn(f"Mutation {subs_from}{subs_pos}{subs_to} is not supported by
EVCouplings statistics and will be ignored for scoring.")
/kfs2/projects/proteinml/repos/aide_predict/aide_predict/bespoke_models/predicto
rs/evmutation.py:186: UserWarning: Mutation V183E is not supported by
EVCouplings statistics and will be ignored for scoring.
  warnings.warn(f"Mutation {subs_from}{subs_pos}{subs_to} is not supported by
EVCouplings statistics and will be ignored for scoring.")
/kfs2/projects/proteinml/repos/aide_predict/aide_predict/bespoke_models/predicto
rs/evmutation.py:186: UserWarning: Mutation V183S is not supported by
EVCouplings statistics and will be ignored for scoring.
  warnings.warn(f"Mutation {subs_from}{subs_pos}{subs_to} is not supported by
EVCouplings statistics and will be ignored for scoring.")
/kfs2/projects/proteinml/repos/aide_predict/aide_predict/bespoke_models/predicto
rs/evmutation.py:186: UserWarning: Mutation F184K is not supported by
EVCouplings statistics and will be ignored for scoring.
  warnings.warn(f"Mutation {subs_from}{subs_pos}{subs_to} is not supported by
EVCouplings statistics and will be ignored for scoring.")
/kfs2/projects/proteinml/repos/aide_predict/aide_predict/bespoke_models/predicto
rs/evmutation.py:186: UserWarning: Mutation V183A is not supported by
EVCouplings statistics and will be ignored for scoring.
  warnings.warn(f"Mutation {subs_from}{subs_pos}{subs_to} is not supported by
EVCouplings statistics and will be ignored for scoring.")
/kfs2/projects/proteinml/repos/aide_predict/aide_predict/bespoke_models/predicto
rs/evmutation.py:186: UserWarning: Mutation F184A is not supported by
EVCouplings statistics and will be ignored for scoring.
  warnings.warn(f"Mutation {subs_from}{subs_pos}{subs_to} is not supported by
EVCouplings statistics and will be ignored for scoring.")
/kfs2/projects/proteinml/repos/aide_predict/aide_predict/bespoke_models/predicto
rs/evmutation.py:186: UserWarning: Mutation F184P is not supported by
EVCouplings statistics and will be ignored for scoring.
  warnings.warn(f"Mutation {subs_from}{subs_pos}{subs_to} is not supported by
EVCouplings statistics and will be ignored for scoring.")
/kfs2/projects/proteinml/repos/aide_predict/aide_predict/bespoke_models/predicto
rs/evmutation.py:186: UserWarning: Mutation F184M is not supported by
EVCouplings statistics and will be ignored for scoring.
  warnings.warn(f"Mutation {subs_from}{subs_pos}{subs_to} is not supported by
EVCouplings statistics and will be ignored for scoring.")
/kfs2/projects/proteinml/repos/aide_predict/aide_predict/bespoke_models/predicto
rs/evmutation.py:186: UserWarning: Mutation F184D is not supported by
EVCouplings statistics and will be ignored for scoring.
  warnings.warn(f"Mutation {subs_from}{subs_pos}{subs_to} is not supported by
EVCouplings statistics and will be ignored for scoring.")
/kfs2/projects/proteinml/repos/aide_predict/aide_predict/bespoke_models/predicto
```

```
rs/evmutation.py:186: UserWarning: Mutation F184L is not supported by
EVCouplings statistics and will be ignored for scoring.
  warnings.warn(f"Mutation {subs_from}{subs_pos}{subs_to} is not supported by
EVCouplings statistics and will be ignored for scoring.")
/kfs2/projects/proteinml/repos/aide_predict/aide_predict/bespoke_models/predicto
rs/evmutation.py:186: UserWarning: Mutation V183I is not supported by
EVCouplings statistics and will be ignored for scoring.
  warnings.warn(f"Mutation {subs_from}{subs_pos}{subs_to} is not supported by
EVCouplings statistics and will be ignored for scoring.")
/kfs2/projects/proteinml/repos/aide_predict/aide_predict/bespoke_models/predicto
rs/evmutation.py:186: UserWarning: Mutation F184T is not supported by
EVCouplings statistics and will be ignored for scoring.
  warnings.warn(f"Mutation {subs_from}{subs_pos}{subs_to} is not supported by
EVCouplings statistics and will be ignored for scoring.")
/kfs2/projects/proteinml/repos/aide_predict/aide_predict/bespoke_models/predicto
rs/evmutation.py:186: UserWarning: Mutation V183F is not supported by
EVCouplings statistics and will be ignored for scoring.
  warnings.warn(f"Mutation {subs_from}{subs_pos}{subs_to} is not supported by
EVCouplings statistics and will be ignored for scoring.")
/kfs2/projects/proteinml/repos/aide_predict/aide_predict/bespoke_models/predicto
rs/evmutation.py:186: UserWarning: Mutation F184Q is not supported by
EVCouplings statistics and will be ignored for scoring.
  warnings.warn(f"Mutation {subs_from}{subs_pos}{subs_to} is not supported by
EVCouplings statistics and will be ignored for scoring.")
/kfs2/projects/proteinml/repos/aide_predict/aide_predict/bespoke_models/predicto
rs/evmutation.py:186: UserWarning: Mutation V183H is not supported by
EVCouplings statistics and will be ignored for scoring.
  warnings.warn(f"Mutation {subs_from}{subs_pos}{subs_to} is not supported by
EVCouplings statistics and will be ignored for scoring.")
/kfs2/projects/proteinml/repos/aide_predict/aide_predict/bespoke_models/predicto
rs/evmutation.py:186: UserWarning: Mutation F184I is not supported by
EVCouplings statistics and will be ignored for scoring.
  warnings.warn(f"Mutation {subs_from}{subs_pos}{subs_to} is not supported by
EVCouplings statistics and will be ignored for scoring.")
/kfs2/projects/proteinml/repos/aide_predict/aide_predict/bespoke_models/predicto
rs/evmutation.py:186: UserWarning: Mutation V183R is not supported by
EVCouplings statistics and will be ignored for scoring.
  warnings.warn(f"Mutation {subs_from}{subs_pos}{subs_to} is not supported by
EVCouplings statistics and will be ignored for scoring.")
/kfs2/projects/proteinml/repos/aide_predict/aide_predict/bespoke_models/predicto
rs/evmutation.py:186: UserWarning: Mutation F184G is not supported by
EVCouplings statistics and will be ignored for scoring.
  warnings.warn(f"Mutation {subs_from}{subs_pos}{subs_to} is not supported by
EVCouplings statistics and will be ignored for scoring.")
/kfs2/projects/proteinml/repos/aide_predict/aide_predict/bespoke_models/predicto
rs/evmutation.py:186: UserWarning: Mutation F184C is not supported by
EVCouplings statistics and will be ignored for scoring.
  warnings.warn(f"Mutation {subs_from}{subs_pos}{subs_to} is not supported by
```

```
EVCouplings statistics and will be ignored for scoring.")
/kfs2/projects/proteinml/repos/aide_predict/aide_predict/bespoke_models/predicto
rs/evmutation.py:186: UserWarning: Mutation V183Y is not supported by
EVCouplings statistics and will be ignored for scoring.
  warnings.warn(f"Mutation {subs_from}{subs_pos}{subs_to} is not supported by
EVCouplings statistics and will be ignored for scoring.")
/kfs2/projects/proteinml/repos/aide_predict/aide_predict/bespoke_models/predicto
rs/evmutation.py:186: UserWarning: Mutation V183C is not supported by
EVCouplings statistics and will be ignored for scoring.
  warnings.warn(f"Mutation {subs_from}{subs_pos}{subs_to} is not supported by
EVCouplings statistics and will be ignored for scoring.")
/kfs2/projects/proteinml/repos/aide_predict/aide_predict/bespoke_models/predicto
rs/evmutation.py:186: UserWarning: Mutation V183W is not supported by
EVCouplings statistics and will be ignored for scoring.
  warnings.warn(f"Mutation {subs_from}{subs_pos}{subs_to} is not supported by
EVCouplings statistics and will be ignored for scoring.")
/kfs2/projects/proteinml/repos/aide_predict/aide_predict/bespoke_models/predicto
rs/evmutation.py:186: UserWarning: Mutation F184N is not supported by
EVCouplings statistics and will be ignored for scoring.
  warnings.warn(f"Mutation {subs_from}{subs_pos}{subs_to} is not supported by
EVCouplings statistics and will be ignored for scoring.")
/kfs2/projects/proteinml/repos/aide_predict/aide_predict/bespoke_models/predicto
rs/evmutation.py:186: UserWarning: Mutation F184S is not supported by
EVCouplings statistics and will be ignored for scoring.
  warnings.warn(f"Mutation {subs_from}{subs_pos}{subs_to} is not supported by
EVCouplings statistics and will be ignored for scoring.")
/kfs2/projects/proteinml/repos/aide_predict/aide_predict/bespoke_models/predicto
rs/evmutation.py:186: UserWarning: Mutation V183Q is not supported by
EVCouplings statistics and will be ignored for scoring.
  warnings.warn(f"Mutation {subs_from}{subs_pos}{subs_to} is not supported by
EVCouplings statistics and will be ignored for scoring.")
/kfs2/projects/proteinml/repos/aide_predict/aide_predict/bespoke_models/predicto
rs/evmutation.py:186: UserWarning: Mutation V183M is not supported by
EVCouplings statistics and will be ignored for scoring.
  warnings.warn(f"Mutation {subs_from}{subs_pos}{subs_to} is not supported by
EVCouplings statistics and will be ignored for scoring.")
100%|      | 159129/159129 [00:23<00:00, 6766.61it/s]
100%|      | 1/1 [00:00<00:00, 7476.48it/s]
```

[25]:
```
X_ = np.concatenate([X_ohe, X_evc], axis=1)
```

[26]:
```
evc_ohe_linear_scores = hyperopt_and_scoring(linear_nopca, X_, y,
 ↪linear_param_space)
joblib.dump(evc_ohe_linear_scores, 'evc_ohe_linear_scores.joblib')
```

[26]:
```
['evc_ohe_linear_scores.joblib']
```

1.6.2 4.2 EVC plus one hot MLP

[27]:
```
evc_ohe_mlp_scores = hyperopt_and_scoring(mlp_nopca, X_, y, mlp_param_space)
joblib.dump(evc_ohe_mlp_scores, 'evc_ohe_mlp_scores.joblib')
```

```
/projects/proteinml/.links/miniconda3/envs/aidep/lib/python3.10/site-
packages/sklearn/neural_network/_multilayer_perceptron.py:690:
ConvergenceWarning: Stochastic Optimizer: Maximum iterations (1000) reached and
the optimization hasn't converged yet.
  warnings.warn(
/projects/proteinml/.links/miniconda3/envs/aidep/lib/python3.10/site-
packages/sklearn/neural_network/_multilayer_perceptron.py:690:
ConvergenceWarning: Stochastic Optimizer: Maximum iterations (1000) reached and
the optimization hasn't converged yet.
  warnings.warn(
/projects/proteinml/.links/miniconda3/envs/aidep/lib/python3.10/site-
packages/sklearn/neural_network/_multilayer_perceptron.py:690:
ConvergenceWarning: Stochastic Optimizer: Maximum iterations (1000) reached and
the optimization hasn't converged yet.
  warnings.warn(
/projects/proteinml/.links/miniconda3/envs/aidep/lib/python3.10/site-
packages/sklearn/neural_network/_multilayer_perceptron.py:690:
ConvergenceWarning: Stochastic Optimizer: Maximum iterations (1000) reached and
the optimization hasn't converged yet.
  warnings.warn(
/projects/proteinml/.links/miniconda3/envs/aidep/lib/python3.10/site-
packages/sklearn/neural_network/_multilayer_perceptron.py:690:
ConvergenceWarning: Stochastic Optimizer: Maximum iterations (1000) reached and
the optimization hasn't converged yet.
  warnings.warn(
/projects/proteinml/.links/miniconda3/envs/aidep/lib/python3.10/site-
packages/sklearn/neural_network/_multilayer_perceptron.py:690:
ConvergenceWarning: Stochastic Optimizer: Maximum iterations (1000) reached and
the optimization hasn't converged yet.
  warnings.warn(
/projects/proteinml/.links/miniconda3/envs/aidep/lib/python3.10/site-
packages/sklearn/neural_network/_multilayer_perceptron.py:690:
ConvergenceWarning: Stochastic Optimizer: Maximum iterations (1000) reached and
the optimization hasn't converged yet.
  warnings.warn(
/projects/proteinml/.links/miniconda3/envs/aidep/lib/python3.10/site-
packages/sklearn/neural_network/_multilayer_perceptron.py:690:
ConvergenceWarning: Stochastic Optimizer: Maximum iterations (1000) reached and
the optimization hasn't converged yet.
  warnings.warn(
/projects/proteinml/.links/miniconda3/envs/aidep/lib/python3.10/site-
packages/sklearn/neural_network/_multilayer_perceptron.py:690:
ConvergenceWarning: Stochastic Optimizer: Maximum iterations (1000) reached and
```

```
the optimization hasn't converged yet.
  warnings.warn(
/projects/proteinml/.links/miniconda3/envs/aidep/lib/python3.10/site-
packages/sklearn/neural_network/_multilayer_perceptron.py:690:
ConvergenceWarning: Stochastic Optimizer: Maximum iterations (1000) reached and
the optimization hasn't converged yet.
  warnings.warn(
/projects/proteinml/.links/miniconda3/envs/aidep/lib/python3.10/site-
packages/sklearn/neural_network/_multilayer_perceptron.py:690:
ConvergenceWarning: Stochastic Optimizer: Maximum iterations (1000) reached and
the optimization hasn't converged yet.
  warnings.warn(
/projects/proteinml/.links/miniconda3/envs/aidep/lib/python3.10/site-
packages/sklearn/neural_network/_multilayer_perceptron.py:690:
ConvergenceWarning: Stochastic Optimizer: Maximum iterations (1000) reached and
the optimization hasn't converged yet.
  warnings.warn(
/projects/proteinml/.links/miniconda3/envs/aidep/lib/python3.10/site-
packages/sklearn/neural_network/_multilayer_perceptron.py:690:
ConvergenceWarning: Stochastic Optimizer: Maximum iterations (1000) reached and
the optimization hasn't converged yet.
  warnings.warn(
/projects/proteinml/.links/miniconda3/envs/aidep/lib/python3.10/site-
packages/sklearn/neural_network/_multilayer_perceptron.py:690:
ConvergenceWarning: Stochastic Optimizer: Maximum iterations (1000) reached and
the optimization hasn't converged yet.
  warnings.warn(
/projects/proteinml/.links/miniconda3/envs/aidep/lib/python3.10/site-
packages/sklearn/neural_network/_multilayer_perceptron.py:690:
ConvergenceWarning: Stochastic Optimizer: Maximum iterations (1000) reached and
the optimization hasn't converged yet.
  warnings.warn(
/projects/proteinml/.links/miniconda3/envs/aidep/lib/python3.10/site-
packages/sklearn/neural_network/_multilayer_perceptron.py:690:
ConvergenceWarning: Stochastic Optimizer: Maximum iterations (1000) reached and
the optimization hasn't converged yet.
  warnings.warn(
/projects/proteinml/.links/miniconda3/envs/aidep/lib/python3.10/site-
packages/sklearn/neural_network/_multilayer_perceptron.py:690:
ConvergenceWarning: Stochastic Optimizer: Maximum iterations (1000) reached and
the optimization hasn't converged yet.
  warnings.warn(
/projects/proteinml/.links/miniconda3/envs/aidep/lib/python3.10/site-
packages/sklearn/neural_network/_multilayer_perceptron.py:690:
ConvergenceWarning: Stochastic Optimizer: Maximum iterations (1000) reached and
the optimization hasn't converged yet.
  warnings.warn(
/projects/proteinml/.links/miniconda3/envs/aidep/lib/python3.10/site-
```

```
packages/sklearn/neural_network/_multilayer_perceptron.py:690:
ConvergenceWarning: Stochastic Optimizer: Maximum iterations (1000) reached and
the optimization hasn't converged yet.
  warnings.warn(
/projects/proteinml/.links/miniconda3/envs/aidep/lib/python3.10/site-
packages/sklearn/neural_network/_multilayer_perceptron.py:690:
ConvergenceWarning: Stochastic Optimizer: Maximum iterations (1000) reached and
the optimization hasn't converged yet.
  warnings.warn(
/projects/proteinml/.links/miniconda3/envs/aidep/lib/python3.10/site-
packages/sklearn/neural_network/_multilayer_perceptron.py:690:
ConvergenceWarning: Stochastic Optimizer: Maximum iterations (1000) reached and
the optimization hasn't converged yet.
  warnings.warn(
/projects/proteinml/.links/miniconda3/envs/aidep/lib/python3.10/site-
packages/sklearn/neural_network/_multilayer_perceptron.py:690:
ConvergenceWarning: Stochastic Optimizer: Maximum iterations (1000) reached and
the optimization hasn't converged yet.
  warnings.warn(
/projects/proteinml/.links/miniconda3/envs/aidep/lib/python3.10/site-
packages/sklearn/neural_network/_multilayer_perceptron.py:690:
ConvergenceWarning: Stochastic Optimizer: Maximum iterations (1000) reached and
the optimization hasn't converged yet.
  warnings.warn(
/projects/proteinml/.links/miniconda3/envs/aidep/lib/python3.10/site-
packages/sklearn/neural_network/_multilayer_perceptron.py:690:
ConvergenceWarning: Stochastic Optimizer: Maximum iterations (1000) reached and
the optimization hasn't converged yet.
  warnings.warn(
/projects/proteinml/.links/miniconda3/envs/aidep/lib/python3.10/site-
packages/sklearn/neural_network/_multilayer_perceptron.py:690:
ConvergenceWarning: Stochastic Optimizer: Maximum iterations (1000) reached and
the optimization hasn't converged yet.
  warnings.warn(
/projects/proteinml/.links/miniconda3/envs/aidep/lib/python3.10/site-
packages/sklearn/neural_network/_multilayer_perceptron.py:690:
ConvergenceWarning: Stochastic Optimizer: Maximum iterations (1000) reached and
the optimization hasn't converged yet.
  warnings.warn(
/projects/proteinml/.links/miniconda3/envs/aidep/lib/python3.10/site-
packages/sklearn/neural_network/_multilayer_perceptron.py:690:
ConvergenceWarning: Stochastic Optimizer: Maximum iterations (1000) reached and
the optimization hasn't converged yet.
  warnings.warn(
/projects/proteinml/.links/miniconda3/envs/aidep/lib/python3.10/site-
packages/sklearn/neural_network/_multilayer_perceptron.py:690:
ConvergenceWarning: Stochastic Optimizer: Maximum iterations (1000) reached and
the optimization hasn't converged yet.
```

```
  warnings.warn(
/projects/proteinml/.links/miniconda3/envs/aidep/lib/python3.10/site-
packages/sklearn/neural_network/_multilayer_perceptron.py:690:
ConvergenceWarning: Stochastic Optimizer: Maximum iterations (1000) reached and
the optimization hasn't converged yet.
  warnings.warn(
/projects/proteinml/.links/miniconda3/envs/aidep/lib/python3.10/site-
packages/sklearn/neural_network/_multilayer_perceptron.py:690:
ConvergenceWarning: Stochastic Optimizer: Maximum iterations (1000) reached and
the optimization hasn't converged yet.
  warnings.warn(
/projects/proteinml/.links/miniconda3/envs/aidep/lib/python3.10/site-
packages/sklearn/neural_network/_multilayer_perceptron.py:690:
ConvergenceWarning: Stochastic Optimizer: Maximum iterations (1000) reached and
the optimization hasn't converged yet.
  warnings.warn(
/projects/proteinml/.links/miniconda3/envs/aidep/lib/python3.10/site-
packages/sklearn/neural_network/_multilayer_perceptron.py:690:
ConvergenceWarning: Stochastic Optimizer: Maximum iterations (1000) reached and
the optimization hasn't converged yet.
  warnings.warn(
/projects/proteinml/.links/miniconda3/envs/aidep/lib/python3.10/site-
packages/sklearn/neural_network/_multilayer_perceptron.py:690:
ConvergenceWarning: Stochastic Optimizer: Maximum iterations (1000) reached and
the optimization hasn't converged yet.
  warnings.warn(
/projects/proteinml/.links/miniconda3/envs/aidep/lib/python3.10/site-
packages/sklearn/neural_network/_multilayer_perceptron.py:690:
ConvergenceWarning: Stochastic Optimizer: Maximum iterations (1000) reached and
the optimization hasn't converged yet.
  warnings.warn(
/projects/proteinml/.links/miniconda3/envs/aidep/lib/python3.10/site-
packages/sklearn/neural_network/_multilayer_perceptron.py:690:
ConvergenceWarning: Stochastic Optimizer: Maximum iterations (1000) reached and
the optimization hasn't converged yet.
  warnings.warn(
/projects/proteinml/.links/miniconda3/envs/aidep/lib/python3.10/site-
packages/sklearn/neural_network/_multilayer_perceptron.py:690:
ConvergenceWarning: Stochastic Optimizer: Maximum iterations (1000) reached and
the optimization hasn't converged yet.
  warnings.warn(
/projects/proteinml/.links/miniconda3/envs/aidep/lib/python3.10/site-
packages/sklearn/neural_network/_multilayer_perceptron.py:690:
ConvergenceWarning: Stochastic Optimizer: Maximum iterations (1000) reached and
the optimization hasn't converged yet.
  warnings.warn(
/projects/proteinml/.links/miniconda3/envs/aidep/lib/python3.10/site-
packages/sklearn/neural_network/_multilayer_perceptron.py:690:
```

```
ConvergenceWarning: Stochastic Optimizer: Maximum iterations (1000) reached and
the optimization hasn't converged yet.
  warnings.warn(
/projects/proteinml/.links/miniconda3/envs/aidep/lib/python3.10/site-
packages/sklearn/neural_network/_multilayer_perceptron.py:690:
ConvergenceWarning: Stochastic Optimizer: Maximum iterations (1000) reached and
the optimization hasn't converged yet.
  warnings.warn(
/projects/proteinml/.links/miniconda3/envs/aidep/lib/python3.10/site-
packages/sklearn/neural_network/_multilayer_perceptron.py:690:
ConvergenceWarning: Stochastic Optimizer: Maximum iterations (1000) reached and
the optimization hasn't converged yet.
  warnings.warn(
/projects/proteinml/.links/miniconda3/envs/aidep/lib/python3.10/site-
packages/sklearn/neural_network/_multilayer_perceptron.py:690:
ConvergenceWarning: Stochastic Optimizer: Maximum iterations (1000) reached and
the optimization hasn't converged yet.
  warnings.warn(
/projects/proteinml/.links/miniconda3/envs/aidep/lib/python3.10/site-
packages/sklearn/neural_network/_multilayer_perceptron.py:690:
ConvergenceWarning: Stochastic Optimizer: Maximum iterations (1000) reached and
the optimization hasn't converged yet.
  warnings.warn(
/projects/proteinml/.links/miniconda3/envs/aidep/lib/python3.10/site-
packages/sklearn/neural_network/_multilayer_perceptron.py:690:
ConvergenceWarning: Stochastic Optimizer: Maximum iterations (1000) reached and
the optimization hasn't converged yet.
  warnings.warn(
/projects/proteinml/.links/miniconda3/envs/aidep/lib/python3.10/site-
packages/sklearn/neural_network/_multilayer_perceptron.py:690:
ConvergenceWarning: Stochastic Optimizer: Maximum iterations (1000) reached and
the optimization hasn't converged yet.
  warnings.warn(
/projects/proteinml/.links/miniconda3/envs/aidep/lib/python3.10/site-
packages/sklearn/neural_network/_multilayer_perceptron.py:690:
ConvergenceWarning: Stochastic Optimizer: Maximum iterations (1000) reached and
the optimization hasn't converged yet.
  warnings.warn(
/projects/proteinml/.links/miniconda3/envs/aidep/lib/python3.10/site-
packages/sklearn/neural_network/_multilayer_perceptron.py:690:
ConvergenceWarning: Stochastic Optimizer: Maximum iterations (1000) reached and
the optimization hasn't converged yet.
  warnings.warn(
/projects/proteinml/.links/miniconda3/envs/aidep/lib/python3.10/site-
packages/sklearn/neural_network/_multilayer_perceptron.py:690:
ConvergenceWarning: Stochastic Optimizer: Maximum iterations (1000) reached and
the optimization hasn't converged yet.
  warnings.warn(
```

```
/projects/proteinml/.links/miniconda3/envs/aidep/lib/python3.10/site-
packages/sklearn/neural_network/_multilayer_perceptron.py:690:
ConvergenceWarning: Stochastic Optimizer: Maximum iterations (1000) reached and
the optimization hasn't converged yet.
  warnings.warn(
/projects/proteinml/.links/miniconda3/envs/aidep/lib/python3.10/site-
packages/sklearn/neural_network/_multilayer_perceptron.py:690:
ConvergenceWarning: Stochastic Optimizer: Maximum iterations (1000) reached and
the optimization hasn't converged yet.
  warnings.warn(
/projects/proteinml/.links/miniconda3/envs/aidep/lib/python3.10/site-
packages/sklearn/neural_network/_multilayer_perceptron.py:690:
ConvergenceWarning: Stochastic Optimizer: Maximum iterations (1000) reached and
the optimization hasn't converged yet.
  warnings.warn(
/projects/proteinml/.links/miniconda3/envs/aidep/lib/python3.10/site-
packages/sklearn/neural_network/_multilayer_perceptron.py:690:
ConvergenceWarning: Stochastic Optimizer: Maximum iterations (1000) reached and
the optimization hasn't converged yet.
  warnings.warn(
/projects/proteinml/.links/miniconda3/envs/aidep/lib/python3.10/site-
packages/sklearn/neural_network/_multilayer_perceptron.py:690:
ConvergenceWarning: Stochastic Optimizer: Maximum iterations (1000) reached and
the optimization hasn't converged yet.
  warnings.warn(
/projects/proteinml/.links/miniconda3/envs/aidep/lib/python3.10/site-
packages/sklearn/neural_network/_multilayer_perceptron.py:690:
ConvergenceWarning: Stochastic Optimizer: Maximum iterations (1000) reached and
the optimization hasn't converged yet.
  warnings.warn(
/projects/proteinml/.links/miniconda3/envs/aidep/lib/python3.10/site-
packages/sklearn/neural_network/_multilayer_perceptron.py:690:
ConvergenceWarning: Stochastic Optimizer: Maximum iterations (1000) reached and
the optimization hasn't converged yet.
  warnings.warn(
/projects/proteinml/.links/miniconda3/envs/aidep/lib/python3.10/site-
packages/sklearn/neural_network/_multilayer_perceptron.py:690:
ConvergenceWarning: Stochastic Optimizer: Maximum iterations (1000) reached and
the optimization hasn't converged yet.
  warnings.warn(
/projects/proteinml/.links/miniconda3/envs/aidep/lib/python3.10/site-
packages/sklearn/neural_network/_multilayer_perceptron.py:690:
ConvergenceWarning: Stochastic Optimizer: Maximum iterations (1000) reached and
the optimization hasn't converged yet.
  warnings.warn(
/projects/proteinml/.links/miniconda3/envs/aidep/lib/python3.10/site-
packages/sklearn/neural_network/_multilayer_perceptron.py:690:
ConvergenceWarning: Stochastic Optimizer: Maximum iterations (1000) reached and
```

```
the optimization hasn't converged yet.
  warnings.warn(
/projects/proteinml/.links/miniconda3/envs/aidep/lib/python3.10/site-
packages/sklearn/neural_network/_multilayer_perceptron.py:690:
ConvergenceWarning: Stochastic Optimizer: Maximum iterations (1000) reached and
the optimization hasn't converged yet.
  warnings.warn(
/projects/proteinml/.links/miniconda3/envs/aidep/lib/python3.10/site-
packages/sklearn/neural_network/_multilayer_perceptron.py:690:
ConvergenceWarning: Stochastic Optimizer: Maximum iterations (1000) reached and
the optimization hasn't converged yet.
  warnings.warn(
/projects/proteinml/.links/miniconda3/envs/aidep/lib/python3.10/site-
packages/sklearn/neural_network/_multilayer_perceptron.py:690:
ConvergenceWarning: Stochastic Optimizer: Maximum iterations (1000) reached and
the optimization hasn't converged yet.
  warnings.warn(
/projects/proteinml/.links/miniconda3/envs/aidep/lib/python3.10/site-
packages/sklearn/neural_network/_multilayer_perceptron.py:690:
ConvergenceWarning: Stochastic Optimizer: Maximum iterations (1000) reached and
the optimization hasn't converged yet.
  warnings.warn(
/projects/proteinml/.links/miniconda3/envs/aidep/lib/python3.10/site-
packages/sklearn/neural_network/_multilayer_perceptron.py:690:
ConvergenceWarning: Stochastic Optimizer: Maximum iterations (1000) reached and
the optimization hasn't converged yet.
  warnings.warn(
/projects/proteinml/.links/miniconda3/envs/aidep/lib/python3.10/site-
packages/sklearn/neural_network/_multilayer_perceptron.py:690:
ConvergenceWarning: Stochastic Optimizer: Maximum iterations (1000) reached and
the optimization hasn't converged yet.
  warnings.warn(
/projects/proteinml/.links/miniconda3/envs/aidep/lib/python3.10/site-
packages/sklearn/neural_network/_multilayer_perceptron.py:690:
ConvergenceWarning: Stochastic Optimizer: Maximum iterations (1000) reached and
the optimization hasn't converged yet.
  warnings.warn(
/projects/proteinml/.links/miniconda3/envs/aidep/lib/python3.10/site-
packages/sklearn/neural_network/_multilayer_perceptron.py:690:
ConvergenceWarning: Stochastic Optimizer: Maximum iterations (1000) reached and
the optimization hasn't converged yet.
  warnings.warn(
/projects/proteinml/.links/miniconda3/envs/aidep/lib/python3.10/site-
packages/sklearn/neural_network/_multilayer_perceptron.py:690:
ConvergenceWarning: Stochastic Optimizer: Maximum iterations (1000) reached and
the optimization hasn't converged yet.
  warnings.warn(
/projects/proteinml/.links/miniconda3/envs/aidep/lib/python3.10/site-
```

```
packages/sklearn/neural_network/_multilayer_perceptron.py:690:
ConvergenceWarning: Stochastic Optimizer: Maximum iterations (1000) reached and
the optimization hasn't converged yet.
  warnings.warn(
/projects/proteinml/.links/miniconda3/envs/aidep/lib/python3.10/site-
packages/sklearn/neural_network/_multilayer_perceptron.py:690:
ConvergenceWarning: Stochastic Optimizer: Maximum iterations (1000) reached and
the optimization hasn't converged yet.
  warnings.warn(
/projects/proteinml/.links/miniconda3/envs/aidep/lib/python3.10/site-
packages/sklearn/neural_network/_multilayer_perceptron.py:690:
ConvergenceWarning: Stochastic Optimizer: Maximum iterations (1000) reached and
the optimization hasn't converged yet.
  warnings.warn(
/projects/proteinml/.links/miniconda3/envs/aidep/lib/python3.10/site-
packages/sklearn/neural_network/_multilayer_perceptron.py:690:
ConvergenceWarning: Stochastic Optimizer: Maximum iterations (1000) reached and
the optimization hasn't converged yet.
  warnings.warn(
/projects/proteinml/.links/miniconda3/envs/aidep/lib/python3.10/site-
packages/sklearn/neural_network/_multilayer_perceptron.py:690:
ConvergenceWarning: Stochastic Optimizer: Maximum iterations (1000) reached and
the optimization hasn't converged yet.
  warnings.warn(
/projects/proteinml/.links/miniconda3/envs/aidep/lib/python3.10/site-
packages/sklearn/neural_network/_multilayer_perceptron.py:690:
ConvergenceWarning: Stochastic Optimizer: Maximum iterations (1000) reached and
the optimization hasn't converged yet.
  warnings.warn(
/projects/proteinml/.links/miniconda3/envs/aidep/lib/python3.10/site-
packages/sklearn/neural_network/_multilayer_perceptron.py:690:
ConvergenceWarning: Stochastic Optimizer: Maximum iterations (1000) reached and
the optimization hasn't converged yet.
  warnings.warn(
/projects/proteinml/.links/miniconda3/envs/aidep/lib/python3.10/site-
packages/sklearn/neural_network/_multilayer_perceptron.py:690:
ConvergenceWarning: Stochastic Optimizer: Maximum iterations (1000) reached and
the optimization hasn't converged yet.
  warnings.warn(
/projects/proteinml/.links/miniconda3/envs/aidep/lib/python3.10/site-
packages/sklearn/neural_network/_multilayer_perceptron.py:690:
ConvergenceWarning: Stochastic Optimizer: Maximum iterations (1000) reached and
the optimization hasn't converged yet.
  warnings.warn(
/projects/proteinml/.links/miniconda3/envs/aidep/lib/python3.10/site-
packages/sklearn/neural_network/_multilayer_perceptron.py:690:
ConvergenceWarning: Stochastic Optimizer: Maximum iterations (1000) reached and
the optimization hasn't converged yet.
```

```
  warnings.warn(
/projects/proteinml/.links/miniconda3/envs/aidep/lib/python3.10/site-
packages/sklearn/neural_network/_multilayer_perceptron.py:690:
ConvergenceWarning: Stochastic Optimizer: Maximum iterations (1000) reached and
the optimization hasn't converged yet.
  warnings.warn(
/projects/proteinml/.links/miniconda3/envs/aidep/lib/python3.10/site-
packages/sklearn/neural_network/_multilayer_perceptron.py:690:
ConvergenceWarning: Stochastic Optimizer: Maximum iterations (1000) reached and
the optimization hasn't converged yet.
  warnings.warn(
/projects/proteinml/.links/miniconda3/envs/aidep/lib/python3.10/site-
packages/sklearn/neural_network/_multilayer_perceptron.py:690:
ConvergenceWarning: Stochastic Optimizer: Maximum iterations (1000) reached and
the optimization hasn't converged yet.
  warnings.warn(
/projects/proteinml/.links/miniconda3/envs/aidep/lib/python3.10/site-
packages/sklearn/neural_network/_multilayer_perceptron.py:690:
ConvergenceWarning: Stochastic Optimizer: Maximum iterations (1000) reached and
the optimization hasn't converged yet.
  warnings.warn(
/projects/proteinml/.links/miniconda3/envs/aidep/lib/python3.10/site-
packages/sklearn/neural_network/_multilayer_perceptron.py:690:
ConvergenceWarning: Stochastic Optimizer: Maximum iterations (1000) reached and
the optimization hasn't converged yet.
  warnings.warn(
/projects/proteinml/.links/miniconda3/envs/aidep/lib/python3.10/site-
packages/sklearn/neural_network/_multilayer_perceptron.py:690:
ConvergenceWarning: Stochastic Optimizer: Maximum iterations (1000) reached and
the optimization hasn't converged yet.
  warnings.warn(
/projects/proteinml/.links/miniconda3/envs/aidep/lib/python3.10/site-
packages/sklearn/neural_network/_multilayer_perceptron.py:690:
ConvergenceWarning: Stochastic Optimizer: Maximum iterations (1000) reached and
the optimization hasn't converged yet.
  warnings.warn(
/projects/proteinml/.links/miniconda3/envs/aidep/lib/python3.10/site-
packages/sklearn/neural_network/_multilayer_perceptron.py:690:
ConvergenceWarning: Stochastic Optimizer: Maximum iterations (1000) reached and
the optimization hasn't converged yet.
  warnings.warn(
/projects/proteinml/.links/miniconda3/envs/aidep/lib/python3.10/site-
packages/sklearn/neural_network/_multilayer_perceptron.py:690:
ConvergenceWarning: Stochastic Optimizer: Maximum iterations (1000) reached and
the optimization hasn't converged yet.
  warnings.warn(
/projects/proteinml/.links/miniconda3/envs/aidep/lib/python3.10/site-
packages/sklearn/neural_network/_multilayer_perceptron.py:690:
```

```
ConvergenceWarning: Stochastic Optimizer: Maximum iterations (1000) reached and
the optimization hasn't converged yet.
  warnings.warn(
/projects/proteinml/.links/miniconda3/envs/aidep/lib/python3.10/site-
packages/sklearn/neural_network/_multilayer_perceptron.py:690:
ConvergenceWarning: Stochastic Optimizer: Maximum iterations (1000) reached and
the optimization hasn't converged yet.
  warnings.warn(
/projects/proteinml/.links/miniconda3/envs/aidep/lib/python3.10/site-
packages/sklearn/neural_network/_multilayer_perceptron.py:690:
ConvergenceWarning: Stochastic Optimizer: Maximum iterations (1000) reached and
the optimization hasn't converged yet.
  warnings.warn(
/projects/proteinml/.links/miniconda3/envs/aidep/lib/python3.10/site-
packages/sklearn/neural_network/_multilayer_perceptron.py:690:
ConvergenceWarning: Stochastic Optimizer: Maximum iterations (1000) reached and
the optimization hasn't converged yet.
  warnings.warn(
/projects/proteinml/.links/miniconda3/envs/aidep/lib/python3.10/site-
packages/sklearn/neural_network/_multilayer_perceptron.py:690:
ConvergenceWarning: Stochastic Optimizer: Maximum iterations (1000) reached and
the optimization hasn't converged yet.
  warnings.warn(
/projects/proteinml/.links/miniconda3/envs/aidep/lib/python3.10/site-
packages/sklearn/neural_network/_multilayer_perceptron.py:690:
ConvergenceWarning: Stochastic Optimizer: Maximum iterations (1000) reached and
the optimization hasn't converged yet.
  warnings.warn(
/projects/proteinml/.links/miniconda3/envs/aidep/lib/python3.10/site-
packages/sklearn/neural_network/_multilayer_perceptron.py:690:
ConvergenceWarning: Stochastic Optimizer: Maximum iterations (1000) reached and
the optimization hasn't converged yet.
  warnings.warn(
/projects/proteinml/.links/miniconda3/envs/aidep/lib/python3.10/site-
packages/sklearn/neural_network/_multilayer_perceptron.py:690:
ConvergenceWarning: Stochastic Optimizer: Maximum iterations (1000) reached and
the optimization hasn't converged yet.
  warnings.warn(
/projects/proteinml/.links/miniconda3/envs/aidep/lib/python3.10/site-
packages/sklearn/neural_network/_multilayer_perceptron.py:690:
ConvergenceWarning: Stochastic Optimizer: Maximum iterations (1000) reached and
the optimization hasn't converged yet.
  warnings.warn(
/projects/proteinml/.links/miniconda3/envs/aidep/lib/python3.10/site-
packages/sklearn/neural_network/_multilayer_perceptron.py:690:
ConvergenceWarning: Stochastic Optimizer: Maximum iterations (1000) reached and
the optimization hasn't converged yet.
  warnings.warn(
```

```
/projects/proteinml/.links/miniconda3/envs/aidep/lib/python3.10/site-
packages/sklearn/neural_network/_multilayer_perceptron.py:690:
ConvergenceWarning: Stochastic Optimizer: Maximum iterations (1000) reached and
the optimization hasn't converged yet.
  warnings.warn(
/projects/proteinml/.links/miniconda3/envs/aidep/lib/python3.10/site-
packages/sklearn/neural_network/_multilayer_perceptron.py:690:
ConvergenceWarning: Stochastic Optimizer: Maximum iterations (1000) reached and
the optimization hasn't converged yet.
  warnings.warn(
/projects/proteinml/.links/miniconda3/envs/aidep/lib/python3.10/site-
packages/sklearn/neural_network/_multilayer_perceptron.py:690:
ConvergenceWarning: Stochastic Optimizer: Maximum iterations (1000) reached and
the optimization hasn't converged yet.
  warnings.warn(
/projects/proteinml/.links/miniconda3/envs/aidep/lib/python3.10/site-
packages/sklearn/neural_network/_multilayer_perceptron.py:690:
ConvergenceWarning: Stochastic Optimizer: Maximum iterations (1000) reached and
the optimization hasn't converged yet.
  warnings.warn(
/projects/proteinml/.links/miniconda3/envs/aidep/lib/python3.10/site-
packages/sklearn/neural_network/_multilayer_perceptron.py:690:
ConvergenceWarning: Stochastic Optimizer: Maximum iterations (1000) reached and
the optimization hasn't converged yet.
  warnings.warn(
/projects/proteinml/.links/miniconda3/envs/aidep/lib/python3.10/site-
packages/sklearn/neural_network/_multilayer_perceptron.py:690:
ConvergenceWarning: Stochastic Optimizer: Maximum iterations (1000) reached and
the optimization hasn't converged yet.
  warnings.warn(
/projects/proteinml/.links/miniconda3/envs/aidep/lib/python3.10/site-
packages/sklearn/neural_network/_multilayer_perceptron.py:690:
ConvergenceWarning: Stochastic Optimizer: Maximum iterations (1000) reached and
the optimization hasn't converged yet.
  warnings.warn(
/projects/proteinml/.links/miniconda3/envs/aidep/lib/python3.10/site-
packages/sklearn/neural_network/_multilayer_perceptron.py:690:
ConvergenceWarning: Stochastic Optimizer: Maximum iterations (1000) reached and
the optimization hasn't converged yet.
  warnings.warn(
/projects/proteinml/.links/miniconda3/envs/aidep/lib/python3.10/site-
packages/sklearn/neural_network/_multilayer_perceptron.py:690:
ConvergenceWarning: Stochastic Optimizer: Maximum iterations (1000) reached and
the optimization hasn't converged yet.
  warnings.warn(
/projects/proteinml/.links/miniconda3/envs/aidep/lib/python3.10/site-
packages/sklearn/neural_network/_multilayer_perceptron.py:690:
ConvergenceWarning: Stochastic Optimizer: Maximum iterations (1000) reached and
```

```
the optimization hasn't converged yet.
  warnings.warn(
/projects/proteinml/.links/miniconda3/envs/aidep/lib/python3.10/site-
packages/sklearn/neural_network/_multilayer_perceptron.py:690:
ConvergenceWarning: Stochastic Optimizer: Maximum iterations (1000) reached and
the optimization hasn't converged yet.
  warnings.warn(
/projects/proteinml/.links/miniconda3/envs/aidep/lib/python3.10/site-
packages/sklearn/neural_network/_multilayer_perceptron.py:690:
ConvergenceWarning: Stochastic Optimizer: Maximum iterations (1000) reached and
the optimization hasn't converged yet.
  warnings.warn(
/projects/proteinml/.links/miniconda3/envs/aidep/lib/python3.10/site-
packages/sklearn/neural_network/_multilayer_perceptron.py:690:
ConvergenceWarning: Stochastic Optimizer: Maximum iterations (1000) reached and
the optimization hasn't converged yet.
  warnings.warn(
/projects/proteinml/.links/miniconda3/envs/aidep/lib/python3.10/site-
packages/sklearn/neural_network/_multilayer_perceptron.py:690:
ConvergenceWarning: Stochastic Optimizer: Maximum iterations (1000) reached and
the optimization hasn't converged yet.
  warnings.warn(
/projects/proteinml/.links/miniconda3/envs/aidep/lib/python3.10/site-
packages/sklearn/neural_network/_multilayer_perceptron.py:690:
ConvergenceWarning: Stochastic Optimizer: Maximum iterations (1000) reached and
the optimization hasn't converged yet.
  warnings.warn(
/projects/proteinml/.links/miniconda3/envs/aidep/lib/python3.10/site-
packages/sklearn/neural_network/_multilayer_perceptron.py:690:
ConvergenceWarning: Stochastic Optimizer: Maximum iterations (1000) reached and
the optimization hasn't converged yet.
  warnings.warn(
/projects/proteinml/.links/miniconda3/envs/aidep/lib/python3.10/site-
packages/sklearn/neural_network/_multilayer_perceptron.py:690:
ConvergenceWarning: Stochastic Optimizer: Maximum iterations (1000) reached and
the optimization hasn't converged yet.
  warnings.warn(
/projects/proteinml/.links/miniconda3/envs/aidep/lib/python3.10/site-
packages/sklearn/neural_network/_multilayer_perceptron.py:690:
ConvergenceWarning: Stochastic Optimizer: Maximum iterations (1000) reached and
the optimization hasn't converged yet.
  warnings.warn(
/projects/proteinml/.links/miniconda3/envs/aidep/lib/python3.10/site-
packages/sklearn/neural_network/_multilayer_perceptron.py:690:
ConvergenceWarning: Stochastic Optimizer: Maximum iterations (1000) reached and
the optimization hasn't converged yet.
  warnings.warn(
/projects/proteinml/.links/miniconda3/envs/aidep/lib/python3.10/site-
```

```
packages/sklearn/neural_network/_multilayer_perceptron.py:690:
ConvergenceWarning: Stochastic Optimizer: Maximum iterations (1000) reached and
the optimization hasn't converged yet.
  warnings.warn(
/projects/proteinml/.links/miniconda3/envs/aidep/lib/python3.10/site-
packages/sklearn/neural_network/_multilayer_perceptron.py:690:
ConvergenceWarning: Stochastic Optimizer: Maximum iterations (1000) reached and
the optimization hasn't converged yet.
  warnings.warn(
/projects/proteinml/.links/miniconda3/envs/aidep/lib/python3.10/site-
packages/sklearn/neural_network/_multilayer_perceptron.py:690:
ConvergenceWarning: Stochastic Optimizer: Maximum iterations (1000) reached and
the optimization hasn't converged yet.
  warnings.warn(
/projects/proteinml/.links/miniconda3/envs/aidep/lib/python3.10/site-
packages/sklearn/neural_network/_multilayer_perceptron.py:690:
ConvergenceWarning: Stochastic Optimizer: Maximum iterations (1000) reached and
the optimization hasn't converged yet.
  warnings.warn(
/projects/proteinml/.links/miniconda3/envs/aidep/lib/python3.10/site-
packages/sklearn/neural_network/_multilayer_perceptron.py:690:
ConvergenceWarning: Stochastic Optimizer: Maximum iterations (1000) reached and
the optimization hasn't converged yet.
  warnings.warn(
/projects/proteinml/.links/miniconda3/envs/aidep/lib/python3.10/site-
packages/sklearn/neural_network/_multilayer_perceptron.py:690:
ConvergenceWarning: Stochastic Optimizer: Maximum iterations (1000) reached and
the optimization hasn't converged yet.
  warnings.warn(
/projects/proteinml/.links/miniconda3/envs/aidep/lib/python3.10/site-
packages/sklearn/neural_network/_multilayer_perceptron.py:690:
ConvergenceWarning: Stochastic Optimizer: Maximum iterations (1000) reached and
the optimization hasn't converged yet.
  warnings.warn(
/projects/proteinml/.links/miniconda3/envs/aidep/lib/python3.10/site-
packages/sklearn/neural_network/_multilayer_perceptron.py:690:
ConvergenceWarning: Stochastic Optimizer: Maximum iterations (1000) reached and
the optimization hasn't converged yet.
  warnings.warn(
/projects/proteinml/.links/miniconda3/envs/aidep/lib/python3.10/site-
packages/sklearn/neural_network/_multilayer_perceptron.py:690:
ConvergenceWarning: Stochastic Optimizer: Maximum iterations (1000) reached and
the optimization hasn't converged yet.
  warnings.warn(
/projects/proteinml/.links/miniconda3/envs/aidep/lib/python3.10/site-
packages/sklearn/neural_network/_multilayer_perceptron.py:690:
ConvergenceWarning: Stochastic Optimizer: Maximum iterations (1000) reached and
the optimization hasn't converged yet.
```

```
  warnings.warn(
/projects/proteinml/.links/miniconda3/envs/aidep/lib/python3.10/site-
packages/sklearn/neural_network/_multilayer_perceptron.py:690:
ConvergenceWarning: Stochastic Optimizer: Maximum iterations (1000) reached and
the optimization hasn't converged yet.
  warnings.warn(
/projects/proteinml/.links/miniconda3/envs/aidep/lib/python3.10/site-
packages/sklearn/neural_network/_multilayer_perceptron.py:690:
ConvergenceWarning: Stochastic Optimizer: Maximum iterations (1000) reached and
the optimization hasn't converged yet.
  warnings.warn(
/projects/proteinml/.links/miniconda3/envs/aidep/lib/python3.10/site-
packages/sklearn/neural_network/_multilayer_perceptron.py:690:
ConvergenceWarning: Stochastic Optimizer: Maximum iterations (1000) reached and
the optimization hasn't converged yet.
  warnings.warn(
/projects/proteinml/.links/miniconda3/envs/aidep/lib/python3.10/site-
packages/sklearn/neural_network/_multilayer_perceptron.py:690:
ConvergenceWarning: Stochastic Optimizer: Maximum iterations (1000) reached and
the optimization hasn't converged yet.
  warnings.warn(
/projects/proteinml/.links/miniconda3/envs/aidep/lib/python3.10/site-
packages/sklearn/neural_network/_multilayer_perceptron.py:690:
ConvergenceWarning: Stochastic Optimizer: Maximum iterations (1000) reached and
the optimization hasn't converged yet.
  warnings.warn(
/projects/proteinml/.links/miniconda3/envs/aidep/lib/python3.10/site-
packages/sklearn/neural_network/_multilayer_perceptron.py:690:
ConvergenceWarning: Stochastic Optimizer: Maximum iterations (1000) reached and
the optimization hasn't converged yet.
  warnings.warn(
/projects/proteinml/.links/miniconda3/envs/aidep/lib/python3.10/site-
packages/sklearn/neural_network/_multilayer_perceptron.py:690:
ConvergenceWarning: Stochastic Optimizer: Maximum iterations (1000) reached and
the optimization hasn't converged yet.
  warnings.warn(
/projects/proteinml/.links/miniconda3/envs/aidep/lib/python3.10/site-
packages/sklearn/neural_network/_multilayer_perceptron.py:690:
ConvergenceWarning: Stochastic Optimizer: Maximum iterations (1000) reached and
the optimization hasn't converged yet.
  warnings.warn(
/projects/proteinml/.links/miniconda3/envs/aidep/lib/python3.10/site-
packages/sklearn/neural_network/_multilayer_perceptron.py:690:
ConvergenceWarning: Stochastic Optimizer: Maximum iterations (1000) reached and
the optimization hasn't converged yet.
  warnings.warn(
/projects/proteinml/.links/miniconda3/envs/aidep/lib/python3.10/site-
packages/sklearn/neural_network/_multilayer_perceptron.py:690:
```

```
ConvergenceWarning: Stochastic Optimizer: Maximum iterations (1000) reached and
the optimization hasn't converged yet.
  warnings.warn(
/projects/proteinml/.links/miniconda3/envs/aidep/lib/python3.10/site-
packages/sklearn/neural_network/_multilayer_perceptron.py:690:
ConvergenceWarning: Stochastic Optimizer: Maximum iterations (1000) reached and
the optimization hasn't converged yet.
  warnings.warn(
/projects/proteinml/.links/miniconda3/envs/aidep/lib/python3.10/site-
packages/sklearn/neural_network/_multilayer_perceptron.py:690:
ConvergenceWarning: Stochastic Optimizer: Maximum iterations (1000) reached and
the optimization hasn't converged yet.
  warnings.warn(
/projects/proteinml/.links/miniconda3/envs/aidep/lib/python3.10/site-
packages/sklearn/neural_network/_multilayer_perceptron.py:690:
ConvergenceWarning: Stochastic Optimizer: Maximum iterations (1000) reached and
the optimization hasn't converged yet.
  warnings.warn(
/projects/proteinml/.links/miniconda3/envs/aidep/lib/python3.10/site-
packages/sklearn/neural_network/_multilayer_perceptron.py:690:
ConvergenceWarning: Stochastic Optimizer: Maximum iterations (1000) reached and
the optimization hasn't converged yet.
  warnings.warn(
/projects/proteinml/.links/miniconda3/envs/aidep/lib/python3.10/site-
packages/sklearn/neural_network/_multilayer_perceptron.py:690:
ConvergenceWarning: Stochastic Optimizer: Maximum iterations (1000) reached and
the optimization hasn't converged yet.
  warnings.warn(
/projects/proteinml/.links/miniconda3/envs/aidep/lib/python3.10/site-
packages/sklearn/neural_network/_multilayer_perceptron.py:690:
ConvergenceWarning: Stochastic Optimizer: Maximum iterations (1000) reached and
the optimization hasn't converged yet.
  warnings.warn(
/projects/proteinml/.links/miniconda3/envs/aidep/lib/python3.10/site-
packages/sklearn/neural_network/_multilayer_perceptron.py:690:
ConvergenceWarning: Stochastic Optimizer: Maximum iterations (1000) reached and
the optimization hasn't converged yet.
  warnings.warn(
/projects/proteinml/.links/miniconda3/envs/aidep/lib/python3.10/site-
packages/sklearn/neural_network/_multilayer_perceptron.py:690:
ConvergenceWarning: Stochastic Optimizer: Maximum iterations (1000) reached and
the optimization hasn't converged yet.
  warnings.warn(
/projects/proteinml/.links/miniconda3/envs/aidep/lib/python3.10/site-
packages/sklearn/neural_network/_multilayer_perceptron.py:690:
ConvergenceWarning: Stochastic Optimizer: Maximum iterations (1000) reached and
the optimization hasn't converged yet.
  warnings.warn(
```

```
/projects/proteinml/.links/miniconda3/envs/aidep/lib/python3.10/site-
packages/sklearn/neural_network/_multilayer_perceptron.py:690:
ConvergenceWarning: Stochastic Optimizer: Maximum iterations (1000) reached and
the optimization hasn't converged yet.
  warnings.warn(
/projects/proteinml/.links/miniconda3/envs/aidep/lib/python3.10/site-
packages/sklearn/neural_network/_multilayer_perceptron.py:690:
ConvergenceWarning: Stochastic Optimizer: Maximum iterations (1000) reached and
the optimization hasn't converged yet.
  warnings.warn(
/projects/proteinml/.links/miniconda3/envs/aidep/lib/python3.10/site-
packages/sklearn/neural_network/_multilayer_perceptron.py:690:
ConvergenceWarning: Stochastic Optimizer: Maximum iterations (1000) reached and
the optimization hasn't converged yet.
  warnings.warn(
/projects/proteinml/.links/miniconda3/envs/aidep/lib/python3.10/site-
packages/sklearn/neural_network/_multilayer_perceptron.py:690:
ConvergenceWarning: Stochastic Optimizer: Maximum iterations (1000) reached and
the optimization hasn't converged yet.
  warnings.warn(
/projects/proteinml/.links/miniconda3/envs/aidep/lib/python3.10/site-
packages/sklearn/neural_network/_multilayer_perceptron.py:690:
ConvergenceWarning: Stochastic Optimizer: Maximum iterations (1000) reached and
the optimization hasn't converged yet.
  warnings.warn(
/projects/proteinml/.links/miniconda3/envs/aidep/lib/python3.10/site-
packages/sklearn/neural_network/_multilayer_perceptron.py:690:
ConvergenceWarning: Stochastic Optimizer: Maximum iterations (1000) reached and
the optimization hasn't converged yet.
  warnings.warn(
/projects/proteinml/.links/miniconda3/envs/aidep/lib/python3.10/site-
packages/sklearn/neural_network/_multilayer_perceptron.py:690:
ConvergenceWarning: Stochastic Optimizer: Maximum iterations (1000) reached and
the optimization hasn't converged yet.
  warnings.warn(
/projects/proteinml/.links/miniconda3/envs/aidep/lib/python3.10/site-
packages/sklearn/neural_network/_multilayer_perceptron.py:690:
ConvergenceWarning: Stochastic Optimizer: Maximum iterations (1000) reached and
the optimization hasn't converged yet.
  warnings.warn(
/projects/proteinml/.links/miniconda3/envs/aidep/lib/python3.10/site-
packages/sklearn/neural_network/_multilayer_perceptron.py:690:
ConvergenceWarning: Stochastic Optimizer: Maximum iterations (1000) reached and
the optimization hasn't converged yet.
  warnings.warn(
/projects/proteinml/.links/miniconda3/envs/aidep/lib/python3.10/site-
packages/sklearn/neural_network/_multilayer_perceptron.py:690:
ConvergenceWarning: Stochastic Optimizer: Maximum iterations (1000) reached and
```

```
the optimization hasn't converged yet.
  warnings.warn(
/projects/proteinml/.links/miniconda3/envs/aidep/lib/python3.10/site-
packages/sklearn/neural_network/_multilayer_perceptron.py:690:
ConvergenceWarning: Stochastic Optimizer: Maximum iterations (1000) reached and
the optimization hasn't converged yet.
  warnings.warn(
/projects/proteinml/.links/miniconda3/envs/aidep/lib/python3.10/site-
packages/sklearn/neural_network/_multilayer_perceptron.py:690:
ConvergenceWarning: Stochastic Optimizer: Maximum iterations (1000) reached and
the optimization hasn't converged yet.
  warnings.warn(
/projects/proteinml/.links/miniconda3/envs/aidep/lib/python3.10/site-
packages/sklearn/neural_network/_multilayer_perceptron.py:690:
ConvergenceWarning: Stochastic Optimizer: Maximum iterations (1000) reached and
the optimization hasn't converged yet.
  warnings.warn(
/projects/proteinml/.links/miniconda3/envs/aidep/lib/python3.10/site-
packages/sklearn/neural_network/_multilayer_perceptron.py:690:
ConvergenceWarning: Stochastic Optimizer: Maximum iterations (1000) reached and
the optimization hasn't converged yet.
  warnings.warn(
/projects/proteinml/.links/miniconda3/envs/aidep/lib/python3.10/site-
packages/sklearn/neural_network/_multilayer_perceptron.py:690:
ConvergenceWarning: Stochastic Optimizer: Maximum iterations (1000) reached and
the optimization hasn't converged yet.
  warnings.warn(
/projects/proteinml/.links/miniconda3/envs/aidep/lib/python3.10/site-
packages/sklearn/neural_network/_multilayer_perceptron.py:690:
ConvergenceWarning: Stochastic Optimizer: Maximum iterations (1000) reached and
the optimization hasn't converged yet.
  warnings.warn(
/projects/proteinml/.links/miniconda3/envs/aidep/lib/python3.10/site-
packages/sklearn/neural_network/_multilayer_perceptron.py:690:
ConvergenceWarning: Stochastic Optimizer: Maximum iterations (1000) reached and
the optimization hasn't converged yet.
  warnings.warn(
/projects/proteinml/.links/miniconda3/envs/aidep/lib/python3.10/site-
packages/sklearn/neural_network/_multilayer_perceptron.py:690:
ConvergenceWarning: Stochastic Optimizer: Maximum iterations (1000) reached and
the optimization hasn't converged yet.
  warnings.warn(
/projects/proteinml/.links/miniconda3/envs/aidep/lib/python3.10/site-
packages/sklearn/neural_network/_multilayer_perceptron.py:690:
ConvergenceWarning: Stochastic Optimizer: Maximum iterations (1000) reached and
the optimization hasn't converged yet.
  warnings.warn(
/projects/proteinml/.links/miniconda3/envs/aidep/lib/python3.10/site-
```

```
packages/sklearn/neural_network/_multilayer_perceptron.py:690:
ConvergenceWarning: Stochastic Optimizer: Maximum iterations (1000) reached and
the optimization hasn't converged yet.
  warnings.warn(
/projects/proteinml/.links/miniconda3/envs/aidep/lib/python3.10/site-
packages/sklearn/neural_network/_multilayer_perceptron.py:690:
ConvergenceWarning: Stochastic Optimizer: Maximum iterations (1000) reached and
the optimization hasn't converged yet.
  warnings.warn(
/projects/proteinml/.links/miniconda3/envs/aidep/lib/python3.10/site-
packages/sklearn/neural_network/_multilayer_perceptron.py:690:
ConvergenceWarning: Stochastic Optimizer: Maximum iterations (1000) reached and
the optimization hasn't converged yet.
  warnings.warn(
/projects/proteinml/.links/miniconda3/envs/aidep/lib/python3.10/site-
packages/sklearn/neural_network/_multilayer_perceptron.py:690:
ConvergenceWarning: Stochastic Optimizer: Maximum iterations (1000) reached and
the optimization hasn't converged yet.
  warnings.warn(
/projects/proteinml/.links/miniconda3/envs/aidep/lib/python3.10/site-
packages/sklearn/neural_network/_multilayer_perceptron.py:690:
ConvergenceWarning: Stochastic Optimizer: Maximum iterations (1000) reached and
the optimization hasn't converged yet.
  warnings.warn(
/projects/proteinml/.links/miniconda3/envs/aidep/lib/python3.10/site-
packages/sklearn/neural_network/_multilayer_perceptron.py:690:
ConvergenceWarning: Stochastic Optimizer: Maximum iterations (1000) reached and
the optimization hasn't converged yet.
  warnings.warn(
/projects/proteinml/.links/miniconda3/envs/aidep/lib/python3.10/site-
packages/sklearn/neural_network/_multilayer_perceptron.py:690:
ConvergenceWarning: Stochastic Optimizer: Maximum iterations (1000) reached and
the optimization hasn't converged yet.
  warnings.warn(
/projects/proteinml/.links/miniconda3/envs/aidep/lib/python3.10/site-
packages/sklearn/neural_network/_multilayer_perceptron.py:690:
ConvergenceWarning: Stochastic Optimizer: Maximum iterations (1000) reached and
the optimization hasn't converged yet.
  warnings.warn(
/projects/proteinml/.links/miniconda3/envs/aidep/lib/python3.10/site-
packages/sklearn/neural_network/_multilayer_perceptron.py:690:
ConvergenceWarning: Stochastic Optimizer: Maximum iterations (1000) reached and
the optimization hasn't converged yet.
  warnings.warn(
/projects/proteinml/.links/miniconda3/envs/aidep/lib/python3.10/site-
packages/sklearn/neural_network/_multilayer_perceptron.py:690:
ConvergenceWarning: Stochastic Optimizer: Maximum iterations (1000) reached and
the optimization hasn't converged yet.
```

```
  warnings.warn(
/projects/proteinml/.links/miniconda3/envs/aidep/lib/python3.10/site-
packages/sklearn/neural_network/_multilayer_perceptron.py:690:
ConvergenceWarning: Stochastic Optimizer: Maximum iterations (1000) reached and
the optimization hasn't converged yet.
  warnings.warn(
/projects/proteinml/.links/miniconda3/envs/aidep/lib/python3.10/site-
packages/sklearn/neural_network/_multilayer_perceptron.py:690:
ConvergenceWarning: Stochastic Optimizer: Maximum iterations (1000) reached and
the optimization hasn't converged yet.
  warnings.warn(
/projects/proteinml/.links/miniconda3/envs/aidep/lib/python3.10/site-
packages/sklearn/neural_network/_multilayer_perceptron.py:690:
ConvergenceWarning: Stochastic Optimizer: Maximum iterations (1000) reached and
the optimization hasn't converged yet.
  warnings.warn(
/projects/proteinml/.links/miniconda3/envs/aidep/lib/python3.10/site-
packages/sklearn/neural_network/_multilayer_perceptron.py:690:
ConvergenceWarning: Stochastic Optimizer: Maximum iterations (1000) reached and
the optimization hasn't converged yet.
  warnings.warn(
/projects/proteinml/.links/miniconda3/envs/aidep/lib/python3.10/site-
packages/sklearn/neural_network/_multilayer_perceptron.py:690:
ConvergenceWarning: Stochastic Optimizer: Maximum iterations (1000) reached and
the optimization hasn't converged yet.
  warnings.warn(
/projects/proteinml/.links/miniconda3/envs/aidep/lib/python3.10/site-
packages/sklearn/neural_network/_multilayer_perceptron.py:690:
ConvergenceWarning: Stochastic Optimizer: Maximum iterations (1000) reached and
the optimization hasn't converged yet.
  warnings.warn(
/projects/proteinml/.links/miniconda3/envs/aidep/lib/python3.10/site-
packages/sklearn/neural_network/_multilayer_perceptron.py:690:
ConvergenceWarning: Stochastic Optimizer: Maximum iterations (1000) reached and
the optimization hasn't converged yet.
  warnings.warn(
/projects/proteinml/.links/miniconda3/envs/aidep/lib/python3.10/site-
packages/sklearn/neural_network/_multilayer_perceptron.py:690:
ConvergenceWarning: Stochastic Optimizer: Maximum iterations (1000) reached and
the optimization hasn't converged yet.
  warnings.warn(
/projects/proteinml/.links/miniconda3/envs/aidep/lib/python3.10/site-
packages/sklearn/neural_network/_multilayer_perceptron.py:690:
ConvergenceWarning: Stochastic Optimizer: Maximum iterations (1000) reached and
the optimization hasn't converged yet.
  warnings.warn(
/projects/proteinml/.links/miniconda3/envs/aidep/lib/python3.10/site-
packages/sklearn/neural_network/_multilayer_perceptron.py:690:
```

```
ConvergenceWarning: Stochastic Optimizer: Maximum iterations (1000) reached and
the optimization hasn't converged yet.
  warnings.warn(
/projects/proteinml/.links/miniconda3/envs/aidep/lib/python3.10/site-
packages/sklearn/neural_network/_multilayer_perceptron.py:690:
ConvergenceWarning: Stochastic Optimizer: Maximum iterations (1000) reached and
the optimization hasn't converged yet.
  warnings.warn(
/projects/proteinml/.links/miniconda3/envs/aidep/lib/python3.10/site-
packages/sklearn/neural_network/_multilayer_perceptron.py:690:
ConvergenceWarning: Stochastic Optimizer: Maximum iterations (1000) reached and
the optimization hasn't converged yet.
  warnings.warn(
/projects/proteinml/.links/miniconda3/envs/aidep/lib/python3.10/site-
packages/sklearn/neural_network/_multilayer_perceptron.py:690:
ConvergenceWarning: Stochastic Optimizer: Maximum iterations (1000) reached and
the optimization hasn't converged yet.
  warnings.warn(
/projects/proteinml/.links/miniconda3/envs/aidep/lib/python3.10/site-
packages/sklearn/neural_network/_multilayer_perceptron.py:690:
ConvergenceWarning: Stochastic Optimizer: Maximum iterations (1000) reached and
the optimization hasn't converged yet.
  warnings.warn(
/projects/proteinml/.links/miniconda3/envs/aidep/lib/python3.10/site-
packages/sklearn/neural_network/_multilayer_perceptron.py:690:
ConvergenceWarning: Stochastic Optimizer: Maximum iterations (1000) reached and
the optimization hasn't converged yet.
  warnings.warn(
/projects/proteinml/.links/miniconda3/envs/aidep/lib/python3.10/site-
packages/sklearn/neural_network/_multilayer_perceptron.py:690:
ConvergenceWarning: Stochastic Optimizer: Maximum iterations (1000) reached and
the optimization hasn't converged yet.
  warnings.warn(
/projects/proteinml/.links/miniconda3/envs/aidep/lib/python3.10/site-
packages/sklearn/neural_network/_multilayer_perceptron.py:690:
ConvergenceWarning: Stochastic Optimizer: Maximum iterations (1000) reached and
the optimization hasn't converged yet.
  warnings.warn(
/projects/proteinml/.links/miniconda3/envs/aidep/lib/python3.10/site-
packages/sklearn/neural_network/_multilayer_perceptron.py:690:
ConvergenceWarning: Stochastic Optimizer: Maximum iterations (1000) reached and
the optimization hasn't converged yet.
  warnings.warn(
/projects/proteinml/.links/miniconda3/envs/aidep/lib/python3.10/site-
packages/sklearn/neural_network/_multilayer_perceptron.py:690:
ConvergenceWarning: Stochastic Optimizer: Maximum iterations (1000) reached and
the optimization hasn't converged yet.
  warnings.warn(
```

```
/projects/proteinml/.links/miniconda3/envs/aidep/lib/python3.10/site-
packages/sklearn/neural_network/_multilayer_perceptron.py:690:
ConvergenceWarning: Stochastic Optimizer: Maximum iterations (1000) reached and
the optimization hasn't converged yet.
  warnings.warn(
/projects/proteinml/.links/miniconda3/envs/aidep/lib/python3.10/site-
packages/sklearn/neural_network/_multilayer_perceptron.py:690:
ConvergenceWarning: Stochastic Optimizer: Maximum iterations (1000) reached and
the optimization hasn't converged yet.
  warnings.warn(
/projects/proteinml/.links/miniconda3/envs/aidep/lib/python3.10/site-
packages/sklearn/neural_network/_multilayer_perceptron.py:690:
ConvergenceWarning: Stochastic Optimizer: Maximum iterations (1000) reached and
the optimization hasn't converged yet.
  warnings.warn(
/projects/proteinml/.links/miniconda3/envs/aidep/lib/python3.10/site-
packages/sklearn/neural_network/_multilayer_perceptron.py:690:
ConvergenceWarning: Stochastic Optimizer: Maximum iterations (1000) reached and
the optimization hasn't converged yet.
  warnings.warn(
/projects/proteinml/.links/miniconda3/envs/aidep/lib/python3.10/site-
packages/sklearn/neural_network/_multilayer_perceptron.py:690:
ConvergenceWarning: Stochastic Optimizer: Maximum iterations (1000) reached and
the optimization hasn't converged yet.
  warnings.warn(
```

[27]: ['evc_ohe_mlp_scores.joblib']

1.6.3 4.3 EVC plus ESM2 linear

[28]:
```
X_ = np.concatenate([X_esm, X_evc], axis=1)
```

[29]:
```
evc_esmfull_linear_scores = hyperopt_and_scoring(linear_pca, X_, y,␣
↪linear_param_space)
joblib.dump(evc_esmfull_linear_scores, 'evc_esmfull_linear_scores.joblib')
```

[29]: ['evc_esmfull_linear_scores.joblib']

1.6.4 4.4 EVC plus ESM2 MLP

[30]:
```
evc_esmfull_mlp_scores = hyperopt_and_scoring(mlp_pca, X_, y, mlp_param_space)
joblib.dump(evc_esmfull_mlp_scores, 'evc_esmfull_mlp_scores.joblib')
```

```
/projects/proteinml/.links/miniconda3/envs/aidep/lib/python3.10/site-
packages/sklearn/neural_network/_multilayer_perceptron.py:690:
ConvergenceWarning: Stochastic Optimizer: Maximum iterations (1000) reached and
the optimization hasn't converged yet.
```

```
  warnings.warn(
/projects/proteinml/.links/miniconda3/envs/aidep/lib/python3.10/site-
packages/sklearn/neural_network/_multilayer_perceptron.py:690:
ConvergenceWarning: Stochastic Optimizer: Maximum iterations (1000) reached and
the optimization hasn't converged yet.
  warnings.warn(
/projects/proteinml/.links/miniconda3/envs/aidep/lib/python3.10/site-
packages/sklearn/neural_network/_multilayer_perceptron.py:690:
ConvergenceWarning: Stochastic Optimizer: Maximum iterations (1000) reached and
the optimization hasn't converged yet.
  warnings.warn(
/projects/proteinml/.links/miniconda3/envs/aidep/lib/python3.10/site-
packages/sklearn/neural_network/_multilayer_perceptron.py:690:
ConvergenceWarning: Stochastic Optimizer: Maximum iterations (1000) reached and
the optimization hasn't converged yet.
  warnings.warn(
/projects/proteinml/.links/miniconda3/envs/aidep/lib/python3.10/site-
packages/sklearn/neural_network/_multilayer_perceptron.py:690:
ConvergenceWarning: Stochastic Optimizer: Maximum iterations (1000) reached and
the optimization hasn't converged yet.
  warnings.warn(
/projects/proteinml/.links/miniconda3/envs/aidep/lib/python3.10/site-
packages/sklearn/neural_network/_multilayer_perceptron.py:690:
ConvergenceWarning: Stochastic Optimizer: Maximum iterations (1000) reached and
the optimization hasn't converged yet.
  warnings.warn(
/projects/proteinml/.links/miniconda3/envs/aidep/lib/python3.10/site-
packages/sklearn/neural_network/_multilayer_perceptron.py:690:
ConvergenceWarning: Stochastic Optimizer: Maximum iterations (1000) reached and
the optimization hasn't converged yet.
  warnings.warn(
/projects/proteinml/.links/miniconda3/envs/aidep/lib/python3.10/site-
packages/sklearn/neural_network/_multilayer_perceptron.py:690:
ConvergenceWarning: Stochastic Optimizer: Maximum iterations (1000) reached and
the optimization hasn't converged yet.
  warnings.warn(
/projects/proteinml/.links/miniconda3/envs/aidep/lib/python3.10/site-
packages/sklearn/neural_network/_multilayer_perceptron.py:690:
ConvergenceWarning: Stochastic Optimizer: Maximum iterations (1000) reached and
the optimization hasn't converged yet.
  warnings.warn(
/projects/proteinml/.links/miniconda3/envs/aidep/lib/python3.10/site-
packages/sklearn/neural_network/_multilayer_perceptron.py:690:
ConvergenceWarning: Stochastic Optimizer: Maximum iterations (1000) reached and
the optimization hasn't converged yet.
  warnings.warn(
/projects/proteinml/.links/miniconda3/envs/aidep/lib/python3.10/site-
packages/sklearn/neural_network/_multilayer_perceptron.py:690:
```

```
ConvergenceWarning: Stochastic Optimizer: Maximum iterations (1000) reached and
the optimization hasn't converged yet.
  warnings.warn(
/projects/proteinml/.links/miniconda3/envs/aidep/lib/python3.10/site-
packages/sklearn/neural_network/_multilayer_perceptron.py:690:
ConvergenceWarning: Stochastic Optimizer: Maximum iterations (1000) reached and
the optimization hasn't converged yet.
  warnings.warn(
/projects/proteinml/.links/miniconda3/envs/aidep/lib/python3.10/site-
packages/sklearn/neural_network/_multilayer_perceptron.py:690:
ConvergenceWarning: Stochastic Optimizer: Maximum iterations (1000) reached and
the optimization hasn't converged yet.
  warnings.warn(
/projects/proteinml/.links/miniconda3/envs/aidep/lib/python3.10/site-
packages/sklearn/neural_network/_multilayer_perceptron.py:690:
ConvergenceWarning: Stochastic Optimizer: Maximum iterations (1000) reached and
the optimization hasn't converged yet.
  warnings.warn(
/projects/proteinml/.links/miniconda3/envs/aidep/lib/python3.10/site-
packages/sklearn/neural_network/_multilayer_perceptron.py:690:
ConvergenceWarning: Stochastic Optimizer: Maximum iterations (1000) reached and
the optimization hasn't converged yet.
  warnings.warn(
/projects/proteinml/.links/miniconda3/envs/aidep/lib/python3.10/site-
packages/sklearn/neural_network/_multilayer_perceptron.py:690:
ConvergenceWarning: Stochastic Optimizer: Maximum iterations (1000) reached and
the optimization hasn't converged yet.
  warnings.warn(
/projects/proteinml/.links/miniconda3/envs/aidep/lib/python3.10/site-
packages/sklearn/neural_network/_multilayer_perceptron.py:690:
ConvergenceWarning: Stochastic Optimizer: Maximum iterations (1000) reached and
the optimization hasn't converged yet.
  warnings.warn(
/projects/proteinml/.links/miniconda3/envs/aidep/lib/python3.10/site-
packages/sklearn/neural_network/_multilayer_perceptron.py:690:
ConvergenceWarning: Stochastic Optimizer: Maximum iterations (1000) reached and
the optimization hasn't converged yet.
  warnings.warn(
/projects/proteinml/.links/miniconda3/envs/aidep/lib/python3.10/site-
packages/sklearn/neural_network/_multilayer_perceptron.py:690:
ConvergenceWarning: Stochastic Optimizer: Maximum iterations (1000) reached and
the optimization hasn't converged yet.
  warnings.warn(
/projects/proteinml/.links/miniconda3/envs/aidep/lib/python3.10/site-
packages/sklearn/neural_network/_multilayer_perceptron.py:690:
ConvergenceWarning: Stochastic Optimizer: Maximum iterations (1000) reached and
the optimization hasn't converged yet.
  warnings.warn(
```

```
/projects/proteinml/.links/miniconda3/envs/aidep/lib/python3.10/site-
packages/sklearn/neural_network/_multilayer_perceptron.py:690:
ConvergenceWarning: Stochastic Optimizer: Maximum iterations (1000) reached and
the optimization hasn't converged yet.
  warnings.warn(
/projects/proteinml/.links/miniconda3/envs/aidep/lib/python3.10/site-
packages/sklearn/neural_network/_multilayer_perceptron.py:690:
ConvergenceWarning: Stochastic Optimizer: Maximum iterations (1000) reached and
the optimization hasn't converged yet.
  warnings.warn(
/projects/proteinml/.links/miniconda3/envs/aidep/lib/python3.10/site-
packages/sklearn/neural_network/_multilayer_perceptron.py:690:
ConvergenceWarning: Stochastic Optimizer: Maximum iterations (1000) reached and
the optimization hasn't converged yet.
  warnings.warn(
/projects/proteinml/.links/miniconda3/envs/aidep/lib/python3.10/site-
packages/sklearn/neural_network/_multilayer_perceptron.py:690:
ConvergenceWarning: Stochastic Optimizer: Maximum iterations (1000) reached and
the optimization hasn't converged yet.
  warnings.warn(
/projects/proteinml/.links/miniconda3/envs/aidep/lib/python3.10/site-
packages/sklearn/neural_network/_multilayer_perceptron.py:690:
ConvergenceWarning: Stochastic Optimizer: Maximum iterations (1000) reached and
the optimization hasn't converged yet.
  warnings.warn(
/projects/proteinml/.links/miniconda3/envs/aidep/lib/python3.10/site-
packages/sklearn/neural_network/_multilayer_perceptron.py:690:
ConvergenceWarning: Stochastic Optimizer: Maximum iterations (1000) reached and
the optimization hasn't converged yet.
  warnings.warn(
/projects/proteinml/.links/miniconda3/envs/aidep/lib/python3.10/site-
packages/sklearn/neural_network/_multilayer_perceptron.py:690:
ConvergenceWarning: Stochastic Optimizer: Maximum iterations (1000) reached and
the optimization hasn't converged yet.
  warnings.warn(
/projects/proteinml/.links/miniconda3/envs/aidep/lib/python3.10/site-
packages/sklearn/neural_network/_multilayer_perceptron.py:690:
ConvergenceWarning: Stochastic Optimizer: Maximum iterations (1000) reached and
the optimization hasn't converged yet.
  warnings.warn(
/projects/proteinml/.links/miniconda3/envs/aidep/lib/python3.10/site-
packages/sklearn/neural_network/_multilayer_perceptron.py:690:
ConvergenceWarning: Stochastic Optimizer: Maximum iterations (1000) reached and
the optimization hasn't converged yet.
  warnings.warn(
/projects/proteinml/.links/miniconda3/envs/aidep/lib/python3.10/site-
packages/sklearn/neural_network/_multilayer_perceptron.py:690:
ConvergenceWarning: Stochastic Optimizer: Maximum iterations (1000) reached and
```

```
the optimization hasn't converged yet.
  warnings.warn(
/projects/proteinml/.links/miniconda3/envs/aidep/lib/python3.10/site-
packages/sklearn/neural_network/_multilayer_perceptron.py:690:
ConvergenceWarning: Stochastic Optimizer: Maximum iterations (1000) reached and
the optimization hasn't converged yet.
  warnings.warn(
/projects/proteinml/.links/miniconda3/envs/aidep/lib/python3.10/site-
packages/sklearn/neural_network/_multilayer_perceptron.py:690:
ConvergenceWarning: Stochastic Optimizer: Maximum iterations (1000) reached and
the optimization hasn't converged yet.
  warnings.warn(
/projects/proteinml/.links/miniconda3/envs/aidep/lib/python3.10/site-
packages/sklearn/neural_network/_multilayer_perceptron.py:690:
ConvergenceWarning: Stochastic Optimizer: Maximum iterations (1000) reached and
the optimization hasn't converged yet.
  warnings.warn(
/projects/proteinml/.links/miniconda3/envs/aidep/lib/python3.10/site-
packages/sklearn/neural_network/_multilayer_perceptron.py:690:
ConvergenceWarning: Stochastic Optimizer: Maximum iterations (1000) reached and
the optimization hasn't converged yet.
  warnings.warn(
/projects/proteinml/.links/miniconda3/envs/aidep/lib/python3.10/site-
packages/sklearn/neural_network/_multilayer_perceptron.py:690:
ConvergenceWarning: Stochastic Optimizer: Maximum iterations (1000) reached and
the optimization hasn't converged yet.
  warnings.warn(
/projects/proteinml/.links/miniconda3/envs/aidep/lib/python3.10/site-
packages/sklearn/neural_network/_multilayer_perceptron.py:690:
ConvergenceWarning: Stochastic Optimizer: Maximum iterations (1000) reached and
the optimization hasn't converged yet.
  warnings.warn(
/projects/proteinml/.links/miniconda3/envs/aidep/lib/python3.10/site-
packages/sklearn/neural_network/_multilayer_perceptron.py:690:
ConvergenceWarning: Stochastic Optimizer: Maximum iterations (1000) reached and
the optimization hasn't converged yet.
  warnings.warn(
/projects/proteinml/.links/miniconda3/envs/aidep/lib/python3.10/site-
packages/sklearn/neural_network/_multilayer_perceptron.py:690:
ConvergenceWarning: Stochastic Optimizer: Maximum iterations (1000) reached and
the optimization hasn't converged yet.
  warnings.warn(
/projects/proteinml/.links/miniconda3/envs/aidep/lib/python3.10/site-
packages/sklearn/neural_network/_multilayer_perceptron.py:690:
ConvergenceWarning: Stochastic Optimizer: Maximum iterations (1000) reached and
the optimization hasn't converged yet.
  warnings.warn(
/projects/proteinml/.links/miniconda3/envs/aidep/lib/python3.10/site-
```

```
packages/sklearn/neural_network/_multilayer_perceptron.py:690:
ConvergenceWarning: Stochastic Optimizer: Maximum iterations (1000) reached and
the optimization hasn't converged yet.
  warnings.warn(
/projects/proteinml/.links/miniconda3/envs/aidep/lib/python3.10/site-
packages/sklearn/neural_network/_multilayer_perceptron.py:690:
ConvergenceWarning: Stochastic Optimizer: Maximum iterations (1000) reached and
the optimization hasn't converged yet.
  warnings.warn(
/projects/proteinml/.links/miniconda3/envs/aidep/lib/python3.10/site-
packages/sklearn/neural_network/_multilayer_perceptron.py:690:
ConvergenceWarning: Stochastic Optimizer: Maximum iterations (1000) reached and
the optimization hasn't converged yet.
  warnings.warn(
/projects/proteinml/.links/miniconda3/envs/aidep/lib/python3.10/site-
packages/sklearn/neural_network/_multilayer_perceptron.py:690:
ConvergenceWarning: Stochastic Optimizer: Maximum iterations (1000) reached and
the optimization hasn't converged yet.
  warnings.warn(
/projects/proteinml/.links/miniconda3/envs/aidep/lib/python3.10/site-
packages/sklearn/neural_network/_multilayer_perceptron.py:690:
ConvergenceWarning: Stochastic Optimizer: Maximum iterations (1000) reached and
the optimization hasn't converged yet.
  warnings.warn(
/projects/proteinml/.links/miniconda3/envs/aidep/lib/python3.10/site-
packages/sklearn/neural_network/_multilayer_perceptron.py:690:
ConvergenceWarning: Stochastic Optimizer: Maximum iterations (1000) reached and
the optimization hasn't converged yet.
  warnings.warn(
/projects/proteinml/.links/miniconda3/envs/aidep/lib/python3.10/site-
packages/sklearn/neural_network/_multilayer_perceptron.py:690:
ConvergenceWarning: Stochastic Optimizer: Maximum iterations (1000) reached and
the optimization hasn't converged yet.
  warnings.warn(
/projects/proteinml/.links/miniconda3/envs/aidep/lib/python3.10/site-
packages/sklearn/neural_network/_multilayer_perceptron.py:690:
ConvergenceWarning: Stochastic Optimizer: Maximum iterations (1000) reached and
the optimization hasn't converged yet.
  warnings.warn(
/projects/proteinml/.links/miniconda3/envs/aidep/lib/python3.10/site-
packages/sklearn/neural_network/_multilayer_perceptron.py:690:
ConvergenceWarning: Stochastic Optimizer: Maximum iterations (1000) reached and
the optimization hasn't converged yet.
  warnings.warn(
/projects/proteinml/.links/miniconda3/envs/aidep/lib/python3.10/site-
packages/sklearn/neural_network/_multilayer_perceptron.py:690:
ConvergenceWarning: Stochastic Optimizer: Maximum iterations (1000) reached and
the optimization hasn't converged yet.
```

```
  warnings.warn(
/projects/proteinml/.links/miniconda3/envs/aidep/lib/python3.10/site-
packages/sklearn/neural_network/_multilayer_perceptron.py:690:
ConvergenceWarning: Stochastic Optimizer: Maximum iterations (1000) reached and
the optimization hasn't converged yet.
  warnings.warn(
/projects/proteinml/.links/miniconda3/envs/aidep/lib/python3.10/site-
packages/sklearn/neural_network/_multilayer_perceptron.py:690:
ConvergenceWarning: Stochastic Optimizer: Maximum iterations (1000) reached and
the optimization hasn't converged yet.
  warnings.warn(
/projects/proteinml/.links/miniconda3/envs/aidep/lib/python3.10/site-
packages/sklearn/neural_network/_multilayer_perceptron.py:690:
ConvergenceWarning: Stochastic Optimizer: Maximum iterations (1000) reached and
the optimization hasn't converged yet.
  warnings.warn(
/projects/proteinml/.links/miniconda3/envs/aidep/lib/python3.10/site-
packages/sklearn/neural_network/_multilayer_perceptron.py:690:
ConvergenceWarning: Stochastic Optimizer: Maximum iterations (1000) reached and
the optimization hasn't converged yet.
  warnings.warn(
/projects/proteinml/.links/miniconda3/envs/aidep/lib/python3.10/site-
packages/sklearn/neural_network/_multilayer_perceptron.py:690:
ConvergenceWarning: Stochastic Optimizer: Maximum iterations (1000) reached and
the optimization hasn't converged yet.
  warnings.warn(
/projects/proteinml/.links/miniconda3/envs/aidep/lib/python3.10/site-
packages/sklearn/neural_network/_multilayer_perceptron.py:690:
ConvergenceWarning: Stochastic Optimizer: Maximum iterations (1000) reached and
the optimization hasn't converged yet.
  warnings.warn(
/projects/proteinml/.links/miniconda3/envs/aidep/lib/python3.10/site-
packages/sklearn/neural_network/_multilayer_perceptron.py:690:
ConvergenceWarning: Stochastic Optimizer: Maximum iterations (1000) reached and
the optimization hasn't converged yet.
  warnings.warn(
/projects/proteinml/.links/miniconda3/envs/aidep/lib/python3.10/site-
packages/sklearn/neural_network/_multilayer_perceptron.py:690:
ConvergenceWarning: Stochastic Optimizer: Maximum iterations (1000) reached and
the optimization hasn't converged yet.
  warnings.warn(
/projects/proteinml/.links/miniconda3/envs/aidep/lib/python3.10/site-
packages/sklearn/neural_network/_multilayer_perceptron.py:690:
ConvergenceWarning: Stochastic Optimizer: Maximum iterations (1000) reached and
the optimization hasn't converged yet.
  warnings.warn(
/projects/proteinml/.links/miniconda3/envs/aidep/lib/python3.10/site-
packages/sklearn/neural_network/_multilayer_perceptron.py:690:
```

```
ConvergenceWarning: Stochastic Optimizer: Maximum iterations (1000) reached and
the optimization hasn't converged yet.
  warnings.warn(
/projects/proteinml/.links/miniconda3/envs/aidep/lib/python3.10/site-
packages/sklearn/neural_network/_multilayer_perceptron.py:690:
ConvergenceWarning: Stochastic Optimizer: Maximum iterations (1000) reached and
the optimization hasn't converged yet.
  warnings.warn(
/projects/proteinml/.links/miniconda3/envs/aidep/lib/python3.10/site-
packages/sklearn/neural_network/_multilayer_perceptron.py:690:
ConvergenceWarning: Stochastic Optimizer: Maximum iterations (1000) reached and
the optimization hasn't converged yet.
  warnings.warn(
/projects/proteinml/.links/miniconda3/envs/aidep/lib/python3.10/site-
packages/sklearn/neural_network/_multilayer_perceptron.py:690:
ConvergenceWarning: Stochastic Optimizer: Maximum iterations (1000) reached and
the optimization hasn't converged yet.
  warnings.warn(
/projects/proteinml/.links/miniconda3/envs/aidep/lib/python3.10/site-
packages/sklearn/neural_network/_multilayer_perceptron.py:690:
ConvergenceWarning: Stochastic Optimizer: Maximum iterations (1000) reached and
the optimization hasn't converged yet.
  warnings.warn(
/projects/proteinml/.links/miniconda3/envs/aidep/lib/python3.10/site-
packages/sklearn/neural_network/_multilayer_perceptron.py:690:
ConvergenceWarning: Stochastic Optimizer: Maximum iterations (1000) reached and
the optimization hasn't converged yet.
  warnings.warn(
/projects/proteinml/.links/miniconda3/envs/aidep/lib/python3.10/site-
packages/sklearn/neural_network/_multilayer_perceptron.py:690:
ConvergenceWarning: Stochastic Optimizer: Maximum iterations (1000) reached and
the optimization hasn't converged yet.
  warnings.warn(
/projects/proteinml/.links/miniconda3/envs/aidep/lib/python3.10/site-
packages/sklearn/neural_network/_multilayer_perceptron.py:690:
ConvergenceWarning: Stochastic Optimizer: Maximum iterations (1000) reached and
the optimization hasn't converged yet.
  warnings.warn(
/projects/proteinml/.links/miniconda3/envs/aidep/lib/python3.10/site-
packages/sklearn/neural_network/_multilayer_perceptron.py:690:
ConvergenceWarning: Stochastic Optimizer: Maximum iterations (1000) reached and
the optimization hasn't converged yet.
  warnings.warn(
/projects/proteinml/.links/miniconda3/envs/aidep/lib/python3.10/site-
packages/sklearn/neural_network/_multilayer_perceptron.py:690:
ConvergenceWarning: Stochastic Optimizer: Maximum iterations (1000) reached and
the optimization hasn't converged yet.
  warnings.warn(
```

```
/projects/proteinml/.links/miniconda3/envs/aidep/lib/python3.10/site-
packages/sklearn/neural_network/_multilayer_perceptron.py:690:
ConvergenceWarning: Stochastic Optimizer: Maximum iterations (1000) reached and
the optimization hasn't converged yet.
  warnings.warn(
/projects/proteinml/.links/miniconda3/envs/aidep/lib/python3.10/site-
packages/sklearn/neural_network/_multilayer_perceptron.py:690:
ConvergenceWarning: Stochastic Optimizer: Maximum iterations (1000) reached and
the optimization hasn't converged yet.
  warnings.warn(
/projects/proteinml/.links/miniconda3/envs/aidep/lib/python3.10/site-
packages/sklearn/neural_network/_multilayer_perceptron.py:690:
ConvergenceWarning: Stochastic Optimizer: Maximum iterations (1000) reached and
the optimization hasn't converged yet.
  warnings.warn(
/projects/proteinml/.links/miniconda3/envs/aidep/lib/python3.10/site-
packages/sklearn/neural_network/_multilayer_perceptron.py:690:
ConvergenceWarning: Stochastic Optimizer: Maximum iterations (1000) reached and
the optimization hasn't converged yet.
  warnings.warn(
/projects/proteinml/.links/miniconda3/envs/aidep/lib/python3.10/site-
packages/sklearn/neural_network/_multilayer_perceptron.py:690:
ConvergenceWarning: Stochastic Optimizer: Maximum iterations (1000) reached and
the optimization hasn't converged yet.
  warnings.warn(
/projects/proteinml/.links/miniconda3/envs/aidep/lib/python3.10/site-
packages/sklearn/neural_network/_multilayer_perceptron.py:690:
ConvergenceWarning: Stochastic Optimizer: Maximum iterations (1000) reached and
the optimization hasn't converged yet.
  warnings.warn(
/projects/proteinml/.links/miniconda3/envs/aidep/lib/python3.10/site-
packages/sklearn/neural_network/_multilayer_perceptron.py:690:
ConvergenceWarning: Stochastic Optimizer: Maximum iterations (1000) reached and
the optimization hasn't converged yet.
  warnings.warn(
/projects/proteinml/.links/miniconda3/envs/aidep/lib/python3.10/site-
packages/sklearn/neural_network/_multilayer_perceptron.py:690:
ConvergenceWarning: Stochastic Optimizer: Maximum iterations (1000) reached and
the optimization hasn't converged yet.
  warnings.warn(
/projects/proteinml/.links/miniconda3/envs/aidep/lib/python3.10/site-
packages/sklearn/neural_network/_multilayer_perceptron.py:690:
ConvergenceWarning: Stochastic Optimizer: Maximum iterations (1000) reached and
the optimization hasn't converged yet.
  warnings.warn(
/projects/proteinml/.links/miniconda3/envs/aidep/lib/python3.10/site-
packages/sklearn/neural_network/_multilayer_perceptron.py:690:
ConvergenceWarning: Stochastic Optimizer: Maximum iterations (1000) reached and
```

```
the optimization hasn't converged yet.
  warnings.warn(
/projects/proteinml/.links/miniconda3/envs/aidep/lib/python3.10/site-
packages/sklearn/neural_network/_multilayer_perceptron.py:690:
ConvergenceWarning: Stochastic Optimizer: Maximum iterations (1000) reached and
the optimization hasn't converged yet.
  warnings.warn(
/projects/proteinml/.links/miniconda3/envs/aidep/lib/python3.10/site-
packages/sklearn/neural_network/_multilayer_perceptron.py:690:
ConvergenceWarning: Stochastic Optimizer: Maximum iterations (1000) reached and
the optimization hasn't converged yet.
  warnings.warn(
/projects/proteinml/.links/miniconda3/envs/aidep/lib/python3.10/site-
packages/sklearn/neural_network/_multilayer_perceptron.py:690:
ConvergenceWarning: Stochastic Optimizer: Maximum iterations (1000) reached and
the optimization hasn't converged yet.
  warnings.warn(
/projects/proteinml/.links/miniconda3/envs/aidep/lib/python3.10/site-
packages/sklearn/neural_network/_multilayer_perceptron.py:690:
ConvergenceWarning: Stochastic Optimizer: Maximum iterations (1000) reached and
the optimization hasn't converged yet.
  warnings.warn(
/projects/proteinml/.links/miniconda3/envs/aidep/lib/python3.10/site-
packages/sklearn/neural_network/_multilayer_perceptron.py:690:
ConvergenceWarning: Stochastic Optimizer: Maximum iterations (1000) reached and
the optimization hasn't converged yet.
  warnings.warn(
/projects/proteinml/.links/miniconda3/envs/aidep/lib/python3.10/site-
packages/sklearn/neural_network/_multilayer_perceptron.py:690:
ConvergenceWarning: Stochastic Optimizer: Maximum iterations (1000) reached and
the optimization hasn't converged yet.
  warnings.warn(
/projects/proteinml/.links/miniconda3/envs/aidep/lib/python3.10/site-
packages/sklearn/neural_network/_multilayer_perceptron.py:690:
ConvergenceWarning: Stochastic Optimizer: Maximum iterations (1000) reached and
the optimization hasn't converged yet.
  warnings.warn(
/projects/proteinml/.links/miniconda3/envs/aidep/lib/python3.10/site-
packages/sklearn/neural_network/_multilayer_perceptron.py:690:
ConvergenceWarning: Stochastic Optimizer: Maximum iterations (1000) reached and
the optimization hasn't converged yet.
  warnings.warn(
/projects/proteinml/.links/miniconda3/envs/aidep/lib/python3.10/site-
packages/sklearn/neural_network/_multilayer_perceptron.py:690:
ConvergenceWarning: Stochastic Optimizer: Maximum iterations (1000) reached and
the optimization hasn't converged yet.
  warnings.warn(
/projects/proteinml/.links/miniconda3/envs/aidep/lib/python3.10/site-
```

```
packages/sklearn/neural_network/_multilayer_perceptron.py:690:
ConvergenceWarning: Stochastic Optimizer: Maximum iterations (1000) reached and
the optimization hasn't converged yet.
  warnings.warn(
/projects/proteinml/.links/miniconda3/envs/aidep/lib/python3.10/site-
packages/sklearn/neural_network/_multilayer_perceptron.py:690:
ConvergenceWarning: Stochastic Optimizer: Maximum iterations (1000) reached and
the optimization hasn't converged yet.
  warnings.warn(
/projects/proteinml/.links/miniconda3/envs/aidep/lib/python3.10/site-
packages/sklearn/neural_network/_multilayer_perceptron.py:690:
ConvergenceWarning: Stochastic Optimizer: Maximum iterations (1000) reached and
the optimization hasn't converged yet.
  warnings.warn(
/projects/proteinml/.links/miniconda3/envs/aidep/lib/python3.10/site-
packages/sklearn/neural_network/_multilayer_perceptron.py:690:
ConvergenceWarning: Stochastic Optimizer: Maximum iterations (1000) reached and
the optimization hasn't converged yet.
  warnings.warn(
/projects/proteinml/.links/miniconda3/envs/aidep/lib/python3.10/site-
packages/sklearn/neural_network/_multilayer_perceptron.py:690:
ConvergenceWarning: Stochastic Optimizer: Maximum iterations (1000) reached and
the optimization hasn't converged yet.
  warnings.warn(
/projects/proteinml/.links/miniconda3/envs/aidep/lib/python3.10/site-
packages/sklearn/neural_network/_multilayer_perceptron.py:690:
ConvergenceWarning: Stochastic Optimizer: Maximum iterations (1000) reached and
the optimization hasn't converged yet.
  warnings.warn(
/projects/proteinml/.links/miniconda3/envs/aidep/lib/python3.10/site-
packages/sklearn/neural_network/_multilayer_perceptron.py:690:
ConvergenceWarning: Stochastic Optimizer: Maximum iterations (1000) reached and
the optimization hasn't converged yet.
  warnings.warn(
/projects/proteinml/.links/miniconda3/envs/aidep/lib/python3.10/site-
packages/sklearn/neural_network/_multilayer_perceptron.py:690:
ConvergenceWarning: Stochastic Optimizer: Maximum iterations (1000) reached and
the optimization hasn't converged yet.
  warnings.warn(
/projects/proteinml/.links/miniconda3/envs/aidep/lib/python3.10/site-
packages/sklearn/neural_network/_multilayer_perceptron.py:690:
ConvergenceWarning: Stochastic Optimizer: Maximum iterations (1000) reached and
the optimization hasn't converged yet.
  warnings.warn(
/projects/proteinml/.links/miniconda3/envs/aidep/lib/python3.10/site-
packages/sklearn/neural_network/_multilayer_perceptron.py:690:
ConvergenceWarning: Stochastic Optimizer: Maximum iterations (1000) reached and
the optimization hasn't converged yet.
```

```
  warnings.warn(
/projects/proteinml/.links/miniconda3/envs/aidep/lib/python3.10/site-
packages/sklearn/neural_network/_multilayer_perceptron.py:690:
ConvergenceWarning: Stochastic Optimizer: Maximum iterations (1000) reached and
the optimization hasn't converged yet.
  warnings.warn(
/projects/proteinml/.links/miniconda3/envs/aidep/lib/python3.10/site-
packages/sklearn/neural_network/_multilayer_perceptron.py:690:
ConvergenceWarning: Stochastic Optimizer: Maximum iterations (1000) reached and
the optimization hasn't converged yet.
  warnings.warn(
/projects/proteinml/.links/miniconda3/envs/aidep/lib/python3.10/site-
packages/sklearn/neural_network/_multilayer_perceptron.py:690:
ConvergenceWarning: Stochastic Optimizer: Maximum iterations (1000) reached and
the optimization hasn't converged yet.
  warnings.warn(
/projects/proteinml/.links/miniconda3/envs/aidep/lib/python3.10/site-
packages/sklearn/neural_network/_multilayer_perceptron.py:690:
ConvergenceWarning: Stochastic Optimizer: Maximum iterations (1000) reached and
the optimization hasn't converged yet.
  warnings.warn(
/projects/proteinml/.links/miniconda3/envs/aidep/lib/python3.10/site-
packages/sklearn/neural_network/_multilayer_perceptron.py:690:
ConvergenceWarning: Stochastic Optimizer: Maximum iterations (1000) reached and
the optimization hasn't converged yet.
  warnings.warn(
/projects/proteinml/.links/miniconda3/envs/aidep/lib/python3.10/site-
packages/sklearn/neural_network/_multilayer_perceptron.py:690:
ConvergenceWarning: Stochastic Optimizer: Maximum iterations (1000) reached and
the optimization hasn't converged yet.
  warnings.warn(
/projects/proteinml/.links/miniconda3/envs/aidep/lib/python3.10/site-
packages/sklearn/neural_network/_multilayer_perceptron.py:690:
ConvergenceWarning: Stochastic Optimizer: Maximum iterations (1000) reached and
the optimization hasn't converged yet.
  warnings.warn(
/projects/proteinml/.links/miniconda3/envs/aidep/lib/python3.10/site-
packages/sklearn/neural_network/_multilayer_perceptron.py:690:
ConvergenceWarning: Stochastic Optimizer: Maximum iterations (1000) reached and
the optimization hasn't converged yet.
  warnings.warn(
/projects/proteinml/.links/miniconda3/envs/aidep/lib/python3.10/site-
packages/sklearn/neural_network/_multilayer_perceptron.py:690:
ConvergenceWarning: Stochastic Optimizer: Maximum iterations (1000) reached and
the optimization hasn't converged yet.
  warnings.warn(
/projects/proteinml/.links/miniconda3/envs/aidep/lib/python3.10/site-
packages/sklearn/neural_network/_multilayer_perceptron.py:690:
```

```
ConvergenceWarning: Stochastic Optimizer: Maximum iterations (1000) reached and
the optimization hasn't converged yet.
  warnings.warn(
/projects/proteinml/.links/miniconda3/envs/aidep/lib/python3.10/site-
packages/sklearn/neural_network/_multilayer_perceptron.py:690:
ConvergenceWarning: Stochastic Optimizer: Maximum iterations (1000) reached and
the optimization hasn't converged yet.
  warnings.warn(
/projects/proteinml/.links/miniconda3/envs/aidep/lib/python3.10/site-
packages/sklearn/neural_network/_multilayer_perceptron.py:690:
ConvergenceWarning: Stochastic Optimizer: Maximum iterations (1000) reached and
the optimization hasn't converged yet.
  warnings.warn(
/projects/proteinml/.links/miniconda3/envs/aidep/lib/python3.10/site-
packages/sklearn/neural_network/_multilayer_perceptron.py:690:
ConvergenceWarning: Stochastic Optimizer: Maximum iterations (1000) reached and
the optimization hasn't converged yet.
  warnings.warn(
/projects/proteinml/.links/miniconda3/envs/aidep/lib/python3.10/site-
packages/sklearn/neural_network/_multilayer_perceptron.py:690:
ConvergenceWarning: Stochastic Optimizer: Maximum iterations (1000) reached and
the optimization hasn't converged yet.
  warnings.warn(
/projects/proteinml/.links/miniconda3/envs/aidep/lib/python3.10/site-
packages/sklearn/neural_network/_multilayer_perceptron.py:690:
ConvergenceWarning: Stochastic Optimizer: Maximum iterations (1000) reached and
the optimization hasn't converged yet.
  warnings.warn(
/projects/proteinml/.links/miniconda3/envs/aidep/lib/python3.10/site-
packages/sklearn/neural_network/_multilayer_perceptron.py:690:
ConvergenceWarning: Stochastic Optimizer: Maximum iterations (1000) reached and
the optimization hasn't converged yet.
  warnings.warn(
/projects/proteinml/.links/miniconda3/envs/aidep/lib/python3.10/site-
packages/sklearn/neural_network/_multilayer_perceptron.py:690:
ConvergenceWarning: Stochastic Optimizer: Maximum iterations (1000) reached and
the optimization hasn't converged yet.
  warnings.warn(
/projects/proteinml/.links/miniconda3/envs/aidep/lib/python3.10/site-
packages/sklearn/neural_network/_multilayer_perceptron.py:690:
ConvergenceWarning: Stochastic Optimizer: Maximum iterations (1000) reached and
the optimization hasn't converged yet.
  warnings.warn(
/projects/proteinml/.links/miniconda3/envs/aidep/lib/python3.10/site-
packages/sklearn/neural_network/_multilayer_perceptron.py:690:
ConvergenceWarning: Stochastic Optimizer: Maximum iterations (1000) reached and
the optimization hasn't converged yet.
  warnings.warn(
```

```
/projects/proteinml/.links/miniconda3/envs/aidep/lib/python3.10/site-
packages/sklearn/neural_network/_multilayer_perceptron.py:690:
ConvergenceWarning: Stochastic Optimizer: Maximum iterations (1000) reached and
the optimization hasn't converged yet.
  warnings.warn(
/projects/proteinml/.links/miniconda3/envs/aidep/lib/python3.10/site-
packages/sklearn/neural_network/_multilayer_perceptron.py:690:
ConvergenceWarning: Stochastic Optimizer: Maximum iterations (1000) reached and
the optimization hasn't converged yet.
  warnings.warn(
/projects/proteinml/.links/miniconda3/envs/aidep/lib/python3.10/site-
packages/sklearn/neural_network/_multilayer_perceptron.py:690:
ConvergenceWarning: Stochastic Optimizer: Maximum iterations (1000) reached and
the optimization hasn't converged yet.
  warnings.warn(
/projects/proteinml/.links/miniconda3/envs/aidep/lib/python3.10/site-
packages/sklearn/neural_network/_multilayer_perceptron.py:690:
ConvergenceWarning: Stochastic Optimizer: Maximum iterations (1000) reached and
the optimization hasn't converged yet.
  warnings.warn(
/projects/proteinml/.links/miniconda3/envs/aidep/lib/python3.10/site-
packages/sklearn/neural_network/_multilayer_perceptron.py:690:
ConvergenceWarning: Stochastic Optimizer: Maximum iterations (1000) reached and
the optimization hasn't converged yet.
  warnings.warn(
/projects/proteinml/.links/miniconda3/envs/aidep/lib/python3.10/site-
packages/sklearn/neural_network/_multilayer_perceptron.py:690:
ConvergenceWarning: Stochastic Optimizer: Maximum iterations (1000) reached and
the optimization hasn't converged yet.
  warnings.warn(
/projects/proteinml/.links/miniconda3/envs/aidep/lib/python3.10/site-
packages/sklearn/neural_network/_multilayer_perceptron.py:690:
ConvergenceWarning: Stochastic Optimizer: Maximum iterations (1000) reached and
the optimization hasn't converged yet.
  warnings.warn(
/projects/proteinml/.links/miniconda3/envs/aidep/lib/python3.10/site-
packages/sklearn/neural_network/_multilayer_perceptron.py:690:
ConvergenceWarning: Stochastic Optimizer: Maximum iterations (1000) reached and
the optimization hasn't converged yet.
  warnings.warn(
/projects/proteinml/.links/miniconda3/envs/aidep/lib/python3.10/site-
packages/sklearn/neural_network/_multilayer_perceptron.py:690:
ConvergenceWarning: Stochastic Optimizer: Maximum iterations (1000) reached and
the optimization hasn't converged yet.
  warnings.warn(
/projects/proteinml/.links/miniconda3/envs/aidep/lib/python3.10/site-
packages/sklearn/neural_network/_multilayer_perceptron.py:690:
ConvergenceWarning: Stochastic Optimizer: Maximum iterations (1000) reached and
```

```
the optimization hasn't converged yet.
  warnings.warn(
/projects/proteinml/.links/miniconda3/envs/aidep/lib/python3.10/site-
packages/sklearn/neural_network/_multilayer_perceptron.py:690:
ConvergenceWarning: Stochastic Optimizer: Maximum iterations (1000) reached and
the optimization hasn't converged yet.
  warnings.warn(
/projects/proteinml/.links/miniconda3/envs/aidep/lib/python3.10/site-
packages/sklearn/neural_network/_multilayer_perceptron.py:690:
ConvergenceWarning: Stochastic Optimizer: Maximum iterations (1000) reached and
the optimization hasn't converged yet.
  warnings.warn(
/projects/proteinml/.links/miniconda3/envs/aidep/lib/python3.10/site-
packages/sklearn/neural_network/_multilayer_perceptron.py:690:
ConvergenceWarning: Stochastic Optimizer: Maximum iterations (1000) reached and
the optimization hasn't converged yet.
  warnings.warn(
/projects/proteinml/.links/miniconda3/envs/aidep/lib/python3.10/site-
packages/sklearn/neural_network/_multilayer_perceptron.py:690:
ConvergenceWarning: Stochastic Optimizer: Maximum iterations (1000) reached and
the optimization hasn't converged yet.
  warnings.warn(
/projects/proteinml/.links/miniconda3/envs/aidep/lib/python3.10/site-
packages/sklearn/neural_network/_multilayer_perceptron.py:690:
ConvergenceWarning: Stochastic Optimizer: Maximum iterations (1000) reached and
the optimization hasn't converged yet.
  warnings.warn(
/projects/proteinml/.links/miniconda3/envs/aidep/lib/python3.10/site-
packages/sklearn/neural_network/_multilayer_perceptron.py:690:
ConvergenceWarning: Stochastic Optimizer: Maximum iterations (1000) reached and
the optimization hasn't converged yet.
  warnings.warn(
/projects/proteinml/.links/miniconda3/envs/aidep/lib/python3.10/site-
packages/sklearn/neural_network/_multilayer_perceptron.py:690:
ConvergenceWarning: Stochastic Optimizer: Maximum iterations (1000) reached and
the optimization hasn't converged yet.
  warnings.warn(
/projects/proteinml/.links/miniconda3/envs/aidep/lib/python3.10/site-
packages/sklearn/neural_network/_multilayer_perceptron.py:690:
ConvergenceWarning: Stochastic Optimizer: Maximum iterations (1000) reached and
the optimization hasn't converged yet.
  warnings.warn(
/projects/proteinml/.links/miniconda3/envs/aidep/lib/python3.10/site-
packages/sklearn/neural_network/_multilayer_perceptron.py:690:
ConvergenceWarning: Stochastic Optimizer: Maximum iterations (1000) reached and
the optimization hasn't converged yet.
  warnings.warn(
/projects/proteinml/.links/miniconda3/envs/aidep/lib/python3.10/site-
```

```
packages/sklearn/neural_network/_multilayer_perceptron.py:690:
ConvergenceWarning: Stochastic Optimizer: Maximum iterations (1000) reached and
the optimization hasn't converged yet.
  warnings.warn(
/projects/proteinml/.links/miniconda3/envs/aidep/lib/python3.10/site-
packages/sklearn/neural_network/_multilayer_perceptron.py:690:
ConvergenceWarning: Stochastic Optimizer: Maximum iterations (1000) reached and
the optimization hasn't converged yet.
  warnings.warn(
/projects/proteinml/.links/miniconda3/envs/aidep/lib/python3.10/site-
packages/sklearn/neural_network/_multilayer_perceptron.py:690:
ConvergenceWarning: Stochastic Optimizer: Maximum iterations (1000) reached and
the optimization hasn't converged yet.
  warnings.warn(
/projects/proteinml/.links/miniconda3/envs/aidep/lib/python3.10/site-
packages/sklearn/neural_network/_multilayer_perceptron.py:690:
ConvergenceWarning: Stochastic Optimizer: Maximum iterations (1000) reached and
the optimization hasn't converged yet.
  warnings.warn(
/projects/proteinml/.links/miniconda3/envs/aidep/lib/python3.10/site-
packages/sklearn/neural_network/_multilayer_perceptron.py:690:
ConvergenceWarning: Stochastic Optimizer: Maximum iterations (1000) reached and
the optimization hasn't converged yet.
  warnings.warn(
/projects/proteinml/.links/miniconda3/envs/aidep/lib/python3.10/site-
packages/sklearn/neural_network/_multilayer_perceptron.py:690:
ConvergenceWarning: Stochastic Optimizer: Maximum iterations (1000) reached and
the optimization hasn't converged yet.
  warnings.warn(
/projects/proteinml/.links/miniconda3/envs/aidep/lib/python3.10/site-
packages/sklearn/neural_network/_multilayer_perceptron.py:690:
ConvergenceWarning: Stochastic Optimizer: Maximum iterations (1000) reached and
the optimization hasn't converged yet.
  warnings.warn(
/projects/proteinml/.links/miniconda3/envs/aidep/lib/python3.10/site-
packages/sklearn/neural_network/_multilayer_perceptron.py:690:
ConvergenceWarning: Stochastic Optimizer: Maximum iterations (1000) reached and
the optimization hasn't converged yet.
  warnings.warn(
/projects/proteinml/.links/miniconda3/envs/aidep/lib/python3.10/site-
packages/sklearn/neural_network/_multilayer_perceptron.py:690:
ConvergenceWarning: Stochastic Optimizer: Maximum iterations (1000) reached and
the optimization hasn't converged yet.
  warnings.warn(
/projects/proteinml/.links/miniconda3/envs/aidep/lib/python3.10/site-
packages/sklearn/neural_network/_multilayer_perceptron.py:690:
ConvergenceWarning: Stochastic Optimizer: Maximum iterations (1000) reached and
the optimization hasn't converged yet.
```

```
  warnings.warn(
/projects/proteinml/.links/miniconda3/envs/aidep/lib/python3.10/site-
packages/sklearn/neural_network/_multilayer_perceptron.py:690:
ConvergenceWarning: Stochastic Optimizer: Maximum iterations (1000) reached and
the optimization hasn't converged yet.
  warnings.warn(
/projects/proteinml/.links/miniconda3/envs/aidep/lib/python3.10/site-
packages/sklearn/neural_network/_multilayer_perceptron.py:690:
ConvergenceWarning: Stochastic Optimizer: Maximum iterations (1000) reached and
the optimization hasn't converged yet.
  warnings.warn(
/projects/proteinml/.links/miniconda3/envs/aidep/lib/python3.10/site-
packages/sklearn/neural_network/_multilayer_perceptron.py:690:
ConvergenceWarning: Stochastic Optimizer: Maximum iterations (1000) reached and
the optimization hasn't converged yet.
  warnings.warn(
/projects/proteinml/.links/miniconda3/envs/aidep/lib/python3.10/site-
packages/sklearn/neural_network/_multilayer_perceptron.py:690:
ConvergenceWarning: Stochastic Optimizer: Maximum iterations (1000) reached and
the optimization hasn't converged yet.
  warnings.warn(
/projects/proteinml/.links/miniconda3/envs/aidep/lib/python3.10/site-
packages/sklearn/neural_network/_multilayer_perceptron.py:690:
ConvergenceWarning: Stochastic Optimizer: Maximum iterations (1000) reached and
the optimization hasn't converged yet.
  warnings.warn(
/projects/proteinml/.links/miniconda3/envs/aidep/lib/python3.10/site-
packages/sklearn/neural_network/_multilayer_perceptron.py:690:
ConvergenceWarning: Stochastic Optimizer: Maximum iterations (1000) reached and
the optimization hasn't converged yet.
  warnings.warn(
/projects/proteinml/.links/miniconda3/envs/aidep/lib/python3.10/site-
packages/sklearn/neural_network/_multilayer_perceptron.py:690:
ConvergenceWarning: Stochastic Optimizer: Maximum iterations (1000) reached and
the optimization hasn't converged yet.
  warnings.warn(
/projects/proteinml/.links/miniconda3/envs/aidep/lib/python3.10/site-
packages/sklearn/neural_network/_multilayer_perceptron.py:690:
ConvergenceWarning: Stochastic Optimizer: Maximum iterations (1000) reached and
the optimization hasn't converged yet.
  warnings.warn(
/projects/proteinml/.links/miniconda3/envs/aidep/lib/python3.10/site-
packages/sklearn/neural_network/_multilayer_perceptron.py:690:
ConvergenceWarning: Stochastic Optimizer: Maximum iterations (1000) reached and
the optimization hasn't converged yet.
  warnings.warn(
/projects/proteinml/.links/miniconda3/envs/aidep/lib/python3.10/site-
packages/sklearn/neural_network/_multilayer_perceptron.py:690:
```

```
ConvergenceWarning: Stochastic Optimizer: Maximum iterations (1000) reached and
the optimization hasn't converged yet.
  warnings.warn(
/projects/proteinml/.links/miniconda3/envs/aidep/lib/python3.10/site-
packages/sklearn/neural_network/_multilayer_perceptron.py:690:
ConvergenceWarning: Stochastic Optimizer: Maximum iterations (1000) reached and
the optimization hasn't converged yet.
  warnings.warn(
/projects/proteinml/.links/miniconda3/envs/aidep/lib/python3.10/site-
packages/sklearn/neural_network/_multilayer_perceptron.py:690:
ConvergenceWarning: Stochastic Optimizer: Maximum iterations (1000) reached and
the optimization hasn't converged yet.
  warnings.warn(
/projects/proteinml/.links/miniconda3/envs/aidep/lib/python3.10/site-
packages/sklearn/neural_network/_multilayer_perceptron.py:690:
ConvergenceWarning: Stochastic Optimizer: Maximum iterations (1000) reached and
the optimization hasn't converged yet.
  warnings.warn(
/projects/proteinml/.links/miniconda3/envs/aidep/lib/python3.10/site-
packages/sklearn/neural_network/_multilayer_perceptron.py:690:
ConvergenceWarning: Stochastic Optimizer: Maximum iterations (1000) reached and
the optimization hasn't converged yet.
  warnings.warn(
/projects/proteinml/.links/miniconda3/envs/aidep/lib/python3.10/site-
packages/sklearn/neural_network/_multilayer_perceptron.py:690:
ConvergenceWarning: Stochastic Optimizer: Maximum iterations (1000) reached and
the optimization hasn't converged yet.
  warnings.warn(
/projects/proteinml/.links/miniconda3/envs/aidep/lib/python3.10/site-
packages/sklearn/neural_network/_multilayer_perceptron.py:690:
ConvergenceWarning: Stochastic Optimizer: Maximum iterations (1000) reached and
the optimization hasn't converged yet.
  warnings.warn(
/projects/proteinml/.links/miniconda3/envs/aidep/lib/python3.10/site-
packages/sklearn/neural_network/_multilayer_perceptron.py:690:
ConvergenceWarning: Stochastic Optimizer: Maximum iterations (1000) reached and
the optimization hasn't converged yet.
  warnings.warn(
/projects/proteinml/.links/miniconda3/envs/aidep/lib/python3.10/site-
packages/sklearn/neural_network/_multilayer_perceptron.py:690:
ConvergenceWarning: Stochastic Optimizer: Maximum iterations (1000) reached and
the optimization hasn't converged yet.
  warnings.warn(
/projects/proteinml/.links/miniconda3/envs/aidep/lib/python3.10/site-
packages/sklearn/neural_network/_multilayer_perceptron.py:690:
ConvergenceWarning: Stochastic Optimizer: Maximum iterations (1000) reached and
the optimization hasn't converged yet.
  warnings.warn(
```

```
/projects/proteinml/.links/miniconda3/envs/aidep/lib/python3.10/site-
packages/sklearn/neural_network/_multilayer_perceptron.py:690:
ConvergenceWarning: Stochastic Optimizer: Maximum iterations (1000) reached and
the optimization hasn't converged yet.
  warnings.warn(
/projects/proteinml/.links/miniconda3/envs/aidep/lib/python3.10/site-
packages/sklearn/neural_network/_multilayer_perceptron.py:690:
ConvergenceWarning: Stochastic Optimizer: Maximum iterations (1000) reached and
the optimization hasn't converged yet.
  warnings.warn(
/projects/proteinml/.links/miniconda3/envs/aidep/lib/python3.10/site-
packages/sklearn/neural_network/_multilayer_perceptron.py:690:
ConvergenceWarning: Stochastic Optimizer: Maximum iterations (1000) reached and
the optimization hasn't converged yet.
  warnings.warn(
/projects/proteinml/.links/miniconda3/envs/aidep/lib/python3.10/site-
packages/sklearn/neural_network/_multilayer_perceptron.py:690:
ConvergenceWarning: Stochastic Optimizer: Maximum iterations (1000) reached and
the optimization hasn't converged yet.
  warnings.warn(
/projects/proteinml/.links/miniconda3/envs/aidep/lib/python3.10/site-
packages/sklearn/neural_network/_multilayer_perceptron.py:690:
ConvergenceWarning: Stochastic Optimizer: Maximum iterations (1000) reached and
the optimization hasn't converged yet.
  warnings.warn(
/projects/proteinml/.links/miniconda3/envs/aidep/lib/python3.10/site-
packages/sklearn/neural_network/_multilayer_perceptron.py:690:
ConvergenceWarning: Stochastic Optimizer: Maximum iterations (1000) reached and
the optimization hasn't converged yet.
  warnings.warn(
/projects/proteinml/.links/miniconda3/envs/aidep/lib/python3.10/site-
packages/sklearn/neural_network/_multilayer_perceptron.py:690:
ConvergenceWarning: Stochastic Optimizer: Maximum iterations (1000) reached and
the optimization hasn't converged yet.
  warnings.warn(
/projects/proteinml/.links/miniconda3/envs/aidep/lib/python3.10/site-
packages/sklearn/neural_network/_multilayer_perceptron.py:690:
ConvergenceWarning: Stochastic Optimizer: Maximum iterations (1000) reached and
the optimization hasn't converged yet.
  warnings.warn(
/projects/proteinml/.links/miniconda3/envs/aidep/lib/python3.10/site-
packages/sklearn/neural_network/_multilayer_perceptron.py:690:
ConvergenceWarning: Stochastic Optimizer: Maximum iterations (1000) reached and
the optimization hasn't converged yet.
  warnings.warn(
/projects/proteinml/.links/miniconda3/envs/aidep/lib/python3.10/site-
packages/sklearn/neural_network/_multilayer_perceptron.py:690:
ConvergenceWarning: Stochastic Optimizer: Maximum iterations (1000) reached and
```

```
the optimization hasn't converged yet.
  warnings.warn(
/projects/proteinml/.links/miniconda3/envs/aidep/lib/python3.10/site-
packages/sklearn/neural_network/_multilayer_perceptron.py:690:
ConvergenceWarning: Stochastic Optimizer: Maximum iterations (1000) reached and
the optimization hasn't converged yet.
  warnings.warn(
/projects/proteinml/.links/miniconda3/envs/aidep/lib/python3.10/site-
packages/sklearn/neural_network/_multilayer_perceptron.py:690:
ConvergenceWarning: Stochastic Optimizer: Maximum iterations (1000) reached and
the optimization hasn't converged yet.
  warnings.warn(
/projects/proteinml/.links/miniconda3/envs/aidep/lib/python3.10/site-
packages/sklearn/neural_network/_multilayer_perceptron.py:690:
ConvergenceWarning: Stochastic Optimizer: Maximum iterations (1000) reached and
the optimization hasn't converged yet.
  warnings.warn(
/projects/proteinml/.links/miniconda3/envs/aidep/lib/python3.10/site-
packages/sklearn/neural_network/_multilayer_perceptron.py:690:
ConvergenceWarning: Stochastic Optimizer: Maximum iterations (1000) reached and
the optimization hasn't converged yet.
  warnings.warn(
/projects/proteinml/.links/miniconda3/envs/aidep/lib/python3.10/site-
packages/sklearn/neural_network/_multilayer_perceptron.py:690:
ConvergenceWarning: Stochastic Optimizer: Maximum iterations (1000) reached and
the optimization hasn't converged yet.
  warnings.warn(
/projects/proteinml/.links/miniconda3/envs/aidep/lib/python3.10/site-
packages/sklearn/neural_network/_multilayer_perceptron.py:690:
ConvergenceWarning: Stochastic Optimizer: Maximum iterations (1000) reached and
the optimization hasn't converged yet.
  warnings.warn(
/projects/proteinml/.links/miniconda3/envs/aidep/lib/python3.10/site-
packages/sklearn/neural_network/_multilayer_perceptron.py:690:
ConvergenceWarning: Stochastic Optimizer: Maximum iterations (1000) reached and
the optimization hasn't converged yet.
  warnings.warn(
/projects/proteinml/.links/miniconda3/envs/aidep/lib/python3.10/site-
packages/sklearn/neural_network/_multilayer_perceptron.py:690:
ConvergenceWarning: Stochastic Optimizer: Maximum iterations (1000) reached and
the optimization hasn't converged yet.
  warnings.warn(
/projects/proteinml/.links/miniconda3/envs/aidep/lib/python3.10/site-
packages/sklearn/neural_network/_multilayer_perceptron.py:690:
ConvergenceWarning: Stochastic Optimizer: Maximum iterations (1000) reached and
the optimization hasn't converged yet.
  warnings.warn(
/projects/proteinml/.links/miniconda3/envs/aidep/lib/python3.10/site-
```

```
packages/sklearn/neural_network/_multilayer_perceptron.py:690:
ConvergenceWarning: Stochastic Optimizer: Maximum iterations (1000) reached and
the optimization hasn't converged yet.
  warnings.warn(
/projects/proteinml/.links/miniconda3/envs/aidep/lib/python3.10/site-
packages/sklearn/neural_network/_multilayer_perceptron.py:690:
ConvergenceWarning: Stochastic Optimizer: Maximum iterations (1000) reached and
the optimization hasn't converged yet.
  warnings.warn(
/projects/proteinml/.links/miniconda3/envs/aidep/lib/python3.10/site-
packages/sklearn/neural_network/_multilayer_perceptron.py:690:
ConvergenceWarning: Stochastic Optimizer: Maximum iterations (1000) reached and
the optimization hasn't converged yet.
  warnings.warn(
/projects/proteinml/.links/miniconda3/envs/aidep/lib/python3.10/site-
packages/sklearn/neural_network/_multilayer_perceptron.py:690:
ConvergenceWarning: Stochastic Optimizer: Maximum iterations (1000) reached and
the optimization hasn't converged yet.
  warnings.warn(
/projects/proteinml/.links/miniconda3/envs/aidep/lib/python3.10/site-
packages/sklearn/neural_network/_multilayer_perceptron.py:690:
ConvergenceWarning: Stochastic Optimizer: Maximum iterations (1000) reached and
the optimization hasn't converged yet.
  warnings.warn(
/projects/proteinml/.links/miniconda3/envs/aidep/lib/python3.10/site-
packages/sklearn/neural_network/_multilayer_perceptron.py:690:
ConvergenceWarning: Stochastic Optimizer: Maximum iterations (1000) reached and
the optimization hasn't converged yet.
  warnings.warn(
/projects/proteinml/.links/miniconda3/envs/aidep/lib/python3.10/site-
packages/sklearn/neural_network/_multilayer_perceptron.py:690:
ConvergenceWarning: Stochastic Optimizer: Maximum iterations (1000) reached and
the optimization hasn't converged yet.
  warnings.warn(
/projects/proteinml/.links/miniconda3/envs/aidep/lib/python3.10/site-
packages/sklearn/neural_network/_multilayer_perceptron.py:690:
ConvergenceWarning: Stochastic Optimizer: Maximum iterations (1000) reached and
the optimization hasn't converged yet.
  warnings.warn(
/projects/proteinml/.links/miniconda3/envs/aidep/lib/python3.10/site-
packages/sklearn/neural_network/_multilayer_perceptron.py:690:
ConvergenceWarning: Stochastic Optimizer: Maximum iterations (1000) reached and
the optimization hasn't converged yet.
  warnings.warn(
/projects/proteinml/.links/miniconda3/envs/aidep/lib/python3.10/site-
packages/sklearn/neural_network/_multilayer_perceptron.py:690:
ConvergenceWarning: Stochastic Optimizer: Maximum iterations (1000) reached and
the optimization hasn't converged yet.
```

```
  warnings.warn(
/projects/proteinml/.links/miniconda3/envs/aidep/lib/python3.10/site-
packages/sklearn/neural_network/_multilayer_perceptron.py:690:
ConvergenceWarning: Stochastic Optimizer: Maximum iterations (1000) reached and
the optimization hasn't converged yet.
  warnings.warn(
/projects/proteinml/.links/miniconda3/envs/aidep/lib/python3.10/site-
packages/sklearn/neural_network/_multilayer_perceptron.py:690:
ConvergenceWarning: Stochastic Optimizer: Maximum iterations (1000) reached and
the optimization hasn't converged yet.
  warnings.warn(
/projects/proteinml/.links/miniconda3/envs/aidep/lib/python3.10/site-
packages/sklearn/neural_network/_multilayer_perceptron.py:690:
ConvergenceWarning: Stochastic Optimizer: Maximum iterations (1000) reached and
the optimization hasn't converged yet.
  warnings.warn(
/projects/proteinml/.links/miniconda3/envs/aidep/lib/python3.10/site-
packages/sklearn/neural_network/_multilayer_perceptron.py:690:
ConvergenceWarning: Stochastic Optimizer: Maximum iterations (1000) reached and
the optimization hasn't converged yet.
  warnings.warn(
/projects/proteinml/.links/miniconda3/envs/aidep/lib/python3.10/site-
packages/sklearn/neural_network/_multilayer_perceptron.py:690:
ConvergenceWarning: Stochastic Optimizer: Maximum iterations (1000) reached and
the optimization hasn't converged yet.
  warnings.warn(
/projects/proteinml/.links/miniconda3/envs/aidep/lib/python3.10/site-
packages/sklearn/neural_network/_multilayer_perceptron.py:690:
ConvergenceWarning: Stochastic Optimizer: Maximum iterations (1000) reached and
the optimization hasn't converged yet.
  warnings.warn(
/projects/proteinml/.links/miniconda3/envs/aidep/lib/python3.10/site-
packages/sklearn/neural_network/_multilayer_perceptron.py:690:
ConvergenceWarning: Stochastic Optimizer: Maximum iterations (1000) reached and
the optimization hasn't converged yet.
  warnings.warn(
/projects/proteinml/.links/miniconda3/envs/aidep/lib/python3.10/site-
packages/sklearn/neural_network/_multilayer_perceptron.py:690:
ConvergenceWarning: Stochastic Optimizer: Maximum iterations (1000) reached and
the optimization hasn't converged yet.
  warnings.warn(
/projects/proteinml/.links/miniconda3/envs/aidep/lib/python3.10/site-
packages/sklearn/neural_network/_multilayer_perceptron.py:690:
ConvergenceWarning: Stochastic Optimizer: Maximum iterations (1000) reached and
the optimization hasn't converged yet.
  warnings.warn(
/projects/proteinml/.links/miniconda3/envs/aidep/lib/python3.10/site-
packages/sklearn/neural_network/_multilayer_perceptron.py:690:
```

```
ConvergenceWarning: Stochastic Optimizer: Maximum iterations (1000) reached and
the optimization hasn't converged yet.
  warnings.warn(
/projects/proteinml/.links/miniconda3/envs/aidep/lib/python3.10/site-
packages/sklearn/neural_network/_multilayer_perceptron.py:690:
ConvergenceWarning: Stochastic Optimizer: Maximum iterations (1000) reached and
the optimization hasn't converged yet.
  warnings.warn(
/projects/proteinml/.links/miniconda3/envs/aidep/lib/python3.10/site-
packages/sklearn/neural_network/_multilayer_perceptron.py:690:
ConvergenceWarning: Stochastic Optimizer: Maximum iterations (1000) reached and
the optimization hasn't converged yet.
  warnings.warn(
/projects/proteinml/.links/miniconda3/envs/aidep/lib/python3.10/site-
packages/sklearn/neural_network/_multilayer_perceptron.py:690:
ConvergenceWarning: Stochastic Optimizer: Maximum iterations (1000) reached and
the optimization hasn't converged yet.
  warnings.warn(
/projects/proteinml/.links/miniconda3/envs/aidep/lib/python3.10/site-
packages/sklearn/neural_network/_multilayer_perceptron.py:690:
ConvergenceWarning: Stochastic Optimizer: Maximum iterations (1000) reached and
the optimization hasn't converged yet.
  warnings.warn(
/projects/proteinml/.links/miniconda3/envs/aidep/lib/python3.10/site-
packages/sklearn/neural_network/_multilayer_perceptron.py:690:
ConvergenceWarning: Stochastic Optimizer: Maximum iterations (1000) reached and
the optimization hasn't converged yet.
  warnings.warn(
/projects/proteinml/.links/miniconda3/envs/aidep/lib/python3.10/site-
packages/sklearn/neural_network/_multilayer_perceptron.py:690:
ConvergenceWarning: Stochastic Optimizer: Maximum iterations (1000) reached and
the optimization hasn't converged yet.
  warnings.warn(
/projects/proteinml/.links/miniconda3/envs/aidep/lib/python3.10/site-
packages/sklearn/neural_network/_multilayer_perceptron.py:690:
ConvergenceWarning: Stochastic Optimizer: Maximum iterations (1000) reached and
the optimization hasn't converged yet.
  warnings.warn(
/projects/proteinml/.links/miniconda3/envs/aidep/lib/python3.10/site-
packages/sklearn/neural_network/_multilayer_perceptron.py:690:
ConvergenceWarning: Stochastic Optimizer: Maximum iterations (1000) reached and
the optimization hasn't converged yet.
  warnings.warn(
/projects/proteinml/.links/miniconda3/envs/aidep/lib/python3.10/site-
packages/sklearn/neural_network/_multilayer_perceptron.py:690:
ConvergenceWarning: Stochastic Optimizer: Maximum iterations (1000) reached and
the optimization hasn't converged yet.
  warnings.warn(
```

```
/projects/proteinml/.links/miniconda3/envs/aidep/lib/python3.10/site-
packages/sklearn/neural_network/_multilayer_perceptron.py:690:
ConvergenceWarning: Stochastic Optimizer: Maximum iterations (1000) reached and
the optimization hasn't converged yet.
  warnings.warn(
/projects/proteinml/.links/miniconda3/envs/aidep/lib/python3.10/site-
packages/sklearn/neural_network/_multilayer_perceptron.py:690:
ConvergenceWarning: Stochastic Optimizer: Maximum iterations (1000) reached and
the optimization hasn't converged yet.
  warnings.warn(
/projects/proteinml/.links/miniconda3/envs/aidep/lib/python3.10/site-
packages/sklearn/neural_network/_multilayer_perceptron.py:690:
ConvergenceWarning: Stochastic Optimizer: Maximum iterations (1000) reached and
the optimization hasn't converged yet.
  warnings.warn(
/projects/proteinml/.links/miniconda3/envs/aidep/lib/python3.10/site-
packages/sklearn/neural_network/_multilayer_perceptron.py:690:
ConvergenceWarning: Stochastic Optimizer: Maximum iterations (1000) reached and
the optimization hasn't converged yet.
  warnings.warn(
/projects/proteinml/.links/miniconda3/envs/aidep/lib/python3.10/site-
packages/sklearn/neural_network/_multilayer_perceptron.py:690:
ConvergenceWarning: Stochastic Optimizer: Maximum iterations (1000) reached and
the optimization hasn't converged yet.
  warnings.warn(
/projects/proteinml/.links/miniconda3/envs/aidep/lib/python3.10/site-
packages/sklearn/neural_network/_multilayer_perceptron.py:690:
ConvergenceWarning: Stochastic Optimizer: Maximum iterations (1000) reached and
the optimization hasn't converged yet.
  warnings.warn(
/projects/proteinml/.links/miniconda3/envs/aidep/lib/python3.10/site-
packages/sklearn/neural_network/_multilayer_perceptron.py:690:
ConvergenceWarning: Stochastic Optimizer: Maximum iterations (1000) reached and
the optimization hasn't converged yet.
  warnings.warn(
/projects/proteinml/.links/miniconda3/envs/aidep/lib/python3.10/site-
packages/sklearn/neural_network/_multilayer_perceptron.py:690:
ConvergenceWarning: Stochastic Optimizer: Maximum iterations (1000) reached and
the optimization hasn't converged yet.
  warnings.warn(
/projects/proteinml/.links/miniconda3/envs/aidep/lib/python3.10/site-
packages/sklearn/neural_network/_multilayer_perceptron.py:690:
ConvergenceWarning: Stochastic Optimizer: Maximum iterations (1000) reached and
the optimization hasn't converged yet.
  warnings.warn(
/projects/proteinml/.links/miniconda3/envs/aidep/lib/python3.10/site-
packages/sklearn/neural_network/_multilayer_perceptron.py:690:
ConvergenceWarning: Stochastic Optimizer: Maximum iterations (1000) reached and
```

```
the optimization hasn't converged yet.
  warnings.warn(
/projects/proteinml/.links/miniconda3/envs/aidep/lib/python3.10/site-
packages/sklearn/neural_network/_multilayer_perceptron.py:690:
ConvergenceWarning: Stochastic Optimizer: Maximum iterations (1000) reached and
the optimization hasn't converged yet.
  warnings.warn(
/projects/proteinml/.links/miniconda3/envs/aidep/lib/python3.10/site-
packages/sklearn/neural_network/_multilayer_perceptron.py:690:
ConvergenceWarning: Stochastic Optimizer: Maximum iterations (1000) reached and
the optimization hasn't converged yet.
  warnings.warn(
/projects/proteinml/.links/miniconda3/envs/aidep/lib/python3.10/site-
packages/sklearn/neural_network/_multilayer_perceptron.py:690:
ConvergenceWarning: Stochastic Optimizer: Maximum iterations (1000) reached and
the optimization hasn't converged yet.
  warnings.warn(
/projects/proteinml/.links/miniconda3/envs/aidep/lib/python3.10/site-
packages/sklearn/neural_network/_multilayer_perceptron.py:690:
ConvergenceWarning: Stochastic Optimizer: Maximum iterations (1000) reached and
the optimization hasn't converged yet.
  warnings.warn(
/projects/proteinml/.links/miniconda3/envs/aidep/lib/python3.10/site-
packages/sklearn/neural_network/_multilayer_perceptron.py:690:
ConvergenceWarning: Stochastic Optimizer: Maximum iterations (1000) reached and
the optimization hasn't converged yet.
  warnings.warn(
/projects/proteinml/.links/miniconda3/envs/aidep/lib/python3.10/site-
packages/sklearn/neural_network/_multilayer_perceptron.py:690:
ConvergenceWarning: Stochastic Optimizer: Maximum iterations (1000) reached and
the optimization hasn't converged yet.
  warnings.warn(
/projects/proteinml/.links/miniconda3/envs/aidep/lib/python3.10/site-
packages/sklearn/neural_network/_multilayer_perceptron.py:690:
ConvergenceWarning: Stochastic Optimizer: Maximum iterations (1000) reached and
the optimization hasn't converged yet.
  warnings.warn(
/projects/proteinml/.links/miniconda3/envs/aidep/lib/python3.10/site-
packages/sklearn/neural_network/_multilayer_perceptron.py:690:
ConvergenceWarning: Stochastic Optimizer: Maximum iterations (1000) reached and
the optimization hasn't converged yet.
  warnings.warn(
/projects/proteinml/.links/miniconda3/envs/aidep/lib/python3.10/site-
packages/sklearn/neural_network/_multilayer_perceptron.py:690:
ConvergenceWarning: Stochastic Optimizer: Maximum iterations (1000) reached and
the optimization hasn't converged yet.
  warnings.warn(
/projects/proteinml/.links/miniconda3/envs/aidep/lib/python3.10/site-
```

```
packages/sklearn/neural_network/_multilayer_perceptron.py:690:
ConvergenceWarning: Stochastic Optimizer: Maximum iterations (1000) reached and
the optimization hasn't converged yet.
  warnings.warn(
/projects/proteinml/.links/miniconda3/envs/aidep/lib/python3.10/site-
packages/sklearn/neural_network/_multilayer_perceptron.py:690:
ConvergenceWarning: Stochastic Optimizer: Maximum iterations (1000) reached and
the optimization hasn't converged yet.
  warnings.warn(
/projects/proteinml/.links/miniconda3/envs/aidep/lib/python3.10/site-
packages/sklearn/neural_network/_multilayer_perceptron.py:690:
ConvergenceWarning: Stochastic Optimizer: Maximum iterations (1000) reached and
the optimization hasn't converged yet.
  warnings.warn(
/projects/proteinml/.links/miniconda3/envs/aidep/lib/python3.10/site-
packages/sklearn/neural_network/_multilayer_perceptron.py:690:
ConvergenceWarning: Stochastic Optimizer: Maximum iterations (1000) reached and
the optimization hasn't converged yet.
  warnings.warn(
/projects/proteinml/.links/miniconda3/envs/aidep/lib/python3.10/site-
packages/sklearn/neural_network/_multilayer_perceptron.py:690:
ConvergenceWarning: Stochastic Optimizer: Maximum iterations (1000) reached and
the optimization hasn't converged yet.
  warnings.warn(
/projects/proteinml/.links/miniconda3/envs/aidep/lib/python3.10/site-
packages/sklearn/neural_network/_multilayer_perceptron.py:690:
ConvergenceWarning: Stochastic Optimizer: Maximum iterations (1000) reached and
the optimization hasn't converged yet.
  warnings.warn(
/projects/proteinml/.links/miniconda3/envs/aidep/lib/python3.10/site-
packages/sklearn/neural_network/_multilayer_perceptron.py:690:
ConvergenceWarning: Stochastic Optimizer: Maximum iterations (1000) reached and
the optimization hasn't converged yet.
  warnings.warn(
/projects/proteinml/.links/miniconda3/envs/aidep/lib/python3.10/site-
packages/sklearn/neural_network/_multilayer_perceptron.py:690:
ConvergenceWarning: Stochastic Optimizer: Maximum iterations (1000) reached and
the optimization hasn't converged yet.
  warnings.warn(
/projects/proteinml/.links/miniconda3/envs/aidep/lib/python3.10/site-
packages/sklearn/neural_network/_multilayer_perceptron.py:690:
ConvergenceWarning: Stochastic Optimizer: Maximum iterations (1000) reached and
the optimization hasn't converged yet.
  warnings.warn(
/projects/proteinml/.links/miniconda3/envs/aidep/lib/python3.10/site-
packages/sklearn/neural_network/_multilayer_perceptron.py:690:
ConvergenceWarning: Stochastic Optimizer: Maximum iterations (1000) reached and
the optimization hasn't converged yet.
```

```
  warnings.warn(
/projects/proteinml/.links/miniconda3/envs/aidep/lib/python3.10/site-
packages/sklearn/neural_network/_multilayer_perceptron.py:690:
ConvergenceWarning: Stochastic Optimizer: Maximum iterations (1000) reached and
the optimization hasn't converged yet.
  warnings.warn(
/projects/proteinml/.links/miniconda3/envs/aidep/lib/python3.10/site-
packages/sklearn/neural_network/_multilayer_perceptron.py:690:
ConvergenceWarning: Stochastic Optimizer: Maximum iterations (1000) reached and
the optimization hasn't converged yet.
  warnings.warn(
/projects/proteinml/.links/miniconda3/envs/aidep/lib/python3.10/site-
packages/sklearn/neural_network/_multilayer_perceptron.py:690:
ConvergenceWarning: Stochastic Optimizer: Maximum iterations (1000) reached and
the optimization hasn't converged yet.
  warnings.warn(
/projects/proteinml/.links/miniconda3/envs/aidep/lib/python3.10/site-
packages/sklearn/neural_network/_multilayer_perceptron.py:690:
ConvergenceWarning: Stochastic Optimizer: Maximum iterations (1000) reached and
the optimization hasn't converged yet.
  warnings.warn(
/projects/proteinml/.links/miniconda3/envs/aidep/lib/python3.10/site-
packages/sklearn/neural_network/_multilayer_perceptron.py:690:
ConvergenceWarning: Stochastic Optimizer: Maximum iterations (1000) reached and
the optimization hasn't converged yet.
  warnings.warn(
/projects/proteinml/.links/miniconda3/envs/aidep/lib/python3.10/site-
packages/sklearn/neural_network/_multilayer_perceptron.py:690:
ConvergenceWarning: Stochastic Optimizer: Maximum iterations (1000) reached and
the optimization hasn't converged yet.
  warnings.warn(
/projects/proteinml/.links/miniconda3/envs/aidep/lib/python3.10/site-
packages/sklearn/neural_network/_multilayer_perceptron.py:690:
ConvergenceWarning: Stochastic Optimizer: Maximum iterations (1000) reached and
the optimization hasn't converged yet.
  warnings.warn(
/projects/proteinml/.links/miniconda3/envs/aidep/lib/python3.10/site-
packages/sklearn/neural_network/_multilayer_perceptron.py:690:
ConvergenceWarning: Stochastic Optimizer: Maximum iterations (1000) reached and
the optimization hasn't converged yet.
  warnings.warn(
/projects/proteinml/.links/miniconda3/envs/aidep/lib/python3.10/site-
packages/sklearn/neural_network/_multilayer_perceptron.py:690:
ConvergenceWarning: Stochastic Optimizer: Maximum iterations (1000) reached and
the optimization hasn't converged yet.
  warnings.warn(
/projects/proteinml/.links/miniconda3/envs/aidep/lib/python3.10/site-
packages/sklearn/neural_network/_multilayer_perceptron.py:690:
```

```
ConvergenceWarning: Stochastic Optimizer: Maximum iterations (1000) reached and
the optimization hasn't converged yet.
  warnings.warn(
/projects/proteinml/.links/miniconda3/envs/aidep/lib/python3.10/site-
packages/sklearn/neural_network/_multilayer_perceptron.py:690:
ConvergenceWarning: Stochastic Optimizer: Maximum iterations (1000) reached and
the optimization hasn't converged yet.
  warnings.warn(
/projects/proteinml/.links/miniconda3/envs/aidep/lib/python3.10/site-
packages/sklearn/neural_network/_multilayer_perceptron.py:690:
ConvergenceWarning: Stochastic Optimizer: Maximum iterations (1000) reached and
the optimization hasn't converged yet.
  warnings.warn(
/projects/proteinml/.links/miniconda3/envs/aidep/lib/python3.10/site-
packages/sklearn/neural_network/_multilayer_perceptron.py:690:
ConvergenceWarning: Stochastic Optimizer: Maximum iterations (1000) reached and
the optimization hasn't converged yet.
  warnings.warn(
/projects/proteinml/.links/miniconda3/envs/aidep/lib/python3.10/site-
packages/sklearn/neural_network/_multilayer_perceptron.py:690:
ConvergenceWarning: Stochastic Optimizer: Maximum iterations (1000) reached and
the optimization hasn't converged yet.
  warnings.warn(
/projects/proteinml/.links/miniconda3/envs/aidep/lib/python3.10/site-
packages/sklearn/neural_network/_multilayer_perceptron.py:690:
ConvergenceWarning: Stochastic Optimizer: Maximum iterations (1000) reached and
the optimization hasn't converged yet.
  warnings.warn(
/projects/proteinml/.links/miniconda3/envs/aidep/lib/python3.10/site-
packages/sklearn/neural_network/_multilayer_perceptron.py:690:
ConvergenceWarning: Stochastic Optimizer: Maximum iterations (1000) reached and
the optimization hasn't converged yet.
  warnings.warn(
/projects/proteinml/.links/miniconda3/envs/aidep/lib/python3.10/site-
packages/sklearn/neural_network/_multilayer_perceptron.py:690:
ConvergenceWarning: Stochastic Optimizer: Maximum iterations (1000) reached and
the optimization hasn't converged yet.
  warnings.warn(
/projects/proteinml/.links/miniconda3/envs/aidep/lib/python3.10/site-
packages/sklearn/neural_network/_multilayer_perceptron.py:690:
ConvergenceWarning: Stochastic Optimizer: Maximum iterations (1000) reached and
the optimization hasn't converged yet.
  warnings.warn(
/projects/proteinml/.links/miniconda3/envs/aidep/lib/python3.10/site-
packages/sklearn/neural_network/_multilayer_perceptron.py:690:
ConvergenceWarning: Stochastic Optimizer: Maximum iterations (1000) reached and
the optimization hasn't converged yet.
  warnings.warn(
```

```
/projects/proteinml/.links/miniconda3/envs/aidep/lib/python3.10/site-
packages/sklearn/neural_network/_multilayer_perceptron.py:690:
ConvergenceWarning: Stochastic Optimizer: Maximum iterations (1000) reached and
the optimization hasn't converged yet.
  warnings.warn(
/projects/proteinml/.links/miniconda3/envs/aidep/lib/python3.10/site-
packages/sklearn/neural_network/_multilayer_perceptron.py:690:
ConvergenceWarning: Stochastic Optimizer: Maximum iterations (1000) reached and
the optimization hasn't converged yet.
  warnings.warn(
/projects/proteinml/.links/miniconda3/envs/aidep/lib/python3.10/site-
packages/sklearn/neural_network/_multilayer_perceptron.py:690:
ConvergenceWarning: Stochastic Optimizer: Maximum iterations (1000) reached and
the optimization hasn't converged yet.
  warnings.warn(
/projects/proteinml/.links/miniconda3/envs/aidep/lib/python3.10/site-
packages/sklearn/neural_network/_multilayer_perceptron.py:690:
ConvergenceWarning: Stochastic Optimizer: Maximum iterations (1000) reached and
the optimization hasn't converged yet.
  warnings.warn(
/projects/proteinml/.links/miniconda3/envs/aidep/lib/python3.10/site-
packages/sklearn/neural_network/_multilayer_perceptron.py:690:
ConvergenceWarning: Stochastic Optimizer: Maximum iterations (1000) reached and
the optimization hasn't converged yet.
  warnings.warn(
```

[30]: ['evc_esmfull_mlp_scores.joblib']

1.6.5 4.5 EVC plus ESM2 only 4 linear

[31]:
```
X_ = np.concatenate([X_esm_only4, X_evc], axis=1)
```

[32]:
```
evc_esm4sites_linear_scores = hyperopt_and_scoring(linear_pca, X_, y,␣
↪linear_param_space)
joblib.dump(evc_esm4sites_linear_scores, 'evc_esm4sites_linear_scores.joblib')
```

[32]: ['evc_esm4sites_linear_scores.joblib']

1.6.6 4.6 EVC plus ESM2 only 4 MLP

[33]:
```
evc_esm4sites_mlp_scores = hyperopt_and_scoring(mlp_pca, X_, y, mlp_param_space)
joblib.dump(evc_esm4sites_mlp_scores, 'evc_esm4sites_mlp_scores.joblib')
```

```
/projects/proteinml/.links/miniconda3/envs/aidep/lib/python3.10/site-
packages/sklearn/neural_network/_multilayer_perceptron.py:690:
ConvergenceWarning: Stochastic Optimizer: Maximum iterations (1000) reached and
the optimization hasn't converged yet.
```

```
  warnings.warn(
/projects/proteinml/.links/miniconda3/envs/aidep/lib/python3.10/site-
packages/sklearn/neural_network/_multilayer_perceptron.py:690:
ConvergenceWarning: Stochastic Optimizer: Maximum iterations (1000) reached and
the optimization hasn't converged yet.
  warnings.warn(
/projects/proteinml/.links/miniconda3/envs/aidep/lib/python3.10/site-
packages/sklearn/neural_network/_multilayer_perceptron.py:690:
ConvergenceWarning: Stochastic Optimizer: Maximum iterations (1000) reached and
the optimization hasn't converged yet.
  warnings.warn(
/projects/proteinml/.links/miniconda3/envs/aidep/lib/python3.10/site-
packages/sklearn/neural_network/_multilayer_perceptron.py:690:
ConvergenceWarning: Stochastic Optimizer: Maximum iterations (1000) reached and
the optimization hasn't converged yet.
  warnings.warn(
/projects/proteinml/.links/miniconda3/envs/aidep/lib/python3.10/site-
packages/sklearn/neural_network/_multilayer_perceptron.py:690:
ConvergenceWarning: Stochastic Optimizer: Maximum iterations (1000) reached and
the optimization hasn't converged yet.
  warnings.warn(
/projects/proteinml/.links/miniconda3/envs/aidep/lib/python3.10/site-
packages/sklearn/neural_network/_multilayer_perceptron.py:690:
ConvergenceWarning: Stochastic Optimizer: Maximum iterations (1000) reached and
the optimization hasn't converged yet.
  warnings.warn(
/projects/proteinml/.links/miniconda3/envs/aidep/lib/python3.10/site-
packages/sklearn/neural_network/_multilayer_perceptron.py:690:
ConvergenceWarning: Stochastic Optimizer: Maximum iterations (1000) reached and
the optimization hasn't converged yet.
  warnings.warn(
/projects/proteinml/.links/miniconda3/envs/aidep/lib/python3.10/site-
packages/sklearn/neural_network/_multilayer_perceptron.py:690:
ConvergenceWarning: Stochastic Optimizer: Maximum iterations (1000) reached and
the optimization hasn't converged yet.
  warnings.warn(
/projects/proteinml/.links/miniconda3/envs/aidep/lib/python3.10/site-
packages/sklearn/neural_network/_multilayer_perceptron.py:690:
ConvergenceWarning: Stochastic Optimizer: Maximum iterations (1000) reached and
the optimization hasn't converged yet.
  warnings.warn(
/projects/proteinml/.links/miniconda3/envs/aidep/lib/python3.10/site-
packages/sklearn/neural_network/_multilayer_perceptron.py:690:
ConvergenceWarning: Stochastic Optimizer: Maximum iterations (1000) reached and
the optimization hasn't converged yet.
  warnings.warn(
/projects/proteinml/.links/miniconda3/envs/aidep/lib/python3.10/site-
packages/sklearn/neural_network/_multilayer_perceptron.py:690:
```

```
ConvergenceWarning: Stochastic Optimizer: Maximum iterations (1000) reached and
the optimization hasn't converged yet.
  warnings.warn(
/projects/proteinml/.links/miniconda3/envs/aidep/lib/python3.10/site-
packages/sklearn/neural_network/_multilayer_perceptron.py:690:
ConvergenceWarning: Stochastic Optimizer: Maximum iterations (1000) reached and
the optimization hasn't converged yet.
  warnings.warn(
/projects/proteinml/.links/miniconda3/envs/aidep/lib/python3.10/site-
packages/sklearn/neural_network/_multilayer_perceptron.py:690:
ConvergenceWarning: Stochastic Optimizer: Maximum iterations (1000) reached and
the optimization hasn't converged yet.
  warnings.warn(
/projects/proteinml/.links/miniconda3/envs/aidep/lib/python3.10/site-
packages/sklearn/neural_network/_multilayer_perceptron.py:690:
ConvergenceWarning: Stochastic Optimizer: Maximum iterations (1000) reached and
the optimization hasn't converged yet.
  warnings.warn(
/projects/proteinml/.links/miniconda3/envs/aidep/lib/python3.10/site-
packages/sklearn/neural_network/_multilayer_perceptron.py:690:
ConvergenceWarning: Stochastic Optimizer: Maximum iterations (1000) reached and
the optimization hasn't converged yet.
  warnings.warn(
/projects/proteinml/.links/miniconda3/envs/aidep/lib/python3.10/site-
packages/sklearn/neural_network/_multilayer_perceptron.py:690:
ConvergenceWarning: Stochastic Optimizer: Maximum iterations (1000) reached and
the optimization hasn't converged yet.
  warnings.warn(
/projects/proteinml/.links/miniconda3/envs/aidep/lib/python3.10/site-
packages/sklearn/neural_network/_multilayer_perceptron.py:690:
ConvergenceWarning: Stochastic Optimizer: Maximum iterations (1000) reached and
the optimization hasn't converged yet.
  warnings.warn(
/projects/proteinml/.links/miniconda3/envs/aidep/lib/python3.10/site-
packages/sklearn/neural_network/_multilayer_perceptron.py:690:
ConvergenceWarning: Stochastic Optimizer: Maximum iterations (1000) reached and
the optimization hasn't converged yet.
  warnings.warn(
/projects/proteinml/.links/miniconda3/envs/aidep/lib/python3.10/site-
packages/sklearn/neural_network/_multilayer_perceptron.py:690:
ConvergenceWarning: Stochastic Optimizer: Maximum iterations (1000) reached and
the optimization hasn't converged yet.
  warnings.warn(
/projects/proteinml/.links/miniconda3/envs/aidep/lib/python3.10/site-
packages/sklearn/neural_network/_multilayer_perceptron.py:690:
ConvergenceWarning: Stochastic Optimizer: Maximum iterations (1000) reached and
the optimization hasn't converged yet.
  warnings.warn(
```

```
/projects/proteinml/.links/miniconda3/envs/aidep/lib/python3.10/site-
packages/sklearn/neural_network/_multilayer_perceptron.py:690:
ConvergenceWarning: Stochastic Optimizer: Maximum iterations (1000) reached and
the optimization hasn't converged yet.
  warnings.warn(
/projects/proteinml/.links/miniconda3/envs/aidep/lib/python3.10/site-
packages/sklearn/neural_network/_multilayer_perceptron.py:690:
ConvergenceWarning: Stochastic Optimizer: Maximum iterations (1000) reached and
the optimization hasn't converged yet.
  warnings.warn(
/projects/proteinml/.links/miniconda3/envs/aidep/lib/python3.10/site-
packages/sklearn/neural_network/_multilayer_perceptron.py:690:
ConvergenceWarning: Stochastic Optimizer: Maximum iterations (1000) reached and
the optimization hasn't converged yet.
  warnings.warn(
/projects/proteinml/.links/miniconda3/envs/aidep/lib/python3.10/site-
packages/sklearn/neural_network/_multilayer_perceptron.py:690:
ConvergenceWarning: Stochastic Optimizer: Maximum iterations (1000) reached and
the optimization hasn't converged yet.
  warnings.warn(
/projects/proteinml/.links/miniconda3/envs/aidep/lib/python3.10/site-
packages/sklearn/neural_network/_multilayer_perceptron.py:690:
ConvergenceWarning: Stochastic Optimizer: Maximum iterations (1000) reached and
the optimization hasn't converged yet.
  warnings.warn(
/projects/proteinml/.links/miniconda3/envs/aidep/lib/python3.10/site-
packages/sklearn/neural_network/_multilayer_perceptron.py:690:
ConvergenceWarning: Stochastic Optimizer: Maximum iterations (1000) reached and
the optimization hasn't converged yet.
  warnings.warn(
/projects/proteinml/.links/miniconda3/envs/aidep/lib/python3.10/site-
packages/sklearn/neural_network/_multilayer_perceptron.py:690:
ConvergenceWarning: Stochastic Optimizer: Maximum iterations (1000) reached and
the optimization hasn't converged yet.
  warnings.warn(
/projects/proteinml/.links/miniconda3/envs/aidep/lib/python3.10/site-
packages/sklearn/neural_network/_multilayer_perceptron.py:690:
ConvergenceWarning: Stochastic Optimizer: Maximum iterations (1000) reached and
the optimization hasn't converged yet.
  warnings.warn(
/projects/proteinml/.links/miniconda3/envs/aidep/lib/python3.10/site-
packages/sklearn/neural_network/_multilayer_perceptron.py:690:
ConvergenceWarning: Stochastic Optimizer: Maximum iterations (1000) reached and
the optimization hasn't converged yet.
  warnings.warn(
/projects/proteinml/.links/miniconda3/envs/aidep/lib/python3.10/site-
packages/sklearn/neural_network/_multilayer_perceptron.py:690:
ConvergenceWarning: Stochastic Optimizer: Maximum iterations (1000) reached and
```

```
the optimization hasn't converged yet.
  warnings.warn(
/projects/proteinml/.links/miniconda3/envs/aidep/lib/python3.10/site-
packages/sklearn/neural_network/_multilayer_perceptron.py:690:
ConvergenceWarning: Stochastic Optimizer: Maximum iterations (1000) reached and
the optimization hasn't converged yet.
  warnings.warn(
/projects/proteinml/.links/miniconda3/envs/aidep/lib/python3.10/site-
packages/sklearn/neural_network/_multilayer_perceptron.py:690:
ConvergenceWarning: Stochastic Optimizer: Maximum iterations (1000) reached and
the optimization hasn't converged yet.
  warnings.warn(
/projects/proteinml/.links/miniconda3/envs/aidep/lib/python3.10/site-
packages/sklearn/neural_network/_multilayer_perceptron.py:690:
ConvergenceWarning: Stochastic Optimizer: Maximum iterations (1000) reached and
the optimization hasn't converged yet.
  warnings.warn(
/projects/proteinml/.links/miniconda3/envs/aidep/lib/python3.10/site-
packages/sklearn/neural_network/_multilayer_perceptron.py:690:
ConvergenceWarning: Stochastic Optimizer: Maximum iterations (1000) reached and
the optimization hasn't converged yet.
  warnings.warn(
/projects/proteinml/.links/miniconda3/envs/aidep/lib/python3.10/site-
packages/sklearn/neural_network/_multilayer_perceptron.py:690:
ConvergenceWarning: Stochastic Optimizer: Maximum iterations (1000) reached and
the optimization hasn't converged yet.
  warnings.warn(
/projects/proteinml/.links/miniconda3/envs/aidep/lib/python3.10/site-
packages/sklearn/neural_network/_multilayer_perceptron.py:690:
ConvergenceWarning: Stochastic Optimizer: Maximum iterations (1000) reached and
the optimization hasn't converged yet.
  warnings.warn(
/projects/proteinml/.links/miniconda3/envs/aidep/lib/python3.10/site-
packages/sklearn/neural_network/_multilayer_perceptron.py:690:
ConvergenceWarning: Stochastic Optimizer: Maximum iterations (1000) reached and
the optimization hasn't converged yet.
  warnings.warn(
/projects/proteinml/.links/miniconda3/envs/aidep/lib/python3.10/site-
packages/sklearn/neural_network/_multilayer_perceptron.py:690:
ConvergenceWarning: Stochastic Optimizer: Maximum iterations (1000) reached and
the optimization hasn't converged yet.
  warnings.warn(
/projects/proteinml/.links/miniconda3/envs/aidep/lib/python3.10/site-
packages/sklearn/neural_network/_multilayer_perceptron.py:690:
ConvergenceWarning: Stochastic Optimizer: Maximum iterations (1000) reached and
the optimization hasn't converged yet.
  warnings.warn(
/projects/proteinml/.links/miniconda3/envs/aidep/lib/python3.10/site-
```

```
packages/sklearn/neural_network/_multilayer_perceptron.py:690:
ConvergenceWarning: Stochastic Optimizer: Maximum iterations (1000) reached and
the optimization hasn't converged yet.
  warnings.warn(
/projects/proteinml/.links/miniconda3/envs/aidep/lib/python3.10/site-
packages/sklearn/neural_network/_multilayer_perceptron.py:690:
ConvergenceWarning: Stochastic Optimizer: Maximum iterations (1000) reached and
the optimization hasn't converged yet.
  warnings.warn(
/projects/proteinml/.links/miniconda3/envs/aidep/lib/python3.10/site-
packages/sklearn/neural_network/_multilayer_perceptron.py:690:
ConvergenceWarning: Stochastic Optimizer: Maximum iterations (1000) reached and
the optimization hasn't converged yet.
  warnings.warn(
/projects/proteinml/.links/miniconda3/envs/aidep/lib/python3.10/site-
packages/sklearn/neural_network/_multilayer_perceptron.py:690:
ConvergenceWarning: Stochastic Optimizer: Maximum iterations (1000) reached and
the optimization hasn't converged yet.
  warnings.warn(
/projects/proteinml/.links/miniconda3/envs/aidep/lib/python3.10/site-
packages/sklearn/neural_network/_multilayer_perceptron.py:690:
ConvergenceWarning: Stochastic Optimizer: Maximum iterations (1000) reached and
the optimization hasn't converged yet.
  warnings.warn(
/projects/proteinml/.links/miniconda3/envs/aidep/lib/python3.10/site-
packages/sklearn/neural_network/_multilayer_perceptron.py:690:
ConvergenceWarning: Stochastic Optimizer: Maximum iterations (1000) reached and
the optimization hasn't converged yet.
  warnings.warn(
/projects/proteinml/.links/miniconda3/envs/aidep/lib/python3.10/site-
packages/sklearn/neural_network/_multilayer_perceptron.py:690:
ConvergenceWarning: Stochastic Optimizer: Maximum iterations (1000) reached and
the optimization hasn't converged yet.
  warnings.warn(
/projects/proteinml/.links/miniconda3/envs/aidep/lib/python3.10/site-
packages/sklearn/neural_network/_multilayer_perceptron.py:690:
ConvergenceWarning: Stochastic Optimizer: Maximum iterations (1000) reached and
the optimization hasn't converged yet.
  warnings.warn(
/projects/proteinml/.links/miniconda3/envs/aidep/lib/python3.10/site-
packages/sklearn/neural_network/_multilayer_perceptron.py:690:
ConvergenceWarning: Stochastic Optimizer: Maximum iterations (1000) reached and
the optimization hasn't converged yet.
  warnings.warn(
/projects/proteinml/.links/miniconda3/envs/aidep/lib/python3.10/site-
packages/sklearn/neural_network/_multilayer_perceptron.py:690:
ConvergenceWarning: Stochastic Optimizer: Maximum iterations (1000) reached and
the optimization hasn't converged yet.
```

```
  warnings.warn(
/projects/proteinml/.links/miniconda3/envs/aidep/lib/python3.10/site-
packages/sklearn/neural_network/_multilayer_perceptron.py:690:
ConvergenceWarning: Stochastic Optimizer: Maximum iterations (1000) reached and
the optimization hasn't converged yet.
  warnings.warn(
/projects/proteinml/.links/miniconda3/envs/aidep/lib/python3.10/site-
packages/sklearn/neural_network/_multilayer_perceptron.py:690:
ConvergenceWarning: Stochastic Optimizer: Maximum iterations (1000) reached and
the optimization hasn't converged yet.
  warnings.warn(
/projects/proteinml/.links/miniconda3/envs/aidep/lib/python3.10/site-
packages/sklearn/neural_network/_multilayer_perceptron.py:690:
ConvergenceWarning: Stochastic Optimizer: Maximum iterations (1000) reached and
the optimization hasn't converged yet.
  warnings.warn(
/projects/proteinml/.links/miniconda3/envs/aidep/lib/python3.10/site-
packages/sklearn/neural_network/_multilayer_perceptron.py:690:
ConvergenceWarning: Stochastic Optimizer: Maximum iterations (1000) reached and
the optimization hasn't converged yet.
  warnings.warn(
/projects/proteinml/.links/miniconda3/envs/aidep/lib/python3.10/site-
packages/sklearn/neural_network/_multilayer_perceptron.py:690:
ConvergenceWarning: Stochastic Optimizer: Maximum iterations (1000) reached and
the optimization hasn't converged yet.
  warnings.warn(
/projects/proteinml/.links/miniconda3/envs/aidep/lib/python3.10/site-
packages/sklearn/neural_network/_multilayer_perceptron.py:690:
ConvergenceWarning: Stochastic Optimizer: Maximum iterations (1000) reached and
the optimization hasn't converged yet.
  warnings.warn(
/projects/proteinml/.links/miniconda3/envs/aidep/lib/python3.10/site-
packages/sklearn/neural_network/_multilayer_perceptron.py:690:
ConvergenceWarning: Stochastic Optimizer: Maximum iterations (1000) reached and
the optimization hasn't converged yet.
  warnings.warn(
/projects/proteinml/.links/miniconda3/envs/aidep/lib/python3.10/site-
packages/sklearn/neural_network/_multilayer_perceptron.py:690:
ConvergenceWarning: Stochastic Optimizer: Maximum iterations (1000) reached and
the optimization hasn't converged yet.
  warnings.warn(
/projects/proteinml/.links/miniconda3/envs/aidep/lib/python3.10/site-
packages/sklearn/neural_network/_multilayer_perceptron.py:690:
ConvergenceWarning: Stochastic Optimizer: Maximum iterations (1000) reached and
the optimization hasn't converged yet.
  warnings.warn(
/projects/proteinml/.links/miniconda3/envs/aidep/lib/python3.10/site-
packages/sklearn/neural_network/_multilayer_perceptron.py:690:
```

```
ConvergenceWarning: Stochastic Optimizer: Maximum iterations (1000) reached and
the optimization hasn't converged yet.
  warnings.warn(
/projects/proteinml/.links/miniconda3/envs/aidep/lib/python3.10/site-
packages/sklearn/neural_network/_multilayer_perceptron.py:690:
ConvergenceWarning: Stochastic Optimizer: Maximum iterations (1000) reached and
the optimization hasn't converged yet.
  warnings.warn(
/projects/proteinml/.links/miniconda3/envs/aidep/lib/python3.10/site-
packages/sklearn/neural_network/_multilayer_perceptron.py:690:
ConvergenceWarning: Stochastic Optimizer: Maximum iterations (1000) reached and
the optimization hasn't converged yet.
  warnings.warn(
/projects/proteinml/.links/miniconda3/envs/aidep/lib/python3.10/site-
packages/sklearn/neural_network/_multilayer_perceptron.py:690:
ConvergenceWarning: Stochastic Optimizer: Maximum iterations (1000) reached and
the optimization hasn't converged yet.
  warnings.warn(
/projects/proteinml/.links/miniconda3/envs/aidep/lib/python3.10/site-
packages/sklearn/neural_network/_multilayer_perceptron.py:690:
ConvergenceWarning: Stochastic Optimizer: Maximum iterations (1000) reached and
the optimization hasn't converged yet.
  warnings.warn(
/projects/proteinml/.links/miniconda3/envs/aidep/lib/python3.10/site-
packages/sklearn/neural_network/_multilayer_perceptron.py:690:
ConvergenceWarning: Stochastic Optimizer: Maximum iterations (1000) reached and
the optimization hasn't converged yet.
  warnings.warn(
/projects/proteinml/.links/miniconda3/envs/aidep/lib/python3.10/site-
packages/sklearn/neural_network/_multilayer_perceptron.py:690:
ConvergenceWarning: Stochastic Optimizer: Maximum iterations (1000) reached and
the optimization hasn't converged yet.
  warnings.warn(
/projects/proteinml/.links/miniconda3/envs/aidep/lib/python3.10/site-
packages/sklearn/neural_network/_multilayer_perceptron.py:690:
ConvergenceWarning: Stochastic Optimizer: Maximum iterations (1000) reached and
the optimization hasn't converged yet.
  warnings.warn(
/projects/proteinml/.links/miniconda3/envs/aidep/lib/python3.10/site-
packages/sklearn/neural_network/_multilayer_perceptron.py:690:
ConvergenceWarning: Stochastic Optimizer: Maximum iterations (1000) reached and
the optimization hasn't converged yet.
  warnings.warn(
/projects/proteinml/.links/miniconda3/envs/aidep/lib/python3.10/site-
packages/sklearn/neural_network/_multilayer_perceptron.py:690:
ConvergenceWarning: Stochastic Optimizer: Maximum iterations (1000) reached and
the optimization hasn't converged yet.
  warnings.warn(
```

```
/projects/proteinml/.links/miniconda3/envs/aidep/lib/python3.10/site-
packages/sklearn/neural_network/_multilayer_perceptron.py:690:
ConvergenceWarning: Stochastic Optimizer: Maximum iterations (1000) reached and
the optimization hasn't converged yet.
  warnings.warn(
/projects/proteinml/.links/miniconda3/envs/aidep/lib/python3.10/site-
packages/sklearn/neural_network/_multilayer_perceptron.py:690:
ConvergenceWarning: Stochastic Optimizer: Maximum iterations (1000) reached and
the optimization hasn't converged yet.
  warnings.warn(
/projects/proteinml/.links/miniconda3/envs/aidep/lib/python3.10/site-
packages/sklearn/neural_network/_multilayer_perceptron.py:690:
ConvergenceWarning: Stochastic Optimizer: Maximum iterations (1000) reached and
the optimization hasn't converged yet.
  warnings.warn(
/projects/proteinml/.links/miniconda3/envs/aidep/lib/python3.10/site-
packages/sklearn/neural_network/_multilayer_perceptron.py:690:
ConvergenceWarning: Stochastic Optimizer: Maximum iterations (1000) reached and
the optimization hasn't converged yet.
  warnings.warn(
/projects/proteinml/.links/miniconda3/envs/aidep/lib/python3.10/site-
packages/sklearn/neural_network/_multilayer_perceptron.py:690:
ConvergenceWarning: Stochastic Optimizer: Maximum iterations (1000) reached and
the optimization hasn't converged yet.
  warnings.warn(
/projects/proteinml/.links/miniconda3/envs/aidep/lib/python3.10/site-
packages/sklearn/neural_network/_multilayer_perceptron.py:690:
ConvergenceWarning: Stochastic Optimizer: Maximum iterations (1000) reached and
the optimization hasn't converged yet.
  warnings.warn(
/projects/proteinml/.links/miniconda3/envs/aidep/lib/python3.10/site-
packages/sklearn/neural_network/_multilayer_perceptron.py:690:
ConvergenceWarning: Stochastic Optimizer: Maximum iterations (1000) reached and
the optimization hasn't converged yet.
  warnings.warn(
/projects/proteinml/.links/miniconda3/envs/aidep/lib/python3.10/site-
packages/sklearn/neural_network/_multilayer_perceptron.py:690:
ConvergenceWarning: Stochastic Optimizer: Maximum iterations (1000) reached and
the optimization hasn't converged yet.
  warnings.warn(
/projects/proteinml/.links/miniconda3/envs/aidep/lib/python3.10/site-
packages/sklearn/neural_network/_multilayer_perceptron.py:690:
ConvergenceWarning: Stochastic Optimizer: Maximum iterations (1000) reached and
the optimization hasn't converged yet.
  warnings.warn(
/projects/proteinml/.links/miniconda3/envs/aidep/lib/python3.10/site-
packages/sklearn/neural_network/_multilayer_perceptron.py:690:
ConvergenceWarning: Stochastic Optimizer: Maximum iterations (1000) reached and
```

```
the optimization hasn't converged yet.
  warnings.warn(
/projects/proteinml/.links/miniconda3/envs/aidep/lib/python3.10/site-
packages/sklearn/neural_network/_multilayer_perceptron.py:690:
ConvergenceWarning: Stochastic Optimizer: Maximum iterations (1000) reached and
the optimization hasn't converged yet.
  warnings.warn(
/projects/proteinml/.links/miniconda3/envs/aidep/lib/python3.10/site-
packages/sklearn/neural_network/_multilayer_perceptron.py:690:
ConvergenceWarning: Stochastic Optimizer: Maximum iterations (1000) reached and
the optimization hasn't converged yet.
  warnings.warn(
/projects/proteinml/.links/miniconda3/envs/aidep/lib/python3.10/site-
packages/sklearn/neural_network/_multilayer_perceptron.py:690:
ConvergenceWarning: Stochastic Optimizer: Maximum iterations (1000) reached and
the optimization hasn't converged yet.
  warnings.warn(
/projects/proteinml/.links/miniconda3/envs/aidep/lib/python3.10/site-
packages/sklearn/neural_network/_multilayer_perceptron.py:690:
ConvergenceWarning: Stochastic Optimizer: Maximum iterations (1000) reached and
the optimization hasn't converged yet.
  warnings.warn(
/projects/proteinml/.links/miniconda3/envs/aidep/lib/python3.10/site-
packages/sklearn/neural_network/_multilayer_perceptron.py:690:
ConvergenceWarning: Stochastic Optimizer: Maximum iterations (1000) reached and
the optimization hasn't converged yet.
  warnings.warn(
/projects/proteinml/.links/miniconda3/envs/aidep/lib/python3.10/site-
packages/sklearn/neural_network/_multilayer_perceptron.py:690:
ConvergenceWarning: Stochastic Optimizer: Maximum iterations (1000) reached and
the optimization hasn't converged yet.
  warnings.warn(
/projects/proteinml/.links/miniconda3/envs/aidep/lib/python3.10/site-
packages/sklearn/neural_network/_multilayer_perceptron.py:690:
ConvergenceWarning: Stochastic Optimizer: Maximum iterations (1000) reached and
the optimization hasn't converged yet.
  warnings.warn(
/projects/proteinml/.links/miniconda3/envs/aidep/lib/python3.10/site-
packages/sklearn/neural_network/_multilayer_perceptron.py:690:
ConvergenceWarning: Stochastic Optimizer: Maximum iterations (1000) reached and
the optimization hasn't converged yet.
  warnings.warn(
/projects/proteinml/.links/miniconda3/envs/aidep/lib/python3.10/site-
packages/sklearn/neural_network/_multilayer_perceptron.py:690:
ConvergenceWarning: Stochastic Optimizer: Maximum iterations (1000) reached and
the optimization hasn't converged yet.
  warnings.warn(
/projects/proteinml/.links/miniconda3/envs/aidep/lib/python3.10/site-
```

```
packages/sklearn/neural_network/_multilayer_perceptron.py:690:
ConvergenceWarning: Stochastic Optimizer: Maximum iterations (1000) reached and
the optimization hasn't converged yet.
  warnings.warn(
/projects/proteinml/.links/miniconda3/envs/aidep/lib/python3.10/site-
packages/sklearn/neural_network/_multilayer_perceptron.py:690:
ConvergenceWarning: Stochastic Optimizer: Maximum iterations (1000) reached and
the optimization hasn't converged yet.
  warnings.warn(
/projects/proteinml/.links/miniconda3/envs/aidep/lib/python3.10/site-
packages/sklearn/neural_network/_multilayer_perceptron.py:690:
ConvergenceWarning: Stochastic Optimizer: Maximum iterations (1000) reached and
the optimization hasn't converged yet.
  warnings.warn(
/projects/proteinml/.links/miniconda3/envs/aidep/lib/python3.10/site-
packages/sklearn/neural_network/_multilayer_perceptron.py:690:
ConvergenceWarning: Stochastic Optimizer: Maximum iterations (1000) reached and
the optimization hasn't converged yet.
  warnings.warn(
/projects/proteinml/.links/miniconda3/envs/aidep/lib/python3.10/site-
packages/sklearn/neural_network/_multilayer_perceptron.py:690:
ConvergenceWarning: Stochastic Optimizer: Maximum iterations (1000) reached and
the optimization hasn't converged yet.
  warnings.warn(
/projects/proteinml/.links/miniconda3/envs/aidep/lib/python3.10/site-
packages/sklearn/neural_network/_multilayer_perceptron.py:690:
ConvergenceWarning: Stochastic Optimizer: Maximum iterations (1000) reached and
the optimization hasn't converged yet.
  warnings.warn(
/projects/proteinml/.links/miniconda3/envs/aidep/lib/python3.10/site-
packages/sklearn/neural_network/_multilayer_perceptron.py:690:
ConvergenceWarning: Stochastic Optimizer: Maximum iterations (1000) reached and
the optimization hasn't converged yet.
  warnings.warn(
/projects/proteinml/.links/miniconda3/envs/aidep/lib/python3.10/site-
packages/sklearn/neural_network/_multilayer_perceptron.py:690:
ConvergenceWarning: Stochastic Optimizer: Maximum iterations (1000) reached and
the optimization hasn't converged yet.
  warnings.warn(
/projects/proteinml/.links/miniconda3/envs/aidep/lib/python3.10/site-
packages/sklearn/neural_network/_multilayer_perceptron.py:690:
ConvergenceWarning: Stochastic Optimizer: Maximum iterations (1000) reached and
the optimization hasn't converged yet.
  warnings.warn(
/projects/proteinml/.links/miniconda3/envs/aidep/lib/python3.10/site-
packages/sklearn/neural_network/_multilayer_perceptron.py:690:
ConvergenceWarning: Stochastic Optimizer: Maximum iterations (1000) reached and
the optimization hasn't converged yet.
```

```
  warnings.warn(
/projects/proteinml/.links/miniconda3/envs/aidep/lib/python3.10/site-
packages/sklearn/neural_network/_multilayer_perceptron.py:690:
ConvergenceWarning: Stochastic Optimizer: Maximum iterations (1000) reached and
the optimization hasn't converged yet.
  warnings.warn(
/projects/proteinml/.links/miniconda3/envs/aidep/lib/python3.10/site-
packages/sklearn/neural_network/_multilayer_perceptron.py:690:
ConvergenceWarning: Stochastic Optimizer: Maximum iterations (1000) reached and
the optimization hasn't converged yet.
  warnings.warn(
/projects/proteinml/.links/miniconda3/envs/aidep/lib/python3.10/site-
packages/sklearn/neural_network/_multilayer_perceptron.py:690:
ConvergenceWarning: Stochastic Optimizer: Maximum iterations (1000) reached and
the optimization hasn't converged yet.
  warnings.warn(
/projects/proteinml/.links/miniconda3/envs/aidep/lib/python3.10/site-
packages/sklearn/neural_network/_multilayer_perceptron.py:690:
ConvergenceWarning: Stochastic Optimizer: Maximum iterations (1000) reached and
the optimization hasn't converged yet.
  warnings.warn(
/projects/proteinml/.links/miniconda3/envs/aidep/lib/python3.10/site-
packages/sklearn/neural_network/_multilayer_perceptron.py:690:
ConvergenceWarning: Stochastic Optimizer: Maximum iterations (1000) reached and
the optimization hasn't converged yet.
  warnings.warn(
/projects/proteinml/.links/miniconda3/envs/aidep/lib/python3.10/site-
packages/sklearn/neural_network/_multilayer_perceptron.py:690:
ConvergenceWarning: Stochastic Optimizer: Maximum iterations (1000) reached and
the optimization hasn't converged yet.
  warnings.warn(
/projects/proteinml/.links/miniconda3/envs/aidep/lib/python3.10/site-
packages/sklearn/neural_network/_multilayer_perceptron.py:690:
ConvergenceWarning: Stochastic Optimizer: Maximum iterations (1000) reached and
the optimization hasn't converged yet.
  warnings.warn(
/projects/proteinml/.links/miniconda3/envs/aidep/lib/python3.10/site-
packages/sklearn/neural_network/_multilayer_perceptron.py:690:
ConvergenceWarning: Stochastic Optimizer: Maximum iterations (1000) reached and
the optimization hasn't converged yet.
  warnings.warn(
/projects/proteinml/.links/miniconda3/envs/aidep/lib/python3.10/site-
packages/sklearn/neural_network/_multilayer_perceptron.py:690:
ConvergenceWarning: Stochastic Optimizer: Maximum iterations (1000) reached and
the optimization hasn't converged yet.
  warnings.warn(
/projects/proteinml/.links/miniconda3/envs/aidep/lib/python3.10/site-
packages/sklearn/neural_network/_multilayer_perceptron.py:690:
```

```
ConvergenceWarning: Stochastic Optimizer: Maximum iterations (1000) reached and
the optimization hasn't converged yet.
  warnings.warn(
/projects/proteinml/.links/miniconda3/envs/aidep/lib/python3.10/site-
packages/sklearn/neural_network/_multilayer_perceptron.py:690:
ConvergenceWarning: Stochastic Optimizer: Maximum iterations (1000) reached and
the optimization hasn't converged yet.
  warnings.warn(
/projects/proteinml/.links/miniconda3/envs/aidep/lib/python3.10/site-
packages/sklearn/neural_network/_multilayer_perceptron.py:690:
ConvergenceWarning: Stochastic Optimizer: Maximum iterations (1000) reached and
the optimization hasn't converged yet.
  warnings.warn(
/projects/proteinml/.links/miniconda3/envs/aidep/lib/python3.10/site-
packages/sklearn/neural_network/_multilayer_perceptron.py:690:
ConvergenceWarning: Stochastic Optimizer: Maximum iterations (1000) reached and
the optimization hasn't converged yet.
  warnings.warn(
/projects/proteinml/.links/miniconda3/envs/aidep/lib/python3.10/site-
packages/sklearn/neural_network/_multilayer_perceptron.py:690:
ConvergenceWarning: Stochastic Optimizer: Maximum iterations (1000) reached and
the optimization hasn't converged yet.
  warnings.warn(
/projects/proteinml/.links/miniconda3/envs/aidep/lib/python3.10/site-
packages/sklearn/neural_network/_multilayer_perceptron.py:690:
ConvergenceWarning: Stochastic Optimizer: Maximum iterations (1000) reached and
the optimization hasn't converged yet.
  warnings.warn(
/projects/proteinml/.links/miniconda3/envs/aidep/lib/python3.10/site-
packages/sklearn/neural_network/_multilayer_perceptron.py:690:
ConvergenceWarning: Stochastic Optimizer: Maximum iterations (1000) reached and
the optimization hasn't converged yet.
  warnings.warn(
/projects/proteinml/.links/miniconda3/envs/aidep/lib/python3.10/site-
packages/sklearn/neural_network/_multilayer_perceptron.py:690:
ConvergenceWarning: Stochastic Optimizer: Maximum iterations (1000) reached and
the optimization hasn't converged yet.
  warnings.warn(
/projects/proteinml/.links/miniconda3/envs/aidep/lib/python3.10/site-
packages/sklearn/neural_network/_multilayer_perceptron.py:690:
ConvergenceWarning: Stochastic Optimizer: Maximum iterations (1000) reached and
the optimization hasn't converged yet.
  warnings.warn(
/projects/proteinml/.links/miniconda3/envs/aidep/lib/python3.10/site-
packages/sklearn/neural_network/_multilayer_perceptron.py:690:
ConvergenceWarning: Stochastic Optimizer: Maximum iterations (1000) reached and
the optimization hasn't converged yet.
  warnings.warn(
```

```
/projects/proteinml/.links/miniconda3/envs/aidep/lib/python3.10/site-
packages/sklearn/neural_network/_multilayer_perceptron.py:690:
ConvergenceWarning: Stochastic Optimizer: Maximum iterations (1000) reached and
the optimization hasn't converged yet.
  warnings.warn(
/projects/proteinml/.links/miniconda3/envs/aidep/lib/python3.10/site-
packages/sklearn/neural_network/_multilayer_perceptron.py:690:
ConvergenceWarning: Stochastic Optimizer: Maximum iterations (1000) reached and
the optimization hasn't converged yet.
  warnings.warn(
/projects/proteinml/.links/miniconda3/envs/aidep/lib/python3.10/site-
packages/sklearn/neural_network/_multilayer_perceptron.py:690:
ConvergenceWarning: Stochastic Optimizer: Maximum iterations (1000) reached and
the optimization hasn't converged yet.
  warnings.warn(
/projects/proteinml/.links/miniconda3/envs/aidep/lib/python3.10/site-
packages/sklearn/neural_network/_multilayer_perceptron.py:690:
ConvergenceWarning: Stochastic Optimizer: Maximum iterations (1000) reached and
the optimization hasn't converged yet.
  warnings.warn(
/projects/proteinml/.links/miniconda3/envs/aidep/lib/python3.10/site-
packages/sklearn/neural_network/_multilayer_perceptron.py:690:
ConvergenceWarning: Stochastic Optimizer: Maximum iterations (1000) reached and
the optimization hasn't converged yet.
  warnings.warn(
/projects/proteinml/.links/miniconda3/envs/aidep/lib/python3.10/site-
packages/sklearn/neural_network/_multilayer_perceptron.py:690:
ConvergenceWarning: Stochastic Optimizer: Maximum iterations (1000) reached and
the optimization hasn't converged yet.
  warnings.warn(
/projects/proteinml/.links/miniconda3/envs/aidep/lib/python3.10/site-
packages/sklearn/neural_network/_multilayer_perceptron.py:690:
ConvergenceWarning: Stochastic Optimizer: Maximum iterations (1000) reached and
the optimization hasn't converged yet.
  warnings.warn(
/projects/proteinml/.links/miniconda3/envs/aidep/lib/python3.10/site-
packages/sklearn/neural_network/_multilayer_perceptron.py:690:
ConvergenceWarning: Stochastic Optimizer: Maximum iterations (1000) reached and
the optimization hasn't converged yet.
  warnings.warn(
/projects/proteinml/.links/miniconda3/envs/aidep/lib/python3.10/site-
packages/sklearn/neural_network/_multilayer_perceptron.py:690:
ConvergenceWarning: Stochastic Optimizer: Maximum iterations (1000) reached and
the optimization hasn't converged yet.
  warnings.warn(
/projects/proteinml/.links/miniconda3/envs/aidep/lib/python3.10/site-
packages/sklearn/neural_network/_multilayer_perceptron.py:690:
ConvergenceWarning: Stochastic Optimizer: Maximum iterations (1000) reached and
```

```
the optimization hasn't converged yet.
  warnings.warn(
/projects/proteinml/.links/miniconda3/envs/aidep/lib/python3.10/site-
packages/sklearn/neural_network/_multilayer_perceptron.py:690:
ConvergenceWarning: Stochastic Optimizer: Maximum iterations (1000) reached and
the optimization hasn't converged yet.
  warnings.warn(
/projects/proteinml/.links/miniconda3/envs/aidep/lib/python3.10/site-
packages/sklearn/neural_network/_multilayer_perceptron.py:690:
ConvergenceWarning: Stochastic Optimizer: Maximum iterations (1000) reached and
the optimization hasn't converged yet.
  warnings.warn(
/projects/proteinml/.links/miniconda3/envs/aidep/lib/python3.10/site-
packages/sklearn/neural_network/_multilayer_perceptron.py:690:
ConvergenceWarning: Stochastic Optimizer: Maximum iterations (1000) reached and
the optimization hasn't converged yet.
  warnings.warn(
/projects/proteinml/.links/miniconda3/envs/aidep/lib/python3.10/site-
packages/sklearn/neural_network/_multilayer_perceptron.py:690:
ConvergenceWarning: Stochastic Optimizer: Maximum iterations (1000) reached and
the optimization hasn't converged yet.
  warnings.warn(
/projects/proteinml/.links/miniconda3/envs/aidep/lib/python3.10/site-
packages/sklearn/neural_network/_multilayer_perceptron.py:690:
ConvergenceWarning: Stochastic Optimizer: Maximum iterations (1000) reached and
the optimization hasn't converged yet.
  warnings.warn(
/projects/proteinml/.links/miniconda3/envs/aidep/lib/python3.10/site-
packages/sklearn/neural_network/_multilayer_perceptron.py:690:
ConvergenceWarning: Stochastic Optimizer: Maximum iterations (1000) reached and
the optimization hasn't converged yet.
  warnings.warn(
/projects/proteinml/.links/miniconda3/envs/aidep/lib/python3.10/site-
packages/sklearn/neural_network/_multilayer_perceptron.py:690:
ConvergenceWarning: Stochastic Optimizer: Maximum iterations (1000) reached and
the optimization hasn't converged yet.
  warnings.warn(
/projects/proteinml/.links/miniconda3/envs/aidep/lib/python3.10/site-
packages/sklearn/neural_network/_multilayer_perceptron.py:690:
ConvergenceWarning: Stochastic Optimizer: Maximum iterations (1000) reached and
the optimization hasn't converged yet.
  warnings.warn(
/projects/proteinml/.links/miniconda3/envs/aidep/lib/python3.10/site-
packages/sklearn/neural_network/_multilayer_perceptron.py:690:
ConvergenceWarning: Stochastic Optimizer: Maximum iterations (1000) reached and
the optimization hasn't converged yet.
  warnings.warn(
/projects/proteinml/.links/miniconda3/envs/aidep/lib/python3.10/site-
```

```
packages/sklearn/neural_network/_multilayer_perceptron.py:690:
ConvergenceWarning: Stochastic Optimizer: Maximum iterations (1000) reached and
the optimization hasn't converged yet.
  warnings.warn(
/projects/proteinml/.links/miniconda3/envs/aidep/lib/python3.10/site-
packages/sklearn/neural_network/_multilayer_perceptron.py:690:
ConvergenceWarning: Stochastic Optimizer: Maximum iterations (1000) reached and
the optimization hasn't converged yet.
  warnings.warn(
/projects/proteinml/.links/miniconda3/envs/aidep/lib/python3.10/site-
packages/sklearn/neural_network/_multilayer_perceptron.py:690:
ConvergenceWarning: Stochastic Optimizer: Maximum iterations (1000) reached and
the optimization hasn't converged yet.
  warnings.warn(
/projects/proteinml/.links/miniconda3/envs/aidep/lib/python3.10/site-
packages/sklearn/neural_network/_multilayer_perceptron.py:690:
ConvergenceWarning: Stochastic Optimizer: Maximum iterations (1000) reached and
the optimization hasn't converged yet.
  warnings.warn(
/projects/proteinml/.links/miniconda3/envs/aidep/lib/python3.10/site-
packages/sklearn/neural_network/_multilayer_perceptron.py:690:
ConvergenceWarning: Stochastic Optimizer: Maximum iterations (1000) reached and
the optimization hasn't converged yet.
  warnings.warn(
/projects/proteinml/.links/miniconda3/envs/aidep/lib/python3.10/site-
packages/sklearn/neural_network/_multilayer_perceptron.py:690:
ConvergenceWarning: Stochastic Optimizer: Maximum iterations (1000) reached and
the optimization hasn't converged yet.
  warnings.warn(
/projects/proteinml/.links/miniconda3/envs/aidep/lib/python3.10/site-
packages/sklearn/neural_network/_multilayer_perceptron.py:690:
ConvergenceWarning: Stochastic Optimizer: Maximum iterations (1000) reached and
the optimization hasn't converged yet.
  warnings.warn(
/projects/proteinml/.links/miniconda3/envs/aidep/lib/python3.10/site-
packages/sklearn/neural_network/_multilayer_perceptron.py:690:
ConvergenceWarning: Stochastic Optimizer: Maximum iterations (1000) reached and
the optimization hasn't converged yet.
  warnings.warn(
/projects/proteinml/.links/miniconda3/envs/aidep/lib/python3.10/site-
packages/sklearn/neural_network/_multilayer_perceptron.py:690:
ConvergenceWarning: Stochastic Optimizer: Maximum iterations (1000) reached and
the optimization hasn't converged yet.
  warnings.warn(
/projects/proteinml/.links/miniconda3/envs/aidep/lib/python3.10/site-
packages/sklearn/neural_network/_multilayer_perceptron.py:690:
ConvergenceWarning: Stochastic Optimizer: Maximum iterations (1000) reached and
the optimization hasn't converged yet.
```

```
  warnings.warn(
/projects/proteinml/.links/miniconda3/envs/aidep/lib/python3.10/site-
packages/sklearn/neural_network/_multilayer_perceptron.py:690:
ConvergenceWarning: Stochastic Optimizer: Maximum iterations (1000) reached and
the optimization hasn't converged yet.
  warnings.warn(
/projects/proteinml/.links/miniconda3/envs/aidep/lib/python3.10/site-
packages/sklearn/neural_network/_multilayer_perceptron.py:690:
ConvergenceWarning: Stochastic Optimizer: Maximum iterations (1000) reached and
the optimization hasn't converged yet.
  warnings.warn(
/projects/proteinml/.links/miniconda3/envs/aidep/lib/python3.10/site-
packages/sklearn/neural_network/_multilayer_perceptron.py:690:
ConvergenceWarning: Stochastic Optimizer: Maximum iterations (1000) reached and
the optimization hasn't converged yet.
  warnings.warn(
/projects/proteinml/.links/miniconda3/envs/aidep/lib/python3.10/site-
packages/sklearn/neural_network/_multilayer_perceptron.py:690:
ConvergenceWarning: Stochastic Optimizer: Maximum iterations (1000) reached and
the optimization hasn't converged yet.
  warnings.warn(
/projects/proteinml/.links/miniconda3/envs/aidep/lib/python3.10/site-
packages/sklearn/neural_network/_multilayer_perceptron.py:690:
ConvergenceWarning: Stochastic Optimizer: Maximum iterations (1000) reached and
the optimization hasn't converged yet.
  warnings.warn(
/projects/proteinml/.links/miniconda3/envs/aidep/lib/python3.10/site-
packages/sklearn/neural_network/_multilayer_perceptron.py:690:
ConvergenceWarning: Stochastic Optimizer: Maximum iterations (1000) reached and
the optimization hasn't converged yet.
  warnings.warn(
/projects/proteinml/.links/miniconda3/envs/aidep/lib/python3.10/site-
packages/sklearn/neural_network/_multilayer_perceptron.py:690:
ConvergenceWarning: Stochastic Optimizer: Maximum iterations (1000) reached and
the optimization hasn't converged yet.
  warnings.warn(
/projects/proteinml/.links/miniconda3/envs/aidep/lib/python3.10/site-
packages/sklearn/neural_network/_multilayer_perceptron.py:690:
ConvergenceWarning: Stochastic Optimizer: Maximum iterations (1000) reached and
the optimization hasn't converged yet.
  warnings.warn(
/projects/proteinml/.links/miniconda3/envs/aidep/lib/python3.10/site-
packages/sklearn/neural_network/_multilayer_perceptron.py:690:
ConvergenceWarning: Stochastic Optimizer: Maximum iterations (1000) reached and
the optimization hasn't converged yet.
  warnings.warn(
/projects/proteinml/.links/miniconda3/envs/aidep/lib/python3.10/site-
packages/sklearn/neural_network/_multilayer_perceptron.py:690:
```

```
ConvergenceWarning: Stochastic Optimizer: Maximum iterations (1000) reached and
the optimization hasn't converged yet.
  warnings.warn(
/projects/proteinml/.links/miniconda3/envs/aidep/lib/python3.10/site-
packages/sklearn/neural_network/_multilayer_perceptron.py:690:
ConvergenceWarning: Stochastic Optimizer: Maximum iterations (1000) reached and
the optimization hasn't converged yet.
  warnings.warn(
/projects/proteinml/.links/miniconda3/envs/aidep/lib/python3.10/site-
packages/sklearn/neural_network/_multilayer_perceptron.py:690:
ConvergenceWarning: Stochastic Optimizer: Maximum iterations (1000) reached and
the optimization hasn't converged yet.
  warnings.warn(
/projects/proteinml/.links/miniconda3/envs/aidep/lib/python3.10/site-
packages/sklearn/neural_network/_multilayer_perceptron.py:690:
ConvergenceWarning: Stochastic Optimizer: Maximum iterations (1000) reached and
the optimization hasn't converged yet.
  warnings.warn(
/projects/proteinml/.links/miniconda3/envs/aidep/lib/python3.10/site-
packages/sklearn/neural_network/_multilayer_perceptron.py:690:
ConvergenceWarning: Stochastic Optimizer: Maximum iterations (1000) reached and
the optimization hasn't converged yet.
  warnings.warn(
/projects/proteinml/.links/miniconda3/envs/aidep/lib/python3.10/site-
packages/sklearn/neural_network/_multilayer_perceptron.py:690:
ConvergenceWarning: Stochastic Optimizer: Maximum iterations (1000) reached and
the optimization hasn't converged yet.
  warnings.warn(
/projects/proteinml/.links/miniconda3/envs/aidep/lib/python3.10/site-
packages/sklearn/neural_network/_multilayer_perceptron.py:690:
ConvergenceWarning: Stochastic Optimizer: Maximum iterations (1000) reached and
the optimization hasn't converged yet.
  warnings.warn(
/projects/proteinml/.links/miniconda3/envs/aidep/lib/python3.10/site-
packages/sklearn/neural_network/_multilayer_perceptron.py:690:
ConvergenceWarning: Stochastic Optimizer: Maximum iterations (1000) reached and
the optimization hasn't converged yet.
  warnings.warn(
/projects/proteinml/.links/miniconda3/envs/aidep/lib/python3.10/site-
packages/sklearn/neural_network/_multilayer_perceptron.py:690:
ConvergenceWarning: Stochastic Optimizer: Maximum iterations (1000) reached and
the optimization hasn't converged yet.
  warnings.warn(
/projects/proteinml/.links/miniconda3/envs/aidep/lib/python3.10/site-
packages/sklearn/neural_network/_multilayer_perceptron.py:690:
ConvergenceWarning: Stochastic Optimizer: Maximum iterations (1000) reached and
the optimization hasn't converged yet.
  warnings.warn(
```

```
/projects/proteinml/.links/miniconda3/envs/aidep/lib/python3.10/site-
packages/sklearn/neural_network/_multilayer_perceptron.py:690:
ConvergenceWarning: Stochastic Optimizer: Maximum iterations (1000) reached and
the optimization hasn't converged yet.
  warnings.warn(
/projects/proteinml/.links/miniconda3/envs/aidep/lib/python3.10/site-
packages/sklearn/neural_network/_multilayer_perceptron.py:690:
ConvergenceWarning: Stochastic Optimizer: Maximum iterations (1000) reached and
the optimization hasn't converged yet.
  warnings.warn(
/projects/proteinml/.links/miniconda3/envs/aidep/lib/python3.10/site-
packages/sklearn/neural_network/_multilayer_perceptron.py:690:
ConvergenceWarning: Stochastic Optimizer: Maximum iterations (1000) reached and
the optimization hasn't converged yet.
  warnings.warn(
/projects/proteinml/.links/miniconda3/envs/aidep/lib/python3.10/site-
packages/sklearn/neural_network/_multilayer_perceptron.py:690:
ConvergenceWarning: Stochastic Optimizer: Maximum iterations (1000) reached and
the optimization hasn't converged yet.
  warnings.warn(
/projects/proteinml/.links/miniconda3/envs/aidep/lib/python3.10/site-
packages/sklearn/neural_network/_multilayer_perceptron.py:690:
ConvergenceWarning: Stochastic Optimizer: Maximum iterations (1000) reached and
the optimization hasn't converged yet.
  warnings.warn(
/projects/proteinml/.links/miniconda3/envs/aidep/lib/python3.10/site-
packages/sklearn/neural_network/_multilayer_perceptron.py:690:
ConvergenceWarning: Stochastic Optimizer: Maximum iterations (1000) reached and
the optimization hasn't converged yet.
  warnings.warn(
/projects/proteinml/.links/miniconda3/envs/aidep/lib/python3.10/site-
packages/sklearn/neural_network/_multilayer_perceptron.py:690:
ConvergenceWarning: Stochastic Optimizer: Maximum iterations (1000) reached and
the optimization hasn't converged yet.
  warnings.warn(
/projects/proteinml/.links/miniconda3/envs/aidep/lib/python3.10/site-
packages/sklearn/neural_network/_multilayer_perceptron.py:690:
ConvergenceWarning: Stochastic Optimizer: Maximum iterations (1000) reached and
the optimization hasn't converged yet.
  warnings.warn(
/projects/proteinml/.links/miniconda3/envs/aidep/lib/python3.10/site-
packages/sklearn/neural_network/_multilayer_perceptron.py:690:
ConvergenceWarning: Stochastic Optimizer: Maximum iterations (1000) reached and
the optimization hasn't converged yet.
  warnings.warn(
/projects/proteinml/.links/miniconda3/envs/aidep/lib/python3.10/site-
packages/sklearn/neural_network/_multilayer_perceptron.py:690:
ConvergenceWarning: Stochastic Optimizer: Maximum iterations (1000) reached and
```

```
the optimization hasn't converged yet.
  warnings.warn(
/projects/proteinml/.links/miniconda3/envs/aidep/lib/python3.10/site-
packages/sklearn/neural_network/_multilayer_perceptron.py:690:
ConvergenceWarning: Stochastic Optimizer: Maximum iterations (1000) reached and
the optimization hasn't converged yet.
  warnings.warn(
/projects/proteinml/.links/miniconda3/envs/aidep/lib/python3.10/site-
packages/sklearn/neural_network/_multilayer_perceptron.py:690:
ConvergenceWarning: Stochastic Optimizer: Maximum iterations (1000) reached and
the optimization hasn't converged yet.
  warnings.warn(
/projects/proteinml/.links/miniconda3/envs/aidep/lib/python3.10/site-
packages/sklearn/neural_network/_multilayer_perceptron.py:690:
ConvergenceWarning: Stochastic Optimizer: Maximum iterations (1000) reached and
the optimization hasn't converged yet.
  warnings.warn(
/projects/proteinml/.links/miniconda3/envs/aidep/lib/python3.10/site-
packages/sklearn/neural_network/_multilayer_perceptron.py:690:
ConvergenceWarning: Stochastic Optimizer: Maximum iterations (1000) reached and
the optimization hasn't converged yet.
  warnings.warn(
/projects/proteinml/.links/miniconda3/envs/aidep/lib/python3.10/site-
packages/sklearn/neural_network/_multilayer_perceptron.py:690:
ConvergenceWarning: Stochastic Optimizer: Maximum iterations (1000) reached and
the optimization hasn't converged yet.
  warnings.warn(
/projects/proteinml/.links/miniconda3/envs/aidep/lib/python3.10/site-
packages/sklearn/neural_network/_multilayer_perceptron.py:690:
ConvergenceWarning: Stochastic Optimizer: Maximum iterations (1000) reached and
the optimization hasn't converged yet.
  warnings.warn(
/projects/proteinml/.links/miniconda3/envs/aidep/lib/python3.10/site-
packages/sklearn/neural_network/_multilayer_perceptron.py:690:
ConvergenceWarning: Stochastic Optimizer: Maximum iterations (1000) reached and
the optimization hasn't converged yet.
  warnings.warn(
/projects/proteinml/.links/miniconda3/envs/aidep/lib/python3.10/site-
packages/sklearn/neural_network/_multilayer_perceptron.py:690:
ConvergenceWarning: Stochastic Optimizer: Maximum iterations (1000) reached and
the optimization hasn't converged yet.
  warnings.warn(
/projects/proteinml/.links/miniconda3/envs/aidep/lib/python3.10/site-
packages/sklearn/neural_network/_multilayer_perceptron.py:690:
ConvergenceWarning: Stochastic Optimizer: Maximum iterations (1000) reached and
the optimization hasn't converged yet.
  warnings.warn(
/projects/proteinml/.links/miniconda3/envs/aidep/lib/python3.10/site-
```

```
packages/sklearn/neural_network/_multilayer_perceptron.py:690:
ConvergenceWarning: Stochastic Optimizer: Maximum iterations (1000) reached and
the optimization hasn't converged yet.
  warnings.warn(
/projects/proteinml/.links/miniconda3/envs/aidep/lib/python3.10/site-
packages/sklearn/neural_network/_multilayer_perceptron.py:690:
ConvergenceWarning: Stochastic Optimizer: Maximum iterations (1000) reached and
the optimization hasn't converged yet.
  warnings.warn(
/projects/proteinml/.links/miniconda3/envs/aidep/lib/python3.10/site-
packages/sklearn/neural_network/_multilayer_perceptron.py:690:
ConvergenceWarning: Stochastic Optimizer: Maximum iterations (1000) reached and
the optimization hasn't converged yet.
  warnings.warn(
/projects/proteinml/.links/miniconda3/envs/aidep/lib/python3.10/site-
packages/sklearn/neural_network/_multilayer_perceptron.py:690:
ConvergenceWarning: Stochastic Optimizer: Maximum iterations (1000) reached and
the optimization hasn't converged yet.
  warnings.warn(
/projects/proteinml/.links/miniconda3/envs/aidep/lib/python3.10/site-
packages/sklearn/neural_network/_multilayer_perceptron.py:690:
ConvergenceWarning: Stochastic Optimizer: Maximum iterations (1000) reached and
the optimization hasn't converged yet.
  warnings.warn(
/projects/proteinml/.links/miniconda3/envs/aidep/lib/python3.10/site-
packages/sklearn/neural_network/_multilayer_perceptron.py:690:
ConvergenceWarning: Stochastic Optimizer: Maximum iterations (1000) reached and
the optimization hasn't converged yet.
  warnings.warn(
/projects/proteinml/.links/miniconda3/envs/aidep/lib/python3.10/site-
packages/sklearn/neural_network/_multilayer_perceptron.py:690:
ConvergenceWarning: Stochastic Optimizer: Maximum iterations (1000) reached and
the optimization hasn't converged yet.
  warnings.warn(
/projects/proteinml/.links/miniconda3/envs/aidep/lib/python3.10/site-
packages/sklearn/neural_network/_multilayer_perceptron.py:690:
ConvergenceWarning: Stochastic Optimizer: Maximum iterations (1000) reached and
the optimization hasn't converged yet.
  warnings.warn(
/projects/proteinml/.links/miniconda3/envs/aidep/lib/python3.10/site-
packages/sklearn/neural_network/_multilayer_perceptron.py:690:
ConvergenceWarning: Stochastic Optimizer: Maximum iterations (1000) reached and
the optimization hasn't converged yet.
  warnings.warn(
/projects/proteinml/.links/miniconda3/envs/aidep/lib/python3.10/site-
packages/sklearn/neural_network/_multilayer_perceptron.py:690:
ConvergenceWarning: Stochastic Optimizer: Maximum iterations (1000) reached and
the optimization hasn't converged yet.
```

```
  warnings.warn(
/projects/proteinml/.links/miniconda3/envs/aidep/lib/python3.10/site-
packages/sklearn/neural_network/_multilayer_perceptron.py:690:
ConvergenceWarning: Stochastic Optimizer: Maximum iterations (1000) reached and
the optimization hasn't converged yet.
  warnings.warn(
/projects/proteinml/.links/miniconda3/envs/aidep/lib/python3.10/site-
packages/sklearn/neural_network/_multilayer_perceptron.py:690:
ConvergenceWarning: Stochastic Optimizer: Maximum iterations (1000) reached and
the optimization hasn't converged yet.
  warnings.warn(
/projects/proteinml/.links/miniconda3/envs/aidep/lib/python3.10/site-
packages/sklearn/neural_network/_multilayer_perceptron.py:690:
ConvergenceWarning: Stochastic Optimizer: Maximum iterations (1000) reached and
the optimization hasn't converged yet.
  warnings.warn(
/projects/proteinml/.links/miniconda3/envs/aidep/lib/python3.10/site-
packages/sklearn/neural_network/_multilayer_perceptron.py:690:
ConvergenceWarning: Stochastic Optimizer: Maximum iterations (1000) reached and
the optimization hasn't converged yet.
  warnings.warn(
/projects/proteinml/.links/miniconda3/envs/aidep/lib/python3.10/site-
packages/sklearn/neural_network/_multilayer_perceptron.py:690:
ConvergenceWarning: Stochastic Optimizer: Maximum iterations (1000) reached and
the optimization hasn't converged yet.
  warnings.warn(
/projects/proteinml/.links/miniconda3/envs/aidep/lib/python3.10/site-
packages/sklearn/neural_network/_multilayer_perceptron.py:690:
ConvergenceWarning: Stochastic Optimizer: Maximum iterations (1000) reached and
the optimization hasn't converged yet.
  warnings.warn(
/projects/proteinml/.links/miniconda3/envs/aidep/lib/python3.10/site-
packages/sklearn/neural_network/_multilayer_perceptron.py:690:
ConvergenceWarning: Stochastic Optimizer: Maximum iterations (1000) reached and
the optimization hasn't converged yet.
  warnings.warn(
/projects/proteinml/.links/miniconda3/envs/aidep/lib/python3.10/site-
packages/sklearn/neural_network/_multilayer_perceptron.py:690:
ConvergenceWarning: Stochastic Optimizer: Maximum iterations (1000) reached and
the optimization hasn't converged yet.
  warnings.warn(
/projects/proteinml/.links/miniconda3/envs/aidep/lib/python3.10/site-
packages/sklearn/neural_network/_multilayer_perceptron.py:690:
ConvergenceWarning: Stochastic Optimizer: Maximum iterations (1000) reached and
the optimization hasn't converged yet.
  warnings.warn(
/projects/proteinml/.links/miniconda3/envs/aidep/lib/python3.10/site-
packages/sklearn/neural_network/_multilayer_perceptron.py:690:
```

```
ConvergenceWarning: Stochastic Optimizer: Maximum iterations (1000) reached and
the optimization hasn't converged yet.
  warnings.warn(
/projects/proteinml/.links/miniconda3/envs/aidep/lib/python3.10/site-
packages/sklearn/neural_network/_multilayer_perceptron.py:690:
ConvergenceWarning: Stochastic Optimizer: Maximum iterations (1000) reached and
the optimization hasn't converged yet.
  warnings.warn(
/projects/proteinml/.links/miniconda3/envs/aidep/lib/python3.10/site-
packages/sklearn/neural_network/_multilayer_perceptron.py:690:
ConvergenceWarning: Stochastic Optimizer: Maximum iterations (1000) reached and
the optimization hasn't converged yet.
  warnings.warn(
/projects/proteinml/.links/miniconda3/envs/aidep/lib/python3.10/site-
packages/sklearn/neural_network/_multilayer_perceptron.py:690:
ConvergenceWarning: Stochastic Optimizer: Maximum iterations (1000) reached and
the optimization hasn't converged yet.
  warnings.warn(
/projects/proteinml/.links/miniconda3/envs/aidep/lib/python3.10/site-
packages/sklearn/neural_network/_multilayer_perceptron.py:690:
ConvergenceWarning: Stochastic Optimizer: Maximum iterations (1000) reached and
the optimization hasn't converged yet.
  warnings.warn(
/projects/proteinml/.links/miniconda3/envs/aidep/lib/python3.10/site-
packages/sklearn/neural_network/_multilayer_perceptron.py:690:
ConvergenceWarning: Stochastic Optimizer: Maximum iterations (1000) reached and
the optimization hasn't converged yet.
  warnings.warn(
/projects/proteinml/.links/miniconda3/envs/aidep/lib/python3.10/site-
packages/sklearn/neural_network/_multilayer_perceptron.py:690:
ConvergenceWarning: Stochastic Optimizer: Maximum iterations (1000) reached and
the optimization hasn't converged yet.
  warnings.warn(
/projects/proteinml/.links/miniconda3/envs/aidep/lib/python3.10/site-
packages/sklearn/neural_network/_multilayer_perceptron.py:690:
ConvergenceWarning: Stochastic Optimizer: Maximum iterations (1000) reached and
the optimization hasn't converged yet.
  warnings.warn(
/projects/proteinml/.links/miniconda3/envs/aidep/lib/python3.10/site-
packages/sklearn/neural_network/_multilayer_perceptron.py:690:
ConvergenceWarning: Stochastic Optimizer: Maximum iterations (1000) reached and
the optimization hasn't converged yet.
  warnings.warn(
/projects/proteinml/.links/miniconda3/envs/aidep/lib/python3.10/site-
packages/sklearn/neural_network/_multilayer_perceptron.py:690:
ConvergenceWarning: Stochastic Optimizer: Maximum iterations (1000) reached and
the optimization hasn't converged yet.
  warnings.warn(
```

```
/projects/proteinml/.links/miniconda3/envs/aidep/lib/python3.10/site-
packages/sklearn/neural_network/_multilayer_perceptron.py:690:
ConvergenceWarning: Stochastic Optimizer: Maximum iterations (1000) reached and
the optimization hasn't converged yet.
  warnings.warn(
/projects/proteinml/.links/miniconda3/envs/aidep/lib/python3.10/site-
packages/sklearn/neural_network/_multilayer_perceptron.py:690:
ConvergenceWarning: Stochastic Optimizer: Maximum iterations (1000) reached and
the optimization hasn't converged yet.
  warnings.warn(
/projects/proteinml/.links/miniconda3/envs/aidep/lib/python3.10/site-
packages/sklearn/neural_network/_multilayer_perceptron.py:690:
ConvergenceWarning: Stochastic Optimizer: Maximum iterations (1000) reached and
the optimization hasn't converged yet.
  warnings.warn(
/projects/proteinml/.links/miniconda3/envs/aidep/lib/python3.10/site-
packages/sklearn/neural_network/_multilayer_perceptron.py:690:
ConvergenceWarning: Stochastic Optimizer: Maximum iterations (1000) reached and
the optimization hasn't converged yet.
  warnings.warn(
/projects/proteinml/.links/miniconda3/envs/aidep/lib/python3.10/site-
packages/sklearn/neural_network/_multilayer_perceptron.py:690:
ConvergenceWarning: Stochastic Optimizer: Maximum iterations (1000) reached and
the optimization hasn't converged yet.
  warnings.warn(
/projects/proteinml/.links/miniconda3/envs/aidep/lib/python3.10/site-
packages/sklearn/neural_network/_multilayer_perceptron.py:690:
ConvergenceWarning: Stochastic Optimizer: Maximum iterations (1000) reached and
the optimization hasn't converged yet.
  warnings.warn(
/projects/proteinml/.links/miniconda3/envs/aidep/lib/python3.10/site-
packages/sklearn/neural_network/_multilayer_perceptron.py:690:
ConvergenceWarning: Stochastic Optimizer: Maximum iterations (1000) reached and
the optimization hasn't converged yet.
  warnings.warn(
/projects/proteinml/.links/miniconda3/envs/aidep/lib/python3.10/site-
packages/sklearn/neural_network/_multilayer_perceptron.py:690:
ConvergenceWarning: Stochastic Optimizer: Maximum iterations (1000) reached and
the optimization hasn't converged yet.
  warnings.warn(
/projects/proteinml/.links/miniconda3/envs/aidep/lib/python3.10/site-
packages/sklearn/neural_network/_multilayer_perceptron.py:690:
ConvergenceWarning: Stochastic Optimizer: Maximum iterations (1000) reached and
the optimization hasn't converged yet.
  warnings.warn(
/projects/proteinml/.links/miniconda3/envs/aidep/lib/python3.10/site-
packages/sklearn/neural_network/_multilayer_perceptron.py:690:
ConvergenceWarning: Stochastic Optimizer: Maximum iterations (1000) reached and
```

```
the optimization hasn't converged yet.
  warnings.warn(
/projects/proteinml/.links/miniconda3/envs/aidep/lib/python3.10/site-
packages/sklearn/neural_network/_multilayer_perceptron.py:690:
ConvergenceWarning: Stochastic Optimizer: Maximum iterations (1000) reached and
the optimization hasn't converged yet.
  warnings.warn(
/projects/proteinml/.links/miniconda3/envs/aidep/lib/python3.10/site-
packages/sklearn/neural_network/_multilayer_perceptron.py:690:
ConvergenceWarning: Stochastic Optimizer: Maximum iterations (1000) reached and
the optimization hasn't converged yet.
  warnings.warn(
/projects/proteinml/.links/miniconda3/envs/aidep/lib/python3.10/site-
packages/sklearn/neural_network/_multilayer_perceptron.py:690:
ConvergenceWarning: Stochastic Optimizer: Maximum iterations (1000) reached and
the optimization hasn't converged yet.
  warnings.warn(
/projects/proteinml/.links/miniconda3/envs/aidep/lib/python3.10/site-
packages/sklearn/neural_network/_multilayer_perceptron.py:690:
ConvergenceWarning: Stochastic Optimizer: Maximum iterations (1000) reached and
the optimization hasn't converged yet.
  warnings.warn(
/projects/proteinml/.links/miniconda3/envs/aidep/lib/python3.10/site-
packages/sklearn/neural_network/_multilayer_perceptron.py:690:
ConvergenceWarning: Stochastic Optimizer: Maximum iterations (1000) reached and
the optimization hasn't converged yet.
  warnings.warn(
/projects/proteinml/.links/miniconda3/envs/aidep/lib/python3.10/site-
packages/sklearn/neural_network/_multilayer_perceptron.py:690:
ConvergenceWarning: Stochastic Optimizer: Maximum iterations (1000) reached and
the optimization hasn't converged yet.
  warnings.warn(
/projects/proteinml/.links/miniconda3/envs/aidep/lib/python3.10/site-
packages/sklearn/neural_network/_multilayer_perceptron.py:690:
ConvergenceWarning: Stochastic Optimizer: Maximum iterations (1000) reached and
the optimization hasn't converged yet.
  warnings.warn(
/projects/proteinml/.links/miniconda3/envs/aidep/lib/python3.10/site-
packages/sklearn/neural_network/_multilayer_perceptron.py:690:
ConvergenceWarning: Stochastic Optimizer: Maximum iterations (1000) reached and
the optimization hasn't converged yet.
  warnings.warn(
/projects/proteinml/.links/miniconda3/envs/aidep/lib/python3.10/site-
packages/sklearn/neural_network/_multilayer_perceptron.py:690:
ConvergenceWarning: Stochastic Optimizer: Maximum iterations (1000) reached and
the optimization hasn't converged yet.
  warnings.warn(
/projects/proteinml/.links/miniconda3/envs/aidep/lib/python3.10/site-
```

```
packages/sklearn/neural_network/_multilayer_perceptron.py:690:
ConvergenceWarning: Stochastic Optimizer: Maximum iterations (1000) reached and
the optimization hasn't converged yet.
  warnings.warn(
/projects/proteinml/.links/miniconda3/envs/aidep/lib/python3.10/site-
packages/sklearn/neural_network/_multilayer_perceptron.py:690:
ConvergenceWarning: Stochastic Optimizer: Maximum iterations (1000) reached and
the optimization hasn't converged yet.
  warnings.warn(
/projects/proteinml/.links/miniconda3/envs/aidep/lib/python3.10/site-
packages/sklearn/neural_network/_multilayer_perceptron.py:690:
ConvergenceWarning: Stochastic Optimizer: Maximum iterations (1000) reached and
the optimization hasn't converged yet.
  warnings.warn(
/projects/proteinml/.links/miniconda3/envs/aidep/lib/python3.10/site-
packages/sklearn/neural_network/_multilayer_perceptron.py:690:
ConvergenceWarning: Stochastic Optimizer: Maximum iterations (1000) reached and
the optimization hasn't converged yet.
  warnings.warn(
/projects/proteinml/.links/miniconda3/envs/aidep/lib/python3.10/site-
packages/sklearn/neural_network/_multilayer_perceptron.py:690:
ConvergenceWarning: Stochastic Optimizer: Maximum iterations (1000) reached and
the optimization hasn't converged yet.
  warnings.warn(
/projects/proteinml/.links/miniconda3/envs/aidep/lib/python3.10/site-
packages/sklearn/neural_network/_multilayer_perceptron.py:690:
ConvergenceWarning: Stochastic Optimizer: Maximum iterations (1000) reached and
the optimization hasn't converged yet.
  warnings.warn(
/projects/proteinml/.links/miniconda3/envs/aidep/lib/python3.10/site-
packages/sklearn/neural_network/_multilayer_perceptron.py:690:
ConvergenceWarning: Stochastic Optimizer: Maximum iterations (1000) reached and
the optimization hasn't converged yet.
  warnings.warn(
/projects/proteinml/.links/miniconda3/envs/aidep/lib/python3.10/site-
packages/sklearn/neural_network/_multilayer_perceptron.py:690:
ConvergenceWarning: Stochastic Optimizer: Maximum iterations (1000) reached and
the optimization hasn't converged yet.
  warnings.warn(
/projects/proteinml/.links/miniconda3/envs/aidep/lib/python3.10/site-
packages/sklearn/neural_network/_multilayer_perceptron.py:690:
ConvergenceWarning: Stochastic Optimizer: Maximum iterations (1000) reached and
the optimization hasn't converged yet.
  warnings.warn(
/projects/proteinml/.links/miniconda3/envs/aidep/lib/python3.10/site-
packages/sklearn/neural_network/_multilayer_perceptron.py:690:
ConvergenceWarning: Stochastic Optimizer: Maximum iterations (1000) reached and
the optimization hasn't converged yet.
```

```
  warnings.warn(
/projects/proteinml/.links/miniconda3/envs/aidep/lib/python3.10/site-
packages/sklearn/neural_network/_multilayer_perceptron.py:690:
ConvergenceWarning: Stochastic Optimizer: Maximum iterations (1000) reached and
the optimization hasn't converged yet.
  warnings.warn(
/projects/proteinml/.links/miniconda3/envs/aidep/lib/python3.10/site-
packages/sklearn/neural_network/_multilayer_perceptron.py:690:
ConvergenceWarning: Stochastic Optimizer: Maximum iterations (1000) reached and
the optimization hasn't converged yet.
  warnings.warn(
/projects/proteinml/.links/miniconda3/envs/aidep/lib/python3.10/site-
packages/sklearn/neural_network/_multilayer_perceptron.py:690:
ConvergenceWarning: Stochastic Optimizer: Maximum iterations (1000) reached and
the optimization hasn't converged yet.
  warnings.warn(
/projects/proteinml/.links/miniconda3/envs/aidep/lib/python3.10/site-
packages/sklearn/neural_network/_multilayer_perceptron.py:690:
ConvergenceWarning: Stochastic Optimizer: Maximum iterations (1000) reached and
the optimization hasn't converged yet.
  warnings.warn(
/projects/proteinml/.links/miniconda3/envs/aidep/lib/python3.10/site-
packages/sklearn/neural_network/_multilayer_perceptron.py:690:
ConvergenceWarning: Stochastic Optimizer: Maximum iterations (1000) reached and
the optimization hasn't converged yet.
  warnings.warn(
/projects/proteinml/.links/miniconda3/envs/aidep/lib/python3.10/site-
packages/sklearn/neural_network/_multilayer_perceptron.py:690:
ConvergenceWarning: Stochastic Optimizer: Maximum iterations (1000) reached and
the optimization hasn't converged yet.
  warnings.warn(
/projects/proteinml/.links/miniconda3/envs/aidep/lib/python3.10/site-
packages/sklearn/neural_network/_multilayer_perceptron.py:690:
ConvergenceWarning: Stochastic Optimizer: Maximum iterations (1000) reached and
the optimization hasn't converged yet.
  warnings.warn(
/projects/proteinml/.links/miniconda3/envs/aidep/lib/python3.10/site-
packages/sklearn/neural_network/_multilayer_perceptron.py:690:
ConvergenceWarning: Stochastic Optimizer: Maximum iterations (1000) reached and
the optimization hasn't converged yet.
  warnings.warn(
/projects/proteinml/.links/miniconda3/envs/aidep/lib/python3.10/site-
packages/sklearn/neural_network/_multilayer_perceptron.py:690:
ConvergenceWarning: Stochastic Optimizer: Maximum iterations (1000) reached and
the optimization hasn't converged yet.
  warnings.warn(
/projects/proteinml/.links/miniconda3/envs/aidep/lib/python3.10/site-
packages/sklearn/neural_network/_multilayer_perceptron.py:690:
```

```
ConvergenceWarning: Stochastic Optimizer: Maximum iterations (1000) reached and
the optimization hasn't converged yet.
  warnings.warn(
/projects/proteinml/.links/miniconda3/envs/aidep/lib/python3.10/site-
packages/sklearn/neural_network/_multilayer_perceptron.py:690:
ConvergenceWarning: Stochastic Optimizer: Maximum iterations (1000) reached and
the optimization hasn't converged yet.
  warnings.warn(
/projects/proteinml/.links/miniconda3/envs/aidep/lib/python3.10/site-
packages/sklearn/neural_network/_multilayer_perceptron.py:690:
ConvergenceWarning: Stochastic Optimizer: Maximum iterations (1000) reached and
the optimization hasn't converged yet.
  warnings.warn(
/projects/proteinml/.links/miniconda3/envs/aidep/lib/python3.10/site-
packages/sklearn/neural_network/_multilayer_perceptron.py:690:
ConvergenceWarning: Stochastic Optimizer: Maximum iterations (1000) reached and
the optimization hasn't converged yet.
  warnings.warn(
/projects/proteinml/.links/miniconda3/envs/aidep/lib/python3.10/site-
packages/sklearn/neural_network/_multilayer_perceptron.py:690:
ConvergenceWarning: Stochastic Optimizer: Maximum iterations (1000) reached and
the optimization hasn't converged yet.
  warnings.warn(
/projects/proteinml/.links/miniconda3/envs/aidep/lib/python3.10/site-
packages/sklearn/neural_network/_multilayer_perceptron.py:690:
ConvergenceWarning: Stochastic Optimizer: Maximum iterations (1000) reached and
the optimization hasn't converged yet.
  warnings.warn(
/projects/proteinml/.links/miniconda3/envs/aidep/lib/python3.10/site-
packages/sklearn/neural_network/_multilayer_perceptron.py:690:
ConvergenceWarning: Stochastic Optimizer: Maximum iterations (1000) reached and
the optimization hasn't converged yet.
  warnings.warn(
/projects/proteinml/.links/miniconda3/envs/aidep/lib/python3.10/site-
packages/sklearn/neural_network/_multilayer_perceptron.py:690:
ConvergenceWarning: Stochastic Optimizer: Maximum iterations (1000) reached and
the optimization hasn't converged yet.
  warnings.warn(
/projects/proteinml/.links/miniconda3/envs/aidep/lib/python3.10/site-
packages/sklearn/neural_network/_multilayer_perceptron.py:690:
ConvergenceWarning: Stochastic Optimizer: Maximum iterations (1000) reached and
the optimization hasn't converged yet.
  warnings.warn(
/projects/proteinml/.links/miniconda3/envs/aidep/lib/python3.10/site-
packages/sklearn/neural_network/_multilayer_perceptron.py:690:
ConvergenceWarning: Stochastic Optimizer: Maximum iterations (1000) reached and
the optimization hasn't converged yet.
  warnings.warn(
```

```
/projects/proteinml/.links/miniconda3/envs/aidep/lib/python3.10/site-
packages/sklearn/neural_network/_multilayer_perceptron.py:690:
ConvergenceWarning: Stochastic Optimizer: Maximum iterations (1000) reached and
the optimization hasn't converged yet.
  warnings.warn(
/projects/proteinml/.links/miniconda3/envs/aidep/lib/python3.10/site-
packages/sklearn/neural_network/_multilayer_perceptron.py:690:
ConvergenceWarning: Stochastic Optimizer: Maximum iterations (1000) reached and
the optimization hasn't converged yet.
  warnings.warn(
/projects/proteinml/.links/miniconda3/envs/aidep/lib/python3.10/site-
packages/sklearn/neural_network/_multilayer_perceptron.py:690:
ConvergenceWarning: Stochastic Optimizer: Maximum iterations (1000) reached and
the optimization hasn't converged yet.
  warnings.warn(
/projects/proteinml/.links/miniconda3/envs/aidep/lib/python3.10/site-
packages/sklearn/neural_network/_multilayer_perceptron.py:690:
ConvergenceWarning: Stochastic Optimizer: Maximum iterations (1000) reached and
the optimization hasn't converged yet.
  warnings.warn(
/projects/proteinml/.links/miniconda3/envs/aidep/lib/python3.10/site-
packages/sklearn/neural_network/_multilayer_perceptron.py:690:
ConvergenceWarning: Stochastic Optimizer: Maximum iterations (1000) reached and
the optimization hasn't converged yet.
  warnings.warn(
/projects/proteinml/.links/miniconda3/envs/aidep/lib/python3.10/site-
packages/sklearn/neural_network/_multilayer_perceptron.py:690:
ConvergenceWarning: Stochastic Optimizer: Maximum iterations (1000) reached and
the optimization hasn't converged yet.
  warnings.warn(
/projects/proteinml/.links/miniconda3/envs/aidep/lib/python3.10/site-
packages/sklearn/neural_network/_multilayer_perceptron.py:690:
ConvergenceWarning: Stochastic Optimizer: Maximum iterations (1000) reached and
the optimization hasn't converged yet.
  warnings.warn(
/projects/proteinml/.links/miniconda3/envs/aidep/lib/python3.10/site-
packages/sklearn/neural_network/_multilayer_perceptron.py:690:
ConvergenceWarning: Stochastic Optimizer: Maximum iterations (1000) reached and
the optimization hasn't converged yet.
  warnings.warn(
/projects/proteinml/.links/miniconda3/envs/aidep/lib/python3.10/site-
packages/sklearn/neural_network/_multilayer_perceptron.py:690:
ConvergenceWarning: Stochastic Optimizer: Maximum iterations (1000) reached and
the optimization hasn't converged yet.
  warnings.warn(
/projects/proteinml/.links/miniconda3/envs/aidep/lib/python3.10/site-
packages/sklearn/neural_network/_multilayer_perceptron.py:690:
ConvergenceWarning: Stochastic Optimizer: Maximum iterations (1000) reached and
```

```
the optimization hasn't converged yet.
  warnings.warn(
/projects/proteinml/.links/miniconda3/envs/aidep/lib/python3.10/site-
packages/sklearn/neural_network/_multilayer_perceptron.py:690:
ConvergenceWarning: Stochastic Optimizer: Maximum iterations (1000) reached and
the optimization hasn't converged yet.
  warnings.warn(
/projects/proteinml/.links/miniconda3/envs/aidep/lib/python3.10/site-
packages/sklearn/neural_network/_multilayer_perceptron.py:690:
ConvergenceWarning: Stochastic Optimizer: Maximum iterations (1000) reached and
the optimization hasn't converged yet.
  warnings.warn(
/projects/proteinml/.links/miniconda3/envs/aidep/lib/python3.10/site-
packages/sklearn/neural_network/_multilayer_perceptron.py:690:
ConvergenceWarning: Stochastic Optimizer: Maximum iterations (1000) reached and
the optimization hasn't converged yet.
  warnings.warn(
/projects/proteinml/.links/miniconda3/envs/aidep/lib/python3.10/site-
packages/sklearn/neural_network/_multilayer_perceptron.py:690:
ConvergenceWarning: Stochastic Optimizer: Maximum iterations (1000) reached and
the optimization hasn't converged yet.
  warnings.warn(
/projects/proteinml/.links/miniconda3/envs/aidep/lib/python3.10/site-
packages/sklearn/neural_network/_multilayer_perceptron.py:690:
ConvergenceWarning: Stochastic Optimizer: Maximum iterations (1000) reached and
the optimization hasn't converged yet.
  warnings.warn(
/projects/proteinml/.links/miniconda3/envs/aidep/lib/python3.10/site-
packages/sklearn/neural_network/_multilayer_perceptron.py:690:
ConvergenceWarning: Stochastic Optimizer: Maximum iterations (1000) reached and
the optimization hasn't converged yet.
  warnings.warn(
/projects/proteinml/.links/miniconda3/envs/aidep/lib/python3.10/site-
packages/sklearn/neural_network/_multilayer_perceptron.py:690:
ConvergenceWarning: Stochastic Optimizer: Maximum iterations (1000) reached and
the optimization hasn't converged yet.
  warnings.warn(
/projects/proteinml/.links/miniconda3/envs/aidep/lib/python3.10/site-
packages/sklearn/neural_network/_multilayer_perceptron.py:690:
ConvergenceWarning: Stochastic Optimizer: Maximum iterations (1000) reached and
the optimization hasn't converged yet.
  warnings.warn(
/projects/proteinml/.links/miniconda3/envs/aidep/lib/python3.10/site-
packages/sklearn/neural_network/_multilayer_perceptron.py:690:
ConvergenceWarning: Stochastic Optimizer: Maximum iterations (1000) reached and
the optimization hasn't converged yet.
  warnings.warn(
/projects/proteinml/.links/miniconda3/envs/aidep/lib/python3.10/site-
```

```
packages/sklearn/neural_network/_multilayer_perceptron.py:690:
ConvergenceWarning: Stochastic Optimizer: Maximum iterations (1000) reached and
the optimization hasn't converged yet.
  warnings.warn(
/projects/proteinml/.links/miniconda3/envs/aidep/lib/python3.10/site-
packages/sklearn/neural_network/_multilayer_perceptron.py:690:
ConvergenceWarning: Stochastic Optimizer: Maximum iterations (1000) reached and
the optimization hasn't converged yet.
  warnings.warn(
/projects/proteinml/.links/miniconda3/envs/aidep/lib/python3.10/site-
packages/sklearn/neural_network/_multilayer_perceptron.py:690:
ConvergenceWarning: Stochastic Optimizer: Maximum iterations (1000) reached and
the optimization hasn't converged yet.
  warnings.warn(
/projects/proteinml/.links/miniconda3/envs/aidep/lib/python3.10/site-
packages/sklearn/neural_network/_multilayer_perceptron.py:690:
ConvergenceWarning: Stochastic Optimizer: Maximum iterations (1000) reached and
the optimization hasn't converged yet.
  warnings.warn(
/projects/proteinml/.links/miniconda3/envs/aidep/lib/python3.10/site-
packages/sklearn/neural_network/_multilayer_perceptron.py:690:
ConvergenceWarning: Stochastic Optimizer: Maximum iterations (1000) reached and
the optimization hasn't converged yet.
  warnings.warn(
/projects/proteinml/.links/miniconda3/envs/aidep/lib/python3.10/site-
packages/sklearn/neural_network/_multilayer_perceptron.py:690:
ConvergenceWarning: Stochastic Optimizer: Maximum iterations (1000) reached and
the optimization hasn't converged yet.
  warnings.warn(
/projects/proteinml/.links/miniconda3/envs/aidep/lib/python3.10/site-
packages/sklearn/neural_network/_multilayer_perceptron.py:690:
ConvergenceWarning: Stochastic Optimizer: Maximum iterations (1000) reached and
the optimization hasn't converged yet.
  warnings.warn(
/projects/proteinml/.links/miniconda3/envs/aidep/lib/python3.10/site-
packages/sklearn/neural_network/_multilayer_perceptron.py:690:
ConvergenceWarning: Stochastic Optimizer: Maximum iterations (1000) reached and
the optimization hasn't converged yet.
  warnings.warn(
/projects/proteinml/.links/miniconda3/envs/aidep/lib/python3.10/site-
packages/sklearn/neural_network/_multilayer_perceptron.py:690:
ConvergenceWarning: Stochastic Optimizer: Maximum iterations (1000) reached and
the optimization hasn't converged yet.
  warnings.warn(
/projects/proteinml/.links/miniconda3/envs/aidep/lib/python3.10/site-
packages/sklearn/neural_network/_multilayer_perceptron.py:690:
ConvergenceWarning: Stochastic Optimizer: Maximum iterations (1000) reached and
the optimization hasn't converged yet.
```

```
  warnings.warn(
/projects/proteinml/.links/miniconda3/envs/aidep/lib/python3.10/site-
packages/sklearn/neural_network/_multilayer_perceptron.py:690:
ConvergenceWarning: Stochastic Optimizer: Maximum iterations (1000) reached and
the optimization hasn't converged yet.
  warnings.warn(
/projects/proteinml/.links/miniconda3/envs/aidep/lib/python3.10/site-
packages/sklearn/neural_network/_multilayer_perceptron.py:690:
ConvergenceWarning: Stochastic Optimizer: Maximum iterations (1000) reached and
the optimization hasn't converged yet.
  warnings.warn(
/projects/proteinml/.links/miniconda3/envs/aidep/lib/python3.10/site-
packages/sklearn/neural_network/_multilayer_perceptron.py:690:
ConvergenceWarning: Stochastic Optimizer: Maximum iterations (1000) reached and
the optimization hasn't converged yet.
  warnings.warn(
/projects/proteinml/.links/miniconda3/envs/aidep/lib/python3.10/site-
packages/sklearn/neural_network/_multilayer_perceptron.py:690:
ConvergenceWarning: Stochastic Optimizer: Maximum iterations (1000) reached and
the optimization hasn't converged yet.
  warnings.warn(
/projects/proteinml/.links/miniconda3/envs/aidep/lib/python3.10/site-
packages/sklearn/neural_network/_multilayer_perceptron.py:690:
ConvergenceWarning: Stochastic Optimizer: Maximum iterations (1000) reached and
the optimization hasn't converged yet.
  warnings.warn(
/projects/proteinml/.links/miniconda3/envs/aidep/lib/python3.10/site-
packages/sklearn/neural_network/_multilayer_perceptron.py:690:
ConvergenceWarning: Stochastic Optimizer: Maximum iterations (1000) reached and
the optimization hasn't converged yet.
  warnings.warn(
/projects/proteinml/.links/miniconda3/envs/aidep/lib/python3.10/site-
packages/sklearn/neural_network/_multilayer_perceptron.py:690:
ConvergenceWarning: Stochastic Optimizer: Maximum iterations (1000) reached and
the optimization hasn't converged yet.
  warnings.warn(
/projects/proteinml/.links/miniconda3/envs/aidep/lib/python3.10/site-
packages/sklearn/neural_network/_multilayer_perceptron.py:690:
ConvergenceWarning: Stochastic Optimizer: Maximum iterations (1000) reached and
the optimization hasn't converged yet.
  warnings.warn(
/projects/proteinml/.links/miniconda3/envs/aidep/lib/python3.10/site-
packages/sklearn/neural_network/_multilayer_perceptron.py:690:
ConvergenceWarning: Stochastic Optimizer: Maximum iterations (1000) reached and
the optimization hasn't converged yet.
  warnings.warn(
/projects/proteinml/.links/miniconda3/envs/aidep/lib/python3.10/site-
packages/sklearn/neural_network/_multilayer_perceptron.py:690:
```

```
ConvergenceWarning: Stochastic Optimizer: Maximum iterations (1000) reached and
the optimization hasn't converged yet.
  warnings.warn(
/projects/proteinml/.links/miniconda3/envs/aidep/lib/python3.10/site-
packages/sklearn/neural_network/_multilayer_perceptron.py:690:
ConvergenceWarning: Stochastic Optimizer: Maximum iterations (1000) reached and
the optimization hasn't converged yet.
  warnings.warn(
/projects/proteinml/.links/miniconda3/envs/aidep/lib/python3.10/site-
packages/sklearn/neural_network/_multilayer_perceptron.py:690:
ConvergenceWarning: Stochastic Optimizer: Maximum iterations (1000) reached and
the optimization hasn't converged yet.
  warnings.warn(
/projects/proteinml/.links/miniconda3/envs/aidep/lib/python3.10/site-
packages/sklearn/neural_network/_multilayer_perceptron.py:690:
ConvergenceWarning: Stochastic Optimizer: Maximum iterations (1000) reached and
the optimization hasn't converged yet.
  warnings.warn(
/projects/proteinml/.links/miniconda3/envs/aidep/lib/python3.10/site-
packages/sklearn/neural_network/_multilayer_perceptron.py:690:
ConvergenceWarning: Stochastic Optimizer: Maximum iterations (1000) reached and
the optimization hasn't converged yet.
  warnings.warn(
/projects/proteinml/.links/miniconda3/envs/aidep/lib/python3.10/site-
packages/sklearn/neural_network/_multilayer_perceptron.py:690:
ConvergenceWarning: Stochastic Optimizer: Maximum iterations (1000) reached and
the optimization hasn't converged yet.
  warnings.warn(
/projects/proteinml/.links/miniconda3/envs/aidep/lib/python3.10/site-
packages/sklearn/neural_network/_multilayer_perceptron.py:690:
ConvergenceWarning: Stochastic Optimizer: Maximum iterations (1000) reached and
the optimization hasn't converged yet.
  warnings.warn(
/projects/proteinml/.links/miniconda3/envs/aidep/lib/python3.10/site-
packages/sklearn/neural_network/_multilayer_perceptron.py:690:
ConvergenceWarning: Stochastic Optimizer: Maximum iterations (1000) reached and
the optimization hasn't converged yet.
  warnings.warn(
/projects/proteinml/.links/miniconda3/envs/aidep/lib/python3.10/site-
packages/sklearn/neural_network/_multilayer_perceptron.py:690:
ConvergenceWarning: Stochastic Optimizer: Maximum iterations (1000) reached and
the optimization hasn't converged yet.
  warnings.warn(
/projects/proteinml/.links/miniconda3/envs/aidep/lib/python3.10/site-
packages/sklearn/neural_network/_multilayer_perceptron.py:690:
ConvergenceWarning: Stochastic Optimizer: Maximum iterations (1000) reached and
the optimization hasn't converged yet.
  warnings.warn(
```

```
/projects/proteinml/.links/miniconda3/envs/aidep/lib/python3.10/site-
packages/sklearn/neural_network/_multilayer_perceptron.py:690:
ConvergenceWarning: Stochastic Optimizer: Maximum iterations (1000) reached and
the optimization hasn't converged yet.
  warnings.warn(
/projects/proteinml/.links/miniconda3/envs/aidep/lib/python3.10/site-
packages/sklearn/neural_network/_multilayer_perceptron.py:690:
ConvergenceWarning: Stochastic Optimizer: Maximum iterations (1000) reached and
the optimization hasn't converged yet.
  warnings.warn(
/projects/proteinml/.links/miniconda3/envs/aidep/lib/python3.10/site-
packages/sklearn/neural_network/_multilayer_perceptron.py:690:
ConvergenceWarning: Stochastic Optimizer: Maximum iterations (1000) reached and
the optimization hasn't converged yet.
  warnings.warn(
/projects/proteinml/.links/miniconda3/envs/aidep/lib/python3.10/site-
packages/sklearn/neural_network/_multilayer_perceptron.py:690:
ConvergenceWarning: Stochastic Optimizer: Maximum iterations (1000) reached and
the optimization hasn't converged yet.
  warnings.warn(
/projects/proteinml/.links/miniconda3/envs/aidep/lib/python3.10/site-
packages/sklearn/neural_network/_multilayer_perceptron.py:690:
ConvergenceWarning: Stochastic Optimizer: Maximum iterations (1000) reached and
the optimization hasn't converged yet.
  warnings.warn(
/projects/proteinml/.links/miniconda3/envs/aidep/lib/python3.10/site-
packages/sklearn/neural_network/_multilayer_perceptron.py:690:
ConvergenceWarning: Stochastic Optimizer: Maximum iterations (1000) reached and
the optimization hasn't converged yet.
  warnings.warn(
/projects/proteinml/.links/miniconda3/envs/aidep/lib/python3.10/site-
packages/sklearn/neural_network/_multilayer_perceptron.py:690:
ConvergenceWarning: Stochastic Optimizer: Maximum iterations (1000) reached and
the optimization hasn't converged yet.
  warnings.warn(
/projects/proteinml/.links/miniconda3/envs/aidep/lib/python3.10/site-
packages/sklearn/neural_network/_multilayer_perceptron.py:690:
ConvergenceWarning: Stochastic Optimizer: Maximum iterations (1000) reached and
the optimization hasn't converged yet.
  warnings.warn(
/projects/proteinml/.links/miniconda3/envs/aidep/lib/python3.10/site-
packages/sklearn/neural_network/_multilayer_perceptron.py:690:
ConvergenceWarning: Stochastic Optimizer: Maximum iterations (1000) reached and
the optimization hasn't converged yet.
  warnings.warn(
/projects/proteinml/.links/miniconda3/envs/aidep/lib/python3.10/site-
packages/sklearn/neural_network/_multilayer_perceptron.py:690:
ConvergenceWarning: Stochastic Optimizer: Maximum iterations (1000) reached and
```

```
the optimization hasn't converged yet.
  warnings.warn(
/projects/proteinml/.links/miniconda3/envs/aidep/lib/python3.10/site-
packages/sklearn/neural_network/_multilayer_perceptron.py:690:
ConvergenceWarning: Stochastic Optimizer: Maximum iterations (1000) reached and
the optimization hasn't converged yet.
  warnings.warn(
/projects/proteinml/.links/miniconda3/envs/aidep/lib/python3.10/site-
packages/sklearn/neural_network/_multilayer_perceptron.py:690:
ConvergenceWarning: Stochastic Optimizer: Maximum iterations (1000) reached and
the optimization hasn't converged yet.
  warnings.warn(
/projects/proteinml/.links/miniconda3/envs/aidep/lib/python3.10/site-
packages/sklearn/neural_network/_multilayer_perceptron.py:690:
ConvergenceWarning: Stochastic Optimizer: Maximum iterations (1000) reached and
the optimization hasn't converged yet.
  warnings.warn(
/projects/proteinml/.links/miniconda3/envs/aidep/lib/python3.10/site-
packages/sklearn/neural_network/_multilayer_perceptron.py:690:
ConvergenceWarning: Stochastic Optimizer: Maximum iterations (1000) reached and
the optimization hasn't converged yet.
  warnings.warn(
/projects/proteinml/.links/miniconda3/envs/aidep/lib/python3.10/site-
packages/sklearn/neural_network/_multilayer_perceptron.py:690:
ConvergenceWarning: Stochastic Optimizer: Maximum iterations (1000) reached and
the optimization hasn't converged yet.
  warnings.warn(
/projects/proteinml/.links/miniconda3/envs/aidep/lib/python3.10/site-
packages/sklearn/neural_network/_multilayer_perceptron.py:690:
ConvergenceWarning: Stochastic Optimizer: Maximum iterations (1000) reached and
the optimization hasn't converged yet.
  warnings.warn(
/projects/proteinml/.links/miniconda3/envs/aidep/lib/python3.10/site-
packages/sklearn/neural_network/_multilayer_perceptron.py:690:
ConvergenceWarning: Stochastic Optimizer: Maximum iterations (1000) reached and
the optimization hasn't converged yet.
  warnings.warn(
/projects/proteinml/.links/miniconda3/envs/aidep/lib/python3.10/site-
packages/sklearn/neural_network/_multilayer_perceptron.py:690:
ConvergenceWarning: Stochastic Optimizer: Maximum iterations (1000) reached and
the optimization hasn't converged yet.
  warnings.warn(
/projects/proteinml/.links/miniconda3/envs/aidep/lib/python3.10/site-
packages/sklearn/neural_network/_multilayer_perceptron.py:690:
ConvergenceWarning: Stochastic Optimizer: Maximum iterations (1000) reached and
the optimization hasn't converged yet.
  warnings.warn(
/projects/proteinml/.links/miniconda3/envs/aidep/lib/python3.10/site-
```

```
packages/sklearn/neural_network/_multilayer_perceptron.py:690:
ConvergenceWarning: Stochastic Optimizer: Maximum iterations (1000) reached and
the optimization hasn't converged yet.
  warnings.warn(
/projects/proteinml/.links/miniconda3/envs/aidep/lib/python3.10/site-
packages/sklearn/neural_network/_multilayer_perceptron.py:690:
ConvergenceWarning: Stochastic Optimizer: Maximum iterations (1000) reached and
the optimization hasn't converged yet.
  warnings.warn(
/projects/proteinml/.links/miniconda3/envs/aidep/lib/python3.10/site-
packages/sklearn/neural_network/_multilayer_perceptron.py:690:
ConvergenceWarning: Stochastic Optimizer: Maximum iterations (1000) reached and
the optimization hasn't converged yet.
  warnings.warn(
/projects/proteinml/.links/miniconda3/envs/aidep/lib/python3.10/site-
packages/sklearn/neural_network/_multilayer_perceptron.py:690:
ConvergenceWarning: Stochastic Optimizer: Maximum iterations (1000) reached and
the optimization hasn't converged yet.
  warnings.warn(
/projects/proteinml/.links/miniconda3/envs/aidep/lib/python3.10/site-
packages/sklearn/neural_network/_multilayer_perceptron.py:690:
ConvergenceWarning: Stochastic Optimizer: Maximum iterations (1000) reached and
the optimization hasn't converged yet.
  warnings.warn(
/projects/proteinml/.links/miniconda3/envs/aidep/lib/python3.10/site-
packages/sklearn/neural_network/_multilayer_perceptron.py:690:
ConvergenceWarning: Stochastic Optimizer: Maximum iterations (1000) reached and
the optimization hasn't converged yet.
  warnings.warn(
/projects/proteinml/.links/miniconda3/envs/aidep/lib/python3.10/site-
packages/sklearn/neural_network/_multilayer_perceptron.py:690:
ConvergenceWarning: Stochastic Optimizer: Maximum iterations (1000) reached and
the optimization hasn't converged yet.
  warnings.warn(
/projects/proteinml/.links/miniconda3/envs/aidep/lib/python3.10/site-
packages/sklearn/neural_network/_multilayer_perceptron.py:690:
ConvergenceWarning: Stochastic Optimizer: Maximum iterations (1000) reached and
the optimization hasn't converged yet.
  warnings.warn(
/projects/proteinml/.links/miniconda3/envs/aidep/lib/python3.10/site-
packages/sklearn/neural_network/_multilayer_perceptron.py:690:
ConvergenceWarning: Stochastic Optimizer: Maximum iterations (1000) reached and
the optimization hasn't converged yet.
  warnings.warn(
/projects/proteinml/.links/miniconda3/envs/aidep/lib/python3.10/site-
packages/sklearn/neural_network/_multilayer_perceptron.py:690:
ConvergenceWarning: Stochastic Optimizer: Maximum iterations (1000) reached and
the optimization hasn't converged yet.
```

```
  warnings.warn(
/projects/proteinml/.links/miniconda3/envs/aidep/lib/python3.10/site-
packages/sklearn/neural_network/_multilayer_perceptron.py:690:
ConvergenceWarning: Stochastic Optimizer: Maximum iterations (1000) reached and
the optimization hasn't converged yet.
  warnings.warn(
/projects/proteinml/.links/miniconda3/envs/aidep/lib/python3.10/site-
packages/sklearn/neural_network/_multilayer_perceptron.py:690:
ConvergenceWarning: Stochastic Optimizer: Maximum iterations (1000) reached and
the optimization hasn't converged yet.
  warnings.warn(
/projects/proteinml/.links/miniconda3/envs/aidep/lib/python3.10/site-
packages/sklearn/neural_network/_multilayer_perceptron.py:690:
ConvergenceWarning: Stochastic Optimizer: Maximum iterations (1000) reached and
the optimization hasn't converged yet.
  warnings.warn(
/projects/proteinml/.links/miniconda3/envs/aidep/lib/python3.10/site-
packages/sklearn/neural_network/_multilayer_perceptron.py:690:
ConvergenceWarning: Stochastic Optimizer: Maximum iterations (1000) reached and
the optimization hasn't converged yet.
  warnings.warn(
/projects/proteinml/.links/miniconda3/envs/aidep/lib/python3.10/site-
packages/sklearn/neural_network/_multilayer_perceptron.py:690:
ConvergenceWarning: Stochastic Optimizer: Maximum iterations (1000) reached and
the optimization hasn't converged yet.
  warnings.warn(
/projects/proteinml/.links/miniconda3/envs/aidep/lib/python3.10/site-
packages/sklearn/neural_network/_multilayer_perceptron.py:690:
ConvergenceWarning: Stochastic Optimizer: Maximum iterations (1000) reached and
the optimization hasn't converged yet.
  warnings.warn(
/projects/proteinml/.links/miniconda3/envs/aidep/lib/python3.10/site-
packages/sklearn/neural_network/_multilayer_perceptron.py:690:
ConvergenceWarning: Stochastic Optimizer: Maximum iterations (1000) reached and
the optimization hasn't converged yet.
  warnings.warn(
/projects/proteinml/.links/miniconda3/envs/aidep/lib/python3.10/site-
packages/sklearn/neural_network/_multilayer_perceptron.py:690:
ConvergenceWarning: Stochastic Optimizer: Maximum iterations (1000) reached and
the optimization hasn't converged yet.
  warnings.warn(
/projects/proteinml/.links/miniconda3/envs/aidep/lib/python3.10/site-
packages/sklearn/neural_network/_multilayer_perceptron.py:690:
ConvergenceWarning: Stochastic Optimizer: Maximum iterations (1000) reached and
the optimization hasn't converged yet.
  warnings.warn(
/projects/proteinml/.links/miniconda3/envs/aidep/lib/python3.10/site-
packages/sklearn/neural_network/_multilayer_perceptron.py:690:
```

```
ConvergenceWarning: Stochastic Optimizer: Maximum iterations (1000) reached and
the optimization hasn't converged yet.
  warnings.warn(
/projects/proteinml/.links/miniconda3/envs/aidep/lib/python3.10/site-
packages/sklearn/neural_network/_multilayer_perceptron.py:690:
ConvergenceWarning: Stochastic Optimizer: Maximum iterations (1000) reached and
the optimization hasn't converged yet.
  warnings.warn(
/projects/proteinml/.links/miniconda3/envs/aidep/lib/python3.10/site-
packages/sklearn/neural_network/_multilayer_perceptron.py:690:
ConvergenceWarning: Stochastic Optimizer: Maximum iterations (1000) reached and
the optimization hasn't converged yet.
  warnings.warn(
/projects/proteinml/.links/miniconda3/envs/aidep/lib/python3.10/site-
packages/sklearn/neural_network/_multilayer_perceptron.py:690:
ConvergenceWarning: Stochastic Optimizer: Maximum iterations (1000) reached and
the optimization hasn't converged yet.
  warnings.warn(
/projects/proteinml/.links/miniconda3/envs/aidep/lib/python3.10/site-
packages/sklearn/neural_network/_multilayer_perceptron.py:690:
ConvergenceWarning: Stochastic Optimizer: Maximum iterations (1000) reached and
the optimization hasn't converged yet.
  warnings.warn(
/projects/proteinml/.links/miniconda3/envs/aidep/lib/python3.10/site-
packages/sklearn/neural_network/_multilayer_perceptron.py:690:
ConvergenceWarning: Stochastic Optimizer: Maximum iterations (1000) reached and
the optimization hasn't converged yet.
  warnings.warn(
/projects/proteinml/.links/miniconda3/envs/aidep/lib/python3.10/site-
packages/sklearn/neural_network/_multilayer_perceptron.py:690:
ConvergenceWarning: Stochastic Optimizer: Maximum iterations (1000) reached and
the optimization hasn't converged yet.
  warnings.warn(
/projects/proteinml/.links/miniconda3/envs/aidep/lib/python3.10/site-
packages/sklearn/neural_network/_multilayer_perceptron.py:690:
ConvergenceWarning: Stochastic Optimizer: Maximum iterations (1000) reached and
the optimization hasn't converged yet.
  warnings.warn(
/projects/proteinml/.links/miniconda3/envs/aidep/lib/python3.10/site-
packages/sklearn/neural_network/_multilayer_perceptron.py:690:
ConvergenceWarning: Stochastic Optimizer: Maximum iterations (1000) reached and
the optimization hasn't converged yet.
  warnings.warn(
/projects/proteinml/.links/miniconda3/envs/aidep/lib/python3.10/site-
packages/sklearn/neural_network/_multilayer_perceptron.py:690:
ConvergenceWarning: Stochastic Optimizer: Maximum iterations (1000) reached and
the optimization hasn't converged yet.
  warnings.warn(
```

```
/projects/proteinml/.links/miniconda3/envs/aidep/lib/python3.10/site-
packages/sklearn/neural_network/_multilayer_perceptron.py:690:
ConvergenceWarning: Stochastic Optimizer: Maximum iterations (1000) reached and
the optimization hasn't converged yet.
  warnings.warn(
/projects/proteinml/.links/miniconda3/envs/aidep/lib/python3.10/site-
packages/sklearn/neural_network/_multilayer_perceptron.py:690:
ConvergenceWarning: Stochastic Optimizer: Maximum iterations (1000) reached and
the optimization hasn't converged yet.
  warnings.warn(
/projects/proteinml/.links/miniconda3/envs/aidep/lib/python3.10/site-
packages/sklearn/neural_network/_multilayer_perceptron.py:690:
ConvergenceWarning: Stochastic Optimizer: Maximum iterations (1000) reached and
the optimization hasn't converged yet.
  warnings.warn(
/projects/proteinml/.links/miniconda3/envs/aidep/lib/python3.10/site-
packages/sklearn/neural_network/_multilayer_perceptron.py:690:
ConvergenceWarning: Stochastic Optimizer: Maximum iterations (1000) reached and
the optimization hasn't converged yet.
  warnings.warn(
/projects/proteinml/.links/miniconda3/envs/aidep/lib/python3.10/site-
packages/sklearn/neural_network/_multilayer_perceptron.py:690:
ConvergenceWarning: Stochastic Optimizer: Maximum iterations (1000) reached and
the optimization hasn't converged yet.
  warnings.warn(
/projects/proteinml/.links/miniconda3/envs/aidep/lib/python3.10/site-
packages/sklearn/neural_network/_multilayer_perceptron.py:690:
ConvergenceWarning: Stochastic Optimizer: Maximum iterations (1000) reached and
the optimization hasn't converged yet.
  warnings.warn(
/projects/proteinml/.links/miniconda3/envs/aidep/lib/python3.10/site-
packages/sklearn/neural_network/_multilayer_perceptron.py:690:
ConvergenceWarning: Stochastic Optimizer: Maximum iterations (1000) reached and
the optimization hasn't converged yet.
  warnings.warn(
/projects/proteinml/.links/miniconda3/envs/aidep/lib/python3.10/site-
packages/sklearn/neural_network/_multilayer_perceptron.py:690:
ConvergenceWarning: Stochastic Optimizer: Maximum iterations (1000) reached and
the optimization hasn't converged yet.
  warnings.warn(
/projects/proteinml/.links/miniconda3/envs/aidep/lib/python3.10/site-
packages/sklearn/neural_network/_multilayer_perceptron.py:690:
ConvergenceWarning: Stochastic Optimizer: Maximum iterations (1000) reached and
the optimization hasn't converged yet.
  warnings.warn(
/projects/proteinml/.links/miniconda3/envs/aidep/lib/python3.10/site-
packages/sklearn/neural_network/_multilayer_perceptron.py:690:
ConvergenceWarning: Stochastic Optimizer: Maximum iterations (1000) reached and
```

```
the optimization hasn't converged yet.
  warnings.warn(
/projects/proteinml/.links/miniconda3/envs/aidep/lib/python3.10/site-
packages/sklearn/neural_network/_multilayer_perceptron.py:690:
ConvergenceWarning: Stochastic Optimizer: Maximum iterations (1000) reached and
the optimization hasn't converged yet.
  warnings.warn(
/projects/proteinml/.links/miniconda3/envs/aidep/lib/python3.10/site-
packages/sklearn/neural_network/_multilayer_perceptron.py:690:
ConvergenceWarning: Stochastic Optimizer: Maximum iterations (1000) reached and
the optimization hasn't converged yet.
  warnings.warn(
/projects/proteinml/.links/miniconda3/envs/aidep/lib/python3.10/site-
packages/sklearn/neural_network/_multilayer_perceptron.py:690:
ConvergenceWarning: Stochastic Optimizer: Maximum iterations (1000) reached and
the optimization hasn't converged yet.
  warnings.warn(
/projects/proteinml/.links/miniconda3/envs/aidep/lib/python3.10/site-
packages/sklearn/neural_network/_multilayer_perceptron.py:690:
ConvergenceWarning: Stochastic Optimizer: Maximum iterations (1000) reached and
the optimization hasn't converged yet.
  warnings.warn(
/projects/proteinml/.links/miniconda3/envs/aidep/lib/python3.10/site-
packages/sklearn/neural_network/_multilayer_perceptron.py:690:
ConvergenceWarning: Stochastic Optimizer: Maximum iterations (1000) reached and
the optimization hasn't converged yet.
  warnings.warn(
/projects/proteinml/.links/miniconda3/envs/aidep/lib/python3.10/site-
packages/sklearn/neural_network/_multilayer_perceptron.py:690:
ConvergenceWarning: Stochastic Optimizer: Maximum iterations (1000) reached and
the optimization hasn't converged yet.
  warnings.warn(
/projects/proteinml/.links/miniconda3/envs/aidep/lib/python3.10/site-
packages/sklearn/neural_network/_multilayer_perceptron.py:690:
ConvergenceWarning: Stochastic Optimizer: Maximum iterations (1000) reached and
the optimization hasn't converged yet.
  warnings.warn(
/projects/proteinml/.links/miniconda3/envs/aidep/lib/python3.10/site-
packages/sklearn/neural_network/_multilayer_perceptron.py:690:
ConvergenceWarning: Stochastic Optimizer: Maximum iterations (1000) reached and
the optimization hasn't converged yet.
  warnings.warn(
/projects/proteinml/.links/miniconda3/envs/aidep/lib/python3.10/site-
packages/sklearn/neural_network/_multilayer_perceptron.py:690:
ConvergenceWarning: Stochastic Optimizer: Maximum iterations (1000) reached and
the optimization hasn't converged yet.
  warnings.warn(
/projects/proteinml/.links/miniconda3/envs/aidep/lib/python3.10/site-
```

```
packages/sklearn/neural_network/_multilayer_perceptron.py:690:
ConvergenceWarning: Stochastic Optimizer: Maximum iterations (1000) reached and
the optimization hasn't converged yet.
  warnings.warn(
/projects/proteinml/.links/miniconda3/envs/aidep/lib/python3.10/site-
packages/sklearn/neural_network/_multilayer_perceptron.py:690:
ConvergenceWarning: Stochastic Optimizer: Maximum iterations (1000) reached and
the optimization hasn't converged yet.
  warnings.warn(
/projects/proteinml/.links/miniconda3/envs/aidep/lib/python3.10/site-
packages/sklearn/neural_network/_multilayer_perceptron.py:690:
ConvergenceWarning: Stochastic Optimizer: Maximum iterations (1000) reached and
the optimization hasn't converged yet.
  warnings.warn(
/projects/proteinml/.links/miniconda3/envs/aidep/lib/python3.10/site-
packages/sklearn/neural_network/_multilayer_perceptron.py:690:
ConvergenceWarning: Stochastic Optimizer: Maximum iterations (1000) reached and
the optimization hasn't converged yet.
  warnings.warn(
/projects/proteinml/.links/miniconda3/envs/aidep/lib/python3.10/site-
packages/sklearn/neural_network/_multilayer_perceptron.py:690:
ConvergenceWarning: Stochastic Optimizer: Maximum iterations (1000) reached and
the optimization hasn't converged yet.
  warnings.warn(
/projects/proteinml/.links/miniconda3/envs/aidep/lib/python3.10/site-
packages/sklearn/neural_network/_multilayer_perceptron.py:690:
ConvergenceWarning: Stochastic Optimizer: Maximum iterations (1000) reached and
the optimization hasn't converged yet.
  warnings.warn(
/projects/proteinml/.links/miniconda3/envs/aidep/lib/python3.10/site-
packages/sklearn/neural_network/_multilayer_perceptron.py:690:
ConvergenceWarning: Stochastic Optimizer: Maximum iterations (1000) reached and
the optimization hasn't converged yet.
  warnings.warn(
/projects/proteinml/.links/miniconda3/envs/aidep/lib/python3.10/site-
packages/sklearn/neural_network/_multilayer_perceptron.py:690:
ConvergenceWarning: Stochastic Optimizer: Maximum iterations (1000) reached and
the optimization hasn't converged yet.
  warnings.warn(
/projects/proteinml/.links/miniconda3/envs/aidep/lib/python3.10/site-
packages/sklearn/neural_network/_multilayer_perceptron.py:690:
ConvergenceWarning: Stochastic Optimizer: Maximum iterations (1000) reached and
the optimization hasn't converged yet.
  warnings.warn(
/projects/proteinml/.links/miniconda3/envs/aidep/lib/python3.10/site-
packages/sklearn/neural_network/_multilayer_perceptron.py:690:
ConvergenceWarning: Stochastic Optimizer: Maximum iterations (1000) reached and
the optimization hasn't converged yet.
```

```
  warnings.warn(
/projects/proteinml/.links/miniconda3/envs/aidep/lib/python3.10/site-
packages/sklearn/neural_network/_multilayer_perceptron.py:690:
ConvergenceWarning: Stochastic Optimizer: Maximum iterations (1000) reached and
the optimization hasn't converged yet.
  warnings.warn(
/projects/proteinml/.links/miniconda3/envs/aidep/lib/python3.10/site-
packages/sklearn/neural_network/_multilayer_perceptron.py:690:
ConvergenceWarning: Stochastic Optimizer: Maximum iterations (1000) reached and
the optimization hasn't converged yet.
  warnings.warn(
/projects/proteinml/.links/miniconda3/envs/aidep/lib/python3.10/site-
packages/sklearn/neural_network/_multilayer_perceptron.py:690:
ConvergenceWarning: Stochastic Optimizer: Maximum iterations (1000) reached and
the optimization hasn't converged yet.
  warnings.warn(
/projects/proteinml/.links/miniconda3/envs/aidep/lib/python3.10/site-
packages/sklearn/neural_network/_multilayer_perceptron.py:690:
ConvergenceWarning: Stochastic Optimizer: Maximum iterations (1000) reached and
the optimization hasn't converged yet.
  warnings.warn(
/projects/proteinml/.links/miniconda3/envs/aidep/lib/python3.10/site-
packages/sklearn/neural_network/_multilayer_perceptron.py:690:
ConvergenceWarning: Stochastic Optimizer: Maximum iterations (1000) reached and
the optimization hasn't converged yet.
  warnings.warn(
/projects/proteinml/.links/miniconda3/envs/aidep/lib/python3.10/site-
packages/sklearn/neural_network/_multilayer_perceptron.py:690:
ConvergenceWarning: Stochastic Optimizer: Maximum iterations (1000) reached and
the optimization hasn't converged yet.
  warnings.warn(
/projects/proteinml/.links/miniconda3/envs/aidep/lib/python3.10/site-
packages/sklearn/neural_network/_multilayer_perceptron.py:690:
ConvergenceWarning: Stochastic Optimizer: Maximum iterations (1000) reached and
the optimization hasn't converged yet.
  warnings.warn(
/projects/proteinml/.links/miniconda3/envs/aidep/lib/python3.10/site-
packages/sklearn/neural_network/_multilayer_perceptron.py:690:
ConvergenceWarning: Stochastic Optimizer: Maximum iterations (1000) reached and
the optimization hasn't converged yet.
  warnings.warn(
/projects/proteinml/.links/miniconda3/envs/aidep/lib/python3.10/site-
packages/sklearn/neural_network/_multilayer_perceptron.py:690:
ConvergenceWarning: Stochastic Optimizer: Maximum iterations (1000) reached and
the optimization hasn't converged yet.
  warnings.warn(
/projects/proteinml/.links/miniconda3/envs/aidep/lib/python3.10/site-
packages/sklearn/neural_network/_multilayer_perceptron.py:690:
```

```
ConvergenceWarning: Stochastic Optimizer: Maximum iterations (1000) reached and
the optimization hasn't converged yet.
  warnings.warn(
/projects/proteinml/.links/miniconda3/envs/aidep/lib/python3.10/site-
packages/sklearn/neural_network/_multilayer_perceptron.py:690:
ConvergenceWarning: Stochastic Optimizer: Maximum iterations (1000) reached and
the optimization hasn't converged yet.
  warnings.warn(
/projects/proteinml/.links/miniconda3/envs/aidep/lib/python3.10/site-
packages/sklearn/neural_network/_multilayer_perceptron.py:690:
ConvergenceWarning: Stochastic Optimizer: Maximum iterations (1000) reached and
the optimization hasn't converged yet.
  warnings.warn(
/projects/proteinml/.links/miniconda3/envs/aidep/lib/python3.10/site-
packages/sklearn/neural_network/_multilayer_perceptron.py:690:
ConvergenceWarning: Stochastic Optimizer: Maximum iterations (1000) reached and
the optimization hasn't converged yet.
  warnings.warn(
/projects/proteinml/.links/miniconda3/envs/aidep/lib/python3.10/site-
packages/sklearn/neural_network/_multilayer_perceptron.py:690:
ConvergenceWarning: Stochastic Optimizer: Maximum iterations (1000) reached and
the optimization hasn't converged yet.
  warnings.warn(
/projects/proteinml/.links/miniconda3/envs/aidep/lib/python3.10/site-
packages/sklearn/neural_network/_multilayer_perceptron.py:690:
ConvergenceWarning: Stochastic Optimizer: Maximum iterations (1000) reached and
the optimization hasn't converged yet.
  warnings.warn(
/projects/proteinml/.links/miniconda3/envs/aidep/lib/python3.10/site-
packages/sklearn/neural_network/_multilayer_perceptron.py:690:
ConvergenceWarning: Stochastic Optimizer: Maximum iterations (1000) reached and
the optimization hasn't converged yet.
  warnings.warn(
/projects/proteinml/.links/miniconda3/envs/aidep/lib/python3.10/site-
packages/sklearn/neural_network/_multilayer_perceptron.py:690:
ConvergenceWarning: Stochastic Optimizer: Maximum iterations (1000) reached and
the optimization hasn't converged yet.
  warnings.warn(
/projects/proteinml/.links/miniconda3/envs/aidep/lib/python3.10/site-
packages/sklearn/neural_network/_multilayer_perceptron.py:690:
ConvergenceWarning: Stochastic Optimizer: Maximum iterations (1000) reached and
the optimization hasn't converged yet.
  warnings.warn(
/projects/proteinml/.links/miniconda3/envs/aidep/lib/python3.10/site-
packages/sklearn/neural_network/_multilayer_perceptron.py:690:
ConvergenceWarning: Stochastic Optimizer: Maximum iterations (1000) reached and
the optimization hasn't converged yet.
  warnings.warn(
```

```
/projects/proteinml/.links/miniconda3/envs/aidep/lib/python3.10/site-
packages/sklearn/neural_network/_multilayer_perceptron.py:690:
ConvergenceWarning: Stochastic Optimizer: Maximum iterations (1000) reached and
the optimization hasn't converged yet.
  warnings.warn(
/projects/proteinml/.links/miniconda3/envs/aidep/lib/python3.10/site-
packages/sklearn/neural_network/_multilayer_perceptron.py:690:
ConvergenceWarning: Stochastic Optimizer: Maximum iterations (1000) reached and
the optimization hasn't converged yet.
  warnings.warn(
/projects/proteinml/.links/miniconda3/envs/aidep/lib/python3.10/site-
packages/sklearn/neural_network/_multilayer_perceptron.py:690:
ConvergenceWarning: Stochastic Optimizer: Maximum iterations (1000) reached and
the optimization hasn't converged yet.
  warnings.warn(
/projects/proteinml/.links/miniconda3/envs/aidep/lib/python3.10/site-
packages/sklearn/neural_network/_multilayer_perceptron.py:690:
ConvergenceWarning: Stochastic Optimizer: Maximum iterations (1000) reached and
the optimization hasn't converged yet.
  warnings.warn(
/projects/proteinml/.links/miniconda3/envs/aidep/lib/python3.10/site-
packages/sklearn/neural_network/_multilayer_perceptron.py:690:
ConvergenceWarning: Stochastic Optimizer: Maximum iterations (1000) reached and
the optimization hasn't converged yet.
  warnings.warn(
/projects/proteinml/.links/miniconda3/envs/aidep/lib/python3.10/site-
packages/sklearn/neural_network/_multilayer_perceptron.py:690:
ConvergenceWarning: Stochastic Optimizer: Maximum iterations (1000) reached and
the optimization hasn't converged yet.
  warnings.warn(
/projects/proteinml/.links/miniconda3/envs/aidep/lib/python3.10/site-
packages/sklearn/neural_network/_multilayer_perceptron.py:690:
ConvergenceWarning: Stochastic Optimizer: Maximum iterations (1000) reached and
the optimization hasn't converged yet.
  warnings.warn(
/projects/proteinml/.links/miniconda3/envs/aidep/lib/python3.10/site-
packages/sklearn/neural_network/_multilayer_perceptron.py:690:
ConvergenceWarning: Stochastic Optimizer: Maximum iterations (1000) reached and
the optimization hasn't converged yet.
  warnings.warn(
/projects/proteinml/.links/miniconda3/envs/aidep/lib/python3.10/site-
packages/sklearn/neural_network/_multilayer_perceptron.py:690:
ConvergenceWarning: Stochastic Optimizer: Maximum iterations (1000) reached and
the optimization hasn't converged yet.
  warnings.warn(
/projects/proteinml/.links/miniconda3/envs/aidep/lib/python3.10/site-
packages/sklearn/neural_network/_multilayer_perceptron.py:690:
ConvergenceWarning: Stochastic Optimizer: Maximum iterations (1000) reached and
```

```
the optimization hasn't converged yet.
  warnings.warn(
/projects/proteinml/.links/miniconda3/envs/aidep/lib/python3.10/site-
packages/sklearn/neural_network/_multilayer_perceptron.py:690:
ConvergenceWarning: Stochastic Optimizer: Maximum iterations (1000) reached and
the optimization hasn't converged yet.
  warnings.warn(
/projects/proteinml/.links/miniconda3/envs/aidep/lib/python3.10/site-
packages/sklearn/neural_network/_multilayer_perceptron.py:690:
ConvergenceWarning: Stochastic Optimizer: Maximum iterations (1000) reached and
the optimization hasn't converged yet.
  warnings.warn(
/projects/proteinml/.links/miniconda3/envs/aidep/lib/python3.10/site-
packages/sklearn/neural_network/_multilayer_perceptron.py:690:
ConvergenceWarning: Stochastic Optimizer: Maximum iterations (1000) reached and
the optimization hasn't converged yet.
  warnings.warn(
/projects/proteinml/.links/miniconda3/envs/aidep/lib/python3.10/site-
packages/sklearn/neural_network/_multilayer_perceptron.py:690:
ConvergenceWarning: Stochastic Optimizer: Maximum iterations (1000) reached and
the optimization hasn't converged yet.
  warnings.warn(
/projects/proteinml/.links/miniconda3/envs/aidep/lib/python3.10/site-
packages/sklearn/neural_network/_multilayer_perceptron.py:690:
ConvergenceWarning: Stochastic Optimizer: Maximum iterations (1000) reached and
the optimization hasn't converged yet.
  warnings.warn(
/projects/proteinml/.links/miniconda3/envs/aidep/lib/python3.10/site-
packages/sklearn/neural_network/_multilayer_perceptron.py:690:
ConvergenceWarning: Stochastic Optimizer: Maximum iterations (1000) reached and
the optimization hasn't converged yet.
  warnings.warn(
/projects/proteinml/.links/miniconda3/envs/aidep/lib/python3.10/site-
packages/sklearn/neural_network/_multilayer_perceptron.py:690:
ConvergenceWarning: Stochastic Optimizer: Maximum iterations (1000) reached and
the optimization hasn't converged yet.
  warnings.warn(
/projects/proteinml/.links/miniconda3/envs/aidep/lib/python3.10/site-
packages/sklearn/neural_network/_multilayer_perceptron.py:690:
ConvergenceWarning: Stochastic Optimizer: Maximum iterations (1000) reached and
the optimization hasn't converged yet.
  warnings.warn(
/projects/proteinml/.links/miniconda3/envs/aidep/lib/python3.10/site-
packages/sklearn/neural_network/_multilayer_perceptron.py:690:
ConvergenceWarning: Stochastic Optimizer: Maximum iterations (1000) reached and
the optimization hasn't converged yet.
  warnings.warn(
/projects/proteinml/.links/miniconda3/envs/aidep/lib/python3.10/site-
```

```
packages/sklearn/neural_network/_multilayer_perceptron.py:690:
ConvergenceWarning: Stochastic Optimizer: Maximum iterations (1000) reached and
the optimization hasn't converged yet.
  warnings.warn(
/projects/proteinml/.links/miniconda3/envs/aidep/lib/python3.10/site-
packages/sklearn/neural_network/_multilayer_perceptron.py:690:
ConvergenceWarning: Stochastic Optimizer: Maximum iterations (1000) reached and
the optimization hasn't converged yet.
  warnings.warn(
/projects/proteinml/.links/miniconda3/envs/aidep/lib/python3.10/site-
packages/sklearn/neural_network/_multilayer_perceptron.py:690:
ConvergenceWarning: Stochastic Optimizer: Maximum iterations (1000) reached and
the optimization hasn't converged yet.
  warnings.warn(
/projects/proteinml/.links/miniconda3/envs/aidep/lib/python3.10/site-
packages/sklearn/neural_network/_multilayer_perceptron.py:690:
ConvergenceWarning: Stochastic Optimizer: Maximum iterations (1000) reached and
the optimization hasn't converged yet.
  warnings.warn(
/projects/proteinml/.links/miniconda3/envs/aidep/lib/python3.10/site-
packages/sklearn/neural_network/_multilayer_perceptron.py:690:
ConvergenceWarning: Stochastic Optimizer: Maximum iterations (1000) reached and
the optimization hasn't converged yet.
  warnings.warn(
/projects/proteinml/.links/miniconda3/envs/aidep/lib/python3.10/site-
packages/sklearn/neural_network/_multilayer_perceptron.py:690:
ConvergenceWarning: Stochastic Optimizer: Maximum iterations (1000) reached and
the optimization hasn't converged yet.
  warnings.warn(
/projects/proteinml/.links/miniconda3/envs/aidep/lib/python3.10/site-
packages/sklearn/neural_network/_multilayer_perceptron.py:690:
ConvergenceWarning: Stochastic Optimizer: Maximum iterations (1000) reached and
the optimization hasn't converged yet.
  warnings.warn(
/projects/proteinml/.links/miniconda3/envs/aidep/lib/python3.10/site-
packages/sklearn/neural_network/_multilayer_perceptron.py:690:
ConvergenceWarning: Stochastic Optimizer: Maximum iterations (1000) reached and
the optimization hasn't converged yet.
  warnings.warn(
/projects/proteinml/.links/miniconda3/envs/aidep/lib/python3.10/site-
packages/sklearn/neural_network/_multilayer_perceptron.py:690:
ConvergenceWarning: Stochastic Optimizer: Maximum iterations (1000) reached and
the optimization hasn't converged yet.
  warnings.warn(
/projects/proteinml/.links/miniconda3/envs/aidep/lib/python3.10/site-
packages/sklearn/neural_network/_multilayer_perceptron.py:690:
ConvergenceWarning: Stochastic Optimizer: Maximum iterations (1000) reached and
the optimization hasn't converged yet.
```

```
  warnings.warn(
/projects/proteinml/.links/miniconda3/envs/aidep/lib/python3.10/site-
packages/sklearn/neural_network/_multilayer_perceptron.py:690:
ConvergenceWarning: Stochastic Optimizer: Maximum iterations (1000) reached and
the optimization hasn't converged yet.
  warnings.warn(
/projects/proteinml/.links/miniconda3/envs/aidep/lib/python3.10/site-
packages/sklearn/neural_network/_multilayer_perceptron.py:690:
ConvergenceWarning: Stochastic Optimizer: Maximum iterations (1000) reached and
the optimization hasn't converged yet.
  warnings.warn(
/projects/proteinml/.links/miniconda3/envs/aidep/lib/python3.10/site-
packages/sklearn/neural_network/_multilayer_perceptron.py:690:
ConvergenceWarning: Stochastic Optimizer: Maximum iterations (1000) reached and
the optimization hasn't converged yet.
  warnings.warn(
/projects/proteinml/.links/miniconda3/envs/aidep/lib/python3.10/site-
packages/sklearn/neural_network/_multilayer_perceptron.py:690:
ConvergenceWarning: Stochastic Optimizer: Maximum iterations (1000) reached and
the optimization hasn't converged yet.
  warnings.warn(
/projects/proteinml/.links/miniconda3/envs/aidep/lib/python3.10/site-
packages/sklearn/neural_network/_multilayer_perceptron.py:690:
ConvergenceWarning: Stochastic Optimizer: Maximum iterations (1000) reached and
the optimization hasn't converged yet.
  warnings.warn(
/projects/proteinml/.links/miniconda3/envs/aidep/lib/python3.10/site-
packages/sklearn/neural_network/_multilayer_perceptron.py:690:
ConvergenceWarning: Stochastic Optimizer: Maximum iterations (1000) reached and
the optimization hasn't converged yet.
  warnings.warn(
/projects/proteinml/.links/miniconda3/envs/aidep/lib/python3.10/site-
packages/sklearn/neural_network/_multilayer_perceptron.py:690:
ConvergenceWarning: Stochastic Optimizer: Maximum iterations (1000) reached and
the optimization hasn't converged yet.
  warnings.warn(
/projects/proteinml/.links/miniconda3/envs/aidep/lib/python3.10/site-
packages/sklearn/neural_network/_multilayer_perceptron.py:690:
ConvergenceWarning: Stochastic Optimizer: Maximum iterations (1000) reached and
the optimization hasn't converged yet.
  warnings.warn(
/projects/proteinml/.links/miniconda3/envs/aidep/lib/python3.10/site-
packages/sklearn/neural_network/_multilayer_perceptron.py:690:
ConvergenceWarning: Stochastic Optimizer: Maximum iterations (1000) reached and
the optimization hasn't converged yet.
  warnings.warn(
/projects/proteinml/.links/miniconda3/envs/aidep/lib/python3.10/site-
packages/sklearn/neural_network/_multilayer_perceptron.py:690:
```

```
ConvergenceWarning: Stochastic Optimizer: Maximum iterations (1000) reached and
the optimization hasn't converged yet.
  warnings.warn(
/projects/proteinml/.links/miniconda3/envs/aidep/lib/python3.10/site-
packages/sklearn/neural_network/_multilayer_perceptron.py:690:
ConvergenceWarning: Stochastic Optimizer: Maximum iterations (1000) reached and
the optimization hasn't converged yet.
  warnings.warn(
/projects/proteinml/.links/miniconda3/envs/aidep/lib/python3.10/site-
packages/sklearn/neural_network/_multilayer_perceptron.py:690:
ConvergenceWarning: Stochastic Optimizer: Maximum iterations (1000) reached and
the optimization hasn't converged yet.
  warnings.warn(
/projects/proteinml/.links/miniconda3/envs/aidep/lib/python3.10/site-
packages/sklearn/neural_network/_multilayer_perceptron.py:690:
ConvergenceWarning: Stochastic Optimizer: Maximum iterations (1000) reached and
the optimization hasn't converged yet.
  warnings.warn(
/projects/proteinml/.links/miniconda3/envs/aidep/lib/python3.10/site-
packages/sklearn/neural_network/_multilayer_perceptron.py:690:
ConvergenceWarning: Stochastic Optimizer: Maximum iterations (1000) reached and
the optimization hasn't converged yet.
  warnings.warn(
/projects/proteinml/.links/miniconda3/envs/aidep/lib/python3.10/site-
packages/sklearn/neural_network/_multilayer_perceptron.py:690:
ConvergenceWarning: Stochastic Optimizer: Maximum iterations (1000) reached and
the optimization hasn't converged yet.
  warnings.warn(
/projects/proteinml/.links/miniconda3/envs/aidep/lib/python3.10/site-
packages/sklearn/neural_network/_multilayer_perceptron.py:690:
ConvergenceWarning: Stochastic Optimizer: Maximum iterations (1000) reached and
the optimization hasn't converged yet.
  warnings.warn(
/projects/proteinml/.links/miniconda3/envs/aidep/lib/python3.10/site-
packages/sklearn/neural_network/_multilayer_perceptron.py:690:
ConvergenceWarning: Stochastic Optimizer: Maximum iterations (1000) reached and
the optimization hasn't converged yet.
  warnings.warn(
/projects/proteinml/.links/miniconda3/envs/aidep/lib/python3.10/site-
packages/sklearn/neural_network/_multilayer_perceptron.py:690:
ConvergenceWarning: Stochastic Optimizer: Maximum iterations (1000) reached and
the optimization hasn't converged yet.
  warnings.warn(
/projects/proteinml/.links/miniconda3/envs/aidep/lib/python3.10/site-
packages/sklearn/neural_network/_multilayer_perceptron.py:690:
ConvergenceWarning: Stochastic Optimizer: Maximum iterations (1000) reached and
the optimization hasn't converged yet.
  warnings.warn(
```

```
/projects/proteinml/.links/miniconda3/envs/aidep/lib/python3.10/site-
packages/sklearn/neural_network/_multilayer_perceptron.py:690:
ConvergenceWarning: Stochastic Optimizer: Maximum iterations (1000) reached and
the optimization hasn't converged yet.
  warnings.warn(
/projects/proteinml/.links/miniconda3/envs/aidep/lib/python3.10/site-
packages/sklearn/neural_network/_multilayer_perceptron.py:690:
ConvergenceWarning: Stochastic Optimizer: Maximum iterations (1000) reached and
the optimization hasn't converged yet.
  warnings.warn(
/projects/proteinml/.links/miniconda3/envs/aidep/lib/python3.10/site-
packages/sklearn/neural_network/_multilayer_perceptron.py:690:
ConvergenceWarning: Stochastic Optimizer: Maximum iterations (1000) reached and
the optimization hasn't converged yet.
  warnings.warn(
/projects/proteinml/.links/miniconda3/envs/aidep/lib/python3.10/site-
packages/sklearn/neural_network/_multilayer_perceptron.py:690:
ConvergenceWarning: Stochastic Optimizer: Maximum iterations (1000) reached and
the optimization hasn't converged yet.
  warnings.warn(
/projects/proteinml/.links/miniconda3/envs/aidep/lib/python3.10/site-
packages/sklearn/neural_network/_multilayer_perceptron.py:690:
ConvergenceWarning: Stochastic Optimizer: Maximum iterations (1000) reached and
the optimization hasn't converged yet.
  warnings.warn(
/projects/proteinml/.links/miniconda3/envs/aidep/lib/python3.10/site-
packages/sklearn/neural_network/_multilayer_perceptron.py:690:
ConvergenceWarning: Stochastic Optimizer: Maximum iterations (1000) reached and
the optimization hasn't converged yet.
  warnings.warn(
/projects/proteinml/.links/miniconda3/envs/aidep/lib/python3.10/site-
packages/sklearn/neural_network/_multilayer_perceptron.py:690:
ConvergenceWarning: Stochastic Optimizer: Maximum iterations (1000) reached and
the optimization hasn't converged yet.
  warnings.warn(
/projects/proteinml/.links/miniconda3/envs/aidep/lib/python3.10/site-
packages/sklearn/neural_network/_multilayer_perceptron.py:690:
ConvergenceWarning: Stochastic Optimizer: Maximum iterations (1000) reached and
the optimization hasn't converged yet.
  warnings.warn(
/projects/proteinml/.links/miniconda3/envs/aidep/lib/python3.10/site-
packages/sklearn/neural_network/_multilayer_perceptron.py:690:
ConvergenceWarning: Stochastic Optimizer: Maximum iterations (1000) reached and
the optimization hasn't converged yet.
  warnings.warn(
/projects/proteinml/.links/miniconda3/envs/aidep/lib/python3.10/site-
packages/sklearn/neural_network/_multilayer_perceptron.py:690:
ConvergenceWarning: Stochastic Optimizer: Maximum iterations (1000) reached and
```

```
the optimization hasn't converged yet.
  warnings.warn(
/projects/proteinml/.links/miniconda3/envs/aidep/lib/python3.10/site-
packages/sklearn/neural_network/_multilayer_perceptron.py:690:
ConvergenceWarning: Stochastic Optimizer: Maximum iterations (1000) reached and
the optimization hasn't converged yet.
  warnings.warn(
/projects/proteinml/.links/miniconda3/envs/aidep/lib/python3.10/site-
packages/sklearn/neural_network/_multilayer_perceptron.py:690:
ConvergenceWarning: Stochastic Optimizer: Maximum iterations (1000) reached and
the optimization hasn't converged yet.
  warnings.warn(
/projects/proteinml/.links/miniconda3/envs/aidep/lib/python3.10/site-
packages/sklearn/neural_network/_multilayer_perceptron.py:690:
ConvergenceWarning: Stochastic Optimizer: Maximum iterations (1000) reached and
the optimization hasn't converged yet.
  warnings.warn(
/projects/proteinml/.links/miniconda3/envs/aidep/lib/python3.10/site-
packages/sklearn/neural_network/_multilayer_perceptron.py:690:
ConvergenceWarning: Stochastic Optimizer: Maximum iterations (1000) reached and
the optimization hasn't converged yet.
  warnings.warn(
/projects/proteinml/.links/miniconda3/envs/aidep/lib/python3.10/site-
packages/sklearn/neural_network/_multilayer_perceptron.py:690:
ConvergenceWarning: Stochastic Optimizer: Maximum iterations (1000) reached and
the optimization hasn't converged yet.
  warnings.warn(
/projects/proteinml/.links/miniconda3/envs/aidep/lib/python3.10/site-
packages/sklearn/neural_network/_multilayer_perceptron.py:690:
ConvergenceWarning: Stochastic Optimizer: Maximum iterations (1000) reached and
the optimization hasn't converged yet.
  warnings.warn(
/projects/proteinml/.links/miniconda3/envs/aidep/lib/python3.10/site-
packages/sklearn/neural_network/_multilayer_perceptron.py:690:
ConvergenceWarning: Stochastic Optimizer: Maximum iterations (1000) reached and
the optimization hasn't converged yet.
  warnings.warn(
/projects/proteinml/.links/miniconda3/envs/aidep/lib/python3.10/site-
packages/sklearn/neural_network/_multilayer_perceptron.py:690:
ConvergenceWarning: Stochastic Optimizer: Maximum iterations (1000) reached and
the optimization hasn't converged yet.
  warnings.warn(
/projects/proteinml/.links/miniconda3/envs/aidep/lib/python3.10/site-
packages/sklearn/neural_network/_multilayer_perceptron.py:690:
ConvergenceWarning: Stochastic Optimizer: Maximum iterations (1000) reached and
the optimization hasn't converged yet.
  warnings.warn(
/projects/proteinml/.links/miniconda3/envs/aidep/lib/python3.10/site-
```

```
packages/sklearn/neural_network/_multilayer_perceptron.py:690:
ConvergenceWarning: Stochastic Optimizer: Maximum iterations (1000) reached and
the optimization hasn't converged yet.
  warnings.warn(
/projects/proteinml/.links/miniconda3/envs/aidep/lib/python3.10/site-
packages/sklearn/neural_network/_multilayer_perceptron.py:690:
ConvergenceWarning: Stochastic Optimizer: Maximum iterations (1000) reached and
the optimization hasn't converged yet.
  warnings.warn(
/projects/proteinml/.links/miniconda3/envs/aidep/lib/python3.10/site-
packages/sklearn/neural_network/_multilayer_perceptron.py:690:
ConvergenceWarning: Stochastic Optimizer: Maximum iterations (1000) reached and
the optimization hasn't converged yet.
  warnings.warn(
/projects/proteinml/.links/miniconda3/envs/aidep/lib/python3.10/site-
packages/sklearn/neural_network/_multilayer_perceptron.py:690:
ConvergenceWarning: Stochastic Optimizer: Maximum iterations (1000) reached and
the optimization hasn't converged yet.
  warnings.warn(
/projects/proteinml/.links/miniconda3/envs/aidep/lib/python3.10/site-
packages/sklearn/neural_network/_multilayer_perceptron.py:690:
ConvergenceWarning: Stochastic Optimizer: Maximum iterations (1000) reached and
the optimization hasn't converged yet.
  warnings.warn(
/projects/proteinml/.links/miniconda3/envs/aidep/lib/python3.10/site-
packages/sklearn/neural_network/_multilayer_perceptron.py:690:
ConvergenceWarning: Stochastic Optimizer: Maximum iterations (1000) reached and
the optimization hasn't converged yet.
  warnings.warn(
/projects/proteinml/.links/miniconda3/envs/aidep/lib/python3.10/site-
packages/sklearn/neural_network/_multilayer_perceptron.py:690:
ConvergenceWarning: Stochastic Optimizer: Maximum iterations (1000) reached and
the optimization hasn't converged yet.
  warnings.warn(
/projects/proteinml/.links/miniconda3/envs/aidep/lib/python3.10/site-
packages/sklearn/neural_network/_multilayer_perceptron.py:690:
ConvergenceWarning: Stochastic Optimizer: Maximum iterations (1000) reached and
the optimization hasn't converged yet.
  warnings.warn(
/projects/proteinml/.links/miniconda3/envs/aidep/lib/python3.10/site-
packages/sklearn/neural_network/_multilayer_perceptron.py:690:
ConvergenceWarning: Stochastic Optimizer: Maximum iterations (1000) reached and
the optimization hasn't converged yet.
  warnings.warn(
/projects/proteinml/.links/miniconda3/envs/aidep/lib/python3.10/site-
packages/sklearn/neural_network/_multilayer_perceptron.py:690:
ConvergenceWarning: Stochastic Optimizer: Maximum iterations (1000) reached and
the optimization hasn't converged yet.
```

```
  warnings.warn(
/projects/proteinml/.links/miniconda3/envs/aidep/lib/python3.10/site-
packages/sklearn/neural_network/_multilayer_perceptron.py:690:
ConvergenceWarning: Stochastic Optimizer: Maximum iterations (1000) reached and
the optimization hasn't converged yet.
  warnings.warn(
/projects/proteinml/.links/miniconda3/envs/aidep/lib/python3.10/site-
packages/sklearn/neural_network/_multilayer_perceptron.py:690:
ConvergenceWarning: Stochastic Optimizer: Maximum iterations (1000) reached and
the optimization hasn't converged yet.
  warnings.warn(
/projects/proteinml/.links/miniconda3/envs/aidep/lib/python3.10/site-
packages/sklearn/neural_network/_multilayer_perceptron.py:690:
ConvergenceWarning: Stochastic Optimizer: Maximum iterations (1000) reached and
the optimization hasn't converged yet.
  warnings.warn(
/projects/proteinml/.links/miniconda3/envs/aidep/lib/python3.10/site-
packages/sklearn/neural_network/_multilayer_perceptron.py:690:
ConvergenceWarning: Stochastic Optimizer: Maximum iterations (1000) reached and
the optimization hasn't converged yet.
  warnings.warn(
/projects/proteinml/.links/miniconda3/envs/aidep/lib/python3.10/site-
packages/sklearn/neural_network/_multilayer_perceptron.py:690:
ConvergenceWarning: Stochastic Optimizer: Maximum iterations (1000) reached and
the optimization hasn't converged yet.
  warnings.warn(
/projects/proteinml/.links/miniconda3/envs/aidep/lib/python3.10/site-
packages/sklearn/neural_network/_multilayer_perceptron.py:690:
ConvergenceWarning: Stochastic Optimizer: Maximum iterations (1000) reached and
the optimization hasn't converged yet.
  warnings.warn(
/projects/proteinml/.links/miniconda3/envs/aidep/lib/python3.10/site-
packages/sklearn/neural_network/_multilayer_perceptron.py:690:
ConvergenceWarning: Stochastic Optimizer: Maximum iterations (1000) reached and
the optimization hasn't converged yet.
  warnings.warn(
/projects/proteinml/.links/miniconda3/envs/aidep/lib/python3.10/site-
packages/sklearn/neural_network/_multilayer_perceptron.py:690:
ConvergenceWarning: Stochastic Optimizer: Maximum iterations (1000) reached and
the optimization hasn't converged yet.
  warnings.warn(
/projects/proteinml/.links/miniconda3/envs/aidep/lib/python3.10/site-
packages/sklearn/neural_network/_multilayer_perceptron.py:690:
ConvergenceWarning: Stochastic Optimizer: Maximum iterations (1000) reached and
the optimization hasn't converged yet.
  warnings.warn(
/projects/proteinml/.links/miniconda3/envs/aidep/lib/python3.10/site-
packages/sklearn/neural_network/_multilayer_perceptron.py:690:
```

```
ConvergenceWarning: Stochastic Optimizer: Maximum iterations (1000) reached and
the optimization hasn't converged yet.
  warnings.warn(
/projects/proteinml/.links/miniconda3/envs/aidep/lib/python3.10/site-
packages/sklearn/neural_network/_multilayer_perceptron.py:690:
ConvergenceWarning: Stochastic Optimizer: Maximum iterations (1000) reached and
the optimization hasn't converged yet.
  warnings.warn(
/projects/proteinml/.links/miniconda3/envs/aidep/lib/python3.10/site-
packages/sklearn/neural_network/_multilayer_perceptron.py:690:
ConvergenceWarning: Stochastic Optimizer: Maximum iterations (1000) reached and
the optimization hasn't converged yet.
  warnings.warn(
/projects/proteinml/.links/miniconda3/envs/aidep/lib/python3.10/site-
packages/sklearn/neural_network/_multilayer_perceptron.py:690:
ConvergenceWarning: Stochastic Optimizer: Maximum iterations (1000) reached and
the optimization hasn't converged yet.
  warnings.warn(
/projects/proteinml/.links/miniconda3/envs/aidep/lib/python3.10/site-
packages/sklearn/neural_network/_multilayer_perceptron.py:690:
ConvergenceWarning: Stochastic Optimizer: Maximum iterations (1000) reached and
the optimization hasn't converged yet.
  warnings.warn(
/projects/proteinml/.links/miniconda3/envs/aidep/lib/python3.10/site-
packages/sklearn/neural_network/_multilayer_perceptron.py:690:
ConvergenceWarning: Stochastic Optimizer: Maximum iterations (1000) reached and
the optimization hasn't converged yet.
  warnings.warn(
/projects/proteinml/.links/miniconda3/envs/aidep/lib/python3.10/site-
packages/sklearn/neural_network/_multilayer_perceptron.py:690:
ConvergenceWarning: Stochastic Optimizer: Maximum iterations (1000) reached and
the optimization hasn't converged yet.
  warnings.warn(
/projects/proteinml/.links/miniconda3/envs/aidep/lib/python3.10/site-
packages/sklearn/neural_network/_multilayer_perceptron.py:690:
ConvergenceWarning: Stochastic Optimizer: Maximum iterations (1000) reached and
the optimization hasn't converged yet.
  warnings.warn(
/projects/proteinml/.links/miniconda3/envs/aidep/lib/python3.10/site-
packages/sklearn/neural_network/_multilayer_perceptron.py:690:
ConvergenceWarning: Stochastic Optimizer: Maximum iterations (1000) reached and
the optimization hasn't converged yet.
  warnings.warn(
/projects/proteinml/.links/miniconda3/envs/aidep/lib/python3.10/site-
packages/sklearn/neural_network/_multilayer_perceptron.py:690:
ConvergenceWarning: Stochastic Optimizer: Maximum iterations (1000) reached and
the optimization hasn't converged yet.
  warnings.warn(
```

```
/projects/proteinml/.links/miniconda3/envs/aidep/lib/python3.10/site-
packages/sklearn/neural_network/_multilayer_perceptron.py:690:
ConvergenceWarning: Stochastic Optimizer: Maximum iterations (1000) reached and
the optimization hasn't converged yet.
  warnings.warn(
/projects/proteinml/.links/miniconda3/envs/aidep/lib/python3.10/site-
packages/sklearn/neural_network/_multilayer_perceptron.py:690:
ConvergenceWarning: Stochastic Optimizer: Maximum iterations (1000) reached and
the optimization hasn't converged yet.
  warnings.warn(
/projects/proteinml/.links/miniconda3/envs/aidep/lib/python3.10/site-
packages/sklearn/neural_network/_multilayer_perceptron.py:690:
ConvergenceWarning: Stochastic Optimizer: Maximum iterations (1000) reached and
the optimization hasn't converged yet.
  warnings.warn(
/projects/proteinml/.links/miniconda3/envs/aidep/lib/python3.10/site-
packages/sklearn/neural_network/_multilayer_perceptron.py:690:
ConvergenceWarning: Stochastic Optimizer: Maximum iterations (1000) reached and
the optimization hasn't converged yet.
  warnings.warn(
/projects/proteinml/.links/miniconda3/envs/aidep/lib/python3.10/site-
packages/sklearn/neural_network/_multilayer_perceptron.py:690:
ConvergenceWarning: Stochastic Optimizer: Maximum iterations (1000) reached and
the optimization hasn't converged yet.
  warnings.warn(
/projects/proteinml/.links/miniconda3/envs/aidep/lib/python3.10/site-
packages/sklearn/neural_network/_multilayer_perceptron.py:690:
ConvergenceWarning: Stochastic Optimizer: Maximum iterations (1000) reached and
the optimization hasn't converged yet.
  warnings.warn(
/projects/proteinml/.links/miniconda3/envs/aidep/lib/python3.10/site-
packages/sklearn/neural_network/_multilayer_perceptron.py:690:
ConvergenceWarning: Stochastic Optimizer: Maximum iterations (1000) reached and
the optimization hasn't converged yet.
  warnings.warn(
/projects/proteinml/.links/miniconda3/envs/aidep/lib/python3.10/site-
packages/sklearn/neural_network/_multilayer_perceptron.py:690:
ConvergenceWarning: Stochastic Optimizer: Maximum iterations (1000) reached and
the optimization hasn't converged yet.
  warnings.warn(
/projects/proteinml/.links/miniconda3/envs/aidep/lib/python3.10/site-
packages/sklearn/neural_network/_multilayer_perceptron.py:690:
ConvergenceWarning: Stochastic Optimizer: Maximum iterations (1000) reached and
the optimization hasn't converged yet.
  warnings.warn(
/projects/proteinml/.links/miniconda3/envs/aidep/lib/python3.10/site-
packages/sklearn/neural_network/_multilayer_perceptron.py:690:
ConvergenceWarning: Stochastic Optimizer: Maximum iterations (1000) reached and
```

```
the optimization hasn't converged yet.
  warnings.warn(
/projects/proteinml/.links/miniconda3/envs/aidep/lib/python3.10/site-
packages/sklearn/neural_network/_multilayer_perceptron.py:690:
ConvergenceWarning: Stochastic Optimizer: Maximum iterations (1000) reached and
the optimization hasn't converged yet.
  warnings.warn(
/projects/proteinml/.links/miniconda3/envs/aidep/lib/python3.10/site-
packages/sklearn/neural_network/_multilayer_perceptron.py:690:
ConvergenceWarning: Stochastic Optimizer: Maximum iterations (1000) reached and
the optimization hasn't converged yet.
  warnings.warn(
/projects/proteinml/.links/miniconda3/envs/aidep/lib/python3.10/site-
packages/sklearn/neural_network/_multilayer_perceptron.py:690:
ConvergenceWarning: Stochastic Optimizer: Maximum iterations (1000) reached and
the optimization hasn't converged yet.
  warnings.warn(
/projects/proteinml/.links/miniconda3/envs/aidep/lib/python3.10/site-
packages/sklearn/neural_network/_multilayer_perceptron.py:690:
ConvergenceWarning: Stochastic Optimizer: Maximum iterations (1000) reached and
the optimization hasn't converged yet.
  warnings.warn(
/projects/proteinml/.links/miniconda3/envs/aidep/lib/python3.10/site-
packages/sklearn/neural_network/_multilayer_perceptron.py:690:
ConvergenceWarning: Stochastic Optimizer: Maximum iterations (1000) reached and
the optimization hasn't converged yet.
  warnings.warn(
/projects/proteinml/.links/miniconda3/envs/aidep/lib/python3.10/site-
packages/sklearn/neural_network/_multilayer_perceptron.py:690:
ConvergenceWarning: Stochastic Optimizer: Maximum iterations (1000) reached and
the optimization hasn't converged yet.
  warnings.warn(
/projects/proteinml/.links/miniconda3/envs/aidep/lib/python3.10/site-
packages/sklearn/neural_network/_multilayer_perceptron.py:690:
ConvergenceWarning: Stochastic Optimizer: Maximum iterations (1000) reached and
the optimization hasn't converged yet.
  warnings.warn(
/projects/proteinml/.links/miniconda3/envs/aidep/lib/python3.10/site-
packages/sklearn/neural_network/_multilayer_perceptron.py:690:
ConvergenceWarning: Stochastic Optimizer: Maximum iterations (1000) reached and
the optimization hasn't converged yet.
  warnings.warn(
/projects/proteinml/.links/miniconda3/envs/aidep/lib/python3.10/site-
packages/sklearn/neural_network/_multilayer_perceptron.py:690:
ConvergenceWarning: Stochastic Optimizer: Maximum iterations (1000) reached and
the optimization hasn't converged yet.
  warnings.warn(
/projects/proteinml/.links/miniconda3/envs/aidep/lib/python3.10/site-
```

```
packages/sklearn/neural_network/_multilayer_perceptron.py:690:
ConvergenceWarning: Stochastic Optimizer: Maximum iterations (1000) reached and
the optimization hasn't converged yet.
  warnings.warn(
/projects/proteinml/.links/miniconda3/envs/aidep/lib/python3.10/site-
packages/sklearn/neural_network/_multilayer_perceptron.py:690:
ConvergenceWarning: Stochastic Optimizer: Maximum iterations (1000) reached and
the optimization hasn't converged yet.
  warnings.warn(
/projects/proteinml/.links/miniconda3/envs/aidep/lib/python3.10/site-
packages/sklearn/neural_network/_multilayer_perceptron.py:690:
ConvergenceWarning: Stochastic Optimizer: Maximum iterations (1000) reached and
the optimization hasn't converged yet.
  warnings.warn(
/projects/proteinml/.links/miniconda3/envs/aidep/lib/python3.10/site-
packages/sklearn/neural_network/_multilayer_perceptron.py:690:
ConvergenceWarning: Stochastic Optimizer: Maximum iterations (1000) reached and
the optimization hasn't converged yet.
  warnings.warn(
/projects/proteinml/.links/miniconda3/envs/aidep/lib/python3.10/site-
packages/sklearn/neural_network/_multilayer_perceptron.py:690:
ConvergenceWarning: Stochastic Optimizer: Maximum iterations (1000) reached and
the optimization hasn't converged yet.
  warnings.warn(
/projects/proteinml/.links/miniconda3/envs/aidep/lib/python3.10/site-
packages/sklearn/neural_network/_multilayer_perceptron.py:690:
ConvergenceWarning: Stochastic Optimizer: Maximum iterations (1000) reached and
the optimization hasn't converged yet.
  warnings.warn(
/projects/proteinml/.links/miniconda3/envs/aidep/lib/python3.10/site-
packages/sklearn/neural_network/_multilayer_perceptron.py:690:
ConvergenceWarning: Stochastic Optimizer: Maximum iterations (1000) reached and
the optimization hasn't converged yet.
  warnings.warn(
/projects/proteinml/.links/miniconda3/envs/aidep/lib/python3.10/site-
packages/sklearn/neural_network/_multilayer_perceptron.py:690:
ConvergenceWarning: Stochastic Optimizer: Maximum iterations (1000) reached and
the optimization hasn't converged yet.
  warnings.warn(
/projects/proteinml/.links/miniconda3/envs/aidep/lib/python3.10/site-
packages/sklearn/neural_network/_multilayer_perceptron.py:690:
ConvergenceWarning: Stochastic Optimizer: Maximum iterations (1000) reached and
the optimization hasn't converged yet.
  warnings.warn(
/projects/proteinml/.links/miniconda3/envs/aidep/lib/python3.10/site-
packages/sklearn/neural_network/_multilayer_perceptron.py:690:
ConvergenceWarning: Stochastic Optimizer: Maximum iterations (1000) reached and
the optimization hasn't converged yet.
```

```
  warnings.warn(
/projects/proteinml/.links/miniconda3/envs/aidep/lib/python3.10/site-
packages/sklearn/neural_network/_multilayer_perceptron.py:690:
ConvergenceWarning: Stochastic Optimizer: Maximum iterations (1000) reached and
the optimization hasn't converged yet.
  warnings.warn(
/projects/proteinml/.links/miniconda3/envs/aidep/lib/python3.10/site-
packages/sklearn/neural_network/_multilayer_perceptron.py:690:
ConvergenceWarning: Stochastic Optimizer: Maximum iterations (1000) reached and
the optimization hasn't converged yet.
  warnings.warn(
/projects/proteinml/.links/miniconda3/envs/aidep/lib/python3.10/site-
packages/sklearn/neural_network/_multilayer_perceptron.py:690:
ConvergenceWarning: Stochastic Optimizer: Maximum iterations (1000) reached and
the optimization hasn't converged yet.
  warnings.warn(
/projects/proteinml/.links/miniconda3/envs/aidep/lib/python3.10/site-
packages/sklearn/neural_network/_multilayer_perceptron.py:690:
ConvergenceWarning: Stochastic Optimizer: Maximum iterations (1000) reached and
the optimization hasn't converged yet.
  warnings.warn(
/projects/proteinml/.links/miniconda3/envs/aidep/lib/python3.10/site-
packages/sklearn/neural_network/_multilayer_perceptron.py:690:
ConvergenceWarning: Stochastic Optimizer: Maximum iterations (1000) reached and
the optimization hasn't converged yet.
  warnings.warn(
/projects/proteinml/.links/miniconda3/envs/aidep/lib/python3.10/site-
packages/sklearn/neural_network/_multilayer_perceptron.py:690:
ConvergenceWarning: Stochastic Optimizer: Maximum iterations (1000) reached and
the optimization hasn't converged yet.
  warnings.warn(
/projects/proteinml/.links/miniconda3/envs/aidep/lib/python3.10/site-
packages/sklearn/neural_network/_multilayer_perceptron.py:690:
ConvergenceWarning: Stochastic Optimizer: Maximum iterations (1000) reached and
the optimization hasn't converged yet.
  warnings.warn(
/projects/proteinml/.links/miniconda3/envs/aidep/lib/python3.10/site-
packages/sklearn/neural_network/_multilayer_perceptron.py:690:
ConvergenceWarning: Stochastic Optimizer: Maximum iterations (1000) reached and
the optimization hasn't converged yet.
  warnings.warn(
/projects/proteinml/.links/miniconda3/envs/aidep/lib/python3.10/site-
packages/sklearn/neural_network/_multilayer_perceptron.py:690:
ConvergenceWarning: Stochastic Optimizer: Maximum iterations (1000) reached and
the optimization hasn't converged yet.
  warnings.warn(
/projects/proteinml/.links/miniconda3/envs/aidep/lib/python3.10/site-
packages/sklearn/neural_network/_multilayer_perceptron.py:690:
```

```
ConvergenceWarning: Stochastic Optimizer: Maximum iterations (1000) reached and
the optimization hasn't converged yet.
  warnings.warn(
/projects/proteinml/.links/miniconda3/envs/aidep/lib/python3.10/site-
packages/sklearn/neural_network/_multilayer_perceptron.py:690:
ConvergenceWarning: Stochastic Optimizer: Maximum iterations (1000) reached and
the optimization hasn't converged yet.
  warnings.warn(
/projects/proteinml/.links/miniconda3/envs/aidep/lib/python3.10/site-
packages/sklearn/neural_network/_multilayer_perceptron.py:690:
ConvergenceWarning: Stochastic Optimizer: Maximum iterations (1000) reached and
the optimization hasn't converged yet.
  warnings.warn(
/projects/proteinml/.links/miniconda3/envs/aidep/lib/python3.10/site-
packages/sklearn/neural_network/_multilayer_perceptron.py:690:
ConvergenceWarning: Stochastic Optimizer: Maximum iterations (1000) reached and
the optimization hasn't converged yet.
  warnings.warn(
/projects/proteinml/.links/miniconda3/envs/aidep/lib/python3.10/site-
packages/sklearn/neural_network/_multilayer_perceptron.py:690:
ConvergenceWarning: Stochastic Optimizer: Maximum iterations (1000) reached and
the optimization hasn't converged yet.
  warnings.warn(
/projects/proteinml/.links/miniconda3/envs/aidep/lib/python3.10/site-
packages/sklearn/neural_network/_multilayer_perceptron.py:690:
ConvergenceWarning: Stochastic Optimizer: Maximum iterations (1000) reached and
the optimization hasn't converged yet.
  warnings.warn(
/projects/proteinml/.links/miniconda3/envs/aidep/lib/python3.10/site-
packages/sklearn/neural_network/_multilayer_perceptron.py:690:
ConvergenceWarning: Stochastic Optimizer: Maximum iterations (1000) reached and
the optimization hasn't converged yet.
  warnings.warn(
/projects/proteinml/.links/miniconda3/envs/aidep/lib/python3.10/site-
packages/sklearn/neural_network/_multilayer_perceptron.py:690:
ConvergenceWarning: Stochastic Optimizer: Maximum iterations (1000) reached and
the optimization hasn't converged yet.
  warnings.warn(
/projects/proteinml/.links/miniconda3/envs/aidep/lib/python3.10/site-
packages/sklearn/neural_network/_multilayer_perceptron.py:690:
ConvergenceWarning: Stochastic Optimizer: Maximum iterations (1000) reached and
the optimization hasn't converged yet.
  warnings.warn(
/projects/proteinml/.links/miniconda3/envs/aidep/lib/python3.10/site-
packages/sklearn/neural_network/_multilayer_perceptron.py:690:
ConvergenceWarning: Stochastic Optimizer: Maximum iterations (1000) reached and
the optimization hasn't converged yet.
  warnings.warn(
```

```
/projects/proteinml/.links/miniconda3/envs/aidep/lib/python3.10/site-
packages/sklearn/neural_network/_multilayer_perceptron.py:690:
ConvergenceWarning: Stochastic Optimizer: Maximum iterations (1000) reached and
the optimization hasn't converged yet.
  warnings.warn(
/projects/proteinml/.links/miniconda3/envs/aidep/lib/python3.10/site-
packages/sklearn/neural_network/_multilayer_perceptron.py:690:
ConvergenceWarning: Stochastic Optimizer: Maximum iterations (1000) reached and
the optimization hasn't converged yet.
  warnings.warn(
/projects/proteinml/.links/miniconda3/envs/aidep/lib/python3.10/site-
packages/sklearn/neural_network/_multilayer_perceptron.py:690:
ConvergenceWarning: Stochastic Optimizer: Maximum iterations (1000) reached and
the optimization hasn't converged yet.
  warnings.warn(
/projects/proteinml/.links/miniconda3/envs/aidep/lib/python3.10/site-
packages/sklearn/neural_network/_multilayer_perceptron.py:690:
ConvergenceWarning: Stochastic Optimizer: Maximum iterations (1000) reached and
the optimization hasn't converged yet.
  warnings.warn(
/projects/proteinml/.links/miniconda3/envs/aidep/lib/python3.10/site-
packages/sklearn/neural_network/_multilayer_perceptron.py:690:
ConvergenceWarning: Stochastic Optimizer: Maximum iterations (1000) reached and
the optimization hasn't converged yet.
  warnings.warn(
/projects/proteinml/.links/miniconda3/envs/aidep/lib/python3.10/site-
packages/sklearn/neural_network/_multilayer_perceptron.py:690:
ConvergenceWarning: Stochastic Optimizer: Maximum iterations (1000) reached and
the optimization hasn't converged yet.
  warnings.warn(
/projects/proteinml/.links/miniconda3/envs/aidep/lib/python3.10/site-
packages/sklearn/neural_network/_multilayer_perceptron.py:690:
ConvergenceWarning: Stochastic Optimizer: Maximum iterations (1000) reached and
the optimization hasn't converged yet.
  warnings.warn(
/projects/proteinml/.links/miniconda3/envs/aidep/lib/python3.10/site-
packages/sklearn/neural_network/_multilayer_perceptron.py:690:
ConvergenceWarning: Stochastic Optimizer: Maximum iterations (1000) reached and
the optimization hasn't converged yet.
  warnings.warn(
/projects/proteinml/.links/miniconda3/envs/aidep/lib/python3.10/site-
packages/sklearn/neural_network/_multilayer_perceptron.py:690:
ConvergenceWarning: Stochastic Optimizer: Maximum iterations (1000) reached and
the optimization hasn't converged yet.
  warnings.warn(
/projects/proteinml/.links/miniconda3/envs/aidep/lib/python3.10/site-
packages/sklearn/neural_network/_multilayer_perceptron.py:690:
ConvergenceWarning: Stochastic Optimizer: Maximum iterations (1000) reached and
```

```
the optimization hasn't converged yet.
  warnings.warn(
/projects/proteinml/.links/miniconda3/envs/aidep/lib/python3.10/site-
packages/sklearn/neural_network/_multilayer_perceptron.py:690:
ConvergenceWarning: Stochastic Optimizer: Maximum iterations (1000) reached and
the optimization hasn't converged yet.
  warnings.warn(
/projects/proteinml/.links/miniconda3/envs/aidep/lib/python3.10/site-
packages/sklearn/neural_network/_multilayer_perceptron.py:690:
ConvergenceWarning: Stochastic Optimizer: Maximum iterations (1000) reached and
the optimization hasn't converged yet.
  warnings.warn(
/projects/proteinml/.links/miniconda3/envs/aidep/lib/python3.10/site-
packages/sklearn/neural_network/_multilayer_perceptron.py:690:
ConvergenceWarning: Stochastic Optimizer: Maximum iterations (1000) reached and
the optimization hasn't converged yet.
  warnings.warn(
/projects/proteinml/.links/miniconda3/envs/aidep/lib/python3.10/site-
packages/sklearn/neural_network/_multilayer_perceptron.py:690:
ConvergenceWarning: Stochastic Optimizer: Maximum iterations (1000) reached and
the optimization hasn't converged yet.
  warnings.warn(
/projects/proteinml/.links/miniconda3/envs/aidep/lib/python3.10/site-
packages/sklearn/neural_network/_multilayer_perceptron.py:690:
ConvergenceWarning: Stochastic Optimizer: Maximum iterations (1000) reached and
the optimization hasn't converged yet.
  warnings.warn(
/projects/proteinml/.links/miniconda3/envs/aidep/lib/python3.10/site-
packages/sklearn/neural_network/_multilayer_perceptron.py:690:
ConvergenceWarning: Stochastic Optimizer: Maximum iterations (1000) reached and
the optimization hasn't converged yet.
  warnings.warn(
/projects/proteinml/.links/miniconda3/envs/aidep/lib/python3.10/site-
packages/sklearn/neural_network/_multilayer_perceptron.py:690:
ConvergenceWarning: Stochastic Optimizer: Maximum iterations (1000) reached and
the optimization hasn't converged yet.
  warnings.warn(
/projects/proteinml/.links/miniconda3/envs/aidep/lib/python3.10/site-
packages/sklearn/neural_network/_multilayer_perceptron.py:690:
ConvergenceWarning: Stochastic Optimizer: Maximum iterations (1000) reached and
the optimization hasn't converged yet.
  warnings.warn(
/projects/proteinml/.links/miniconda3/envs/aidep/lib/python3.10/site-
packages/sklearn/neural_network/_multilayer_perceptron.py:690:
ConvergenceWarning: Stochastic Optimizer: Maximum iterations (1000) reached and
the optimization hasn't converged yet.
  warnings.warn(
/projects/proteinml/.links/miniconda3/envs/aidep/lib/python3.10/site-
```

```
packages/sklearn/neural_network/_multilayer_perceptron.py:690:
ConvergenceWarning: Stochastic Optimizer: Maximum iterations (1000) reached and
the optimization hasn't converged yet.
  warnings.warn(
/projects/proteinml/.links/miniconda3/envs/aidep/lib/python3.10/site-
packages/sklearn/neural_network/_multilayer_perceptron.py:690:
ConvergenceWarning: Stochastic Optimizer: Maximum iterations (1000) reached and
the optimization hasn't converged yet.
  warnings.warn(
/projects/proteinml/.links/miniconda3/envs/aidep/lib/python3.10/site-
packages/sklearn/neural_network/_multilayer_perceptron.py:690:
ConvergenceWarning: Stochastic Optimizer: Maximum iterations (1000) reached and
the optimization hasn't converged yet.
  warnings.warn(
/projects/proteinml/.links/miniconda3/envs/aidep/lib/python3.10/site-
packages/sklearn/neural_network/_multilayer_perceptron.py:690:
ConvergenceWarning: Stochastic Optimizer: Maximum iterations (1000) reached and
the optimization hasn't converged yet.
  warnings.warn(
/projects/proteinml/.links/miniconda3/envs/aidep/lib/python3.10/site-
packages/sklearn/neural_network/_multilayer_perceptron.py:690:
ConvergenceWarning: Stochastic Optimizer: Maximum iterations (1000) reached and
the optimization hasn't converged yet.
  warnings.warn(
/projects/proteinml/.links/miniconda3/envs/aidep/lib/python3.10/site-
packages/sklearn/neural_network/_multilayer_perceptron.py:690:
ConvergenceWarning: Stochastic Optimizer: Maximum iterations (1000) reached and
the optimization hasn't converged yet.
  warnings.warn(
/projects/proteinml/.links/miniconda3/envs/aidep/lib/python3.10/site-
packages/sklearn/neural_network/_multilayer_perceptron.py:690:
ConvergenceWarning: Stochastic Optimizer: Maximum iterations (1000) reached and
the optimization hasn't converged yet.
  warnings.warn(
/projects/proteinml/.links/miniconda3/envs/aidep/lib/python3.10/site-
packages/sklearn/neural_network/_multilayer_perceptron.py:690:
ConvergenceWarning: Stochastic Optimizer: Maximum iterations (1000) reached and
the optimization hasn't converged yet.
  warnings.warn(
/projects/proteinml/.links/miniconda3/envs/aidep/lib/python3.10/site-
packages/sklearn/neural_network/_multilayer_perceptron.py:690:
ConvergenceWarning: Stochastic Optimizer: Maximum iterations (1000) reached and
the optimization hasn't converged yet.
  warnings.warn(
/projects/proteinml/.links/miniconda3/envs/aidep/lib/python3.10/site-
packages/sklearn/neural_network/_multilayer_perceptron.py:690:
ConvergenceWarning: Stochastic Optimizer: Maximum iterations (1000) reached and
the optimization hasn't converged yet.
```

```
  warnings.warn(
/projects/proteinml/.links/miniconda3/envs/aidep/lib/python3.10/site-
packages/sklearn/neural_network/_multilayer_perceptron.py:690:
ConvergenceWarning: Stochastic Optimizer: Maximum iterations (1000) reached and
the optimization hasn't converged yet.
  warnings.warn(
/projects/proteinml/.links/miniconda3/envs/aidep/lib/python3.10/site-
packages/sklearn/neural_network/_multilayer_perceptron.py:690:
ConvergenceWarning: Stochastic Optimizer: Maximum iterations (1000) reached and
the optimization hasn't converged yet.
  warnings.warn(
/projects/proteinml/.links/miniconda3/envs/aidep/lib/python3.10/site-
packages/sklearn/neural_network/_multilayer_perceptron.py:690:
ConvergenceWarning: Stochastic Optimizer: Maximum iterations (1000) reached and
the optimization hasn't converged yet.
  warnings.warn(
/projects/proteinml/.links/miniconda3/envs/aidep/lib/python3.10/site-
packages/sklearn/neural_network/_multilayer_perceptron.py:690:
ConvergenceWarning: Stochastic Optimizer: Maximum iterations (1000) reached and
the optimization hasn't converged yet.
  warnings.warn(
/projects/proteinml/.links/miniconda3/envs/aidep/lib/python3.10/site-
packages/sklearn/neural_network/_multilayer_perceptron.py:690:
ConvergenceWarning: Stochastic Optimizer: Maximum iterations (1000) reached and
the optimization hasn't converged yet.
  warnings.warn(
/projects/proteinml/.links/miniconda3/envs/aidep/lib/python3.10/site-
packages/sklearn/neural_network/_multilayer_perceptron.py:690:
ConvergenceWarning: Stochastic Optimizer: Maximum iterations (1000) reached and
the optimization hasn't converged yet.
  warnings.warn(
/projects/proteinml/.links/miniconda3/envs/aidep/lib/python3.10/site-
packages/sklearn/neural_network/_multilayer_perceptron.py:690:
ConvergenceWarning: Stochastic Optimizer: Maximum iterations (1000) reached and
the optimization hasn't converged yet.
  warnings.warn(
```

[33]:
```
['evc_esm4sites_mlp_scores.joblib']
```

1.7 5. Compare and visualize results

[2]:
```
zs_scores = {
    'evc_none_none': joblib.load('evc_none_none_scores.joblib'),
    'esm_none_none': joblib.load('esm_none_none_scores.joblib'),
    'msa_none_none': joblib.load('msa_none_none_scores.joblib'),
}
supervised_scores = {
```

```
    'none_ohe_linear': joblib.load('none_ohe_linear_scores.joblib'),
    'none_ohe_mlp': joblib.load('none_ohe_mlp_scores.joblib'),
    'none_esmfull_linear': joblib.load('none_esmfull_linear_scores.joblib'),
    'none_esmfull_mlp': joblib.load('none_esmfull_mlp_scores.joblib'),
    'none_esm4sites_linear': joblib.load('none_esm4sites_linear_scores.joblib'),
    'none_esm4sites_mlp': joblib.load('none_esm4sites_mlp_scores.joblib'),
    'evc_ohe_linear': joblib.load('evc_ohe_linear_scores.joblib'),
    'evc_ohe_mlp': joblib.load('evc_ohe_mlp_scores.joblib'),
    'evc_esmfull_linear': joblib.load('evc_esmfull_linear_scores.joblib'),
    'evc_esmfull_mlp': joblib.load('evc_esmfull_mlp_scores.joblib'),
    'evc_esm4sites_linear': joblib.load('evc_esm4sites_linear_scores.joblib'),
    'evc_esm4sites_mlp': joblib.load('evc_esm4sites_mlp_scores.joblib'),
}
```

[3]:
```
data = []
for model, score_list in supervised_scores.items():
    for score_dict in score_list:
        training_size = score_dict['training_size']
        for metric in ['kendall_tau', 'spearman', 'top10_in_plate']:
            for point in score_dict[f'test_{metric}']:
                data.append({
                    'Model': model,
                    'Metric': metric,
                    'Score': point,
                    'Training Size': training_size
                })

supervised_df = pd.DataFrame(data)
supervised_df[['zs', 'embedding', 'topmodel']] = supervised_df['Model'].str.
 ↪split('_', expand=True)
data = []
for model, scores in zs_scores.items():
    for metric in ['kendall_tau', 'spearman', 'top10_in_plate']:
        for point in scores[f'test_{metric}']:
            data.append({
                'Model': model,
                'Metric': metric,
                'Score': point,
                'Training Size': 0.0
            })
zs_df = pd.DataFrame(data)
zs_df[['zs', 'embedding', 'topmodel']] = zs_df['Model'].str.split('_',␣
 ↪expand=True)
```

[4]:
```
# determined by embedding type
color_map = {
    'none': 'grey',
```

```
    'ohe': 'tab:blue',
    'esmfull': 'tab:orange',
    'esm4sites': 'tab:green',
}
# determined by ZS method
linestyle_map = {
    'none': ':',
    'evc': '-',
    'esm': '--',
    'msa': '-.',
}
# determined by top model
marker_map = {
    'none': 'o',
    'linear': 's',
    'mlp': 'd',
}
```

[5]:
```
def get_name_logic(string):
    split = string.split('_')
    zs_input = split[0]
    embedding = split[1]
    topmodel = split[2]
    if embedding == 'none' or topmodel == 'none':
        type_ = 'ZS'
    elif zs_input != 'none':
        type_ = 'Aug.'
    else:
        type_ = 'Sup.'

    output = ''
    if zs_input != 'none':
        output += zs_input + ''
    if embedding != 'none':
        if len(output) > 0:
            output += '+'
        output += embedding + ''
    if topmodel != 'none':
        output += '->'
        output += topmodel

    output += ' (' + type_ + ')'
    return output
```

[6]:
```
supervised_df['name'] = supervised_df.apply(lambda x:␣
↪get_name_logic(x['Model']), axis=1)
```

```
zs_df['name'] = zs_df.apply(lambda x: get_name_logic(x['Model']), axis=1)
```

[7]:
```
df
```

```
---------------------------------------------------------------------------
NameError                                 Traceback (most recent call last)
Cell In[7], line 1
----> 1 df

NameError: name 'df' is not defined
```

[9]:
```
# create plot of ZS vs supervised vs augmented linear, like original paper
metric = 'kendall_tau'

fig, ax = plt.subplots(figsize=(6, 6))

for model, df in supervised_df.groupby('Model'):
    df_ = df[df['Metric'] == metric]
    color, linestyle, marker = color_map[model.split('_')[1]],␣
↪linestyle_map[model.split('_')[0]], marker_map[model.split('_')[2]]
    if marker == 'd':
        continue
    name = df_['name'].iloc[0]

    sns.lineplot(x='Training Size', y='Score', data=df_, ax=ax,
                label=name, color=color, linestyle=linestyle,
                # change the marker shape and edeg style to black
                marker=marker, markeredgecolor='black', markeredgewidth=2,␣
↪alpha=.7)

for model, df in zs_df.groupby('Model'):
    df_ = df[df['Metric'] == metric]
    color, linestyle, marker = color_map[model.split('_')[1]],␣
↪linestyle_map[model.split('_')[0]], marker_map[model.split('_')[2]]
    name = df_['name'].iloc[0]
    xmin = ax.get_xlim()[0]
    xmax = ax.get_xlim()[1]
    mean = df_['Score'].mean()
    ax.hlines(mean, xmin, xmax, color=color, linestyle=linestyle, label=name)

ax.set_xlabel('training size')
ax.set_ylabel("Kendall's tau")
ax.set_xscale('log')
plt.legend(loc='upper right', bbox_to_anchor=(2.0, .8))
plt.savefig('zs_vs_supervised_vs_augmented.png', bbox_inches='tight', dpi=300)
```

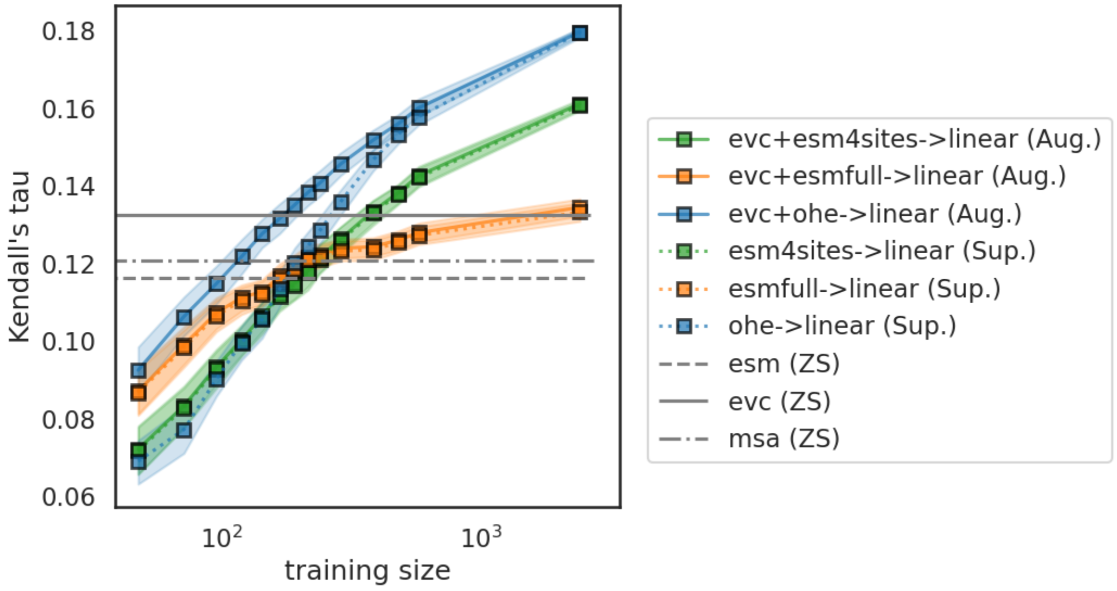

[10]:

```
# now compare linear to nonlinear models
metric = 'kendall_tau'

fig, ax = plt.subplots(figsize=(6, 6))

for model, df in supervised_df.groupby('Model'):
    df_ = df[df['Metric'] == metric]
    color, linestyle, marker = color_map[model.split('_')[1]],␣
↪linestyle_map[model.split('_')[0]], marker_map[model.split('_')[2]]
    if linestyle != ':':
        continue
    name = df_['name'].iloc[0]
    sns.lineplot(x='Training Size', y='Score', data=df_, ax=ax,
                 label=name, color=color, linestyle=linestyle, marker=marker)

ax.set_xlabel('Training Size')
ax.set_ylabel("Kendall's Tau")
plt.legend(loc='upper right', bbox_to_anchor=(2.0, .8))
# plt.savefig('nonlinear_vs_linear_kendall.png', bbox_inches='tight', dpi=300)
```

[10]: <matplotlib.legend.Legend at 0x7fccbddba6e0>

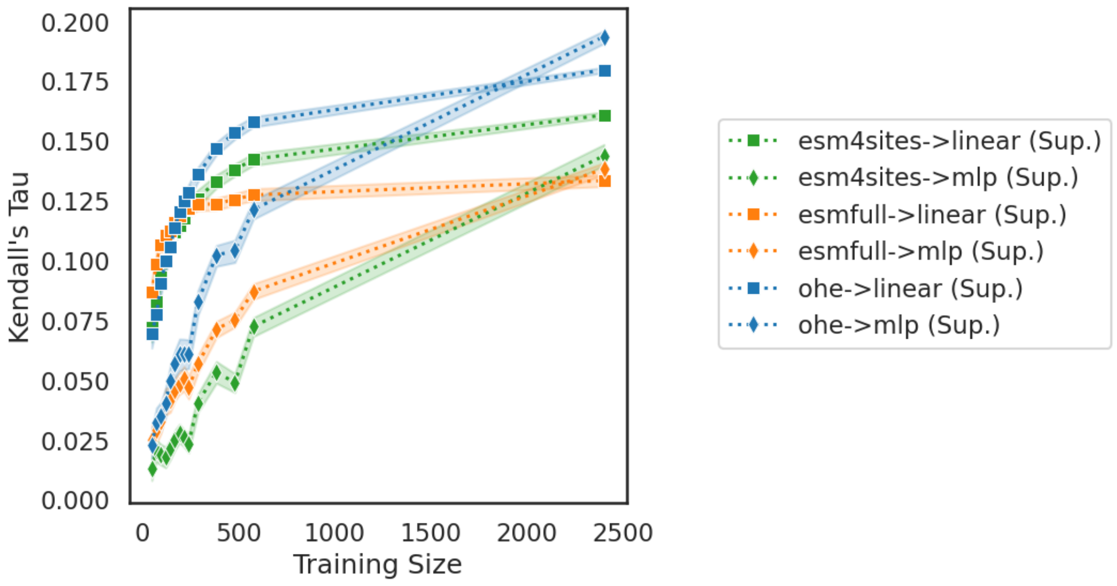

[11]:
```
# now compare linear to nonlinear models
metric = 'top10_in_plate'

fig, ax = plt.subplots(figsize=(6, 6))

for model, df in supervised_df.groupby('Model'):
    df_ = df[df['Metric'] == metric]
    color, linestyle, marker = color_map[model.split('_')[1]],␣
 ↪linestyle_map[model.split('_')[0]], marker_map[model.split('_')[2]]
    if model.split('_')[0] != 'none':
        continue

    name = df_['name'].iloc[0]
    sns.lineplot(x='Training Size', y='Score', data=df_, ax=ax,
                label=name, color=color, linestyle=linestyle,␣
 ↪marker=marker, markeredgewidth=2, markeredgecolor='black', alpha=0.7)
    # log scale training size
    ax.set_xscale('log')

ax.set_xlabel('training size')
ax.set_ylabel("top10 recovery, 1 96w-plate")
plt.legend(loc='upper right', bbox_to_anchor=(2.0, .8))
plt.savefig('nonlinear_vs_linear_top10.png', bbox_inches='tight', dpi=300)
```

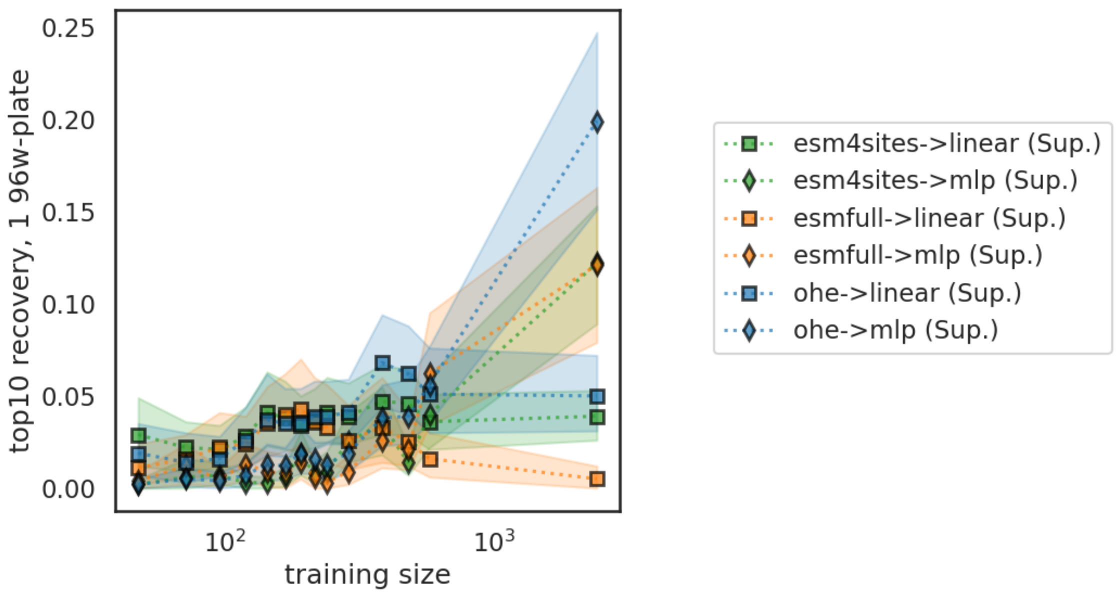

[12]:
```
joint_df = pd.concat([supervised_df, zs_df])
joint_df.to_csv('fig2_data.csv', index=False)
```

[ ]:
```
```

[ ]:
```
```
